# Alternative Polyadenylation Dynamics During the Rice Blast Immune Response

**DOI:** 10.1111/mpp.70301

**Published:** 2026-06-26

**Authors:** Dewei Yang, Xinyi Li, Haohua He, Niqing He, Qingshun Q. Li, Haihui Fu

**Affiliations:** ^1^ Rice Research Institute Fujian Academy of Agricultural Sciences Fuzhou Fujian China; ^2^ Sanming Academy of Agricultural Sciences Sanming Fujian China; ^3^ Key Laboratory of Crop Physiology, Ecology and Genetic Breeding, Ministry of Education Jiangxi Agricultural University Nanchang Jiangxi China; ^4^ Biomedical Sciences, College of Dental Medicine Western University of Health Sciences Pomona California USA

**Keywords:** alternative polyadenylation, miRNA targets, miRNA‐seq, PAT‐seq, post‐transcriptional regulation, rice blast

## Abstract

Alternative polyadenylation (APA) is a widespread post‐transcriptional regulation that generates transcripts with variable 3′ untranslated region (UTR) lengths. In this study, poly(A)‐tag sequencing (PAT‐seq) was performed during the process of *Magnaporthe oryzae* infection of rice. Genome‐wide dynamic changes of APA profiles were identified during the response, with widespread shortening of gene transcripts. Shortened genes were found to function in biological processes associated with stress responses. Importantly, the changes of 3′ UTR length were negatively related to the expression levels of the corresponding genes. In addition, we found that the expression levels of miRNAs that bind to the 3′ UTRs of the APA genes were negatively correlated with the expression levels of the corresponding APA genes. This correlation suggests a potential regulatory strategy where immune‐related genes might evade miRNA‐mediated repression via 3′ UTR shortening. To assess the biological impact of these APA dynamics, we functionally characterised a representative candidate, *Os05g0509500*, that exhibits complex and dynamic APA site switching upon infection. Knockout of *Os05g0509500* via CRISPR/Cas9 revealed its positive regulatory role in rice blast, offering initial insights into the function of APA in this disease. Several core polyadenylation protein factors were significantly differentially expressed during the rice response. Our results revealed that the precise transcriptome‐wide poly(A) site selection of genes during rice blast challenge, and the relationships between miRNAs and targeted APA genes, are tightly regulated. Such a mechanism provides a new perspective for designing novel strategies to control rice blast disease.

## Graphical abstract

In rice, infection by the blast fungus *Magnaporthe oryzae* triggers genome‐wide alternative polyadenylation (APA) dynamics, leading to widespread 3′ UTR shortening in stress‐responsive transcripts. This shortening enables immunity‐related genes to evade miRNA‐mediated suppression. This mechanism provides new molecular targets for engineering blast‐resistant rice varieties.

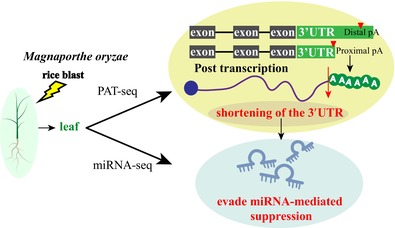

## 1 Introduction

Rice blast is a worldwide disease caused by the fungus *Magnaporthe oryzae* that affects the growth of rice ( *Oryza sativa* ) and manifests as empty and shrivelled grains, leading to a serious yield reduction (Dean et al. 2012). Moreover, it is associated with ecological problems such as environmental pollution due to the excessive use of pesticides in the rice field (Liu et al. 2021). The trend of current scientific research on combating this disease is to discover resistance genes, explain the genetic rules of rice blast occurrence, and cultivate resistant rice varieties. However, there is a lack of understanding of how rice can modulate dynamic gene expression networks at the post‐transcriptional level to boost host disease response.

Rice blast resistance relies on complex immune mechanisms, primarily PAMP‐triggered immunity (PTI) and effector‐triggered immunity (ETI), which ultimately converge on massive transcriptional reprogramming. Previous studies have identified numerous critical components in these pathways, ranging from signalling proteins and reactive oxygen species (ROS) regulators (Park et al. 2012; Maeda et al. 2016), to broad‐spectrum NBS‐LRR resistance genes (Kawasaki et al. 2006; Deng et al. 2017; Wang et al. 2018). The activation of these immune responses relies on ion channels and RNA‐binding transcription factors (Wang et al. 2019; Zhai et al. 2019), functioning within an integrated immune metabolic network (Zhai et al. 2022). Crucially, mounting a successful defence requires not only the initial activation of these signalling pathways but also the rapid, highly coordinated regulation of downstream gene expression at the post‐transcriptional level to ensure appropriate protein outputs.

Among post‐transcriptional regulatory mechanisms, the role of small RNAs in tuning rice blast resistance is increasingly evident. For instance, specific microRNAs (miRNAs) such as miR319, Osa‐miR398B, miR168, miR812w and Osa‐miR535 are dynamically responsive to *M. oryzae* infection, subsequently cleaving or inhibiting the translation of key target genes to modulate immune and developmental trade‐offs (Zhang et al. 2018; Li et al. 2019; Campo et al. 2021; Wang et al. 2021; Zhang et al. 2022). While the impact of miRNAs on immune responses is well documented, a critical unanswered question is how host defence‐related mRNAs can dynamically evade or succumb to this miRNA‐mediated repression during the arms race with pathogens. Because miRNA binding sites tend to be located in the 3′ end of transcripts, this dynamic evasion is largely governed by mRNA 3′ end processing, specifically alternative polyadenylation (APA).

The process of transcription of eukaryotic genes into mature mRNAs requires mRNA processing of 3′ ends, which involves cleavage and polyadenylation. The site of pre‐mRNA cleavage and polyadenylation, generally called the poly(A) site (or PAS), is determined by the interaction of the *cis*‐element (poly(A) signals) and a polyadenylation protein complex composed of cleavage and polyadenylation specificity factor (CPSF), cleavage stimulation factor (CstF), poly(A) polymerase (PAP), cleavage factor Im (CFIIm) and others (Lin and Li 2023). Selection of different poly(A) sites of transcripts from the same gene can lead to very different outcomes, a process called alternative polyadenylation (APA). Studies have shown that up to 70% of genes involved in environmental response and development in rice undergo APA (Shen et al. 2011). Systematic studies of 13 samples of different tissues and different stages of rice growth and development via 3′‐terminal sequencing technology reported that approximately 50% of genes undergo APA and that the highly expressed transcripts related to APA are enriched in important agronomic traits, such as blast disease resistance (Fu et al. 2016). In addition, comparison of differences in the growth and development process between the indica rice 93–11 and the japonica rice Nipponbare indicated the differential usage of the APA to confer resistance to bacterial blight (Zhou et al. 2019).

During biotic stress responses, APA serves as a rapid and highly effective regulatory strategy. APA selectively adds poly(A) tails to different regions of pre‐mRNA, producing transcripts with variable 3′ untranslated regions (3′ UTRs) or heterogeneous truncated proteins that participate in the physiological, biochemical and stress resistance processes of plants (Lin and Li 2023; Zhou and Li 2023; Yu et al. 2024). The most profound effect of APA during pathogen infection is the dynamic alteration of 3′ UTR length. The 3′ UTR contains numerous *cis*‐elements, most notably the binding sites for miRNAs and RNA‐binding proteins (Di Giammartino and Manley, 2014). Consequently, when a defence‐related gene shifts to use a proximal (closer to the 5′ end) PAS, it generates a shorter 3′ UTR. This transcript shortening provides a mechanistic advantage by allowing the mRNA to effectively escape miRNA‐mediated degradation or translational inhibition, thereby stabilising the transcript and boosting the production of immune‐related proteins. In contrast, the use of a distal PAS generates a longer 3′ UTR, subjecting the transcript to tighter negative regulation.

Recent studies indicated that changes in the concentration of polyadenylation factors are involved in PAS selections (Zhang et al. 2015). For example, mutation of the *Arabidopsis* CPSF30 causes significant changes in the PAS of thousands of genes, and the mutant exhibits a stronger ability to respond to antioxidants and alter bacterial resistance (Bruggeman et al. 2014; Liu et al. 2014). The nonstandard mRNA subtypes produced by PAS addition in the 5′ UTR, intron and coding sequence (CDS) regions are characteristic of abiotic stress processes, and these shorter subtypes are more stable and overexpressed (de Lorenzo et al. 2017). Another polyadenylation factor FIP1 is crucial for plant growth and development and the response of roots to abiotic stresses, while reduction of FIP1 activity reduces the use of poly(A) sites in these non‐3′ UTRs (Téllez‐Robledo et al. 2019). These studies indicate not only that poly(A) factors can regulate the selection of PAS but also that APA plays an important role in the process of plant stress responses.

To further elucidate the functional role of APA in rice blast resistance, we sought to address whether dynamic APA regulation contributes to gene expression reprogramming during pathogen infection, and how this process may interact with miRNA‐mediated post‐transcriptional regulation. We hypothesised that *M. oryzae* infection induces genome‐wide shifts in poly(A) site usage, leading to alterations in 3′ UTR length that modulate miRNA targeting efficiency and transcript stability.

To test this hypothesis, we performed poly(A) tag sequencing (PAT‐seq) to comprehensively profile poly(A) sites (PAS) and miRNA expression across a time course of *M. oryzae* isolate Guy11 infection in rice. We systematically analysed APA dynamics and their association with gene expression changes, and further examined the relationship between miRNA abundance and the expression of APA‐affected target genes.

We expected to uncover widespread, infection‐induced APA reprogramming events, particularly 3′ UTR shortening, that facilitate rapid transcriptional responses by evading miRNA‐mediated repression. Compared with previous studies on rice APA, this work provides a dynamic, genome‐wide insight of APA regulation during pathogen infection and reveals its coordinated interplay with miRNAs, thereby offering new mechanistic insights into post‐transcriptional regulation in plant immunity.

## 2 Results

### 2.1 Dynamic APA Profiles in Rice Upon *M. oryzae* Challenge

**FIGURE 1:**
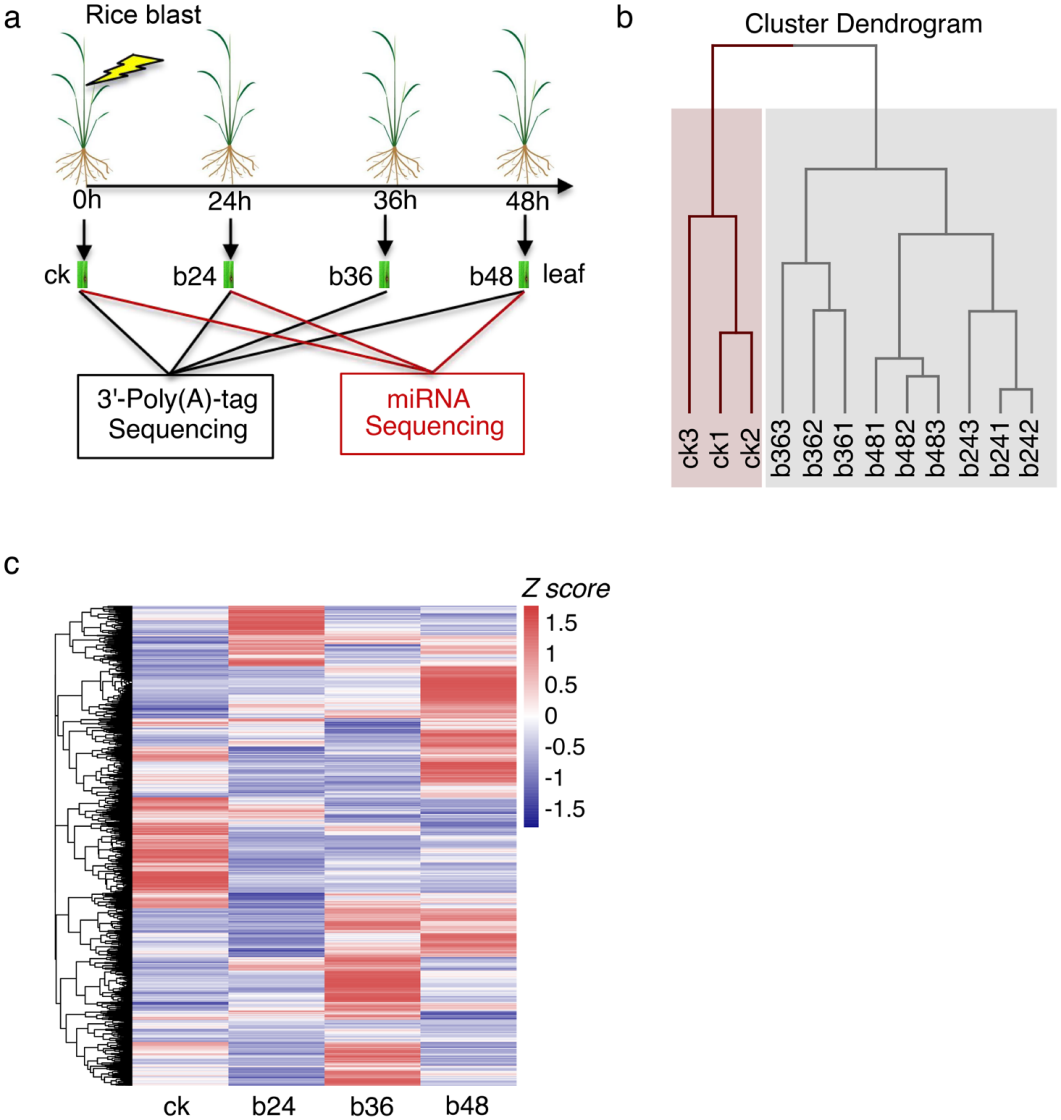
Overview of transcriptome sequencing of rice leave infected with *Magnaporthe oryzae* Guy11. (a) Design of the PAT‐seq and miRNA sequencing during rice blast infection time course. ck, control; b24 indicates sample 24 h after *M. oryzae* challenge, and so on. (b) Cluster of normalised reads among all samples. Three replicates are designed as 1, 2 and 3 after the sample name. (c) Heatmap showing the *Z* score values of RPM_dis_/RPM_pro_ (reads per million) during rice blast infection. Red colour represents the significant usage of the distal poly(A) sites (_dis_), and blue colour represents the usage of the proximal poly(A) sites (_pro_).

To investigate the role of APA in the rice blast response, total RNA from rice leaves at 0, 24, 36 and 48 h post‐infection (hpi) with *M. oryzae* Guy11 were extracted, made into libraries, and sequenced via PAT‐seq (Figure 1a). Different biological replicates were clustered on the same branch (Figure 1b), which was also confirmed via principal component analysis (Figure S1), indicative that the above data were reliable and could be used for subsequent analysis (Figure S2). Mapping the PAS coordinates onto genes revealed that 24,550 genes had two or more PAS, accounting for approximately 63% of the total number of genes. The normalised ratio heatmap of long and short transcripts of the same gene revealed significant changes in APA following infection at different time points (Figure 1c). These findings indicate that APA profiles change significantly and dynamically during the response to M. oryzae.

### 2.2 APA Causes Significant Increase of the Number of Shortened Transcripts Compared to Lengthened Ones During Rice Blast Response

**FIGURE 2:**
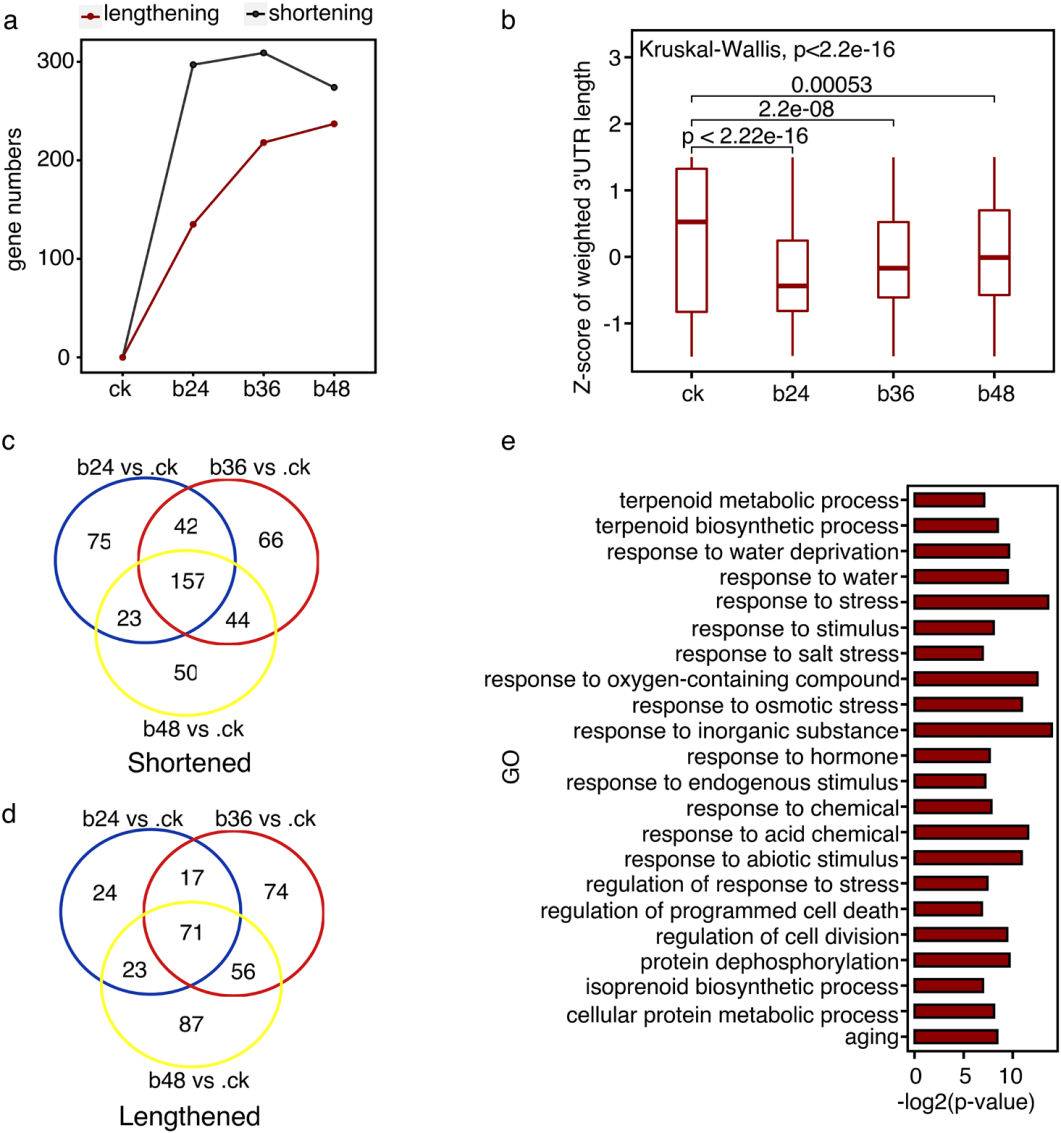
Transcriptome‐wide characteristics of the poly(A) site usages during rice blast response. (a) Number of genes with significant 3′ untranslated region (UTR) lengthening (red) or shortening (black) at 24, 36 and 48 h after *Magnaporthe oryzae* infection compared with the control (ck). (b) Global distribution of *Z*‐scores of weighted 3′ UTR length across the infection time course. The *p*‐values are based on the Kruskal–Wallis test. (c) Venn analysis of genes showing significant 3′ UTR shortening across the infection stages. The 157 overlapping genes represent a core set of genes that continue to shorten their transcripts throughout the fungal challenge. (d) Venn analysis of genes showing significant 3′ UTR lengthening. A total of 71 genes exhibited consistent lengthening across all three time points. (e) Gene Ontology (GO) enrichment of the core genes with shortened transcripts (the 157‐gene set).

To understand the involvement of APA in this response, we analysed the usage of the APA transcripts at different time points and found that many transcripts became longer or shorter and that the number of shorter ones was greater than the number of longer ones (Figure 2a). This finding was confirmed by 3′ UTR length analysis where the medians of the 3′ UTR length significantly turned lower after fungal challenge (Figure 2b). Venn analysis revealed that the 3′ UTRs of the transcripts of 157 genes continued to be shortened along over the infection time course (Figure 2c), while only 71 genes continued to be lengthened (Figure 2d). These 157 genes represent a responsive subset of APA genes, suggesting that their transcript shortening within a specific time range may be associated with the rice immune response. Further GO analysis revealed that genes with shortened 3′ UTRs were significantly enriched in biological processes related to rice blast infection (Figure 2e), including response to stress, response to stimulus and regulation of programmed cell death. In contrast, the 71 lengthened genes within a specific time range were primarily associated with basal plant growth and metabolic processes, such as seedling development, seed germination and cofactor metabolic processes (Figure S3). Detailed information regarding these shortened and lengthened genes, including their specific APA site coordinates, is provided in Tables S1 and S2.

Collectively, these results suggest that APA is probably involved in the regulation of gene expression during the rice blast response, although further studies are required to determine the extent to which these patterns represent a general feature of plant immunity.

### 2.3 APA Influences the Expression Levels of Rice Blast Response‐Related Genes

**FIGURE 3:**
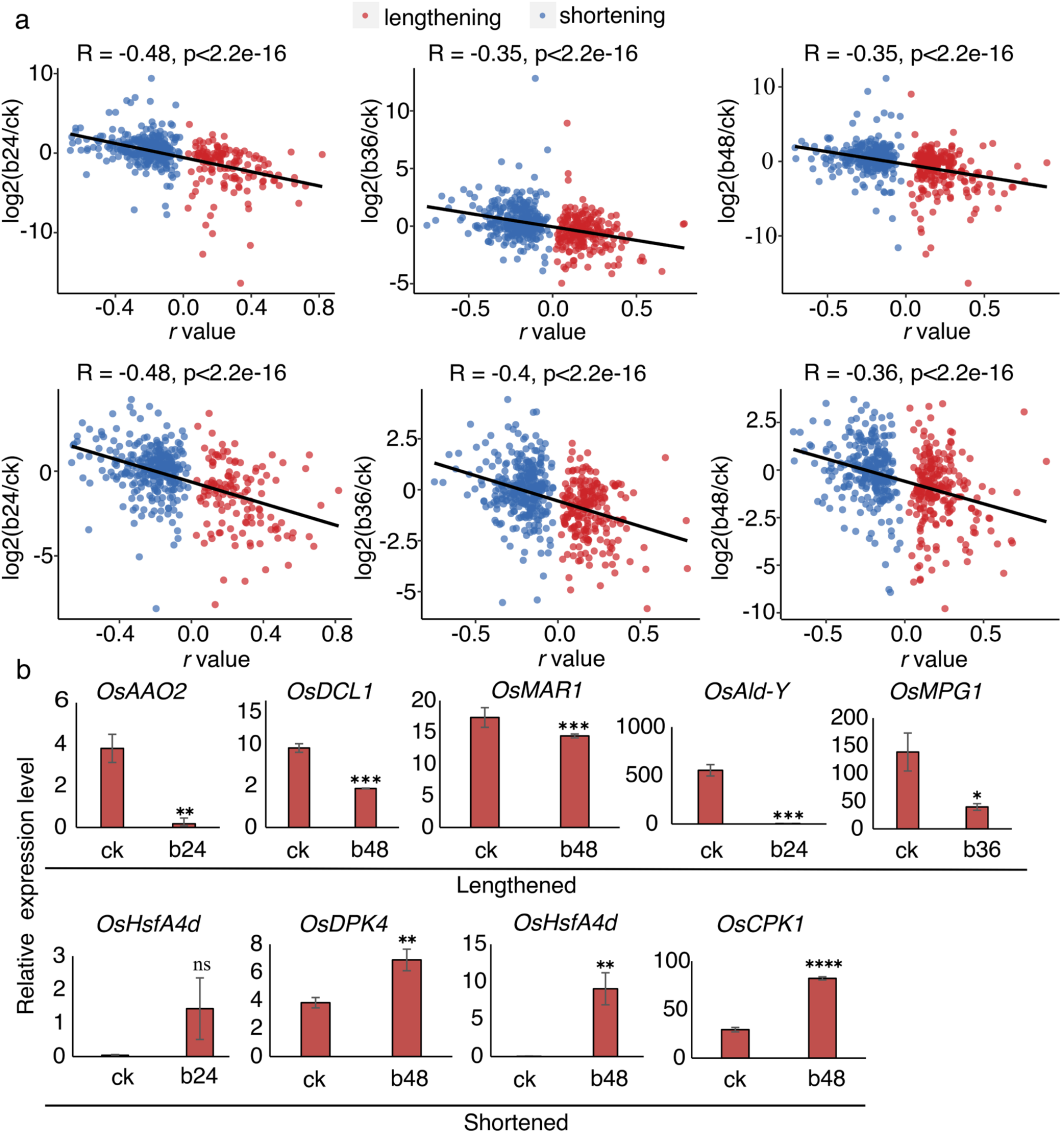
Relationships between the usage of poly(A) sites on lengthening or shortening transcripts and their corresponding gene expression levels. (a) Global negative correlation between alternative polyadenylation (APA) switching and gene expression changes. Scatter plots display the relationship between the 3′ untranslated region (UTR) length shift and the mRNA expression at 24, 36 and 48 h post‐inoculation (b24, b36, b48, respectively) compared to the control. The upper images are from RNA‐seq data, and the lower images are from PAT‐seq. (b) Some example genes related to stress response that were lengthened (top panels) or shortened (lower panels). Asterisks indicate significant difference compared with the control (ck) by means of *t* test; *p < 0.05, **p < 0.01, ***p < 0.001, ****p < 0.0001; *n* = 3.

Studies have shown that APA can affect the expression levels of genes (Lin and Li 2023; Yu et al. 2024). Therefore, by analysing the correlation trend at each specific time point between the changes of transcript length and the expression pattern of corresponding APA genes, we found that there was a significant negative correlation (Figure 3a). These findings indicate that APA affects the expression levels of the APA genes related to stress resistance, including *OsAAO2, OsDCL1*, *OsMAR1, OsAld‐Y* and *OsMPG1* in the lengthening group, and *OsHsfA4d, OsDPK4* and *OsCPK1* in the shortening group (Figure 3b).

### 2.4 APA‐Mediated Changes in 3′ UTR Length Affect miRNA Targeting That Regulate Gene Expression

**FIGURE 4:**
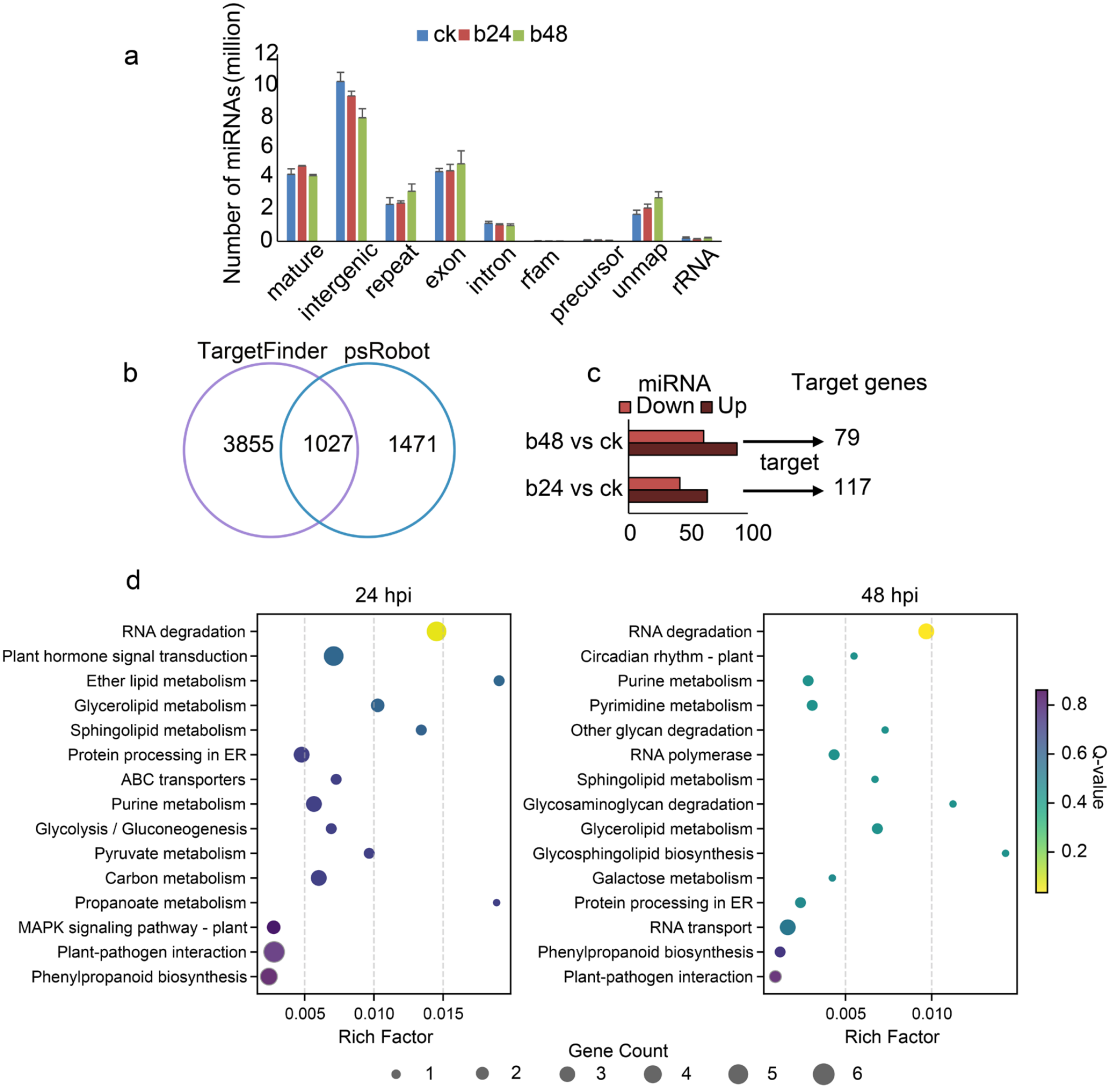
Profile of the expression patterns of miRNAs during response to infection with *Magnaporthe oryzae*. (a) Size distribution of small RNAs identified in rice leaves. (b) Overlap of predicted miRNA target genes identified by different algorithms. (c) Differential expression of miRNAs and their corresponding target genes during infection. ck, control; b24 and b48, 24 and 48 h post‐inoculation (hpi), respectively. (d) Functional enrichment analysis of differentially expressed miRNA target genes.

APA can cause changes in the length of 3′ UTR of the gene, thereby introducing or escaping the action of miRNA and further affecting the expression of the corresponding APA gene. To explore the association between miRNA regulation and APA gene expression, we performed small RNA sequencing on rice leaves at 0 (control), 24 and 48 hpi with *M. oryzae* (Tables S3 and S4). Small RNA profiling showed a dominant size distribution of 21 nucleotide (nt) species, consistent with typical miRNA populations in plants (Figure 4a; Figure S4). This sequencing process identified a wide range of known and predicted miRNAs, alongside numerous siRNAs and unannotated sRNA tags (Tables S5–, S8).

To investigate miRNA‐mediated regulation during infection, target genes were predicted using TargetFinder and psRobot, and high‐confidence targets were identified based on the intersection of both methods (Figure 4b; Table S9). Differential expression analysis revealed dynamic changes in miRNA expression at 24 and 48 hpi (Figure 4c).

Functional enrichment analysis indicated that the target genes of differentially expressed miRNAs were significantly associated with key rice blast‐related pathways, including plant–pathogen interaction, MAPK signalling and phenylpropanoid biosynthesis (Figure 4d). These results suggest that miRNA‐mediated regulation is closely linked to immune‐related transcriptional regulation during rice blast infection.

**FIGURE 5:**
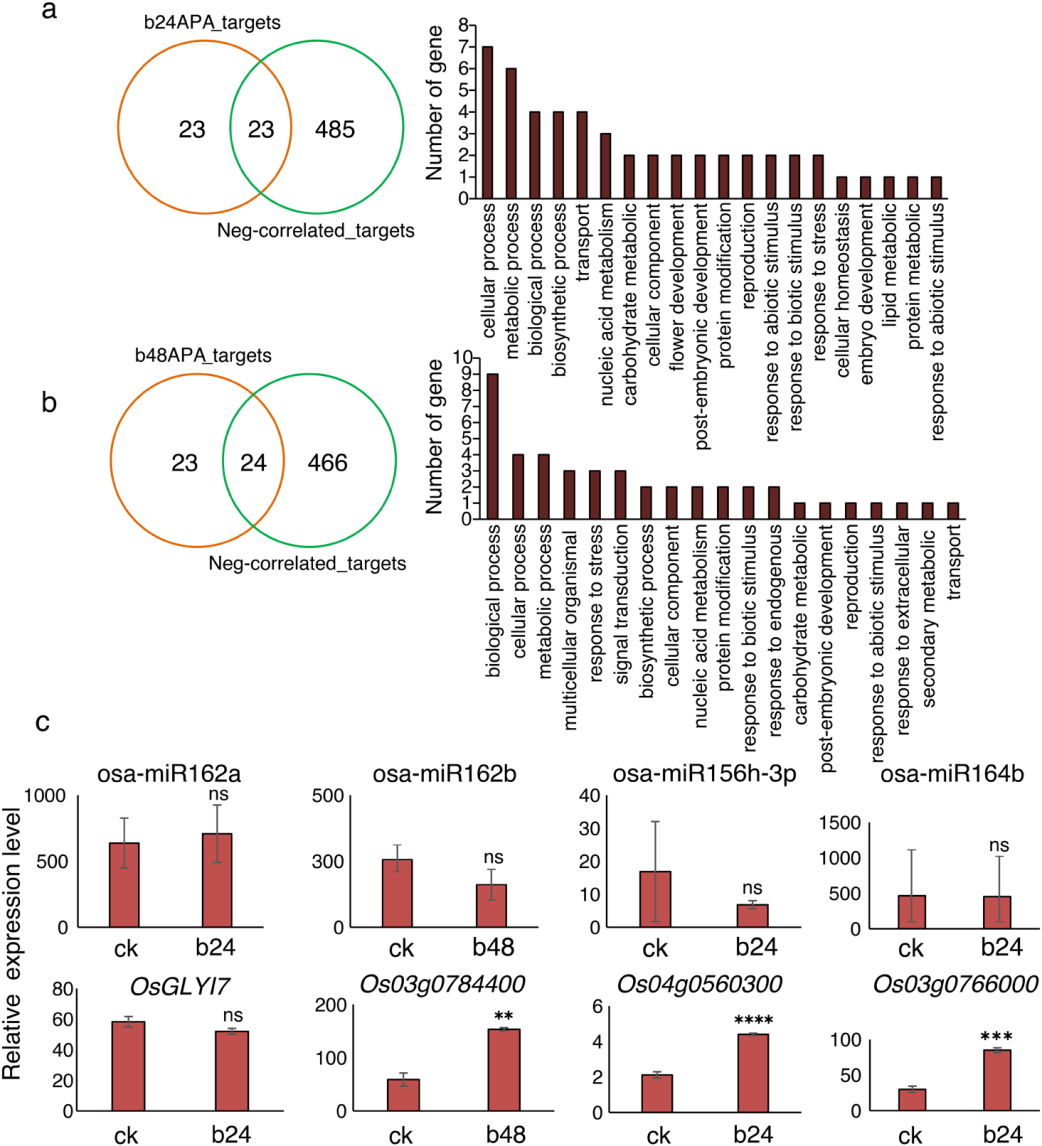
Correlations of the expression patterns of the miRNA targeted alternative polyadenylation (APA) genes. (a, b) Venn diagrams showing the intersection between APA genes targeted by miRNAs (red circle) and those exhibiting negative expression correlation with their corresponding miRNAs (green circle) at 24 (a) and 48 h post‐inoculation (b24 and b48, respectively) (b). The intersection represents genes where 3′ untranslated region (UTR) shortening likely leads to miRNA evasion and subsequent expression upregulation. The GO functions of the targeted genes (23 and 24) are shown on the right‐hand side. (c) Pairs of the negative correlation of the expression levels of miRNA (top) and their corresponding targeted APA genes (bottom). Asterisks indicate significant difference compared with the control (ck) by means of *t* test; **p < 0.01, ***p < 0.001, ****p < 0.0001; *n* = 3.

To further explore the association between APA gene expression and miRNAs, we predicted the 300 bp upstream and downstream of the target APA gene PAS and found that 1212 sequences were targeted by 45 miRNAs (Table S10). To further explore the association between APA gene expression and miRNAs, we assessed the relationship between miRNA expression and target mRNA levels. A significant general negative correlation was observed across all differentially expressed miRNA‐target pairs at 24 and 48 hpi (*R* = −0.33 and −0.43, respectively; Figure S5). To pinpoint the regulatory impact of APA, we overlapped these negatively correlated pairs with genes undergoing significant 3′ UTR switching. We identified 23 and 24 core candidates at 24 and 48 hpi, respectively, that exhibited both 3′ UTR shortening and the expected negative expression correlation with their targeting miRNAs (Figure 5a,b). Further GO functional annotation was performed on these negatively correlated APA genes, and some gene annotations were found for items such as response to biological stimuli, response to stress and response to endogenous stimuli. The negative expression relationship of some of the miRNA and their target pairs is shown in Figure 5c. To explicitly verify that this expression advantage is specifically driven by APA dynamics rather than transcriptional regulation, we quantitatively compared the absolute expression levels of miRNA‐targeted APA genes against miRNA‐targeted non‐APA genes. Notably, at both 24 and 48 hpi, the targeted APA genes maintained significantly higher expression abundances than the targeted non‐APA background (Figure S6).

To further investigate the mechanistic link between APA and miRNA evasion, we focused on two immunity‐related genes, *LOC_Os11g33120* (Zhao et al. 2024) (*OsRbohI*) and *LOC_Os08g44820* (Xiong et al. 2025) (*OsNTL5*), both of which underwent consistent 3′ UTR shortening. Our integrated analysis identified that specific miRNAs targeting these genes, such as osa‐miR156f‐5p (Zhang et al. 2020) (targeting *OsRbohI*) and osa‐miR159b (Chen et al. 2021) (targeting *OsNTL5*), were upregulated during *M. oryzae* infection. Bioinformatic mapping revealed that the target sites for these induced miRNAs are located exclusively within the alternative 3′ UTR segments (the region between the proximal and distal PAS). PAT‐seq data demonstrated that whereas the long transcripts (distal PAS usage) were prevalent in the control samples, their absolute abundance dropped upon infection as the proximal PAS became dominant (Figure S7). This structural shift physically eliminates the specific miRNA binding sites from the mature mRNA, allowing these critical defence genes to successfully escape post‐transcriptional repression and maintain robust expression despite the heightened abundance of targeting miRNAs.

These findings suggest that shorter 3′ UTRs may affect the expression level of genes by escaping miRNAs, thereby regulating the response to *M. oryzae*.

### 2.5 Differential Expression Patterns of Polyadenylation Core Protein Factors Could Modulate the Choice of Poly(A) Sites

**FIGURE 6:**
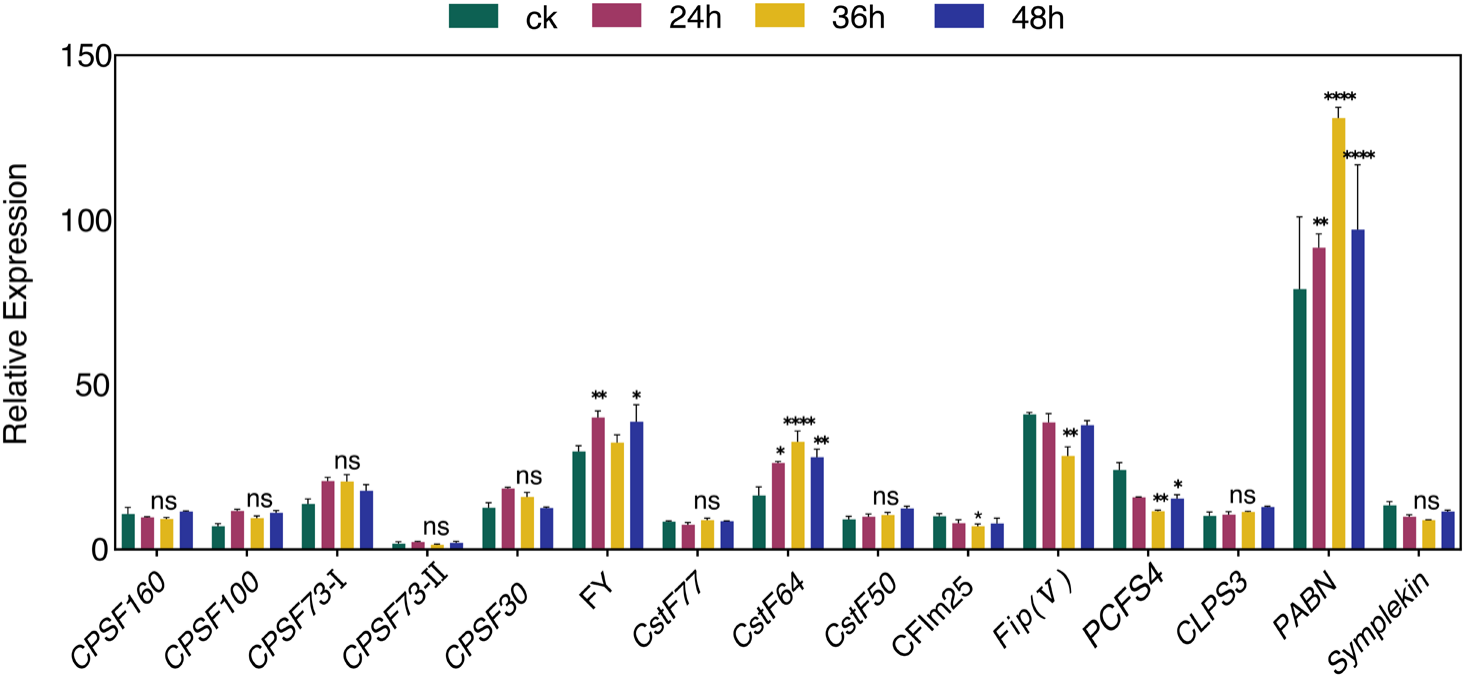
Expression patterns of 15 polyadenylation core factors across the response time point of *Magnaporthe oryzae* infection. The expression levels of polyadenylation core factors were analysed at 0, 24, 36 and 48 h post‐inoculation (hpi) time point in rice. The corresponding rice locus IDs for the gene abbreviations shown in the figure are as follows: *CPSF160*: Os04g0252200; *CPSF100*: Os09g0569400; *CPSF73‐I*: Os03g0852900; *CPSF73‐II*: Os09g0397900; *CPSF30:* Os06g0677700; *FY*: Os01g0951000; *CstF77*: Os12g0571900; *CstF64*: Os11g0176100; *CstF50*: Os03g0754900; *CFIm25*: Os08g0398800; *Fip(V)*: *Os03g0725100*; *PCFS4*: Os08g0187700; *CLPS3*: Os02g0217500; *PABN*: Os06g0219600; *Symplekin*: Os07g0693900. Asterisks indicate significant difference at sample time point in rice compared with the control (ck) by means of ANOVA; *p < 0.05, **p < 0.01, ****p < 0.0001; *n* = 3. With or without ns denotes that the expression level of the core factor at these time points showed no significant difference compared to the control (ck).

Studies have shown that the expression level of poly(A) factors can significantly change the use of PAS (Lin et al. 2017; Yu et al. 2019). Therefore, we analysed the expression levels of core poly(A) factors and found that many of their expression levels were significantly altered in the response to rice blast. *CFIm25*, *PCF11*, *FIP1* and *Symplekin‐C* were significantly downregulated during *M. oryzae* infection, whereas *CSTF64*, *CPSF73*, *FY* and *CPSF100* were significantly upregulated (Figure 6). *CPSF30* was significantly upregulated at 24 and 36 hpi but not significantly downregulated at 48 hpi. *CLP1* was continuously upregulated but only significantly upregulated at 48 hpi. The significant changes in the expression levels of these core factors suggest that many genes undergo APA changes during the occurrence of rice blast disease.

To understand the sequence determinants guiding these altered core factors in PAS selection, we performed a discriminative motif analysis (STREME) comparing the 100‐bp upstream and downstream regions of the preferred versus abandoned PAS. For genes undergoing 3′ UTR shortening, the downstream regions of the preferred proximal PAS were significantly enriched with U/GU‐rich motifs (e.g., YUUGUUYGCUB). These motifs function as canonical cleavage downstream elements (CDEs) recognised by CstF64. This perfectly aligns with our observation that CstF64 is significantly upregulated during infection, suggesting it drives transcript shortening by preferentially binding these proximal downstream elements. Conversely, for lengthened genes, the upstream regions of the preferred distal PAS were overwhelmingly dominated by highly conserved A/U‐rich canonical signals (e.g., AMAUACA, UGAAAMAW) (Figure S8). These results suggest that the dynamic APA switching during the immune response is strictly governed by the interplay between specific *cis*‐regulatory elements and the altered abundance of polyadenylation core factors.

### 2.6 The APA Gene *Os05g0509500* Positively Modulates Rice Resistance to *M. oryzae*

**FIGURE 7:**
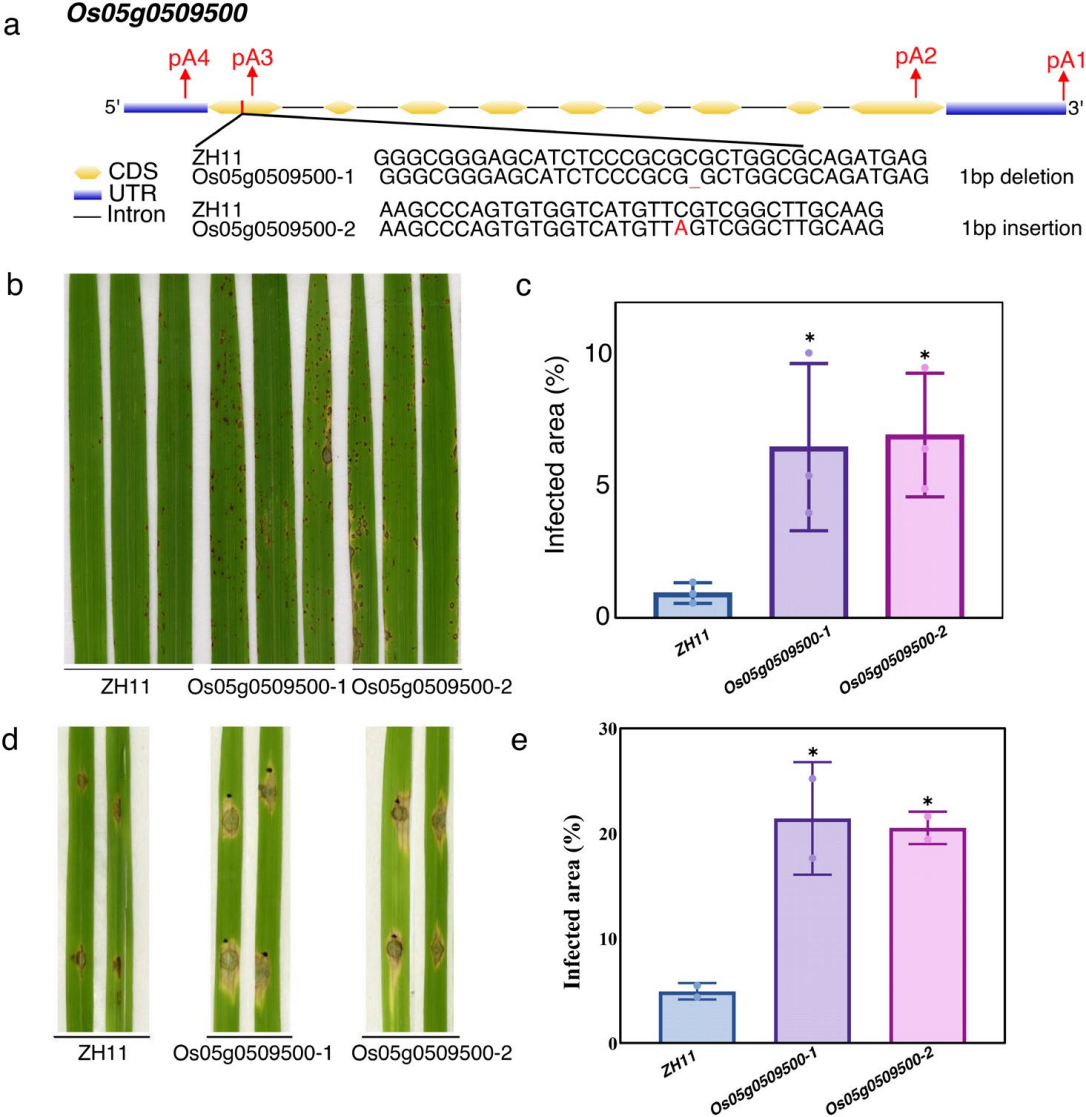
The impact of an alternative polyadenylation (APA) gene on performance on rice blast resistance. (a) Gene structure and mutation site of *Os05g0509500*. (b) Blast resistance of two independent *Os05g0509500* knockout lines compared with parental Zhonghua 11 (ZH11) plants. Three representative leaves photographed at 3 days post‐inoculation with the *Magnaporthe oryzae* isolate Guy11 are shown. (c) The proportion of disease spots on the leaves in (b) to the total area of the leaves was statistically analysed using ImagePro Plus v. 6.0. Asterisks indicate significant difference compared with the ZH11 by means of ANOVA; *p < 0.05; *n* = 3. (d) The same as (b) but spot inoculated and photographed at 4 days post‐inoculation. (e) The proportion of disease spots on the leaves in (d) to the total area of the leaves was statistically analysed using ImagePro Plus v. 6.0. Asterisks indicate significant difference compared with the ZH11 by means of ANOVA; *p < 0.05; *n* = 2.

The ultimate molecular effect of APA‐mediated transcript length changes is the alteration of the protein production of its gene. To validate the biological importance of genes undergoing significant APA during the rice blast response, we selected a candidate APA gene for functional validation via CRISPR/Cas9 knockout, and observed the resistance phenotype of the mutants to rice blast. Among the APA genes, the signal recognition particle gene *Os05g0509500* attracted our attention. Based on our PAT‐seq data, *Os05g0509500* possesses four APA sites: one in the 3′ UTR (pA1), two in the CDS (pA2 and pA3) and one in the 5′ UTR (pA4). During the *M. oryzae* infection time course, the usage of the canonical 3′ UTR PAS (pA1) dramatically increased, representing the overwhelmingly dominant and infection‐responsive isoform. Meanwhile, the non‐canonical sites (pA2–pA4) were used at much lower frequencies relative to the canonical PAS. Notably, the selection of these alternative sites was highly dynamic across the infection stages, remaining completely unused at certain time points while becoming detectable at others, which collectively reflects the complex APA regulation of this gene (Figure S9). To assess the involvement of *Os05g0509500* in rice blast resistance, we generated *Os05g0509500* knockout lines in the rice cultivar ZH11 background via CRISPR/Cas9 technology (primers listed in Table S11). Through screening and sequencing validations, we obtained two *Os05g0509500* mutants that contain a one‐base insertion (named *Os05g0509500–1* hereafter) or a one‐base deletion (named *Os05g0509500–2* hereafter) (Figure 7a), both disrupting the open reading frame of the gene. Under standard greenhouse conditions prior to inoculation, both mutant lines exhibited normal growth and development and were phenotypically indistinguishable from the wild‐type ZH11 plants in terms of plant architecture and leaf size.

We then inoculated the *Os05g0509500* mutants and ZH11 plants with the *M. oryzae* isolate Guy11. We found that, compared with ZH11, *Os05g0509500–1* and *Os05g0509500–2* were more susceptible to rice blast (Figure 7b,c). To further determine the phenotype of the *Os05g0509500* mutants, we used the method of inoculation in vitro. Leaves of 15‐day‐old plants of ZH11, *Os05g0509500‐1* and *Os05g0509500‐2* were perforated and inoculated (Guy11). The results revealed that, compared with wild‐type ZH11, *Os05g0509500‐1* and *Os05g0509500‐2* had larger plaques, indicative of being more susceptible to the fungal infection (Figure 7d,e). Thus, we concluded that *Os05g0509500* to some extent contributes to rice resistance to blast disease.

Notably, this functional validation is based on a single representative APA gene and therefore does not fully capture the genome‐wide complexity of APA‐mediated regulation during rice blast response. While this case to some extent provides supportive evidence linking APA dynamics to disease resistance, further functional characterisation of additional APA genes will be required to establish a more comprehensive mechanistic framework.

## 3 Discussion

Aiming to reveal the role of post‐transcriptional regulation in response to *M. oryzae* Guy11 in rice, we systematically characterised the profiles of APAs, miRNAs and comprehensively analysed the relationships between miRNAs and targeted APA genes' expression. It was revealed that global shortening of the 3′ UTR via APA on many genes occurred during the response to *M. oryzae* in rice, with 3′ UTRs being the shortest at 24 hpi.

### 3.1 The Shortening of Transcripts Mediated by APA Plays an Important Role in the Occurrence of Rice Blast Disease

Our comprehensive profiling revealed a clear transcriptome‐wide tendency toward proximal PAS usage during the rice blast response, resulting in a widespread shortening of 3′ UTRs in stress‐responsive genes. This active mRNA truncation parallels the APA phenomenon observed in the disease resistance gene *RIM2* (He et al. 2000). The specific enrichment of these shortened genes in stress and stimulus response pathways highlights the biological significance of APA as a rapid post‐transcriptional immune mechanism, allowing host plants to quickly adjust protein outputs without de novo transcription.

Previous studies have shown that many differentially expressed genes (DEGs) were also enriched in the abovementioned pathways during the occurrence of rice blast disease (Peng et al. 2023). The shortened transcripts mediated by APA are the reason for high protein expression in cancer cells because the shortened genes escape the regulation of miRNAs (Mayr and Bartel 2009). During the infection process by vascular stomatitis virus of macrophages, the genes tend to gradually shorten the 3′ UTR (Jia et al. 2017). Similarly, many genes in plants also adapt to biological stress and disease resistance through changes in 3′ UTR length. The PAS of *Xa1*, which encodes an NLR protein recognising transcription activator‐like effectors (TALEs), may be related to the difference in resistance to blast disease between the indica rice lines Nihon Haru and japonica rice lines 93–11 (Zhou et al. 2019).

However, the prevalence of shorter transcripts in response to rice blast stress observed in this study is not fully consistent with previous reports. For instance, a prior study based on APA trap analysis suggested that transcript lengthening events were more frequent following *M. oryzae* infection (Ye et al. 2019). Several factors may contribute to this discrepancy.

First, differences in rice genetic backgrounds may influence APA regulation under stress conditions. Distinct responses between indica and japonica subspecies have been reported for disease resistance loci, suggesting that genotype‐specific regulatory mechanisms may also affect APA dynamics.

Second, variation in pathogen strains may lead to different host transcriptional responses. In this study, the Guy11 strain was used, whereas previous work employed MoHrip1, which may trigger distinct signalling pathways and downstream regulatory programs.

Third, and importantly, differences in experimental and analytical approaches may substantially affect APA detection. Previous studies primarily relied on conventional RNA‐seq data combined with computational inference (e.g., APA trap), which may have limited resolution in precisely identifying poly(A) sites. In contrast, our study employed PAT‐seq, a method specifically designed to capture 3′ end cleavage and polyadenylation events, enabling more accurate and sensitive detection of APA dynamics.

Taken together, these technical and biological differences may jointly contribute to the observed divergence in APA patterns. Therefore, while our results highlight a potential role of transcript shortening during rice blast response, further comparative and experimental validation will be required to determine whether this represents a generalisable feature across different conditions.

### 3.2 APA Regulates Gene Expression Levels by Influencing miRNA Binding Sites

We detected a negative correlation between APA and the expression levels of corresponding genes, suggesting that the expression levels of these genes may be influenced by APA. A possible reason is that during the occurrence of rice blast disease, the related gene's APA leads to a shorter 3′ UTR, allowing the APA gene to escape the action of miRNA, resulting in a change in the expression level. The variable 3′ UTR of the APA gene predicts a certain number of miRNA binding sites, suggesting that these miRNAs may affect the target APA genes. Previous studies have shown that although only a small but certain number of miRNAs in plants target the 3′ UTR (Bonnet et al. 2004; Sunkar and Zhu 2004; Wang et al. 2004; Adai et al. 2005). For example, approximately 22% of miRNAs target 3′ UTRs, and 16.3% target 5′ UTRs (Archak and Nagaraju 2007) in rice. However, some of the binding miRNAs presented a significant negative correlation with the expression level of the corresponding target APA (Figure 5c), suggesting that miRNAs affect the expression level of the APA genes. Previous studies have also shown that the expression of miR395 in plants was influenced by environmental factors where external low sulfonate concentrations lead to the expression of miR395, while the expression level of the target gene APS1 decreases (Jones‐Rhoades and Bartel 2004). The expression level of miR397b.2 was induced by heat, and after heat induction, the oxidation of its target gene l‐ascorbate was downregulated (Jeong et al. 2011).

We predicted some miRNA binding sites in the variable 3′ UTR of APA genes during *M. oryzae* infection, potentially impacting of miRNA binding on the expression level of the target APA genes in the 3′ UTR. Previous studies showed that the silencing of miR156 does not affect the yield but enhances resistance (Zhang et al. 2020). Osa‐miR167 regulates the immunity of rice to *M. oryzae* through the target gene *ARF12* (Zhao et al. 2020). Osa‐miR169s regulate rice blast resistance through the target gene NF‐YA (Li, Zhao, et al. 2017). Osa‐miR162a can balance rice yield and resistance to *M. oryzae* and yield by precisely regulating the expression level of *DCL1* (Li et al. 2020). In our study, we provided structural evidence for this evasion mechanism. For instance, key immunity genes like *OsRbohI* (Zhao et al. 2024) and *OsNTL5* (Xiong et al. 2025) physically lose the binding sites for upregulated miRNAs (e.g., osa‐miR156f‐5p and osa‐miR159b, respectively) (Zhang et al. 2020; Chen et al. 2021) by switching to proximal PAS during infection, ensuring their robust expression. Thus, the regulation of these APA genes by miRNA identified herein is highly probable.

However, a limitation of the current study is that this proposed miRNA evasion mechanism is primarily derived from the association of transcriptome‐wide correlative omics data. While functional validation of specific candidates like *Os05g0509500* supports the biological importance of APA, further direct biochemical validations will be required to definitively confirm these specific miRNA–APA interactions. In addition, more validation of APA genes will also be required to confirm support for broad genome‐wide mechanistic studies in rice blast.

### 3.3 Significant Changes in the Expression Levels of Core APA Factors Contribute to Length Changes in Related Genes in Response to Rice Blast

Previous studies have shown that the abundance of core polyadenylation factors has a significant effect on the selection of downstream regulated genes PAS (Takagaki et al. 1996; Kubo et al. 2006; Yang et al. 2011; Yao et al. 2012; Li et al. 2015; Song et al. 2021). Among them, the CPSFs play an important role. Inactivation of CPSF30 results in a significant impact (≥ 45%) on the APA of the 3′ UTR of genes, and it is mainly responsible for recognising poly(A) signals like AAUAAA (Thomas et al. 2012). In *Arabidopsis*, CPSF30‐L (a longer version of the gene coded by *CPSF30*) can affect APA in the 3′ UTR of *NRT1.1* mRNA, leading to nitrogen uptake phenotypes (Li et al. 2017). The hypomorphic mutation of the *AtCPSF100* gene leads to global changes in the APA profile in *Arabidopsis*, which is sensitive to bacterial infection (Lin et al. 2017). This study revealed significant changes in the expression levels of several important APA core factors. For example, the expression level of *CFIm25* is significantly downregulated during *M. oryzae* infection. Previous studies have shown that many genes tend to use the proximal PAS after interference with the expression of this factor (Masamha et al. 2014), indicating that the downregulation of this factor during rice blast infection has a significant effect on the use of proximal PAS in many genes.

CstF64 can bind to the downstream sequence in animals, and overexpression of this factor promotes the use of the proximal PAS (Takagaki et al. 1996). This factor was significantly upregulated in this study, which perfectly coincides with our STREME analysis showing strong enrichment of GU‐rich motifs exclusively downstream of the used proximal PAS in shortened transcripts. This suggests a coordinated mechanism where upregulated CstF64 binds to proximal DSEs to drive transcriptome‐wide 3′ UTR shortening. On the other hand, the lengthened transcripts possessed robust canonical A‐rich signals at their distal PAS, allowing them to resist the shortening trend, possibly facilitated by the concurrent downregulation of factors. Deficiency of *CPSF30*‐L leads to elongation of the 3′ UTRs of related transcripts, accelerating their mRNA degradation and leading to delayed and abscisic acid hypersensitivity reactions (Song et al. 2021). Interestingly, in this study, the expression level of *CPSF30* was increased at 24 and 36 hpi, indicating that the upregulation of this factor in the early stages of *M. oryzae* infection may have an important impact on the shortening of some genes.

On the other hand, knockout of *FY* leads to the use of a large number of proximal PAS in genes (Yu et al. 2019). This gene is significantly upregulated in the rice blast response in the current study, indicating that *FY* may contribute to the elongation of related genes in the occurrence of rice blast. The *PCF11 (PCFS4)* knockout line has a large number of distal PAS (Boss et al. 2004; Li et al. 2015), whereas the expression level of this factor is significantly downregulated during *M. oryzae* infection, indicating that the downregulation of this factor strongly contributes to the elongation of related genes in the occurrence of rice blast.

In summary, significant changes in the expression levels of these polyadenylation core factors have led to significant changes in the length of APA genes during the occurrence of rice blast disease. Unravelling these post‐transcriptional regulatory networks not only advances our fundamental understanding of plant immunity but also offers broader implications for agriculture. Exploiting APA dynamics provides potential genetic targets for breeding broad‐spectrum disease‐resistant rice varieties.

## 4 Experimental Procedures

### 4.1 *Magnaporthe oryzae* Isolate

The *M. oryzae* isolate Guy11 was provided by the State Key Laboratory for Ecological Pest Control of Fujian and Taiwan Crops, Fujian Agriculture and Forestry University. It was isolated via the single‐spore separation method and preserved on sterilised rice stems in a freezer at −20°C.

### 4.2 Inoculation and Evaluation of Resistance

Rice seedlings at the three‐ to four‐leaf stage were inoculated according to a slightly modified procedure from previous investigation (Lii, Xu, et al. 2020). In a high‐humidity chamber, 25 mL of *M. oryzae* Guy11 conidial suspension (10^5^ conidia/mL) was evenly applied to the foliage using an air compressor‐driven airbrush. Following inoculation, the plants were incubated in the dark at 25°C for 24 h inside a plastic chamber containing wet sponges, maintaining 95%–100% humidity. Subsequently, the inoculated seedlings were translocated into a greenhouse at 25°C–28°C for 3–4 days (Lii, Xu, et al. 2020). The ability to resist rice blast disease was evaluated using the method described previously (Wei et al. 2013). Leaves were sampled at 0, 24, 36 and 48 hpi for subsequent study.

### 4.3 RNA Isolation and Poly(A) Tag Sequencing

Total RNA isolation was performed according to the TRIzol (Invitrogen) protocol, and the RNA was purified with DNase I (Qiagen). Total RNA was subsequently used for PAT‐seq library construction as described in our previous study (Téllez‐Robledo et al. 2019). Briefly, 5 μg of total RNA was fragmented in 5× fragmentation buffer at 94°C for 4 min, and the poly(A) fragments were enriched via oligo(dT) 25 beads (NEB). Next, the fragmented RNA was reverse transcribed, and the first cDNA chain was synthesised via SMART Scribe (Clontech) and a 3′ adaptor. SMART Scribe and a 5′ adaptor were subsequently added. Double‐stranded cDNA was purified with AMPure Beads (BECKMAN Agencourt). Next, the cDNA was used for 18 cycles of amplification. Finally, the library was checked via 2% gel electrophoresis, and 300–500‐bp fragments were purified via a Zymo gel purification kit. The library was subjected to Illumina HiSeq 2500 sequencing (BGI, Shenzhen).

### 4.4 Analysis of 3′‐Terminal Sequencing Data

The raw reads with Q‐scores > 20 were processed using the FASTX Toolkit to remove adapter and poly(A) sequences. Clean reads were aligned to the japonica rice reference genome using Bowtie2 (v. 2.1.0). Only uniquely mapped reads were retained for downstream analysis, and internal priming events were filtered out.

According to previous research (Wu et al. 2011), where poly(A) tags located within a 24 nt window were merged into a single PAC. PACs supported by more than 10 total tags across all samples were retained. Genes containing at least two PACs were defined as APA genes.

The reads of PAC across samples were normalised, PCA was performed via DESeq2 (Love et al. 2014), and clustering analysis was conducted using factoextra. To visualise relative usage patterns, the ratio of distal to proximal PAC usage (RPM_dis_/RPM_pro_) was calculated for each gene, and Z‐score normalisation was applied prior to heatmap construction using the R package heatmap.

To identify shortening and lengthening events, genes with at least two poly(A) sites (PACs) in their 3′ UTRs were analysed. For each gene, a weighted mean 3′ UTR length was calculated based on poly(A) site usage (PSU). Changes in 3′ UTR length between conditions were quantified using Pearson's correlation coefficient (*r*) (Lin et al. 2020), where *r* < 0 indicates 3′ UTR shortening and *r* > 0 indicates 3′ UTR lengthening. Statistical significance was assessed using chi‐square tests on PAC usage differences, followed by multiple testing correction. Genes with adjusted *p* < 0.05 were defined as significantly shortened or lengthened.

The differential expression levels of the genes were determined via DESeq2. GO and KEGG enrichment of APA genes was performed via the R package cluster Profiler, the entire *Oryza sativa* genome was used as the background gene universe, and GO annotations of some APA genes were carried out via an online website (Rice Genome Annotation Project funded by the NSF). To account for multiple testing, *p*‐values were adjusted using the Benjamini–Hochberg (BH) method to control the false discovery rate (FDR). An adjusted *p*‐value (*q*‐value) < 0.05 was used as the significance cut‐off for the KEGG and GO enrichment results.

### 4.5 3′ UTR Weight Length Calculation

We selected genes with two or more PACs and calculated the length from each PAC (poly(A) site peak) to the termination codon. We then multiplied the 3′ UTR length of each PAC by the number of reads in each sample, summed, and divided it by the reads of all samples for that gene. Due to the large fluctuation range of the 3′ UTRs of different genes, the *Z* score of the effective 3′ UTR length of each gene across samples was computed.

### 4.6 Production of miRNA‐Seq Libraries

Briefly, an approximately 18–30 nt RNA segment was isolated via PAGE, and the 5‐adenosine modified, 3‐shut single‐stranded DNA linker was then connected to the 3′ end of the selected small RNA from the above step. Second, the RT primer was added to the solution from the above step and cross‐linked to the 3′ linker of the RNA, and the excess free 3′ linker was added. Next, the 5′ adaptor was linked to the 5′ end of the product above, and the RT primer was reverse extended to synthesise strand cDNA. The cDNA was amplified by high‐speed DNA polymerase and enriched with both 3′ and 5′ adaptors. The 100–120 bp PCR products were separated via PAGE. Finally, the Thermo Fisher Qubit Micro RNA Assay kit (Q32880) was used for library quantification, followed by pooled cyclisation.

### 4.7 Analyses of miRNA‐Seq Data

After we obtained the raw sequencing data, the raw data were filtered (including removing tags with low sequencing quality, with 5′ adapter contamination, without a 3′ linker sequence, without the inserted fragment, containing poly(A), and smaller than 18 nt, as well as determining the length distribution of small RNA fragments), and clean tags were obtained. Clean reads were mapped to the reference genome and other small RNA databases via the software AASRA. Next, the comparisons and annotations of all the sRNAs and various RNAs were summarised. For each unique sRNA to have a unique annotation, the sRNA was traversed through the annotation in the order of priority: MiRbase > pirnabank > snoRNA (human/plant) > Rfam>other sRNA. Tags matching miRBase with no more than two mismatches were identified as known miRNAs. Sequences that could not be assigned to any known ncRNA category were defined as unannotated sRNA tags. Novel miRNAs were predicted using miRA with the following stringent criteria to avoid false positives: (i) candidates must fold into a stable hairpin secondary structure with a calculated minimum free energy (MFE); (ii) the sequences must exhibit clear read stacking patterns, with reads primarily concentrated on the mature and star (miRNA/miRNA*) strands rather than the loop or flanking regions (Evers et al. 2015). TPM was used to standardise the expression levels of small RNAs, and DEGseq was used to analyse the differential expression of small RNAs in different samples. PsRobot (‐gl 17 ‐p 8 ‐gn 1) and TargetFinder (‐c 4) were used for target gene prediction, and the intersection was subsequently analysed.

### 4.8 Analysis of APA Gene Targets

The 300‐bp nucleotide sequence of the 3′ UTR upstream of the PAS of the APA‐transformed gene was intercepted for target prediction via TargetFinder (‐c 4). Correlation analysis was conducted between miRNA expression levels and targeted APA gene expression levels.

### 4.9 Motif Enrichment Analysis of Alternative PAS

To identify regulatory sequence motifs associated with PAS selection, sequences spanning 100 bp upstream and 100 bp downstream of both the proximal and distal PAS were extracted using bedtools. Discriminative de novo motif discovery was performed using STREME from the MEME Suite (Bailey 2021). The sequences of the preferentially used PAS (e.g., proximal PAS for shortened genes) were set as the primary input, while the sequences of the abandoned PAS (e.g., distal PAS for shortened genes) were used as the background control. The analysis was configured to search for RNA motifs with a minimum width of 5 and a maximum width of 12, applying a significance threshold of *p* < 0.05.

## Author Contributions

**Xinyi Li:** conceptualization, visualization, data curation, validation. **Haohua He:** formal analysis, data curation. **Qingshun Q. Li:** writing – review and editing, project administration, funding acquisition, supervision. **Niqing He:** validation. **Haihui Fu:** writing – original draft, methodology, resources, project administration, funding acquisition, software. **Dewei Yang:** writing – original draft, validation, conceptualization, investigation, funding acquisition.

## Funding

This work was supported by the Special Fund for Agro‐scientific Research in the Public Interest of Fujian Province (No. 2024R1055), the National Natural Science Foundation of China (32360650, 32060045, 32402387, 32270344), the National Natural Science Foundation extension research project (No. GJYS05009), the 5511 Collaborative Engineering Project (No. KXXYJBG0021) and the 100 Talent Plan of Fujian Province, Key Project of Fujian Provincial Natural Science Foundation (2025J02027) and grants (jxsq2019101057) from the Double Thousand Plan of Jiangxi Province.

## Conflicts of Interest

The authors declare no conflicts of interest.

## Supporting information


**Figure S1:** Primary component analysis of PAT‐seq libraries of the RNA samples from rice blast infected leaves (refer to Figure 1a,b).

**Figure S2:** Pair‐wise comparisons of correlation across replicated samples at each time point, indicative of good biological repetition of the results. Refer to Figure 1a for sample names.

**Figure S3:** Gene Ontology (GO) enrichment analysis of the 71 continuously lengthened genes.

**Figure S4:** Length distribution of small RNAs across all sequencing samples.

**Figure S5:** Relationship of the expression between APA genes and targeting miRNA. Fc1 denotes the expression pattern of RNA‐seq and fc2 denotes the expression pattern of miRNA‐seq.

**Figure S6:** Quantitative comparison of absolute expression abundances between miRNA‐targeted APA genes and miRNA‐targeted non‐APA genes at 24 and 48 h post‐inoculation (hpi).

**Figure S7:** Dynamics of alternative polyadenylation and miRNA evasion in key immune genes. Structural models and expression profiles for (A) *OsRbohD* (*LOC_Os11g33120*) targeted by osa‐miR156f‐5p and (B) *OsNTL5* (*LOC_Os08g44820*) targeted by osa‐miR159b.

**Figure S8:** Discriminative sequence motif analysis (STREME) of the 100‐bp flanking regions surrounding the preferred versus abandoned alternative poly(A) sites.

**Figure S9:** Dynamics of alternative poly(A) site usage for the *Os05g0509500* gene across the *Magnaporthe oryzae* infection time course.

**Table S1:** List of the 157 continuously shortened genes and their alternative poly(A) site coordinates during the *Magnaporthe oryzae* infection time course.

**Table S2:** List of the 71 continuously lengthened genes and their alternative poly(A) site coordinates during the *Magnaporthe oryzae* infection time course.

**Table S3:** Alignment statistics of tags aligned to the reference genome.

**Table S4:** Summary of sequencing data for each sample.

**Table S5:** Known miRNAs across all samples.

**Table S6:** Unknown miRNAs across all samples.

**Table S7:** Predicted miRNAs across all samples.

**Table S8:** Predicted siRNAs across all samples.

**Table S9:** Intersection target genes of miRNAs predicted by TargetFinder and psRobot.

**Table S10:** miRNAs that bind to APA genes as predicted by psRNATarget.

**Table S11:** Sequences of primers used for synthesising gRNA spacers and genotyping CRISPR‐edited mutants.

## Data Availability

The raw sequencing data (PAT‐seq and miRNA‐seq) generated in this study have been deposited in the Genome Sequence Archive (GSA) at the National Genomics Data Center (NGDC), Beijing Institute of Genomics, Chinese Academy of Sciences/China National Center for Bioinformation (CNCB) under the BioProject accession number PRJCA064196.
