# Supplementary material for: Alternative Polyadenylation Dynamics During the Rice Blast Immune Response
Source: Mol Plant Pathol. 2026 Jun 26;27(7):e70301. doi: 10.1111/mpp.70301 (PMC13305335; doi:10.1111/mpp.70301)
Supplement: Supplementary file 1 — Figure S1: Primary component analysis of PAT‐seq libraries of the RNA samples from rice blast infected leaves (refer to Figure 1a,b). [file MPP-27-e70301-s005.pptx]

## Slide 1
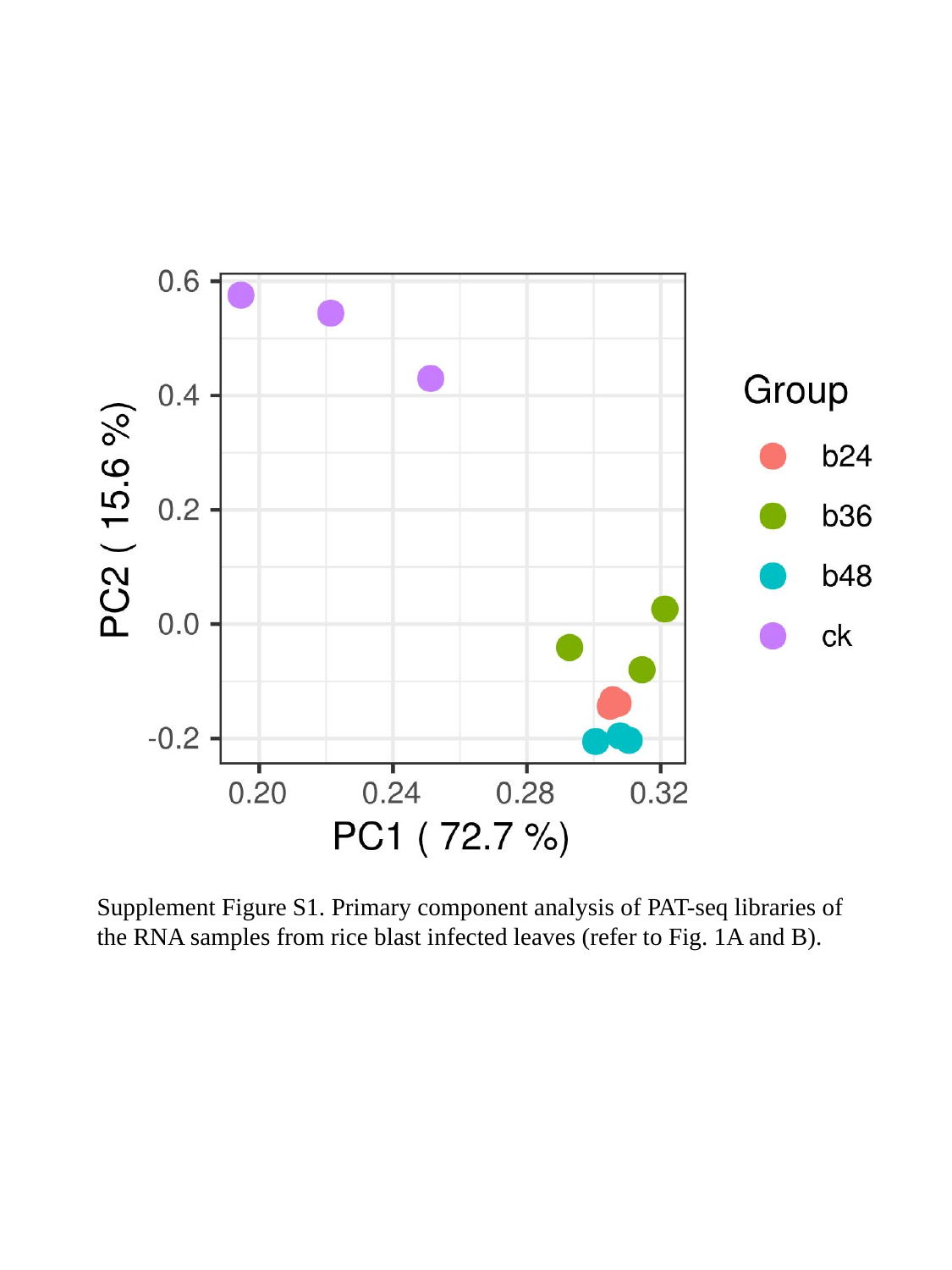

Supplement Figure S1. Primary component analysis of PAT-seq libraries of the RNA samples from rice blast infected leaves (refer to Fig. 1A and B).
