# Supplementary material for: Alternative Polyadenylation Dynamics During the Rice Blast Immune Response
Source: Mol Plant Pathol. 2026 Jun 26;27(7):e70301. doi: 10.1111/mpp.70301 (PMC13305335; doi:10.1111/mpp.70301)
Supplement: Supplementary file 2 — Figure S2: Pair‐wise comparisons of correlation across replicated samples at each time point, indicative of good biological repetition of the results. Refer to Figure 1a for sample names. [file MPP-27-e70301-s011.pptx]

## Slide 1
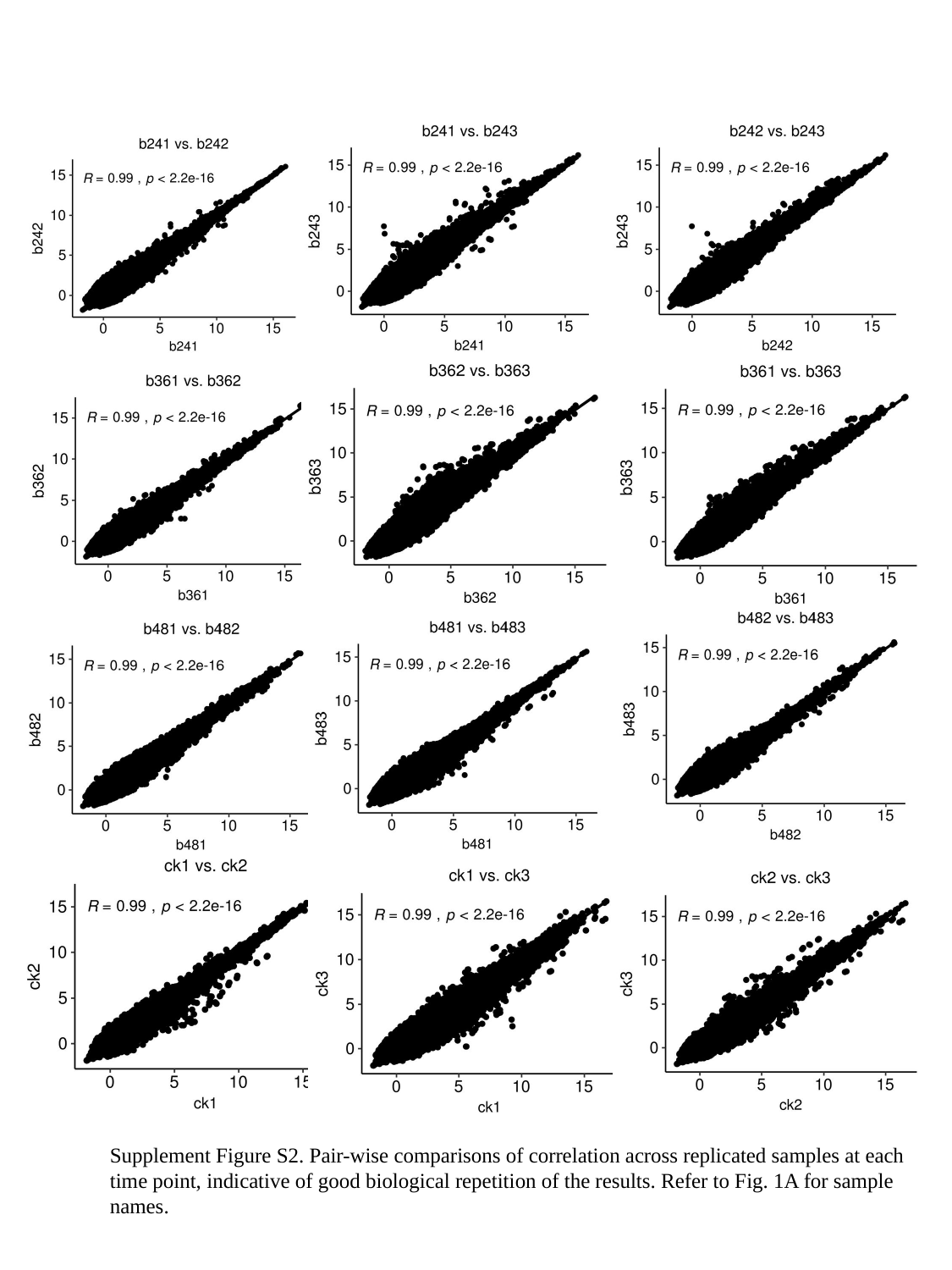

Supplement Figure S2. Pair-wise comparisons of correlation across replicated samples at each time point, indicative of good biological repetition of the results. Refer to Fig. 1A for sample names.
