# Supplementary material for: Alternative Polyadenylation Dynamics During the Rice Blast Immune Response
Source: Mol Plant Pathol. 2026 Jun 26;27(7):e70301. doi: 10.1111/mpp.70301 (PMC13305335; doi:10.1111/mpp.70301)
Supplement: Supplementary file 3 — Figure S3: Gene Ontology (GO) enrichment analysis of the 71 continuously lengthened genes. [file MPP-27-e70301-s008.pptx]

## Slide 1
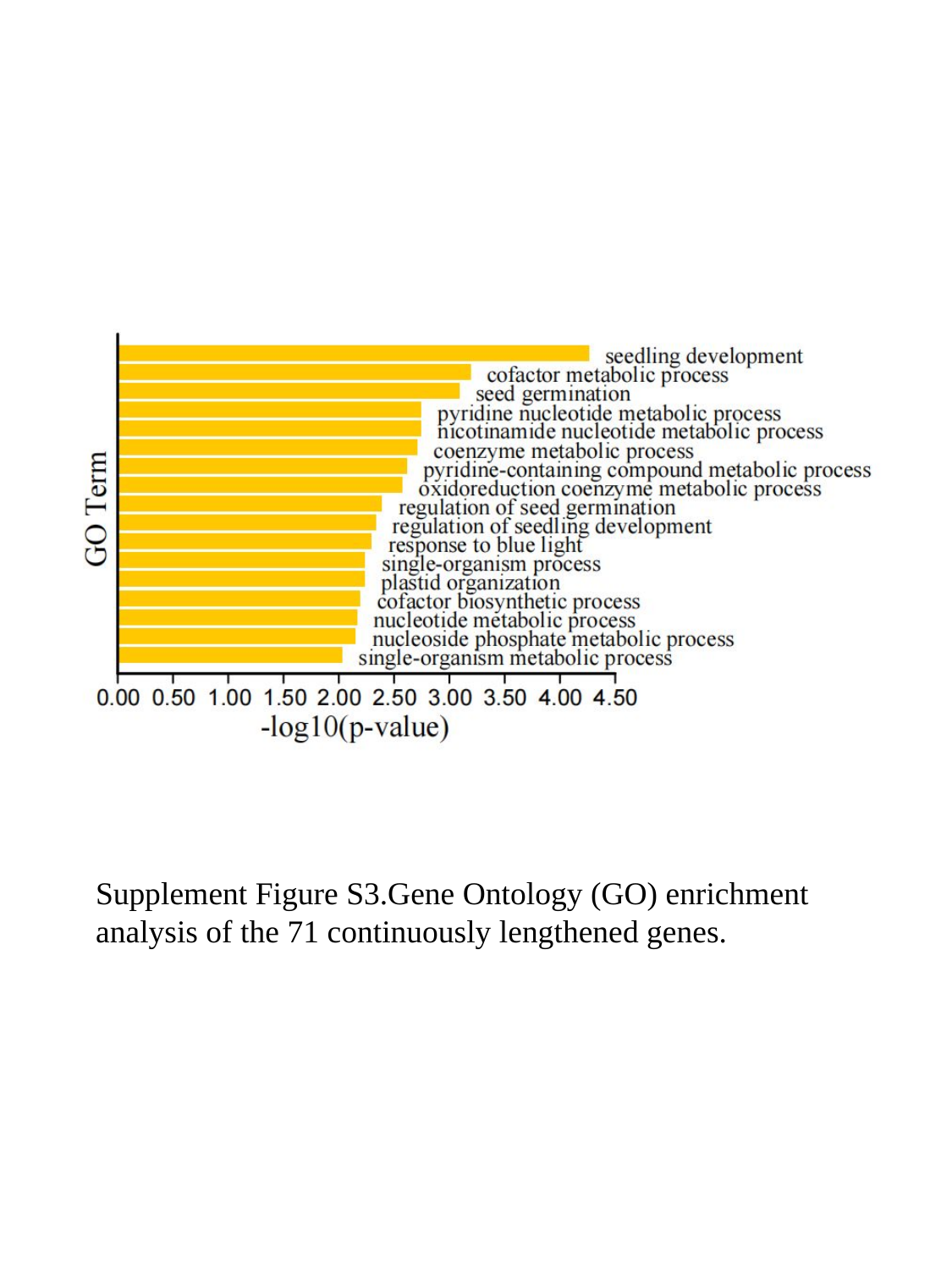

Supplement Figure S3.Gene Ontology (GO) enrichment analysis of the 71 continuously lengthened genes.
