# Supplementary figures and images for: Alternative Polyadenylation Dynamics During the Rice Blast Immune Response
Source: Mol Plant Pathol. 2026 Jun 26;27(7):e70301. doi: 10.1111/mpp.70301 (PMC13305335; doi:10.1111/mpp.70301)

## Slide 1
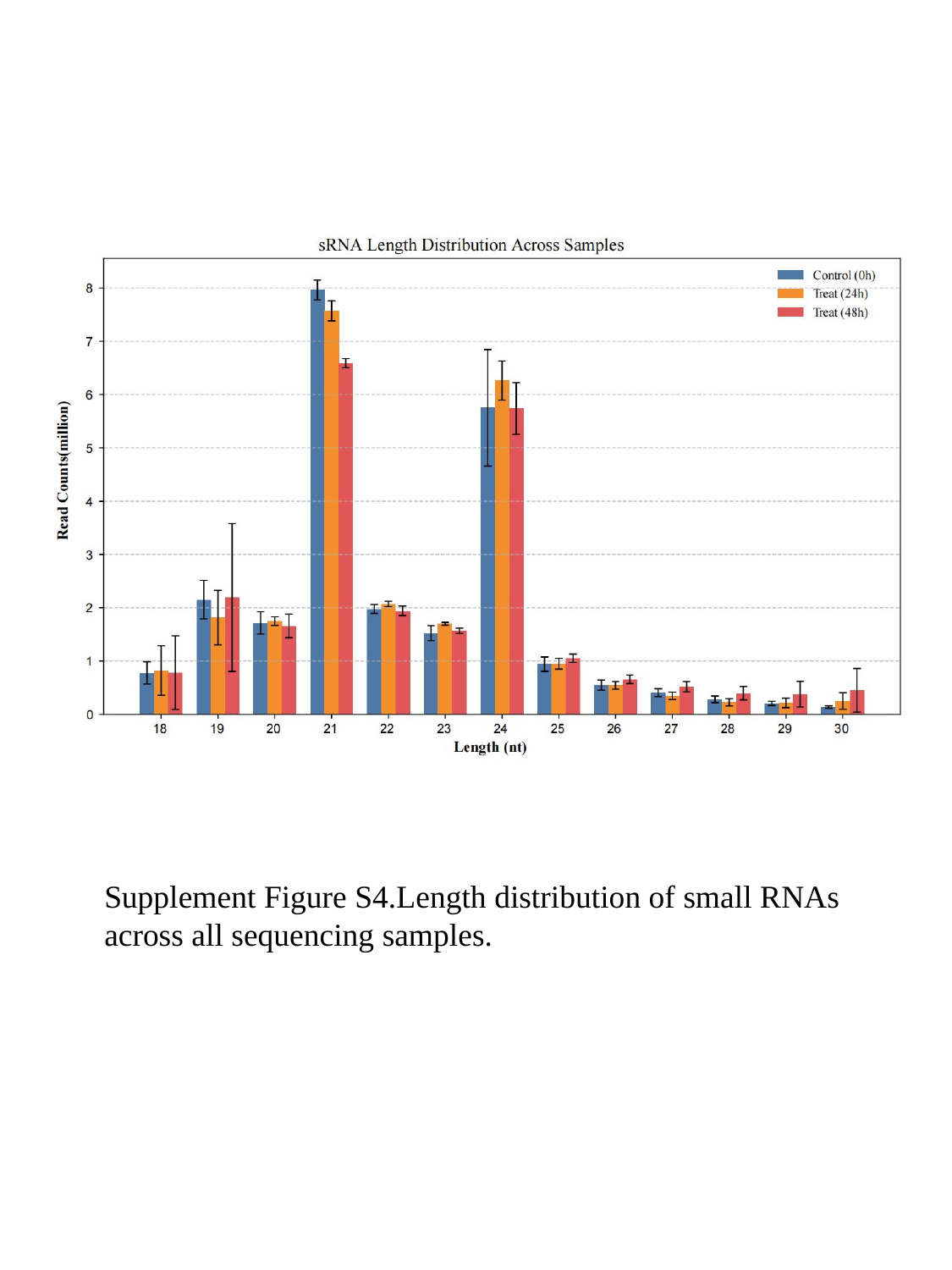

Supplement Figure S4.Length distribution of small RNAs across all sequencing samples.

Supplement: Supplementary file 4 — Figure S4: Length distribution of small RNAs across all sequencing samples. [file MPP-27-e70301-s014.pptx]
