# Supplementary material for: Alternative Polyadenylation Dynamics During the Rice Blast Immune Response
Source: Mol Plant Pathol. 2026 Jun 26;27(7):e70301. doi: 10.1111/mpp.70301 (PMC13305335; doi:10.1111/mpp.70301)
Supplement: Supplementary file 5 — Figure S5: Relationship of the expression between APA genes and targeting miRNA. Fc1 denotes the expression pattern of RNA‐seq and fc2 denotes the expression pattern of miRNA‐seq. [file MPP-27-e70301-s004.pptx]

## Slide 1
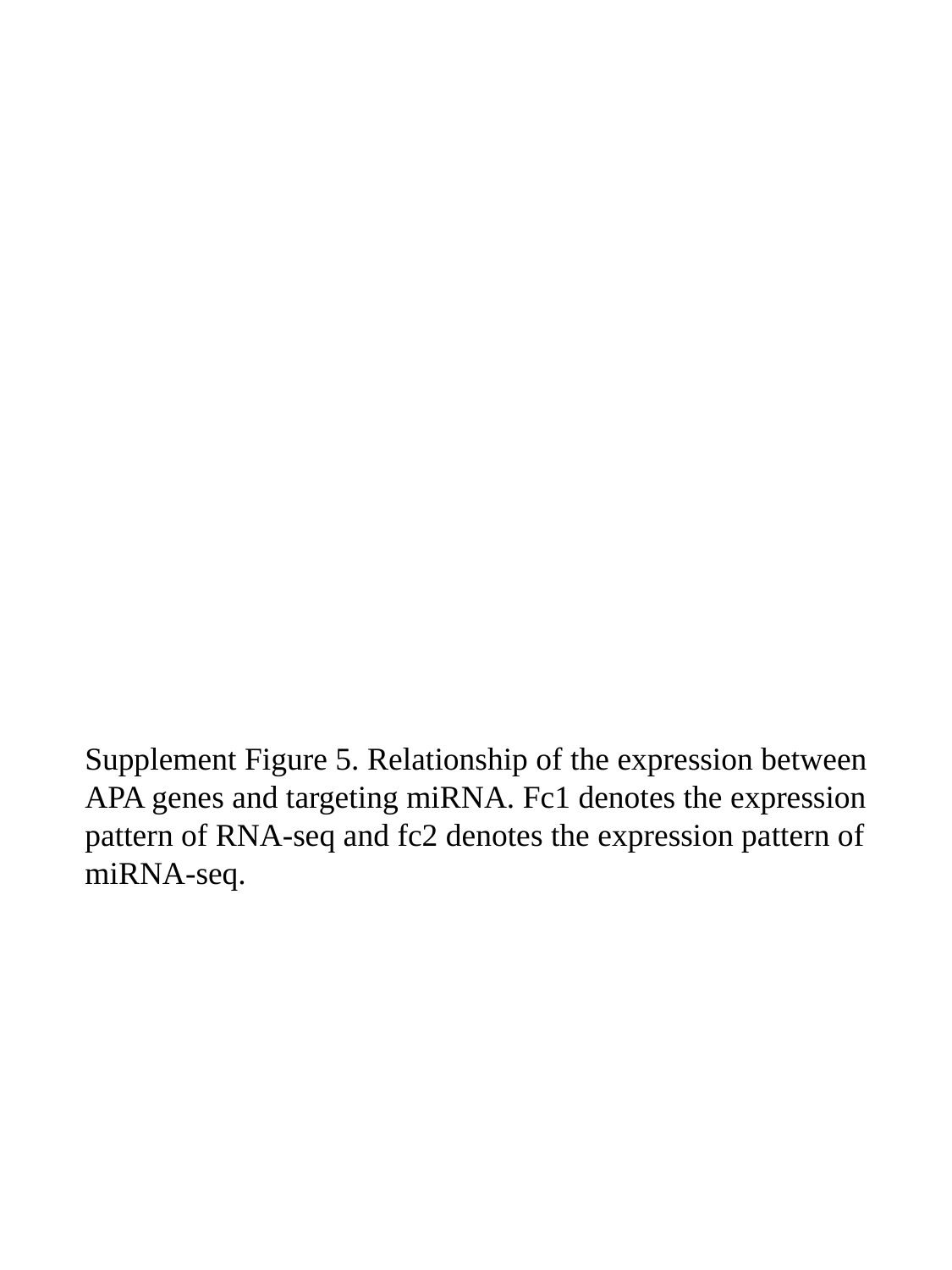

Supplement Figure 5. Relationship of the expression between APA genes and targeting miRNA. Fc1 denotes the expression pattern of RNA-seq and fc2 denotes the expression pattern of miRNA-seq.
