# Supplementary material for: Alternative Polyadenylation Dynamics During the Rice Blast Immune Response
Source: Mol Plant Pathol. 2026 Jun 26;27(7):e70301. doi: 10.1111/mpp.70301 (PMC13305335; doi:10.1111/mpp.70301)
Supplement: Supplementary file 6 — Figure S6: Quantitative comparison of absolute expression abundances between miRNA‐targeted APA genes and miRNA‐targeted non‐APA genes at 24 and 48 h post‐inoculation (hpi). [file MPP-27-e70301-s012.pptx]

## Slide 1
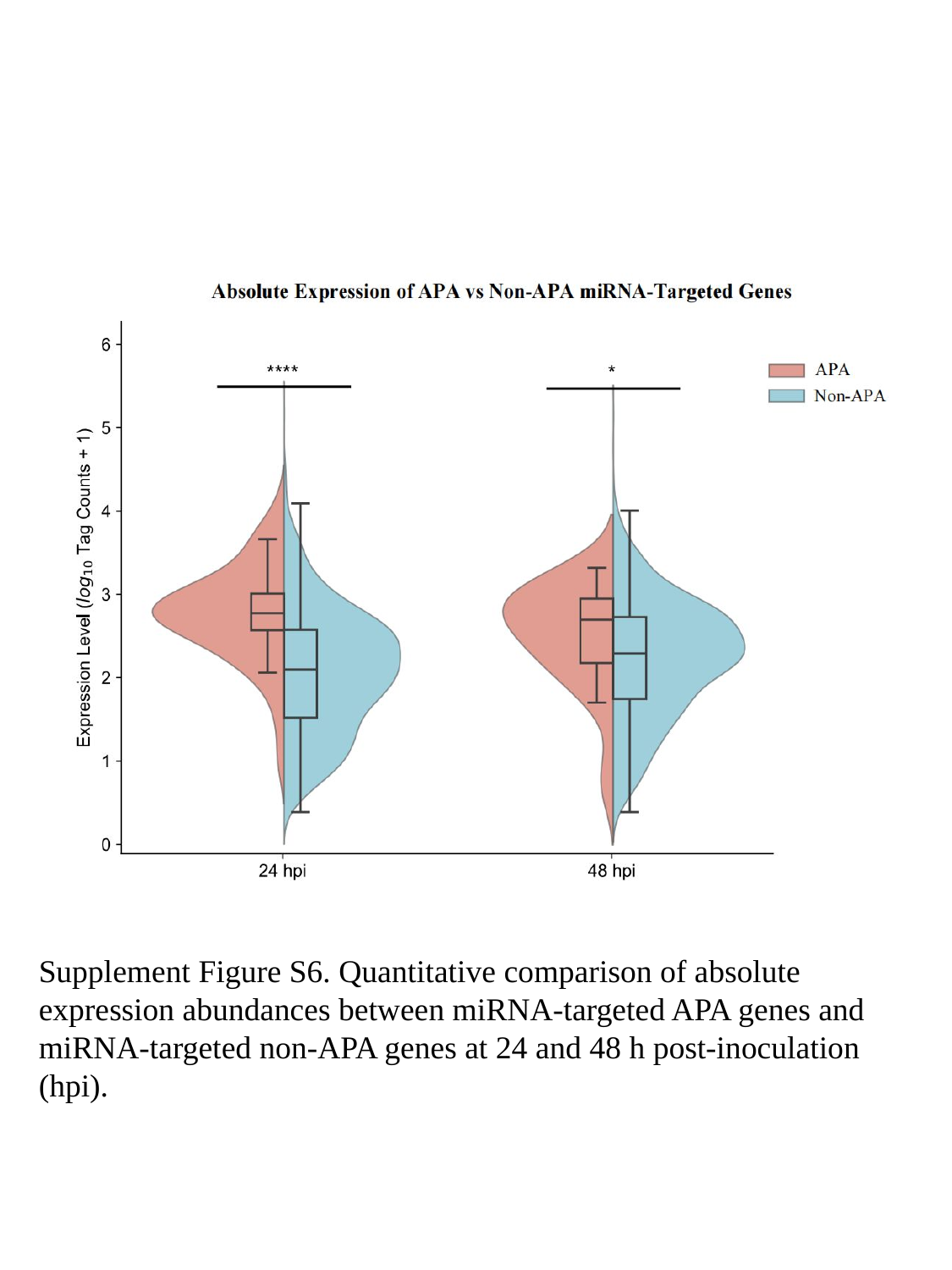

Supplement Figure S6. Quantitative comparison of absolute expression abundances between miRNA-targeted APA genes and miRNA-targeted non-APA genes at 24 and 48 h post-inoculation (hpi).
