# Supplementary material for: Alternative Polyadenylation Dynamics During the Rice Blast Immune Response
Source: Mol Plant Pathol. 2026 Jun 26;27(7):e70301. doi: 10.1111/mpp.70301 (PMC13305335; doi:10.1111/mpp.70301)
Supplement: Supplementary file 7 — Figure S7: Dynamics of alternative polyadenylation and miRNA evasion in key immune genes. Structural models and expression profiles for (A) OsRbohD (LOC_Os11g33120) targeted by osa‐miR156f‐5p and (B) OsNTL5 (LOC_Os08g44820) targeted by osa‐miR159b. [file MPP-27-e70301-s016.pptx]

## Slide 1
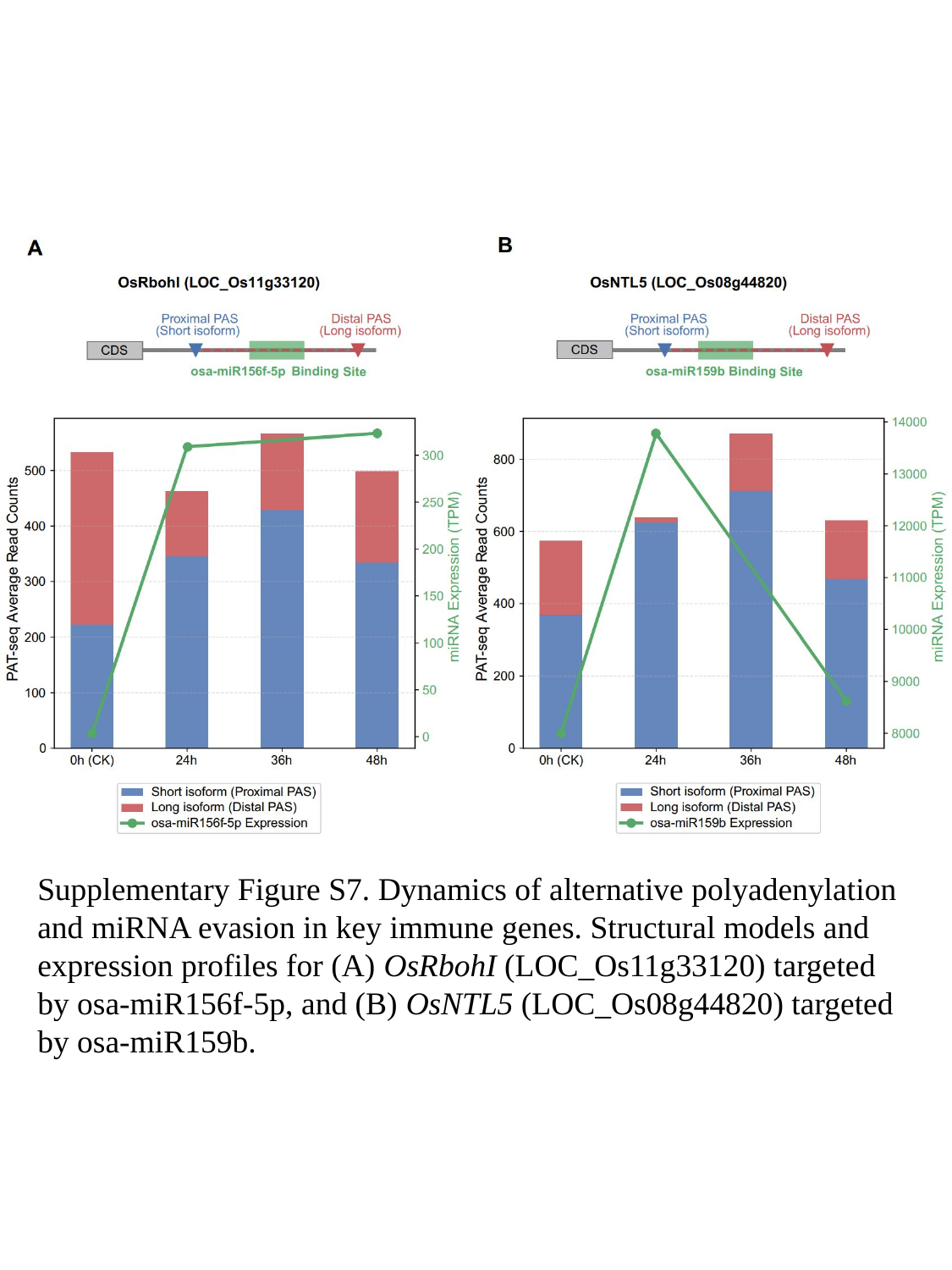

Supplementary Figure S7. Dynamics of alternative polyadenylation and miRNA evasion in key immune genes. Structural models and expression profiles for (A) OsRbohI (LOC_Os11g33120) targeted by osa-miR156f-5p, and (B) OsNTL5 (LOC_Os08g44820) targeted by osa-miR159b.
