# Supplementary material for: Alternative Polyadenylation Dynamics During the Rice Blast Immune Response
Source: Mol Plant Pathol. 2026 Jun 26;27(7):e70301. doi: 10.1111/mpp.70301 (PMC13305335; doi:10.1111/mpp.70301)
Supplement: Supplementary file 8 — Figure S8: Discriminative sequence motif analysis (STREME) of the 100‐bp flanking regions surrounding the preferred versus abandoned alternative poly(A) sites. [file MPP-27-e70301-s018.pptx]

## Slide 1
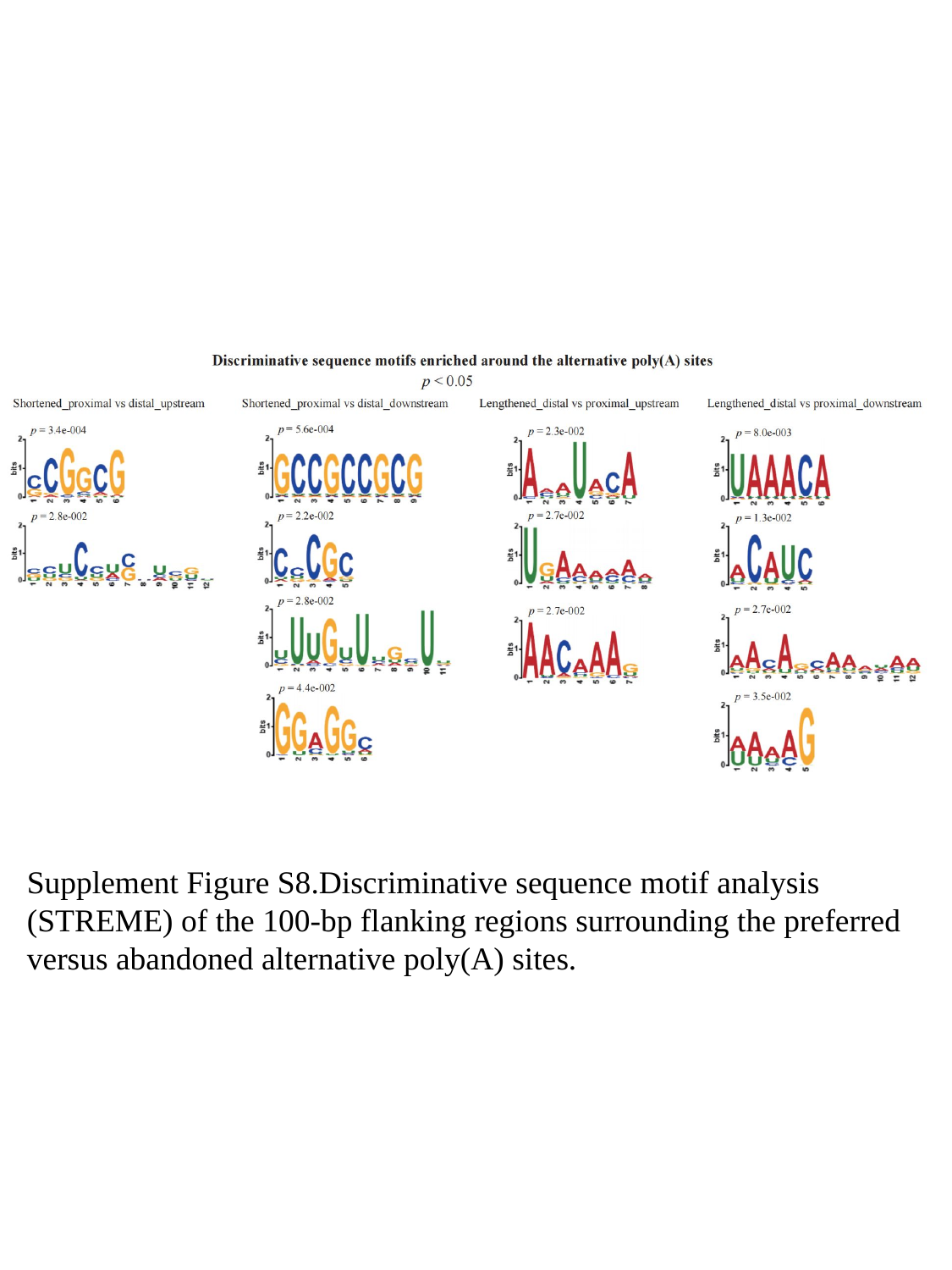

Supplement Figure S8.Discriminative sequence motif analysis (STREME) of the 100-bp flanking regions surrounding the preferred versus abandoned alternative poly(A) sites.
