# Supplementary material for: Alternative Polyadenylation Dynamics During the Rice Blast Immune Response
Source: Mol Plant Pathol. 2026 Jun 26;27(7):e70301. doi: 10.1111/mpp.70301 (PMC13305335; doi:10.1111/mpp.70301)
Supplement: Supplementary file 9 — Figure S9: Dynamics of alternative poly(A) site usage for the Os05g0509500 gene across the Magnaporthe oryzae infection time course. [file MPP-27-e70301-s002.pptx]

## Slide 1
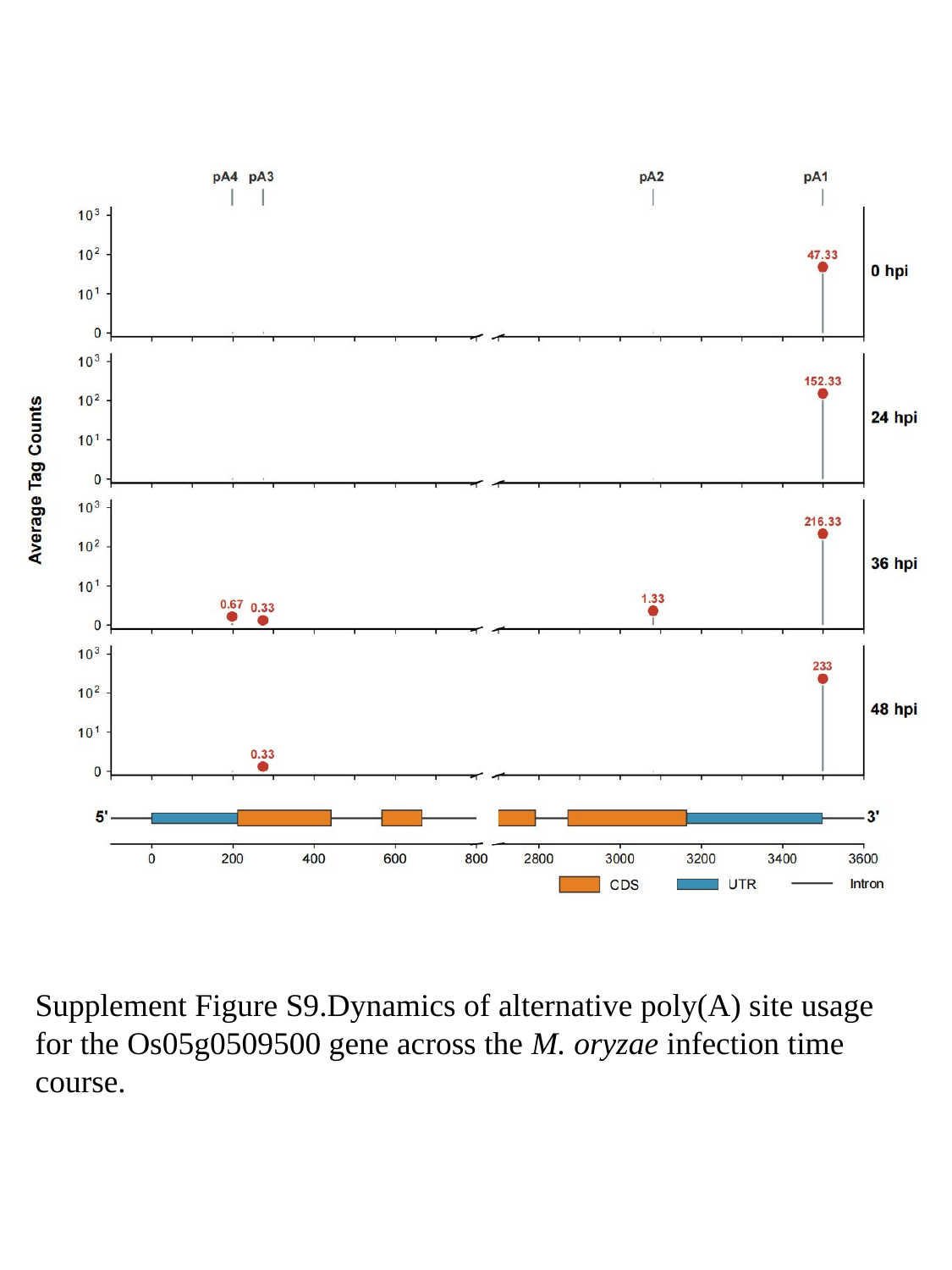

Supplement Figure S9.Dynamics of alternative poly(A) site usage for the Os05g0509500 gene across the M. oryzae infection time course.
