# Supplementary material for: Alternative Polyadenylation Dynamics During the Rice Blast Immune Response
Source: Mol Plant Pathol. 2026 Jun 26;27(7):e70301. doi: 10.1111/mpp.70301 (PMC13305335; doi:10.1111/mpp.70301)
Supplement: Supplementary file 12 — Table S3: Alignment statistics of tags aligned to the reference genome. [file MPP-27-e70301-s003.pdf]

Supplemental Table 3 Statistics of target and reference genome alignment in miRNA-seq data

Sample name	Total tag	Mapped tag	Percentage(%)
control_0h_1	25377782	23531128	92.72
control_0h_2	23011111	21576972	93.77
control_0h_3	25528164	23446034	91.84
treat_12h_1	24025877	22484467	93.58
treat_12h_2	24676264	22902584	92.81
treat_12h_3	23450702	21480258	91.6
treat_24h_1	25627317	23563860	91.95
treat_24h_2	23755338	21806722	91.8
treat_24h_3	24493895	21961459	89.66
treat_48h_1	23588358	20830414	88.31
treat_48h_2	24692396	22276577	90.22
treat_48h_3	25019143	21672263	86.62
