# Supplementary material for: Alternative Polyadenylation Dynamics During the Rice Blast Immune Response
Source: Mol Plant Pathol. 2026 Jun 26;27(7):e70301. doi: 10.1111/mpp.70301 (PMC13305335; doi:10.1111/mpp.70301)
Supplement: Supplementary file 13 — Table S4: Summary of sequencing data for each sample. [file MPP-27-e70301-s013.pdf]

Supplemental Table 4 Summary of miRNA-seq data for each sample

Sample name	Sequence type	Raw tag count	Low quallity tag count	Invalid adapter tag count	PolyA tag count	Short valid length tag	Clean tag count	Q20 of clean tag (%)	Percentage of clean tag(%)
control_0h_1	SE50	27423291	581356	1225792	758	237603	25377782	98.0	92.54
control_0h_2	SE50	25771502	671033	981576	470	1107312	23011111	98.1	89.29
control_0h_3	SE50	27310736	656141	916041	1073	209317	25528164	98.1	93.47
treat_12h_1	SE50	26235651	643517	1001004	281	564972	24025877	98.4	91.58
treat_12h_2	SE50	27167378	711227	1122213	260	657414	24676264	98.3	90.83
treat_12h_3	SE50	25402013	641646	1076416	351	232898	23450702	98.3	92.32
treat_24h_1	SE50	27499086	592673	792490	685	485921	25627317	97.7	93.19
treat_24h_2	SE50	25644832	549533	1079347	740	259874	23755338	98.2	92.63
treat_24h_3	SE50	26814844	670268	1222321	268	428092	24493895	97.9	91.34
treat_48h_1	SE50	25584888	778908	992371	358	224893	23588358	98.5	92.20
treat_48h_2	SE50	26871274	479151	1137994	387	561346	24692396	98.8	91.89
treat_48h_3	SE50	27289054	607459	958973	585	702894	25019143	98.8	91.68
