# Supplementary material for: Alternative Polyadenylation Dynamics During the Rice Blast Immune Response
Source: Mol Plant Pathol. 2026 Jun 26;27(7):e70301. doi: 10.1111/mpp.70301 (PMC13305335; doi:10.1111/mpp.70301)
Supplement: Supplementary file 17 — Table S8: Predicted siRNAs across all samples. [file MPP-27-e70301-s019.pdf]

siRNA id	Guide sequence	Passenger sequence	Tag info
novel_sir1	TTTTTTTCACCGATGTAAATCAAA	AAAAAGTGGCTACATTTAGTTTGC	tag193842
novel_sir2	TTTTTTTCACCGATGTAAATCAAA	AAAAAGTGGCTACATTTAGTTTGT	tag193842
novel_sir3	GGTTTTTGTAGTGTAGCTTAGAAA	AAAAACATCACATCGAATCTTTGG	tag139538
novel_sir4	CTTTTCTAAAAGCACCGTGCGAAA	AAAGATTTTCGTGGCACGCTTTTC	tag253303
novel_sir5	AAAGCGACGACGGCACTTTCG	TGTTTCGCTGCTGCCGTGAAA	tag84388
novel_sir6	CCTTTGAGTACCGACGTGAATAAA	AAACTCATGGCTGCACTTATTTTG	tag168060
novel_sir7	AAAGACAGCGGGCTATAGATTTGA	TCTTTCTGTCGCCCGATATCTAAA	tag6660
novel_sir8	TCTTTTCGTCGCCCGATGTCTAAA	AAAAGCAGCGGGCTACAGATTTAT	tag92284
novel_sir9	TATTTTTATCGCCTGATGTCTAAA	AAAAATAGCGGACTACAGATTTGT	tag128775
novel_sir10	TCTTTTTGTCGCCTGATGTCTAAA	AAAAACAGCGGACTACAGATTTAT	tag52861
novel_sir11	AAAAATAGCGGGCTACAAATTTAT	TCTTTTTATCGCCCGATGTTTAAA	tag124693
novel_sir12	TTTTTATAGATGCAAATCAAACAA	AAATATCTACGTTTAGTTTGTTGT	tag120781
novel_sir13	TATTTCACACCGGTTATTAAACAA	AAAGTGTGGCCAATAATTTGTTGG	tag137158
novel_sir14	TCTTTCCGGAACTCAAGTTTACAA	AAAGGCCTTGAGTTCAAATGTTGC	tag109059
novel_sir15	GCTTTTTTTTCGTAGTGCAAGCAA	AAAAAAAAGCATCACGTTCGTTAT	tag249545
novel_sir16	AAATCCTCTCCTAAGTCGTTGG	AATTTAGGAGAGGATTCAGCAA	tag277818
novel_sir17	GTTTTCATACCGGTTCTTTATCAA	AAAGTATGGCCAAGAAATAGTTAG	tag116204
novel_sir18	CGTTGTATAAGTCTAAGTCGTCAA	AACATATTCAGATTCAGCAGTTGA	tag89862
novel_sir19	ACTTGGACGGTAGCGGTTCAA	AACCTGCCATCGCCAAGTTGG	tag237817
novel_sir20	AAAGTCGACTCCAAATAAAGTTTC	AGTTTCAGCTGAGGTTTATTTCAA	tag68851
novel_sir21	TGTTTAGACATCGAGCGAAAAGAA	AAATCTGTAGCTCGCTTTTCTTCT	tag147062
novel_sir22	TGTTTAGACATCGGGCGAAAAGAA	AAATCTGTAGCCCGCTTTTCTTCT	tag113895
novel_sir23	AACATGTTTGACCGTTCGTCTTAT	ATTTGTACAAACTGGCAAGCAGAA	tag42025
novel_sir24	TGTTTAGATATCGGGTGAAGAGAA	AAATCTATAGCCCACTTCTCTTCT	tag213
novel_sir25	AAATCTGTAGTCCGCTGCTCTTCT	TGTTTAGACATCAGGCGACGAGAA	tag2546
novel_sir26	AAATCTGTAGTCCGCTGCTCTTCC	TGTTTAGACATCAGGCGACGAGAA	tag2546
novel_sir27	TGTTTAGACATCCGGCGACGAGAA	AAATCTGTAGGCCGCTGCTCTTCT	tag63575
novel_sir28	AAATCTATAGGCCGCTGCTCTTCT	TATTTAGATATCCGGCGACGAGAA	tag261986
novel_sir29	TGTTTAGACATCGGGCGACGAGAA	AAATCTGTAGCCCGCTGCTCTTCT	tag66076
novel_sir30	TGTTTAGATATCGGGCGACGAGAA	AAATCTATAGCCCGCTGCTCTTCT	tag105438
novel_sir31	TATTTAGATATCGGGCGACGAGAA	AAATCTATAGCCCGCTGCTCTTCT	tag174299
novel_sir32	TGTTTAGACATTGGGCGACGAGAA	AAATCTGTAACCCGCTGCTCTTCT	tag110948
novel_sir33	AAATCTATAGCTCGTTCCTCTTCT	TGTTTAGATATCGAGCAAGGAGAA	tag162906

novel_sir34	TGTTTAGACATCGGGCGATGAGAA	AAATCTGTAGCCCGCTACTCTTCT	tag147075
novel_sir35	AAATTTATAGCCCGCTACTCTTCT	TATTTAAATATCGGGCGATGAGAA	tag10256
novel_sir36	TCTTGAGGCTTCAATTCGCACGAA	AACTCCGAAGTTAAGCGTGCTTGG	tag14911
novel_sir37	CGTTTGACACCGTTTCACACCGAA	AAACTGTGGCAAAGTGTGGCTTGC	tag3198
novel_sir38	AATTTTATACAAATATCGACCGAA	AAAATATGTTTATAGCTGGCTTCC	tag71814
novel_sir39	AATTTTATACAAATATCGACCGAA	AAAATATGTTTATAGCTGGCTTGT	tag71814
novel_sir40	AACATCGAGCAGTCGGCTTGG	CGTTGTAGCTCGTCAGCCGAA	tag168655
novel_sir41	ACTTTCCTCGGTGGTGCAAGCGAA	AAAGGAGCCACCACGTTCGCTTGG	tag154532
novel_sir42	TATTTAGATATCGGGCAACAGGAA	AAATCTATAGCCCGTTGTCCTTCT	tag235380
novel_sir43	TGTTTAGACATCGGGCGACGGGAA	AAATCTGTAGCCCGCTGCCCTTCT	tag30560
novel_sir44	AAATCTATAGCCCGCTGCCCTTCT	TGTTTAGATATCGGGCGACGGGAA	tag134974
novel_sir45	AAATCTGTAGCCCGCTACCCTTCT	TGTTTAGACATCGGGCGATGGGAA	tag43266
novel_sir46	GTTTTATAAACTGCGGCAACTGAA	AAATATTTGACGCCGTTGACTTTT	tag204310
novel_sir47	AACTCTTAGTTGTCAAGCACTTTG	AGTTGAGAATCAACAGTTCGTGAA	tag116207
novel_sir48	TGTTTACTTGGCGACGGTTTTGAA	AAATGAACCGCTGCCAAAACTTAT	tag215279
novel_sir49	AAATAAACCGCTGCCAAAACTTAT	TGTTTATTTGGCGACGGTTTTGAA	tag28055
novel_sir50	AAATTGCGCGGCTAGTATTATTAT	CGTTTAACGCGCCGATCATAATAA	tag174808
novel_sir51	GGTTGCTAAACGAGGCTAATAA	AACGATTTGCTCCGATTATTGG	tag154112
novel_sir52	AACGGTCTAGATTTATTGTATTAG	ACTTGCCAGATCTAAATAACATAA	tag24901
novel_sir53	TTTTTCAAAAATTGGCCCTGATAA	AAAGTTTTTAACCGGGACTATTGT	tag15049
novel_sir54	CCTTTACGATCTTACTGAATATAA	AAATGCTAGAATGACTTATATTGT	tag76330
novel_sir55	TCTTTACGATCTTACTGAATATAA	AAATGCTAGAATGACTTATATTGT	tag247258
novel_sir56	AATTCGTTTTCAGCTACCGTATAA	AAGCAAAAGTCGATGGCATATTGT	tag32017
novel_sir57	AACGTAATTAATGGCCTTGATTCT	AGTTGCATTAATTACCGGAACTAA	tag237880
novel_sir58	GATTGATACCTATCTGACGACTAA	AACTATGGATAGACTGCTGATTAT	tag55338
novel_sir59	GCTTGTGTTACCACATTAGCCTAA	AACACAATGGTGTAATCGGATTGT	tag50266
novel_sir60	GCTTCGATGGCACACGGCCTAA	AAGCTACCGTGTGCCGGATTAT	tag148000
novel_sir61	GGTTGCTGAATCCTCTCCTAA	AACGACTTAGGAGAGGATTTA	tag168410
novel_sir62	AAACTTCGGCCCGATTGAGATTAC	GCTTTGAAGCCGGGCTAACTCTAA	tag46315
novel_sir63	AAATTACGAGGCCATTCAGATTGT	TCTTTAATGCTCCGGTAAGTCTAA	tag270073
novel_sir64	GGTTGAAAACCGAAGATTAAGTAA	AACTTTTGGCTTCTAATTCATTTT	tag28809
novel_sir65	AAAGGTCAGCGCCACTCTCATTGG	AGTTTCCAGTCGCGGTGAGAGTAA	tag201141
novel_sir66	AAATTGCGCGGCTAGTATCATTAT	CGTTTAACGCGCCGATCATAGTAA	tag231387
novel_sir67	CGTTAGACGCGCCGATCATAGTAA	AATCTGCGCGGCTAGTATCATTAT	tag197224

novel_sir68	AATTTATGCGCTGCCCCGTAA	AAATACGCGACGGGGCATTGT	tag127854
novel_sir69	TGTTTAGATATCGGGTGACGGTAA	AAATCTATAGCCCACTGCCATTCT	tag134112
novel_sir70	AATATGCGTGGGACCCATTAG	TTTTATACGCACCCTGGGTAA	tag287634
novel_sir71	AAAGTCAACGACGTTATACATTAA	CTTTTCAGTTGCTGCAATATGTAA	tag159069
novel_sir72	CCTTAGCCCCTCTTTTAACTGTAA	AATCGGGGAGAAAATTGACATTGT	tag107158
novel_sir73	TTTTTCAGTTGCCGCAGTTTGTAA	AAAGTCAACGGCGTCAAACATTTT	tag192080
novel_sir74	GATTCCGGGTAAGCTTCTAATTAA	AAGGCCCATTCGAAGATTAATTCT	tag16347
novel_sir75	AAGATGGGTGGCCCGCGTAATTGC	TGTTCTACCCACCGGGCGCATTAA	tag136244
novel_sir76	AAGTGACTCTCTAAGTTGAATTTT	GGTTCACTGAGAGATTCAACTTAA	tag17250
novel_sir77	GCTTGATAGCGCCAGCTGACTTAA	AACTATCGCGGTCGACTGAATTAC	tag177720
novel_sir78	AACTATCGCGGTCGTCCGAATTAC	GCTTGATAGCGCCAGCAGGCTTAA	tag166385
novel_sir79	GCTTGATAGCGCCAGCTGGCTTAA	AACTATCGCGGTCGACCGAATTAT	tag290549
novel_sir80	AACTATCGCGGTCGACCGAATTAC	GCTTGATAGCGCCAGCTGGCTTAA	tag290549
novel_sir81	AGTTGATACGTTTGGCCTGCTTAA	AACTATGCAAACCGGACGAATTAC	tag39917
novel_sir82	GGTTGATACGTTTGGCCTGCTTAA	AACTATGCAAACCGGACGAATTAC	tag200627
novel_sir83	AATACAACAGCCAGAGACAATTGT	TGTTATGTTGTCGGTCTCTGTTAA	tag232072
novel_sir84	GGTTCACTGAGAGATTCGATTTAA	AAGTGACTCTCTAAGCTAAATTTT	tag175681
novel_sir85	TTTTAACTTTCAAATCAGCTTTAA	AATTGAAAGTTTAGTCGAAATTGT	tag77619
novel_sir86	AAAAGACCGAGACCGAGAAATTCG	GCTTTTCTGGCTCTGGCTCTTTAA	tag86040
novel_sir87	AACAACCTCTCTAAGCCAAATTTT	GGTTGTTGGAGAGATTCGGTTTAA	tag122127
novel_sir88	AAGACTGTAAGCTGGACAAATTGT	CCTTCTGACATTCGACCTGTTTAA	tag112518
novel_sir89	GGTTCAGAAACAGGATATTTTTAA	AAGTCTTTGTCCTATAAAAATTAT	tag36108
novel_sir90	TATTACAGAACCTCAGGCACAACA	AATGTCTTGGAGTCCGTGTTGTAG	tag102572
novel_sir91	TGTTTAGACATCGGGCGGAGAACA	AAATCTGTAGCCCGCCTCTTGTCT	tag36187
novel_sir92	AACGGTTGTTTTGTCTGATTGTGT	CTTTGCCAACAAAACAGACTAACA	tag39558
novel_sir93	AATGAGATGGTTCCAGTTGTGTGT	CCTTACTCTACCAAGGTCAACACA	tag203038
novel_sir94	GCTTGAGGCTCTACATTACACACA	AACTCCGAGATGTAATGTGTGTAT	tag59138
novel_sir95	ACTTACTATCAATTCGCCACA	AATGATAGTTAAGCGGTGTCT	tag238677
novel_sir96	AATTATAGTACAATCGTAGTGTAA	TATTAATATCATGTTAGCATCACA	tag30589
novel_sir97	GATTTCACACCGCACCGTTTCACA	AAAGTGTGGCGTGGCAAAGTGTGG	tag156765
novel_sir98	GGTTTGCGATTCGATTGTTAGACA	AAACGCTAAGCTAACAATCTGTGG	tag219105
novel_sir99	AAACTGAAAGTAGAGATGCTGTCC	GGTTTGACTTTCATCTCTACGACA	tag207445
novel_sir100	ACAAATTTGGATAGGAGGCTGTTC	TATGTTTAAACCTATCCTCCGACA	tag111580
novel_sir101	GCTTGACGCGGTACCTCGACA	AACTGCGCCATGGAGCTGTCC	tag49409

novel_sir102	GGTTAGAACAGCGAATCCCGGACA	AATCTTGTCGCTTAGGGCCTGTTT	tag81077
novel_sir103	AATTGCCGAATCTTTAGAATGACA	AACGGCTTAGAAATCTTACTGTCT	tag155409
novel_sir104	GATTGGAATCTGCACCGTTTGACA	AACCTTAGACGTGGCAAACTGTGG	tag88993
novel_sir105	ACAGGCTAGAAGTCACTGATGTGA	AGTGTCCGATCTTCAGTGACTACA	tag86276
novel_sir106	AGTTCTAAGCAACATGATACTACA	AAGATTCGTTGTACTATGATGTGT	tag14552
novel_sir107	GATTTTAATCACCGCGACTCTACA	AAAATTAGTGGCGCTGAGATGTTG	tag107809
novel_sir108	ACAGGCTAGAAGTCACAGATGTGA	AGTGTCCGATCTTCAGTGTCTACA	tag74941
novel_sir109	TGTTTGCGAGAGTTTGGCGGTACA	AAACGCTCTCAAACCGCCATGTGG	tag184921
novel_sir110	AAGACGAATGGTCAAACATGTGA	TATTCTGCTTACCAGTTTGTACA	tag59826
novel_sir111	TATTCTGCTTGCCAGTTTGTACA	AAGACGAACGGTCAAACATGTGC	tag208577
novel_sir112	TATTCTGCTTGCCAGTTTGTACA	AAGACGAACGGTCAAACATGTTT	tag208577
novel_sir113	TCTTGCCTGAGGTTCTGTATTACA	AACGGACTCCAAGACATAATGTGT	tag109053
novel_sir114	AACATCCGCCGATCCCTGGTCG	CGTTGTAGGCGGCTAGGGACCA	tag122656
novel_sir115	AAAATTATAATGGCATGGTTC	CGTTTTAATATTACCGTACCA	tag83795
novel_sir116	ACTTAGTAGCATCCGGAACCCA	AATCATCGTAGGCCTTGGGTTA	tag132291
novel_sir117	ACAGGTATCTAGTCAGACGGGTTC	AGTGTCCATAGATCAGTCTGCCCA	tag58877
novel_sir118	TTTTGATTCGCCTATGTTAAGCCA	AACTAAGCGGATACAATTCGGTGC	tag56059
novel_sir119	TTTTAATCGAACTAGTGTCAGCCA	AATTAGCTTGATCACAGTCGGTCA	tag213703
novel_sir120	ATTTCAAATTCTATACGGTAGCCA	AAGTTTAAGATATGCCATCGGTAT	tag12425
novel_sir121	ACTTTTAACCAAAAGCGATCGCCA	AAAATTGGTTTTCGCTAGCGGTCC	tag3604
novel_sir122	GATTTCTACTAGAAATCAGGGCCA	AAAGATGATCTTTAGTCCCGGTTG	tag129005
novel_sir123	GATTTCTACTAGAAATCAGGGCCA	AAAGATGATCTTTAGTCCCGGTTC	tag129005
novel_sir124	AAAGATCATCTTTAGTCCCGGTTG	GATTTCTAGTAGAAATCAGGGCCA	tag119148
novel_sir125	AAGATGCATCGTTAGTCCCGGTTG	ATTTCTACGTAGCAATCAGGGCCA	tag161444
novel_sir126	TGTTATATAATAACGATCTGGCCA	AATATATTATTGCTAGACCGGTTG	tag31676
novel_sir127	TTTTAAAGTATATTCTGCCTGCCA	AATTTCATATAAGACGGACGGTCA	tag94187
novel_sir128	AATATTTTGAAAAGCAGACGGTTG	AATTATAAAACTTTTCGTCTGCCA	tag135142
novel_sir129	CCTGTTACTTAGACCTGTTTGCCA	ACAATGAATCTGGACAAACGGTCT	tag149888
novel_sir130	GCTGTCGTGTCGCCTCCTCCA	ACAGCACAGCGGAGGAGGTGG	tag110752
novel_sir131	AATTATGTCACGCCAGCGAGGTTG	TATTAATACAGTGCGGTCGCTCCA	tag234717
novel_sir132	ACAACGTAAGTGTGAGTAAGGTGA	GCTGTTGCATTCACACTCATTCCA	tag109338
novel_sir133	CGTGGGCGTTACCATTTCATTCCA	ACCCGCAATGGTAAAGTAAGGTGC	tag125373
novel_sir134	GTTGGCTGGCCAACCGATATTCCA	ACCGACCGGTTGGCTATAAGGTTC	tag81319
novel_sir135	TATTCACGTCGATATCTATAAGCA	AAGTGCAGCTATAGATATTCGTCT	tag234726

novel_sir136	CGTTAAATCCGAGTTGTCTAAGCA	AATTTAGGCTCAACAGATTCGTCT	tag65222
novel_sir137	AACATTCATGCAAATTCGTGG	GTTTGTAAGTACGTTTAAGCA	tag119571
novel_sir138	TGTTACAATGATAGTGAAACAGCA	AATGTTACTATCACTTTGTCGTAT	tag141696
novel_sir139	GGTGGCGTAGCCGCGACAGCA	ACCGCATCGGCGCTGTCGTCG	tag173242
novel_sir140	AAATCCTCTCCTAAGTCGTTG	AATTTAGGAGAGGATTCAGCA	tag123440
novel_sir141	CCTGTATTATTGTATGACCGAGCA	ACATAATAACATACTGGCTCGTCT	tag230926
novel_sir142	AATGTTGTGTTTTGGAGATCGTGT	AGTTACAACACAAAACCTCTAGCA	tag57705
novel_sir143	AACGTAGGAATTGAAGTGGCGTGT	TTTTGCATCCTTAACTTCACCGCA	tag254497
novel_sir144	AGTGGCTGCCGCTGGTCCGCA	ACCGACGGCGACCAGGCGTTC	tag277435
novel_sir145	ACGAGAGGTGACGGCGCGTGG	CCTGCTCTCCACTGCCGCGCA	tag195815
novel_sir146	AAGCTTTGCCGTGACGCGTCT	TTTTCGAAACGGCACTGCGCA	tag100792
novel_sir147	TCTTATATTACTATGATCTGCGCA	AATATAATGATACTAGACGCGTAA	tag19244
novel_sir148	ACCTCACATCTTACGAACGCGTCG	TGTGGAGTGTAGAATGCTTGCGCA	tag49084
novel_sir149	AACAAACAAGCAGCTTTTCCGTTT	ATTTGTTTGTTCGTCGAAAAGGCA	tag208894
novel_sir150	GTTGCTTAAACCTATCCTCAGGCA	ACGAATTTGGATAGGAGTCCGTCC	tag232382
novel_sir151	AATCGCTAATGGACCGCTCCGTTT	AGTTAGCGATTACCTGGCGAGGCA	tag290020
novel_sir152	CATTGAAATGCAGTCTCCTAGGCA	AACTTTACGTCAGAGGATCCGTTT	tag162204
novel_sir153	AACAAATTTTGGACGGAGCCGTCA	TGTTGTTTAAAACCTGCCTCGGCA	tag74047
novel_sir154	GGTGGACGGTTCAACAACATGGCA	ACCTGCCAAGTTGTTGTACCGTGT	tag285716
novel_sir155	GGTGGACGGTTCAACAACATGGCA	ACCTGCCAAGTTGTTGTACCGTGG	tag285716
novel_sir156	ACCTGCCAAGCTGTTGTACCGTGG	GGTGGACGGTTCGACAACATGGCA	tag156791
novel_sir157	ACCATGTCCAGTGTTTGACCGTTT	CTTGGTACAGGTCACAAACTGGCA	tag208538
novel_sir158	TTTGGCATAGGCTGCAAACTGGCA	ACCGTATCCGACGTTTGACCGTTC	tag263493
novel_sir159	AAAGTTGTTTTTTGGAGACCGTGC	AGTTTCAACAAAAAACCTCTGGCA	tag79545
novel_sir160	AGTTTCAACAAAAAACCTCTGGCA	AAAGTTGTTTTTTGGAGACCGTAC	tag79545
novel_sir161	AGTTTCAACACAAAACCTCTGGCA	AAAGTTGTGTTTTGGAGACCGTGT	tag102412
novel_sir162	ACAATAAATCTAAACAGACCGTTT	TGTGTTATTTAGATTTGTCTGGCA	tag26396
novel_sir163	AGTTATATCGTAGAGCTTCTGGCA	AATATAGCATCTCGAAGACCGTGC	tag38310
novel_sir164	ACCTCCTGGAAGCTGCATACGTTG	AGTGGAGGACCTTCGACGTATGCA	tag51476
novel_sir165	AAAATTGAACTGAATGAGACGTTT	GATTTTAACTTGACTTACTCTGCA	tag54956
novel_sir166	CCTTTTTAGCGCCAGGAGAAATCA	AAAAATCGCGGTCCTCTTTAGTGA	tag135808
novel_sir167	AAACTGATCGGATGTTGTTAGTTT	CTTTTGACTAGCCTACAACAATCA	tag127979
novel_sir168	GCTGCGCTGGATGTTGAAACATCA	ACGCGACCTACAACTTTGTAGTTG	tag196947
novel_sir169	CCTTTACAGAATAGACTAAGATCA	AAATGTCTTATCTGATTCTAGTTC	tag12970

novel_sir170	GATGGCATGAAGGTGTACAGATCA	ACCGTACTTCCACATGTCTAGTGG	tag211056
novel_sir171	AATTCTAAGAATGTTCGTCGATCA	AAGATTCTTACAAGCAGCTAGTTG	tag104303
novel_sir172	TCTTAGGCTAAATTTCTTCGATCA	AATCCGATTTAAAGAAGCTAGTGA	tag293011
novel_sir173	TGTGCCGTACCAATAGGCTGATCA	ACGGCATGGTTATCCGACTAGTAA	tag108301
novel_sir174	ACGGCATGGTTATCCGACTAGTTA	TGTGCCGTACCAATAGGCTGATCA	tag108301
novel_sir175	TGTGCCGTACCAATAGGCTGATCA	ACGGCATGGTTATCCGACTAGTCA	tag108301
novel_sir176	GCTGGGGCTGGCCTTTCGGTATCA	ACCCCGACCGGAAAGCCATAGTCC	tag272996
novel_sir177	TATTATCGGTTAAGTATGTTATCA	AATAGCCAATTCATACAATAGTTG	tag182859
novel_sir178	CGTTTCAATCCGTTTCCTAACTCA	AAAGTTAGGCAAAGGATTGAGTCT	tag282274
novel_sir179	AATCCACTTAGTGCCACTGAGTTT	TCTTAGGTGAATCACGGTGACTCA	tag81396
novel_sir180	ACAAAGATCTGAATGTAGGAGTTC	AGTGTTTCTAGACTTACATCCTCA	tag99986
novel_sir181	TTTGGTTTGTGTTCGGATTCCTCA	ACCAAACACAAGCCTAAGGAGTTG	tag232398
novel_sir182	GATGCCGAACGCGTATGTAGCTCA	ACGGCTTGCGCATACATCGAGTGG	tag200104
novel_sir183	ATTGCGACGGTGCGATCGGTCTCA	ACGCTGCCACGCTAGCCAGAGTGG	tag69375
novel_sir184	AATTGTTTGGCTGAGATTCAGTTC	CTTTAACAAACCGACTCTAAGTCA	tag81131
novel_sir185	GGTTCTTCAGATGTGTTACAGTCA	AAGAAGTCTACACAATGTCAGTGT	tag190539
novel_sir186	ACGAGTCGTAGCTGAGTCAGTCG	TGTGCTCAGCATCGACTCAGTCA	tag4300
novel_sir187	AAAATCGGACGCTGACATCAGTAT	CTTTTTAGCCTGCGACTGTAGTCA	tag15672
novel_sir188	AGTTTACGATTCGCATACAGGTCA	AAATGCTAAGCGTATGTCCAGTTT	tag230240
novel_sir189	AAGCCACTTATACACCAGTCG	TTTTCGGTGAATATGTGGTCA	tag211660
novel_sir190	AAAGCCACTTATACACCAGTCG	CTTTTCGGTGAATATGTGGTCA	tag262479
novel_sir191	ACGCTGCCTTAGGTATTACAGTTC	TTTGCGACGGAATCCATAATGTCA	tag57758
novel_sir192	GTTTCTTATGAGTCCGACCTGTCA	AAGAATACTCAGGCTGGACAGTTT	tag247226
novel_sir193	ACCTAAGATTGGATGGGACAGTCC	CTTGGATTCTAACCTACCCTGTCA	tag254823
novel_sir194	CTTGGATTCTAACCTACCCTGTCA	ACCTAAGATTGGATGGGACAGTTC	tag254823
novel_sir195	TTTTAAAGTATATTCTGCCTGTCA	AATTTCATATAAGACGGACAGTCA	tag214832
novel_sir196	ACAGCTCTGGCTCTAGGTAAGTTG	TGTGTCGAGACCGAGATCCATTCA	tag119773
novel_sir197	CGTGTCGAGGCCGAGATCTATTCA	ACAGCTCCGGCTCTAGATAAGTTG	tag268923
novel_sir198	TCTGGATCGTATCTGACTAGTTCA	ACCTAGCATAGACTGATCAAGTTT	tag45802
novel_sir199	TCTTACTTTTTACTACACCGTTCA	AATGAAAAATGATGTGGCAAGTGA	tag121572
novel_sir200	AATGTTGGGTACCAAAGAATTTCA	ACAACCCATGGTTTCTTAAAGTGG	tag34460
novel_sir201	GATGTTGGGTACCAAAGAATTTCA	ACAACCCATGGTTTCTTAAAGTGG	tag45029
novel_sir202	AAATAAGACGTACGATTAAAGTTG	AATTTATTCTGCATGCTAATTTCA	tag291296
novel_sir203	AATTTATTCTGCCTGCTAATTTCA	AAATAAGACGGACGATTAAAGTTG	tag114393

novel_sir204	AGTTTATTCTGCCTGCTAATTTCA	AAATAAGACGGACGATTAAAGTTG	tag157809
novel_sir205	ACCGACAGCACAAAATTAAAGTAT	TATGGCTGTCGTGTTTTAATTTCA	tag16659
novel_sir206	GCTGCACTGCCTTTTCAACTTTCA	ACGTGACGGAAAAGTTGAAAGTTT	tag281836
novel_sir207	AAGGTTTGATGTGATGGAAAGTTG	CTTTCCAAACTACACTACCTTTCA	tag152302
novel_sir208	CCTTCAAACACACACATCCTTTCA	AAGTTTGTGTGTGTAGGAAAGTTT	tag91199
novel_sir209	ACTTATACCTGTTACGATCTTTCA	AATATGGACAATGCTAGAAAGTTT	tag255527
novel_sir210	ACTTATACCTGTTACGATCTTTCA	AATATGGACAATGCTAGAAAGTCT	tag255527
novel_sir211	TCTGCACCGTTTGACACCGTTTCA	ACGTGGCAAACTGTGGCAAAGTGT	tag258831
novel_sir212	TATGAGAGTAAACGATCGGTTTCA	ACTCTCATTTGCTAGCCAAAGTTT	tag37820
novel_sir213	ACGCAGATCCTAGGCACAAAGTCC	AGTGCGTCTAGGATCCGTGTTTCA	tag158210
novel_sir214	AATCCACGCTAAAATTAAAAGTTT	AATTAGGTGCGATTTTAATTTTCA	tag33052
novel_sir215	ACTTGTTAACCGTTTGCATTTTCA	AACAATTGGCAAACGTAAAAGTGG	tag142493
novel_sir216	ACCGTTTAGCAGTTTGAAAAGTGT	CGTGGCAAATCGTCAAACTTTTCA	tag88127
novel_sir217	AATTTCTACTGCACTGACTTTTCA	AAAGATGACGTGACTGAAAAGTTG	tag211379
novel_sir218	ACGCGTCATACTCTCGTTTTCTAG	GGTGCGCAGTATGAGAGCAAAAGA	tag76323
novel_sir219	ACAAATCTGTAGCCCGCTTTCTTC	GATGTTTAGACATCGGGCGAAAGA	tag128398
novel_sir220	ACACTTATGGCCAACTATTTCTAA	GGTGTGAATACCGGTTGATAAAGA	tag88303
novel_sir221	TATGACACACGCGATACAAGA	ACTGTGTGCGCTATGTTCTTG	tag233375
novel_sir222	AACTTTACAGTTGAGTCGTTCTGA	TATTGAAATGTCAACTCAGCAAGA	tag153395
novel_sir223	ACGCTTAACTTCGGAGTTCTGA	CGTGCGAATTGAAGCCTCAAGA	tag73469
novel_sir224	ACTATGTTCGTCAAACGCTTCTAT	TGTGATACAAGCAGTTTGCGAAGA	tag130727
novel_sir225	TGTTAAGTATCCAGGAATCGAAGA	AATTCATAGGTCCTTAGCTTCTGA	tag27793
novel_sir226	ACCTCTGACGGCTTCCACTTCTGG	TGTGGAGACTGCCGAAGGTGAAGA	tag42775
novel_sir227	TCTGGAGACAACGACACTATAAGA	ACCTCTGTTGCTGTGATATTCTGT	tag192680
novel_sir228	TGTTCTAGCACCGGTTGCCTAAGA	AAGATCGTGGCCAACGGATTCTAG	tag154092
novel_sir229	TGTTTTGGCCAGTGGACTCTAAGA	AAAACCGGTCACCTGAGATTCTTT	tag12643
novel_sir230	ACAACAACGGAGGCAATTCTGC	GATGTTGTTGCCTCCGTTAAGA	tag250101
novel_sir231	AAACATCTATATGGCCAATTCTCC	GGTTTGTAGATATACCGGTTAAGA	tag183551
novel_sir232	AAGAGCAGCGGGCTACAATTCTGT	TCTTCTCGTCGCCCGATGTTAAGA	tag115751
novel_sir233	TGTGAACCGATCTGAGATTTAAGA	ACTTGGCTAGACTCTAAATTCTTA	tag92610
novel_sir234	GTTTTCGCTGAAACACGCCACAGA	AAAGCGACTTTGTGCGGTGTCTTA	tag217757
novel_sir235	GTTGTACACACCTTAGCACAGA	ACATGTGTGGAATCGTGTCTTG	tag154856
novel_sir236	CCTGATAAACGGCGCCTTGACAGA	ACTATTTGCCGCGGAACTGTCTAT	tag263621
novel_sir237	GTTTTGAAGTAAACTGGCACCAGA	AAACTTCATTTGACCGTGGTCTTG	tag202866

novel_sir238	GCTGCTGCCACTCGGAGCCAGA	ACGACGGTGAGCCTCGGTCTTT	tag249131
novel_sir239	GGTGTAGGTAGGTGAAAATCCAGA	ACATCCATCCACTTTTAGGTCTCG	tag71043
novel_sir240	GATGTAGGTAGGTGAAAATCCAGA	ACATCCATCCACTTTTAGGTCTCG	tag112203
novel_sir241	AATATCACAGCAACAGAGGTCTCT	TCTTATAGTGTCGTTGTCTCCAGA	tag13073
novel_sir242	AAGCTCTGAACCCAAGTCGTCTAG	ACTTCGAGACTTGGGTTCAGCAGA	tag137096
novel_sir243	ACTTCGAAGCTTGGGTTCAGCAGA	AAGCTTCGAACCCAAGTCGTCTAG	tag146753
novel_sir244	TTTGGTTGAAAACCGTTACGCAGA	ACCAACTTTTGGCAATGCGTCTAG	tag59704
novel_sir245	CATTGGCAGCTGTCGCTCCGCAGA	AACCGTCGACAGCGAGGCGTCTGT	tag133893
novel_sir246	ACTTCTCGCCGCTTCTCAGTCTGG	CCTGAAGAGCGGCGAAGAGTCAGA	tag232795
novel_sir247	CCTGAAGAGCGGCGAAGTGTCAGA	ACTTCTCGCCGCTTCACAGTCTGG	tag51553
novel_sir248	CATTGAAGTGCTCACTTTGTCAGA	AACTTCACGAGTGAAACAGTCTGA	tag172322
novel_sir249	GATTTGCTAAACCCAAGCTTCAGA	AAACGATTTGGGTTCGAAGTCTCA	tag11895
novel_sir250	AAACGATCTGGGTTCGAAGTCTCA	GATTTGCTAGACCCAAGCTTCAGA	tag273525
novel_sir251	AACCGGCCTGGGTTCGAAGTCTCA	GATTGGCCGGACCCAAGCTTCAGA	tag59291
novel_sir252	AATGGTTATCTATAGGTTCTCTAT	TGTTACCAATAGATATCCAAGAGA	tag158057
novel_sir253	GTTGGGCGACTTTACAGTAAGAGA	ACCCGCTGAAATGTCATTCTCTTC	tag123520
novel_sir254	TGTTGAAGAGGTGGTTGTAAGAGA	AACTTCTCCACCAACATTCTCTTC	tag265669
novel_sir255	AATGGTTATCTATAGGCTCTCTAT	TGTTACCAATAGATATCCGAGAGA	tag8892
novel_sir256	AATGGTTATCTATAGGCTCTCTAT	TATTACCAATAGATATCCGAGAGA	tag28574
novel_sir257	AATGGTTATCTATAGGCTCTCTAT	TCTTACCAATAGATATCCGAGAGA	tag242912
novel_sir258	AATGGTTATCTATAGGCTCTCTAT	CGTTACCAATAGATATCCGAGAGA	tag265098
novel_sir259	ACTTTTTAGTCACATCATCTCTAA	GTTGAAAAATCAGTGTAGTAGAGA	tag71857
novel_sir260	ACTTTTCAGTCACATCATCTCTAA	ATTGAAAAGTCAGTGTAGTAGAGA	tag186744
novel_sir261	AATTAACGATTTGCCATGCTCTGT	CGTTAATTGCTAAACGGTACGAGA	tag5181
novel_sir262	GGTTCTCTGACCGATTTACCGAGA	AAGAGACTGGCTAAATGGCTCTCC	tag70196
novel_sir263	CGTTAATTGCTAAACGGTGCGAGA	AATTAACGATTTGCCACGCTCTGT	tag155668
novel_sir264	GGTGAGCTTGATAACCTTAGGAGA	ACTCGAACTATTGGAATCCTCTCC	tag279641
novel_sir265	ACTCTCGTTGGCGCTGACCTCTGT	GGTGAGAGCAACCGCGACTGGAGA	tag193168
novel_sir266	AAGCCACCGCCGCCACCTCTCG	GGTTCGGTGGCGGCGGTGGAGA	tag240499
novel_sir267	GGTTGTCGGAGAGATTAACTGAGA	AACAGCCTCTCTAATTGACTCTCC	tag257542
novel_sir268	GGTTTGTGAGTATATCGGTTGAGA	AAACACTCATATAGCCAACTCTCC	tag51866
novel_sir269	AAACACCCATATAGCCAACTCTCC	GGTTTGTGGGTATATCGGTTGAGA	tag259052
novel_sir270	AAACACCCATATAGCCAACTCTTC	GGTTTGTGGGTATATCGGTTGAGA	tag259052
novel_sir271	AAAAGACTAGGCCGGTGTATCTTG	GATTTTCTGATCCGGCCACATAGA	tag176562

novel_sir272	ACTGTGCCAGATGTTCTATATAGA	ACACGGTCTACAAGATATATCTTT	tag145080
novel_sir273	ATTGTGCCAGATGTTCTATATAGA	ACACGGTCTACAAGATATATCTTT	tag210038
novel_sir274	AAATTTATAGCCCCGCTGATCTTC	TGTTTAAATATCGGGGCGACTAGA	tag168776
novel_sir275	TCTTCTCGTCGCCCGATATCTAGA	AAGAGCAGCGGGCTATAGATCTGT	tag226148
novel_sir276	TCTTTTCGTCGCCTGATATCTAGA	AAAAGCAGCGGACTATAGATCTGT	tag201380
novel_sir277	TCTTTTCGTCGCCTGATATCTAGA	AAAAGCAGCGGACTATAGATCTAT	tag201380
novel_sir278	AATCGGGACTCAAGATAGATCTTT	TATTAGCCCTGAGTTCTATCTAGA	tag291649
novel_sir279	TTTGGTGTGGAAGCGGACTCTAGA	ACCACACCTTCGCCTGAGATCTGC	tag248342
novel_sir280	CTTTTGTGCTCCGGACCCTCTAGA	AAACACGAGGCCTGGGAGATCTGC	tag248448
novel_sir281	TCTTATCGTCACCCGATGTCTAGA	AATAGCAGTGGGCTACAGATCTGT	tag197715
novel_sir282	AAGAGCAGTGGGCTACAGATCTGT	TCTTCTCGTCACCCGATGTCTAGA	tag72407
novel_sir283	AATAGCAGCGGGCTACAGATCTGT	TCTTATCGTCGCCCGATGTCTAGA	tag71433
novel_sir284	TCTTCTCGTCGCCCGATGTCTAGA	AAGAGCAGCGGGCTACAGATCTGT	tag121974
novel_sir285	TCTTTTCGTCGCCCGATGTCTAGA	AAAAGCAGCGGGCTACAGATCTGT	tag84948
novel_sir286	TCTTCTCGTTGCCCGATGTCTAGA	AAGAGCAACGGGCTACAGATCTGT	tag62435
novel_sir287	TCTTCTCGTCGCTCGATGTCTAGA	AAGAGCAGCGAGCTACAGATCTGT	tag173671
novel_sir288	TCTTCGCAGTGCTCGATGTCTAGA	AAGCGTCACGAGCTACAGATCTGT	tag258447
novel_sir289	TCTTTTCGTTGTTCGATGTCTAGA	AAAAGCAACAAGCTACAGATCTGT	tag14467
novel_sir290	CCTTCTCGTCGCCTGATGTCTAGA	AAGAGCAGCGGACTACAGATCTGT	tag74141
novel_sir291	TCTTCTCGTCGCCTGATGTCTAGA	AAGAGCAGCGGACTACAGATCTGT	tag105029
novel_sir292	GTTGGCCCTGATTTCAATTCTAGA	ACCGGGACTAAAGTTAAGATCTTT	tag6591
novel_sir293	AACTTGTAGCGGTATGTCATCTAG	ATTTGAACATCGCCATACAGTAGA	tag212276
novel_sir294	AGTGCTTAACTCAGTGAACGTAGA	ACGAATTGAGTCACTTGCATCTTA	tag77094
novel_sir295	AACTAGCTGCTTGTAAGAATCTTA	AGTTGATCGACGAACATTCTTAGA	tag140788
novel_sir296	AAACTCGGCTCAATCTGA	CATTTGAGCCGAGTTAGA	tag26814
novel_sir297	AAACTCGGCTCAATCTGA	ATTTTGAGCCGAGTTAGA	tag140526
novel_sir298	AAACTCGGCTCAATCTGA	AATTTGAGCCGAGTTAGA	tag292117
novel_sir299	AATTAAACTCGGCTCAATCTGA	CATTAATTTGAGCCGAGTTAGA	tag96180
novel_sir300	TATTACTACGATCGGCACGTTAGA	AATGATGCTAGCCGTGCAATCTGC	tag56570
novel_sir301	AATGATGTTAGCCGCGCAATCTGC	TATTACTACAATCGGCGCGTTAGA	tag96119
novel_sir302	AATAATGTTAGCCGCGCAATCTGC	TATTATTACAATCGGCGCGTTAGA	tag186487
novel_sir303	TATTACTACGATCGGCGCGTTAGA	AATGATGCTAGCCGCGCAATCTGC	tag62221
novel_sir304	AATGATACTAGCCGCGCAATCTGT	TATTACTATGATCGGCGCGTTAGA	tag216064
novel_sir305	TATTACTATGATCGGCGCGTTAGA	AATGATACTAGCCGCGCAATCTGC	tag216064

novel_sir306	TATTGCTATGATCGGCGCGTTAGA	AACGATACTAGCCGCGCAATCTGC	tag183686
novel_sir307	TATTGCTATGATCGGCGCGTTAGA	AACGATACTAGCCGCGCAATCTAT	tag183686
novel_sir308	TATTATTATGATCGGCGCGTTAGA	AATAATACTAGCCGCGCAATCTGC	tag206796
novel_sir309	TATTGGCTACACCACGAATTTAGA	AACCGATGTGGTGCTTAAATCTTC	tag188152
novel_sir310	AATTTTTCGCACGGTGCTTTTAGA	AAAAAGCGTGCCACGAAAATCTTA	tag255402
novel_sir311	AATGGGTGAACAGGCTGACAACGA	ACCCACTTGTCCGACTGTTGCTAA	tag186549
novel_sir312	AGACGAAAGTGCCCATGTTGCTGA	CTTCTGCTTTCACGGGTACAACGA	tag258474
novel_sir313	AGAAATACCCAATATCTTGCTGA	AATCTTTATGGGTTATAGAACGA	tag46331
novel_sir314	AGAAATACCCAATATCTTGCTGA	CTTCTTTATGGGTTATAGAACGA	tag108939
novel_sir315	AGAAATACCCAATATCTTGCTGA	TCTCTTTATGGGTTATAGAACGA	tag160641
novel_sir316	AGAAATACCCAATATCTTGCTGA	TTTCTTTATGGGTTATAGAACGA	tag176210
novel_sir317	AGAAATACCCAATATCTTGCTGA	CATCTTTATGGGTTATAGAACGA	tag246182
novel_sir318	AGAAATACCCAATATCTTGCTGA	ATTCTTTATGGGTTATAGAACGA	tag284615
novel_sir319	AACTCATCGTAGCCGGCTTGCTCC	TTTTGAGTAGCATCGGCCGAACGA	tag286253
novel_sir320	CTTGAGGCTTCAATTCGCACGA	ACTCCGAAGTTAAGCGTGCTTG	tag90199
novel_sir321	TGTGACACAGAACCTCAGGCACGA	ACTGTGTCTTGGAGTCCGTGCTGC	tag290804
novel_sir322	TGTGACACAGAACCTCAGGCACGA	ACTGTGTCTTGGAGTCCGTGCTAC	tag290804
novel_sir323	TGTGACGCAGAACCTCAGGCACGA	ACTGCGTCTTGGAGTCCGTGCTGC	tag192336
novel_sir324	TGTGACGCAGAACCTCAGGCACGA	ACTGCGTCTTGGAGTCCGTGCTAC	tag192336
novel_sir325	ACTGCGTCTTGGAGTCCGTGCTAT	TGTGACGCAGAACCTCAGGCACGA	tag192336
novel_sir326	TATGACACAGAATCTCAGGCACGA	ACTGTGTCTTAGAGTCCGTGCTAC	tag33842
novel_sir327	TGTGACACAGAATCTCAGGCACGA	ACTGTGTCTTAGAGTCCGTGCTAC	tag153008
novel_sir328	GGTTATGCAGAACCTTAGGCACGA	AATACGTCTTGGAATCCGTGCTGC	tag214128
novel_sir329	TATGACGCAGAATCTTAGGCACGA	ACTGCGTCTTAGAATCCGTGCTGC	tag256033
novel_sir330	AATCGATCGTGTAGTTAGTGCTTG	TTTTAGCTAGCACATCAATCACGA	tag101402
novel_sir331	ACTGTTTCCTGTATCGAGTGCTTC	GTTGACAAAGGACATAGCTCACGA	tag14696
novel_sir332	TGTGTGGCCGGACCCTCTAGACGA	ACACCGGCCTGGGAGATCTGCTTA	tag202512
novel_sir333	ACCCTCTAGATCCGCCACTGCTTA	GGTGGGAGATCTAGGCGGTGACGA	tag23700
novel_sir334	AATACGTCTTAGAGTCTATGCTAA	TGTTATGCAGAATCTCAGATACGA	tag109893
novel_sir335	TGTTAGACGCGCCCGGTGATACGA	AATCTGCGCGGGCCACTATGCTAG	tag194740
novel_sir336	CGTTAGACGCGCCCGGTGATACGA	AATCTGCGCGGGCCACTATGCTAG	tag289377
novel_sir337	TGTTAGACGCGCCCGGTGATACGA	AATCTGCGCGGGCCACTATGCTTG	tag194740
novel_sir338	CGTTAGACGCGCCCGGTGATACGA	AATCTGCGCGGGCCACTATGCTTG	tag289377
novel_sir339	ACTGCGTCTTAGAGTCCATGCTGT	TGTGACGCAGAATCTCAGGTACGA	tag81958

novel_sir340	CGTTAGACGCGCCCGGTGGTACGA	AATCTGCGCGGGCCACCATGCTAG	tag168118
novel_sir341	ACACCTTAGACATGGTTTGGCTAA	GGTGTGGAATCTGTACCAAACCGA	tag147927
novel_sir342	AACAAAGTCAAAGGGGTTGGCTCT	TCTTGTTTCAGTTTCCCCAACCGA	tag274513
novel_sir343	AGTTATAAGTCATGTTTCAACCGA	AATATTCAGTACAAAGTTGGCTTT	tag236773
novel_sir344	GCTGACCACATATTCACCGA	ACTGGTGTATAAGTGGCTTT	tag213424
novel_sir345	TATTGTATCCAGGGCGTAGACCGA	AACATAGGTCCCGCATCTGGCTTG	tag8506
novel_sir346	AATTAACGGTCTAGCGATGGCTTC	CCTTAATTGCCAGATCGCTACCGA	tag127623
novel_sir347	AGACTTCGAACTCAGGTCGGCTAG	ATTCTGAAGCTTGAGTCCAGCCGA	tag284492
novel_sir348	GATTGAACCCATGTAACGAGCCGA	AACTTGGGTACATTGCTCGGCTTG	tag104233
novel_sir349	AAGACTAAACAAACAAGCGGCTTT	GATTCTGATTTGTTTGTTCGCCGA	tag45134
novel_sir350	ACTCTGAAGCTTGAGTCTGGCCGA	AGACTTCGAACTCAGACCGGCTAG	tag15149
novel_sir351	ACTCTAACACAGTCTGCAGGCTAC	TTTGAGATTGTGTCAGACGTCCGA	tag210441
novel_sir352	CTTTTTCCTCGGAGGTGCAAGCGA	AAAAGGAGCCTCCACGTTCGCTCT	tag76749
novel_sir353	AAAAGAAGGCACCACGTTCGCTGT	GCTTTTCTTCCGTGGTGCAAGCGA	tag229966
novel_sir354	AACAAATTTGGACGGATTCGCTGT	TGTTGTTTAAACCTGCCTAAGCGA	tag102479
novel_sir355	AACTTGCATACGCTCGGTCGCTCC	GCTTGAACGTATGCGAGCCAGCGA	tag255854
novel_sir356	AATTAGTCTTAGTCTGATCGCTTT	TTTTAATCAGAATCAGACTAGCGA	tag158911
novel_sir357	GCTGGCCGACTACGCACGCGA	ACCGGCTGATGCGTGCGCTGC	tag150756
novel_sir358	GCTGGGAAGACGGCCGCTACGCGA	ACCCTTCTGCCGGCGATGCGCTCC	tag104317
novel_sir359	AGACCGGATAAACGAGCGCGCTGC	TGTCTGGCCTATTTGCTCGCGCGA	tag21779
novel_sir360	TGTTACACAGAATCTAAGGCGCGA	AATGTGTCTTAGATTCCGCGCTGC	tag184306
novel_sir361	TGTCTGGCCTATTTCCTTGCGCGA	AGACCGGATAAAGGAACGCGCTGT	tag42619
novel_sir362	AGACCGATCCCTAGAGCGCTTG	CCTCTGGCTAGGGATCTCGCGA	tag57069
novel_sir363	AAGATCTGCACCGACGGCCGCTCC	GGTTCTAGACGTGGCTGCCGGCGA	tag159798
novel_sir364	TCTTCGGGAACAGCCATGCGA	AAGCCCTTGTCGGTACGCTTG	tag245100
novel_sir365	GCTGGCCTTGATCGGGCACTGCGA	ACCGGAACTAGCCCGTGACGCTCC	tag191310
novel_sir366	AATTTTCGTTGGTGGCCACGCTAA	AGTTAAAAGCAACCACCGGTGCGA	tag177891
novel_sir367	AATCCGGCACACGGTAGCTTC	TATTAGGCCGTGTGCCATCGA	tag57381
novel_sir368	CCTGGCAAACCGTGTTGAGATCGA	ACCGTTTGGCACAACTCTAGCTCT	tag28639
novel_sir369	TGTCGGAGCTGAGCATAGGATCGA	AGCCTCGACTCGTATCCTAGCTCG	tag84021
novel_sir370	TGTCGGAGCTGAGCATAGGATCGA	AGCCTCGACTCGTATCCTAGCTCA	tag84021
novel_sir371	ACCGGTCCTTAGCTTCTGAGCTAG	AGTGGCCAGGAATCGAAGACTCGA	tag259894
novel_sir372	TTTTACATGATCTCAACACCTCGA	AATGTACTAGAGTTGTGGAGCTGG	tag121421
novel_sir373	TTTTACTTGATCTCAACACCTCGA	AATGAACTAGAGTTGTGGAGCTGG	tag68705

novel_sir374	TTTCGATCTGACAAACTGCCTCGA	AGCTAGACTGTTTGACGGAGCTTC	tag24988
novel_sir375	CATCTCCTACTGCAGCGCTCGA	AGAGGATGACGTCGCGAGCTCA	tag151279
novel_sir376	TCTGACTTTGACAAACCGGCTCGA	ACTGAAACTGTTTGGCCGAGCTCC	tag14743
novel_sir377	AAGGATTACACGGCGTAGAGCTGT	GATTCCTAATGTGCCGCATCTCGA	tag109777
novel_sir378	ACCGTTTGGTTGGACTTCAGCTCC	CTTGGCAAACCAACCTGAAGTCGA	tag162501
novel_sir379	ACACCTCAGCGCCACGTCAGCTGG	TCTGTGGAGTCGCGGTGCAGTCGA	tag149448
novel_sir380	ACACCTCAACGCCACGTCAGCTGG	TCTGTGGAGTTGCGGTGCAGTCGA	tag78294
novel_sir381	CCTGGCAAACCGTGTCGAGGTCGA	ACCGTTTGGCACAGCTCCAGCTCT	tag175189
novel_sir382	CCTGGCAAACCGTGTCGAGGTCGA	ACCGTTTGGCACAGCTCCAGCTCC	tag175189
novel_sir383	ACCAGCTCCGGATCCAGCTCC	CGTGGTCGAGGCCTAGGTCGA	tag60865
novel_sir384	GATGTCTAGACATCGATCGGTCGA	ACAGATCTGTAGCTAGCCAGCTGC	tag264290
novel_sir385	AATACTACGAATCTGGACAGCTAG	GATTATGATGCTTAGACCTGTCGA	tag252060
novel_sir386	AGAACAGACTGAATCGGAAGCTAC	GGTCTTGTCTGACTTAGCCTTCGA	tag8588
novel_sir387	TTTTCGGTCACCGCGCTTCGA	AAGCCAGTGGCGCGAAGCTAC	tag208108
novel_sir388	CTTTTCGGTCACCGCGCTTCGA	AAAGCCAGTGGCGCGAAGCTAC	tag58416
novel_sir389	AAGTCTATGTCCTAGGAAAGCTGC	CCTTCAGATACAGGATCCTTTCGA	tag63908
novel_sir390	TTTCGACGCTGTTTGAAAGGA	AGCTGCGACAAACTTTCCTGG	tag71826
novel_sir391	ATTGGACAGTCACTGGTGCAAGGA	ACCTGTCAGTGACCACGTTCCTCT	tag275447
novel_sir392	ACTGTCTGGCGCCGGAGTTCCTCT	GATGACAGACCGCGGCCTCAAGGA	tag154477
novel_sir393	ACGATGGCCGGGACTTCCTCT	GCTGCTACCGGCCCTGAAGGA	tag42332
novel_sir394	ACCAGCCAAGACTTATTCCTAA	GTTGGTCGGTTCTGAATAAGGA	tag138499
novel_sir395	TTTTGAGTGAAGCACGCCACAGGA	AACTCACTTCGTGCGGTGTCCTGG	tag122603
novel_sir396	TTTTGGTGTTTCAGCGCTACAGGA	AACCACAAAGTCGCGATGTCCTGT	tag275301
novel_sir397	AAAACCGTTCGCTTGAGGTCCTCT	TGTTTTGGCAAGCGAACTCCAGGA	tag247921
novel_sir398	AAAACCGTTCGCTTGAGGTCCTTC	TGTTTTGGCAAGCGAACTCCAGGA	tag247921
novel_sir399	CGTGAGAGCCACATCAGCGCAGGA	ACTCTCGGTGTAGTCGCGTCCTTT	tag207394
novel_sir400	ATTTGTAGGCTATACTATTCAGGA	AACATCCGATATGATAAGTCCTGT	tag193112
novel_sir401	ACTGCTGCCACGTTGAAGTCCTGG	AGTGACGACGGTGCAACTTCAGGA	tag79446
novel_sir402	CATTCAAATGTCGGGCGAAGAGGA	AAGTTTACAGCCCGCTTCTCCTCT	tag225078
novel_sir403	ACCGCTCGGCCATCTCTCCTCC	CTTGGCGAGCCGGTAGAGAGGA	tag53817
novel_sir404	AACCGCTCGGCCATCTCTCCTCC	ACTTGGCGAGCCGGTAGAGAGGA	tag218610
novel_sir405	TGTTTAGATATCAGGCGACGAGGA	AAATCTATAGTCCGCTGCTCCTCT	tag182992
novel_sir406	GGTCGAGGCCTAGGTCGAGGA	AGCTCCGGATCCAGCTCCTGT	tag185190
novel_sir407	ACCAGCTCCGGATCCAGCTCCTGT	CGTGGTCGAGGCCTAGGTCGAGGA	tag115696

novel_sir408	ATTGAGTCTGAAATCCGCCTAGGA	ACTCAGACTTTAGGCGGATCCTTA	tag260680
novel_sir409	GGTTCGGTTTTGCTATAGCTAGGA	AAGCCAAAACGATATCGATCCTAT	tag66431
novel_sir410	ACGTACCTACATAGCCGATCCTGT	CATGCATGGATGTATCGGCTAGGA	tag72638
novel_sir411	AAGCCGAAATGATACCGATCCTAC	GGTTCGGCTTTACTATGGCTAGGA	tag35321
novel_sir412	TGTTTTGGCAAGCGAACTCTAGGA	AAAACCGTTCGCTTGAGATCCTCC	tag160772
novel_sir413	ACTCCTCCGTTGCCATCCTCG	GGTGAGGAGGCAACGGTAGGA	tag22749
novel_sir414	GGTTGCCCGCCGCCCGACTTAGGA	AACGGGCGGCGGGCTGAATCCTTT	tag161308
novel_sir415	TGTCTGCTTCGGTTATACAACGGA	AGACGAAGCCAATATGTTGCCTTG	tag271607
novel_sir416	AGTGACAGCTACCTATGCAACGGA	ACTGTCGATGGATACGTTGCCTAC	tag220339
novel_sir417	CTTTTAGCGAATTCACCAGACGGA	AAATCGCTTAAGTGGTCTGCCTGC	tag262174
novel_sir418	GCTTGGCACTTTCGCACCGGA	AACCGTGAAAGCGTGGCCTAT	tag4995
novel_sir419	AATTCAACGTAATTAATGGCCTTG	AGTTAAGTTGCATTAATTACCGGA	tag261068
novel_sir420	AATGCAGCAGAACTGTTCCGGA	ACGTCGTCTTGACAAGGCCTCC	tag83860
novel_sir421	TATGACAGTACCAGCTCGAGCGGA	ACTGTCATGGTCGAGCTCGCCTGA	tag244251
novel_sir422	CATGGGCAGCTGTCGTTACGCGGA	ACCCGTCGACAGCAATGCGCCTGT	tag82537
novel_sir423	GGTTCGATCGTTCGCCGCGGA	AAGCTAGCAAGCGGCGCCTGC	tag35644
novel_sir424	AACCGTCGACAGTGAGGCGCCTGT	CATTGGCAGCTGTCACTCCGCGGA	tag18971
novel_sir425	CATGGGCAGCTGTCGCTCCGCGGA	ACCCGTCGACAGCGAGGCGCCTGT	tag63423
novel_sir426	CTTGGGCAGCTGTCGCTCCGCGGA	ACCCGTCGACAGCGAGGCGCCTGT	tag161186
novel_sir427	AGGAACCCCGGCCGCGCCTCC	ACTCCTTGGGGCCGGCGCGGA	tag41414
novel_sir428	CATGGGCAGCTGTCGCTTCGCGGA	ACCCGTCGACAGCGAAGCGCCTGT	tag198487
novel_sir429	GGTGACAATAGTTAGCAATGCGGA	ACTGTTATCAATCGTTACGCCTCC	tag90934
novel_sir430	CATGGGCAGCTGTCGCTCTGCGGA	ACCCGTCGACAGCGAGACGCCTGT	tag30833
novel_sir431	ACCCGTCGACAGCGAAACGCCTGT	CATGGGCAGCTGTCGCTTTGCGGA	tag7803
novel_sir432	AGACGAATCTTTTGAGCCTAA	GCTCTGCTTAGAAAACTCGGA	tag35226
novel_sir433	GGTGAGGCCGTCAGCGTCGGA	ACTCCGGCAGTCGCAGCCTCC	tag140176
novel_sir434	AGACGAATCTTTTAAGCCTAA	GCTCTGCTTAGAAAATTCGGA	tag84809
novel_sir435	GGTTTGCTGGACACAAACTTCGGA	AAACGACCTGTGTTTGAAGCCTCA	tag16804
novel_sir436	AGACGATTTTGGTTTGAAGCCTTG	GATCTGCTAAAACCAAACTTCGGA	tag116435
novel_sir437	AGACGACCTAGATTTGAAGCCTCA	GATCTGCTGGATCTAAACTTCGGA	tag131128
novel_sir438	GATCTGCTAGACCCAAGCTTCGGA	AGACGATCTGGGTTCGAAGCCTCA	tag144569
novel_sir439	GATCTGCTGGATCCAAGCTTCGGA	AGACGACCTAGGTTCGAAGCCTCA	tag140594
novel_sir440	AGACGATTTGAGTTCGAAGCCTCA	GATCTGCTAAACTCAAGCTTCGGA	tag171169
novel_sir441	AGCTAGCCTAGATTCGAAGCCTCA	GATCGATCGGATCTAAGCTTCGGA	tag210486

novel_sir442	AGACGACCTAGATTCGAAGCCTCG	GATCTGCTGGATCTAAGCTTCGGA	tag167497
novel_sir443	AGACGACCTGAGTTTAAAGCCTCA	GATCTGCTGGACTCAAATTTCGGA	tag77937
novel_sir444	GATTTGCTAAACCCAAGTTTCGGA	AAACGATTTGGGTTCAAAGCCTCA	tag162257
novel_sir445	AGACGACCTGGGTTCAAAGCCTCA	GATCTGCTGGACCCAAGTTTCGGA	tag263331
novel_sir446	GCTGCGGAGAACAGAGAGGGA	ACGCCTCTTGTCTCTCCCTCC	tag6845
novel_sir447	ACTGAAAATTTGTCTGATCCCTTG	GGTGACTTTTAAACAGACTAGGGA	tag199563
novel_sir448	ACCAAGCTGCGCTACATCCCTTT	AATGGTTCGACGCGATGTAGGGA	tag105623
novel_sir449	TGTGGCTCAAACAAACTGTAGGGA	ACCGAGTTTGTTTGACATCCCTTA	tag287687
novel_sir450	AATGTTAGATACTGCACACCGGGA	ACAATCTATGACGTGTGGCCCTTG	tag207685
novel_sir451	AATCGTTCAGGCGAGACCGGGA	AGCAAGTCCGCTCTGGCCCTGG	tag39717
novel_sir452	TATCGTTCAGGCGAGACCGGGA	AGCAAGTCCGCTCTGGCCCTGG	tag113434
novel_sir453	AAAAATTTTCTACGTAGCCCTTT	TATTTTTAAAAAGATGCATCGGGA	tag22283
novel_sir454	GATGGCTTGACTCGATATCGGGA	ACCGAACTGAGCTATAGCCCTTG	tag179092
novel_sir455	GGTTACATAAACCGCGACTGGGGA	AATGTATTTGGCGCTGACCCCTTG	tag259405
novel_sir456	ACGTGCCAGTCAGTTTGACCCTGC	CCTGCACGGTCAGTCAAACTGGGA	tag17630
novel_sir457	CGTTAATTGCTAAACGGTGTGGGA	AATTAACGATTTGCCACACCCTGT	tag238952
novel_sir458	TGTTGGTGTCAAGCTGTCCATGGA	AACCACAGTTCGACAGGTACCTGT	tag106644
novel_sir459	GTTGTGAAACTAGGCCGAGATGGA	ACACTTTGATCCGGCTCTACCTAA	tag4416
novel_sir460	GGTCGACGTACAATGGGCTATGGA	AGCTGCATGTTACCCGATACCTGT	tag27953
novel_sir461	ACCTCCAGCCATTTAGGGACCTTT	GTTGGAGGTCGGTAAATCCCTGGA	tag288821
novel_sir462	GGTCGGTGTACTGGCCCAGCTGGA	AGCCACATGACCGGGTCGACCTCC	tag79194
novel_sir463	GGTGCCGTACCCACCGCTGGA	ACGGCATGGGTGGCGACCTCG	tag123719
novel_sir464	AGACGTTGAACCATAGAGACCTAT	AGTCTGCAACTTGGTATCTCTGGA	tag194778
novel_sir465	ATTCCGCTGACGATCGCTTCTGGA	AGGCGACTGCTAGCGAAGACCTGT	tag110947
novel_sir466	CATTCAAATGTCGGGCGAAGTGGA	AAGTTTACAGCCCGCTTCACCTCT	tag182024
novel_sir467	GTTCTTAGGTCTGAGATCAGTGGA	AGAATCCAGACTCTAGTCACCTTC	tag289527
novel_sir468	GGTGGGCAAAGAACGTACGGTGGA	ACCCGTTTCTTGCATGCCACCTAA	tag78132
novel_sir469	AATATAAATACCGCGGTAACCTCC	CATTATATTTATGGCGCCATTGGA	tag177233
novel_sir470	GATCTGCTGGATCCAAGCTTTGGA	AGACGACCTAGGTTCGAAACCTGA	tag195745
novel_sir471	AAGCCTTGACAACTGAGTTACTTT	GTTTCGGAACTGTTGACTCAATGA	tag224975
novel_sir472	CGTGGGCAGCAACGTGGCGAATGA	ACCCGTCGTTGCACCGCTTACTCT	tag59463
novel_sir473	AGGTCACCCATCCTAGTACTAC	CCTCCAGTGGGTAGGATCATGA	tag6851
novel_sir474	ACTTTATATGGCCTCGTCTACTAC	AGTGAAATATACCGGAGCAGATGA	tag134799
novel_sir475	ACACGATCTCTAATATGCTACTTT	GTTGTGCTAGAGATTATACGATGA	tag243937

novel_sir476	AATCTGCGCGGGCCACTATACTTG	CGTTAGACGCGCCCGGTGATATGA	tag290026
novel_sir477	CGTGTAACCGGGTGCGCAGTATGA	ACATTGGCCCACGCGTCATACTCT	tag253962
novel_sir478	AATCTGCGCGGGCCACCATACTAG	CGTTAGACGCGCCCGGTGGTATGA	tag250455
novel_sir479	AATCTGCGCGGACCACCATACTAG	TGTTAGACGCGCCTGGTGGTATGA	tag227759
novel_sir480	ACTGAAAAACAGGTTGCAAACTGA	ACTTTTTGTCCAACGTTTGACTAT	tag214825
novel_sir481	ACTGAAAAGCAGGTTGCAAACTGA	ACTTTTCGTCCAACGTTTGACTAT	tag192381
novel_sir482	TCTGAAAAGCAGGTTGCAAACTGA	ACTTTTCGTCCAACGTTTGACTAT	tag251841
novel_sir483	ACGTTCCTGGCCTGGGTTGACTGT	CCTGCAAGGACCGGACCCAACTGA	tag216151
novel_sir484	AGTTTTACAAACTGCGGCAACTGA	AAAATGTTTGACGCCGTTGACTTT	tag19148
novel_sir485	GGTTTTACAAACTGCGGCAACTGA	AAAATGTTTGACGCCGTTGACTTT	tag31758
novel_sir486	TGTTTTACAAACTGCGGCAACTGA	AAAATGTTTGACGCCGTTGACTTT	tag291132
novel_sir487	GGTTTTATAAACTGCGGCAACTGA	AAAATATTTGACGCCGTTGACTTT	tag2873
novel_sir488	AGTTTTATAAACTGCGGCAACTGA	AAAATATTTGACGCCGTTGACTTT	tag267405
novel_sir489	GGTTTTATAAATTGCGGCAACTGA	AAAATATTTAACGCCGTTGACTTT	tag121395
novel_sir490	GGTTTTATAAACTGTGGCAACTGA	AAAATATTTGACACCGTTGACTTT	tag26566
novel_sir491	AGTTTTATAAACTGTGGCAACTGA	AAAATATTTGACACCGTTGACTTT	tag253778
novel_sir492	GTTGATCCGGGCAGTGTCTACTGA	ACTAGGCCCGTCACAGATGACTCA	tag233717
novel_sir493	AAAAAGAGAATAGCAGCGGACTAC	TCTTTTTCTCTTATCGTCGCCTGA	tag209485
novel_sir494	AGGCCTCGATCCTATGTCGACTCT	CCTCCGGAGCTAGGATACAGCTGA	tag38711
novel_sir495	TCTCCAGTGAAGGCCGACATCTGA	AGGTCACTTCCGGCTGTAGACTAG	tag215128
novel_sir496	TGTCTAAGAGTTTTCGATATCTGA	AGATTCTCAAAAGCTATAGACTGT	tag8527
novel_sir497	AGGCCTGTTAATTCGTGAGACTTG	CCTCCGGACAATTAAGCACTCTGA	tag107186
novel_sir498	AACCCATTTCGTGCTGCAGACTGC	GGTTGGGTAAAGCACGACGTCTGA	tag58939
novel_sir499	AACGCCGCGTGGAATGAAGACTCG	AGTTGCGGCGCACCTTACTTCTGA	tag182286
novel_sir500	AGTTGGGTACACTGGGGCTTCTGA	AACCCATGTGACCCCGAAGACTTG	tag285261
novel_sir501	AACGCCACGCGTAAAGAAGACTCG	AGTTGCGGTGCGCATTTCTTCTGA	tag29593
novel_sir502	ATTTTAAGTTGAATCTCTCAGTGA	AAATTCAACTTAGAGAGTCACTTG	tag57263
novel_sir503	ACTGATACTAGGAGGCCTCACTCT	TTTGACTATGATCCTCCGGAGTGA	tag14040
novel_sir504	CCTGCGGTTGGCCATTACTAGTGA	ACGCCAACCGGTAATGATCACTAT	tag28203
novel_sir505	CCTCACTGCCGCGGTCCGTGA	AGTGACGGCGCCAGGCACTGG	tag226024
novel_sir506	AATTACAAAGAGAGGACGCACTTT	TATTAATGTTTCTCTCCTGCGTGA	tag38068
novel_sir507	AATCAACGGGTCGACATCCACTCG	TGTTAGTTGCCCAGCTGTAGGTGA	tag203099
novel_sir508	AGAGAATCCACTTAGTGCCACTGA	TCTCTCTTAGGTGAATCACGGTGA	tag203899
novel_sir509	AGCAAGGCCACTCTGCCACTTA	CGTCGTTCCGGTGAGACGGTGA	tag53102

novel_sir510	AGCAAGGCCACTCTGCCACTTA	TGTCGTTCCGGTGAGACGGTGA	tag290634
novel_sir511	ACCACCCTCTAGATCCGCCACTGC	CCTGGTGGGAGATCTAGGCGGTGA	tag285127
novel_sir512	GCTGGAGCTGTAGTCATAGGGTGA	ACCTCGACATCAGTATCCCACTCG	tag243285
novel_sir513	GTTCTGGCCTCGATAGTTGGTGA	AGACCGGAGCTATCAACCACTCA	tag37596
novel_sir514	ATTCTGGCCTCGATAGTTGGTGA	AGACCGGAGCTATCAACCACTCA	tag212911
novel_sir515	CGTCGTGCCTGAGGTTCTATGTGA	AGCACGGACTCCAAGATACACTGT	tag194261
novel_sir516	CGTGAAAAGCGGTGCTGGGTGTGA	ACTTTTCGCCACGACCCACACTAC	tag59356
novel_sir517	CGTCGTGCCTGAGGTTCTGTGTGA	AGCACGGACTCCAAGACACACTGT	tag58598
novel_sir518	CATCGTGCCTGAGGTTCTGTGTGA	AGCACGGACTCCAAGACACACTGT	tag189883
novel_sir519	CATGGCAGCACTGGTTTATTGTGA	ACCGTCGTGACCAAATAACACTGC	tag155952
novel_sir520	AAAATATTTGGCGCCGTTAACTAT	GGTTTTATAAACCGCGGCAATTGA	tag213675
novel_sir521	GTTGGCCCTGATTTCTAGCATTGA	ACCGGGACTAAAGATCGTAACTTT	tag7101
novel_sir522	GCTGTGCCAGAGATTTTGTATTGA	ACACGGTCTCTAAAACATAACTTT	tag31070
novel_sir523	GTTGTGCCAGAGATTTTGTATTGA	ACACGGTCTCTAAAACATAACTTT	tag147656
novel_sir524	ACTGTGCCAGAGATTTTGTATTGA	ACACGGTCTCTAAAACATAACTTT	tag230228
novel_sir525	ACACGGTCTCCAAAACATAACTTT	ACTGTGCCAGAGGTTTTGTATTGA	tag16162
novel_sir526	ACACGGTCTCCAAAACATAACTTT	GCTGTGCCAGAGGTTTTGTATTGA	tag251262
novel_sir527	TCTCCAGCTGGTATTTTTTATTGA	AGGTCGACCATAAAAAATAACTCT	tag33928
novel_sir528	ACTTTTAATTTTGGCGTGAACTAA	TTTGAAAATTAAAACCGCACTTGA	tag243793
novel_sir529	ACTTTTAATTTTAGCGTGAACTAA	TTTGAAAATTAAAATCGCACTTGA	tag41125
novel_sir530	TTTGAAGATTAAAATCGCACTTGA	ACTTCTAATTTTAGCGTGAACTAA	tag231995
novel_sir531	TTTGAAGGTTAAAATCGCACTTGA	ACTTCCAATTTTAGCGTGAACTAA	tag139741
novel_sir532	TTTGAAAATTAAAACTGCACTTGA	ACTTTTAATTTTGACGTGAACTAA	tag134408
novel_sir533	CGTCATGGGCGAGGTACTTGA	AGTACCCGCTCCATGAACTTA	tag184583
novel_sir534	ACATCAACAATCGGAACTTA	TTTGTAGTTGTTAGCCTTGA	tag200187
novel_sir535	AACATCAACAATCGGAACTTA	TTTTGTAGTTGTTAGCCTTGA	tag250397
novel_sir536	TCTTTGTTGTTAGCCTTGA	AAACAACAATCGGAACTTA	tag21178
novel_sir537	ACTTTGTTGTTAGCCTTGA	AAACAACAATCGGAACTTA	tag234706
novel_sir538	AACCGTTTATTGCATGTCAACTAA	CGTTGGCAAATAACGTACAGTTGA	tag232571
novel_sir539	ACTTTCAATTTTAGCGTCAACTAA	GTTGAAAGTTAAAATCGCAGTTGA	tag138590
novel_sir540	TCTGTGGAGTCGCGGTGCAGTTGA	ACACCTCAGCGCCACGTCAACTGG	tag276570
novel_sir541	TCTTCCGCTCCGTACTCACGTTGA	AAGGCGAGGCATGAGTGCAACTGT	tag202230
novel_sir542	AATTTACCTTTGTCGTTGA	AAATGGAAACAGCAACTTT	tag543
novel_sir543	ATTTTACCTTTGTCGTTGA	AAATGGAAACAGCAACTTT	tag47684

novel_sir544	CATTTACCTTTGTCGTTGA	AAATGGAAACAGCAACTTT	tag160285
novel_sir545	TCTTTACCTTTGTCGTTGA	AAATGGAAACAGCAACTTT	tag263027
novel_sir546	CTTTTACCTTTGTCGTTGA	AAATGGAAACAGCAACTTT	tag273819
novel_sir547	TTTTTACCTTTGTCGTTGA	AAATGGAAACAGCAACTTT	tag292235
novel_sir548	TATGAAAACGGTGTACGGTGTTGA	ACTTTTGCCACATGCCACAACTTA	tag45290
novel_sir549	ACATGGTCTCCAGAACACAACTTT	GCTGTACCAGAGGTCTTGTGTTGA	tag140128
novel_sir550	ACACGGTCTCCAAAACACAACTTT	GCTGTGCCAGAGGTTTTGTGTTGA	tag261026
novel_sir551	ACCAGACTGAGCCACAAACTAA	ATTGGTCTGACTCGGTGTTTGA	tag15153
novel_sir552	AACCGTGTCCAACTCTAAAACTAG	TATTGGCACAGGTTGAGATTTTGA	tag269001
novel_sir553	TATTGGCACAGGTTGAGATTTTGA	AACCGTGTCCAACTCTAAAACTTC	tag269001
novel_sir554	GTTGAAAAGGTAGTGTAGTTTTGA	ACTTTTCCATCACATCAAAACTTT	tag276804
novel_sir555	ACCAACCGATCCGGCCAAAACTGG	CCTGGTTGGCTAGGCCGGTTTTGA	tag267679
novel_sir556	AGGCGCCACGTCAGCCAAAACTGC	CATCCGCGGTGCAGTCGGTTTTGA	tag116085
novel_sir557	TTTGCCAGGTCTAAGTGGTTTTGA	ACGGTCCAGATTCACCAAAACTTG	tag186843
novel_sir558	CCTGGTTGGCTAGGCGGTTTTTGA	ACCAACCGATCCGCCAAAAACTGG	tag271757
novel_sir559	AGAATCAGCCGAGACATTTTATAG	AATCTTAGTCGGCTCTGTAAAATA	tag106613
novel_sir560	GCTCGAACTGGCACGTATCAAATA	AGCTTGACCGTGCATAGTTTATAT	tag98679
novel_sir561	AGGACCAGACGAGACACTTTATAG	GATCCTGGTCTGCTCTGTGAAATA	tag234675
novel_sir562	TTTGAAAACCAGAGATTTGAAATA	ACTTTTGGTCTCTAAACTTTATAT	tag1870
novel_sir563	AGAGAGCGAGCTATAGATTTATAG	CTTCTCTCGCTCGATATCTAAATA	tag199257
novel_sir564	AGAAAGCGGGCTACAGATTTATAG	CCTCTTTCGCCCGATGTCTAAATA	tag62951
novel_sir565	AAATAGCGGGCTACAAATTTATAG	TTTTTATCGCCCGATGTTTAAATA	tag878
novel_sir566	GTTTTAAAACCGTCCTAAAGAATA	AAATTTTGGCAGGATTTCTTATGT	tag207071
novel_sir567	GTTTTAAAACCGTCCTAAAGAATA	AAATTTTGGCAGGATTTCTTATAT	tag207071
novel_sir568	AACGTTTGACCATTCGTCTTATTC	AGTTGCAAACTGGTAAGCAGAATA	tag85517
novel_sir569	AACGTTTGACCATTCGTCTTATTC	GGTTGCAAACTGGTAAGCAGAATA	tag130979
novel_sir570	ACTGTTTGACCACTCGTCTTATTC	CGTGACAAACTGGTGAGCAGAATA	tag33985
novel_sir571	ACTGTTTGACCACTCGTCTTATTC	CATGACAAACTGGTGAGCAGAATA	tag75032
novel_sir572	ACTGTTTGACCACTCGTCTTATTC	TGTGACAAACTGGTGAGCAGAATA	tag149649
novel_sir573	ACTGTTTGACCACTCGTCTTATTC	CTTGACAAACTGGTGAGCAGAATA	tag155699
novel_sir574	CATGACAAATTGGTGAGCAGAATA	ACTGTTTAACCACTCGTCTTATTC	tag135320
novel_sir575	CGTGACAAATTGGTGAGCAGAATA	ACTGTTTAACCACTCGTCTTATTC	tag260795
novel_sir576	TCTTGGTAAACCGATCAGAGAATA	AACCATTTGGCTAGTCTCTTATAG	tag117881
novel_sir577	TCTTCCTCTTCTGCAAACCGAATA	AAGGAGAAGACGTTTGGCTTATAC	tag183285

novel_sir578	ACAAAACAGTTAATAGCCTTATCC	TTTGTTTTGTCAATTATCGGAATA	tag192417
novel_sir579	AATCCGAATCTGAAAAGCTGAATA	AGGCTTAGACTTTTCGACTTATAA	tag168837
novel_sir580	CGTTGTGCCTGAGGTTCTATAATA	AACACGGACTCCAAGATATTATGT	tag16701
novel_sir581	TGTTGTGCCTGAGGTTCTGTAATA	AACACGGACTCCAAGACATTATGT	tag63592
novel_sir582	CGTTGTGCCTGAGGTTCTGTAATA	AACACGGACTCCAAGACATTATGT	tag87432
novel_sir583	AGCACGGACTCCAAAACATTATGT	CGTCGTGCCTGAGGTTTTGTAATA	tag293192
novel_sir584	TGTGATGCTAACATGATATTAATA	ACTACGATTGTACTATAATTATAT	tag208962
novel_sir585	AGTATTTGTCTGCCTCTGAACATA	ATAAACAGACGGAGACTTGTATGG	tag212918
novel_sir586	ACGAAAAAAGTTGGAAGTGTATGT	ATTGCTTTTTTCAACCTTCACATA	tag206727
novel_sir587	AGTCGTTCTATAACCCATA	AGCAAGATATTGGGTATTC	tag253459
novel_sir588	AGGCTTTAAACTCAGATCGTATAG	ACTCCGAAATTTGAGTCTAGCATA	tag130160
novel_sir589	CATCAAAACCAGATGCACCGCATA	AGTTTTGGTCTACGTGGCGTATGC	tag134115
novel_sir590	ACAATGCCCCGTCGCGTATTT	AATGTTACGGGGCAGCGCATA	tag170027
novel_sir591	CCTTGTATTGGATCATGTTGCATA	AACATAACCTAGTACAACGTATCT	tag178266
novel_sir592	ACATGTCCTAGTCACAACGTATCT	ACTGTACAGGATCAGTGTTGCATA	tag42624
novel_sir593	GATTTGCTGAAGGAAATACTCATA	AAACGACTTCCTTTATGAGTATGG	tag5490
novel_sir594	GATTTGCTGAAGGAAATGCTCATA	AAACGACTTCCTTTACGAGTATGG	tag6567
novel_sir595	AATCGGACGCTGACATCAGTATGA	TTTTAGCCTGCGACTGTAGTCATA	tag57834
novel_sir596	TCTTGTATCGCGTGTGTCATA	AACATAGCGCACACAGTATAG	tag289151
novel_sir597	AGACGATCTGGGTTCGAAGTATCA	GATCTGCTAGACCCAAGCTTCATA	tag189165
novel_sir598	TCTCGAGAAAGAACTAAGATA	AGCTCTTTCTTGATTCTATGG	tag177279
novel_sir599	ATAACTCAGCGCCAAGGTCTATGG	AATATTGAGTCGCGGTTCCAGATA	tag178115
novel_sir600	GGTCCCGGTCTCGCCTGAACGATA	AGGGCCAGAGCGGACTTGCTATCC	tag85388
novel_sir601	CTTTACAAGAGAGGCGTCCGGATA	AATGTTCTCTCCGCAGGCCTATCG	tag194384
novel_sir602	AACGACTTTCGGCAAAACCTATAG	GGTTGCTGAAAGCCGTTTTGGATA	tag98093
novel_sir603	TATCTGTGATGAATACCTGTGATA	AGACACTACTTATGGACACTATGG	tag199385
novel_sir604	TGTTTTTAACATTCGCCCTTGATA	AAAAATTGTAAGCGGGAACTATAC	tag222470
novel_sir605	CTTCCACTGAGCCTGCCGTTGATA	AGGTGACTCGGACGGCAACTATAC	tag23617
novel_sir606	CATGTTGCTTAGACCTGTTTGATA	ACAACGAATCTGGACAAACTATAG	tag127449
novel_sir607	GTTGAAATTGACAGGCAGAATATA	ACTTTAACTGTCCGTCTTATATAA	tag24448
novel_sir608	GCTCCCGATGTTCAAACTATA	AGGGCTACAAGTTTGATATAA	tag217676
novel_sir609	ACTGCGCCAGTTCGACGGATATCC	CTTGACGCGGTCAAGCTGCCTATA	tag153407
novel_sir610	ACGTAAATTGGCTATAGGATATCG	TTTGCATTTAACCGATATCCTATA	tag234559
novel_sir611	ACTCCCGGACACACGTATCCTATA	AGGGCCTGTGTGCATAGGATATGC	tag93002

novel_sir612	AGGGCCTGTGTGCATAGGATATGT	ACTCCCGGACACACGTATCCTATA	tag93002
novel_sir613	CATCATGTATGCACCAGAGCTATA	AGTACATACGTGGTCTCGATATCT	tag170270
novel_sir614	AGGACAAGTGGTCAAACGATATAA	TATCCTGTTCACCAGTTTGCTATA	tag288350
novel_sir615	AAGAGCTGCGGGCTGTAGATATAT	TCTTCTCGACGCCCGACATCTATA	tag98104
novel_sir616	ACTTTAGGCCCGTTTGAGATATGG	GTTGAAATCCGGGCAAACTCTATA	tag232446
novel_sir617	GTTGAACTCCGGGCAAACTCTATA	ACTTGAGGCCCGTTTGAGATATGG	tag93960
novel_sir618	TCTTCTCATCGCCCGATGTCTATA	AAGAGTAGCGGGCTACAGATATGT	tag124181
novel_sir619	TCTTCTGATCGTCCGATGTCTATA	AAGACTAGCAGGCTACAGATATGT	tag40335
novel_sir620	TCTTCTCGTCGCCTGATGTCTATA	AAGAGCAGCGGACTACAGATATGT	tag8418
novel_sir621	AGAGTTCGTATGACCAAGATATGA	AGTCTCAAGCATACTGGTTCTATA	tag194753
novel_sir622	ACAAGAAATTAGATCGCCATATCC	TTTGTTCTTTAATCTAGCGGTATA	tag166489
novel_sir623	AATTTTTGTACTCGCTTAATATAA	TGTTAAAAACATGAGCGAATTATA	tag242733
novel_sir624	CCTGAAATCCGGCAAGGCATTATA	ACTTTAGGCCGTTCCGTAATATAC	tag107954
novel_sir625	AGTGGACTCCAAGACGCAATATGT	CGTCACCTGAGGTTCTGCGTTATA	tag193933
novel_sir626	TCTCATACTGCGCACCTGGTTATA	AGTATGACGCGTGGACCAATATGC	tag228458
novel_sir627	AGTTTGGATTGAGACTAAATATTT	AATCAAACCTAACTCTGATTTATA	tag9517
novel_sir628	ATTTTTCAGTTGCCACAGTTTATA	AAAAGTCAACGGTGTCAAATATTT	tag87546
novel_sir629	TTTTTTCAGTTGCCACAGTTTATA	AAAAGTCAACGGTGTCAAATATTT	tag107611
novel_sir630	TTTTTTCAGTTACCGCAGTTTATA	AAAAGTCAATGGCGTCAAATATTT	tag47176
novel_sir631	AAAAGTCAACGGCGTCAAATATTT	TGTTTTCAGTTGCCGCAGTTTATA	tag61709
novel_sir632	AAAAGTCAACGGCGTCAAATATTT	ATTTTTCAGTTGCCGCAGTTTATA	tag249608
novel_sir633	ACCGGGACTAAAGATCAAATATGC	GTTGGCCCTGATTTCTAGTTTATA	tag259737
novel_sir634	GTTGCCACAGTATGTAATTTTATA	ACGGTGTCATACATTAAAATATGG	tag239250
novel_sir635	ACTCAAAAGCGCAGGTTGAAACTA	AGTTTTCGCGTCCAACTTTGATCG	tag64912
novel_sir636	AATTAGTGGCCAAACATTTGATGT	TATTAATCACCGGTTTGTAAACTA	tag61713
novel_sir637	TGTATAAGTCTAAGTCGTCAACTA	ATATTCAGATTCAGCAGTTGATTG	tag194202
novel_sir638	CCTTTCAAAACCTACACTACACTA	AAAGTTTTGGATGTGATGTGATGT	tag87700
novel_sir639	GGTGTTTTTGCACTGTGCCGACTA	ACAAAAACGTGACACGGCTGATGG	tag291824
novel_sir640	CTTCTTTAAGCGTCAGGACTA	AGAAATTCGCAGTCCTGATGG	tag150089
novel_sir641	CCTGAAGGTCGATTCACCGGACTA	ACTTCCAGCTAAGTGGCCTGATGT	tag90425
novel_sir642	ACGGCGCCTGCGAACATATGATTT	TTTGCCGCGGACGCTTGTATACTA	tag173054
novel_sir643	ACACTAGTGGCCGCCGGATGATGG	ACTGTGATCACCGGCGGCCTACTA	tag29479
novel_sir644	AATTGGGTTCCGGATGCTACTA	AACCCAAGGCCTACGATGATTC	tag144272
novel_sir645	CTTTGCATTATGGGTTCAGTACTA	AACGTAATACCCAAGTCATGATCT	tag140099

novel_sir646	AAGCGGCGCCTGCGAAAATGATTT	AATTCGCCGCGGACGCTTTTACTA	tag181774
novel_sir647	TATCTTTTACTGACTTCAAACCTA	AGAAAATGACTGAAGTTTGGATAC	tag182044
novel_sir648	AGCTTGGAAACTACTGTTGGATCG	CTTCGAACCTTTGATGACAACCTA	tag106704
novel_sir649	AGGCTATATACTCGTTGGATAC	GTTCCGATATATGAGCAACCTA	tag135089
novel_sir650	ACGGCTAAGTCCCTAGTTGGATAT	ATTGCCGATTCAGGGATCAACCTA	tag228068
novel_sir651	GGTGAAAATCGATACTTAGACCTA	ACTTTTAGCTATGAATCTGGATAC	tag156449
novel_sir652	GTTGAAAATCGATACTTAGACCTA	ACTTTTAGCTATGAATCTGGATAC	tag157812
novel_sir653	GCTCTTACCGGGAGGCGACCTA	AGAATGGCCCTCCGCTGGATGG	tag59268
novel_sir654	ACGCCGACAGGAGCTGGATCC	GCTGCGGCTGTCCTCGACCTA	tag22625
novel_sir655	GTTGCCGCAGTTTGTAAAACCCTA	ACGGCGTCAAACATTTTGGGATGG	tag141257
novel_sir656	AGGACGAAAGTGTAAGCGGGATAC	GCTCCTGCTTTCACATTCGCCCTA	tag163504
novel_sir657	TCTTAGAAAGTCGGGTGCAGCCTA	AATCTTTCAGCCCACGTCGGATAG	tag7613
novel_sir658	CCTTAGAAAGTCGGGTGCAGCCTA	AATCTTTCAGCCCACGTCGGATAG	tag185449
novel_sir659	AGGCTGACATGTAGACTCGGATAG	CTTCCGACTGTACATCTGAGCCTA	tag147206
novel_sir660	AACAGGACTTATCATATCGGATGT	CTTTGTCCTGAATAGTATAGCCTA	tag24556
novel_sir661	AGTTTGGCGTATCATATCGGATAT	TTTCAAACCGCATAGTATAGCCTA	tag275372
novel_sir662	TGTTTGTGTTGCTACATTAGCCTA	AAACACAACGATGTAATCGGATCG	tag209327
novel_sir663	GCTTCGATGGCACACGGCCTA	AAGCTACCGTGTGCCGGATTA	tag180838
novel_sir664	ACGCCAAGACAAGCCGCCGGATAT	TATGCGGTTCTGTTCGGCGGCCTA	tag154732
novel_sir665	ACGTTTGACTGTCCGACCGGATGT	CCTGCAAACTGACAGGCTGGCCTA	tag118224
novel_sir666	ATCAATCTACGACTATACGGATTT	TATAGTTAGATGCTGATATGCCTA	tag186429
novel_sir667	TGTCCGCGAAGCGACAGCTGCCTA	AGGCGCTTCGCTGTCGACGGATAC	tag133827
novel_sir668	TGTCCGCGGAGCGACAGCTGCCTA	AGGCGCCTCGCTGTCGACGGATAC	tag174450
novel_sir669	TGTCTGCGGAGCGACAGCTGCCTA	AGACGCCTCGCTGTCGACGGATAC	tag62073
novel_sir670	AGACGCCTCACTGTCGACGGATAC	TGTCTGCGGAGTGACAGCTGCCTA	tag274807
novel_sir671	ACGGCGTCGAATATTTTAGGATGA	GTTGCCGCAGCTTATAAAATCCTA	tag9839
novel_sir672	GTTGCCGCAGTTTATAAAATCCTA	ACGGCGTCAAATATTTTAGGATGG	tag120682
novel_sir673	TGTTGCTTAGACCTATCAATCCTA	AACGAATCTGGATAGTTAGGATGT	tag149584
novel_sir674	ACTCGTCTAAGTATCAGGATCCTA	AGCAGATTCATAGTCCTAGGATGG	tag190428
novel_sir675	AAACGTAAATTGGCTATAGGATAT	TTTTTGCATTTAACCGATATCCTA	tag133338
novel_sir676	TGTTGCTTAGATCTGTATATCCTA	AACGAATCTAGACATATAGGATGT	tag80261
novel_sir677	CCTCCTTATCCTTTTTGTATCCTA	AGGAATAGGAAAAACATAGGATTC	tag182867
novel_sir678	TCTCCTTATCCTTTTTGTATCCTA	AGGAATAGGAAAAACATAGGATTC	tag260115
novel_sir679	AACGAAACCATGTTTTGAGGATGG	GGTTGCTTTGGTACAAAACTCCTA	tag179405

novel_sir680	CGTCGAACTCTAGCAAAACTCCTA	AGCTTGAGATCGTTTTGAGGATGT	tag232456
novel_sir681	ACATACTTCGACTGCAGAGGATCC	GGTGTATGAAGCTGACGTCTCCTA	tag264889
novel_sir682	AGATGGATAGCAAACCAAGGATCC	TGTCTACCTATCGTTTGGTTCCTA	tag71661
novel_sir683	ACTCCGAAGGTCTAAAGGATCG	TTTGAGGCTTCCAGATTTCCTA	tag76774
novel_sir684	ACTGTGAATGGCCTAAAAGGATAT	CGTGACACTTACCGGATTTTCCTA	tag7830
novel_sir685	AGCCGCGCCGCGCCGTCGATTC	GGTCGGCGCGGCGCGGCAGCTA	tag123283
novel_sir686	GCTTCCGTAGCAGCATCAGCTA	AAGGCATCGTCGTAGTCGATCC	tag204817
novel_sir687	ACGGAACCCTAACGGCGATGG	CTTGCCTTGGGATTGCCGCTA	tag70393
novel_sir688	GATGACTACTGGCACGGCGCTA	ACTGATGACCGTGCCGCGATAG	tag235850
novel_sir689	CCTTCGGGGCTCGCGGCGCTA	AAGCCCCGAGCGCCGCGATGG	tag76709
novel_sir690	ACCCGGGACTAAAGATAGCGATCT	AGTGGGCCCTGATTTCTATCGCTA	tag147109
novel_sir691	ACGTCCTAGACTCGAAAGCGATCT	TGTGCAGGATCTGAGCTTTCGCTA	tag236275
novel_sir692	TATTAATTAAAAGTTTGTAGGCTA	AATTAATTTTCAAACATCCGATGT	tag73570
novel_sir693	AATTAGTGTCCAAACATCCGATGT	TATTAATCACAGGTTTGTAGGCTA	tag153499
novel_sir694	TGTGGATGCCTAGTCTGCGGGCTA	ACCTACGGATCAGACGCCCGATCC	tag210530
novel_sir695	CGTCTAACGCGCGGATCCTGGCTA	AGATTGCGCGCCTAGGACCGATAT	tag220886
novel_sir696	TATTGTTGAAAATCGGAAAATCTA	AACAACTTTTAGCCTTTTAGATTT	tag175880
novel_sir697	GTTCGGTCGGTAATTGGTAATCTA	AGCCAGCCATTAACCATTAGATGT	tag129235
novel_sir698	GATCAAACTGATCGGTTTAATCTA	AGTTTGACTAGCCAAATTAGATGG	tag203397
novel_sir699	GATCCTCACTGAATATAACATCTA	AGGAGTGACTTATATTGTAGATGC	tag233269
novel_sir700	AGGCACAACGTTCGATGTAGATGC	CTTCCGTGTTGCAAGCTACATCTA	tag152808
novel_sir701	ACCGGCACAAAATAGAGTAGATGC	TTTGGCCGTGTTTTATCTCATCTA	tag174050
novel_sir702	AGAAAAACATCGGGCTATAGATTT	TCTCTTTTTGTAGCCCGATATCTA	tag274385
novel_sir703	AGCCGACACTGATACTATAGATGC	TTTCGGCTGTGACTATGATATCTA	tag223211
novel_sir704	CCTCTTTTCGTCGCCTGATATCTA	AGAAAAGCAGCGGACTATAGATCT	tag92813
novel_sir705	ACTAGTGACGGGCCATATAGATCC	AGTGATCACTGCCCGGTATATCTA	tag245345
novel_sir706	GATGACCTGGGTGTATATTATCTA	ACTGGACCCACATATAATAGATAC	tag256229
novel_sir707	GATATCAACTCCGGGCTAACTCTA	ATAGTTGAGGCCCGATTGAGATGA	tag29718
novel_sir708	TGTTTTCATGTGCCTACCTCTA	AAAAGTACACGGATGGAGATGG	tag18256
novel_sir709	AGAAATGGACGTCCGAGATTC	GCTCTTTACCTGCAGGCTCTA	tag278576
novel_sir710	TGTGAAATACTAGAAATCAGTCTA	ACTTTATGATCTTTAGTCAGATTG	tag146034
novel_sir711	AGCGGCCTTGGACTAATCAGATCT	CGTCGCCGGAACCTGATTAGTCTA	tag147123
novel_sir712	AAGCATAGTGGCCCGCGCAGATTG	TGTTCGTATCACCGGGCGCGTCTA	tag193193
novel_sir713	ACCGCTCTCATATAGTACAGATCC	CCTGGCGAGAGTATATCATGTCTA	tag254365

novel_sir714	CGTCGCCCGATGTCTAGATGTCTA	AGCGGGCTACAGATCTACAGATAT	tag261388
novel_sir715	TCTCTTTTCGTCGCCCGATGTCTA	AGAAAAGCAGCGGGCTACAGATCT	tag60782
novel_sir716	TGTCTTTTCGTCGCCCGATGTCTA	AGAAAAGCAGCGGGCTACAGATCT	tag60927
novel_sir717	TCTCTTTTTGTCGCCTGATGTCTA	AGAAAAACAGCGGACTACAGATTT	tag215724
novel_sir718	AGCGGGCTATACAGATACAGATGT	CGTCGCCCGATATGTCTATGTCTA	tag40552
novel_sir719	GATCGACGCGTACTTGAGATTCTA	AGCTGCGCATGAACTCTAAGATGT	tag160991
novel_sir720	AATCGGTCCAGTCTTCGAAGATGC	AGTTAGCCAGGTCAGAAGCTTCTA	tag30136
novel_sir721	ACTCTGATACGGTTTCCAAGATGC	CCTGAGACTATGCCAAAGGTTCTA	tag99182
novel_sir722	CCTGACATCGTGCCTGAGGTTCTA	ACTGTAGCACGGACTCCAAGATGC	tag7345
novel_sir723	TGTTGGCTGTGTATATCATTTCTA	AACCGACACATATAGTAAAGATGT	tag69562
novel_sir724	AACACCAACCGGGACTAAAGATGT	AATTGTGGTTGGCCCTGATTTCTA	tag95054
novel_sir725	AACACCAACCGGGACTAAAGATGT	CATTGTGGTTGGCCCTGATTTCTA	tag177989
novel_sir726	AACACCAACCGGGACTAAAGATCT	AATTGTGGTTGGCCCTGATTTCTA	tag95054
novel_sir727	AACACCAACCGGGACTAAAGATCT	CATTGTGGTTGGCCCTGATTTCTA	tag177989
novel_sir728	AACACCAACCGGGACTAAAGATGG	AATTGTGGTTGGCCCTGATTTCTA	tag95054
novel_sir729	AACACCAACCGGGACTAAAGATGG	CATTGTGGTTGGCCCTGATTTCTA	tag177989
novel_sir730	AATTGTGGTTGGCCCTGATTTCTA	AACACCAACCGGGACTAAAGATCG	tag95054
novel_sir731	CATTGTGGTTGGCCCTGATTTCTA	AACACCAACCGGGACTAAAGATCG	tag177989
novel_sir732	AACACCAACCGGGACTAAAGATCC	AATTGTGGTTGGCCCTGATTTCTA	tag95054
novel_sir733	AACACCAACCGGGACTAAAGATCC	CATTGTGGTTGGCCCTGATTTCTA	tag177989
novel_sir734	TATCGACCGAAATTCACTTTTCTA	AGCTGGCTTTAAGTGAAAAGATTG	tag220361
novel_sir735	AGTGGGCGCTAGCTGAAAAGATAG	AGTCACCCGCGATCGACTTTTCTA	tag38029
novel_sir736	GTTGCTTAGACGGTATTTTTTCTA	ACGAATCTGCCATAAAAAAGATAG	tag166761
novel_sir737	GGTCGAAGACCGAAGAATCAAGTA	AGCTTCTGGCTTCTTAGTTCATTT	tag218441
novel_sir738	GTTATTATGTGAGCCGCCTAAGTA	ATAATACACTCGGCGGATTCATCC	tag177061
novel_sir739	GATTACAAATCGGATGGTTAAGTA	AATGTTTAGCCTACCAATTCATGA	tag53505
novel_sir740	TTTCTTTAGGAATTGGAAACAGTA	AGAAATCCTTAACCTTTGTCATAA	tag27497
novel_sir741	AGAAATCCTTAAACTTTGTCATAA	TTTCTTTAGGAATTTGAAACAGTA	tag240998
novel_sir742	TGTCTTGAACAGTATCACACAGTA	AGAACTTGTCATAGTGTGTCATCT	tag119647
novel_sir743	GGTCTTGAACAGTATCACACAGTA	AGAACTTGTCATAGTGTGTCATCT	tag155658
novel_sir744	GTTTGAACAAGCTGACACAGTA	AACTTGTTCGACTGTGTCATAC	tag98815
novel_sir745	AGTATTGCACAGATAGCCACAGTA	ATAACGTGTCTATCGGTGTCATAG	tag11995
novel_sir746	ATCCGACATGCTTTGGCGTCATTC	CGTAGGCTGTACGAAACCGCAGTA	tag194602
novel_sir747	TCTTTTTCAGTTGCCGCAGTA	AAAAAGTCAACGGCGTCATCT	tag89882

novel_sir748	TGTTTTTCAGTTGCCGCAGTA	AAAAAGTCAACGGCGTCATCT	tag185336
novel_sir749	TATTTTTCAGTTGCCGCAGTA	AAAAAGTCAACGGCGTCATCT	tag201692
novel_sir750	AGTTTTTCAGTTGCCGCAGTA	AAAAAGTCAACGGCGTCATCT	tag246851
novel_sir751	AATTTTTCAGTTGCCGCAGTA	AAAAAGTCAACGGCGTCATCT	tag258046
novel_sir752	ATTTTTTCAGTTGCCGCAGTA	AAAAAGTCAACGGCGTCATCT	tag259967
novel_sir753	AGAAAAAGTCAACGGCGTCATCT	ACTCTTTTTCAGTTGCCGCAGTA	tag85992
novel_sir754	CATTCTTTTTCAGTTGCCGCAGTA	AAGAAAAAGTCAACGGCGTCATCT	tag47748
novel_sir755	AGCACATTGGCCCACGCGTCATAC	AATCGTGTAACCGGGTGCGCAGTA	tag66780
novel_sir756	AGCACATTGGCCCACGCGTCATAC	GATCGTGTAACCGGGTGCGCAGTA	tag238253
novel_sir757	ACTTAGAGGCGTTCAGTCATAA	ACTGAATCTCCGCAAGTCAGTA	tag25660
novel_sir758	ATCTTGAGATCGACGAAGTCATCA	TATAGAACTCTAGCTGCTTCAGTA	tag106352
novel_sir759	ACAAAACAGTTAATAGTCTCATCC	TTTGTTTTGTCAATTATCAGAGTA	tag82794
novel_sir760	TTTGTTTTGTCAATTATCGGAGTA	ACAAAACAGTTAATAGCCTCATCC	tag194666
novel_sir761	ACAAAACACTTAATAGCCTCATCC	TTTGTTTTGTGAATTATCGGAGTA	tag281251
novel_sir762	AGGCCCGTCACAGATGACTCATCA	GATCCGGGCAGTGTCTACTGAGTA	tag43912
novel_sir763	CATTAGACGCGCCCGGTGATAGTA	AATCTGCGCGGGCCACTATCATAG	tag196086
novel_sir764	AATGGTTGGCCCTGATTTCTAGTA	ACCAACCGGGACTAAAGATCATCT	tag85406
novel_sir765	TGTGGTTGGCCCTGATTTCTAGTA	ACCAACCGGGACTAAAGATCATCT	tag255181
novel_sir766	ATTTAGGTGACCGAATATTTAGTA	AATCCACTGGCTTATAAATCATAT	tag48990
novel_sir767	ATTTAGGTGGCCGAATATTTAGTA	AATCCACCGGCTTATAAATCATAT	tag217391
novel_sir768	AGTTTTAACACGTGCCTTTTAGTA	AAAATTGTGCACGGAAAATCATGG	tag216312
novel_sir769	GATGCTGTAGCTCCTGACGTA	ACGACATCGAGGACTGCATCG	tag281110
novel_sir770	ACTAGTTGGTGTTTAGATGCATGT	CATGATCAACCACAAATCTACGTA	tag213586
novel_sir771	CCTACTTAGGCTTGGCAAACCGTA	ATGAATCCGAACCGTTTGGCATAA	tag229920
novel_sir772	GATCCAGAAGCAGGGTGCACCGTA	AGGTCTTCGTCCCACGTGGCATGG	tag184227
novel_sir773	AGGTCTCCGTCCCACGTGGCATTG	GATCCAGAGGCAGGGTGCACCGTA	tag238983
novel_sir774	GATCCAGAAGCAGGTTGCACCGTA	AGGTCTTCGTCCAACGTGGCATTG	tag13142
novel_sir775	GCTGAATTTATGCGCTGCCCCGTA	ACTTAAATACGCGACGGGGCATTG	tag255565
novel_sir776	AGTAGTTGCCGTCGGTGCCGTA	ATCAACGGCAGCCACGGCATGG	tag251521
novel_sir777	ACGAGGTGAGCCATTAGCGCATGA	TTTGCTCCACTCGGTAATCGCGTA	tag120017
novel_sir778	ATATAAGTCATTCTAGCATTT	GTTATATTCAGTAAGATCGTA	tag120290
novel_sir779	TTTAGGACTGGCAACTAGATCGTA	ATCCTGACCGTTGATCTAGCATCA	tag44260
novel_sir780	CGTCAAGCACCGCGGCTGATCGTA	AGTTCGTGGCGCCGACTAGCATGG	tag106584
novel_sir781	GCTCCTCGTCGGCGCCTCGTA	AGGAGCAGCCGCGGAGCATGA	tag181625

novel_sir782	GATCTGCTAGACTCAAGCTTCGTA	AGACGATCTGAGTTCGAAGCATCA	tag123700
novel_sir783	ACTCCAGAAGCACTCCAGAAGGTA	AGGTCTTCGTGAGGTCTTCCATGA	tag71235
novel_sir784	AATAGTTCGAGTGGACGTCCATCC	GGTTATCAAGCTCACCTGCAGGTA	tag286876
novel_sir785	TGTATGTGACACAGAACTCAGGTA	ATACACTGTGTCTTGAGTCCATGC	tag148950
novel_sir786	ATCTATATGGCCAATTCTCCATCT	TGTAGATATACCGGTTAAGAGGTA	tag220216
novel_sir787	ACTAGCGGGCCTACGGCTCCATCT	CCTGATCGCCCGGATGCCGAGGTA	tag244788
novel_sir788	GTTCGGATTCGGTGTGCTACGGTA	AGCCTAAGCCACACGATGCCATAT	tag210021
novel_sir789	CTTCTCGAGCGGACGCCGGTA	AGAGCTCGCCTGCGGCCATGG	tag265921
novel_sir790	ATAAGATCTTATGACTAGCCATAT	GGTATTCTAGAATACTGATCGGTA	tag267452
novel_sir791	GGTATTCTGGAATACTGATCGGTA	ATAAGACCTTATGACTAGCCATAT	tag281728
novel_sir792	AACTTTTTGTCACTGTACCCATAT	AATTGAAAAACAGTGACATGGGTA	tag40465
novel_sir793	ACTATAAAGATATTAGACCCATAT	AATGATATTTCTATAATCTGGGTA	tag261624
novel_sir794	TCTTAAAGGGCCAGCCAGTGGGTA	AATTTCCCGGTCGGTCACCCATCC	tag226
novel_sir795	AAACTACACTCTACAACCCATGG	TCTTTGATGTGAGATGTTGGGTA	tag291082
novel_sir796	AGCCTATCAGACTTGGTACCATAG	CCTCGGATAGTCTGAACCATGGTA	tag276146
novel_sir797	GATCTAACAAAGGATCTTCTGGTA	AGATTGTTTCCTAGAAGACCATGC	tag20396
novel_sir798	TCTCTCTGGCGATTATTTCTGGTA	AGAGACCGCTAATAAAGACCATTG	tag196112
novel_sir799	CGTTAGACGCGCCCGGTGGTA	AATCTGCGCGGGCCACCATGC	tag210535
novel_sir800	CGTTAATGGCTAAACGGTGTGGTA	AATTACCGATTTGCCACACCATGG	tag119736
novel_sir801	AAAGTTAGACGCGGAAAACCATGG	AGTTTCAATCTGCGCCTTTTGGTA	tag86537
novel_sir802	AAAGTTAAACGCGGAAAACCATAG	AGTTTCAATTTGCGCCTTTTGGTA	tag32669
novel_sir803	ATGCTAGAAAGTCTTACATTG	GTTACGATCTTTCAGAATGTA	tag284290
novel_sir804	TTTACGATCTTACTGAATGTA	ATGCTAGAATGACTTACATTG	tag136101
novel_sir805	ATACTAGAATGACTTACATTG	TTTATGATCTTACTGAATGTA	tag169071
novel_sir806	GCTCAGATAACGACTCTACATGTA	AGTCTATTGCTGAGATGTACATGT	tag167724
novel_sir807	AACCACAGTTCGACAGGTACATGT	TGTTGGTGTCAAGCTGTCCATGTA	tag179471
novel_sir808	GGTCGACGCTCCGGCTCATGTA	AGCTGCGAGGCCGAGTACATGG	tag282209
novel_sir809	AAGGTCCTATAGCAGTATACATGG	TGTTCCAGGATATCGTCATATGTA	tag154193
novel_sir810	AGTTTTCAGTTGCCGCAGTATGTA	AAAAGTCAACGGCGTCATACATAC	tag101389
novel_sir811	ATTTTTCAGTTGCCGCAGTATGTA	AAAAGTCAACGGCGTCATACATAC	tag120604
novel_sir812	GTTTTTCAGTTGCCGCAGTATGTA	AAAAGTCAACGGCGTCATACATAC	tag128197
novel_sir813	GCTTTTCAGTTGCCGCAGTATGTA	AAAAGTCAACGGCGTCATACATAC	tag210666
novel_sir814	AAAAGTCAACGGCGTCATACATTA	ATTTTTCAGTTGCCGCAGTATGTA	tag120604
novel_sir815	AAAAGTCAACGGCGTCATACATTA	GTTTTTCAGTTGCCGCAGTATGTA	tag128197

novel_sir816	ACAAGGGCTCGGTTGACATGC	TATGTTCCCGAGCCAACTGTA	tag182555
novel_sir817	AGGAAGGCTGCGCCGTTGACATGC	TGTCCTTCCGACGCGGCAACTGTA	tag120213
novel_sir818	GGTGATTGAGGAATTCGAACTGTA	ACTAACTCCTTAAGCTTGACATGT	tag220350
novel_sir819	CCTGGACTGATTTACCGCACTGTA	ACCTGACTAAATGGCGTGACATGT	tag14190
novel_sir820	GATCCAGAGGCAGGGTGCACTGTA	AGGTCTCCGTCCCACGTGACATTG	tag131310
novel_sir821	TCTGCCTGTCAGTTTCAACCTGTA	ACGGACAGTCAAAGTTGGACATGG	tag125629
novel_sir822	GGTCTATGCAACACTGATCCTGTA	AGATACGTTGTGACTAGGACATGT	tag194875
novel_sir823	ATGCGGCATGTGGAGACATGA	TCTACGCCGTACACCTCTGTA	tag230402
novel_sir824	AATCTAGGACCGTACGAGACATTT	CGTTAGATCCTGGCATGCTCTGTA	tag258972
novel_sir825	CGTTAGATCCTGGCCTGTTCTGTA	AATCTAGGACCGGACAAGACATTT	tag167101
novel_sir826	AATATCAGAATCCTATACAGTGTA	ATAGTCTTAGGATATGTCACATCC	tag270797
novel_sir827	TGTGTGTGTTGAAAAGTCAGTGTA	ACACACAACTTTTCAGTCACATCA	tag65834
novel_sir828	TTTTGAAAATTCAGGGATAGTGTA	AACTTTTAAGTCCCTATCACATCG	tag244813
novel_sir829	TTTTGAAAATTCAGGGATAGTGTA	AACTTTTAAGTCCCTATCACATCA	tag244813
novel_sir830	ACACTCAACTTTTTCATCACATCA	TTTGTGAGTTGAAAAAGTAGTGTA	tag256813
novel_sir831	AGTGCTTGCCTCGTTCGTGTA	ACGAACGGAGCAAGCACATCC	tag42054
novel_sir832	AAGACACGATTCCACACATGT	TGTTCTGTGCTAAGGTGTGTA	tag282264
novel_sir833	GGTGATTGAGGAATTCGAATTGTA	ACTAACTCCTTAAGCTTAACATGC	tag233771
novel_sir834	ATACCAAAACTTCGTGGAACATGT	CTTATGGTTTTGAAGCACCTTGTA	tag213366
novel_sir835	TGTTTTTTCGCTCGACCTTGTA	AAAAAAGCGAGCTGGAACATCT	tag48425
novel_sir836	AGCAGGCGCGACGTGGGAACATGG	CTTCGTCCGCGCTGCACCCTTGTA	tag36602
novel_sir837	AAAAGTCAACGGTGTCAAACATTT	ATTTTTCAGTTGCCACAGTTTGTA	tag441
novel_sir838	AAAAGTCAACGGTGTCAAACATTT	TTTTTTCAGTTGCCACAGTTTGTA	tag273282
novel_sir839	AAGACGAATGGTCAAACATGT	TATTCTGCTTACCAGTTTGTA	tag101696
novel_sir840	AAAAGTCAACGGCGTCAAACATTC	ATTTTTCAGTTGCCGCAGTTTGTA	tag112534
novel_sir841	AAAAGTCAACGGCGTCAAACATTT	ATTTTTCAGTTGCCGCAGTTTGTA	tag112534
novel_sir842	TTTCGCACGGTGCTTTTAAAATTA	AGCGTGCCACGAAAATTTTAATCT	tag141978
novel_sir843	AGTCTAACTCGGCTCAAATTA	AGATTGAGCCGAGTTTAATTA	tag43706
novel_sir844	GCTTTCTATGGCCAAGTGAAATTA	AAAGATACCGGTTCACTTTAATAG	tag220577
novel_sir845	AATTTCCATGCTCAACTTTAATTG	ATTTAAAGGTACGAGTTGAAATTA	tag102160
novel_sir846	TTTGGCCTTGGGAATCATAAATTA	ACCGGAACCCTTAGTATTTAATAC	tag77738
novel_sir847	TTTAGTCCGATATAAGTTAAATTA	ATCAGGCTATATTCAATTTAATAG	tag225641
novel_sir848	AGTCTGCAACCAGTTCTTCAATTA	AGACGTTGGTCAAGAAGTTAATGG	tag82880
novel_sir849	TTTCACACGGTGCTTTTAGAATTA	AGTGTGCCACGAAAATCTTAATTT	tag32092

novel_sir850	TTTCACACGGTGCTTTTAGAATTA	AGTGTGCCACGAAAATCTTAATCT	tag32092
novel_sir851	TTTCGCACGGTGCTTTTAGAATTA	AGCGTGCCACGAAAATCTTAATCT	tag482
novel_sir852	TTTCGCACGGTGCTTTTAGAATTA	AGCGTGCCACGAAAATCTTAATTT	tag482
novel_sir853	ATTGTAGGGCCGGATCCCGAATTA	ACATCCCGGCCTAGGGCTTAATAG	tag164998
novel_sir854	TGTTATGCCAGAGATTAGTAATTA	AATACGGTCTCTAATCATTAATGT	tag233297
novel_sir855	AGTAGAAGCCGGTAATTAATTA	ATCTTCGGCCATTAATTAATTG	tag260829
novel_sir856	TGTCGTGCTTGAGGCTCTACATTA	AGCACGAACTCCGAGATGTAATGT	tag28195
novel_sir857	AGCACGGACTCCAAGATGTAATGT	CGTCGTGCCTGAGGTTCTACATTA	tag76670
novel_sir858	AGCACGGACTCCAAGATGTAATGT	CATCGTGCCTGAGGTTCTACATTA	tag179826
novel_sir859	AACACGGACTCCAAGATGTAATGT	TGTTGTGCCTGAGGTTCTACATTA	tag100861
novel_sir860	AGCACGGACTCTAAGACGTAATGT	CGTCGTGCCTGAGATTCTGCATTA	tag70784
novel_sir861	AGCACGGACTCCAAGACGTAATGT	CGTCGTGCCTGAGGTTCTGCATTA	tag126395
novel_sir862	CGTTGTGCCTGAGGTTCTGCATTA	AACACGGACTCCAAGACGTAATGT	tag283584
novel_sir863	ACTGGCACGGCGCTATCATTA	ACCGTGCCGCGATAGTAATTC	tag195517
novel_sir864	TATGAATTAATTAGTGCACGATTA	ACTTAATTAATCACGTGCTAATGG	tag132306
novel_sir865	TTTTATACAAATATCGACCGATTA	AATATGTTTATAGCTGGCTAATAG	tag215797
novel_sir866	AATATATTTATAGCTGGCTAATAG	TTTTATATAAATATCGACCGATTA	tag112375
novel_sir867	CGTACAAACCTAGCTACCCGATTA	ATGTTTGGATCGATGGGCTAATTC	tag194711
novel_sir868	TATGAATTAATTAGTGCGCGATTA	ACTTAATTAATCACGCGCTAATGG	tag132557
novel_sir869	ACAATAAAGAGAGACCGCTAATAA	ACTGTTATTTCTCTCTGGCGATTA	tag192606
novel_sir870	GGTCCCGATTAAAACTCTCGATTA	AGGGCTAATTTTGAGAGCTAATGT	tag157433
novel_sir871	GGTGAGCAGCTGCGGTCTCGATTA	ACTCGTCGACGCCAGAGCTAATGC	tag72538
novel_sir872	ACTAAGACCAAGAAAAGCTAATAA	TGTGATTCTGGTTCTTTTCGATTA	tag63517
novel_sir873	TTTTTGAACCAAAGGCACGGATTA	AAACTTGGTTTCCGTGCCTAATAT	tag164089
novel_sir874	GCTCTACTTAGAAAACTCGGATTA	AGATGAATCTTTTGAGCCTAATTA	tag256511
novel_sir875	GCTCTGCTTAGAAAATTCGGATTA	AGACGAATCTTTTAAGCCTAATTG	tag119641
novel_sir876	TGTGATTGGCCCTGATTTCTATTA	ACTAACCGGGACTAAAGATAATCT	tag270055
novel_sir877	AGCACGGACTCCAAGACATAATGT	CATCGTGCCTGAGGTTCTGTATTA	tag218197
novel_sir878	TATTTTTACCAGCCCCAATTATTA	AAAAATGGTCGGGGTTAATAATAG	tag177940
novel_sir879	GTTGGCACACTCGGAGACTTATTA	ACCGTGTGAGCCTCTGAATAATTC	tag132984
novel_sir880	GCTGGCACACTCGGAGACTTATTA	ACCGTGTGAGCCTCTGAATAATTC	tag280063
novel_sir881	GTTGGCACACTCGGAGACTTATTA	ACCGTGTGAGCCTCTGAATAATCC	tag132984
novel_sir882	GCTGGCACACTCGGAGACTTATTA	ACCGTGTGAGCCTCTGAATAATCC	tag280063
novel_sir883	GTTCCGGTAATTAATGCAACTTA	AGGCCATTAATTACGTTGAATTG	tag47542

novel_sir884	AGTTCCGGTAATTAATGCAACTTA	AAGGCCATTAATTACGTTGAATTA	tag65578
novel_sir885	AGTTCCGGTAATTAATGCAACTTA	AAGGCCATTAATTACGTTGAATTG	tag65578
novel_sir886	GTTCAAAACCAAGCAAGGTAACTTA	AGTTTTGGTTCGTTCCATTGAATCA	tag209134
novel_sir887	AAAAATGATGTGGCAAGTGAATAA	GTTTTTTACTACACCGTTCACTTA	tag65647
novel_sir888	AAAAATGATGTGGCAAGTGAATAA	ACTTTTTACTACACCGTTCACTTA	tag264128
novel_sir889	AAAAATAATGTGGCAAGTGAATAA	ACTTTTTATTACACCGTTCACTTA	tag212905
novel_sir890	GGTTGCCCGCCGCCCGACTTA	AACGGGCGGCGGGCTGAATCC	tag165787
novel_sir891	TATCATTTATCGACAACATACTTA	AGTAAATAGCTGTTGTATGAATTG	tag275412
novel_sir892	GATAATCTAACCGATATCTACTTA	ATTAGATTGGCTATAGATGAATTG	tag256810
novel_sir893	CGTGCGGACCAAGGGTTGTACTTA	ACGCCTGGTTCCCAACATGAATAG	tag83725
novel_sir894	TTTTGCATTCTAAAACCATCCTTA	AACGTAAGATTTTGGTAGGAATGC	tag165424
novel_sir895	CGTCCTAACTCTTTTGCATCCTTA	AGGATTGAGAAAACGTAGGAATAG	tag199528
novel_sir896	TTTTGCATCCTTATTGTATCCTTA	AACGTAGGAATAACATAGGAATGT	tag45596
novel_sir897	TTTTGCATCCTAAAATTATCCTTA	AACGTAGGATTTTAATAGGAATGT	tag12195
novel_sir898	CTTGCCAAAACATTCAGCTCCTTA	ACGGTTTTGTAAGTCGAGGAATGT	tag236258
novel_sir899	TTTTGTATCCTAAGACTGTCCTTA	AACATAGGATTCTGACAGGAATGT	tag232472
novel_sir900	CTTTTCTCTGGCTCGCATTCCTTA	AAAGAGACCGAGCGTAAGGAATGC	tag157958
novel_sir901	GGTCTCGTTAGGTTCTCCAGCTTA	AGAGCAATCCAAGAGGTCGAATGT	tag253562
novel_sir902	CATGTTGCTTAGATCTGTCGCTTA	ACAACGAATCTAGACAGCGAATCC	tag129529
novel_sir903	AGACGTTTGATCGGACACGAATGA	AGTCTGCAAACTAGCCTGTGCTTA	tag1218
novel_sir904	AGTGCTATATTCAGTAAGATCTTA	ACGATATAAGTCATTCTAGAATTT	tag13716
novel_sir905	CTTACACCCTTTACGATCTTA	ATGTGGGAAATGCTAGAATGA	tag39214
novel_sir906	ATCGAAGCCAGCTAGTGAGAATAT	TCTAGCTTCGGTCGATCACTCTTA	tag109609
novel_sir907	TTTGTCAGGTGCCGAAGACTCTTA	ACAGTCCACGGCTTCTGAGAATTT	tag63699
novel_sir908	TTTGCCAGGTGTCGAAGACTCTTA	ACGGTCCACAGCTTCTGAGAATCT	tag54350
novel_sir909	ATGGTAACATGATGCTGAGAATAG	TATACCATTGTACTACGACTCTTA	tag129740
novel_sir910	CATCAAATCAAACTAGACCTCTTA	AGTTTAGTTTGATCTGGAGAATGT	tag117955
novel_sir911	CATACTGGATTTAGACGCCTCTTA	ATGACCTAAATCTGCGGAGAATAC	tag56951
novel_sir912	ATTGCGGAGATTCAGTCTTA	ACGCCTCTAAGTCAGAATCC	tag214683
novel_sir913	ACTTGCGGAGATTCAGTCTTA	AACGCCTCTAAGTCAGAATCC	tag138634
novel_sir914	CTTACTCTGATCGTCGACTTCTTA	ATGAGACTAGCAGCTGAAGAATCT	tag71190
novel_sir915	CATTAGTTCCCTGCTGTCAAGTTA	AATCAAGGGACGACAGTTCAATAC	tag220403
novel_sir916	AAACGTATGTCCAAAAGTCAATGG	AGTTTGCATACAGGTTTTCAGTTA	tag88934
novel_sir917	AAACATGTTTTAAAAAGTCAATGG	AGTTTGTACAAAATTTTTCAGTTA	tag284407

novel_sir918	AGTTTGTACACGATTTTTCAGTTA	AAACATGTGCTAAAAAGTCAATGG	tag107844
novel_sir919	AATTTGTACACGATTTTTCAGTTA	AAACATGTGCTAAAAAGTCAATGG	tag242480
novel_sir920	AAACATATGCTAAAAAGTCAATGG	AATTTGTATACGATTTTTCAGTTA	tag261803
novel_sir921	AGTTTGCAGTCAGTTTTTCAGTTA	AAACGTCAGTCAAAAAGTCAATCG	tag17313
novel_sir922	AATTTGTACAAATTTTTTCAGTTA	AAACATGTTTAAAAAAGTCAATGG	tag56190
novel_sir923	AGTTTGTACAAATTTTTTCAGTTA	AAACATGTTTAAAAAAGTCAATGG	tag145071
novel_sir924	AGTTTGTATAAATTTTTTCAGTTA	AAACATATTTAAAAAAGTCAATGG	tag230376
novel_sir925	ATAATATACTCGGCGGATCAATCC	GTTATTATATGAGCCGCCTAGTTA	tag267195
novel_sir926	ATAATACACTCGGCGGATCAATCC	GTTATTATGTGAGCCGCCTAGTTA	tag260107
novel_sir927	CCTTCCTGCAGATCCTTTTAGTTA	AAGGACGTCTAGGAAAATCAATGT	tag260442
novel_sir928	AATAGGAAACTCAGCTTGCAATGG	GGTTATCCTTTGAGTCGAACGTTA	tag83500
novel_sir929	CATTGGTAGATTAACTGTACGTTA	AACCATCTAATTGACATGCAATAG	tag35551
novel_sir930	ATAATGATGCTAGCCGCGCAATCT	TATATTACTACGATCGGCGCGTTA	tag65020
novel_sir931	TATATTACTACGATCGGCGCGTTA	ATAATGATGCTAGCCGCGCAATAT	tag65020
novel_sir932	TATATTATTATGATCGGCGCGTTA	ATAATAATACTAGCCGCGCAATCT	tag21182
novel_sir933	AACACGGACTCCAAAGCGCAATGT	CGTTGTGCCTGAGGTTTCGCGTTA	tag198366
novel_sir934	AGTATGGATTCCAAGACGCAATAC	CGTCATACCTAAGGTTCTGCGTTA	tag233522
novel_sir935	CGTCGTGCCTGAGGTTCTGCGTTA	AGCACGGACTCCAAGACGCAATAT	tag11025
novel_sir936	AGCACGGACTCCAAGACGCAATGT	CGTCGTGCCTGAGGTTCTGCGTTA	tag11025
novel_sir937	AGCACAGACTCCAAGACGCAATGT	CGTCGTGTCTGAGGTTCTGCGTTA	tag163167
novel_sir938	ATTCGCACGAACCGGACCTCGTTA	AGCGTGCTTGGCCTGGAGCAATTT	tag81469
novel_sir939	AAAACATGTACATCTCAGCAATAG	TATTTTGTACATGTAGAGTCGTTA	tag77651
novel_sir940	CCTGCCTAAGGTCCGAATTCGTTA	ACGGATTCCAGGCTTAAGCAATTG	tag155832
novel_sir941	TATATGGCCGTATCAGAAAGGTTA	ATACCGGCATAGTCTTTCCAATAT	tag207936
novel_sir942	ACCAAGCGCGATGACCAATTG	GTTGGTTCGCGCTACTGGTTA	tag100469
novel_sir943	AACCAAGCGCGATGACCAATTG	AGTTGGTTCGCGCTACTGGTTA	tag205537
novel_sir944	GCTCTCATACTGCGCACCTGGTTA	AGAGTATGACGCGTGGACCAATAT	tag29658
novel_sir945	TATGAAAAAAGATCCGCCTGGTTA	ACTTTTTTCTAGGCGGACCAATCA	tag212870
novel_sir946	ATGGAACGTTTCATAGTACAATGA	TCTACCTTGCAAAGTATCATGTTA	tag218508
novel_sir947	ATGGTCCCACCTGTCATACAATCA	CGTACCAGGGTGGACAGTATGTTA	tag52930
novel_sir948	GATACGTATCTTGTCCAGCTGTTA	ATGCATAGAACAGGTCGACAATGC	tag252395
novel_sir949	AGCACGGACTCAAAGGCACAATGT	TGTCGTGCCTGAGTTTCCGTGTTA	tag30741
novel_sir950	ACTGCGGACTCCGAGACACAATGT	CGTGACGCCTGAGGCTCTGTGTTA	tag94672
novel_sir951	AGCACGGATTACCAGACACAATGT	CATCGTGCCTAATGGTCTGTGTTA	tag218148

novel_sir952	CGTCGTGCCTGAGATTCTGTGTTA	AGCACGGACTCTAAGACACAATGT	tag12948
novel_sir953	AGCACGGACTTCAAGACACAATGT	CGTCGTGCCTGAAGTTCTGTGTTA	tag67725
novel_sir954	CGTCGCACCTGAGGTTCTGTGTTA	AGCGTGGACTCCAAGACACAATGT	tag243896
novel_sir955	CGTCGCGCCTGAGGTTCTGTGTTA	AGCGCGGACTCCAAGACACAATGT	tag12562
novel_sir956	CGTCGTGCCTGAGGTTCTGTGTTA	AGCACGGACTCCAAGACACAATGT	tag68211
novel_sir957	TATCGTGCCTGAGGTTCTGTGTTA	AGCACGGACTCCAAGACACAATGT	tag102331
novel_sir958	CATCGTGCCTGAGGTTCTGTGTTA	AGCACGGACTCCAAGACACAATGT	tag115373
novel_sir959	CGTTGATTTGCGATTCGATTGTTA	AACTAAACGCTAAGCTAACAATCT	tag166078
novel_sir960	AAAAATGTAAACCGGGTTAAATCT	TGTTTTTACATTTGGCCCAATTTA	tag286047
novel_sir961	ATTGTAGGTGCCGGTATTAAATAC	TCTAACATCCACGGCCATAATTTA	tag144884
novel_sir962	TGTGAACCGATCTGAGATTTA	ACTTGGCTAGACTCTAAATTC	tag185213
novel_sir963	ATGATGTGGCAAGTGAATAAATGA	TTTACTACACCGTTCACTTATTTA	tag143516
novel_sir964	ACTGAGCCCAACCCGCTGAAATGC	GTTGACTCGGGTTGGGCGACTTTA	tag208859
novel_sir965	GTTTGACGATCGGTTGTTCCTTTA	AACTGCTAGCCAACAAGGAAATCC	tag255843
novel_sir966	GGTTTTTCTGGCTCTGGCTCTTTA	AAAAAGACCGAGACCGAGAAATAT	tag45037
novel_sir967	AATTTTTTCAGTTACCGCAGTTTA	AAAAAAGTCAATGGCGTCAAATAT	tag102506
novel_sir968	AAAAAAGTCAACGGCGTCAAATAT	AATTTTTTCAGTTGCCGCAGTTTA	tag18310
novel_sir969	ATCGAATACTAGGCCGGCAAATCC	AGTAGCTTATGATCCGGCCGTTTA	tag85238
novel_sir970	ACGGACTCGGAATCAGGCAAATGA	TATGCCTGAGCCTTAGTCCGTTTA	tag212339
novel_sir971	GATTAATTAGGCAGTAATCGTTTA	AATTAATCCGTCATTAGCAAATGT	tag175332
novel_sir972	AGCTGTGCCAAACGGTCCAAATCC	CCTCGACACGGTTTGCCAGGTTTA	tag95420
novel_sir973	AGCTGTGCCAAACGGTCCAAATCC	CTTCGACACGGTTTGCCAGGTTTA	tag124463
novel_sir974	ATCTATAAGAGACTAGCCAAATGG	CGTAGATATTCTCTGATCGGTTTA	tag290710
novel_sir975	GATTGTATGTGATGATGTGGTTTA	AACATACACTACTACACCAAATAT	tag257918
novel_sir976	ACACTCCGTGTGGATGACAAATGG	ACTGTGAGGCACACCTACTGTTTA	tag240846
novel_sir977	ACTGTGAGGCTCACCTACTGTTTA	ACACTCCGAGTGGATGACAAATGG	tag189229
novel_sir978	GGTGGATATGTAAACAGCTGTTTA	ACCTATACATTTGTCGACAAATTT	tag110372
novel_sir979	ATAAAACATCACGACAACAAATTT	AGTATTTTGTAGTGCTGTTGTTTA	tag155007
novel_sir980	ACTGGCAGGCAGAATAAACTTTTA	ACCGTCCGTCTTATTTGAAAATTT	tag184409
novel_sir981	AGTTTATTCTGCCTGCTAGTTTTA	AAATAAGACGGACGATCAAAATTG	tag85133
novel_sir982	GGTTCTAAGCACGACGTTTTA	AAGATTCGTGCTGCAAAATGT	tag286002
novel_sir983	CATATTTGTTGGCCCTGATTTTTA	ATAAACAACCGGGACTAAAAATAG	tag239626
novel_sir984	CTTTTATACCGATAAGTCTTTTTA	AAATATGGCTATTCAGAAAAATAT	tag205757
novel_sir985	AACTAGTGGCGCCTGCAAAAATCA	TATTGATCACCGCGGACGTTTTTA	tag152125

novel_sir986	GTTGCCGCAGTAGATAATTTTTTA	ACGGCGTCATCTATTAAAAAATGG	tag74111
novel_sir987	ACGGCGTCATCTATTAAAAAATAG	GTTGCCGCAGTAGATAATTTTTTA	tag74111
novel_sir988	GTTGGTTTGTGATCGGATTTTTTA	ACCAAACACTAGCCTAAAAAATTG	tag33525
novel_sir989	ACTCCGATAATTCACAAAACAAAC	AGGCTATTAAGTGTTTTGTTTGGT	tag4177
novel_sir990	ACTCCGATAATTGACAAAACAAAC	AGGCTATTAACTGTTTTGTTTGGT	tag68213
novel_sir991	CCTGTGCCTTTGGGTCCCAAAC	ACACGGAAACCCAGGGTTTGCC	tag83123
novel_sir992	CCTCCAACTTCGGCTGCAAAC	AGGTTGAAGCCGACGTTTGGG	tag180946
novel_sir993	ATGACACAGTCGAACAAGTTTGAG	CCTACTGTGTCAGCTTGTTCAAAC	tag60937
novel_sir994	ATGACACAGTCGAACAAGTTTGAG	CTTACTGTGTCAGCTTGTTCAAAC	tag173957
novel_sir995	CGTGGACGCAAAAATCGGAAAC	ACCTGCGTTTTTAGCCTTTGGA	tag16547
novel_sir996	CCTACTCACGTAGGCCGGCTAAAC	ATGAGTGCATCCGGCCGATTTGGC	tag258935
novel_sir997	ATTATCATAGCCGTGTCATTTGTA	AGTAATAGTATCGGCACAGTAAAC	tag81134
novel_sir998	AATTCTGGGGAAAAACGAAACAAC	AAGACCCCTTTTTGCTTTGTTGAT	tag92486
novel_sir999	CGTTCGACCAGGAACCACAAC	AAGCTGGTCCTTGGTGTTGAT	tag166555
novel_sir1000	AGCCGCCGCTTCCTCCGTTGAA	CGTCGGCGGCGAAGGAGGCAAC	tag87428
novel_sir1001	ACAAAAGGTAAAGCCGTTGCC	TATGTTTTCCATTTCGGCAAC	tag262639
novel_sir1002	ATTCAGCCCGCCGCCCGTTGGG	CCTAAGTCGGGCGGCGGGCAAC	tag1592
novel_sir1003	TCTCCTAGTAGGCGATGCAAC	AGGATCATCCGCTACGTTGCG	tag2634
novel_sir1004	GTTCGCAAGTATCGCTGCAAC	AGCGTTCATAGCGACGTTGCT	tag8519
novel_sir1005	GTTAGGGACCTAGTCATCAAC	ATCCCTGGATCAGTAGTTGGT	tag100888
novel_sir1006	GGTTAGGGACCTAGTCATCAAC	AATCCCTGGATCAGTAGTTGGT	tag32572
novel_sir1007	ACCAAAACTGGGAGTGGAGTTGGG	GGTGGTTTTGACCCTCACCTCAAC	tag49731
novel_sir1008	AGCTAAATCAGATGGAGAGTTGGC	GATCGATTTAGTCTACCTCTCAAC	tag188589
novel_sir1009	GATCCGTTTAATCTACTTCTCAAC	AGGCAAATTAGATGAAGAGTTGGC	tag264198
novel_sir1010	AGCTCTGGCTCTAGGTAAGTTGGA	TGTCGAGACCGAGATCCATTCAAC	tag89232
novel_sir1011	GCTCAGAAACTTGCGTTCAAC	AGTCTTTGAACGCAAGTTGCG	tag13257
novel_sir1012	ACTCTAGGGTTAAGCGTGCTTGGC	CTTGAGATCCCAATTCGCACGAAC	tag247195
novel_sir1013	ACTCCAGGGTTAAGCGTGCTTGAC	CTTGAGGTCCCAATTCGCACGAAC	tag123331
novel_sir1014	CTTGAGGTCCCAATTCGCACGAAC	ACTCCAGGGTTAAGCGTGCTTGGC	tag123331
novel_sir1015	TCTCCACCGCCGCCACCGAAC	AGGTGGCGGCGGTGGCTTGGA	tag251076
novel_sir1016	AGGGGAGGCCGGCGGCTTGGA	CCTCCCCTCCGGCCGCCGAAC	tag98878
novel_sir1017	AGGCGCCGCTTGCTAGCTTGGA	CGTCCGCGGCGAACGATCGAAC	tag272251
novel_sir1018	TTTCCCTACATCGCGTCGAAC	AGGGATGTAGCGCAGCTTGGA	tag8173
novel_sir1019	TTTCCCTACATCGCGTCGAAC	AGGGATGTAGCGCAGCTTGGT	tag8173

novel_sir1020	CATTCACCGTCTCACCGGAAC	AAGTGGCAGAGTGGCCTTGCT	tag113901
novel_sir1021	AGGTTGCTCATATATCGGAAC	CAACGAGTATATAGCCTTGGC	tag92166
novel_sir1022	CCGTTTGGATTGACCTGGAAC	CAAACCTAACTGGACCTTGTT	tag106589
novel_sir1023	ATGGCCTCGGGCGCAACTTGCG	CCTACCGGAGCCCGCGTTGAAC	tag31325
novel_sir1024	TATCGCTAGAAATCAAGGCCTAAC	AGCGATCTTTAGTTCCGGATTGGT	tag219870
novel_sir1025	ATTTATGCGCTGCCCCGTAAC	AATACGCGACGGGGCATTGTA	tag103308
novel_sir1026	ATTTATGCGCTGCCCCGTAAC	AATACGCGACGGGGCATTGTT	tag103308
novel_sir1027	AGACATGAGCGCCACATTGCC	CCTCTGTACTCGCGGTGTAAC	tag74617
novel_sir1028	CCTCTAGCCGCGACTCGTTAAC	AGATCGGCGCTGAGCAATTGGG	tag227561
novel_sir1029	CAAGCGCGATGACCAATTGTG	TGGTTCGCGCTACTGGTTAAC	tag243224
novel_sir1030	AACCAAGCGCGATGACCAATTGTG	AGTTGGTTCGCGCTACTGGTTAAC	tag258216
novel_sir1031	CAAGCGAAACAGGATTGCTGTGCC	AGGTTCGCTTTGTCCTAACGACAC	tag93658
novel_sir1032	ATCCGGCCAAACGTTCGATGTGAC	TGTAGGCCGGTTTGCAAGCTACAC	tag263205
novel_sir1033	ACGAATGGTCAAACATGTGAG	TCTGCTTACCAGTTTGTACAC	tag201461
novel_sir1034	AGCTGGATCCGGAGCTGGTGCG	CCTCGACCTAGGCCTCGACCAC	tag133931
novel_sir1035	CAGTTCGCACTTACCCTATACCAC	CAAGCGTGAATGGGATATGGTGGT	tag51568
novel_sir1036	GATGTACAGTAACTCTATGCCCAC	ACATGTCATTGAGATACGGGTGGC	tag139184
novel_sir1037	CGTGCTGTCGTCGGTAGCCAC	ACGACAGCAGCCATCGGTGGC	tag257789
novel_sir1038	AGTTCTGATGGGATCCGGTGCT	CCTCAAGACTACCCTAGGCCAC	tag168128
novel_sir1039	CAAACTTACAAAACCGGTGCA	GGGTTTGAATGTTTTGGCCAC	tag128539
novel_sir1040	AGGTCTGGACTGCGAGACGGTGGT	GCTCCAGACCTGACGCTCTGCCAC	tag59035
novel_sir1041	ACAGCACAGCGGAGGAGGTGGA	GCTGTCGTGTCGCCTCCTCCAC	tag96696
novel_sir1042	ACTGCCGGCCGCTGCTCTCCAC	ACGGCCGGCGACGAGAGGTGGC	tag181246
novel_sir1043	CCTACACGAACGAGGCAAGCAC	ATGTGCTTGCTCCGTTCGTGAA	tag277694
novel_sir1044	CTTCCTCTCCCGCTGCAGCAC	AGGAGAGGGCGACGTCGTGCG	tag10233
novel_sir1045	TGTGTATTACAAAACCTCAGGCAC	ACATAATGTTTTGGAGTCCGTGCT	tag87766
novel_sir1046	TGTGTGACGCAGAACCTCAGGCAC	ACACTGCGTCTTGGAGTCCGTGCT	tag273512
novel_sir1047	AGGTCGCCACCCATGCCGTGGC	GCTCCAGCGGTGGGTACGGCAC	tag266453
novel_sir1048	TGGTGGGGGTAGAAAATTCGGCAC	CACCCCCATCTTTTAAGCCGTGCT	tag180854
novel_sir1049	CACCTCAACCGTACATATAGTGGC	TGGTGGAGTTGGCATGTATATCAC	tag273346
novel_sir1050	TTTCCGCGGCCGGCCGCTCAC	AGGCGCCGGCCGGCGAGTGGG	tag185974
novel_sir1051	CCTCGCGGCGGCCAGGCTCAC	AGCGCCGCCGGTCCGAGTGAA	tag191782
novel_sir1052	GGGTGGCGGCGGCTATCTCAC	CACCGCCGCCGATAGAGTGAA	tag58958
novel_sir1053	CACCGACGCGATTCCAGTGCT	GGGTGGCTGCGCTAAGGTCAC	tag225016

novel_sir1054	CATTGATCAGTGCCCGGTCTTCAC	AACTAGTCACGGGCCAGAAGTGGA	tag42553
novel_sir1055	GATCGTCACTCTCCTCAAGAC	AGCAGTGAGAGGAGTTCTGGC	tag221229
novel_sir1056	CAAACGTCTTCTCCTTCTGGA	CGGTTTGCAGAAGAGGAAGAC	tag128587
novel_sir1057	TGTTGTTGCCTCCGTTAAGAC	AACAACGGAGGCAATTCTGCC	tag6246
novel_sir1058	ACTTAACGGACAGATACAGAC	AATTGCCTGTCTATGTCTGTG	tag132229
novel_sir1059	AGATCTGTAGCCCGCTGCTCTGCT	TGTCTAGACATCGGGCGACGAGAC	tag147605
novel_sir1060	GTTCCACCACCCGATCTCCTAGAC	AGGTGGTGGGCTAGAGGATCTGGA	tag186526
novel_sir1061	CCGTTTGAAAATCGAGGTCTAGAC	CAAACTTTTAGCTCCAGATCTGAA	tag88854
novel_sir1062	ATTAAACTCGGCTCAATCTGAA	GTTAATTTGAGCCGAGTTAGAC	tag198943
novel_sir1063	ATTAAACTCGGCTCAATCTGAA	ATTAATTTGAGCCGAGTTAGAC	tag242465
novel_sir1064	AATTAAACTCGGCTCAATCTGAA	AGTTAATTTGAGCCGAGTTAGAC	tag94679
novel_sir1065	AGGTGGAGGAGGCGACACGAC	CACCTCCTCCGCTGTGCTGTC	tag256855
novel_sir1066	CCGTTTGATCTGAAAGCCGAC	CAAACTAGACTTTCGGCTGGC	tag114793
novel_sir1067	GGGTGGACGCCCAGAAGCCGAC	CACCTGCGGGTCTTCGGCTGCC	tag200347
novel_sir1068	AGTTGCCTCCTTCGCCGCCGAC	AACGGAGGAAGCGGCGGCTGCA	tag284548
novel_sir1069	CCTGCTTCCGCTGGCGCCGAC	ACGAAGGCGACCGCGGCTGCG	tag106207
novel_sir1070	ACGCTCCGCCGGGCCGCTGCC	GCTGCGAGGCGGCCCGGCGAC	tag47145
novel_sir1071	CATTTCGGCAACGGGTGCGAC	AAAGCCGTTGCCCACGCTGGC	tag245382
novel_sir1072	GGTGGTTTTGACCCTCACCTCGAC	ACCAAAACTGGGAGTGGAGCTGGG	tag64184
novel_sir1073	TGTTGAATAGATCTCGGCCTCGAC	AACTTATCTAGAGCCGGAGCTGTG	tag242731
novel_sir1074	ACGTAGTCAACGCGAGCTGAT	CCTGCATCAGTTGCGCTCGAC	tag259696
novel_sir1075	CGGTTTCTCCGACCGCGGCAGGAC	CAAAGAGGCTGGCGCCGTCCTGTT	tag111950
novel_sir1076	GTTCGATCGTTCGCCGCGGAC	AGCTAGCAAGCGGCGCCTGCG	tag269585
novel_sir1077	CACATCAGTGACTTCTAGCCTGTG	AAGTGTAGTCACTGAAGATCGGAC	tag22367
novel_sir1078	TGGTCGTGACAAAGGACCCTGAC	CAGCACTGTTTCCTGGGACTGGT	tag30637
novel_sir1079	ACCCTCTAGATCCGCCACTGCT	GGTGGGAGATCTAGGCGGTGAC	tag276906
novel_sir1080	CACCCTCTAGATCCGCCACTGCT	TGGTGGGAGATCTAGGCGGTGAC	tag21457
novel_sir1081	CAGACACATACCTAACAAACTGTG	TGGTCTGTGTATGGATTGTTTGAC	tag140854
novel_sir1082	CACCTCACTATTTTAGCTTATGTG	AGGTGGAGTGATAAAATCGAATAC	tag290293
novel_sir1083	TCTCCCACCAGGCCAACTTAATAC	AGGGTGGTCCGGTTGAATTATGGA	tag165097
novel_sir1084	CACTACTGGCGTGAAGACTATGAG	GGGTGATGACCGCACTTCTGATAC	tag76931
novel_sir1085	AATGGTACCTCACTCGGATGTG	CTTTACCATGGAGTGAGCCTAC	tag232544
novel_sir1086	CAACATACTTGCCAACCGGATGGC	AGGTTGTATGAACGGTTGGCCTAC	tag117764
novel_sir1087	ACGTGTTCCTAAGGTTTCCTAC	CACAAGGATTCCAAAGGATGGC	tag139176

novel_sir1088	ACATCCGGTCAAACGTTCGATGTG	GCTGTAGGCCAGTTTGCAAGCTAC	tag129336
novel_sir1089	CCTTCGGGGCTCGCGGCGCTAC	AAGCCCCGAGCGCCGCGATGGC	tag9217
novel_sir1090	TGGTTCTTGTTATGATCGATCTAC	CAAGAACAATACTAGCTAGATGTT	tag89661
novel_sir1091	ACGGCGCAAACCGACAAAGATGGA	AATGCCGCGTTTGGCTGTTTCTAC	tag169869
novel_sir1092	CACCGACAGCGAGACGTTCATGAT	CGGTGGCTGTCGCTCTGCAAGTAC	tag111709
novel_sir1093	GGTCGCCAGGAATTTGAACAGTAC	AGCGGTCCTTAAACTTGTCATGAG	tag198073
novel_sir1094	TAGTTGCCGTCGGTGCCGTAC	CAACGGCAGCCACGGCATGGG	tag274678
novel_sir1095	CAGTAGTTGCCGTCGGTGCCGTAC	CATCAACGGCAGCCACGGCATGGG	tag52935
novel_sir1096	CGGTGCTGTCGACGCACATCGTAC	CACGACAGCTGCGTGTAGCATGAC	tag31531
novel_sir1097	CGTGGGAGAATCTGATCGTAC	ACCCTCTTAGACTAGCATGGC	tag95825
novel_sir1098	CACGACTTTCAAAACCATGTG	TCGTGCTGAAAGTTTTGGTAC	tag22470
novel_sir1099	TTGTTCGTATTTGTCATGTAC	CAAGCATAAACAGTACATGTG	tag148079
novel_sir1100	TGGTTCTTGTTATGATCGATGTAC	CAAGAACAATACTAGCTACATGTT	tag10184
novel_sir1101	AGATGCGGCATGTGGAGACATGAG	ATTCTACGCCGTACACCTCTGTAC	tag12649
novel_sir1102	ATTCTGCTTGCCAGTTTGTAC	AGACGAACGGTCAAACATGTT	tag273370
novel_sir1103	ATTCTGCTTGCCAGTTTGTAC	AGACGAACGGTCAAACATGTG	tag273370
novel_sir1104	CGGTTCCTGTCTGCTAGTTTGTAC	CAAGGACAGACGATCAAACATGAG	tag207582
novel_sir1105	TCTCGCAAACATCCTTCAAATTAC	AGCGTTTGTAGGAAGTTTAATGGA	tag98838
novel_sir1106	GAGTCAGACCGTGGGGCGTTAC	CAGTCTGGCACCCCGCAATGAA	tag134307
novel_sir1107	CACCCCCAAAAGCTAGCCAATGAG	AGGTGGGGGTTTTCGATCGGTTAC	tag194737
novel_sir1108	CAGTATAGCGCTAACAATGGG	GTGTCATATCGCGATTGTTAC	tag89920
novel_sir1109	TGTGTCATATCGCGATTGTTAC	ACAGTATAGCGCTAACAATGGG	tag32304
novel_sir1110	CGGTTTAAGCGTCGTTTGATTTAC	CAAATTCGCAGCAAACTAAATGGT	tag75485
novel_sir1111	AGAAGAAATGGATGAAATGAC	ATTCTTCTTTACCTACTTTAC	tag132812
novel_sir1112	CACTTTCGGCCCGTCACAAATGAC	AGGTGAAAGCCGGGCAGTGTTTAC	tag112191
novel_sir1113	GGTGCGGCTAGACGGCAAACC	ACGCCGATCTGCCGTTTGGAT	tag64233
novel_sir1114	AGAGGACGAGAAATGTTGGCA	GCTCTCCTGCTCTTTACAACC	tag256864
novel_sir1115	GCTAGCGAGCTCTTGCCAACC	ATCGCTCGAGAACGGTTGGTG	tag986
novel_sir1116	ATCCTCTCCTAAGTCGTTGGGC	TTTAGGAGAGGATTCAGCAACC	tag293770
novel_sir1117	AAGGCTATATACTCGTTGGAT	GGTTCCGATATATGAGCAACC	tag59429
novel_sir1118	CAAGGCTATATACTCGTTGGAT	CGGTTCCGATATATGAGCAACC	tag291468
novel_sir1119	AGGGGAGGCCGGCGGCTTGGAC	CCTCCCCTCCGGCCGCCGAACC	tag16492
novel_sir1120	ACGTCGCTATGAACGCTTGGCT	GTTGCAGCGATACTTGCGAACC	tag254111
novel_sir1121	TTTCCCTACATCGCGTCGAACC	AGGGATGTAGCGCAGCTTGGCA	tag17034

novel_sir1122	AGGGATGTAGCGCAGCTTGGTG	TTTCCCTACATCGCGTCGAACC	tag17034
novel_sir1123	TTTCCCTACATCGCGTCGAACC	AGGGATGTAGCGCAGCTTGGAA	tag17034
novel_sir1124	TTTCCCTACATCGCGTCGAACC	AGGGATGTAGCGCAGCTTGGTA	tag17034
novel_sir1125	TTTCCCTACATCGCGTCGAACC	AGGGATGTAGCGCAGCTTGGTC	tag17034
novel_sir1126	AGGGATGTAGCGCAGCTTGGTT	TTTCCCTACATCGCGTCGAACC	tag17034
novel_sir1127	ACATCAACGGTGTAGATTGTGGGT	TGTGTAGTTGCCACATCTAACACC	tag139574
novel_sir1128	GTTTGCCTCGAGCCCTACACC	AACGGAGCTCGGGATGTGGCG	tag158753
novel_sir1129	TCGTCACCGCCTAGATCTCCCACC	CAGTGGCGGATCTAGAGGGTGGTC	tag172084
novel_sir1130	AAGCTGACGTGGCAGACGGTGGGT	CGTTCGACTGCACCGTCTGCCACC	tag148921
novel_sir1131	CACGCACAAGGTCCTTAGGTGGCT	CAGTGCGTGTTCCAGGAATCCACC	tag63785
novel_sir1132	CCTCCAAGGCCGGCATCCACC	AGGTTCCGGCCGTAGGTGGGG	tag61194
novel_sir1133	TAGTAACGGCCAGCCGCGGGCACC	CATTGCCGGTCGGCGCCCGTGGTT	tag34220
novel_sir1134	AGGTACAGTAGCAGGTGCACC	CATGTCATCGTCCACGTGGCG	tag212766
novel_sir1135	CTGGTTCAGTTTCGTCGGTGCACC	CCAAGTCAAAGCAGCCACGTGGGT	tag233661
novel_sir1136	AGGCGCCGGCCGGCGAGTGGGG	TTTCCGCGGCCGGCCGCTCACC	tag283540
novel_sir1137	CGTAACATTCACCGTCTCACC	ATTGTAAGTGGCAGAGTGGCC	tag54018
novel_sir1138	CCGTAACATTCACCGTCTCACC	CATTGTAAGTGGCAGAGTGGCC	tag218839
novel_sir1139	GCTCACGTTGTTGAACTGTTCACC	AGTGCAACAACTTGACAAGTGGGT	tag7058
novel_sir1140	CGGTTTGCAGAAGAGGAAGACC	CAAACGTCTTCTCCTTCTGGAA	tag68890
novel_sir1141	CAAGTGCACTCAGCCGGTCTGGAG	TCGTTCACGTGAGTCGGCCAGACC	tag117825
novel_sir1142	AATCCTTATTCAGAACCGACC	AGGAATAAGTCTTGGCTGGTT	tag25062
novel_sir1143	TGGTGATAAGCGCAATAGCCGACC	CACTATTCGCGTTATCGGCTGGGT	tag94836
novel_sir1144	CCTCGACCTAGGCCTCGACC	AGCTGGATCCGGAGCTGGTG	tag40662
novel_sir1145	CCACAGATTGGCAAGCTGGTC	GGGGTGTCTAACCGTTCGACC	tag91685
novel_sir1146	AGGCCGGCGGCTTGGACTGGGC	CCTCCGGCCGCCGAACCTGACC	tag187472
novel_sir1147	CACGAGACCTAAAAGTGGATGGAT	GCGTGCTCTGGATTTTCACCTACC	tag175935
novel_sir1148	CCTTACTCTGATCAGGATCCTACC	AATGAGACTAGTCCTAGGATGGGT	tag132719
novel_sir1149	AGGACGACGACGAGGATGGCA	CCTCCTGCTGCTGCTCCTACC	tag240781
novel_sir1150	AGAACTAGGAGAAAGATGGGA	GTTCTTGATCCTCTTTCTACC	tag280187
novel_sir1151	AGTTGCCGTCGGTGCCGTACC	AACGGCAGCCACGGCATGGGT	tag136389
novel_sir1152	TAGTTGCCGTCGGTGCCGTACC	CAACGGCAGCCACGGCATGGGT	tag140150
novel_sir1153	TCTCGAGCGGCCGCCGGTACC	AGCTCGCCGGCGGCCATGGCC	tag14322
novel_sir1154	AGTATAGCGCTAACAATGGGA	TGTCATATCGCGATTGTTACC	tag277208
novel_sir1155	GTGTCATATCGCGATTGTTACC	CAGTATAGCGCTAACAATGGGA	tag75593

novel_sir1156	TGGTCGAACGGTTAGACACCC	CAGCTTGCCAATCTGTGGGGT	tag223293
novel_sir1157	AGGCGAGCGCGGCGGTGGGTC	CGTCCGCTCGCGCCGCCACCC	tag242523
novel_sir1158	GTTCGACTGCACCGTCTGCCACCC	AGCTGACGTGGCAGACGGTGGGTC	tag272318
novel_sir1159	AGTGCGAGCGGCGCGTGGGGG	CCTCACGCTCGCCGCGCACCC	tag202626
novel_sir1160	AGAGGTGACGGCGCGTGGGGA	GCTCTCCACTGCCGCGCACCC	tag165029
novel_sir1161	GCTGCCGCGCCGCGCCGACCC	ACGGCGCGGCGCGGCTGGGCG	tag84748
novel_sir1162	CCACAGGCAGTCGACTGGGGA	ACGGTGTCCGTCAGCTGACCC	tag199129
novel_sir1163	AGTTGCCGTCGGTGCCGTACCC	AACGGCAGCCACGGCATGGGTG	tag290707
novel_sir1164	CACAGGCAGTCGACTGGGGAA	CGGTGTCCGTCAGCTGACCCC	tag69055
novel_sir1165	AGGGTTTGCTAGGCTCCCCC	CCAAACGATCCGAGGGGGCG	tag61876
novel_sir1166	AAGGGTTTGCTAGGCTCCCCC	CCCAAACGATCCGAGGGGGCC	tag47462
novel_sir1167	CAGGGTTTGCTAGGCTCCCCC	CCCAAACGATCCGAGGGGGCC	tag187673
novel_sir1168	CCCAAACGATCCGAGGGGGCG	AAGGGTTTGCTAGGCTCCCCC	tag47462
novel_sir1169	CCCAAACGATCCGAGGGGGCG	CAGGGTTTGCTAGGCTCCCCC	tag187673
novel_sir1170	GCTCCGACCGCTGTCCGCCCC	AGGCTGGCGACAGGCGGGGGC	tag62905
novel_sir1171	CACTCCGGGTTGAGCCAAGGGGGT	TGGTGAGGCCCAACTCGGTTCCCC	tag192293
novel_sir1172	CCTTCGCCGCGGCCACCGCCC	AAGCGGCGCCGGTGGCGGGGG	tag200017
novel_sir1173	AAGCGGCGCCGGTGGCGGGGA	CCTTCGCCGCGGCCACCGCCC	tag200017
novel_sir1174	GCTCGCCACTACCGCGCGCCC	AGCGGTGATGGCGCGCGGGGA	tag127289
novel_sir1175	CAACGGCGTCAAATATTTAGGGAT	CAGTTGCCGCAGTTTATAAATCCC	tag128573
novel_sir1176	CAACGGCGTCAAATATTTAGGGAC	CAGTTGCCGCAGTTTATAAATCCC	tag128573
novel_sir1177	CAGTTGCCGCAGTTTGTAAATCCC	CAACGGCGTCAAACATTTAGGGAC	tag624
novel_sir1178	CTGTCAACCGCGCGGTCCATCCC	CAGTTGGCGCGCCAGGTAGGGGT	tag45582
novel_sir1179	AGGTGACGGCGCGTGGGGAGGGAG	TCTCCACTGCCGCGCACCCCTCCC	tag20829
novel_sir1180	CATTCGTCACCGCCTAGATCTCCC	AAGCAGTGGCGGATCTAGAGGGTG	tag201007
novel_sir1181	CATTCGTCACCGCCTAGATCTCCC	AAGCAGTGGCGGATCTAGAGGGTA	tag201007
novel_sir1182	CATGGTTGATGACTAGGTCCC	ACCAACTACTGATCCAGGGAT	tag231808
novel_sir1183	CAGGTTTGTAGGCTACACTGTCCC	CCAAACATCCGATGTGACAGGGAC	tag186760
novel_sir1184	CCTTCGCCGCCGACGTGTCCC	AAGCGGCGGCTGCACAGGGCA	tag44317
novel_sir1185	TAGTGCTTCTAATCGTGTCCC	CACGAAGATTAGCACAGGGCG	tag65160
novel_sir1186	CAGTACCCCAATGCAAGGGCC	TCGTCATGGGGTTACGTTCCC	tag230509
novel_sir1187	CAGTACCCCAATGCAAGGGCG	TCGTCATGGGGTTACGTTCCC	tag230509
novel_sir1188	GGTCGCTTTGGTGTCGGTTCCC	AGCGAAACCACAGCCAAGGGAA	tag125934
novel_sir1189	AGCGACGACGGCACTTTCGGCG	TTTCGCTGCTGCCGTGAAAGCC	tag156312

novel_sir1190	AGGGCTATAGCTCAGTTCGGGA	GTTCCCGATATCGAGTCAAGCC	tag241820
novel_sir1191	AGGGCTATAGCTCAGTTCGGGA	ATTCCCGATATCGAGTCAAGCC	tag266313
novel_sir1192	AGGGCTATAGCTCAGTTCGGTA	GTTCCCGATATCGAGTCAAGCC	tag241820
novel_sir1193	AGGGCTATAGCTCAGTTCGGTA	ATTCCCGATATCGAGTCAAGCC	tag266313
novel_sir1194	AGGGCTATAGCTCAGTTCGGCA	GTTCCCGATATCGAGTCAAGCC	tag241820
novel_sir1195	AGGGCTATAGCTCAGTTCGGCA	ATTCCCGATATCGAGTCAAGCC	tag266313
novel_sir1196	GTTCCCGATATCGAGTCAAGCC	AGGGCTATAGCTCAGTTCGGAA	tag241820
novel_sir1197	ATTCCCGATATCGAGTCAAGCC	AGGGCTATAGCTCAGTTCGGAA	tag266313
novel_sir1198	AGGGCTATAGCTCAGTTCGGTG	GTTCCCGATATCGAGTCAAGCC	tag241820
novel_sir1199	AGGGCTATAGCTCAGTTCGGTG	ATTCCCGATATCGAGTCAAGCC	tag266313
novel_sir1200	AGGGCTATAGCTCAGTTCGGTT	GTTCCCGATATCGAGTCAAGCC	tag241820
novel_sir1201	AGGGCTATAGCTCAGTTCGGTT	ATTCCCGATATCGAGTCAAGCC	tag266313
novel_sir1202	CAGGGTCGGGTTGCCAGTTCGGTA	GGGTCCCAGCCCAACGGTCAAGCC	tag86622
novel_sir1203	CAGGGTCGGGTTGCCAGTTCGGTC	GGGTCCCAGCCCAACGGTCAAGCC	tag86622
novel_sir1204	CATCGTCCATCCTTGTCGGCG	TCGTAGCAGGTAGGAACAGCC	tag273175
novel_sir1205	CAGACGTTTGACCGGATGTCGGAA	TAGTCTGCAAACTGGCCTACAGCC	tag272111
novel_sir1206	CAAACGTTTGACCGGATGTCGGAA	TAGTTTGCAAACTGGCCTACAGCC	tag233280
novel_sir1207	GCTCGACCCTGGGTCCCAGCC	AGCTGGGACCCAGGGTCGGGT	tag34692
novel_sir1208	AGCTCAGTGGTAGAGCGGTCGGCC	CATCGAGTCACCATCTCGCCAGCC	tag64253
novel_sir1209	CATCGAGTCACCATCTCGCCAGCC	AGCTCAGTGGTAGAGCGGTCGGCT	tag64253
novel_sir1210	AGGTCGAGGACAGCCGCAGCC	CAGCTCCTGTCGGCGTCGGCG	tag182204
novel_sir1211	TTTGAAAGGACCAGCGCAGCC	ACTTTCCTGGTCGCGTCGGGG	tag62218
novel_sir1212	ATAAGAGGAGCCTACGCGTCGGGC	CTTATTCTCCTCGGATGCGCAGCC	tag18271
novel_sir1213	ACTTGGCCTTCTCCGTCGGCT	CCTGAACCGGAAGAGGCAGCC	tag61143
novel_sir1214	CCCTCACCATGCTAGTCGGCG	CAGGGAGTGGTACGATCAGCC	tag176498
novel_sir1215	AGCGGAGGGCCATTCTCGGGG	GGTCGCCTCCCGGTAAGAGCC	tag59317
novel_sir1216	CAGCGGAGGGCCATTCTCGGGG	AGGTCGCCTCCCGGTAAGAGCC	tag136115
novel_sir1217	ACGGCGGCGGCTTGCTCGGCG	GCTGCCGCCGCCGAACGAGCC	tag181280
novel_sir1218	ATTAAACGTTTGCCTCGAGCC	ATTTGCAAACGGAGCTCGGGA	tag107174
novel_sir1219	GATTAAACGTTTGCCTCGAGCC	AATTTGCAAACGGAGCTCGGGA	tag173842
novel_sir1220	TGGGTGCACCGGACTGGGGGAGCC	CCACGTGGCCTGACCCCCTCGGGG	tag108636
novel_sir1221	CAGACGTTTGACCGGATATCGGAA	TAGTCTGCAAACTGGCCTATAGCC	tag93105
novel_sir1222	GCTCTTTGTCGCTAGCTAGCC	AGAAACAGCGATCGATCGGCT	tag78511
novel_sir1223	AGCCTAGGGGTTATGGAATCGGTT	TATCGGATCCCCAATACCTTAGCC	tag51878

novel_sir1224	CCTCTCCCGCTGCAGCACGCC	AGAGGGCGACGTCGTGCGGGC	tag192991
novel_sir1225	AGAGGGCGTTGCCGTGCGGGC	CCTCTCCCGCAACGGCACGCC	tag171426
novel_sir1226	AGGAGAGCAGCGGCTGCGGGC	CCTCCTCTCGTCGCCGACGCC	tag94422
novel_sir1227	ATGTCAGGCAAGCCAGATGCGGGA	GATACAGTCCGTTCGGTCTACGCC	tag220069
novel_sir1228	ACGTGGGTCGGGGCGGGCGGCG	ATTGCACCCAGCCCCGCCCGCC	tag233170
novel_sir1229	ACGCGGCGGCGGCGGGCGGGT	CCTGCGCCGCCGCCGCCCGCC	tag221150
novel_sir1230	GTGTCCCGTGTCGTGCCCGCC	CAGGGCACAGCACGGGCGGCT	tag72433
novel_sir1231	CGTGTCCCGTGTCGTGCCCGCC	ACAGGGCACAGCACGGGCGGCT	tag272266
novel_sir1232	GAGGGAAGGGTTGCCCGCCGCC	CCCTTCCCAACGGGCGGCGGGC	tag63266
novel_sir1233	AGGGTGTCGTCGGCGGCGGGA	CCTCCCACAGCAGCCGCCGCC	tag193739
novel_sir1234	CCTCCTCCGCTCGCCGCCGCC	AGGAGGCGAGCGGCGGCGGGG	tag66
novel_sir1235	CATCCGCTCGAGGCCGCCGCC	AGGCGAGCTCCGGCGGCGGCG	tag59009
novel_sir1236	CGTCCGCTCGAGGCCGCCGCC	AGGCGAGCTCCGGCGGCGGCG	tag145064
novel_sir1237	CGTTCGACTGCACCGTCTGCCGCC	AAGCTGACGTGGCAGACGGCGGGT	tag33070
novel_sir1238	GCTGCGCGAACCGCATCCGCC	ACGCGCTTGGCGTAGGCGGTC	tag291392
novel_sir1239	CACCCGAGTGATGCATAGGCGGTC	TAGTGGGCTCACTACGTATCCGCC	tag199249
novel_sir1240	TATCTGCCAGCTGGGTCCGCC	AGACGGTCGACCCAGGCGGAT	tag59900
novel_sir1241	CCTTAGCTGCCGCGCCGCGCC	AATCGACGGCGCGGCGCGGCT	tag8725
novel_sir1242	GCTCCTGCTTCCGCTGGCGCC	AGGACGAAGGCGACCGCGGCT	tag284012
novel_sir1243	ACGAATCCGTGCGACGCGGGG	GCTGCTTAGGCACGCTGCGCC	tag79876
novel_sir1244	ACGAATCCGTGCGACGCGGGG	ACTGCTTAGGCACGCTGCGCC	tag126542
novel_sir1245	ACGAATCCGTGCGACGCGGGG	CCTGCTTAGGCACGCTGCGCC	tag206166
novel_sir1246	ACGAATCCGTGCGACGCGGGG	CTTGCTTAGGCACGCTGCGCC	tag249519
novel_sir1247	CATACCCATATCTGGATCGCC	ATGGGTATAGACCTAGCGGAG	tag157906
novel_sir1248	CCTCCTCTCTACCGGCTCGCC	AGGAGAGATGGCCGAGCGGTT	tag136837
novel_sir1249	TCTTAGGTTCGATCGTTCGCC	AATCCAAGCTAGCAAGCGGCG	tag267036
novel_sir1250	CAGTCTTAGGTTCGATCGTTCGCC	CAGAATCCAAGCTAGCAAGCGGCG	tag216641
novel_sir1251	CACGATGTGGCAGGTCTTCCGGTT	CAGTGCTACACCGTCCAGAAGGCC	tag202326
novel_sir1252	CCGGCTGGAGAGATCGAGGCC	CCGACCTCTCTAGCTCCGGCG	tag227229
novel_sir1253	GCTCAGGGAGCCCGGCTAGGCC	AGTCCCTCGGGCCGATCCGGGC	tag287782
novel_sir1254	CCTCGATCTCTCCAGCCGGCC	AGCTAGAGAGGTCGGCCGGCC	tag232122
novel_sir1255	AGCTTCGAGGCGGCCGGCC	CCTCGAAGCTCCGCCGGCC	tag100964
novel_sir1256	GCTTCCCCCTCCGCGCCGGCC	AAGGGGGAGGCGCGGCCGGGG	tag216134
novel_sir1257	GCGGCGGTGGAGAGCAGCGGCC	CCGCCACCTCTCGTCGCCGGCC	tag228409

novel_sir1258	CCGAACCTGTATACATCGCCGGCG	CGGGCTTGGACATATGTAGCGGCC	tag272148
novel_sir1259	AGAGGGAAAAAGGCGCCGGCC	CCTCTCCCTTTTTCCGCGGCC	tag114162
novel_sir1260	GCGTCGGTAGGGGCGGCGGCC	CAGCCATCCCCGCCGCCGGCC	tag223548
novel_sir1261	CGTCCGACTTCGGCTGCGGCC	AGGCTGAAGCCGACGCCGGTC	tag40956
novel_sir1262	CAGGGAGTGGCACGATCGGCC	CCCTCACCGTGCTAGCCGGCG	tag141805
novel_sir1263	TATGGACATTGCCCGGTATCGGCC	ACCTGTAACGGGCCATAGCCGGAT	tag216702
novel_sir1264	AATGGATACTGCCCAGTGTCGGCC	ACCTATGACGGGTCACAGCCGGAC	tag52835
novel_sir1265	TTTCTAGCGATAGAAATCAGGGCC	AGATCGCTATCTTTAGTCCCGGGC	tag35625
novel_sir1266	CCGATTTCCACAATAGTCCCGGAT	CCGGCTAAAGGTGTTATCAGGGCC	tag135385
novel_sir1267	GAGTACCGTTAATTCCTGGCC	CATGGCAATTAAGGACCGGTT	tag36275
novel_sir1268	GAGTACTGTTAATTCCTGGCC	CATGACAATTAAGGACCGGTT	tag127624
novel_sir1269	GGGTTCCCGGGCACGGTGGCC	CAAGGGCCCGTGCCACCGGCC	tag81821
novel_sir1270	GGGGTTCCCGGGCACGGTGGCC	CCAAGGGCCCGTGCCACCGGCC	tag185798
novel_sir1271	GCGGCTGCCGCCGATCGATGCC	CCGACGGCGGCTAGCTACGGCG	tag205373
novel_sir1272	CCGATTTTGGTCTACGTGACGGCT	AGGGCTAAAACCAGATGCACTGCC	tag276006
novel_sir1273	CCAAGATCTGCACCGACGGCC	GTGGTTCTAGACGTGGCTGCC	tag76185
novel_sir1274	GGTTGCGCTGCTTGCTGCTGCC	AACGCGACGAACGACGACGGAT	tag252578
novel_sir1275	TGGTCGCTTTTGGCTAAAAGTGCC	CAGCGAAAACCGATTTTCACGGGT	tag237998
novel_sir1276	CCGCACGTTGCCGCTGTCACGGAC	TCGGCGTGCAACGGCGACAGTGCC	tag38874
novel_sir1277	AATTACTATCGCGGCACGGTC	ACTTAATGATAGCGCCGTGCC	tag194103
novel_sir1278	CAGGCAAATGATATATCCACGGAC	TAGTCCGTTTACTATATAGGTGCC	tag36418
novel_sir1279	CCGGCGATCACCACCACGGCT	GCGGCCGCTAGTGGTGGTGCC	tag125596
novel_sir1280	GCGGTCGCTAGTGGTGGTGCC	CCAGCGATCACCACCACGGCT	tag256956
novel_sir1281	TTTGTACACTCTTTTTCAGTTGCC	ACATGTGAGAAAAAGTCAACGGTG	tag35496
novel_sir1282	TTTGTACACTCTTTTTCAGTTGCC	ACATGTGAGAAAAAGTCAACGGCG	tag35496
novel_sir1283	TTTGTACACTCTTTTTCAGTTGCC	ACATGTGAGAAAAAGTCAACGGCA	tag35496
novel_sir1284	TTTGTACAAATTTTTTCAGTTGCC	ACATGTTTAAAAAAGTCAACGGTG	tag230404
novel_sir1285	ACATGTTTAAAAAAGTCAACGGCG	TTTGTACAAATTTTTTCAGTTGCC	tag230404
novel_sir1286	ACATGTTTAAAAAAGTCAACGGGC	TTTGTACAAATTTTTTCAGTTGCC	tag230404
novel_sir1287	TTTGTACAAATTTTTTCAGTTGCC	ACATGTTTAAAAAAGTCAACGGCA	tag230404
novel_sir1288	CAGTGCTACTGCAGTAGTTGCC	CACGATGACGTCATCAACGGCA	tag245389
novel_sir1289	ACGACGAGGATGGCAACGGAG	GCTGCTGCTCCTACCGTTGCC	tag143554
novel_sir1290	ACGAGTGATCTGCAAGTTTAGGGA	TTTGCTCACTAGACGTTCAAATCC	tag71471
novel_sir1291	ACGAGTGATCTGCAAGTTTAGGAC	TTTGCTCACTAGACGTTCAAATCC	tag71471

novel_sir1292	ACCGGTCAGATCGCGGGTTAGGTG	TCTGGCCAGTCTAGCGCCCAATCC	tag217074
novel_sir1293	TTTGCCAAGTCGGCCTCCATCC	ACGGTTCAGCCGGAGGTAGGGT	tag194106
novel_sir1294	CAGTCCTCGATGTCGTAGGAG	ACGTCAGGAGCTACAGCATCC	tag290082
novel_sir1295	GGTAGCCAGCGGGCTGCGCATCC	ATCGGTCGCCCGACGCGTAGGCT	tag170114
novel_sir1296	GGGTAGCCAGCGGGCTGCGCATCC	CATCGGTCGCCCGACGCGTAGGCT	tag103415
novel_sir1297	ATCGGGCGTCTGATCCGTAGGTG	CCTAGCCCGCAGACTAGGCATCC	tag87706
novel_sir1298	TGGTCCAGTTGTGGTGGTGCATCC	CAGGTCAACACCACCACGTAGGTG	tag53224
novel_sir1299	CACCGGCCGTCCGATCTCTAGGGT	CTGTGGCCGGCAGGCTAGAGATCC	tag234389
novel_sir1300	ATGACTCGCGCTTACTAGGCA	ACTACTGAGCGCGAATGATCC	tag208997
novel_sir1301	GTTTGCTTTCTCCTCCTGATCC	AACGAAAGAGGAGGACTAGGGG	tag218435
novel_sir1302	GTGGTTAAAGTTCTACTATCC	CCAATTTCAAGATGATAGGGT	tag254472
novel_sir1303	TAGTTTGCCGACAGTGTTCACTCC	CAAACGGCTGTCACAAGTGAGGGT	tag209831
novel_sir1304	TGGTTTGCCGACAGTGTTCACTCC	CAAACGGCTGTCACAAGTGAGGGT	tag222954
novel_sir1305	AGTTCCGCGCCATAGTACTCC	AAGGCGCGGTATCATGAGGAC	tag211910
novel_sir1306	AGCAGCCGGCACGTGGAGGAG	CATCGTCGGCCGTGCACCTCC	tag290107
novel_sir1307	CCGGCCGCTGCTTCCCCCTCC	CCGGCGACGAAGGGGGAGGCG	tag2786
novel_sir1308	CATGGCGAGTGCGGGGAGGCA	TGGTACCGCTCACGCCCCTCC	tag218107
novel_sir1309	AGCGGCGCGGCGGCGGAGGCA	CCTCGCCGCGCCGCCGCCTCC	tag20079
novel_sir1310	CCTCACCATCCAGCGGAGGGC	ACGGAGTGGTAGGTCGCCTCC	tag1460
novel_sir1311	AACGACTACAACAACGGAGGCA	ACTTGCTGATGTTGTTGCCTCC	tag216410
novel_sir1312	CGTTGCATACGTCGAAGGTCCTCC	AACGTATGCAGCTTCCAGGAGGTG	tag260079
novel_sir1313	AAGTTGTAGATCGCGTCGAGGGC	GTTTCAACATCTAGCGCAGCTCC	tag184491
novel_sir1314	TGTTTCAACATCTAGCGCAGCTCC	AAAGTTGTAGATCGCGTCGAGGGC	tag146552
novel_sir1315	TTTCAACATCCAGTGCAGCTCC	AGTTGTAGGTCACGTCGAGGGC	tag229965
novel_sir1316	CGGTGAACCGGTGCGCGCTCC	CACTTGGCCACGCGCGAGGAG	tag189844
novel_sir1317	CCGGTGAACCGGTGCGCGCTCC	CCACTTGGCCACGCGCGAGGAG	tag77403
novel_sir1318	GCTCTGCCTCGCCGCTGCTCC	AGACGGAGCGGCGACGAGGCG	tag198777
novel_sir1319	AGCTTGGATTCTGACTTAGAGGCG	GATCGAACCTAAGACTGAATCTCC	tag173224
novel_sir1320	TCGTGCCACGTAACTGATATCTCC	CACGGTGCATTGACTATAGAGGAT	tag97425
novel_sir1321	CTGGCCGTTACTAGTGATATCTCC	CCGGCAATGATCACTATAGAGGAG	tag37325
novel_sir1322	TCGGGTTGCTGAATCCTCTCC	CCCAACGACTTAGGAGAGGAT	tag210748
novel_sir1323	CACGTGACTTCGACTGCAGAGGAT	CAGTGCACTGAAGCTGACGTCTCC	tag7510
novel_sir1324	TATCAAAATGAGACCGGTCTCC	AGTTTTACTCTGGCCAGAGGTG	tag42970
novel_sir1325	CAGCCCACACAACCAGAGGTT	CTGTCGGGTGTGTTGGTCTCC	tag180162

novel_sir1326	CCGCCAGGAAGATACAGAGGGT	ACGGCGGTCCTTCTATGTCTCC	tag100583
novel_sir1327	CCGGGGTTGGTTGTTCAGGTT	CTGGCCCCAACCAACAAGTCC	tag214399
novel_sir1328	CGTCGCCGCTCGCCGCCGTCC	AGCGGCGAGCGGCGGCAGGCG	tag21695
novel_sir1329	AACGTATGCAGCTTCCAGGAG	CGTTGCATACGTCGAAGGTCC	tag45086
novel_sir1330	CCTGGCTGGGTGAAGGATAGGTCC	ACCGACCCACTTCCTATCCAGGAT	tag77462
novel_sir1331	TGGTTGCGCCGTCAAAGCTGGTCC	CAACGCGGCAGTTTCGACCAGGTC	tag62350
novel_sir1332	CCTCCGTCCCATAATATAAGGGA	AGGGAGGCAGGGTATTATATTCC	tag227649
novel_sir1333	CCGCCGCCCGTTGGGAAGGGA	CGGGCGGCGGGCAACCCTTCC	tag80194
novel_sir1334	AGTGGATCGTGGCAGCAAGGCC	TGTCACCTAGCACCGTCGTTCC	tag103761
novel_sir1335	AGTGGATCGTGGCAGCAAGGCC	AGTCACCTAGCACCGTCGTTCC	tag286716
novel_sir1336	ACTGCTACCTGCGGCTGTTCC	ACGATGGACGCCGACAAGGAT	tag282099
novel_sir1337	AAACTCCGAAGGTCTAAAGGAT	AGTTTGAGGCTTCCAGATTTCC	tag169991
novel_sir1338	TCTCCGTAAGAAAAGCAAAGC	AGGCATTCTTTTCGTTTCGAT	tag111588
novel_sir1339	TCTCCGTAAGAAAAGCAAAGC	AGGCATTCTTTTCGTTTCGAA	tag111588
novel_sir1340	CAGGCAACCATACACAATTTCGTG	TAGTCCGTTGGTATGTGTTAAAGC	tag2498
novel_sir1341	GCGTCCGGGGGGAAACGGCCAAGC	CAGGCCCCCCTTTGCCGGTTCGGC	tag2859
novel_sir1342	TGTGCCCGCTACATATGTCCAAGC	ACGGGCGATGTATACAGGTTCGGG	tag138570
novel_sir1343	TGTGCCCGCTACATCTGTCCAAGC	ACGGGCGATGTAGACAGGTTCGGG	tag165617
novel_sir1344	AAGGCTATAGGCCAGTTTGCAAGC	CCGATATCCGGTCAAACGTTCGAT	tag181605
novel_sir1345	CCGACATCCGGCCAAACGTTCGAT	AAGGCTGTAGGCCGGTTTGCAAGC	tag79398
novel_sir1346	AGGGCTATAGCTCAGTTCGGT	GTTCCCGATATCGAGTCAAGC	tag268645
novel_sir1347	AGGGCTATAGCTCAGTTCGGC	GTTCCCGATATCGAGTCAAGC	tag268645
novel_sir1348	AGGGCTATAGCTCAGTTCGGG	GTTCCCGATATCGAGTCAAGC	tag268645
novel_sir1349	ATTCCCGATATCGAGTCAAGC	AGGGCTATAGCTCAGTTCGGA	tag257946
novel_sir1350	GTTCCCGATATCGAGTCAAGC	AGGGCTATAGCTCAGTTCGGA	tag268645
novel_sir1351	AGCTCCATGGCGCAGTTCGTG	TGTCGAGGTACCGCGTCAAGC	tag219915
novel_sir1352	CTGTCGAGGTACCGCGTCAAGC	CAGCTCCATGGCGCAGTTCGTG	tag2019
novel_sir1353	GGTCCCAGCCCAACGGTCAAGC	AGGGTCGGGTTGCCAGTTCGGT	tag8565
novel_sir1354	CCGGCACACGGTAGCTTCGCG	TAGGCCGTGTGCCATCGAAGC	tag275250
novel_sir1355	AATCCGGCACACGGTAGCTTCGCG	TATTAGGCCGTGTGCCATCGAAGC	tag293831
novel_sir1356	CCGTTGGGAAGGGAGCTTCGAG	CGGGCAACCCTTCCCTCGAAGC	tag212684
novel_sir1357	CAGGACCGTTCCGTGTGAAGC	CCTGGCAAGGCACACTTCGTC	tag16664
novel_sir1358	TTGGCTCCCGCGCCGATAAGC	CCGAGGGCGCGGCTATTCGAA	tag86889
novel_sir1359	GGGGCGCAGCGTGCCTAAGC	CCGCGTCGCACGGATTCGTT	tag191970

novel_sir1360	GGGGCGCAGCGTGCCTAAGC	CCGCGTCGCACGGATTCGTG	tag191970
novel_sir1361	GGGGCGCAGCGTGCCTAAGC	CCGCGTCGCACGGATTCGTC	tag191970
novel_sir1362	GGGGCGCAGCGTGCCTAAGC	CCGCGTCGCACGGATTCGTA	tag191970
novel_sir1363	CGGGGCGCAGCGTGCCTAAGC	CCCGCGTCGCACGGATTCGTG	tag168898
novel_sir1364	CGGGGCGCAGCGTGCCTAAGC	CCCGCGTCGCACGGATTCGTA	tag168898
novel_sir1365	CGGGGCGCAGCGTGCCTAAGC	CCCGCGTCGCACGGATTCGTC	tag168898
novel_sir1366	CGGGGCGCAGCGTGCCTAAGC	CCCGCGTCGCACGGATTCGTT	tag168898
novel_sir1367	TCGGGGCGCAGCGTGCCTAAGC	CCCCGCGTCGCACGGATTCGTC	tag101814
novel_sir1368	TCGGGGCGCAGCGTGCCTAAGC	CCCCGCGTCGCACGGATTCGTA	tag101814
novel_sir1369	TCGGGGCGCAGCGTGCCTAAGC	CCCCGCGTCGCACGGATTCGTG	tag101814
novel_sir1370	TCGGGGCGCAGCGTGCCTAAGC	CCCCGCGTCGCACGGATTCGTT	tag101814
novel_sir1371	GAGCTTTTACTCAAGCTAAGC	CGAAAATGAGTTCGATTCGGA	tag252922
novel_sir1372	TCTGTCTGTCGAATAGATCTAAGC	ACAGACAGCTTATCTAGATTCGTA	tag259076
novel_sir1373	TGGTTTAGGTTGCCGACTCTAAGC	CAAATCCAACGGCTGAGATTCGTC	tag256495
novel_sir1374	AGGTATGTATACAGATTTCTAAGC	CATACATATGTCTAAAGATTCGAT	tag48263
novel_sir1375	CATGCATGTGTCCAAAGATTCGAT	AAGTACGTACACAGGTTTCTAAGC	tag156142
novel_sir1376	CATGCATATGTCCAAAGATTCGAT	AAGTACGTATACAGGTTTCTAAGC	tag80506
novel_sir1377	ATTAATCCGAGTTTTCTAAGC	ATTAGGCTCAAAAGATTCGTC	tag48815
novel_sir1378	CAGTGGTGTCCGCGGAGCGACAGC	CACCACAGGCGCCTCGCTGTCGAC	tag283060
novel_sir1379	CCGGATCCAGCTCCTGTCGGC	GAGGCCTAGGTCGAGGACAGC	tag253749
novel_sir1380	GGGCTTACTGCGGTTTCGTACAGC	CGAATGACGCCAAAGCATGTCGGA	tag288875
novel_sir1381	ACGCTGTTTGAAAGGACCAGC	CGACAAACTTTCCTGGTCGCG	tag16936
novel_sir1382	CAGTAGGCAGCAGCAAGCAGC	CATCCGTCGTCGTTCGTCGCG	tag203237
novel_sir1383	CCGCGCCTCCCCCTTCGTCGCC	CCGGCGCGGAGGGGGAAGCAGC	tag232970
novel_sir1384	CCGCATCGGCGCTGTCGTCGCT	GTGGCGTAGCCGCGACAGCAGC	tag272494
novel_sir1385	GCGGGGGCCGTCGGCAGCAGC	CCCCCGGCAGCCGTCGTCGGC	tag147968
novel_sir1386	AGGTCGAGGACAGCCGCAGC	CAGCTCCTGTCGGCGTCGGC	tag235994
novel_sir1387	TAGGTCGAGGACAGCCGCAGC	CCAGCTCCTGTCGGCGTCGGC	tag128900
novel_sir1388	AACTTTCCTGGTCGCGTCGGG	GTTTGAAAGGACCAGCGCAGC	tag179954
novel_sir1389	AATAAGAGGAGCCTACGCGTCGGG	CCTTATTCTCCTCGGATGCGCAGC	tag66163
novel_sir1390	CCAGCCGCGCCGCGCCGTCGAT	CGGGTCGGCGCGGCGCGGCAGC	tag119299
novel_sir1391	CCGTAGCTAGCCGCCGTCGGC	GCGGCATCGATCGGCGGCAGC	tag144617
novel_sir1392	CCACTTACAATGCCCCGTCGCG	ACGGTGAATGTTACGGGGCAGC	tag147987
novel_sir1393	CCACTTACAATGCCCCGTCGCG	TCGGTGAATGTTACGGGGCAGC	tag236488

novel_sir1394	ATGGACTTCTCGCGACGTCGGG	GTTACCTGAAGAGCGCTGCAGC	tag177549
novel_sir1395	CTTCCGAGGGGTGTTCTGCAGC	AGGCTCCCCACAAGACGTCGGT	tag232341
novel_sir1396	ATGGAAACAGCAACTTTAGTCGCC	ATTACCTTTGTCGTTGAAATCAGC	tag102756
novel_sir1397	AGCTGCGGCCCGGTAGTCGAG	GCTCGACGCCGGGCCATCAGC	tag172699
novel_sir1398	CCTCCGGCGAGCGGCATCAGC	AGGCCGCTCGCCGTAGTCGCC	tag12806
novel_sir1399	AGGCCGCTCGCCATAGTCGCC	CCTCCGGCGAGCGGTATCAGC	tag148622
novel_sir1400	ACGGAGCAATCGACCGTCAGC	CCTCGTTAGCTGGCAGTCGGT	tag17190
novel_sir1401	CCGTCGCGTATTTAAGTCGTC	GGGGCAGCGCATAAATTCAGC	tag165037
novel_sir1402	CCCGTCGCGTATTTAAGTCGTC	CGGGGCAGCGCATAAATTCAGC	tag193771
novel_sir1403	TCGCTGGGGGTGCCTGAAGAGC	CGACCCCCACGGACTTCTCGCC	tag100520
novel_sir1404	AGCGGAGGGCCATTCTCGGG	GGTCGCCTCCCGGTAAGAGC	tag10247
novel_sir1405	CATCACAGGTAGTCGGCACAGAGC	AGTGTCCATCAGCCGTGTCTCGTT	tag96689
novel_sir1406	ATCCATCCACTTTTAGGTCTCGTG	CGTAGGTAGGTGAAAATCCAGAGC	tag216153
novel_sir1407	ATCCATCCACTTTTAGGTCTCGTA	CGTAGGTAGGTGAAAATCCAGAGC	tag216153
novel_sir1408	ATGGAGCAATCTAAGCAGAGC	CCTCGTTAGATTCGTCTCGCA	tag195882
novel_sir1409	ACATGGATATAGTAAGTCTCGCT	AATGTACCTATATCATTCAGAGC	tag283442
novel_sir1410	GGGGTAGAGGGCTCAAGAGAGC	CCATCTCCCGAGTTCTCTCGTG	tag129003
novel_sir1411	GGGTGCGCGGCAGTGGAGAGC	CACGCGCCGTCACCTCTCGTC	tag174448
novel_sir1412	CATCCTAGTACTACTCTCGCC	GGGTAGGATCATGATGAGAGC	tag20649
novel_sir1413	CCGGGACACGGTACTGTAGAGC	CCCTGTGCCATGACATCTCGAC	tag262950
novel_sir1414	CGACGGCGGCGGCTTGCTCGGC	CCGCTGCCGCCGCCGAACGAGC	tag91437
novel_sir1415	CGTCCCTATGACGGAGGCGAGC	AGGGATACTGCCTCCGCTCGGC	tag263325
novel_sir1416	CAGGGATACTGCCTCCGCTCGGC	ACGTCCCTATGACGGAGGCGAGC	tag26668
novel_sir1417	CCTCCTGGCATCGTTCCTCGCC	AGGGAGGACCGTAGCAAGGAGC	tag243588
novel_sir1418	CAGTCGCAGCAAAGGTTCAGGAGC	CAGCGTCGTTTCCAAGTCCTCGGT	tag94538
novel_sir1419	CCAACTCTCTGCCTCCTCGCG	CGGGTTGAGAGACGGAGGAGC	tag224385
novel_sir1420	CATACGCTAATAGCTCACCTCGTT	TAGTATGCGATTATCGAGTGGAGC	tag161050
novel_sir1421	CAACTGACACGTCCTAGACTCGAA	TGGTTGACTGTGCAGGATCTGAGC	tag63163
novel_sir1422	CCCTAAACTTGCAGATCACTCGTT	CAGGGATTTGAACGTCTAGTGAGC	tag209366
novel_sir1423	CCCTAAACTTACAGATCACTCGTT	CAGGGATTTGAATGTCTAGTGAGC	tag43303
novel_sir1424	AAAAACATTGCTCAACTCGCC	GCTTTTTGTAACGAGTTGAGC	tag33900
novel_sir1425	CAAGACAAGACGTCGGATATCGAC	CGGTTCTGTTCTGCAGCCTATAGC	tag17365
novel_sir1426	CAAGACAAGACGCCGGATATCGAC	CAGTTCTGTTCTGCGGCCTATAGC	tag42831
novel_sir1427	AATGTCTTCCGCCCGGATCGGC	TGTTACAGAAGGCGGGCCTAGC	tag20506

novel_sir1428	AGAGTTGCTCCAAGGATCGAC	CCTCTCAACGAGGTTCCTAGC	tag209228
novel_sir1429	CACAACTCACCGAAAGATCGGG	TAGTGTTGAGTGGCTTTCTAGC	tag119325
novel_sir1430	CAGTTCGGTCTGAATCATCGTA	CGGTCAAGCCAGACTTAGTAGC	tag43331
novel_sir1431	ATGCTTGTGTTGCCACATTAGC	CGAACACAACGGTGTAATCGGA	tag191872
novel_sir1432	CGATGGGTTTGGAGAAAAATCGGA	TGGCTACCCAAACCTCTTTTTAGC	tag240093
novel_sir1433	TTGTGGTTCCTGGTCGAACGC	CACCAAGGACCAGCTTGCGGA	tag274969
novel_sir1434	AGTGGAGAGCAGGAGACACGC	ACCTCTCGTCCTCTGTGCGCC	tag106472
novel_sir1435	AAAGGCTAAAAACGCAGGTGCGGC	GGTTTCCGATTTTTGCGTCCACGC	tag288238
novel_sir1436	GGTAGGTGAAAATCCAGAGCACGC	ATCCACTTTTAGGTCTCGTGCGCT	tag11897
novel_sir1437	GGTAGGTGAAAATCCAGAGCACGC	ATCCACTTTTAGGTCTCGTGCGTT	tag11897
novel_sir1438	GCGGAAAAAGGGAGAGGCACGC	CCTTTTTCCCTCTCCGTGCGCG	tag147063
novel_sir1439	ACCTCTTGTAAGCCGCCGTGCGGT	TATGGAGAACATTCGGCGGCACGC	tag194558
novel_sir1440	TATGGAGAACATTCGGCGGCACGC	ACCTCTTGTAAGCCGCCGTGCGGC	tag194558
novel_sir1441	ATGTCAGGCAAGCCAGATGCGGG	GATACAGTCCGTTCGGTCTACGC	tag65579
novel_sir1442	CCCCGCGCCGCGCCATGCGTG	AAGGGGCGCGGCGCGGTACGC	tag119919
novel_sir1443	CCATCTGGCTAGTCTCTTGGCGAT	TCGGTAGACCGATCAGAGAACCGC	tag123870
novel_sir1444	CGTACCAGTAACCGACACCGC	ATGGTCATTGGCTGTGGCGAT	tag262014
novel_sir1445	AGGTACCGCGTCAAGCACCGC	CATGGCGCAGTTCGTGGCGCC	tag126723
novel_sir1446	AGTGCCGTGGAGGAGGACCGC	ACGGCACCTCCTCCTGGCGCA	tag198916
novel_sir1447	GGGGACCTCGCCATCTACCGC	CCTGGAGCGGTAGATGGCGAG	tag114709
novel_sir1448	GTTGACAAAAGCTGACCCCGC	ACTGTTTTCGACTGGGGCGGC	tag12894
novel_sir1449	ACGTGGGTCGGGGCGGGCGGC	ATTGCACCCAGCCCCGCCCGC	tag125091
novel_sir1450	AACGTGGGTCGGGGCGGGCGGC	GATTGCACCCAGCCCCGCCCGC	tag262785
novel_sir1451	CCTCTGTGCCTCCTCGCCCGC	AGACACGGAGGAGCGGGCGGC	tag132685
novel_sir1452	ACAGGGCACAGCACGGGCGGC	CGTGTCCCGTGTCGTGCCCGC	tag38752
novel_sir1453	CTTCGAGGGAAGGGTTGCCCGC	AGCTCCCTTCCCAACGGGCGGC	tag209561
novel_sir1454	CCTAGGTCGAGGACAGCCGC	ATCCAGCTCCTGTCGGCGTC	tag261996
novel_sir1455	GGTCGAGGACAGCCGCAGCCGC	AGCTCCTGTCGGCGTCGGCGGC	tag192689
novel_sir1456	CCGTGCCTCCTCGCCCGCCGC	CACGGAGGAGCGGGCGGCGGG	tag40418
novel_sir1457	CTGTGCCTCCTCGCCCGCCGC	CACGGAGGAGCGGGCGGCGGG	tag293750
novel_sir1458	GAGGACAGCCGCAGCCGCCGC	CCTGTCGGCGTCGGCGGCGGC	tag77313
novel_sir1459	AAGGACAGCCGCAGCCGCCGC	CCTGTCGGCGTCGGCGGCGGC	tag292283
novel_sir1460	AGCTCCTGTCGGCGGCGGCGCA	CGTCGAGGACAGCCGCCGCCGC	tag263782
novel_sir1461	AAGTGAGATAGCCGCCGCCGC	CACTCTATCGGCGGCGGCGGG	tag271655

novel_sir1462	GCTCGAGGCCGCCGCCGCCGC	AGCTCCGGCGGCGGCGGCGGG	tag200715
novel_sir1463	CCTCCCTCGCCGCGCCGCCGC	AGGGAGCGGCGCGGCGGCGGA	tag109433
novel_sir1464	AGGGAGCGGCACGGCGGCGGA	CCTCCCTCGCCGTGCCGCCGC	tag246486
novel_sir1465	CCTAAGTTGCCTCCTTCGCCGC	ATTCAACGGAGGAAGCGGCGGC	tag53799
novel_sir1466	CTTAGGTTCGATCGTTCGCCGC	ATCCAAGCTAGCAAGCGGCGCC	tag231590
novel_sir1467	CGGCTGGAGAGATCGAGGCCGC	CGACCTCTCTAGCTCCGGCGAG	tag228292
novel_sir1468	AGGGTGTCGGCGGCCGGCGAG	ACTCCCACAGCCGCCGGCCGC	tag204063
novel_sir1469	GCGGGGTGAGCGGCCGGCCGC	CCCCACTCGCCGGCCGGCGCC	tag222439
novel_sir1470	GCTGCCGCCGATCGATGCCGC	ACGGCGGCTAGCTACGGCGGC	tag287407
novel_sir1471	CCGCTGCCGCCTTAGCTGCCGC	CGACGGCGGAATCGACGGCGCG	tag278250
novel_sir1472	TGTACACTCTTTTTCAGTTGCCGC	ATGTGAGAAAAAGTCAACGGCGCC	tag30524
novel_sir1473	TGTACACTCTTTTTCAGTTGCCGC	ATGTGAGAAAAAGTCAACGGCGTC	tag30524
novel_sir1474	TGTACAAATTTTTTCAGTTGCCGC	ATGTTTAAAAAAGTCAACGGCGTC	tag81290
novel_sir1475	TGTACAAATTTTTTCAGTTGCCGC	ATGTTTAAAAAAGTCAACGGCGCC	tag81290
novel_sir1476	CAGTTGACGTGGCGCTGAGGCGTC	CAGTCAACTGCACCGCGACTCCGC	tag263783
novel_sir1477	TCTGCCTCGCCGCTGCTCCGC	ACGGAGCGGCGACGAGGCGCC	tag164162
novel_sir1478	GAGTGGCTGCCGCTGGTCCGC	CACCGACGGCGACCAGGCGTT	tag50642
novel_sir1479	GAGGAGGACGGTGCAGCCAAGCGC	CCTCCTGCCACGTCGGTTCGCGTG	tag238987
novel_sir1480	AGTAGGCAGCAGCAAGCAGCGC	ATCCGTCGTCGTTCGTCGCGTT	tag62733
novel_sir1481	ACTTACAATGCCCCGTCGCGTA	GGTGAATGTTACGGGGCAGCGC	tag151677
novel_sir1482	CACTTACAATGCCCCGTCGCGTA	CGGTGAATGTTACGGGGCAGCGC	tag183597
novel_sir1483	CAACTCTCTGCCTCCTCGCGCG	GGGTTGAGAGACGGAGGAGCGC	tag280624
novel_sir1484	AGGTTGAATTACTATCGCGGC	GATCCAACTTAATGATAGCGC	tag196230
novel_sir1485	AAGTGTGTTAACCAGTAGCGC	CACACAATTGGTCATCGCGCT	tag175297
novel_sir1486	TTGGCCGTTTCCCCCCGGACGCGC	CCGGCAAAGGGGGGCCTGCGCGGT	tag203564
novel_sir1487	CGTTGCTCGCCGCCTACGCGC	AACGAGCGGCGGATGCGCGGG	tag98203
novel_sir1488	GCTGCCGCCTTAGCTGCCGCGC	ACGGCGGAATCGACGGCGCGGC	tag278187
novel_sir1489	CCGCTGCTTCCCCCTCCGCGC	CGACGAAGGGGGAGGCGCGGC	tag156513
novel_sir1490	GGTGAATTCCCACTAGCGCGC	ACTTAAGGGTGATCGCGCGGT	tag9659
novel_sir1491	CTGCTCGCCACTACCGCGCGC	CGAGCGGTGATGGCGCGCGGG	tag55562
novel_sir1492	ATACATCAGTGTAGCGCGCGTG	CCTATGTAGTCACATCGCGCGC	tag179356
novel_sir1493	GATCGTACCACTCCCTGCGCGC	AGCATGGTGAGGGACGCGCGTC	tag64294
novel_sir1494	CCTCCGATCCCCCAACGGCGC	AGGCTAGGGGGTTGCCGCGGC	tag252639
novel_sir1495	GCGGAGGGGGTGTAGCGGCGC	CCTCCCCCACATCGCCGCGCC	tag261649

novel_sir1496	GCGGCGAGCGGCGGTGGGCGC	CCGCTCGCCGCCACCCGCGCC	tag48648
novel_sir1497	AGGAATAAGAGGAGCCTACGCGTC	GTTCCTTATTCTCCTCGGATGCGC	tag73816
novel_sir1498	CTTTTCGAAACGGCACTGCGC	AAAGCTTTGCCGTGACGCGTC	tag186996
novel_sir1499	CTGTGGCTGCGGCTCCTGCGC	CACCGACGCCGAGGACGCGCG	tag138593
novel_sir1500	GTTCGGCCGGGCCGGCTGCGC	AGCCGGCCCGGCCGACGCGCC	tag248739
novel_sir1501	ACCGCCGCCGCGTCACGCGCC	GGTGGCGGCGGCGCAGTGCGC	tag207616
novel_sir1502	CCTGTTCCCATTGTTAGCGCT	GAGGACAAGGGTAACAATCGC	tag163132
novel_sir1503	GAGGAAGATGACGACAATCGC	CCTTCTACTGCTGTTAGCGCT	tag113972
novel_sir1504	TGTTACCCTTGTCCTCATCGC	AATGGGAACAGGAGTAGCGCT	tag140251
novel_sir1505	TTGTTACCCTTGTCCTCATCGC	CAATGGGAACAGGAGTAGCGCT	tag98118
novel_sir1506	CCGTCGGTTCGCAAGTATCGC	CAGCCAAGCGTTCATAGCGAC	tag220440
novel_sir1507	CTGCTGCCTCCCGCGCCTCGC	CGACGGAGGGCGCGGAGCGGC	tag220167
novel_sir1508	CCTCCTCCAGCAGCCGCTCGC	AGGAGGTCGTCGGCGAGCGCG	tag118961
novel_sir1509	AATACCTCCTCTCTACCGGCTCGC	ATGGAGGAGAGATGGCCGAGCGGT	tag45437
novel_sir1510	CATACCTCCTCTCTACCGGCTCGC	ATGGAGGAGAGATGGCCGAGCGGT	tag267612
novel_sir1511	GGTACAGGACCAGCCTCTCGC	ATGTCCTGGTCGGAGAGCGGC	tag13579
novel_sir1512	CGTCAGCTCGTTCGGTCTCGC	AGTCGAGCAAGCCAGAGCGCC	tag127647
novel_sir1513	CCGTTCGCGTTCCTTTCTCGC	CAAGCGCAAGGAAAGAGCGGT	tag106293
novel_sir1514	TGTCAATGAGGTACCAGTCGC	AGTTACTCCATGGTCAGCGGT	tag34463
novel_sir1515	AGTGCTGCCAGTCGCCGTCGC	ACGACGGTCAGCGGCAGCGCG	tag284941
novel_sir1516	GGTACCGGCGGCGTCGGTCGC	ATGGCCGCCGCAGCCAGCGCC	tag180012
novel_sir1517	GGTACCGGCGGCGTCGGTCGC	ATGGCCGCCGCAGCCAGCGCT	tag180012
novel_sir1518	TTGGCACGGTTGGTGTTCGGTCGC	CCGTGCCAACCACAAGCCAGCGTG	tag112007
novel_sir1519	ACACTTCGTCGGGTACAGCGAC	AATGTGAAGCAGCCCATGTCGC	tag44416
novel_sir1520	AAGCTACTTACTATCAATTCGC	CGATGAATGATAGTTAAGCGGT	tag92274
novel_sir1521	AGTCTTGAGGCTTCAATTCGC	AGAACTCCGAAGTTAAGCGTG	tag111335
novel_sir1522	AACGGCGGCGAGGGGAAGCGGC	GATTGCCGCCGCTCCCCTTCGC	tag179542
novel_sir1523	GGTCTCGCGGACCCAGCTTCGC	AGAGCGCCTGGGTCGAAGCGGC	tag128778
novel_sir1524	TCTTAGGTTCGATCGTTCGC	AATCCAAGCTAGCAAGCGGC	tag127118
novel_sir1525	AGTCTTAGGTTCGATCGTTCGC	AGAATCCAAGCTAGCAAGCGGC	tag181121
novel_sir1526	ACGGCCGAGCCAGCAAGCGCG	GCTGCCGGCTCGGTCGTTCGC	tag268691
novel_sir1527	ATCGCTAGATCGTTATTCCGCT	GGTAGCGATCTAGCAATAAGGC	tag105560
novel_sir1528	TCGGTAGCGATCTAGCAATAAGGC	CCATCGCTAGATCGTTATTCCGCT	tag265367
novel_sir1529	CGCCGCGCCGTCGATTCCGCC	GCGCGGCGCGGCAGCTAAGGC	tag52414

novel_sir1530	ACTCCTAAATAGCAAGATTCCGGA	TATGAGGATTTATCGTTCTAAGGC	tag26004
novel_sir1531	CCCCATGCCAAATGTGTCCGAA	TCGGGGTACGGTTTACACAGGC	tag222653
novel_sir1532	AAGGCTATAGGCCAGTTTGCAGGC	CCGATATCCGGTCAAACGTCCGAT	tag176442
novel_sir1533	AAGGCTGTAGGCCAGTTTGCAGGC	CCGACATCCGGTCAAACGTCCGAC	tag2992
novel_sir1534	CCGACATCCGGTCAAACGTCCGAT	AAGGCTGTAGGCCAGTTTGCAGGC	tag2992
novel_sir1535	AGGCTCCACTGTAGCCTATCAGGC	CGAGGTGACATCGGATAGTCCGAT	tag214676
novel_sir1536	TGTGTGTAACACGGAAACTCAGGC	ACACATTGTGCCTTTGAGTCCGTG	tag268029
novel_sir1537	TGTGTGTATTACAAAACCTCAGGC	ACACATAATGTTTTGGAGTCCGTG	tag288921
novel_sir1538	TGTGTGTAACGCGAAACCTCAGGC	ACACATTGCGCTTTGGAGTCCGTG	tag285281
novel_sir1539	TATGTGTGACACAGAACCTCAGGC	ACACACTGTGTCTTGGAGTCCGTG	tag6172
novel_sir1540	TGTGTGTGACACAGAACCTCAGGC	ACACACTGTGTCTTGGAGTCCGTG	tag96654
novel_sir1541	ACACACTGTGTCTTGGAGTCCGCT	TATGTGTGACACAGAACCTCAGGC	tag6172
novel_sir1542	TATGTGTGACACAGAACCTCAGGC	ACACACTGTGTCTTGGAGTCCGTA	tag6172
novel_sir1543	TGTGTGTGACACAGAACCTCAGGC	ACACACTGTGTCTTGGAGTCCGTA	tag96654
novel_sir1544	TGTGTGTAACGCAGAACCTCAGGC	ACACATTGCGTCTTGGAGTCCGTG	tag157927
novel_sir1545	TGTGTGTAACGCAGAACCTCAGGC	ACACATTGCGTCTTGGAGTCCGCG	tag157927
novel_sir1546	ACACATTGCGTCTTGGAGTCCGTA	TGTGTGTAACGCAGAACCTCAGGC	tag157927
novel_sir1547	ACACTGCGTCTTGGAGTCCGTG	TGTGTGACGCAGAACCTCAGGC	tag252325
novel_sir1548	TCTGTGTGACGCAGAACCTCAGGC	ACACACTGCGTCTTGGAGTCCGTA	tag165917
novel_sir1549	TGTGTGTGACGCAGAACCTCAGGC	ACACACTGCGTCTTGGAGTCCGTA	tag183363
novel_sir1550	TGTGTGTAATGCAGAACCTCAGGC	ACACATTACGTCTTGGAGTCCGTA	tag174673
novel_sir1551	TGTGTGTAATGCAGAACCTCAGGC	ACACATTACGTCTTGGAGTCCGTG	tag174673
novel_sir1552	TAGTAAACGGACTAAGGCTCAGGC	CATTTGCCTGATTCCGAGTCCGTA	tag50480
novel_sir1553	ACACACTGTGTCTTAGAGTCCGTG	TGTGTGTGACACAGAATCTCAGGC	tag275108
novel_sir1554	AGGGAGATAAACAGCGGGTCAGGC	CCTCTATTTGTCGCCCAGTCCGAT	tag149124
novel_sir1555	AGCAGTCGCCTTAAGAAGTCCGCT	GATCGTCAGCGGAATTCTTCAGGC	tag229642
novel_sir1556	CCCGAGAATGGCCCTCCGCT	TGGGGCTCTTACCGGGAGGC	tag202206
novel_sir1557	CCCCGAGAATGGCCCTCCGCT	ATGGGGCTCTTACCGGGAGGC	tag268273
novel_sir1558	TATGGGGCTCTTACCGGGAGGC	ACCCCGAGAATGGCCCTCCGCT	tag86144
novel_sir1559	CCTCTAGCTTCAAACTCCGAA	GTGGAGATCGAAGTTTGAGGC	tag274511
novel_sir1560	ACCTCTAGCTTCAAACTCCGAA	TGTGGAGATCGAAGTTTGAGGC	tag188330
novel_sir1561	CCTTCAACAGCCTCTCTATCCGGT	TGGGAAGTTGTCGGAGAGATAGGC	tag40369
novel_sir1562	GCGGCTGTCCTCGACCTAGGC	CCGACAGGAGCTGGATCCGGA	tag3348
novel_sir1563	AGTGCTGGTATGATCGCATCCGAC	AATCACGACCATACTAGCGTAGGC	tag257261

novel_sir1564	CGAAAACCCCTTCCAACATCCGGT	AGGCTTTTGGGGAAGGTTGTAGGC	tag290481
novel_sir1565	AGGCGTTCAGTCATAATCCGGC	TCTCCGCAAGTCAGTATTAGGC	tag78863
novel_sir1566	CGCTTCCTCCGTTGAATCCGCC	CGGCGAAGGAGGCAACTTAGGC	tag214231
novel_sir1567	CGGCTGACGAGCTACAACGGC	CGACTGCTCGATGTTGCCGCA	tag247380
novel_sir1568	GCGCTTCGATGGCACACGGC	CGAAGCTACCGTGTGCCGGA	tag170642
novel_sir1569	CCGCGCTTCGATGGCACACGGC	CGCGAAGCTACCGTGTGCCGGA	tag265619
novel_sir1570	TGGGATGACTACTGGCACGGC	CCTACTGATGACCGTGCCGCG	tag133195
novel_sir1571	ATGGGATGACTACTGGCACGGC	CCCTACTGATGACCGTGCCGCA	tag192805
novel_sir1572	ATGGGATGACTACTGGCACGGC	CCCTACTGATGACCGTGCCGCG	tag192805
novel_sir1573	CACCCTATTGCGCCAGCTGCCGAC	CGGTGGGATAACGCGGTCGACGGC	tag216978
novel_sir1574	TTGCTCATATATCGGAACCGGC	CGAGTATATAGCCTTGGCCGAC	tag7278
novel_sir1575	CCGCTACATATGTCCAAGCCCGGC	CGATGTATACAGGTTCGGGCCGCT	tag182465
novel_sir1576	ACCATCTTTCGTCGGTCGGCCGAT	TTTGGTAGAAAGCAGCCAGCCGGC	tag212843
novel_sir1577	ACCATCTTTCGTCGGTCGGCCGAA	TTTGGTAGAAAGCAGCCAGCCGGC	tag212843
novel_sir1578	AGCTTCGAGGCGGCCGGC	CCTCGAAGCTCCGCCGGC	tag248176
novel_sir1579	AGGGAGCTTCGAGGCGGCCGGC	CTTCCCTCGAAGCTCCGCCGGC	tag153132
novel_sir1580	AGCTTCGAGGCGGCCGGCCGCG	CCTCGAAGCTCCGCCGGCCGGC	tag239002
novel_sir1581	CTGCGCGGCGCCGGCCGGC	CGCGCCGCGGCCGGCCGCC	tag15599
novel_sir1582	CCCTTAGATAGGCCGGCCGAC	ACGGGAATCTATCCGGCCGGC	tag73489
novel_sir1583	GAGGGGGAAGCAGCGGCCGGC	CCCCCTTCGTCGCCGGCCGCC	tag235967
novel_sir1584	CACCTCTCGTCGCCGGCCGTC	CGGTGGAGAGCAGCGGCCGGC	tag73914
novel_sir1585	GCGGTGGAGAGCAGCGGCCGGC	CCACCTCTCGTCGCCGGCCGTC	tag31203
novel_sir1586	CATCGTTCCTCGCCGGCCGGT	CCGTAGCAAGGAGCGGCCGGC	tag147753
novel_sir1587	AGGGAAAAAGGCGCCGGCCGGC	TCTCCCTTTTTCCGCGGCCGGC	tag128219
novel_sir1588	TGGCCGGTTCGGCCGGGCCGGC	CGGCCAAGCCGGCCCGGCCGAC	tag43223
novel_sir1589	TTGGAGTGAATCGCGGGGGCCGGC	CCTCACTTAGCGCCCCCGGCCGAA	tag259078
novel_sir1590	CTGGGTTGGGGTGGATGCCGGC	CCCAACCCCACCTACGGCCGGA	tag116692
novel_sir1591	CGACGACGGATGACGGCCGGC	TTGCTGCTGCCTACTGCCGGC	tag137489
novel_sir1592	AGGGGAGGCCCAAAGCTCCGGC	CCCTCCGGGTTTCGAGGCCGAG	tag170707
novel_sir1593	ACGACATACCGGAAAGAGGCCGTG	GGTGCTGTATGGCCTTTCTCCGGC	tag270687
novel_sir1594	CGGGAGGGCAACCGACAGCGGC	CCTCCCGTTGGCTGTCGCCGCT	tag93099
novel_sir1595	CGCCTCCCCCTTCGTCGCCGGC	GCGCGGAGGGGGAAGCAGCGGC	tag184151
novel_sir1596	GCGGCGGTGGAGAGCAGCGGC	CCGCCACCTCTCGTCGCCGGC	tag81593
novel_sir1597	TCGTTCGGCGGCGGCAGCGGC	CAAGCCGCCGCCGTCGCCGGC	tag93476

novel_sir1598	CGCCATACGCCAGTCGCCGCT	ACGCGGTATGCGGTCAGCGGC	tag94161
novel_sir1599	CCCAAAAATGCCATCGCCGTT	CAGGGTTTTTACGGTAGCGGC	tag229619
novel_sir1600	GCGCGGAGGGGGTGTAGCGGC	CGCCTCCCCCACATCGCCGCG	tag246571
novel_sir1601	CGCCGACAGGAGCTGCGCCGCC	CGGCGGCTGTCCTCGACGCGGC	tag187650
novel_sir1602	CCTCTCCCTTTTTCCGCGGC	AGAGGGAAAAAGGCGCCGGC	tag172140
novel_sir1603	CGGCCGGGCCGGCTGCGCGGC	CGGCCCGGCCGACGCGCCGCG	tag191953
novel_sir1604	AAGGAGGGAGGGGTGCGCGGC	CCTCCCTCCCCACGCGCCGTC	tag216973
novel_sir1605	CCGCGCCGTCGATTCCGCCGTC	GCGGCGCGGCAGCTAAGGCGGC	tag80301
novel_sir1606	CGGCGAGGCACAGGAGGCGGC	CGCTCCGTGTCCTCCGCCGTC	tag99391
novel_sir1607	GGGGAAGCAGCGGCCGGCGGC	CCTTCGTCGCCGGCCGCCGTA	tag249998
novel_sir1608	CGATTCCGCCGTCGCCGCCGCT	CAGCTAAGGCGGCAGCGGCGGC	tag135851
novel_sir1609	CGGCTGTCCTCGACGCGGCGGC	CGACAGGAGCTGCGCCGCCGCC	tag261872
novel_sir1610	AGGAGCTGCGCCGCCGCCGAC	TGTCCTCGACGCGGCGGCGGC	tag190239
novel_sir1611	CGGGAGCTTGTGGGCGGCGGC	CCTCGAACACCCGCCGCCGCC	tag204440
novel_sir1612	CCGCCAGCCGCGCCCGCCGTT	GCGGCGGTCGGCGCGGGCGGC	tag86780
novel_sir1613	GCGGCGGTGGGCGCGGGCGGC	CCGCCACCCGCGCCCGCCGTT	tag214831
novel_sir1614	AAAGGATTCAGCCCGCCGCC	CGTTTCCTAAGTCGGGCGGC	tag260448
novel_sir1615	CAAAGGATTCAGCCCGCCGCC	ACGTTTCCTAAGTCGGGCGGC	tag190872
novel_sir1616	CAATAAACTCTTTGGACCGCCGGT	CAGTTATTTGAGAAACCTGGCGGC	tag76108
novel_sir1617	GCGCGAGCGGGCGGCTGCGGC	CGCTCGCCCGCCGACGCCGCC	tag113264
novel_sir1618	TAGTGCTGAAGCACCAAAATCGGC	CACGACTTCGTGGTTTTAGCCGTA	tag269427
novel_sir1619	CGCCCACAGCTGCCGGAGCCGTG	AGGCGGGTGTCGACGGCCTCGGC	tag58730
novel_sir1620	CATGGACACAGCCGAGCCGCC	TCGTACCTGTGTCGGCTCGGC	tag143699
novel_sir1621	GCGGAACTCCTGCTGCTCGGC	CCTTGAGGACGACGAGCCGTA	tag287202
novel_sir1622	CGGCGGTGCGGCTCGTTCGGC	CGCCACGCCGAGCAAGCCGCC	tag153324
novel_sir1623	CGGGCACGGTGGCCGGTTCGGC	CCGTGCCACCGGCCAAGCCGGC	tag270693
novel_sir1624	CGGCTAAAGGTGTTATCAGGGC	CGATTTCCACAATAGTCCCGGA	tag195848
novel_sir1625	CCGATTTCCACAATAGTCCCGGA	CCGGCTAAAGGTGTTATCAGGGC	tag8144
novel_sir1626	CGCCGGACCTCCTCTCCCGCC	CGGCGGCCTGGAGGAGAGGGC	tag290060
novel_sir1627	CCGTTGAATCCGCCACATCCCGAG	GAGGCAACTTAGGCGGTGTAGGGC	tag91897
novel_sir1628	GATAAGCTTATCGGCACGGGC	ATTCGAATAGCCGTGCCCGCG	tag170090
novel_sir1629	CCGCGGACGCGGGCGGCGGGC	CGCCTGCGCCCGCCGCCCGCC	tag278901
novel_sir1630	AGGATTCAGCCCGCCGCCCGTT	TTTCCTAAGTCGGGCGGCGGGC	tag103893
novel_sir1631	TGTGGATGCCTAGTCTGCGGGC	ACCTACGGATCAGACGCCCGAT	tag24629

novel_sir1632	TGGCTCATGGCAGGTGCGGGC	CGAGTACCGTCCACGCCCGGT	tag9898
novel_sir1633	CCTCTGCCGTCGACCGGGGGC	AGACGGCAGCTGGCCCCCGCC	tag254411
novel_sir1634	CACCGACCGTCCGCCCCCGCG	GGGTGGCTGGCAGGCGGGGGC	tag95017
novel_sir1635	GCGGTCGGTGGCGAACTGGGC	CCAGCCACCGCTTGACCCGCG	tag69756
novel_sir1636	GCGCTCCGTCGGCTTCTGGGC	CGAGGCAGCCGAAGACCCGCA	tag191519
novel_sir1637	CGGCGATGCCGGGAGCTTGTGGGC	CGCTACGGCCCTCGAACACCCGCC	tag136050
novel_sir1638	CCTGGGAATCCTCATCCATTGGGC	ACCCTTAGGAGTAGGTAACCCGTT	tag142639
novel_sir1639	ACTGGGAATCCTCATCCATTGGGC	ACCCTTAGGAGTAGGTAACCCGTT	tag281581
novel_sir1640	ACCCTTAGGAGTAGGTAACCCGTA	ACTGGGAATCCTCATCCATTGGGC	tag281581
novel_sir1641	TCGCCTGAGGCGCAATCGAATGGC	CGGACTCCGCGTTAGCTTACCGAA	tag67537
novel_sir1642	CACCTGCCAAGCTGTTGTACCGTG	GGGTGGACGGTTCGACAACATGGC	tag196270
novel_sir1643	CATGACATCTCGACTACCGGG	CGGTACTGTAGAGCTGATGGC	tag177888
novel_sir1644	ACGGTACTGTAGAGCTGATGGC	CCATGACATCTCGACTACCGGG	tag146040
novel_sir1645	CAGGATGTACAGTAACTCTATGGC	CCTACATGTCATTGAGATACCGAT	tag41170
novel_sir1646	TAGGATGTACAGTAACTCTATGGC	CCTACATGTCATTGAGATACCGAT	tag203630
novel_sir1647	CACCACCTGCTGCTTGACCGAC	CGGTGGTGGACGACGAACTGGC	tag227091
novel_sir1648	ACGGCGACTGGCAGCACTGGC	CCGCTGACCGTCGTGACCGAC	tag22273
novel_sir1649	GGTGACTTTCTGGAGCCTACTGGC	ACTGAAAGACCTCGGATGACCGGT	tag62002
novel_sir1650	GTGCGTGCACTGGCCCCTGGC	CGCACGTGACCGGGGACCGGA	tag34808
novel_sir1651	CAGCTAAACGCTCAAATCCCTGGC	CGATTTGCGAGTTTAGGGACCGGA	tag280797
novel_sir1652	TGGCGATGTTCAAATTCCTGGC	CGCTACAAGTTTAAGGACCGCC	tag293307
novel_sir1653	AGCCTACGCGTCGGGCGACCGAT	CCTCGGATGCGCAGCCCGCTGGC	tag202647
novel_sir1654	CGCGCGTGTCGGCTAGACCGTC	TCGCGCGCACAGCCGATCTGGC	tag32094
novel_sir1655	AGCGCGCGTGTCGGCTAGACCGTC	CGTCGCGCGCACAGCCGATCTGGC	tag159780
novel_sir1656	TGGCTTGACTTTTCTGACTCTGGC	CGAACTGAAAAGACTGAGACCGAG	tag136648
novel_sir1657	CGAACCGAAAAGACTGAGACCGAG	TGGCTTGGCTTTTCTGACTCTGGC	tag201481
novel_sir1658	CGAACCGAAAAGACTGAGACCGAA	TGGCTTGGCTTTTCTGACTCTGGC	tag201481
novel_sir1659	CAGTTTCAACACAAAACCTCTGGC	CAAAGTTGTGTTTTGGAGACCGTT	tag139128
novel_sir1660	CAGTTTCAACACAAAACCTCTGGC	CAAAGTTGTGTTTTGGAGACCGTG	tag139128
novel_sir1661	CAAAATAGCATCTCGGAGACCGTG	CAGTTTTATCGTAGAGCCTCTGGC	tag45558
novel_sir1662	ACCCACGTAAGTTTAGAGACCGCC	TGTGGGTGCATTCAAATCTCTGGC	tag287031
novel_sir1663	CCGGATGCTACTAAGTCTGGC	CCTACGATGATTCAGACCGAA	tag248511
novel_sir1664	CAGTTATATCGTAGAGCTTCTGGC	CAATATAGCATCTCGAAGACCGTG	tag39630
novel_sir1665	GGTTCCCGGGCACGGTGGC	AAGGGCCCGTGCCACCGGC	tag109119

novel_sir1666	GGGTTCCCGGGCACGGTGGC	CAAGGGCCCGTGCCACCGGC	tag125060
novel_sir1667	GGGGTTCCCGGGCACGGTGGC	CCAAGGGCCCGTGCCACCGGC	tag137271
novel_sir1668	GGGGGTTCCCGGGCACGGTGGC	CCCAAGGGCCCGTGCCACCGGC	tag138838
novel_sir1669	CGGGGGTTCCCGGGCACGGTGGC	CCCCAAGGGCCCGTGCCACCGGC	tag214976
novel_sir1670	CGCGATCTTACGGTGCCCACCGGC	AAGCGCTAGAATGCCACGGGTGGC	tag291235
novel_sir1671	GTGTACGTGGGTGTTAGATGTGGC	CATGCACCCACAATCTACACCGTT	tag153238
novel_sir1672	GCTGACGGACACCGTACAATTGGC	ACTGCCTGTGGCATGTTAACCGGG	tag288760
novel_sir1673	CGGTGAACCGCCGGTTGAACCGCG	TGGCCACTTGGCGGCCAACTTGGC	tag53767
novel_sir1674	TTGGATCATGCTCTCCTTGGC	CCTAGTACGAGAGGAACCGTT	tag1264
novel_sir1675	GCGTTGTCATCGAGCGCTTGGC	CAACAGTAGCTCGCGAACCGGT	tag253394
novel_sir1676	CGAGACAGTCCCAACTAAACCGAT	TGGCTCTGTCAGGGTTGATTTGGC	tag13158
novel_sir1677	CCGCGGAGCGACAGCTGCCCATGC	CGCCTCGCTGTCGACGGGTACGTC	tag289012
novel_sir1678	GCGGCTGCCGCCGATCGATGC	CCGACGGCGGCTAGCTACGGC	tag38568
novel_sir1679	CGCCTCGCTGTCGACGGATACGTC	CCGCGGAGCGACAGCTGCCTATGC	tag166094
novel_sir1680	CTTCCGGACACACGTATCCTATGC	AGGCCTGTGTGCATAGGATACGTT	tag202357
novel_sir1681	GCTTATGCTCTGCCTTGCTTATGC	AATACGAGACGGAACGAATACGGT	tag61870
novel_sir1682	CGCCGCGATGGCCTTGACGGC	TCGCGGCGCTACCGGAACTGC	tag286267
novel_sir1683	GCGCGGAACTCCATGAACTGC	CGCCTTGAGGTACTTGACGTC	tag264052
novel_sir1684	TAGTCACTGAAGATCGGACACTGC	CAGTGACTTCTAGCCTGTGACGGA	tag121923
novel_sir1685	AATAGCTTTTCGAAACGGCACTGC	ATCGAAAAGCTTTGCCGTGACGCG	tag129344
novel_sir1686	GGGCTGGCCTTGATCGGGCACTGC	CGACCGGAACTAGCCCGTGACGCT	tag20281
novel_sir1687	TGGCTGGCCTTGATCGGGCACTGC	CGACCGGAACTAGCCCGTGACGCT	tag61307
novel_sir1688	AGAGAATTGGTGAGTGACGCA	GGTCTCTTAACCACTCACTGC	tag86316
novel_sir1689	CAGAGAATTGGTGAGTGACGCA	TGGTCTCTTAACCACTCACTGC	tag67885
novel_sir1690	TGGCCCAGAAACAGGATGTACTGC	CGGGTCTTTGTCCTACATGACGCT	tag111116
novel_sir1691	CAGACGAAGCAGGTGGACGCC	TCGTCTGCTTCGTCCACCTGC	tag164237
novel_sir1692	CAGTGCAGTTACGGTGCACCCTGC	CACGTCAATGCCACGTGGGACGGA	tag116777
novel_sir1693	CCGGCTGCCAACTCCCCCTGC	CCGACGGTTGAGGGGGACGAC	tag181401
novel_sir1694	GCTGATCGTACCACTCCCTGC	ACTAGCATGGTGAGGGACGCG	tag91249
novel_sir1695	CGCGAAGGCTTGCTCGGACGGG	ACGCGCTTCCGAACGAGCCTGC	tag236144
novel_sir1696	GCGCTGCCGCCGGCCGCCTGC	CGACGGCGGCCGGCGGACGGC	tag205591
novel_sir1697	ACCATGATCGGGATAGCGGACGTC	ATTGGTACTAGCCCTATCGCCTGC	tag163065
novel_sir1698	ACCATGATCGGGATAGCGGACGTC	TTTGGTACTAGCCCTATCGCCTGC	tag167757
novel_sir1699	ATTGGTACTAGCCCTATCGCCTGC	ACCATGATCGGGATAGCGGACGTA	tag163065

novel_sir1700	TTTGGTACTAGCCCTATCGCCTGC	ACCATGATCGGGATAGCGGACGTA	tag167757
novel_sir1701	TGGGACTTGAAAGTTATGGCCTGC	CCTGAACTTTCAATACCGGACGAA	tag236147
novel_sir1702	TGGTGCTTGAAATTTATGGCCTGC	CACGAACTTTAAATACCGGACGAA	tag91529
novel_sir1703	CCCCAACTATGTAAACCGGACGAA	TGGGGGTTGATACATTTGGCCTGC	tag187486
novel_sir1704	GTGCGCACAACCAGCTCCTGC	CGCGTGTTGGTCGAGGACGGA	tag8057
novel_sir1705	CACTCCACAGGCATCCTCGACGGC	TGGTGAGGTGTCCGTAGGAGCTGC	tag79643
novel_sir1706	CTGCCTACTGCCGGCCGCTGC	CGGATGACGGCCGGCGACGAG	tag210252
novel_sir1707	TCTCCTCCCGCCGCGGCGCTGC	AGGAGGGCGGCGCCGCGACGGC	tag126469
novel_sir1708	TCGGTTCGCAAGTATCGCTGC	CCAAGCGTTCATAGCGACGTT	tag146775
novel_sir1709	CCGGTTCGGCCGGGCCGGCTGC	CCAAGCCGGCCCGGCCGACGCG	tag285222
novel_sir1710	AGCTGCGCCGCCGCCGACGCC	CCTCGACGCGGCGGCGGCTGC	tag95615
novel_sir1711	TGGTTGCGCTGCTTGCTGCTGC	CAACGCGACGAACGACGACGGA	tag152987
novel_sir1712	GCGGCGAGCCGGATCTGCTGC	CCGCTCGGCCTAGACGACGCC	tag68523
novel_sir1713	CCGGTCGGACAGAGCCTCTGC	CCAGCCTGTCTCGGAGACGAC	tag79943
novel_sir1714	TGGGGATCCGATAAAACGCTCTGC	CCCTAGGCTATTTTGCGAGACGAA	tag4210
novel_sir1715	CCGCCGTTCGACTGCACCGTCTGC	CGGCAAGCTGACGTGGCAGACGGC	tag275698
novel_sir1716	TAGTCTAACGACGATGTCTGC	CAGATTGCTGCTACAGACGCA	tag226595
novel_sir1717	CGATAGGCCACGCTTTCACGGT	TAGCTATCCGGTGCGAAAGTGC	tag214270
novel_sir1718	CCACCGCCGCCGCGTCACGCG	GCGGTGGCGGCGGCGCAGTGC	tag177325
novel_sir1719	GGTCCTGTTGAGGCCCTAGTGC	AGGACAACTCCGGGATCACGCT	tag204117
novel_sir1720	TGGCGGCGCCGGTAGACGTGC	CGCCGCGGCCATCTGCACGAC	tag234026
novel_sir1721	CTGCCTGTCAGTTTGCAACCGTGC	CGGACAGTCAAACGTTGGCACGGG	tag234962
novel_sir1722	CGGCTTGGCCGGTGGCACGGG	CGGCCGAACCGGCCACCGTGC	tag27025
novel_sir1723	CCGGCTTGGCCGGTGGCACGGG	CCGGCCGAACCGGCCACCGTGC	tag73623
novel_sir1724	CGTAGCTGGTGATGCTCGTGC	ATCGACCACTACGAGCACGCC	tag87877
novel_sir1725	CATCGACCACTACGAGCACGCC	ACGTAGCTGGTGATGCTCGTGC	tag135397
novel_sir1726	ACTACTCTCGCCCAAGCACGCT	CATGATGAGAGCGGGTTCGTGC	tag207722
novel_sir1727	CGGTCGACATCATGCCTGAGGTGC	CAGCTGTAGTACGGACTCCACGAC	tag203807
novel_sir1728	CATGCTAGTCGGCGCCACGAA	TGGTACGATCAGCCGCGGTGC	tag63844
novel_sir1729	TGGCACGATCGGCCGCGGTGC	CGTGCTAGCCGGCGCCACGAA	tag102485
novel_sir1730	CAGTATCTGTGTACACCTCGGTGC	CATAGACACATGTGGAGCCACGTG	tag270242
novel_sir1731	ACGACGACGGTGGCCCACGCT	GCTGCTGCTGCCACCGGGTGC	tag112351
novel_sir1732	ACTCCCCAGCGACCCCCACGGA	CCTGAGGGGTCGCTGGGGGTGC	tag36734
novel_sir1733	CGGCGGGCGGGGCTGGGTGC	CGCCCGCCCCGACCCACGTT	tag100064

novel_sir1734	GCGGCGGGCGGGGCTGGGTGC	CCGCCCGCCCCGACCCACGTT	tag111665
novel_sir1735	GTGCACGGTGTATGGTGGTGC	CGTGCCACATACCACCACGGT	tag285403
novel_sir1736	CATGTGCCTCAATACCACCACGGT	TGGTACACGGAGTTATGGTGGTGC	tag93573
novel_sir1737	CATGGACGATAGCGATGACACGGA	CAGTACCTGCTATCGCTACTGTGC	tag111373
novel_sir1738	CTGCCTGTCAGTTTCAACCTGTGC	CGGACAGTCAAAGTTGGACACGGA	tag172129
novel_sir1739	CAGTCATAATCCGGCACACGGT	TAGTCAGTATTAGGCCGTGTGC	tag282423
novel_sir1740	CAGTCATAATCCGGCACACGGT	AAGTCAGTATTAGGCCGTGTGC	tag286440
novel_sir1741	GATCGCCAGGAATTTGAACATTGC	AGCGGTCCTTAAACTTGTAACGAA	tag247654
novel_sir1742	GATCGCCAGGAATTTGAACATTGC	AGCGGTCCTTAAACTTGTAACGAT	tag247654
novel_sir1743	GATCGCCAGGAATTTGAACATTGC	AGCGGTCCTTAAACTTGTAACGAG	tag247654
novel_sir1744	AGGAGGAGGTGCCGTGAACGAC	GGTCCTCCTCCACGGCACTTGC	tag18313
novel_sir1745	AGCAACGTCGCTATGAACGCT	TTTCGTTGCAGCGATACTTGC	tag71973
novel_sir1746	AAGCAACGTCGCTATGAACGCT	TTTTCGTTGCAGCGATACTTGC	tag181951
novel_sir1747	TGGTCGAAGACGGTATCACCTTGC	CAGCTTCTGCCATAGTGGAACGGA	tag138400
novel_sir1748	GTTTGTACAAATTTTTTCAGTTGC	AACATGTTTAAAAAAGTCAACGGC	tag246575
novel_sir1749	GTTTGTACAAATTTTTTCAGTTGC	AACATGTTTAAAAAAGTCAACGGT	tag246575
novel_sir1750	CAGTGCTACTGCAGTAGTTGC	CACGATGACGTCATCAACGGC	tag185802
novel_sir1751	ATCTTGGAACACATCAACGGT	CTTAGAACCTTGTGTAGTTGC	tag28260
novel_sir1752	GTGCTTAGAACCTTGTGTAGTTGC	CGAATCTTGGAACACATCAACGGT	tag126755
novel_sir1753	AGGCACTGGAACGGCAACGGC	GGTCCGTGACCTTGCCGTTGC	tag270371
novel_sir1754	GAGCTTCGAGGGAAGGGTTGC	CGAAGCTCCCTTCCCAACGGG	tag29569
novel_sir1755	CCTGACACAGCCTGACAACGTG	CCGGACTGTGTCGGACTGTTGC	tag238087
novel_sir1756	AAACTTCCGTGGCCTAAACGGC	AGTTTGAAGGCACCGGATTTGC	tag57741
novel_sir1757	ACTTGTTCAACCTCCTGGATTTGC	AACAAGTTGGAGGACCTAAACGAG	tag273111
novel_sir1758	CGGCGTCAAACATTTCGAAACGGA	TTGCCGCAGTTTGTAAAGCTTTGC	tag192289
novel_sir1759	AATCTACTGTACGTTCTTTGC	AGATGACATGCAAGAAACGGG	tag99933
novel_sir1760	GGGGAAGGCTGTAGGCCAGTTTGC	CCTTCCGACATCCGGTCAAACGTC	tag236908
novel_sir1761	TGGGAAGGCTGTAGGCCAGTTTGC	CCTTCCGACATCCGGTCAAACGTC	tag239401
novel_sir1762	CCTTCCGACATCCGGTCAAACGTT	GGGGAAGGCTGTAGGCCAGTTTGC	tag236908
novel_sir1763	CCTTCCGACATCCGGTCAAACGTT	TGGGAAGGCTGTAGGCCAGTTTGC	tag239401
novel_sir1764	GGGGAGTTGATTAAACGTTTGC	CCTCAACTAATTTGCAAACGGA	tag132037
novel_sir1765	ACTTTGAATCGGTTCGTCAAAATC	AAACTTAGCCAAGCAGTTTTAGCT	tag250698
novel_sir1766	ACTGTAGCACGACACATTTTAGTA	TATGACATCGTGCTGTGTAAAATC	tag228135
novel_sir1767	CAGGGACAGTGTAGCCTACAAATC	CCCTGTCACATCGGATGTTTAGAC	tag164497

novel_sir1768	ATGGCATGCATGTGGAGTTTAGTA	CCTACCGTACGTACACCTCAAATC	tag203134
novel_sir1769	TTGCCTAGATGCTAATAAATC	CGGATCTACGATTATTTAGTT	tag55714
novel_sir1770	CGGGAGGCCTTAGAACGATAAATC	CCTCCGGAATCTTGCTATTTAGGA	tag203626
novel_sir1771	CGGGAAGCCTTAGAATGATAAATC	CCTTCGGAATCTTACTATTTAGGA	tag36828
novel_sir1772	CGGGAGGCCTTAGAATGATAAATC	CCTCCGGAATCTTACTATTTAGGA	tag62544
novel_sir1773	AACGGCGTCAAACATTTAGGG	AGTTGCCGCAGTTTGTAAATC	tag22254
novel_sir1774	CGGGCGGGGCTGGGTGCAATC	CCGCCCCGACCCACGTTAGGG	tag76661
novel_sir1775	GCGGGCGGGGCTGGGTGCAATC	CCCGCCCCGACCCACGTTAGGG	tag266346
novel_sir1776	TGGCTATAACGTCCCCTCAATC	CGATATTGCAGGGGAGTTAGTA	tag89668
novel_sir1777	TCGGCGAACAAACAAATCAGAATC	CCGCTTGTTTGTTTAGTCTTAGAC	tag88691
novel_sir1778	ATTAGCCAAACGCTGCCTTAGGT	GGTAATCGGTTTGCGACGGAATC	tag4637
novel_sir1779	CATTAGCCAAACGCTGCCTTAGGT	GGGTAATCGGTTTGCGACGGAATC	tag128083
novel_sir1780	AGCTTGGATTCTGACTTAGAG	GATCGAACCTAAGACTGAATC	tag240248
novel_sir1781	TGGATTTCAACACCGAGGCTAATC	CTAAAGTTGTGGCTCCGATTAGAC	tag245171
novel_sir1782	ACCGCAAAAGAATGGTAATTAGCT	TGTGGCGTTTTCTTACCATTAATC	tag253547
novel_sir1783	ATCTTTTGAGCCTAATTAGTC	CTTAGAAAACTCGGATTAATC	tag224502
novel_sir1784	GTTCTGCAGCCATGACACATC	AGACGTCGGTACTGTGTAGCA	tag179345
novel_sir1785	TAGCCATAGCAAAGCCGAACCATC	CGGTATCGTTTCGGCTTGGTAGGA	tag215782
novel_sir1786	TAGCCATAGCAAAGCCGAACCATC	CGGTATCGTTTCGGCTTGGTAGCA	tag215782
novel_sir1787	AGGCCGGGCCGATGGGTAGGG	CGTCCGGCCCGGCTACCCATC	tag273155
novel_sir1788	TTGCCGACAGTGTTCACTCCCATC	CGGCTGTCACAAGTGAGGGTAGAC	tag52570
novel_sir1789	TCTGTACACCTTCATGCCATC	ACATGTGGAAGTACGGTAGTG	tag40753
novel_sir1790	CATAATCCGGCACACGGTAGCT	CAGTATTAGGCCGTGTGCCATC	tag253189
novel_sir1791	TTTGCCAAGTCGGCCTCCATC	ACGGTTCAGCCGGAGGTAGGG	tag159415
novel_sir1792	CTTTGCCAAGTCGGCCTCCATC	AACGGTTCAGCCGGAGGTAGGG	tag146396
novel_sir1793	GGGTAGCCAGCGGGCTGCGCATC	CATCGGTCGCCCGACGCGTAGGC	tag226757
novel_sir1794	CTAAGTCAACATCGCCACGTAGGC	CTGATTCAGTTGTAGCGGTGCATC	tag22526
novel_sir1795	CTGGGCCAGTTATGGCGGTGCATC	CCCGGTCAATACCGCCACGTAGGT	tag267869
novel_sir1796	CTGGGCCAGTTATGGCGGTGCATC	CCCGGTCAATACCGCCACGTAGGC	tag267869
novel_sir1797	CCGGTCAAGACCGCCACGTAGGC	TGGGCCAGTTCTGGCGGTGCATC	tag22733
novel_sir1798	CTGGATCAGTTTGGTCGGTGCATC	CCTAGTCAAACCAGCCACGTAGGC	tag140315
novel_sir1799	TCGCACGAACCCGCTCTCATC	CGTGCTTGGGCGAGAGTAGTA	tag24061
novel_sir1800	CGCGGCACGGTCATCAGTAGGG	TAGCGCCGTGCCAGTAGTCATC	tag257410
novel_sir1801	TCTTATACAGTGTAGGCTAAGATC	AATATGTCACATCCGATTCTAGAT	tag204239

novel_sir1802	AATATTACATTCAGTAAGATC	ATAATGTAAGTCATTCTAGTA	tag288735
novel_sir1803	ACAATGTAAGTCATTCTAGCA	AGTGTTACATTCAGTAAGATC	tag38282
novel_sir1804	CGTTGCAATCACTCCAGTCTAGGG	CCGCAACGTTAGTGAGGTCAGATC	tag215297
novel_sir1805	CAGCCAGCCGAAAGTCTAGTT	GAGTCGGTCGGCTTTCAGATC	tag50185
novel_sir1806	GTTGAATATTCAGCTTTTCAGATC	ACTTATAAGTCGAAAAGTCTAGGC	tag273912
novel_sir1807	AGGCCGGCCGACCTCTCTAGCT	TATCCGGCCGGCTGGAGAGATC	tag76340
novel_sir1808	TGGTGTGTACGAAGTTCACCGATC	CACACATGCTTCAAGTGGCTAGGA	tag33195
novel_sir1809	CTGGAGCGGCTGCCGCCGATC	CCTCGCCGACGGCGGCTAGCT	tag239170
novel_sir1810	CGTCAAGCACCGCGGCCGATC	AGTTCGTGGCGCCGGCTAGCA	tag93529
novel_sir1811	CGCCGGCCGCCGTAGCTAGCC	CAGCGGCCGGCGGCATCGATC	tag182759
novel_sir1812	GATTCAGTCTTAGGTTCGATC	AAGTCAGAATCCAAGCTAGCA	tag122310
novel_sir1813	GAGATTCAGTCTTAGGTTCGATC	CTAAGTCAGAATCCAAGCTAGCA	tag69545
novel_sir1814	ACGTCCTATCCAGTCCTAGGT	TTTGCAGGATAGGTCAGGATC	tag185092
novel_sir1815	AGGTGCAAGCGAGAGTACCGGATC	CACGTTCGCTCTCATGGCCTAGAA	tag22166
novel_sir1816	AGGTGTAAGCGAGAGTGCCGGATC	CACATTCGCTCTCACGGCCTAGAA	tag95940
novel_sir1817	ACTAGTCAAGTACTCCGGATC	ATCAGTTCATGAGGCCTAGGG	tag253905
novel_sir1818	AACGGTTCCTCTCGTACTAGGT	AGTTGCCAAGGAGAGCATGATC	tag23703
novel_sir1819	AACGAAAGAGGAGGACTAGGG	GTTTGCTTTCTCCTCCTGATC	tag183560
novel_sir1820	CGTCAAGCACCGCGGCTGATC	AGTTCGTGGCGCCGACTAGCA	tag163852
novel_sir1821	AGCTCCGGTGCCGCACTAGGC	GCTCGAGGCCACGGCGTGATC	tag120390
novel_sir1822	GCTCGAGGCCGCGGCGTGATC	AGCTCCGGCGCCGCACTAGGC	tag71374
novel_sir1823	TTGGTAAACCGATCAGAGAATATC	CCATTTGGCTAGTCTCTTATAGAT	tag18589
novel_sir1824	TCGCCACAACCGTACCTGAATATC	CGGTGTTGGCATGGACTTATAGAC	tag120375
novel_sir1825	TAGCTTAGAAACTGTATACATATC	CGAATCTTTGACATATGTATAGAA	tag118507
novel_sir1826	CGATACCTTTAATGCAATATAGGT	TGGCTATGGAAATTACGTTATATC	tag116281
novel_sir1827	AGGCAGCGCCGAAAGTTGATAGTG	TGTCCGTCGCGGCTTTCAACTATC	tag14440
novel_sir1828	ACCGAGAGTGCTAGCTGGATAGTG	TGTGGCTCTCACGATCGACCTATC	tag189906
novel_sir1829	AGGCTTCGAACTCAGGTCATAGTC	ACTCCGAAGCTTGAGTCCAGTATC	tag93095
novel_sir1830	TCTTGTCCTTGTTCTTGTATC	AACAGGAACAAGAACATAGCG	tag26370
novel_sir1831	CGGCACCTTTAAGTAATAATAGGT	TGGCCGTGGAAATTCATTATTATC	tag276716
novel_sir1832	TAGGCTGTAACAAGGAACTTTATC	CCGACATTGTTCCTTGAAATAGAT	tag142833
novel_sir1833	CCTCTACCGCTAGGTCAAAAACTC	AGATGGCGATCCAGTTTTTGAGTG	tag3644
novel_sir1834	CGTTGGTGAGTATCGTTGAGCT	GCGCAACCACTCATAGCAACTC	tag271590
novel_sir1835	CGTTTGCAAATTAGTTGAGGG	AGGCAAACGTTTAATCAACTC	tag199596

novel_sir1836	TTGTAACTACTCATCACACTC	CATTGATGAGTAGTGTGAGAC	tag72321
novel_sir1837	AGATGGAAAACGATGTGAGAG	ACTCTACCTTTTGCTACACTC	tag194111
novel_sir1838	CTGCTTACCAGTTTGTACACTC	CGAATGGTCAAACATGTGAGAA	tag256534
novel_sir1839	CCGCGGCTGATCGTACCACTC	CGCCGACTAGCATGGTGAGGG	tag155620
novel_sir1840	GTGGCGGCTGGGTGGGCACTC	CCGCCGACCCACCCGTGAGAG	tag77385
novel_sir1841	CGACGTACCTGTCAACAGTGAGGC	CCGCTGCATGGACAGTTGTCACTC	tag205444
novel_sir1842	GGGTTTGTCAGGTGCCGAAGACTC	CAAACAGTCCACGGCTTCTGAGAA	tag214529
novel_sir1843	ATGTGTCACGTCGTAGTATGAGGT	CATACACAGTGCAGCATCATACTC	tag44888
novel_sir1844	TGGCCAGGAATTAACAGTACTC	CGGTCCTTAATTGTCATGAGCA	tag140620
novel_sir1845	CGGTCCTTAATTGCCATGAGCA	TGGCCAGGAATTAACGGTACTC	tag103
novel_sir1846	AGGTGTACGTACGGTAGGGTACTC	CACATGCATGCCATCCCATGAGGT	tag141141
novel_sir1847	ACTACACCGTTCACTTATTTACTC	ATGTGGCAAGTGAATAAATGAGGA	tag63177
novel_sir1848	CGGGCGACCGATGGGTTTGGAGAC	CAGCCCGCTGGCTACCCAAACCTC	tag74204
novel_sir1849	CGGGCGACCGATGGGTTTGGAGAA	CAGCCCGCTGGCTACCCAAACCTC	tag74204
novel_sir1850	CGATTTAGATAGGCTGTTGGAGAT	AGGCTAAATCTATCCGACAACCTC	tag215962
novel_sir1851	ATACACACACTGCGTCTTGGAGTC	AGTATGTGTGTGACGCAGAACCTC	tag192770
novel_sir1852	TCGGTAAATCGATCAGAGAACCTC	CCATTTAGCTAGTCTCTTGGAGAT	tag281273
novel_sir1853	CCATTTAGCCAGTCTCTTGGAGAT	TCGGTAAATCGGTCAGAGAACCTC	tag122423
novel_sir1854	AAGTTGAATCTCTCAGTGAACCTC	CAACTTAGAGAGTCACTTGGAGAG	tag110147
novel_sir1855	TGGATTTTCACCTACCTACACCTC	CTAAAAGTGGATGGATGTGGAGAC	tag205125
novel_sir1856	TATTCGATTTTATCACTCCACCTC	AAGCTAAAATAGTGAGGTGGAGGA	tag176690
novel_sir1857	TTGGTACCGCTCACGCCCCTC	CCATGGCGAGTGCGGGGAGGC	tag210774
novel_sir1858	CGTCTGGCTATAACGTCCCCTC	AGACCGATATTGCAGGGGAGTT	tag209108
novel_sir1859	GGTCCTTAGCTTGCGCCCTC	AGGAATCGAACGCGGGAGTG	tag11019
novel_sir1860	CGAGGAAGGATGCCGGGAGGG	CCGCTCCTTCCTACGGCCCTC	tag232604
novel_sir1861	CTTCTACTTATCACGGTCCCTC	AGATGAATAGTGCCAGGGAGGC	tag236078
novel_sir1862	CGCCCGTTGGGAAGGGAGCT	CGGCGGGCAACCCTTCCCTC	tag122103
novel_sir1863	CCGCCCGTTGGGAAGGGAGCT	GCGGCGGGCAACCCTTCCCTC	tag68204
novel_sir1864	AGCACGCTTAACTTCGGAGTT	GTTCGTGCGAATTGAAGCCTC	tag115384
novel_sir1865	CCAAGCACGCTTAACTTCGGAGTT	CGGGTTCGTGCGAATTGAAGCCTC	tag20326
novel_sir1866	TGGATTTTCACCTACCTACGCCTC	CTAAAAGTGGATGGATGCGGAGAC	tag159968
novel_sir1867	ACAGGAGCTGGATCCGGAGCT	GCTGTCCTCGACCTAGGCCTC	tag256945
novel_sir1868	AAGGCGGGTGTCGGCGGCCTC	CCGCCCACAGCCGCCGGAGCC	tag234190
novel_sir1869	TGGGACCTTTGCCAAGTCGGCCTC	CCTGGAAACGGTTCAGCCGGAGGT	tag263149

novel_sir1870	CCTACACCGCCTAAGTTGCCTC	ATGTGGCGGATTCAACGGAGGA	tag119409
novel_sir1871	CAGTTTATAAAACTTTGCCTC	CAAATATTTTGAAACGGAGGG	tag97751
novel_sir1872	AGTTGATTAAACGTTTGCCTC	AACTAATTTGCAAACGGAGCT	tag229678
novel_sir1873	TAGTCGGGTTGCTGAATCCTC	CAGCCCAACGACTTAGGAGAG	tag31702
novel_sir1874	TCGATTTTATCACTCCACCTCCTC	CTAAAATAGTGAGGTGGAGGAGAG	tag280267
novel_sir1875	CCGCTGCCGGCTCACCTCCTC	CGACGGCCGAGTGGAGGAGGA	tag34676
novel_sir1876	CCGCTGCCGGCTCGCCTCCTC	CGACGGCCGAGCGGAGGAGGA	tag204669
novel_sir1877	GCGCTTGGCTGCACCGTCCTCCTC	CGAACCGACGTGGCAGGAGGAGGG	tag86850
novel_sir1878	GGTGAACCGGTGCGCGCTCCTC	ACTTGGCCACGCGCGAGGAGGC	tag121194
novel_sir1879	CGACAGCCGAGCAGAGGAGGA	CCGCTGTCGGCTCGTCTCCTC	tag111153
novel_sir1880	TCGCTGCTGGCTCGTCTCCTC	CGACGACCGAGCAGAGGAGGA	tag63026
novel_sir1881	CAGCCGATTGATCACCTCGTCCTC	CGGCTAACTAGTGGAGCAGGAGGT	tag236871
novel_sir1882	CGTTGCATACGTCGAAGGTCCTC	AACGTATGCAGCTTCCAGGAGGT	tag92458
novel_sir1883	CCGTTGCATACGTCGAAGGTCCTC	CAACGTATGCAGCTTCCAGGAGGT	tag159326
novel_sir1884	CCGCTCCTTGCTACGGTCCTC	CGAGGAACGATGCCAGGAGGG	tag171657
novel_sir1885	CGCCGACGCCGACAGGAGCT	CGGCGGCTGCGGCTGTCCTC	tag85753
novel_sir1886	GCGGCGGCTGCGGCTGTCCTC	CCGCCGACGCCGACAGGAGCT	tag259038
novel_sir1887	GCGATTGTTACCCTTGTCCTC	CTAACAATGGGAACAGGAGTA	tag91420
novel_sir1888	ATGTTTCAACATCCAGCGCAGCTC	CAAAGTTGTAGGTCGCGTCGAGGG	tag79809
novel_sir1889	CAAAGTTGTAGGTCGCGTCGAGCG	ATGTTTCAACATCCAGCGCAGCTC	tag79809
novel_sir1890	AGATCACTAAGCAGTCGAGCA	CCTCTAGTGATTCGTCAGCTC	tag149606
novel_sir1891	GCTCACTCTGGCCGCCCGCTC	AGTGAGACCGGCGGGCGAGCG	tag210020
novel_sir1892	GCGGCGGGGGCTGCAGCGCTC	CCGCCCCCGACGTCGCGAGAA	tag157944
novel_sir1893	CCGGTGAACCGGTGCGCGCTC	CCACTTGGCCACGCGCGAGGA	tag229003
novel_sir1894	AGGGACGTAGTCAACGCGAGCT	CGTCCCTGCATCAGTTGCGCTC	tag77114
novel_sir1895	CCGCTGCAAGGGTAGCTATCGCTC	CGACGTTCCCATCGATAGCGAGGC	tag227403
novel_sir1896	GCGTACCGCTGAATCGTATCGCTC	CATGGCGACTTAGCATAGCGAGAG	tag134064
novel_sir1897	CCGCTGCATGGACAGTCGTCGCTC	CGACGTACCTGTCAGCAGCGAGAC	tag38929
novel_sir1898	CGACGTACCCGTCGACAGCGAGGC	CCGCTGCATGGGCAGCTGTCGCTC	tag159180
novel_sir1899	CCGCTGCATGGGTAGCTGTCGCTC	CGACGTACCCATCGACAGCGAGGC	tag225975
novel_sir1900	AATGCAAGAAGCATCCGAGAA	GATTACGTTCTTCGTAGGCTC	tag285165
novel_sir1901	AAGCAAACGGGGTAGAGGGCTC	CGTTTGCCCCATCTCCCGAGTT	tag293043
novel_sir1902	ATGGCAGGACACCGTACGAGGC	GCTACCGTCCTGTGGCATGCTC	tag50127
novel_sir1903	CCGGCCGCGTGTCTCCTGCTC	CCGGCGCACAGAGGACGAGCG	tag27350

novel_sir1904	ACGCCTTGAGGACGACGAGCC	CCTGCGGAACTCCTGCTGCTC	tag108920
novel_sir1905	GCGCGTACTTACCTAATTGCTC	CGCATGAATGGATTAACGAGAT	tag248141
novel_sir1906	CTGGCTGACGGTCGATTGCTC	CCGACTGCCAGCTAACGAGGC	tag121991
novel_sir1907	TCGTCAGCTGACCCCTTGCTC	CAGTCGACTGGGGAACGAGAC	tag247652
novel_sir1908	CGTGCGCGCGGAAAACGAGAG	TCGCACGCGCGCCTTTTGCTC	tag174301
novel_sir1909	CAGTTGCCGCAGTTCATAAATCTC	CAACGGCGTCAAGTATTTAGAGAC	tag262037
novel_sir1910	AGGGACTCGGGCCTTAGAGCC	GCTCCCTGAGCCCGGAATCTC	tag287595
novel_sir1911	CCGCTGGAGCGGCCTCGATCTC	CGACCTCGCCGGAGCTAGAGAG	tag31712
novel_sir1912	ACATGTCATCAACTCAATAGAGTG	GGTGTACAGTAGTTGAGTTATCTC	tag247129
novel_sir1913	CGACGAGAGACTGGGAGAGGG	CTGCTGCTCTCTGACCCTCTC	tag20360
novel_sir1914	CGTGCCGCCGGCTGCCCTCTC	ACGGCGGCCGACGGGAGAGGA	tag235722
novel_sir1915	AGTCGGGTTGCTGAATCCTCTC	AGCCCAACGACTTAGGAGAGGA	tag38041
novel_sir1916	CGACGTGGGAGAAGGAGAGGG	CCGCTGCACCCTCTTCCTCTC	tag102350
novel_sir1917	GGTGCGCGCTCCTCCGTCTCTC	ACGCGCGAGGAGGCAGAGAGTT	tag238801
novel_sir1918	CGTCCGGTTGTTGATAGTCTC	AGGCCAACAACTATCAGAGAA	tag155264
novel_sir1919	AGAGATGGAAGGGCAGAGCC	GTTCTCTACCTTCCCGTCTC	tag229890
novel_sir1920	GTTCTCTACCTTCCCGTCTC	AGAGATGGAAGGGCAGAGCA	tag229890
novel_sir1921	AGAGATGGAAGGGCAGAGCT	GTTCTCTACCTTCCCGTCTC	tag229890
novel_sir1922	CCAAGAGATGGAAGGGCAGAGCC	TAGGTTCTCTACCTTCCCGTCTC	tag186515
novel_sir1923	CCGGTGCGCGCTCCTCCGTCTC	CCACGCGCGAGGAGGCAGAGAG	tag168149
novel_sir1924	GCGTCGCTGCTGGCTCGTCTC	CAGCGACGACCGAGCAGAGGA	tag94063
novel_sir1925	CTTCGGGTTCGGTCTAGGTCTC	AGCCCAAGCCAGATCCAGAGGC	tag143044
novel_sir1926	ATGGCCGCCGCAGCCAGAGCC	GGTACCGGCGGCGTCGGTCTC	tag90525
novel_sir1927	TAGTGCCCTGTAAAGTGTCTC	CACGGGACATTTCACAGAGAA	tag200746
novel_sir1928	CGGTGGTGCTGTATGGCCTTTCTC	CACCACGACATACCGGAAAGAGGC	tag207123
novel_sir1929	AAGGCCTACGATGATTCAGAC	GGTTCCGGATGCTACTAAGTC	tag103457
novel_sir1930	TCGCCAGGAATTTGAACAGTC	CGGTCCTTAAACTTGTCAGCA	tag239852
novel_sir1931	CCGCCAGGAATTTGAACAGTC	CGGTCCTTAAACTTGTCAGCA	tag270503
novel_sir1932	TGGTCGCCAGGAATTTGAACAGTC	CAGCGGTCCTTAAACTTGTCAGCA	tag47069
novel_sir1933	CGGTCGCCAGGAATTTGAACAGTC	CAGCGGTCCTTAAACTTGTCAGCA	tag193579
novel_sir1934	TAGCTTGCAAACTGGCTTACAGTC	CGAACGTTTGACCGAATGTCAGAA	tag186930
novel_sir1935	CGGTGCATCCGCGGTGCAGTC	CACGTAGGCGCCACGTCAGCC	tag182601
novel_sir1936	CCTGTTGTCGACTACGAGTCAGAT	TGGGACAACAGCTGATGCTCAGTC	tag187385
novel_sir1937	TGGGACAACAGCTGATGCTCAGTC	CCTGTTGTCGACTACGAGTCAGAC	tag187385

novel_sir1938	CTGACTTGCGGAGATTCAGTC	CTGAACGCCTCTAAGTCAGAA	tag137148
novel_sir1939	GCTCGTTACATGGGTTCAGTC	AGCAATGTACCCAAGTCAGTG	tag159092
novel_sir1940	CGGTTTGTACAAATCTTTTCAGTC	CAAACATGTTTAGAAAAGTCAGGG	tag185589
novel_sir1941	GGGAGGAAGGTTTTCAGAGTC	CTCCTTCCAAAAGTCTCAGTC	tag223946
novel_sir1942	ACGCGGGGCTGGATCTCAGTG	GCTGCGCCCCGACCTAGAGTC	tag31541
novel_sir1943	CCTAGCCGCGTCGGACGAGTC	ATCGGCGCAGCCTGCTCAGGC	tag114600
novel_sir1944	TCTGGCCTCGATAGTTGGTGAGTC	ACCGGAGCTATCAACCACTCAGCC	tag35887
novel_sir1945	GGTTAAAAATGTGGATGCCTAGTC	AATTTTTACACCTACGGATCAGAC	tag68182
novel_sir1946	ATCGCGGCACGGTCATCAGTA	GATAGCGCCGTGCCAGTAGTC	tag122690
novel_sir1947	GATCATTCGCGCTCAGTAGTC	AGTAAGCGCGAGTCATCAGCT	tag7833
novel_sir1948	GTGATTTCGTGGCCTAGGGTAGTC	CTAAAGCACCGGATCCCATCAGAA	tag81896
novel_sir1949	CTAATTTGACTAGCTAAATCAGAT	TAGATTAAACTGATCGATTTAGTC	tag172408
novel_sir1950	CCGCCCGACTTAGGAAACGTC	CGGGCTGAATCCTTTGCAGAC	tag139779
novel_sir1951	CCTAGCCTCCGGTACCACGTC	ATCGGAGGCCATGGTGCAGCC	tag101219
novel_sir1952	CCTAGCCTCCGGTACCACGTC	ATCGGAGGCCATGGTGCAGTC	tag101219
novel_sir1953	ATGCAGAACCTCAGGTACGACGTC	CGTCTTGGAGTCCATGCTGCAGCT	tag139472
novel_sir1954	CAGCACTCCGTTGCATACGTC	CGTGAGGCAACGTATGCAGCT	tag288992
novel_sir1955	AGGGTGCCATGTTGTCGAACCGTC	CCACGGTACAACAGCTTGGCAGGT	tag118305
novel_sir1956	ATCTCAGTGGATCGTGGCAGCA	CCTAGAGTCACCTAGCACCGTC	tag84121
novel_sir1957	ATGCACCGCATGCGCACCGTC	CGTGGCGTACGCGTGGCAGTC	tag142520
novel_sir1958	CTGCCCCGTAACATTCACCGTC	CGGGGCATTGTAAGTGGCAGAG	tag155384
novel_sir1959	CGTGGCCGGCGGCTGGCAGGG	CCGCACCGGCCGCCGACCGTC	tag6399
novel_sir1960	CGGGTGGGCGCGGCAGCCGTC	CCACCCGCGCCGTCGGCAGCC	tag59703
novel_sir1961	CGGGAGATGCCGAACGCCGTC	CCTCTACGGCTTGCGGCAGGC	tag74027
novel_sir1962	CGATGGATCCAAACGGCAGAT	CTGCTACCTAGGTTTGCCGTC	tag211885
novel_sir1963	CTGCCTATATGGGTAATCCGTC	CGGATATACCCATTAGGCAGGG	tag205263
novel_sir1964	GAGGCGACCTACCACTCCGTC	CCGCTGGATGGTGAGGCAGAA	tag78115
novel_sir1965	CCGCCGGCCGCCGTCGCAGCG	CAGGCGGCCGGCGGCAGCGTC	tag110803
novel_sir1966	AGGCCCTGCCTCGCCCGCGTC	CGGGACGGAGCGGGCGCAGCG	tag133150
novel_sir1967	ACGTTGCGGATGGTAGGCGTC	CAACGCCTACCATCCGCAGCC	tag116481
novel_sir1968	CATCTCGACTACCGGGCCGCAGCT	CTGTAGAGCTGATGGCCCGGCGTC	tag90538
novel_sir1969	CATCGTGCCTGAGGTTCTGCGTC	AGCACGGACTCCAAGACGCAGTG	tag43657
novel_sir1970	CGTGGCCTCGAGCGGTCGTGCGTC	ACCGGAGCTCGCCAGCACGCAGGA	tag137471
novel_sir1971	CTTCGTTTACTACTGATCGTC	AGCAAATGATGACTAGCAGGA	tag7078

novel_sir1972	TGGCTGCTGCCTCGTGGTCGTC	CGACGACGGAGCACCAGCAGGC	tag58158
novel_sir1973	CGGCTCCTCTAGTGATTCGTC	CGAGGAGATCACTAAGCAGTC	tag168712
novel_sir1974	CAGATTCGGTTTTGTTTGTTCGTC	CTAAGCCAAAACAAACAAGCAGCT	tag292935
novel_sir1975	ACTCCGTTGCATACGTCGAAGGTC	AGGCAACGTATGCAGCTTCCAGGA	tag177439
novel_sir1976	CCTCCTACTATAGTTTGTCCAGAT	GGGGAGGATGATATCAAACAGGTC	tag263234
novel_sir1977	AACAAACAGATTTAGTGTCCAGGA	CTTTGTTTGTCTAAATCACAGGTC	tag49878
novel_sir1978	AGGTGGTGGCCCTGTCCAGCT	CGTCCACCACCGGGACAGGTC	tag257212
novel_sir1979	AAAGCATAACAAGGTCCAGTT	GTTTTCGTATTGTTCCAGGTC	tag279862
novel_sir1980	CGTACGCGTGGCAGTCCAGTC	CTGCATGCGCACCGTCAGGTC	tag142344
novel_sir1981	TGGGTCGGCTGGTAGTCAGAGGTC	CCAGCCGACCATCAGTCTCCAGAT	tag46681
novel_sir1982	CCGCGTGGTCGAGGCCTAGGTC	CGCACCAGCTCCGGATCCAGCT	tag283029
novel_sir1983	GGTGCTAGGTGACTCTAGGTC	ACGATCCACTGAGATCCAGCC	tag268341
novel_sir1984	AGTGGTACACCTCTGGCCAGAG	TGTCACCATGTGGAGACCGGTC	tag173973
novel_sir1985	CCGCGCGTCGTCCGCCCCCGGTC	CGCGCAGCAGGCGGGGGCCAGCT	tag171268
novel_sir1986	CCGGCATCGCCGCCGTCGGTC	CCGTAGCGGCGGCAGCCAGCG	tag265756
novel_sir1987	CGTGCCAACCACAAGCCAGCG	TGGCACGGTTGGTGTTCGGTC	tag276211
novel_sir1988	CCGTGCCAACCACAAGCCAGCG	TTGGCACGGTTGGTGTTCGGTC	tag10835
novel_sir1989	ATGCAACACGAGGACTTCCCAGGA	CCTACGTTGTGCTCCTGAAGGGTC	tag28769
novel_sir1990	ACTTCATTCTTCAGAGGGTC	AAGTAAGAAGTCTCCCAGAT	tag40783
novel_sir1991	CTCACGACGGTCTAATCCCAGCT	CAGAGTGCTGCCAGATTAGGGTC	tag270716
novel_sir1992	GGGGCCGCCGGAAGAGGGGTC	CCGGCGGCCTTCTCCCCAGCC	tag212476
novel_sir1993	CTGCAAACCGAATATGGGGTC	CGTTTGGCTTATACCCCAGTT	tag25848
novel_sir1994	CCGAGTGGCTAACGTTGGGTC	CTCACCGATTGCAACCCAGCT	tag29933
novel_sir1995	CACGACGGTCTAAACCCAGCT	GAGTGCTGCCAGATTTGGGTC	tag263685
novel_sir1996	CTCACGACGGTCTAAACCCAGCT	CAGAGTGCTGCCAGATTTGGGTC	tag134768
novel_sir1997	CTACATCAACACCAAGGACCAGCT	TCGATGTAGTTGTGGTTCCTGGTC	tag23880
novel_sir1998	CGACACGTGTAAAGGAGACCAGGT	TTGCTGTGCACATTTCCTCTGGTC	tag44607
novel_sir1999	CCTCCTCCAGATGCACGTGGTC	AGGAGGTCTACGTGCACCAGCC	tag152207
novel_sir2000	CACAACTGTAGTAGTACACCAGAC	CAGTGTTGACATCATCATGTGGTC	tag271361
novel_sir2001	AAAGCCACTTATACACCAGTC	CTTTTCGGTGAATATGTGGTC	tag86115
novel_sir2002	ACCGCACCGACGTTTACAGCT	CTTGGCGTGGCTGCAAATGTC	tag174869
novel_sir2003	CCGTGTGAAGCAGCCCATGTC	CACACTTCGTCGGGTACAGCG	tag113258
novel_sir2004	ACGCAGAACCTCAGGCACGATGTC	CGTCTTGGAGTCCGTGCTACAGCT	tag120664
novel_sir2005	ACGCGGAACCTCAGGCACGATGTC	CGCCTTGGAGTCCGTGCTACAGAT	tag226916

novel_sir2006	CGTGTTAGATAGTGTTTTGATGTC	ACAATCTATCACAAAACTACAGAT	tag196991
novel_sir2007	TAGTAAAAAGTAGGATCCACTGTC	CATTTTTCATCCTAGGTGACAGCT	tag237465
novel_sir2008	CGTGTGAGCAAGCCAGGCACTGTC	ACACTCGTTCGGTCCGTGACAGGT	tag113528
novel_sir2009	TAGGAGACTGCACCAGTGACTGTC	CCTCTGACGTGGTCACTGACAGGT	tag38483
novel_sir2010	CACTCACTCCCTACACGGACAGCT	GGGTGAGTGAGGGATGTGCCTGTC	tag107321
novel_sir2011	CGCGTCGTGGCTGTCGACAGTC	CGGCGCAGCACCGACAGCTGTC	tag136851
novel_sir2012	AGGGCCGTAGCGGCGACAGCC	GCTCCCGGCATCGCCGCTGTC	tag181072
novel_sir2013	CGCGCTCGGGACGGCGACAGCC	GGGCGCGAGCCCTGCCGCTGTC	tag158211
novel_sir2014	CCGCCGCCGACGCCGACAGGA	GCGGCGGCGGCTGCGGCTGTC	tag238853
novel_sir2015	GTGTGATTAAAGAAAAGTGTC	CACTAATTTCTTTTCACAGAG	tag259397
novel_sir2016	CCTCCTTCGCCGCCGACGTGTC	AGGAAGCGGCGGCTGCACAGGG	tag255546
novel_sir2017	CCCGGTTAACATGCCACAGGC	CCGGGCCAATTGTACGGTGTC	tag102613
novel_sir2018	CCACGTAGGCAAGACCACAGTC	ACGGTGCATCCGTTCTGGTGTC	tag89070
novel_sir2019	TCGAGTACCGTTCAGTGTGTC	CTCATGGCAAGTCACACAGAT	tag275811
novel_sir2020	CATATTTCATCTTGGCACACAGAC	CAGTATAAAGTAGAACCGTGTGTC	tag121738
novel_sir2021	CATATTTCATCTTGGCACACAGAT	CAGTATAAAGTAGAACCGTGTGTC	tag121738
novel_sir2022	TGTTCTTGTATCGCGTGTGTC	AAGAACATAGCGCACACAGTA	tag289165
novel_sir2023	CTTGTCCTCATCGCGATTGTC	ACAGGAGTAGCGCTAACAGCA	tag39230
novel_sir2024	TCTAGCGCCCAATCCACCGTTGTC	ATCGCGGGTTAGGTGGCAACAGGC	tag147089
novel_sir2025	GTGCCAATTACAGAGCGTTGTC	CGGTTAATGTCTCGCAACAGTA	tag52903
novel_sir2026	CACGGGCCGCCAGCGTAAACAGGT	CAGTGCCCGGCGGTCGCATTTGTC	tag27283
novel_sir2027	AGGAGCAGGATCCACTTTGTC	CTCGTCCTAGGTGAAACAGCC	tag199811
novel_sir2028	TAGTCACAGCCGAAAAAAAAATTC	CAGTGTCGGCTTTTTTTTTAAGAA	tag160764
novel_sir2029	CGGTCGACGTCGTGCCTGAAATTC	CAGCTGCAGCACGGACTTTAAGAC	tag217187
novel_sir2030	CGGTCGACGTCGTGCCTGAAATTC	CAGCTGCAGCACGGACTTTAAGAT	tag217187
novel_sir2031	ATGCCCCGTCGCGTATTTAAGTC	GTTACGGGGCAGCGCATAAATTC	tag37914
novel_sir2032	AATGCCCCGTCGCGTATTTAAGTC	TGTTACGGGGCAGCGCATAAATTC	tag43245
novel_sir2033	AGGCACGACCCGGCCAGTTAAGGC	CCTCCGTGCTGGGCCGGTCAATTC	tag250965
novel_sir2034	GTGGCGCGCTAGTGGGAATTC	CCGCGCGATCACCCTTAAGTG	tag220087
novel_sir2035	ATGCGCTGCCCCGTAACATTC	CGCGACGGGGCATTGTAAGTG	tag119126
novel_sir2036	ATGCGCTGCCCCGTAACATTC	CGCGACGGGGCATTGTAAGTT	tag119126
novel_sir2037	CTCCGTTTCACAATGTAAGTC	AGGAGGCAAAGTGTTACATTC	tag139885
novel_sir2038	CCGTATTGAGGTCGAGATCCATTC	CATAACTCCAGCTCTAGGTAAGGT	tag145450
novel_sir2039	AGGAGGTGTATTCGAATATCATTC	CTCCACATAAGCTTATAGTAAGTT	tag56444

novel_sir2040	CGACAACGTAAGTGTGAGTAAGGT	TCGCTGTTGCATTCACACTCATTC	tag166722
novel_sir2041	AATACTGACTTGCGGAGATTC	ATGACTGAACGCCTCTAAGTC	tag143810
novel_sir2042	AATACTGACTTGCGGAGATTC	ATGACTGAACGCCTCTAAGTT	tag143810
novel_sir2043	CAGCTGCAGTACGGACTCTAAGAC	TGGTCGACGTCATGCCTGAGATTC	tag15332
novel_sir2044	TGGTCGACGTCATGCCTGAGATTC	CAGCTGCAGTACGGACTCTAAGAT	tag15332
novel_sir2045	CGGTCGACGTCATGCCTGAGATTC	CAGCTGCAGTACGGACTCTAAGAT	tag177210
novel_sir2046	CGGTCGACATCGTGCCTGAGATTC	CAGCTGTAGCACGGACTCTAAGAT	tag188116
novel_sir2047	TGGTCGACATCGTGCCTGAGATTC	CAGCTGTAGCACGGACTCTAAGAT	tag270475
novel_sir2048	CGGTCGACATCGTGCCTGAGATTC	CAGCTGTAGCACGGACTCTAAGAC	tag188116
novel_sir2049	TGGTCGACATCGTGCCTGAGATTC	CAGCTGTAGCACGGACTCTAAGAC	tag270475
novel_sir2050	CAACTGTAGCACGGACTCTAAGAC	CGGTTGACATCGTGCCTGAGATTC	tag228441
novel_sir2051	CGGTCGACGTCGTGCCTGAGATTC	CAGCTGCAGCACGGACTCTAAGAT	tag56611
novel_sir2052	CGGTCGACGTCGTGCCTGAGATTC	CAGCTGCAGCACGGACTCTAAGAC	tag56611
novel_sir2053	CCTCTCTATCCGGTTGGCTAAGCC	TCGGAGAGATAGGCCAACCGATTC	tag93855
novel_sir2054	CTTAATTAGAAGCTCACCCGATTC	ATTAATCTTCGAGTGGGCTAAGGC	tag134294
novel_sir2055	TGTCTACACCGTATAGGATTC	AGATGTGGCATATCCTAAGAC	tag204093
novel_sir2056	TGTTGTTTCACGAACTGTTGATTC	AACAAAGTGCTTGACAACTAAGAG	tag189512
novel_sir2057	CCGAATCTGAAAAGCTGAATATTC	CTTAGACTTTTCGACTTATAAGTT	tag216734
novel_sir2058	CAACTGCAGTACGGACTATAAGAT	CGGTTGACGTCATGCCTGATATTC	tag105103
novel_sir2059	ACTTGAACTAGTGGCATATAAGGG	TTTGAACTTGATCACCGTATATTC	tag187202
novel_sir2060	AGACGGTCTAGTACCAGATAAGAC	TGTCTGCCAGATCATGGTCTATTC	tag105872
novel_sir2061	TGGCCCCACTGTGTTGACTTATTC	CGGGGTGACACAACTGAATAAGTT	tag179588
novel_sir2062	ATGTGGCAAGTGAATAAATAAGGA	ACTACACCGTTCACTTATTTATTC	tag66975
novel_sir2063	TAGGTTGATCAGTGCGTTTTATTC	CCAACTAGTCACGCAAAATAAGAT	tag288505
novel_sir2064	AGTCTGACAAAGTGAGCACTTC	AGACTGTTTCACTCGTGAAGTT	tag249775
novel_sir2065	AGGCTAAATTTCTTCGATCACTTC	CGATTTAAAGAAGCTAGTGAAGAT	tag236937
novel_sir2066	AGTTACGGTGCAGTCTACTTC	AATGCCACGTCAGATGAAGAC	tag119438
novel_sir2067	CTTGGGCGTAGCAATCGAACCTTC	ACCCGCATCGTTAGCTTGGAAGGC	tag36207
novel_sir2068	GTTCGTATAAACCACTGCACCTTC	AGCATATTTGGTGACGTGGAAGAG	tag288404
novel_sir2069	CGTACCGCGCCGCGCCCCTTC	ATGGCGCGGCGCGGGGAAGGG	tag23815
novel_sir2070	AAGCAACTCCTTCCAAGCCTTC	CGTTGAGGAAGGTTCGGAAGTG	tag82310
novel_sir2071	TTTGCAAACTGACCTACAGCCTTC	ACGTTTGACTGGATGTCGGAAGGA	tag2004
novel_sir2072	CTTGCAAACTGACCTACAGCCTTC	ACGTTTGACTGGATGTCGGAAGGA	tag5132
novel_sir2073	TTTGCAAACTGACCTACAGCCTTC	ACGTTTGACTGGATGTCGGAAGGG	tag2004

novel_sir2074	CTTGCAAACTGACCTACAGCCTTC	ACGTTTGACTGGATGTCGGAAGGG	tag5132
novel_sir2075	ACGTTTGGTCGGATGTCGGAAGGG	CCTGCAAACCAGCCTACAGCCTTC	tag228660
novel_sir2076	ATGTTTAATCGGATGTCGGAAGGG	TCTACAAATTAGCCTACAGCCTTC	tag9289
novel_sir2077	CCTGCAAACTGGCCTACAGCCTTC	ACGTTTGACCGGATGTCGGAAGGG	tag68620
novel_sir2078	TTTGCAAACTGGCCTACAGCCTTC	ACGTTTGACCGGATGTCGGAAGGG	tag79791
novel_sir2079	TCTGCAAACTGGCCTACAGCCTTC	ACGTTTGACCGGATGTCGGAAGGG	tag100484
novel_sir2080	CTTGCAAACTGGCCTACAGCCTTC	ACGTTTGACCGGATGTCGGAAGGG	tag193233
novel_sir2081	CCTGCAAACTGGCCTACAGCCTTC	ACGTTTGACCGGATGTCGGAAGGA	tag68620
novel_sir2082	TTTGCAAACTGGCCTACAGCCTTC	ACGTTTGACCGGATGTCGGAAGGA	tag79791
novel_sir2083	TCTGCAAACTGGCCTACAGCCTTC	ACGTTTGACCGGATGTCGGAAGGA	tag100484
novel_sir2084	CTTGCAAACTGGCCTACAGCCTTC	ACGTTTGACCGGATGTCGGAAGGA	tag193233
novel_sir2085	ACGTTTAACCGGATGTCGGAAGGA	TCTGCAAATTGGCCTACAGCCTTC	tag237627
novel_sir2086	AAAGTTGATAGGATGTCGGAAGGG	GCTTTCAACTATCCTACAGCCTTC	tag212264
novel_sir2087	CTTACAAATTAGTCTACAGCCTTC	ATGTTTAATCAGATGTCGGAAGGG	tag99133
novel_sir2088	CCTGCAAACTGGTCTACAGCCTTC	ACGTTTGACCAGATGTCGGAAGGG	tag103110
novel_sir2089	GCGCCGGCCGGCGGAGCTTC	CGGCCGGCCGCCTCGAAGCT	tag36155
novel_sir2090	CGGCGCCGGCCGGCGGAGCTTC	CGCGGCCGGCCGCCTCGAAGCT	tag223429
novel_sir2091	CTTTTCGGTCACCGCGCTTC	AAAGCCAGTGGCGCGAAGCT	tag271282
novel_sir2092	ACTTTTCGGTCACCGCGCTTC	AAAAGCCAGTGGCGCGAAGCT	tag233809
novel_sir2093	AAGAATTCCGCTGACGATCGCTTC	CTTAAGGCGACTGCTAGCGAAGAC	tag66982
novel_sir2094	TCTGTGGTCAGCCGCCGCTGCTTC	ACACCAGTCGGCGGCGACGAAGGC	tag27137
novel_sir2095	GATGCCGCCGGCCGCTGCTTC	ACGGCGGCCGGCGACGAAGGG	tag27399
novel_sir2096	CATCGCGATTGTCGTCATCTTC	AGCGCTAACAGCAGTAGAAGGA	tag45642
novel_sir2097	AGCCACACTTCAGCCATAGAAGAG	CTTCGGTGTGAAGTCGGTATCTTC	tag21855
novel_sir2098	ATTGTCGTCATCTTCCTCTTC	ACAGCAGTAGAAGGAGAAGAT	tag60503
novel_sir2099	CGTTCCACTATGGCAGAAGCT	AGGCAAGGTGATACCGTCTTC	tag18788
novel_sir2100	CTCCGTTCCACTATGGCAGAAGCT	CGGAGGCAAGGTGATACCGTCTTC	tag269509
novel_sir2101	CTTCACTCCGCTACGGCAGAAGCT	CGGAAGTGAGGCGATGCCGTCTTC	tag40070
novel_sir2102	CAGCTGTAGCACGGACTTCAAGAT	TGGTCGACATCGTGCCTGAAGTTC	tag275838
novel_sir2103	CGGTCGACATCGTGCCTGAAGTTC	CAGCTGTAGCACGGACTTCAAGAC	tag19926
novel_sir2104	TGGTCGACATCGTGCCTGAAGTTC	CAGCTGTAGCACGGACTTCAAGAC	tag275838
novel_sir2105	CAGTCGACGTCGTGCCTGAAGTTC	CAGCTGCAGCACGGACTTCAAGAT	tag59393
novel_sir2106	CGGTCGACGTCGTGCCTGAAGTTC	CAGCTGCAGCACGGACTTCAAGAT	tag154429
novel_sir2107	CAGCTGCAGCACGGACTTCAAGAC	CGGTCGACGTCGTGCCTGAAGTTC	tag154429

novel_sir2108	CGGTCGATGTCGTGCCTGAAGTTC	CAGCTACAGCACGGACTTCAAGAC	tag7578
novel_sir2109	CAGCTGCAGTAAGGACTTCAAGAT	CGGTCGACGTCATTCCTGAAGTTC	tag32022
novel_sir2110	CGGTCGACGTCGTGCTTGAAGTTC	CAGCTGCAGCACGAACTTCAAGAC	tag160142
novel_sir2111	ACTGGGCCGAGCCTCTACTAGTTC	ACCCGGCTCGGAGATGATCAAGTG	tag11636
novel_sir2112	CACTCCACAAACATGCAAGTT	TAGTGAGGTGTTTGTACGTTC	tag103840
novel_sir2113	CAGTACCCCAATGCAAGGG	TCGTCATGGGGTTACGTTC	tag70130
novel_sir2114	AATGGTTTCTATGTCAGGCAAGCC	CTTTACCAAAGATACAGTCCGTTC	tag160109
novel_sir2115	AGTCAATCCGCTTTAGCGTTC	AGTTAGGCGAAATCGCAAGTT	tag279640
novel_sir2116	GCTGGGTGCAATCCCCGCGTTC	ACCCACGTTAGGGGCGCAAGCC	tag284158
novel_sir2117	AATTGGTGAGTGACGCAAGAG	TCTTAACCACTCACTGCGTTC	tag116617
novel_sir2118	AGTCTTAGGTTCGATCGTTC	AGAATCCAAGCTAGCAAGCG	tag269531
novel_sir2119	CAGTCTTAGGTTCGATCGTTC	CAGAATCCAAGCTAGCAAGCG	tag132999
novel_sir2120	CTGCTACATGGACAGCTATCGTTC	CGATGTACCTGTCGATAGCAAGAC	tag246162
novel_sir2121	CGGCGGCGGTGCGGCTCGTTC	CGCCGCCACGCCGAGCAAGCC	tag19849
novel_sir2122	GCGGCGGCGGTGCGGCTCGTTC	CCGCCGCCACGCCGAGCAAGCC	tag145214
novel_sir2123	AGTGGATCGTGGCAGCAAGGC	AGTCACCTAGCACCGTCGTTC	tag21205
novel_sir2124	CAGTGGATCGTGGCAGCAAGGC	GAGTCACCTAGCACCGTCGTTC	tag41563
novel_sir2125	CAGTGGATCGTGGCAGCAAGGC	TAGTCACCTAGCACCGTCGTTC	tag74085
novel_sir2126	CCGCTGCCGGCTCGGTCGTTC	CGACGGCCGAGCCAGCAAGCG	tag198937
novel_sir2127	CGACATCGACACCAGCAAGAG	CCGCTGTAGCTGTGGTCGTTC	tag283384
novel_sir2128	TTTGAGCAGTCGCAGCAAAGGTTC	ACTCGTCAGCGTCGTTTCCAAGTC	tag279189
novel_sir2129	CGGTCGACGTCGTGCCTAAGGTTC	CAGCTGCAGCACGGATTCCAAGAC	tag240286
novel_sir2130	CAGCTACAGCACGGATTCCAAGAC	CGGTCGATGTCGTGCCTAAGGTTC	tag148901
novel_sir2131	CAGCTGCAGCATGGACTCCAAGAC	CGGTCGACGTCGTACCTGAGGTTC	tag130658
novel_sir2132	CAGCTGTAGTACGGACTCCAAGAC	CAGTCGACATCATGCCTGAGGTTC	tag32414
novel_sir2133	CGGTCGACGTCATGCCTGAGGTTC	CAGCTGCAGTACGGACTCCAAGAC	tag48283
novel_sir2134	CGGTCGATATCCTGCCTGAGGTTC	CAGCTATAGGACGGACTCCAAGAT	tag226900
novel_sir2135	GGTCGACATCGTGCCTGAGGTTC	AGCTGTAGCACGGACTCCAAGAC	tag237835
novel_sir2136	TAGTCGACATCGTGCCTGAGGTTC	CAGCTGTAGCACGGACTCCAAGAC	tag129804
novel_sir2137	TGGTCGACATCGTGCCTGAGGTTC	CAGCTGTAGCACGGACTCCAAGAC	tag151881
novel_sir2138	CGGTCGACATCGTGCCTGAGGTTC	CAGCTGTAGCACGGACTCCAAGAC	tag160480
novel_sir2139	CAGTCGACATCGTGCCTGAGGTTC	CAGCTGTAGCACGGACTCCAAGAC	tag161079
novel_sir2140	CGGTTGACATCGTGCCTGAGGTTC	CAACTGTAGCACGGACTCCAAGAC	tag29141
novel_sir2141	GGTCGATATCGTGCCTGAGGTTC	AGCTATAGCACGGACTCCAAGAC	tag137992

novel_sir2142	CAGCTATAGCACGGACTCCAAGAC	CAGTCGATATCGTGCCTGAGGTTC	tag268881
novel_sir2143	AGCTGCAGCACGGACTCCAAGAC	GGTCGACGTCGTGCCTGAGGTTC	tag255328
novel_sir2144	CGGTCGACGTCGTGCCTGAGGTTC	CAGCTGCAGCACGGACTCCAAGAT	tag41366
novel_sir2145	CAGTCGACGTCGTGCCTGAGGTTC	CAGCTGCAGCACGGACTCCAAGAT	tag65224
novel_sir2146	TGGTCGACGTCGTGCCTGAGGTTC	CAGCTGCAGCACGGACTCCAAGAT	tag69729
novel_sir2147	CAGCTGCAGCACGGACTCCAAGAC	CGGTCGACGTCGTGCCTGAGGTTC	tag41366
novel_sir2148	CAGCTGCAGCACGGACTCCAAGAC	CAGTCGACGTCGTGCCTGAGGTTC	tag65224
novel_sir2149	CAGCTGCAGCACGGACTCCAAGAC	TGGTCGACGTCGTGCCTGAGGTTC	tag69729
novel_sir2150	CAGCTGCAACACGGACTCCAAGAC	CGGTCGACGTTGTGCCTGAGGTTC	tag115821
novel_sir2151	CAGCTGTAGCACGAACTCCAAGAC	CGGTCGACATCGTGCTTGAGGTTC	tag245122
novel_sir2152	CAGAGATTCAGTCTTAGGTTC	CTCTAAGTCAGAATCCAAGCT	tag202101
novel_sir2153	CGGAGATTCAGTCTTAGGTTC	CTCTAAGTCAGAATCCAAGCT	tag255827
novel_sir2154	GCGGAGATTCAGTCTTAGGTTC	CCTCTAAGTCAGAATCCAAGCT	tag108963
novel_sir2155	TTGCGGAGATTCAGTCTTAGGTTC	CGCCTCTAAGTCAGAATCCAAGCT	tag8571
novel_sir2156	CTCCTCGCGCGTGGCCAAGTG	CGGAGGAGCGCGCACCGGTTC	tag285012
novel_sir2157	CCTCCTCGCGCGTGGCCAAGTG	ACGGAGGAGCGCGCACCGGTTC	tag78762
novel_sir2158	TCTTAAATCTCAGATCGGTTC	AATTTAGAGTCTAGCCAAGTG	tag57213
novel_sir2159	AAGAATTTAGAGTCTAGCCAAGTG	AATTCTTAAATCTCAGATCGGTTC	tag291761
novel_sir2160	GTTCGGCTCGTTACATGGGTTC	AGCCGAGCAATGTACCCAAGTT	tag182996
novel_sir2161	TCGCAGGACGTTGTAGACTGGTTC	CGTCCTGCAACATCTGACCAAGAT	tag42442
novel_sir2162	CCGCAGGACGTTGTAGACTGGTTC	CGTCCTGCAACATCTGACCAAGAT	tag221920
novel_sir2163	ATCATAGCTACTGAAGACCAAGCA	AGTAGTATCGATGACTTCTGGTTC	tag183891
novel_sir2164	CCACTGGCTTTTCAACCAAGCG	GCGGTGACCGAAAAGTTGGTTC	tag80599
novel_sir2165	TGGCCCTACTGTGTCGCCATGTTC	CGGGATGACACAGCGGTACAAGTT	tag82623
novel_sir2166	AAGGTTCTTTACGGTAGCATGTTC	CCAAGAAATGCCATCGTACAAGCG	tag19563
novel_sir2167	CATCTAGCGCAGCTCCCGATGTTC	AGATCGCGTCGAGGGCTACAAGTT	tag165106
novel_sir2168	TGGATCTACTGTATGGCGATGTTC	CTAGATGACATACCGCTACAAGTT	tag32821
novel_sir2169	CGGCTCCAGCAACGACAAGGC	CTGCCGAGGTCGTTGCTGTTC	tag196092
novel_sir2170	AGCCAACATACGCCAAGACAAGCC	GTTCGGTTGTATGCGGTTCTGTTC	tag115044
novel_sir2171	GTTCGGTTGTATGCGGTTCTGTTC	AGCCAACATACGCCAAGACAAGAC	tag115044
novel_sir2172	CACATCATCATCCACTAACAAGAT	CGGTGTAGTAGTAGGTGATTGTTC	tag113523
novel_sir2173	TGGTCCCACTGTGTTGACTTGTTC	CAGGGTGACACAACTGAACAAGTT	tag280768
novel_sir2174	CGGCACATATAATTTCTTAAAGAT	TGGCCGTGTATATTAAAGAATTTC	tag123768
novel_sir2175	GGTGTACAGTAGTTGAGTGATTTC	ACATGTCATCAACTCACTAAAGTG	tag261168

novel_sir2176	AAGCCTTGCAGCCTTTGATTTC	CGGAACGTCGGAAACTAAAGCG	tag132296
novel_sir2177	CTTAGTCTTGGAAACGTCAAAGTG	ACGAATCAGAACCTTTGCAGTTTC	tag153244
novel_sir2178	GGTGATCGGGTTTAACGTTTC	ACTAGCCCAAATTGCAAAGGA	tag249413
novel_sir2179	ACATGTCATCAACTCAACAAAGTG	GGTGTACAGTAGTTGAGTTGTTTC	tag44471
novel_sir2180	AGTAGCGCGAACCAACTTTTC	ATCGCGCTTGGTTGAAAAGCC	tag222712
novel_sir2181	ACAGGTCGATGTGATGGAAAAGGA	ACTGTCCAGCTACACTACCTTTTC	tag85780
novel_sir2182	GAGGCGGCCACTTCTCTTTTC	CCGCCGGTGAAGAGAAAAGTG	tag169283
novel_sir2183	GCGCGTGCCTCTCCCTTTTTC	CGCACGGAGAGGGAAAAAGGC	tag46742
novel_sir2184	AGCCAACTTATAAGTCAAAAAGTA	ATTCGGTTGAATATTCAGTTTTTC	tag74599
novel_sir2185	TTGCCAGTTTGTACAAATTTTTTC	CGGTCAAACATGTTTAAAAAAGTC	tag118168
novel_sir2186	AGTGAACGTGGTGCCTTTTTTCGA	TGTCACTTGCACCACGGAAAAAAG	tag238199
novel_sir2187	AACGGCCATTCCTGTGTTTTTCCT	GTTTGCCGGTAAGGACACAAAAAG	tag288556
novel_sir2188	TATCTACTGCGACAACTGAAAAAG	AGATGACGCTGTTGACTTTTTCTC	tag278503
novel_sir2189	AGATGACGCCGTTGACTTTTTCTT	TATCTACTGCGGCAACTGAAAAAG	tag4065
novel_sir2190	TATCTACTGCGGCAACTGAAAAAG	AGATGACGCCGTTGACTTTTTCTC	tag4065
novel_sir2191	AGATGACGCCGTTGACTTTTTCCA	TATCTACTGCGGCAACTGAAAAAG	tag4065
novel_sir2192	AGATGACGCCATTGACTTTTTCTC	TATCTACTGCGGTAACTGAAAAAG	tag214783
novel_sir2193	CCGGATGTCGGAAGGGGTTTTCGG	CTGGCCTACAGCCTTCCCCAAAAG	tag142891
novel_sir2194	CAGGTAGTCGATACAGTGCAAAAG	CCATCAGCTATGTCACGTTTTCAT	tag83755
novel_sir2195	CTTAAAGAGCCAGAGCCAGAAAAG	ATTTCTCGGTCTCGGTCTTTTCGG	tag15340
novel_sir2196	GCTGACCACATATTCACCGAAAAG	ACTGGTGTATAAGTGGCTTTTCTC	tag190024
novel_sir2197	CTTCGCGCCACTGGCTTTTCAA	TCGAAGCGCGGTGACCGAAAAG	tag27007
novel_sir2198	ACTATTGAGCTCGACGGCGAAAAG	ATAACTCGAGCTGCCGCTTTTCAT	tag289554
novel_sir2199	AACCCTCCACCATTCACTTTTCCC	TGTTGGGAGGTGGTAAGTGAAAAG	tag197962
novel_sir2200	TGGGCGTGTGCTTTTAACTAAAAG	CCGCACACGAAAATTGATTTTCGT	tag265260
novel_sir2201	TCTCTGACAACCTCACGCTAAAAG	AGACTGTTGGAGTGCGATTTTCGT	tag168980
novel_sir2202	CTGTCTATGTCTGTGTTTCTC	CGGACAGATACAGACACAAAG	tag19746
novel_sir2203	AACCTAACTCCGCTCGTGTTTCAT	CGTTGGATTGAGGCGAGCACAAAG	tag16923
novel_sir2204	ATCACTAATGAAGCCGTGTTTCAG	TGTAGTGATTACTTCGGCACAAAG	tag253838
novel_sir2205	GATGTGAGATGTTGGGTACCAAAG	ACACTCTACAACCCATGGTTTCTT	tag99566
novel_sir2206	CTTGGTGGCACGCGGTTTCGT	GCGAACCACCGTGCGCCAAAG	tag264701
novel_sir2207	AACTTTTCCGTCACATCGTTTCAA	GGTTGAAAAGGCAGTGTAGCAAAG	tag238740
novel_sir2208	AACCTCACCTGCCAAGCGTTTCGC	GGTTGGAGTGGACGGTTCGCAAAG	tag244435
novel_sir2209	CTCTAAGTTTACAGTCCGTTTCTT	CGGAGATTCAAATGTCAGGCAAAG	tag198200

novel_sir2210	CCTCCGGCTGAACCGTTTCCA	ATGGAGGCCGACTTGGCAAAG	tag130360
novel_sir2211	GGTACCCAACATCTCACATCAAAG	ATGGGTTGTAGAGTGTAGTTTCTA	tag145467
novel_sir2212	CCTCGAGTCGGTTTGTCAAAG	AGCTCAGCCAAACAGTTTCAG	tag167085
novel_sir2213	ATCTCAATCGGGCCGAAGTTTCGC	ATTAGAGTTAGCCCGGCTTCAAAG	tag221113
novel_sir2214	CATGCTAACCAATACATTTCAAAG	ACGATTGGTTATGTAAAGTTTCTT	tag92178
novel_sir2215	TGTTGCTTGCACCACAGAAGAAAG	AACGAACGTGGTGTCTTCTTTCAA	tag288262
novel_sir2216	AGTATTGACTCGGTCTTCTTTCCA	CGTCATAACTGAGCCAGAAGAAAG	tag137377
novel_sir2217	TGTCGCTTGCACCACTGAAGAAAG	AGCGAACGTGGTGACTTCTTTCAA	tag104930
novel_sir2218	ATCGATAGGCCACGCTTTCAC	CCTAGCTATCCGGTGCGAAAG	tag86783
novel_sir2219	AGCCAAGCCGGGTGAAGCTTTCTC	CCTCGGTTCGGCCCACTTCGAAAG	tag199948
novel_sir2220	AGCGAACGTGGTGTCTCCTTTCAA	TGTCGCTTGCACCACAGAGGAAAG	tag230669
novel_sir2221	AGCGAACGTGGTGGCTCCTTTCAA	GTTCGCTTGCACCACCGAGGAAAG	tag144139
novel_sir2222	TGTCACTTGCACCACGGAGGAAAG	AGTGAACGTGGTGCCTCCTTTCAA	tag198070
novel_sir2223	AGCGAACGTGGTGCCTCCTTTCAA	TGTCGCTTGCACCACGGAGGAAAG	tag279592
novel_sir2224	TGTCGCTTGTACCACGGAGGAAAG	AGCGAACATGGTGCCTCCTTTCAA	tag214323
novel_sir2225	AGTTTGCTGTCAAACTTCTGAAAG	AAACGACAGTTTGAAGACTTTCCT	tag226610
novel_sir2226	TGTTTCGCTGCTGCCGTGAAAG	AAAGCGACGACGGCACTTTCGG	tag252667
novel_sir2227	TAGATGAAAACTATCAGTATAAAG	CTACTTTTGATAGTCATATTTCAT	tag122471
novel_sir2228	TTTCAGTTGCCGCGGTTTATAAAG	AGTCAACGGCGCCAAATATTTCGA	tag250700
novel_sir2229	AACAACGGATGGCCTAGATTTCAT	CCTTGTTGCCTACCGGATCTAAAG	tag63939
novel_sir2230	TTTCAGTTGCCGCAGTTTGTAAAG	AGTCAACGGCGTCAAACATTTCGA	tag275151
novel_sir2231	TGGTCCAGTGGTGCCTAGTTAAAG	CAGGTCACCACGGATCAATTTCGT	tag25575
novel_sir2232	ACTTTAGATCCGGTAGGCAACAAG	AAATCTAGGCCATCCGTTGTTCCT	tag10360
novel_sir2233	CACGGCTAAGGTTGTGTTCCA	TGGTGCCGATTCCAACACAAG	tag255926
novel_sir2234	ATCCGATTACACCGTTGTGTTCGT	GCTAGGCTAATGTGGCAACACAAG	tag141036
novel_sir2235	ATCCGATTACACCGTTGTGTTCAT	GCTAGGCTAATGTGGCAACACAAG	tag141036
novel_sir2236	TCGCACATAAGGAAAACGCACAAG	CGTGTATTCCTTTTGCGTGTTCGC	tag262260
novel_sir2237	CATCGGAACATCCATAGGCACAAG	AGCCTTGTAGGTATCCGTGTTCAA	tag11051
novel_sir2238	ATCCTATAGCAAATACGTGTTCTT	TATAGGATATCGTTTATGCACAAG	tag236799
novel_sir2239	CATTGGCCAGAGGCTGTTCAC	AGGTAACCGGTCTCCGACAAG	tag241861
novel_sir2240	ACCAATCTGTAGCCCGCTGTTCTT	GATGGTTAGACATCGGGCGACAAG	tag4988
novel_sir2241	TGTTCACTCCATCTGCCGACCAAG	AAGTGAGGTAGACGGCTGGTTCAG	tag255125
novel_sir2242	AGGTATCTAGTCAGACGGGTTCCA	TGTCCATAGATCAGTCTGCCCAAG	tag57661
novel_sir2243	CCTCCTCCTGCCACGTCGGTTCGC	CGGGAGGAGGACGGTGCAGCCAAG	tag262131

novel_sir2244	ACTCCTCTCTACCGGCTCGCCAAG	AGGAGAGATGGCCGAGCGGTTCAA	tag20173
novel_sir2245	CCTCCTCTCTACCGGCTCGCCAAG	AGGAGAGATGGCCGAGCGGTTCAA	tag171766
novel_sir2246	AGGCCACGCTTTCACGGTTCGT	TATCCGGTGCGAAAGTGCCAAG	tag253244
novel_sir2247	CGGAGGCAACAACATCAGCAAG	CTCCGTTGTTGTAGTCGTTCAC	tag274302
novel_sir2248	ATTCTGACTTAGAGGCGTTCAG	CCTAAGACTGAATCTCCGCAAG	tag153188
novel_sir2249	CTTCGGCTGCCTCGCGTTCGC	CAGAAGCCGACGGAGCGCAAG	tag85305
novel_sir2250	ACCCTTCGACAGCGAGCCGTTCGT	CATGGGAAGCTGTCGCTCGGCAAG	tag4936
novel_sir2251	GGGGATTTGAAGGAGTTTGGCAAG	CCTAAACTTCCTCAAACCGTTCAC	tag264217
novel_sir2252	TATCTGAACTAGACAATCTGCAAG	AGACTTGATCTGTTAGACGTTCAG	tag281536
novel_sir2253	GTTGGATAGCTGTCACTCTGCAAG	ACCTATCGACAGTGAGACGTTCGT	tag268350
novel_sir2254	CGCCACCGACAGCGAGACGTTCAT	GGGCGGTGGCTGTCGCTCTGCAAG	tag225083
novel_sir2255	ATTAAGCCAGCTGGCGCTATCAAG	ATTCGGTCGACCGCGATAGTTCGG	tag92781
novel_sir2256	ATTAAGCCAGCTGGCGCTATCAAG	ATTCGGTCGACCGCGATAGTTCAG	tag92781
novel_sir2257	CCGATGTGCGTTTTATAAGTTCGT	ATGGCTACACGCAAAATATTCAAG	tag102251
novel_sir2258	TGTTTAGACATCGGGCGAAAGAAG	AAATCTGTAGCCCGCTTTCTTCTC	tag28773
novel_sir2259	TGGCCGGCTGCCCTCAAGAAG	CGGCCGACGGGAGTTCTTCAC	tag194468
novel_sir2260	CATGGCAGTCCGGATTCGAAGAAG	ACCGTCAGGCCTAAGCTTCTTCTT	tag260179
novel_sir2261	CTTGGTGTTTTGGCCTGTAAGAAG	ACCACAAAACCGGACATTCTTCAC	tag111155
novel_sir2262	CATAACAGGAACGTGTCGCAGAAG	ATTGTCCTTGCACAGCGTCTTCTG	tag234775
novel_sir2263	ACTACGAATTTGGACAGTCTTCTA	CATGATGCTTAAACCTGTCAGAAG	tag245236
novel_sir2264	TAGGAAGCTACAGCCGAGAAG	CCTTCGATGTCGGCTCTTCCT	tag258801
novel_sir2265	CGTCGAAGCCTTCTAGGCGAGAAG	AGCTTCGGAAGATCCGCTCTTCGT	tag149861
novel_sir2266	CGATGGCCGGGACTTCCTCTTCAT	CTGCTACCGGCCCTGAAGGAGAAG	tag228247
novel_sir2267	CCTGGCACTATTCATCTTCAA	AGGGACCGTGATAAGTAGAAG	tag287133
novel_sir2268	GTTGAAAAGTCAGTGTAGTAGAAG	ACTTTTCAGTCACATCATCTTCAA	tag144798
novel_sir2269	ATTGAAAAGTCAGTGTAGTAGAAG	ACTTTTCAGTCACATCATCTTCAA	tag239897
novel_sir2270	GGTAGCGCCGCGAGCCACGAAG	ATCGCGGCGCTCGGTGCTTCCG	tag265574
novel_sir2271	CGTCCTTTAGGCGTACCGAAG	AGGAAATCCGCATGGCTTCTC	tag223499
novel_sir2272	GTTCGCATGGCTGTTCCCGAAG	AGCGTACCGACAAGGGCTTCTC	tag218026
novel_sir2273	TATCGTAACCGGGATGAAGCGAAG	AGCATTGGCCCTACTTCGCTTCGC	tag50112
novel_sir2274	AATCGTAACCGGGATGAAGCGAAG	AGCATTGGCCCTACTTCGCTTCGC	tag70286
novel_sir2275	CATCGTAACCGGGATGAAGCGAAG	AGCATTGGCCCTACTTCGCTTCGC	tag191240
novel_sir2276	TATCGTAACCGGGATGAAGCGAAG	AGCATTGGCCCTACTTCGCTTCGA	tag50112
novel_sir2277	AATCGTAACCGGGATGAAGCGAAG	AGCATTGGCCCTACTTCGCTTCGA	tag70286

novel_sir2278	CATCGTAACCGGGATGAAGCGAAG	AGCATTGGCCCTACTTCGCTTCGA	tag191240
novel_sir2279	GGGGCGGTGGCCGCGGCGAAG	CCGCCACCGGCGCCGCTTCCC	tag132079
novel_sir2280	AAGGCGGCAGCGGCGGCGAAG	CCGCCGTCGCCGCCGCTTCCC	tag276819
novel_sir2281	CGGGACACGTCGGCGGCGAAG	CCTGTGCAGCCGCCGCTTCCT	tag87243
novel_sir2282	ATCCGGCACACGGTAGCTTCGC	ATTAGGCCGTGTGCCATCGAAG	tag60539
novel_sir2283	ATGTCCCCAGAAGGCCGATCGAAG	CAGGGGTCTTCCGGCTAGCTTCAC	tag40535
novel_sir2284	GGGTCCCCAGAAGGCCGATCGAAG	CAGGGGTCTTCCGGCTAGCTTCAC	tag77573
novel_sir2285	CCGTTGGGAAGGGAGCTTCGA	CGGGCAACCCTTCCCTCGAAG	tag35333
novel_sir2286	CCCGTTGGGAAGGGAGCTTCGA	GCGGGCAACCCTTCCCTCGAAG	tag70388
novel_sir2287	CGATACCATCTCTAAGCTTCTC	GCGCTATGGTAGAGATTCGAAG	tag103416
novel_sir2288	CATATTGCTTAGCCCTGTTCGAAG	ATAACGAATCGGGACAAGCTTCTG	tag116860
novel_sir2289	AAATCTATAGCCCACTGCCTTCTC	TGTTTAGATATCGGGTGACGGAAG	tag17278
novel_sir2290	CCGGCGGAGCTTCGAGGGAAG	CCGCCTCGAAGCTCCCTTCCC	tag274934
novel_sir2291	AATAAGCACAGGCTTTTCGGGAAG	ATTCGTGTCCGAAAAGCCCTTCCG	tag117928
novel_sir2292	CCGTACTCGCGGCGCTGGAAG	CATGAGCGCCGCGACCTTCCA	tag97596
novel_sir2293	ACTCTGTCAAACGGTCTACTTCGT	AATGAGACAGTTTGCCAGATGAAG	tag46302
novel_sir2294	GGTCGACCCGGTTGAGATGAAG	AGCTGGGCCAACTCTACTTCGT	tag261613
novel_sir2295	CGGGACCCTCTTGGACTACTTCGT	ATGCCCTGGGAGAACCTGATGAAG	tag65255
novel_sir2296	GAAACCTTGTTACGACTTCTC	GCCTTTGGAACAATGCTGAAG	tag171509
novel_sir2297	CGCCGCGATCCGAACACTTCAC	CAGCGGCGCTAGGCTTGTGAAG	tag229069
novel_sir2298	AGCCATCGCTAGATCGTTATTCCG	TTTCGGTAGCGATCTAGCAATAAG	tag163720
novel_sir2299	TTGCAAACTGGTGAGCAGAATAAG	CGTTTGACCACTCGTCTTATTCAA	tag214169
novel_sir2300	GTTCCGGTTCTGATTCCTAATAAG	AGGCCAAGACTAAGGATTATTCAC	tag69938
novel_sir2301	CGGAAACCCTGATACCATATTCGT	CTGCCTTTGGGACTATGGTATAAG	tag797
novel_sir2302	CCAAGGCCTACGATGATTCAG	TGGGTTCCGGATGCTACTAAG	tag68891
novel_sir2303	TTGGGTTCCGGATGCTACTAAG	CCCAAGGCCTACGATGATTCAG	tag46755
novel_sir2304	CGCTTGCTAGCTTGGATTCTG	CGGCGAACGATCGAACCTAAG	tag45660
novel_sir2305	CCGCTTGCTAGCTTGGATTCTG	GCGGCGAACGATCGAACCTAAG	tag141403
novel_sir2306	AGTAGGTGGCCGAATCGGATTCCA	CATCATCCACCGGCTTAGCCTAAG	tag9370
novel_sir2307	GGTGACTGTACACCTTGGCCTAAG	ACTGACATGTGGAACCGGATTCAC	tag126986
novel_sir2308	TCGGGGCGCAGCGTGCCTAAG	CCCCGCGTCGCACGGATTCGT	tag255629
novel_sir2309	TCGGGGCGCAGCGTGCCTAAG	CCCCGCGTCGCACGGATTCGC	tag255629
novel_sir2310	TCGGGGCGCAGCGTGCCTAAG	CCCCGCGTCGCACGGATTCGG	tag255629
novel_sir2311	TCGGGGCGCAGCGTGCCTAAG	CCCCGCGTCGCACGGATTCGA	tag255629

novel_sir2312	TCGGGGCGCAGCGTGCCTAAG	CCCCGCGTCGCACGGATTCAT	tag255629
novel_sir2313	GGTCGGGGCGCAGCGTGCCTAAG	AGCCCCGCGTCGCACGGATTCGA	tag113122
novel_sir2314	GGTCGGGGCGCAGCGTGCCTAAG	AGCCCCGCGTCGCACGGATTCGG	tag113122
novel_sir2315	GGTCGGGGCGCAGCGTGCCTAAG	AGCCCCGCGTCGCACGGATTCGT	tag113122
novel_sir2316	GGTCGGGGCGCAGCGTGCCTAAG	AGCCCCGCGTCGCACGGATTCGC	tag113122
novel_sir2317	CCGAAAGTTAGATTTGCCTAAG	CTTTCAATCTAAACGGATTCAA	tag73525
novel_sir2318	CCTGATAGCGGAGACAGTCCTAAG	ACTATCGCCTCTGTCAGGATTCGG	tag181175
novel_sir2319	AGTCGTCTGCAAAGGATTCAG	ATTCAGCAGACGTTTCCTAAG	tag144476
novel_sir2320	AAGTCGTCTGCAAAGGATTCAG	AATTCAGCAGACGTTTCCTAAG	tag149257
novel_sir2321	CGCGCCGCGCCGTCGATTCCG	CGGCGCGGCGCGGCAGCTAAG	tag261247
novel_sir2322	CCGCGCCGCGCCGTCGATTCCG	TCGGCGCGGCGCGGCAGCTAAG	tag36892
novel_sir2323	ATTAGCCCAACTATTTAGATTCTT	TTTAATCGGGTTGATAAATCTAAG	tag67915
novel_sir2324	CCAGGCGCCACGGGAGATTCGG	GAGGTCCGCGGTGCCCTCTAAG	tag161852
novel_sir2325	CCGTTGATGTGTTCCAAGATTCGT	GTGGCAACTACACAAGGTTCTAAG	tag183817
novel_sir2326	TCTCGTTGATCCAAATTTTCTAAG	AGCAACTAGGTTTAAAAGATTCGT	tag6609
novel_sir2327	ATTAATTAGGCTCAAAAGATTCGT	CCTAATTAATCCGAGTTTTCTAAG	tag227484
novel_sir2328	CCTGATTAATCCGAGTTTTCTAAG	ACTAATTAGGCTCAAAAGATTCGT	tag25025
novel_sir2329	ATCTGGCCATCGATTTTCATTCAA	TGTAGACCGGTAGCTAAAAGTAAG	tag238778
novel_sir2330	CATCCTAGGACTAGTCTCATTCCA	GGGTAGGATCCTGATCAGAGTAAG	tag132048
novel_sir2331	ACCGCTTAACTATCATTCAT	TGTGGCGAATTGATAGTAAG	tag167948
novel_sir2332	CACCGCTTAACTATCATTCAA	CTGTGGCGAATTGATAGTAAG	tag26414
novel_sir2333	CACCGCTTAACTATCATTCAT	CTGTGGCGAATTGATAGTAAG	tag26414
novel_sir2334	AAGGCGCAGGTTGAAACTAGTAAG	CCGCGTCCAACTTTGATCATTCGT	tag185515
novel_sir2335	CTCGCGCTTACTAGGCATTCCT	CTGAGCGCGAATGATCCGTAAG	tag109922
novel_sir2336	GGTAGGTCGCCTCCCGGTAAG	ATCCAGCGGAGGGCCATTCTC	tag239981
novel_sir2337	ACCAGAAATTACGAGGCCATTCAG	CTTGGTCTTTAATGCTCCGGTAAG	tag29347
novel_sir2338	CGCTTGCAACGTTTGACCATTCAT	AAGCGAACGTTGCAAACTGGTAAG	tag32360
novel_sir2339	AAGGCGCAGGTTGAAACTGGTAAG	CCGCGTCCAACTTTGACCATTCGT	tag22724
novel_sir2340	CGGTTGGGTCCGGTTGGTAAG	CAACCCAGGCCAACCATTCCA	tag42210
novel_sir2341	GGTGCACCGCCGGATTCTTGTAAG	ACGTGGCGGCCTAAGAACATTCGT	tag274308
novel_sir2342	AGGCCCATTCGAAGATTAATTCTT	ATTCCGGGTAAGCTTCTAATTAAG	tag283071
novel_sir2343	TGGCACGGCGCTATCATTAAG	CGTGCCGCGATAGTAATTCAA	tag251324
novel_sir2344	CTGGCACGGCGCTATCATTAAG	CCGTGCCGCGATAGTAATTCAA	tag47846
novel_sir2345	ACAGACCAAGGATAAGTAATTCTA	TGTGTCTGGTTCCTATTCATTAAG	tag247904

novel_sir2346	AGTCTTATACGGTAGCTTAAG	AGAATATGCCATCGAATTCGC	tag128052
novel_sir2347	ACAACAACGGAGGCAATTCTG	GATGTTGTTGCCTCCGTTAAG	tag147853
novel_sir2348	GAGAACGCAGTGAGTGGTTAAG	CTTGCGTCACTCACCAATTCTC	tag72945
novel_sir2349	GGTACACCGCAGGCATCATTTAAG	ATGTGGCGTCCGTAGTAAATTCGT	tag215909
novel_sir2350	CGCTCTCATGGCCTAGAAATTCTC	AAGCGAGAGTACCGGATCTTTAAG	tag194000
novel_sir2351	TTTATCCGGAATGGTTTAAAACAG	ATAGGCCTTACCAAATTTTGTCAG	tag58681
novel_sir2352	TTTATCCGGAATGGTTTAAAACAG	ATAGGCCTTACCAAATTTTGTCAA	tag58681
novel_sir2353	GGTGATTGAACAGATACTAAACAG	ACTAACTTGTCTATGATTTGTCAC	tag192298
novel_sir2354	TGTGAAACACCGATCGTTAAACAG	ACTTTGTGGCTAGCAATTTGTCCA	tag177046
novel_sir2355	TGCTTAGACCTATGTCTCCAACAG	GAATCTGGATACAGAGGTTGTCTT	tag269698
novel_sir2356	AGCTTGACTCGATATCGGGAACAG	GAACTGAGCTATAGCCCTTGTCAA	tag37304
novel_sir2357	GGCTTGACTCGATATCGGGAACAG	GAACTGAGCTATAGCCCTTGTCAA	tag288419
novel_sir2358	ACCAGCGGTCCTTAAACTTGTCAG	CGTGGTCGCCAGGAATTTGAACAG	tag148354
novel_sir2359	ACCAGCGGTCCTTAAACTTGTCAG	TGTGGTCGCCAGGAATTTGAACAG	tag158437
novel_sir2360	CCTAAGCTTGTACCGTTGTGTCAT	AGGGATTCGAACATGGCAACACAG	tag176941
novel_sir2361	GTGCCCGCCGAGCCGACACAG	CGGGCGGCTCGGCTGTGTCCA	tag42064
novel_sir2362	CGTGCCCGCCGAGCCGACACAG	ACGGGCGGCTCGGCTGTGTCCA	tag115875
novel_sir2363	CCTAAACTTGTACCGCTGTGTCAT	AGGGATTTGAACATGGCGACACAG	tag28737
novel_sir2364	AGGAGTTTGAACAAGCTGACACAG	CTCAAACTTGTTCGACTGTGTCAT	tag273986
novel_sir2365	TGCTTAGATTTGTCAAATACACAG	GAATCTAAACAGTTTATGTGTCAC	tag139656
novel_sir2366	TCTAAGCAACATGATCCTACACAG	ATTCGTTGTACTAGGATGTGTCCC	tag253930
novel_sir2367	GTTGGGTTACTGATCCTACCACAG	ACCCAATGACTAGGATGGTGTCCA	tag46355
novel_sir2368	CATTGGCCGGCCACGCGGTGTCGC	TGGTAACCGGCCGGTGCGCCACAG	tag62608
novel_sir2369	CGACTTAGCTACGACTCGTGTCGC	TGGCTGAATCGATGCTGAGCACAG	tag224022
novel_sir2370	TAGATGAAAGAATTATTGGCACAG	CTACTTTCTTAATAACCGTGTCCA	tag57917
novel_sir2371	TGGATGAAAGAATTATTGGCACAG	CTACTTTCTTAATAACCGTGTCCA	tag210383
novel_sir2372	CTGGACCAGAGGAAATGTGCACAG	CCTGGTCTCCTTTACACGTGTCGT	tag133385
novel_sir2373	ACCCAATGACTAGGATAGTGTCCA	GTTGGGTTACTGATCCTATCACAG	tag133464
novel_sir2374	ATCCAACGTTTGACAGTCTGTCTT	CATAGGTTGCAAACTGTCAGACAG	tag130336
novel_sir2375	CCGCCGCCGCGTCGAGGACAG	CGGCGGCGCAGCTCCTGTCGG	tag73243
novel_sir2376	TCTAAAGATCATGATACTATACAG	ATTTCTAGTACTATGATATGTCAA	tag141489
novel_sir2377	TCTAAATAACATGATCCTATACAG	ATTTATTGTACTAGGATATGTCAT	tag183693
novel_sir2378	ATGCTATTCTGACTCATATGTCAT	TATACGATAAGACTGAGTATACAG	tag127535
novel_sir2379	CTGCGCCGTCTCGACATATGTCAT	GAGACGCGGCAGAGCTGTATACAG	tag222782

novel_sir2380	TCTAAGCAACATGATCTTATACAG	ATTCGTTGTACTAGAATATGTCAT	tag125801
novel_sir2381	ACCCAATGACTAGGATGATGTCCA	GTTGGGTTACTGATCCTACTACAG	tag15542
novel_sir2382	TGTAGCTTGCAAACTGACCTACAG	ATCGAACGTTTGACTGGATGTCGG	tag51211
novel_sir2383	TGGAAACTGCAAACTAGCCTACAG	CTTTGACGTTTGATCGGATGTCGG	tag124580
novel_sir2384	TGTAGCTTGCAAACCGGCCTACAG	ATCGAACGTTTGGCCGGATGTCGG	tag164739
novel_sir2385	TGTAGCTTGCAAACTGGCCTACAG	ATCGAACGTTTGACCGGATGTCGG	tag94209
novel_sir2386	TGTAGCTTGCAAACTGGCCTACAG	ATCGAACGTTTGACCGGATGTCGA	tag94209
novel_sir2387	CGGTGTGAAAAAGTCATCGTACAG	CACACTTTTTCAGTAGCATGTCCC	tag245105
novel_sir2388	ACTGAGTTACAGTGGCTCTTACAG	ACTCAATGTCACCGAGAATGTCAC	tag5433
novel_sir2389	ATCCTAACGATGGCTAAATGTCAC	GATAGGATTGCTACCGATTTACAG	tag89221
novel_sir2390	CGGTGCATCCTGTTTCTGAACCAG	CACGTAGGACAAAGACTTGGTCAA	tag206974
novel_sir2391	CTGAGGCGTGCTCGTAGTGGTCGA	TCGACTCCGCACGAGCATCACCAG	tag76092
novel_sir2392	AGCAAATCCGCAAGCTGGTCCT	ACTCGTTTAGGCGTTCGACCAG	tag136448
novel_sir2393	TCTACGTAACAGGATCTTGCCCAG	ATGCATTGTCCTAGAACGGGTCGC	tag79722
novel_sir2394	ACATCTTTAGTCCCGGTCGGTCAA	TTTGTAGAAATCAGGGCCAGCCAG	tag44735
novel_sir2395	TGTGCTCAGCATCGATTCAGCCAG	ACGAGTCGTAGCTAAGTCGGTCGA	tag282044
novel_sir2396	CAGCCGCAGCCGAGGAGCCAG	CGGCGTCGGCTCCTCGGTCCT	tag13485
novel_sir2397	ACGTGGCTAATTTGACTCGGTCTT	GATGCACCGATTAAACTGAGCCAG	tag54211
novel_sir2398	CCTTTGTGTAGAAGCGATCGCCAG	AAACACATCTTCGCTAGCGGTCGC	tag267630
novel_sir2399	CCTCAACTATACAAATCCGGTCAT	AGGGAGTTGATATGTTTAGGCCAG	tag285265
novel_sir2400	TACTTAGACCTGTCTCTCGGCCAG	GAATCTGGACAGAGAGCCGGTCCA	tag79944
novel_sir2401	TTGTCATCGAGCGCTTGGCCAG	CAGTAGCTCGCGAACCGGTCCT	tag72484
novel_sir2402	TGTTGTCATCGAGCGCTTGGCCAG	AACAGTAGCTCGCGAACCGGTCCT	tag22198
novel_sir2403	AAGAAATACTCGAAACGATGCCAG	CTTTATGAGCTTTGCTACGGTCCA	tag55447
novel_sir2404	ATTACTATCGCGGCACGGTCAT	CTTAATGATAGCGCCGTGCCAG	tag72935
novel_sir2405	CCTGTGGTCCAATCAAACGGTCAT	AAGGACACCAGGTTAGTTTGCCAG	tag138350
novel_sir2406	CGCATCCATCCACTTTTAGGTCTC	AGGCGTAGGTAGGTGAAAATCCAG	tag109140
novel_sir2407	CACATCCATCCACTTTTAGGTCTC	AGGTGTAGGTAGGTGAAAATCCAG	tag153119
novel_sir2408	GTTTTGCTCCATTCGGTAATCCAG	AAACGAGGTAAGCCATTAGGTCAT	tag149229
novel_sir2409	CGGCTATCTAATCCGCTAGGTCTA	GAGCCGATAGATTAGGCGATCCAG	tag272581
novel_sir2410	GATGCACCGAACAAACTGATCCAG	ACGTGGCTTGTTTGACTAGGTCTC	tag171044
novel_sir2411	GATGCACCGACCAAACTGATCCAG	ACGTGGCTGGTTTGACTAGGTCTC	tag106172
novel_sir2412	CGGTTAAGGATACCGCGACTCCAG	CAATTCCTATGGCGCTGAGGTCAC	tag225651
novel_sir2413	GAGAGATCGAGGCCGCTCCAG	CTCTAGCTCCGGCGAGGTCGC	tag75874

novel_sir2414	AGCACATGTTTGACCGTTCGTCTT	AATCGTGTACAAACTGGCAAGCAG	tag228114
novel_sir2415	AGCACATGTTTGACCGTTCGTCTA	AATCGTGTACAAACTGGCAAGCAG	tag228114
novel_sir2416	AATCGTGTACAAATTGGCAAGCAG	AGCACATGTTTAACCGTTCGTCTT	tag78768
novel_sir2417	AGCCGCGGTCGCCTTCGTCCT	CGTCGGCGCCAGCGGAAGCAG	tag259323
novel_sir2418	CCGCGCCTCCCCCTTCGTCGC	CCGGCGCGGAGGGGGAAGCAG	tag225310
novel_sir2419	GCTCCGCGGACACCACTGAAGCAG	AGGCGCCTGTGGTGACTTCGTCAA	tag262232
novel_sir2420	AGTGTACAAACTGGTAAGCAG	ACATGTTTGACCATTCGTCTT	tag10521
novel_sir2421	CACATGTTTGACCATTCGTCTT	GAGTGTACAAACTGGTAAGCAG	tag29209
novel_sir2422	AAGAGTGTACAAACTGGTAAGCAG	CTCACATGTTTGACCATTCGTCTT	tag41137
novel_sir2423	AATCGTGTACAAACTGGTAAGCAG	AGCACATGTTTGACCATTCGTCTT	tag126910
novel_sir2424	GGTGGCGTAGCCGCGACAGCAG	ACCGCATCGGCGCTGTCGTCGC	tag135661
novel_sir2425	GGTCGAGCACAGCAGCAGCAG	AGCTCGTGTCGTCGTCGTCCG	tag180594
novel_sir2426	AGCGTGGGCCACCGTCGTCGT	ATTCGCACCCGGTGGCAGCAG	tag82954
novel_sir2427	ATAGATTCTACCGAAAGTCGTCGG	GATATCTAAGATGGCTTTCAGCAG	tag13524
novel_sir2428	GTGCGCGGCAGTGGAGAGCAG	CGCGCCGTCACCTCTCGTCCT	tag83965
novel_sir2429	ACCGCCGCCACCTCTCGTCGC	GGTGGCGGCGGTGGAGAGCAG	tag150056
novel_sir2430	CACCGCCGCCACCTCTCGTCGC	CGGTGGCGGCGGTGGAGAGCAG	tag225732
novel_sir2431	GCTCGGAGGGAGATACAGTAGCAG	AGCCTCCCTCTATGTCATCGTCGA	tag4796
novel_sir2432	AGCCTCCCTCTATGCCATCGTCGA	GCTCGGAGGGAGATACGGTAGCAG	tag278569
novel_sir2433	TGCTGTGCCTACTCTGTCACGCAG	GACACGGATGAGACAGTGCGTCTC	tag270682
novel_sir2434	ATGGGGCTACGGATCGGGCGTCTG	CCTACCCCGATGCCTAGCCCGCAG	tag10614
novel_sir2435	ATGGGGCTACGGATCGGGCGTCTC	CCTACCCCGATGCCTAGCCCGCAG	tag10614
novel_sir2436	ATGGGGCTACGGATCGGGCGTCTT	CCTACCCCGATGCCTAGCCCGCAG	tag10614
novel_sir2437	ATCCAGCTCCTGTCGGCGTCGG	CCTAGGTCGAGGACAGCCGCAG	tag197198
novel_sir2438	ATTTAAAAAAGTCAACGGCGTCAA	TATAAATTTTTCAGTTGCCGCAG	tag179985
novel_sir2439	CTTTCCTGGTCGCGTCGG	TTGAAAGGACCAGCGCAG	tag259247
novel_sir2440	TGTTTGAAAGGACCAGCGCAG	AAACTTTCCTGGTCGCGTCGG	tag202754
novel_sir2441	CGCTGTTTGAAAGGACCAGCGCAG	GACAAACTTTCCTGGTCGCGTCGG	tag216822
novel_sir2442	ACGGTGAGGGACGCGCGTCCT	CGTGCCACTCCCTGCGCGCAG	tag68017
novel_sir2443	GTGACTCTAGGTCGGGGCGCAG	CTGAGATCCAGCCCCGCGTCGC	tag58674
novel_sir2444	GTGACTCTAGGTCGGGGCGCAG	CTGAGATCCAGCCCCGCGTCGT	tag58674
novel_sir2445	GAATAAGAGGAGCCTACGCGTCGG	TCCTTATTCTCCTCGGATGCGCAG	tag247428
novel_sir2446	CTAGCACATTGGCCCACGCGTCAT	ATGATCGTGTAACCGGGTGCGCAG	tag3748
novel_sir2447	GATTAGAACAAACCGGGATCGCAG	AATCTTGTTTGGCCCTAGCGTCGT	tag202741

novel_sir2448	TCGTGGGTTGAAACTAGCAGGCAG	CACCCAACTTTGATCGTCCGTCAT	tag120633
novel_sir2449	GTGTGGGTTGAAACTAGCAGGCAG	CACCCAACTTTGATCGTCCGTCAT	tag193924
novel_sir2450	TTGCTTAAACCTATCCTCAGGCAG	CGAATTTGGATAGGAGTCCGTCCA	tag161118
novel_sir2451	TGCTTAGATCTGTCGCTTAGGCAG	GAATCTAGACAGCGAATCCGTCCA	tag259102
novel_sir2452	CAGCGGGCTCTCGCTTGCCGGCAG	CGCCCGAGAGCGAACGGCCGTCTG	tag266785
novel_sir2453	CCCAGCCGCGCCGCGCCGTCGA	GCGGGTCGGCGCGGCGCGGCAG	tag23075
novel_sir2454	CGAGCAAGCCGCCGCCGTCGC	CGGCTCGTTCGGCGGCGGCAG	tag107996
novel_sir2455	CGCCGTAGCTAGCCGCCGTCGG	CGGCGGCATCGATCGGCGGCAG	tag187823
novel_sir2456	CCACTTACAATGCCCCGTCGC	ACGGTGAATGTTACGGGGCAG	tag263455
novel_sir2457	CGTCGAAGTTTTGCGCCCTGGCAG	AGCTTCAAAACGCGGGACCGTCAG	tag208804
novel_sir2458	CCGTTCCGGACACCGTGGCAG	CAAGGCCTGTGGCACCGTCTA	tag55703
novel_sir2459	ACGGAGCGACAGCTGCCCATGCAG	CCTCGCTGTCGACGGGTACGTCGC	tag47133
novel_sir2460	TAGTCGAGCGCAACTGATGCAG	CAGCTCGCGTTGACTACGTCCC	tag289516
novel_sir2461	TGCTTTTGTTATCGTAGCCTGCAG	GAAAACAATAGCATCGGACGTCCG	tag66843
novel_sir2462	CTTCCGAGGGGTGTTCTGCAG	AGGCTCCCCACAAGACGTCGG	tag212120
novel_sir2463	TGCTTTAATTATCCTACGGTGCAG	GAAATTAATAGGATGCCACGTCCT	tag68205
novel_sir2464	TACTTTAATTATCCTACGGTGCAG	GAAATTAATAGGATGCCACGTCCT	tag190965
novel_sir2465	CTCCGGACTTCCTAACGTCGC	CAGAGGCCTGAAGGATTGCAG	tag34950
novel_sir2466	AGCGGCCTCTTAACATAACGTCAG	GATCGCCGGAGAATTGTATTGCAG	tag61898
novel_sir2467	GGGAAGCTGTAGGCCAGTTTGCAG	CTTCGACATCCGGTCAAACGTCCG	tag190081
novel_sir2468	AAGTAAGCACAGGCTAGTTTGCAG	CATTCGTGTCCGATCAAACGTCTG	tag73341
novel_sir2469	TAGAAAACTCGGATTAATCAG	CTTTTGAGCCTAATTAGTCCA	tag208164
novel_sir2470	AATCTTTTGAGCCTAATTAGTCCA	GCTTAGAAAACTCGGATTAATCAG	tag47169
novel_sir2471	CTTTAGTCCCGGATTGGTAGTCCC	TAGAAATCAGGGCCTAACCATCAG	tag283058
novel_sir2472	TTTGTGCCAGATACAAAAGATCAG	ACACGGTCTATGTTTTCTAGTCAT	tag106581
novel_sir2473	GTGCCGTACCAATAGGCTGATCAG	CGGCATGGTTATCCGACTAGTCAT	tag13598
novel_sir2474	ATGCCGTACCAATAGGCTGATCAG	CGGCATGGTTATCCGACTAGTCAT	tag26779
novel_sir2475	TATGTATATAATGCAAAATATCAG	ACATATATTACGTTTTATAGTCCG	tag32983
novel_sir2476	AGCACCCGATTGAGATATAGTCGC	TTTCGTGGGCTAACTCTATATCAG	tag193398
novel_sir2477	AGGGAGGTAGATGAAAACTATCAG	CCTCCATCTACTTTTGATAGTCAT	tag171827
novel_sir2478	TGTATGCACAGGTCGAAGTATCAG	ATACGTGTCCAGCTTCATAGTCAG	tag272755
novel_sir2479	TCTCCTGGCGTACACTTTTATCAG	AGGACCGCATGTGAAAATAGTCAT	tag90290
novel_sir2480	CGGCCGCCGCAGGCCGACACTCAG	CGGCGGCGTCCGGCTGTGAGTCAT	tag249212
novel_sir2481	CGGGCGACAATATATGACACTCAG	CCGCTGTTATATACTGTGAGTCGT	tag158369

novel_sir2482	TATGTGTACAATACAGAACCTCAG	ACACATGTTATGTCTTGGAGTCCG	tag226888
novel_sir2483	TATGTGTATGATACAGAACCTCAG	ACACATACTATGTCTTGGAGTCCG	tag43984
novel_sir2484	TATGTATGTAACGCAGAACCTCAG	ACATACATTGCGTCTTGGAGTCCG	tag74950
novel_sir2485	TATGTGTGTAACGCAGAACCTCAG	ACACACATTGCGTCTTGGAGTCCG	tag97259
novel_sir2486	TATGTGTATGACGCAGAACCTCAG	ACACATACTGCGTCTTGGAGTCCA	tag177188
novel_sir2487	ACACATACTGCGTCTTGGAGTCCG	TATGTGTATGACGCAGAACCTCAG	tag177188
novel_sir2488	AGATACACTGCGTCTTGGAGTCCG	TATCTATGTGACGCAGAACCTCAG	tag224785
novel_sir2489	TGTGTGTGACGCAGAACCTCAG	ACACACTGCGTCTTGGAGTCCG	tag171351
novel_sir2490	ATACACACTGCGTCTTGGAGTCCG	AGTATGTGTGACGCAGAACCTCAG	tag183993
novel_sir2491	ATACACACTGCGTCTTGGAGTCCA	AGTATGTGTGACGCAGAACCTCAG	tag183993
novel_sir2492	TATGTGTGTGACGCAGAACCTCAG	ACACACACTGCGTCTTGGAGTCCG	tag102338
novel_sir2493	TATGTGTGCAATGCAGAACCTCAG	ACACACGTTACGTCTTGGAGTCCG	tag274058
novel_sir2494	TATATGTATAATGCAGAACCTCAG	ATACATATTACGTCTTGGAGTCCG	tag92497
novel_sir2495	ACACATATTACGTCTTGGAGTCCA	TATGTGTATAATGCAGAACCTCAG	tag238239
novel_sir2496	TATGTGTATAATGCAGAACCTCAG	ACACATATTACGTCTTGGAGTCCG	tag238239
novel_sir2497	TATGTATGTAATGCAGAACCTCAG	ACATACATTACGTCTTGGAGTCCA	tag240910
novel_sir2498	TATGTATGTAATGCAGAACCTCAG	ACATACATTACGTCTTGGAGTCCG	tag240910
novel_sir2499	TATATGTGTAATGCAGAACCTCAG	ATACACATTACGTCTTGGAGTCCG	tag106060
novel_sir2500	TATGTGTGTAATGCAGAACCTCAG	ACACACATTACGTCTTGGAGTCCA	tag23044
novel_sir2501	TGTGTGTGTAATGCAGAACCTCAG	ACACACATTACGTCTTGGAGTCCA	tag214730
novel_sir2502	TATGTGTGTAATGCAGAACCTCAG	ACACACATTACGTCTTGGAGTCCG	tag23044
novel_sir2503	TGTGTGTGTAATGCAGAACCTCAG	ACACACATTACGTCTTGGAGTCCG	tag214730
novel_sir2504	TATGTGTGGCATGCAGAACCTCAG	ACACACCGTACGTCTTGGAGTCCG	tag30406
novel_sir2505	TATGTGTGTCATGCAGAACCTCAG	ACACACAGTACGTCTTGGAGTCCG	tag160923
novel_sir2506	GATTCACCGGACTACAAGCCTCAG	AAGTGGCCTGATGTTCGGAGTCGG	tag9269
novel_sir2507	ACAAAAGTTCGCCGCGCGAGTCGC	ACTGTTTTCAAGCGGCGCGCTCAG	tag23417
novel_sir2508	GATGCACCGTTATAACTGGCTCAG	ACGTGGCAATATTGACCGAGTCTT	tag20896
novel_sir2509	CGGTACAGTCTACTTCTGGCTCAG	CATGTCAGATGAAGACCGAGTCAA	tag225549
novel_sir2510	CGGTGCAGTCTACTTCTGGCTCAG	CACGTCAGATGAAGACCGAGTCAA	tag247025
novel_sir2511	GATGGGACAACAGCTGATGCTCAG	ACCCTGTTGTCGACTACGAGTCAG	tag156925
novel_sir2512	GATGGGACAACAGCTGATGCTCAG	ACCCTGTTGTCGACTACGAGTCAA	tag156925
novel_sir2513	TATGTGTGTAATGCAGAATCTCAG	ACACACATTACGTCTTAGAGTCCG	tag266156
novel_sir2514	GATCCGGGCAATCTCTACTCTCAG	AGGCCCGTTAGAGATGAGAGTCAT	tag57306
novel_sir2515	ACGAGTCGTAGCTGAGTCAGTCGA	TGTGCTCAGCATCGACTCAGTCAG	tag15741

novel_sir2516	CCGAATCAAACACCGAGTCAG	CTTAGTTTGTGGCTCAGTCTG	tag253290
novel_sir2517	GAGCATCACCAGCTACGTCAG	CGTAGTGGTCGATGCAGTCCT	tag228326
novel_sir2518	AAACAGGTCTTCGCTAGCAGTCGC	CATTTGTCCAGAAGCGATCGTCAG	tag121418
novel_sir2519	ACATACGCGTGGCAGTCCAGTCAG	ACTGTATGCGCACCGTCAGGTCAG	tag18880
novel_sir2520	CCCTGCGGAGGCGTTGGTGGTCAG	GACGCCTCCGCAACCACCAGTCGA	tag31191
novel_sir2521	AGCCACTTATACACCAGTCGA	TTTCGGTGAATATGTGGTCAG	tag100160
novel_sir2522	CCCGTCGCGTATTTAAGTCGT	CGGGGCAGCGCATAAATTCAG	tag59385
novel_sir2523	CCCCGTCGCGTATTTAAGTCGT	ACGGGGCAGCGCATAAATTCAG	tag112742
novel_sir2524	ATGCCCCGTCGCGTATTTAAGTCGT	GTTACGGGGCAGCGCATAAATTCAG	tag267533
novel_sir2525	AGGGAGGCAAAATGTTACATTCAG	CCTCCGTTTTACAATGTAAGTCAT	tag195413
novel_sir2526	GAGGCAAAGTGTTACATTCAG	CCGTTTCACAATGTAAGTCAT	tag50931
novel_sir2527	AGGAGGCAAAGTGTTACATTCAG	CTCCGTTTCACAATGTAAGTCAT	tag112550
novel_sir2528	GGGAGGCAAAGTGTTACATTCAG	CTCCGTTTCACAATGTAAGTCAT	tag254047
novel_sir2529	AAGGAGGCAAAGTGTTACATTCAG	CCTCCGTTTCACAATGTAAGTCAT	tag32529
novel_sir2530	AGGGAGGCAAAGTGTTACATTCAG	CCTCCGTTTCACAATGTAAGTCAT	tag40839
novel_sir2531	AAGGAGGCAAAGTGTTACATTCAG	CCTCCGTTTCACAATGTAAGTCAA	tag32529
novel_sir2532	AGGGAGGCAAAGTGTTACATTCAG	CCTCCGTTTCACAATGTAAGTCAA	tag40839
novel_sir2533	CCTCCGTTTCACAATGTAAGTCGT	AAGGAGGCAAAGTGTTACATTCAG	tag32529
novel_sir2534	ATAAACTACATCAGCATAAGTCGA	CTTATTTGATGTAGTCGTATTCAG	tag75301
novel_sir2535	ACACACATTGCGTCTTGAAGTCCA	TATGTGTGTAACGCAGAACTTCAG	tag52603
novel_sir2536	TATGTGTGTAACGCAGAACTTCAG	ACACACATTGCGTCTTGAAGTCCG	tag52603
novel_sir2537	ACACACGTTACGTCTTGAAGTCCG	TATGTGTGCAATGCAGAACTTCAG	tag6613
novel_sir2538	TATTTGTGTAATGCAGAACTTCAG	AAACACATTACGTCTTGAAGTCCG	tag32345
novel_sir2539	GGGAGGCATGAGCATTTCCTTCAG	CTCCGTACTCGTAAAGGAAGTCGT	tag216526
novel_sir2540	AGGATCCTCTGTAGCCGAAGTCAC	GGTCCTAGGAGACATCGGCTTCAG	tag164661
novel_sir2541	ACGCTGTTGGCTACTAGAAGTCAG	GATGCGACAACCGATGATCTTCAG	tag74769
novel_sir2542	GGGGGCTGCAGCGCTCTTCAG	CCCGACGTCGCGAGAAGTCCA	tag79961
novel_sir2543	AGGAATCCCGATTTGTCAAGTCTC	ACTCCTTAGGGCTAAACAGTTCAG	tag242602
novel_sir2544	AGGAATCCCGATTTGTCAAGTCTA	ACTCCTTAGGGCTAAACAGTTCAG	tag242602
novel_sir2545	CATCCGTGGTGCCAGATAGTTCAG	AGGCACCACGGTCTATCAAGTCTT	tag188308
novel_sir2546	TCTGGCTAATAACGTTCCGTTCAG	ACCGATTATTGCAAGGCAAGTCAC	tag292258
novel_sir2547	AGTCCACTACTGGCTCCAAGTCAT	TATCAGGTGATGACCGAGGTTCAG	tag196491
novel_sir2548	TATGTGGGTAATGCAAAATTTCAG	ACACCCATTACGTTTTAAGTCCG	tag282632
novel_sir2549	ACGCACAATGTGTCTTAAAGTCCA	TATGCGTGTTACACAGAATTTCAG	tag271815

novel_sir2550	GATGCCAAGGAGACTACATTTCAG	ACGGTTCCTCTGATGTAAAGTCAG	tag74805
novel_sir2551	CTCCGTACTCGTAAAGAAAGTCGT	GGGAGGCATGAGCATTTCTTTCAG	tag170185
novel_sir2552	CAGGTAACATCGGATTTTCAG	CCATTGTAGCCTAAAAGTCCA	tag85596
novel_sir2553	AGTGCCACGTAAACTAAAAGTCCT	TATCACGGTGCATTTGATTTTCAG	tag157632
novel_sir2554	CCATTGCAGCTCAAAAGTCCA	CGGGTAACGTCGAGTTTTCAG	tag150058
novel_sir2555	GAACCCTCGGTAAACAAAAGTCTA	AGCTTGGGAGCCATTTGTTTTCAG	tag182001
novel_sir2556	ACTAGTTTGCAACCAATTTTTCAG	ATCAAACGTTGGTTAAAAAGTCAA	tag258584
novel_sir2557	AGTTTGTACACTCTTTTTCAG	AAACATGTGAGAAAAAGTCAA	tag3431
novel_sir2558	ATGACGCCGTTGATTTTTTCTCAC	TCTACTGCGGCAACTAAAAAAGAG	tag215830
novel_sir2559	TCTACTGCGGCAACTGAAAAAGAG	ATGACGCCGTTGACTTTTTCTCGC	tag203226
novel_sir2560	TCTACTGCGGCAACTGAAAAAGAG	ATGACGCCGTTGACTTTTTCTCAC	tag203226
novel_sir2561	TCTACTGCGGCAACTGAAAAAGAG	ATGACGCCGTTGACTTTTTCTCAT	tag203226
novel_sir2562	ATGACACCGTTGACTTTTTCTCAC	TCTACTGTGGCAACTGAAAAAGAG	tag245967
novel_sir2563	ATGACGCCATTGACTTTTTCTCAC	TCTACTGCGGTAACTGAAAAAGAG	tag134740
novel_sir2564	TCTACTGCGGCAATTGAAAAAGAG	ATGACGCCGTTAACTTTTTCTCAC	tag230882
novel_sir2565	AGCTTCTCACCGGTTGTTTCTCAG	GATCGAAGAGTGGCCAACAAAGAG	tag28534
novel_sir2566	TGCTTAGAGCCTGCAGGTAAAGAG	GAATCTCGGACGTCCATTTCTCGA	tag85109
novel_sir2567	TCGCATGGCTGTTCCCGAAGAG	CGTACCGACAAGGGCTTCTCTT	tag163590
novel_sir2568	GTTCGCATGGCTGTTCCCGAAGAG	AGCGTACCGACAAGGGCTTCTCTT	tag60856
novel_sir2569	TAGGTCGCCTCCCGGTAAGAG	CCAGCGGAGGGCCATTCTCGG	tag101310
novel_sir2570	ACCTGTCTAAATCCTTTGTCTCCA	CTTGGACAGATTTAGGAAACAGAG	tag369
novel_sir2571	CTTGGACAGATTTAGGAAACAGAG	ACCTGTCTAAATCCTTTGTCTCCG	tag369
novel_sir2572	ACCTGTCTAAATCCTTTGTCTCTG	CTTGGACAGATTTAGGAAACAGAG	tag369
novel_sir2573	CTTGGACATATTTAGGAAACAGAG	ACCTGTATAAATCCTTTGTCTCCA	tag207183
novel_sir2574	CGTTACACCGCGAGTACAGAG	AATGTGGCGCTCATGTCTCCA	tag74871
novel_sir2575	CATGATGCTTAGACCTGTACAGAG	ACTACGAATCTGGACATGTCTCAT	tag7267
novel_sir2576	CATGATGCTTAGACCTGTACAGAG	ACTACGAATCTGGACATGTCTCCA	tag7267
novel_sir2577	CGCCGCCGGCCTTGGTCTCCG	CGGCGGCGGCCGGAACCAGAG	tag288476
novel_sir2578	TGTTACCAACAGTTATCACCAGAG	AATGGTTGTCAATAGTGGTCTCTT	tag84095
novel_sir2579	AAGCCATCTTGCGCCCAGAG	CGGTAGAACGCGGGTCTCCA	tag263295
novel_sir2580	AGTCAAGCCATCTTGCGCCCAGAG	AGTTCGGTAGAACGCGGGTCTCCA	tag160645
novel_sir2581	AGCTAGCCGCCATCGTCGTCTCTG	TGTCGATCGGCGGTAGCAGCAGAG	tag80138
novel_sir2582	AGGCCCTGCCACGTCGCGTCTCGC	ACTCCGGGACGGTGCAGCGCAGAG	tag28522
novel_sir2583	CGGAGCAGCGGCGAGGCAGAG	CTCGTCGCCGCTCCGTCTCGA	tag81361

novel_sir2584	GATGAAGCACGACGGGCAGAG	ACTTCGTGCTGCCCGTCTCAG	tag264891
novel_sir2585	ATTATCAGTCTGGACGAGTCTCCT	CATAATAGTCAGACCTGCTCAGAG	tag204921
novel_sir2586	CGCGGCTCAGCTTCAGTCTCTA	CAGCGCCGAGTCGAAGTCAGAG	tag213144
novel_sir2587	AAGGGAGGAAGGTTTTCAGAG	CCCTCCTTCCAAAAGTCTCAG	tag141035
novel_sir2588	GAGGGAGGAAGGTTTTCAGAG	CCCTCCTTCCAAAAGTCTCAG	tag253684
novel_sir2589	CTGTAGCCCGCTTTCTTCTCTCTC	TAGACATCGGGCGAAAGAAGAGAG	tag273878
novel_sir2590	ACCGCTCGGCCATCTCTCCT	CTTGGCGAGCCGGTAGAGAG	tag231373
novel_sir2591	CGCTCCACCGGCCGCTCTCCG	TCGCGAGGTGGCCGGCGAGAG	tag146991
novel_sir2592	CGGCGGCCGGAGGGGAGGAGAG	CGCCGGCCTCCCCTCCTCTCAG	tag150440
novel_sir2593	CGGCCGCGGAAAAAGGGAGAG	CGGCGCCTTTTTCCCTCTCCG	tag6798
novel_sir2594	CCGGCCGCGGAAAAAGGGAGAG	CCGGCGCCTTTTTCCCTCTCCG	tag205825
novel_sir2595	CCGCAGCCAGCGAGGGAGAG	CGTCGGTCGCTCCCTCTCCC	tag103485
novel_sir2596	ACCGCGTTTGGGCAACCCTCTCAA	TATGGCGCAAACCCGTTGGGAGAG	tag171408
novel_sir2597	AGCCACCGCCGCCACCTCTCGT	GTTCGGTGGCGGCGGTGGAGAG	tag230090
novel_sir2598	TGTCTAGATATCCGGCGATGAGAG	AGATCTATAGGCCGCTACTCTCCT	tag92742
novel_sir2599	CGACGCGGGGCTGGATCTCAG	ACGCTGCGCCCCGACCTAGAG	tag6397
novel_sir2600	AACTTTTTAGTCACATCATCTCTA	TGTTGAAAAATCAGTGTAGTAGAG	tag255454
novel_sir2601	TGTTGAAAAGTCAGTGTAGTAGAG	AACTTTTCAGTCACATCATCTCTA	tag204594
novel_sir2602	AACTTTTCAGTCACATCATCTCCA	TGTTGAAAAGTCAGTGTAGTAGAG	tag204594
novel_sir2603	TGGGGGCTGAAGCAGGTAGAG	CCCCGACTTCGTCCATCTCCA	tag209981
novel_sir2604	GAGAGATATTTTGTACATGTAGAG	CTCTATAAAACATGTACATCTCAG	tag141339
novel_sir2605	GATAGATATTTTGTACATGTAGAG	ATCTATAAAACATGTACATCTCAG	tag23460
novel_sir2606	CCGGGACACGGTACTGTAGAG	CCCTGTGCCATGACATCTCGA	tag29289
novel_sir2607	ACAACTCTCATCGGCTAATCTCAG	ACTGTTGAGAGTAGCCGATTAGAG	tag261375
novel_sir2608	CTGAAAGTTTTGGTACACGAG	CTTTCAAAACCATGTGCTCAC	tag276198
novel_sir2609	CCTGCTTCGTCTGCTGCTCCG	ATGGACGAAGCAGACGACGAG	tag50579
novel_sir2610	ATAAATCTATAGGCCGCTGCTCTT	GATATTTAGATATCCGGCGACGAG	tag257429
novel_sir2611	GATGTCTAGACATCGGGCGACGAG	ACAGATCTGTAGCCCGCTGCTCTT	tag203455
novel_sir2612	GATGTTTAGACATCGGGCGACGAG	ACAAATCTGTAGCCCGCTGCTCTT	tag247188
novel_sir2613	ACAAATCTGTAGCCCGCTGCTCTC	GATGTTTAGACATCGGGCGACGAG	tag247188
novel_sir2614	TCGCCTGATCGCCCGGATGCCGAG	CGGACTAGCGGGCCTACGGCTCCA	tag24613
novel_sir2615	TAGCCAGCGGGCTGCGCATCCGAG	CGGTCGCCCGACGCGTAGGCTCCT	tag276455
novel_sir2616	AAGCCAGCGGGCTGCGCATCCGAG	CGGTCGCCCGACGCGTAGGCTCCT	tag286533
novel_sir2617	CTGGTCCTGTACCGGCGCGAG	CCAGGACATGGCCGCGCTCGC	tag105965

novel_sir2618	GGGCGGCGGCGCAGCGGCGAG	CGCCGCCGCGTCGCCGCTCCG	tag162017
novel_sir2619	ATTAAACTTCCTACAAACGCTCCT	GGTAATTTGAAGGATGTTTGCGAG	tag19737
novel_sir2620	ATTAAACTTCCTACAAACGCTCTC	GGTAATTTGAAGGATGTTTGCGAG	tag19737
novel_sir2621	CGCGACTTCGTGGTTTTAGCTCTA	TAGCGCTGAAGCACCAAAATCGAG	tag89355
novel_sir2622	CTGAAACTGTTTGGCTGAGCTCCA	TCGACTTTGACAAACCGACTCGAG	tag269456
novel_sir2623	CGATGTCGTAGGAGAGCTCGC	GAGCTACAGCATCCTCTCGAG	tag214989
novel_sir2624	GTGGTCGAGGCCTAGGTCGAG	CCAGCTCCGGATCCAGCTCCT	tag194162
novel_sir2625	CCGGCCGGCGGAGCTTCGAG	CCGGCCGCCTCGAAGCTCCC	tag145283
novel_sir2626	GCGCCGGCCGGCGGAGCTTCGAG	CGGCCGGCCGCCTCGAAGCTCCC	tag82060
novel_sir2627	CCTCCGCGCCGGCCCCAAGGAG	AGGCGCGGCCGGGGTTCCTCAC	tag223820
novel_sir2628	CCTCCTGGCATCGTTCCTCGC	AGGGAGGACCGTAGCAAGGAG	tag63153
novel_sir2629	CGCTTACTAGGCATTCCTCGT	GCGCGAATGATCCGTAAGGAG	tag217363
novel_sir2630	GTTGGGATCCGGTCCTTGCAGGAG	ACCCTAGGCCAGGAACGTCCTCTC	tag118348
novel_sir2631	CCGTCCGCCGCCGCTCCTCCA	GCGGCAGGCGGCGGCGAGGAG	tag270610
novel_sir2632	CCGACTCGAGGTGGCGGAGGAG	CTGAGCTCCACCGCCTCCTCGC	tag211849
novel_sir2633	AGCTCGCGACGTCATCCTCTA	ACTCGAGCGCTGCAGTAGGAG	tag245095
novel_sir2634	GAGCTCGCGACGTCATCCTCTA	AACTCGAGCGCTGCAGTAGGAG	tag266034
novel_sir2635	TTTGTCAAATACACAGTGTAGGAG	ACAGTTTATGTGTCACATCCTCCA	tag211882
novel_sir2636	CCGAAAGTCTAGTTTGCCTCCC	TCGGCTTTCAGATCAAACGGAG	tag172660
novel_sir2637	TATTAAGATAAAATCAGGACGGAG	AATTCTATTTTAGTCCTGCCTCCT	tag195978
novel_sir2638	CGGCGGCACGCGGTGACGGAG	CGCCGTGCGCCACTGCCTCTC	tag41154
novel_sir2639	CTGTTCTACATGATGCCTCTA	AGGACAAGATGTACTACGGAG	tag74626
novel_sir2640	GCGGGCGAGCGGAAGCCGGAG	CCCGCTCGCCTTCGGCCTCCG	tag77714
novel_sir2641	GGTCAGGTTCGGCGGCCGGAG	AGTCCAAGCCGCCGGCCTCCC	tag257412
novel_sir2642	GGGTCAGGTTCGGCGGCCGGAG	CAGTCCAAGCCGCCGGCCTCCC	tag142640
novel_sir2643	GGTGTAGTCGTCTGGCCGGAG	ACATCAGCAGACCGGCCTCTC	tag53075
novel_sir2644	GACAAGTTGGTATCAGAGCCTCCT	CACTGTTCAACCATAGTCTCGGAG	tag286301
novel_sir2645	TGTTGCTGAACCCAAATTTCGGAG	AACGACTTGGGTTTAAAGCCTCAC	tag222694
novel_sir2646	CACTAGGCACCGTTCCCTCCC	GCGTGATCCGTGGCAAGGGAG	tag113586
novel_sir2647	CCGCACTAGGCACCGTTCCCTCCC	ACGGCGTGATCCGTGGCAAGGGAG	tag59193
novel_sir2648	CCGACGACGCGCGTCCCTCAC	GCGGCTGCTGCGCGCAGGGAG	tag293808
novel_sir2649	AGGCACCGTTCCCTCCCTCCC	GATCCGTGGCAAGGGAGGGAG	tag164767
novel_sir2650	TTTGTGAATTATCGGAGTAGGGAG	ACACTTAATAGCCTCATCCCTCTA	tag16243
novel_sir2651	CCCGCACGACGTCGCCCTCTC	GCGGGCGTGCTGCAGCGGGAG	tag225404

novel_sir2652	GATAAGCTTATCGGCGCGGGAG	ATTCGAATAGCCGCGCCCTCGG	tag293383
novel_sir2653	CCGTCGCGGCGCCGCCCTCCT	GCGGCAGCGCCGCGGCGGGAG	tag231257
novel_sir2654	AAGTAAAACATCGAACGCCCTCTA	AGTTCATTTTGTAGCTTGCGGGAG	tag76122
novel_sir2655	CGTCGCCGCCGCTTCCCCTCGC	CGGCAGCGGCGGCGAAGGGGAG	tag270470
novel_sir2656	AGGTTCGGCGGCCGGAGGGGAG	CAAGCCGCCGGCCTCCCCTCCT	tag108175
novel_sir2657	ATAATTCAACCGGACCACCCTCTA	GGTATTAAGTTGGCCTGGTGGGAG	tag47652
novel_sir2658	ACCATTGACCAAGGCTGACCTCAA	AGTGGTAACTGGTTCCGACTGGAG	tag242484
novel_sir2659	CTTGGCTATGGTTAGGACCTCAT	TGGAACCGATACCAATCCTGGAG	tag162141
novel_sir2660	CCTTGGCTATGGTTAGGACCTCAT	ATGGAACCGATACCAATCCTGGAG	tag145455
novel_sir2661	GAGGAACTTGCGGCGCTGGAG	CCTTGAACGCCGCGACCTCTG	tag10022
novel_sir2662	CTTAGATAGGCCGGCCGACCTCTC	GGGAATCTATCCGGCCGGCTGGAG	tag41467
novel_sir2663	CGCGCTCGCCGACGACCTCCT	TCGCGCGAGCGGCTGCTGGAG	tag145890
novel_sir2664	GGTGGCAGAGCGTCAGGTCTGGAG	ACCGTCTCGCAGTCCAGACCTCGA	tag150957
novel_sir2665	GAGGGGTGCGCGGCAGTGGAG	CCCCACGCGCCGTCACCTCTC	tag291918
novel_sir2666	AGGGAGGGGTGCGCGGCAGTGGAG	CCTCCCCACGCGCCGTCACCTCTC	tag119420
novel_sir2667	GCGCCGGCCCCAAGGAGTGGAG	CGGCCGGGGTTCCTCACCTCCG	tag250858
novel_sir2668	CAAGCCACCGCCGCCACCTCTC	AGGTTCGGTGGCGGCGGTGGAG	tag56761
novel_sir2669	GATGACGACAATCGCGATGAG	ACTGCTGTTAGCGCTACTCCT	tag136027
novel_sir2670	CACTGAGGCACACGGTTCGATGAG	GACTCCGTGTGCCAAGCTACTCCC	tag155047
novel_sir2671	GGGCTGCTCGAGGTGGATGAG	CGACGAGCTCCACCTACTCCC	tag76106
novel_sir2672	CGGCCAAGGCTATATACTCGT	CAGCCGGTTCCGATATATGAG	tag165172
novel_sir2673	CAGCCTTGCGACCATACTCCC	AAGTCGGAACGCTGGTATGAG	tag241953
novel_sir2674	AGGACTAGGTGTACAGTAACTGAG	CTGATCCACATGTCATTGACTCAT	tag89210
novel_sir2675	CTGACCCACATGTCATTGACTCAT	GGGACTGGGTGTACAGTAACTGAG	tag55632
novel_sir2676	CGGATCGGCCCGAGGGACTCGG	GGGCCTAGCCGGGCTCCCTGAG	tag88827
novel_sir2677	GATCTATGAGTGAGGTTGTCTGAG	AGATACTCACTCCAACAGACTCTC	tag16232
novel_sir2678	TCGACGGCAGAGGCGGGGTGAG	CTGCCGTCTCCGCCCCACTCGC	tag186885
novel_sir2679	GTGCTTTTTGTAACGAGTTGAG	CGAAAAACATTGCTCAACTCGC	tag245075
novel_sir2680	CTGACGCCGTTGACTTTTTATCAC	CAGACTGCGGCAACTGAAAAATAG	tag115915
novel_sir2681	TGTGCACTAGGGTATATCAAATAG	ACGTGATCCCATATAGTTTATCGG	tag111208
novel_sir2682	GAGAACGGTGGGCTATAGCAATAG	CTTGCCACCCGATATCGTTATCTC	tag172768
novel_sir2683	GGTGGCGAGAGCATGGGTGAATAG	ACCGCTCTCGTACCCACTTATCAG	tag248007
novel_sir2684	TGGATCTTGGCTTGCCCTACATAG	CTAGAACCGAACGGGATGTATCCT	tag198377
novel_sir2685	CGTGACAAAGGACCCTGACCATAG	ACTGTTTCCTGGGACTGGTATCGA	tag204020

novel_sir2686	ACTGTTTCCTGGGACTGGTATCGG	CGTGACAAAGGACCCTGACCATAG	tag204020
novel_sir2687	ACCCAATGACTAGGATGGTATCCG	GTTGGGTTACTGATCCTACCATAG	tag43644
novel_sir2688	GTTGGGTTACTGATCCTACCATAG	ACCCAATGACTAGGATGGTATCCA	tag43644
novel_sir2689	CCGGAAGAGAGAGCCAGATAG	CCTTCTCTCTCGGTCTATCCC	tag56830
novel_sir2690	ACGGAAGAGAGAGCCAGATAG	CCTTCTCTCTCGGTCTATCCC	tag176587
novel_sir2691	CCAGGGCGGACTTGCTATCTA	CCGGTCCCGCCTGAACGATAG	tag29653
novel_sir2692	CCGGTCTCGCCTGAACGATAG	CCAGAGCGGACTTGCTATCCA	tag163666
novel_sir2693	TGGCACTTTCGCACCGGATAG	CGTGAAAGCGTGGCCTATCGA	tag277709
novel_sir2694	TTGGCACTTTCGCACCGGATAG	CCGTGAAAGCGTGGCCTATCGA	tag272298
novel_sir2695	TTTACAAGAGAGGCGTCCGGATAG	ATGTTCTCTCCGCAGGCCTATCGA	tag94498
novel_sir2696	ACCGCTTATGGGCCGACCTATCAA	GGTGGCGAATACCCGGCTGGATAG	tag124201
novel_sir2697	CGGGGGCTACAGATGTTATATCAT	CAGCCCCCGATGTCTACAATATAG	tag98549
novel_sir2698	CATTCGTGTGGCCATATATATCGA	AAGTAAGCACACCGGTATATATAG	tag116841
novel_sir2699	TAGGATGATGCTTAGCCTATATAG	CCTACTACGAATCGGATATATCGC	tag259349
novel_sir2700	AACACGGTCTACAAGATATATCTT	TATTGTGCCAGATGTTCTATATAG	tag207566
novel_sir2701	GTTGGCTAGCTCATGTTTATATAG	ACCGATCGAGTACAAATATATCAC	tag212255
novel_sir2702	TTTTATTCACATCGGCACCTATAG	AATAAGTGTAGCCGTGGATATCCG	tag60797
novel_sir2703	GGTTCTGTTCTGCGGCCTATAG	AAGACAAGACGCCGGATATCGA	tag104633
novel_sir2704	TGTAAGCTGCAAACTGGCCTATAG	ATTCGACGTTTGACCGGATATCAA	tag76935
novel_sir2705	CTTATCGTGACAAGTGGCCTATAG	ATAGCACTGTTCACCGGATATCCG	tag253673
novel_sir2706	GAACTGGCTTTGTCATCATATCAG	TCCTTGACCGAAACAGTAGTATAG	tag176133
novel_sir2707	ACCATCCGTAGCGCGCCATATCGG	GTTGGTAGGCATCGCGCGGTATAG	tag244385
novel_sir2708	ATTAACTCTCTTGCCACATATCAG	AGTAATTGAGAGAACGGTGTATAG	tag128445
novel_sir2709	TCTTATTCACATCGGCACTTATAG	AATAAGTGTAGCCGTGAATATCCG	tag164506
novel_sir2710	ACCTAAGCGTCATGTCAATATCAT	GGTGGATTCGCAGTACAGTTATAG	tag48166
novel_sir2711	CGGTCATTCGCGGTGCAGTTATAG	CAGTAAGCGCCACGTCAATATCAC	tag34881
novel_sir2712	ACTTTTTCTCACATGTTTGATCAT	ACTGAAAAAGAGTGTACAAACTAG	tag95167
novel_sir2713	ACTTTTTGTCTAACGTTTGATCAT	ACTGAAAAACAGATTGCAAACTAG	tag289746
novel_sir2714	ACTGAAAAATAGGTTGCAAACTAG	ACTTTTTATCCAACGTTTGATCAT	tag84467
novel_sir2715	ATTACCGCGCCTAACTTTGATCAT	GGTAATGGCGCGGATTGAAACTAG	tag74474
novel_sir2716	AGGTCCTCCACTTCCAACTAG	CAGGAGGTGAAGGTTGATCAA	tag176206
novel_sir2717	GGTCAGCGGTGGATTGGCAACTAG	AGTCGCCACCTAACCGTTGATCTG	tag47826
novel_sir2718	CAGACATCGTCGTTAGACTAG	CTGTAGCAGCAATCTGATCTC	tag33452
novel_sir2719	ACGCAGACATCGTCGTTAGACTAG	CGTCTGTAGCAGCAATCTGATCTC	tag91365

novel_sir2720	TATCAGGTGATATCAGGTGACTAG	AGTCCACTATAGTCCACTGATCTT	tag185760
novel_sir2721	CGGGCGCCGACCGGCAATGATCAC	GTGCCCGCGGCTGGCCGTTACTAG	tag266022
novel_sir2722	TTTGAACCTTTGATGGCAACCTAG	ACTTGGAAACTACCGTTGGATCGA	tag232490
novel_sir2723	GATCGTCACTGACTGTCCACCTAG	AGCAGTGACTGACAGGTGGATCCG	tag147802
novel_sir2724	CGCCGACAGGAGCTGGATCCG	CTGCGGCTGTCCTCGACCTAG	tag714
novel_sir2725	GCTGCGGCTGTCCTCGACCTAG	ACGCCGACAGGAGCTGGATCCG	tag184763
novel_sir2726	AAACACATTCAAAAACTGGATCGC	GGTTTGTGTAAGTTTTTGACCTAG	tag193262
novel_sir2727	AAACACACTCAAAAACTGGATCGC	GGTTTGTGTGAGTTTTTGACCTAG	tag252352
novel_sir2728	AATGTCTTCCGCCCGGATCGG	TGTTACAGAAGGCGGGCCTAG	tag88718
novel_sir2729	CAATGTCTTCCGCCCGGATCGG	CTGTTACAGAAGGCGGGCCTAG	tag213657
novel_sir2730	CAGGTTAAAAATGTGGATGCCTAG	CCAATTTTTACACCTACGGATCAG	tag293201
novel_sir2731	CCCTTCAGCCGTTTTGCCTAG	GAAGTCGGCAAAACGGATCCG	tag131155
novel_sir2732	CCTACCGTCGAGTCCTAG	ATGGCAGCTCAGGATCAA	tag11583
novel_sir2733	CCTACCGTCGAGTCCTAG	ATGGCAGCTCAGGATCAG	tag11583
novel_sir2734	CGTGGATGGCAGCTCAGGATCAG	AAGCACCTACCGTCGAGTCCTAG	tag132101
novel_sir2735	CGTGGATGGCAGCTCAGGATCAG	ACGCACCTACCGTCGAGTCCTAG	tag292806
novel_sir2736	CCGCACCTACCGTCGAGTCCTAG	CGTGGATGGCAGCTCAGGATCAA	tag27184
novel_sir2737	AAGCACCTACCGTCGAGTCCTAG	CGTGGATGGCAGCTCAGGATCAA	tag132101
novel_sir2738	ACGCACCTACCGTCGAGTCCTAG	CGTGGATGGCAGCTCAGGATCAA	tag292806
novel_sir2739	CGTGGATGGCAGCTCAGGATCAC	AAGCACCTACCGTCGAGTCCTAG	tag132101
novel_sir2740	CGTGGATGGCAGCTCAGGATCAC	ACGCACCTACCGTCGAGTCCTAG	tag292806
novel_sir2741	ACACTTCCGTCAATTCTCGATCGT	ATTGTGAAGGCAGTTAAGAGCTAG	tag64323
novel_sir2742	CACTTTCGCACCGGATAGCTAG	GAAAGCGTGGCCTATCGATCCT	tag245041
novel_sir2743	TGGTTGGCCCTGATTTCTAGCTAG	CAACCGGGACTAAAGATCGATCTT	tag117036
novel_sir2744	TTTGAACATCGGGAGCTGCGCTAG	ACTTGTAGCCCTCGACGCGATCTA	tag275018
novel_sir2745	GGGCCCTGATTTCTATCGCTAG	CGGGACTAAAGATAGCGATCTT	tag281097
novel_sir2746	TTGGCCCTGATTTCTATCGCTAG	CCGGGACTAAAGATAGCGATCTT	tag141681
novel_sir2747	TGGGCCCTGATTTCTATCGCTAG	CCGGGACTAAAGATAGCGATCTT	tag187442
novel_sir2748	GTGGGCCCTGATTTCTATCGCTAG	CCCGGGACTAAAGATAGCGATCTT	tag245881
novel_sir2749	CTTCTACTTGCCCCGATTCGCTAG	AGATGAACGGGGCTAAGCGATCTG	tag72787
novel_sir2750	ACTCGACGTTAATCCTCAGGCTAG	AGCTGCAATTAGGAGTCCGATCGT	tag266617
novel_sir2751	CATAGGGCTATGATGCTATGCTAG	ATCCCGATACTACGATACGATCCT	tag39307
novel_sir2752	TTTAAAGTTTATTCTGCCTGCTAG	ATTTCAAATAAGACGGACGATCAA	tag261933
novel_sir2753	ATCTCTGAACTCCGTCTGTGCTAG	GAGACTTGAGGCAGACACGATCAC	tag54897

novel_sir2754	CGTATATAGGATCATGGTTGCTAG	ATATATCCTAGTACCAACGATCCC	tag10761
novel_sir2755	GAGAACCGGTTGGTGTGCATCTAG	CTTGGCCAACCACACGTAGATCGG	tag167008
novel_sir2756	CTCTTTTCGTCGCCTGATATCTAG	GAAAAGCAGCGGACTATAGATCTT	tag36749
novel_sir2757	CTCTTTTCGTCGCCCGATGTCTAG	GAAAAGCAGCGGGCTACAGATCTG	tag265336
novel_sir2758	GGCTTGGCCTCATCTCTATTCTAG	GAACCGGAGTAGAGATAAGATCTA	tag74760
novel_sir2759	CGGCCGGTTGGCGTACACTTCTAG	CGGCCAACCGCATGTGAAGATCGA	tag155969
novel_sir2760	GGTTAGCGACTGAACTACTTCTAG	AATCGCTGACTTGATGAAGATCGG	tag77648
novel_sir2761	ATGAAAAGCTAGCCGGAAGATCCC	AATACTTTTCGATCGGCCTTCTAG	tag201849
novel_sir2762	AATGCCACGTAGGACGAAGATCTA	AGTTACGGTGCATCCTGCTTCTAG	tag174046
novel_sir2763	AGTGAACGGTGTAGTAAAAAGTAG	ACTTGCCACATCATTTTTCATCCT	tag12455
novel_sir2764	AGTGAACGGTGTAGTAAAAAGTAG	ACTTGCCACATCATTTTTCATCTT	tag12455
novel_sir2765	CTGGAACGTTCTGAAAAGTAG	CCTTGCAAGACTTTTCATCGA	tag144117
novel_sir2766	AGATCGCTTAGCCCCGTTCATCTT	CGTCTAGCGAATCGGGGCAAGTAG	tag84338
novel_sir2767	CTCCCTGGCACTATTCATCTT	CGGAGGGACCGTGATAAGTAG	tag2426
novel_sir2768	CGCTTAACTATCATTCATCGA	TGGCGAATTGATAGTAAGTAG	tag138076
novel_sir2769	GAACTTGTCATAGTGTGTCATCTA	GGCTTGAACAGTATCACACAGTAG	tag275308
novel_sir2770	GATTTGAACATGGCGACACAGTAG	AAACTTGTACCGCTGTGTCATCCC	tag283266
novel_sir2771	GATTTGAACATGGCGACACAGTAG	AAACTTGTACCGCTGTGTCATCGC	tag283266
novel_sir2772	GATTTGAACATGGCGACACAGTAG	AAACTTGTACCGCTGTGTCATCTC	tag283266
novel_sir2773	ACTATCGCGGCACGGTCATCAG	AATGATAGCGCCGTGCCAGTAG	tag13
novel_sir2774	CTTTTTCAGTTGCCGCAGTAG	AAAAGTCAACGGCGTCATCTA	tag60983
novel_sir2775	CTCTTTTTCAGTTGCCGCAGTAG	GAAAAAGTCAACGGCGTCATCTA	tag57366
novel_sir2776	ACTCTTTTTCAGTTGCCGCAGTAG	AGAAAAAGTCAACGGCGTCATCTA	tag141941
novel_sir2777	GCTCTTTTTCAGTTGCCGCAGTAG	AGAAAAAGTCAACGGCGTCATCTA	tag184516
novel_sir2778	ATTCTTTTTCAGTTGCCGCAGTAG	AGAAAAAGTCAACGGCGTCATCTA	tag279211
novel_sir2779	ACTCTTTTTTAGTTGCCGCAGTAG	AGAAAAAATCAACGGCGTCATCTA	tag175739
novel_sir2780	GAGAGCAGCGGCCGGCAGTAG	CTCGTCGCCGGCCGTCATCCG	tag124028
novel_sir2781	ACGGATCATTCGCGCTCAGTAG	CCTAGTAAGCGCGAGTCATCAG	tag49486
novel_sir2782	CTTACTCTCGACTGTCATCATCTT	CAGAATGAGAGCTGACAGTAGTAG	tag24618
novel_sir2783	ACAACTTTTCAGTCACATCATCTT	TGTGTTGAAAAGTCAGTGTAGTAG	tag45710
novel_sir2784	TGTGTTGAAAAGTCAGTGTAGTAG	ACAACTTTTCAGTCACATCATCTC	tag45710
novel_sir2785	TGGTTGGCCCTGATTGCTACGTAG	CAACCGGGACTAACGATGCATCTT	tag126759
novel_sir2786	ACAAATTGCACCGGCTAGCATCAT	CGTGTTTAACGTGGCCGATCGTAG	tag63753
novel_sir2787	CGGAGTGCACCGATCCTCTCGTAG	CTCACGTGGCTAGGAGAGCATCCT	tag119108

novel_sir2788	AATCATTATCGGCTGCAGCATCTC	TCTTAGTAATAGCCGACGTCGTAG	tag5069
novel_sir2789	GATCTGGGTTCGAAGCTCCATCCC	AGCTAGACCCAAGCTTCGAGGTAG	tag200292
novel_sir2790	GGTGAGCTCGATAATCTTAGGTAG	ACTCGAGCTATTAGAATCCATCCC	tag40585
novel_sir2791	GACTAGGACTCGACGGTAG	GATCCTGAGCTGCCATCCA	tag6389
novel_sir2792	AACTAGGACTCGACGGTAG	GATCCTGAGCTGCCATCCA	tag126073
novel_sir2793	ACTCAAAAACTGGATCGCCATCTC	TGTGAGTTTTTGACCTAGCGGTAG	tag220121
novel_sir2794	CTTAAAGGGCCAGCCAGTGGGTAG	ATTTCCCGGTCGGTCACCCATCCC	tag237196
novel_sir2795	AGAAAAATCGGACGCTGACATCAG	CCTCTTTTTAGCCTGCGACTGTAG	tag175208
novel_sir2796	CTTATTTAATGCTATGGACATCAT	TCGAATAAATTACGATACCTGTAG	tag81629
novel_sir2797	GGGTTCGTTGGACCATTGCTGTAG	CAAGCAACCTGGTAACGACATCGA	tag102550
novel_sir2798	CGCGTCAACCGGTCACATCCC	TTGCGCAGTTGGCCAGTGTAG	tag154215
novel_sir2799	CAGATTTAGGAAACAGAGGTGTAG	CTAAATCCTTTGTCTCCACATCCA	tag26188
novel_sir2800	TACTTAGACCTGTACACTGTGTAG	GAATCTGGACATGTGACACATCAT	tag211761
novel_sir2801	AGACGGAAACTAGCGACACATCAC	TCTCTGCCTTTGATCGCTGTGTAG	tag194282
novel_sir2802	AGCACGAATCTTGGAACACATCAA	CGTCGTGCTTAGAACCTTGTGTAG	tag100042
novel_sir2803	CCCTCATAATACTTTCCTCCAATTAG	GAGTATTATGAAAGGAGGTTAATCAT	tag272838
novel_sir2804	GCTTAGATAACTCGGATTAATTAG	AATCTATTGAGCCTAATTAATCCA	tag50244
novel_sir2805	ATCCAGCGTTCCTTAACTAATCAA	TATAGGTCGCAAGGAATTGATTAG	tag150325
novel_sir2806	TATCGCCGGGCTTGGACATATTAG	AGCGGCCCGAACCTGTATAATCTT	tag113715
novel_sir2807	TATCGCCGGGCTTGGACATATTAG	AGCGGCCCGAACCTGTATAATCCC	tag113715
novel_sir2808	GAGGCGTTCAGTCATAATCCG	ATCTCCGCAAGTCAGTATTAG	tag215506
novel_sir2809	GGTCGCGCGGTTGATTAAACTTAG	AGCGCGCCAACTAATTTGAATCAA	tag247522
novel_sir2810	CCGCTTCCTCCGTTGAATCCG	GCGGCGAAGGAGGCAACTTAG	tag252593
novel_sir2811	GTTGCCCGCCGCCCGACTTAG	ACGGGCGGCGGGCTGAATCCT	tag238621
novel_sir2812	GGTTGCCCGCCGCCCGACTTAG	AACGGGCGGCGGGCTGAATCCT	tag146157
novel_sir2813	TATGTGTATAATGCAGAACCTTAG	ACACATATTACGTCTTGGAATCCG	tag82871
novel_sir2814	GTTGAATGATTCGGATTCCCTTAG	ACTTACTAAGCCTAAGGGAATCTT	tag156390
novel_sir2815	ACTTACTAAGCCTAAGGGAATCCT	GTTGAATGATTCGGATTCCCTTAG	tag156390
novel_sir2816	GACTCTTCGATCGACCACTCTTAG	GAGAAGCTAGCTGGTGAGAATCTG	tag284073
novel_sir2817	CTTGCGGAGATTCAGTCTTAG	ACGCCTCTAAGTCAGAATCCA	tag210692
novel_sir2818	ACTTGCGGAGATTCAGTCTTAG	AACGCCTCTAAGTCAGAATCCA	tag149166
novel_sir2819	CAACTAGCTGCTTGTAAGAATCTT	TAGTTGATCGACGAACATTCTTAG	tag222187
novel_sir2820	TGTGGGCTGAACCTGTGTTCTTAG	ACCCGACTTGGACACAAGAATCAA	tag235249
novel_sir2821	ATTAAACTCGGCTCAATCTG	TTTAATTTGAGCCGAGTTAG	tag30875

novel_sir2822	AATTAAACTCGGCTCAATCTG	CATTAATTTGAGCCGAGTTAG	tag11142
novel_sir2823	AATTAAACTCGGCTCAATCTG	CCTTAATTTGAGCCGAGTTAG	tag98148
novel_sir2824	CAATTAAACTCGGCTCAATCTG	CCGTTAATTTGAGCCGAGTTAG	tag128191
novel_sir2825	CAATTAAACTCGGCTCAATCTG	AAGTTAATTTGAGCCGAGTTAG	tag244013
novel_sir2826	GAGGCTTGAGGCAGACACAATCGC	GTCTCCGAACTCCGTCTGTGTTAG	tag220614
novel_sir2827	TGGAGTTCAGTGGACGTGTGTTAG	CTCAAGTCACCTGCACACAATCAG	tag47277
novel_sir2828	CTTGGTGTTGGGACAGCTTGTTAG	ACCACAACCCTGTCGAACAATCAC	tag46877
novel_sir2829	CATCGCCTCGGTCTTTAAATTTAG	AGCGGAGCCAGAAATTTAAATCTG	tag204250
novel_sir2830	CGTAGGCCCTGATAACACCTTTAG	ATCCGGGACTATTGTGGAAATCGG	tag280573
novel_sir2831	ACTGCTAGCCAACAGGGAAATCCC	TTTGACGATCGGTTGTCCCTTTAG	tag82149
novel_sir2832	TTTGACGATCGGTTGTTCCTTTAG	ACTGCTAGCCAACAAGGAAATCCC	tag213318
novel_sir2833	AGTCGGCGGTGTATCTGGCTTTAG	AGCCGCCACATAGACCGAAATCGG	tag100952
novel_sir2834	CAGACGACGATTTTGGTGCTTTAG	CTGCTGCTAAAACCACGAAATCGC	tag78927
novel_sir2835	CATTGACAGGGTGTGGCAAATCGT	CAGTAACTGTCCCACACCGTTTAG	tag161395
novel_sir2836	CATAGACACGGTGTGGCAAATCGT	CAGTATCTGTGCCACACCGTTTAG	tag5903
novel_sir2837	CCGGTCCCTAAACTCGCAAATCGA	AGGGCCAGGGATTTGAGCGTTTAG	tag136138
novel_sir2838	CCTGCATAGGGTCATGATGTTTAG	ACGTATCCCAGTACTACAAATCTG	tag201393
novel_sir2839	CACTCTCACGGCCTATAAAATCCC	AAGTGAGAGTGCCGGATATTTTAG	tag242453
novel_sir2840	AATGTGGCCGTCAGCGAAAATCAG	AATTACACCGGCAGTCGCTTTTAG	tag117002
novel_sir2841	TATCGAGGCGCCTTCTTTTAG	AGCTCCGCGGAAGAAAATCTA	tag17769
novel_sir2842	GATGAAACGGTCCGCCAAAATCGG	CCCTACTTTGCCAGGCGGTTTTAG	tag287254
novel_sir2843	GACGAAACGGTCCGCCAAAATCGG	CCCTGCTTTGCCAGGCGGTTTTAG	tag27889
novel_sir2844	AGTCGGCAGTGCATCTGGTTTTAG	AGCCGTCACGTAGACCAAAATCGG	tag166152
novel_sir2845	ACTAGTGGCGCCTGCAAAAATCAC	ATTGATCACCGCGGACGTTTTTAG	tag2938
novel_sir2846	AGTCCTCCTCTTTCGTTTGCCC	GATCAGGAGGAGAAAGCAAACG	tag87389
novel_sir2847	CATCCCGAGCTCCGTTTGCAA	GTGTAGGGCTCGAGGCAAACG	tag243844
novel_sir2848	ACCTCGATGTGCGCTTTGCAA	TCTGGAGCTACACGCGAAACG	tag108340
novel_sir2849	ACTACAGCGCTAAGGCCATAAACG	ATGTCGCGATTCCGGTATTTGCAT	tag67932
novel_sir2850	AATGTCTTGGAGTCCGTGTTGCAG	TATTACAGAACCTCAGGCACAACG	tag50068
novel_sir2851	CGCATCCGCCGCTCGTTGCCG	GCGCGTAGGCGGCGAGCAACG	tag187861
novel_sir2852	CGGACACACGTATCCTATGCAACG	CTGTGTGCATAGGATACGTTGCCC	tag266269
novel_sir2853	GTTCGCAAGTATCGCTGCAACG	AGCGTTCATAGCGACGTTGCTT	tag19255
novel_sir2854	AAGAGGCCCTTTCATACAACCAGAAC	CTCCGGGAAAGTATGTTGGTCTTGCA	tag132633
novel_sir2855	CTCCGGGAAAGTATGTTGGTCTTGCA	AAGAGGCCCTTTCATACAACCAGAAC	tag132633

novel_sir2856	AATCTTTATGGGTTATAGAACG	AGAAATACCCAATATCTTGCTG	tag38164
novel_sir2857	CTTCTTTATGGGTTATAGAACG	AGAAATACCCAATATCTTGCTG	tag46215
novel_sir2858	ATTCTTTATGGGTTATAGAACG	AGAAATACCCAATATCTTGCTG	tag173342
novel_sir2859	TTTCTTTATGGGTTATAGAACG	AGAAATACCCAATATCTTGCTG	tag289564
novel_sir2860	CGGCGACGGCGGCGGCTTGCTC	CGGCCGCTGCCGCCGCCGAACG	tag40967
novel_sir2861	CGGTGCAGTCGGTTTTGGCGAACG	CACGTCAGCCAAAACCGCTTGCAA	tag49728
novel_sir2862	GGTGGCAGACGGTGCAGTCGAACG	ACCGTCTGCCACGTCAGCTTGCCG	tag81797
novel_sir2863	GGTGGCAGACGGTGCAGTCGAACG	ACCGTCTGCCACGTCAGCTTGCTG	tag81797
novel_sir2864	ATTCACCGTCTCACCGGAACG	AGTGGCAGAGTGGCCTTGCTG	tag38917
novel_sir2865	ATTCACCGTCTCACCGGAACG	AGTGGCAGAGTGGCCTTGCTT	tag38917
novel_sir2866	CATTCACCGTCTCACCGGAACG	AAGTGGCAGAGTGGCCTTGCTT	tag22355
novel_sir2867	CATTCACCGTCTCACCGGAACG	AAGTGGCAGAGTGGCCTTGCTG	tag22355
novel_sir2868	TCCTGGTCGAACGCCTGAACG	GACCAGCTTGCGGACTTGCTC	tag291401
novel_sir2869	AGGGCCAGAGCGGACTTGCTA	GGTCCCGGTCTCGCCTGAACG	tag170073
novel_sir2870	CATGGATCCATATCTTGATTGCAG	ATGTACCTAGGTATAGAACTAACG	tag227951
novel_sir2871	CATGGTGGCCCGTGCAGATTGCGC	TCGTACCACCGGGCACGTCTAACG	tag223894
novel_sir2872	TCGTACCACCGGGCGCGTCTAACG	CATGGTGGCCCGCGCAGATTGCGT	tag72442
novel_sir2873	TCGTATCACCGGGCGCGTCTAACG	CATAGTGGCCCGCGCAGATTGCGC	tag11841
novel_sir2874	TCGTACTACCGGGCGCGTCTAACG	CATGATGGCCCGCGCAGATTGCGC	tag283664
novel_sir2875	CCACCTGTCATACACACATTGCGT	AGGGTGGACAGTATGTGTGTAACG	tag202633
novel_sir2876	GTCTAACTCGGCTCAAATTAACG	GATTGAGCCGAGTTTAATTGCAC	tag41622
novel_sir2877	GTCTAACTCGGCTCAAATTAACG	GATTGAGCCGAGTTTAATTGCAA	tag41622
novel_sir2878	ATCCAATAGCCTCTTCAATTGCTT	TATAGGTTATCGGAGAAGTTAACG	tag89109
novel_sir2879	CCGCGCTTCGATGGCACACG	CGCGAAGCTACCGTGTGCCG	tag6967
novel_sir2880	CTGTACCCGACGAAGTGTGCCT	GCGACATGGGCTGCTTCACACG	tag81167
novel_sir2881	CTGCTTGCCAGTTTGTACACG	CGAACGGTCAAACATGTGCTA	tag89204
novel_sir2882	GACGAACGGTCAAACATGTGCTA	TTCTGCTTGCCAGTTTGTACACG	tag177604
novel_sir2883	AGACGAACGGTCAAACATGTGCCA	ATTCTGCTTGCCAGTTTGTACACG	tag261422
novel_sir2884	AGACGAACGGTCAAACATGTGCTA	ATTCTGCTTGCCAGTTTGTACACG	tag261422
novel_sir2885	TCGACCTAGGCCTCGACCACG	CTGGATCCGGAGCTGGTGCGC	tag290797
novel_sir2886	CCGGTAGCGCCGCGAGCCACG	CCATCGCGGCGCTCGGTGCTT	tag126458
novel_sir2887	ATGTGTTCCAAGATTCGTGCTG	ACTACACAAGGTTCTAAGCACG	tag120327
novel_sir2888	AGGTAGGTGAAAATCCAGAGCACG	CATCCACTTTTAGGTCTCGTGCGC	tag158817
novel_sir2889	AGGTAGGTGAAAATCCAGAGCACG	CATCCACTTTTAGGTCTCGTGCGT	tag158817

novel_sir2890	CTCCGGCCCGTCCGCGTGCGT	CGGAGGCCGGGCAGGCGCACG	tag200971
novel_sir2891	CTTGAGGCTTCAATTCGCACG	ACTCCGAAGTTAAGCGTGCTT	tag117252
novel_sir2892	GTGTAACGCAGAACCTCAGGCACG	CATTGCGTCTTGGAGTCCGTGCTG	tag133580
novel_sir2893	CACTGCGTCTTGGAGTCCGTGCTA	GTGTGACGCAGAACCTCAGGCACG	tag45287
novel_sir2894	GTGTAATGCAGAACCTCAGGCACG	CATTACGTCTTGGAGTCCGTGCTG	tag153735
novel_sir2895	ATGTAATGCAGAACCTCAGGCACG	CATTACGTCTTGGAGTCCGTGCTG	tag261653
novel_sir2896	ATGGGATGACTACTGGCACG	CCCTACTGATGACCGTGCCG	tag55512
novel_sir2897	CAGACTCAGCTGCACGGTTGCACG	CTGAGTCGACGTGCCAACGTGCAT	tag105611
novel_sir2898	GGGCCGGCGGACGACATCACG	CGGCCGCCTGCTGTAGTGCTG	tag284682
novel_sir2899	CCTCCCTTGCCACGGATCACG	AGGGAACGGTGCCTAGTGCGG	tag159202
novel_sir2900	AGGAAACAGTGCCTAGTGCGG	CCTCCTTTGTCACGGATCACG	tag56141
novel_sir2901	GTTTGCTGTGCCTACTCTGTCACG	AACGACACGGATGAGACAGTGCGT	tag190001
novel_sir2902	AAGACGAGTGGTCAAACAGTGCCA	TATTCTGCTCACCAGTTTGTCACG	tag231562
novel_sir2903	TATTCTGCTCACCAGTTTGTCACG	AAGACGAGTGGTCAAACAGTGCAA	tag231562
novel_sir2904	GATGTCTTGGAGTCCGTGCTGCAG	TGCTACAGAACCTCAGGCACGACG	tag48163
novel_sir2905	CGTCTTGGAGTCCGTGCTGCAG	ACGCAGAACCTCAGGCACGACG	tag235016
novel_sir2906	CTGTCCCGGTGGTGGACGACG	CAGGGCCACCACCTGCTGCTT	tag169355
novel_sir2907	CGCTATGAACGCTTGGCTGCCA	CAGCGATACTTGCGAACCGACG	tag206707
novel_sir2908	GTTGCCTCCTTCGCCGCCGACG	ACGGAGGAAGCGGCGGCTGCAC	tag125392
novel_sir2909	CTACAGATTTGTAGCCGGCTGCAA	GCGATGTCTAAACATCGGCCGACG	tag212877
novel_sir2910	CACTATCAACTTTCGGCGCTGCCT	CAGTGATAGTTGAAAGCCGCGACG	tag81686
novel_sir2911	CGCTCCGCCGGGCCGCTGCCG	CTGCGAGGCGGCCCGGCGACG	tag199151
novel_sir2912	CCGGCGAAGGCAGCGGCGACG	CCGCTTCCGTCGCCGCTGCCT	tag39905
novel_sir2913	GGGGGAAAACTGAGCGCGGCGACG	CCCTTTTGACTCGCGCCGCTGCGT	tag136510
novel_sir2914	TTGATATCTAGACATCGATCGACG	CTATAGATCTGTAGCTAGCTGCGC	tag52131
novel_sir2915	CCGATATCTAGACATCGATCGACG	CTATAGATCTGTAGCTAGCTGCGC	tag286142
novel_sir2916	CTGATGTCTAGACATCGGTCGACG	CTACAGATCTGTAGCCAGCTGCAG	tag83246
novel_sir2917	CCGATGTCTAGACATCGGTCGACG	CTACAGATCTGTAGCCAGCTGCAG	tag134731
novel_sir2918	TCGATGTCTAGACATCGGTCGACG	CTACAGATCTGTAGCCAGCTGCAG	tag145663
novel_sir2919	CAGCGGCCGCTGGTGTCGACG	CGCCGGCGACCACAGCTGCTG	tag168550
novel_sir2920	AACACTGAGAGGCACAAGCTGCAC	CCTTGTGACTCTCCGTGTTCGACG	tag291756
novel_sir2921	GGCTTGGCCGTTTCCCCCCGGACG	GAACCGGCAAAGGGGGGCCTGCGC	tag209812
novel_sir2922	GTTCGATCGTTCGCCGCGGACG	AGCTAGCAAGCGGCGCCTGCGC	tag158097
novel_sir2923	CGGTACAAGGGTGCAGCGCGGACG	CATGTTCCCACGTCGCGCCTGCTT	tag148489

novel_sir2924	CAGTACAAGGGTGCAGCGCGGACG	CATGTTCCCACGTCGCGCCTGCTT	tag167646
novel_sir2925	ACCTCCTAGCCGCGTCGGACG	GAGGATCGGCGCAGCCTGCTC	tag140203
novel_sir2926	CGGCTGCGGCGGAGCGGGACG	CGACGCCGCCTCGCCCTGCCG	tag7084
novel_sir2927	CAGAGCAAGGGGTCAGCTGACG	CTCGTTCCCCAGTCGACTGCTT	tag182904
novel_sir2928	CCTCTAGATCCGCCACTGCTT	TGGGAGATCTAGGCGGTGACG	tag128364
novel_sir2929	CACCCTCTAGATCCGCCACTGCTT	TGGTGGGAGATCTAGGCGGTGACG	tag100237
novel_sir2930	AGGGTGGACAGTATGTGTGTGACG	CCACCTGTCATACACACACTGCGT	tag86929
novel_sir2931	AGTCGGCGCCACGAACTGCGC	GATCAGCCGCGGTGCTTGACG	tag50545
novel_sir2932	ACTTTGGTCAAAGTCAAACTGCTT	ACTGAAACCAGTTTCAGTTTGACG	tag273168
novel_sir2933	ACTGAAATCAGTTTCAGTTTGACG	ACTTTAGTCAAAGTCAAACTGCTT	tag285959
novel_sir2934	AATCCGGTTCGCAGAATTATGTATAC[illegible]	AGGCCAAGCGTCTTAATACATATGCA[illegible]	tag52900
novel_sir2935	AATCCGGTTCGCAGAATTATGTATAC[illegible]	AGGCCAAGCGTCTTAATACATATGCA[illegible]	tag52900
novel_sir2936	GCTCTGGATTTTCACCTACCTACG	AGACCTAAAAGTGGATGGATGCGG	tag82207
novel_sir2937	AGACCTAAAAGTGGATGGATGCAG	GCTCTGGATTTTCACCTACCTACG	tag82207
novel_sir2938	TACTGCGGTTTCGTACAGCCTACG	GACGCCAAAGCATGTCGGATGCGA	tag253343
novel_sir2939	GCCTCAACTGCCACTCCTACG	GAGTTGACGGTGAGGATGCCC	tag188413
novel_sir2940	CGCTGCTGTCATCGTTCCGCTACG	GACGACAGTAGCAAGGCGATGCCA	tag159031
novel_sir2941	AAGGTCGCGGCGCTCATGCCG	CCTTCCAGCGCCGCGAGTACG	tag227985
novel_sir2942	CCGCCGAGCCGACACAGGTACG	CGGCTCGGCTGTGTCCATGCTT	tag18785
novel_sir2943	CCGCGAGTACAGAGGTGTACG	CGCTCATGTCTCCACATGCCG	tag28595
novel_sir2944	TTGCATTCTAAAACCATCCTTACG	CGTAAGATTTTGGTAGGAATGCAA	tag95458
novel_sir2945	CGAAGCGAAGTAGGGCCAATGCTA	ACGCTTCGCTTCATCCCGGTTACG	tag4427
novel_sir2946	CGAAGCGAAGTAGGGCCAATGCTA	CCGCTTCGCTTCATCCCGGTTACG	tag5251
novel_sir2947	CGAAGCGAAGTAGGGCCAATGCTA	TCGCTTCGCTTCATCCCGGTTACG	tag108261
novel_sir2948	ACTCTGCCACTTACAATGCCC	GGTGAGACGGTGAATGTTACG	tag113226
novel_sir2949	CACTCTGCCACTTACAATGCCC	CGGTGAGACGGTGAATGTTACG	tag77968
novel_sir2950	CCTCCGGCATACAGCTGATTTACG	AGGCCGTATGTCGACTAAATGCTG	tag287251
novel_sir2951	TTGCCTCCTAGATCTGACAAACCG	CGGAGGATCTAGACTGTTTGGCAC	tag276219
novel_sir2952	TAGTCTCCTAGATCTGACAAACCG	CAGAGGATCTAGACTGTTTGGCAC	tag175777
novel_sir2953	GAGGACGAGAAATGTTGGCAA	CTCTCCTGCTCTTTACAACCG	tag247844
novel_sir2954	TCGCAGGCGGGAGGGCAACCG	CGTCCGCCCTCCCGTTGGCTG	tag31472
novel_sir2955	ATTCTGCCTGTCAGTTTGCAACCG	AGACGGACAGTCAAACGTTGGCAC	tag138927
novel_sir2956	TCTGAAAAGCTGAATATTCAACCG	ACTTTTCGACTTATAAGTTGGCTT	tag279463
novel_sir2957	AACGTCGCTATGAACGCTTGGCTG	CGTTGCAGCGATACTTGCGAACCG	tag280572

novel_sir2958	CCGGCTGCACCCGGTGAACCG	CCGACGTGGGCCACTTGGCCA	tag241696
novel_sir2959	ACACACTTTGACACTTGGCAC	TGTGTGTGAAACTGTGAACCG	tag223038
novel_sir2960	CTTAGACGTGGCAAACTGTGGCAA	TGGAATCTGCACCGTTTGACACCG	tag72481
novel_sir2961	CTTTCCGGTATGTCGTGGTGGCAT	GAGAAAGGCCATACAGCACCACCG	tag107506
novel_sir2962	CGGGTGGGTCGGCGGTGGCGG	GTGCCCACCCAGCCGCCACCG	tag143023
novel_sir2963	GCAGGCGAGCTCCGGTGGCGG	CTCGTCCGCTCGAGGCCACCG	tag113070
novel_sir2964	TAGAGCTGCAAACCTGGTCCACCG	CTCGACGTTTGGACCAGGTGGCAC	tag172340
novel_sir2965	TAGAGCTGTCAACCTGGTCCACCG	CTCGACAGTTGGACCAGGTGGCAC	tag212401
novel_sir2966	GAGGTACCGCGTCAAGCACCG	CCATGGCGCAGTTCGTGGCGC	tag5504
novel_sir2967	ATGCTTGGCACTTTCGCACCG	CGAACCGTGAAAGCGTGGCCT	tag42450
novel_sir2968	ATGCTTGGCACTTTCGCACCG	CGAACCGTGAAAGCGTGGCCA	tag42450
novel_sir2969	CGTTCGCCAAAACCGACTGCACCG	AAGCGGTTTTGGCTGACGTGGCGC	tag79484
novel_sir2970	CTGGCTCAGAAGCAAGGTGCACCG	CCGAGTCTTCGTTCCACGTGGCGA	tag43336
novel_sir2971	CGTAACATTCACCGTCTCACCG	ATTGTAAGTGGCAGAGTGGCCT	tag97091
novel_sir2972	GACATCAAACGTCGACAGTGGCGC	CACTGTAGTTTGCAGCTGTCACCG	tag20644
novel_sir2973	GACATCAAACGTCGACAGTGGCGC	CTCTGTAGTTTGCAGCTGTCACCG	tag178946
novel_sir2974	GACATCAAACGTCGACAGTGGCGC	CGCTGTAGTTTGCAGCTGTCACCG	tag236679
novel_sir2975	GCTGCCCCGTAACATTCACCG	ACGGGGCATTGTAAGTGGCAG	tag80049
novel_sir2976	CAGCTGACCACATATTCACCG	CGACTGGTGTATAAGTGGCTT	tag228586
novel_sir2977	CTCGCGCGTGGCCAAGTGGCCC	AGGAGCGCGCACCGGTTCACCG	tag77220
novel_sir2978	ACCTATCGTTCAGGCGAGACCG	GATAGCAAGTCCGCTCTGGCCC	tag107063
novel_sir2979	AGACGACTCGGGCGCTGGCTG	CCTCTGCTGAGCCCGCGACCG	tag44996
novel_sir2980	AAGTGCCGTGGAGGAGGACCG	CACGGCACCTCCTCCTGGCGC	tag12417
novel_sir2981	TCTCAATCCGAGGGTAGCCTACCG	AGTTAGGCTCCCATCGGATGGCCT	tag143654
novel_sir2982	ATGACATGTGGGTCAGGATGGCAT	GTTACTGTACACCCAGTCCTACCG	tag148675
novel_sir2983	GGTGAAGGTCGTACCGTAAACCCG	ACTTCCAGCATGGCATTTGGGCGA	tag238350
novel_sir2984	TTCTGCCTGCCAGTTTACAACCCG	GACGGACGGTCAAATGTTGGGCAC	tag150699
novel_sir2985	TTCTGCCTGCCAGTTTGCAACCCG	GACGGACGGTCAAACGTTGGGCAC	tag265442
novel_sir2986	ATTCTGCTTGCCAGTTTCAACCCG	AGACGAACGGTCAAAGTTGGGCGC	tag206250
novel_sir2987	CATATTGGCTTCGTCTGTGGGCAC	TTGTATAACCGAAGCAGACACCCG	tag61238
novel_sir2988	GTGCCGAGGCCGTCGACACCCG	CGGCTCCGGCAGCTGTGGGCGG	tag204612
novel_sir2989	CAAGACGATCACGCGTGGGCGT	CTGTTCTGCTAGTGCGCACCCG	tag20717
novel_sir2990	ACAAGACGATCACGCGTGGGCGT	ACTGTTCTGCTAGTGCGCACCCG	tag193355
novel_sir2991	TAGCCAGTTCTGACAGTGCACCCG	CGGTCAAGACTGTCACGTGGGCGC	tag157315

novel_sir2992	CTGCCGCGCCGCGCCGACCCG	CGGCGCGGCGCGGCTGGGCGT	tag290787
novel_sir2993	CGGTCACCCGAGATCCCTGGGCGG	TGGCCAGTGGGCTCTAGGGACCCG	tag199724
novel_sir2994	CCGGCCGCCGAACCTGACCCG	CCGGCGGCTTGGACTGGGCCG	tag91394
novel_sir2995	GCTGAGACAGAGATTCACCCCG	ACTCTGTCTCTAAGTGGGGCGT	tag88370
novel_sir2996	CTGAATTTATGCGCTGCCCCG	CTTAAATACGCGACGGGGCAT	tag69869
novel_sir2997	GCTGAATTTATGCGCTGCCCCG	ACTTAAATACGCGACGGGGCAT	tag143261
novel_sir2998	TGCTGAATTTATGCGCTGCCCCG	GACTTAAATACGCGACGGGGCAT	tag131016
novel_sir2999	GGTGATCTGCTATCCCCG	ACTAGACGATAGGGGCAT	tag138037
novel_sir3000	GCTGACCGAAAAAACGTAAGCCCG	ACTGGCTTTTTTGCATTCGGGCCC	tag104913
novel_sir3001	AAGAGGAGCCTACGCGTCGGGCGT	TATTCTCCTCGGATGCGCAGCCCG	tag157709
novel_sir3002	AAGAGGAGCCTACGCGTCGGGCGA	TATTCTCCTCGGATGCGCAGCCCG	tag157709
novel_sir3003	TCCTCACCTCTGCTGCGAGCCCG	GAGTGGAGACGACGCTCGGGCTC	tag92248
novel_sir3004	AGTGGAGACGACTCGGGCGC	CCTCACCTCTGCTGAGCCCG	tag262097
novel_sir3005	ATGGGGCTACGGATCGGGCGT	CCTACCCCGATGCCTAGCCCG	tag252711
novel_sir3006	GATGGGGCTACGGATCGGGCGT	ACCTACCCCGATGCCTAGCCCG	tag74024
novel_sir3007	GAATGTTATCTCAATCGGGCCT	ACCTTACAATAGAGTTAGCCCG	tag22664
novel_sir3008	TCTCCCGCTGCAGCACGCCCG	AGGGCGACGTCGTGCGGGCGA	tag228577
novel_sir3009	AACGTGGGTCGGGGCGGGCGG	GATTGCACCCAGCCCCGCCCG	tag154763
novel_sir3010	CCTCCGGCTTCCGCCCGCCCG	AGGCCGAAGGCGGGCGGGCGC	tag174242
novel_sir3011	CCGCTCCGCCGCAGCCGCCCG	CGAGGCGGCGTCGGCGGGCGA	tag147198
novel_sir3012	AGGCCGAAGGCGAGCGGGCGC	CCTCCGGCTTCCGCTCGCCCG	tag61986
novel_sir3013	CTTTTTGACTAGAAGTACGGGCCT	TTGAAAAACTGATCTTCATGCCCG	tag13042
novel_sir3014	CTTGGCCGGTGGCACGGGCCC	CCGAACCGGCCACCGTGCCCG	tag176178
novel_sir3015	GTTTGCTGCCGGCGGTGCCCG	AACGACGGCCGCCACGGGCGG	tag53921
novel_sir3016	GCTTCGAGGGAAGGGTTGCCCG	AAGCTCCCTTCCCAACGGGCGG	tag11831
novel_sir3017	CCTCCGTTTGATCTGAAAGCCG	AGGCAAACTAGACTTTCGGCTG	tag32090
novel_sir3018	CGGGACTATTGTGGAATTCGGCCG	AGGCCCTGATAACACCTTAAGCCG	tag246978
novel_sir3019	ACCTGCCTTTGACCCTGTCGGCCT	CCTGGACGGAAACTGGGACAGCCG	tag223727
novel_sir3020	CTTGGAATATCGGTCGTCCAGCCG	ACCTTATAGCCAGCAGGTCGGCTG	tag138587
novel_sir3021	ACTCCCTGCGCGCAGCAGCCG	AGGGACGCGCGTCGTCGGCGT	tag46407
novel_sir3022	CACTCCCTGCGCGCAGCAGCCG	GAGGGACGCGCGTCGTCGGCGT	tag49792
novel_sir3023	AGCTCCTGTCGGCGTCGGCGG	GGTCGAGGACAGCCGCAGCCG	tag235417
novel_sir3024	CCTCACCATGCTAGTCGGCGC	AGGGAGTGGTACGATCAGCCG	tag244126
novel_sir3025	CTTGGGTACATTGCTCGGCTT	TTGAACCCATGTAACGAGCCG	tag288497

novel_sir3026	CGTCCCTATGACGGAGGCGAGCCG	AGGGATACTGCCTCCGCTCGGCTA	tag160148
novel_sir3027	ATCCAAACGGCAGATCGGCGT	CCTAGGTTTGCCGTCTAGCCG	tag149112
novel_sir3028	CGTGGGTCGGGGCGGGCGGCGG	TTGCACCCAGCCCCGCCCGCCG	tag170680
novel_sir3029	CCGCGTCGAGGACAGCCGCCG	CGCAGCTCCTGTCGGCGGCGG	tag197397
novel_sir3030	TCGAGGACAGCCGCAGCCGCCG	CTCCTGTCGGCGTCGGCGGCGG	tag244993
novel_sir3031	GTGCCCACCCAGCCGCCGCCG	CGGGTGGGTCGGCGGCGGCGG	tag225243
novel_sir3032	CTCGTCCGCTCGAGGCCGCCG	GCAGGCGAGCTCCGGCGGCGG	tag263693
novel_sir3033	CGCAATTTTGGCCGACGGCGGCAG	TCGCGTTAAAACCGGCTGCCGCCG	tag185747
novel_sir3034	CGCAATTTTGGCCGACGGCGGCAG	TAGCGTTAAAACCGGCTGCCGCCG	tag244714
novel_sir3035	CACTTCACAAGCCTAGCGCCG	GAAGTGTTCGGATCGCGGCGA	tag140894
novel_sir3036	GCGGCAGTTCCGGTAGCGCCG	CCGTCAAGGCCATCGCGGCGC	tag173430
novel_sir3037	CCTTAGCTGCCGCGCCGCGCCG	AATCGACGGCGCGGCGCGGCTG	tag194110
novel_sir3038	CTTACTTCTTGATTCGCGCGGCGA	TTGAATGAAGAACTAAGCGCGCCG	tag14421
novel_sir3039	CCTTGACGGCGAGCGCGGCCA	CCGGAACTGCCGCTCGCGCCG	tag226514
novel_sir3040	CGGCCGCTGGCCGGTGCGCCG	CGGCGACCGGCCACGCGGCGG	tag108949
novel_sir3041	ACAACACGATGGACGCAGCGGCGC	TGTGTTGTGCTACCTGCGTCGCCG	tag87071
novel_sir3042	TTGCCGCCGCTCCCCTTCGCCG	CGGCGGCGAGGGGAAGCGGCGG	tag250495
novel_sir3043	GCCTAAGTTGCCTCCTTCGCCG	GATTCAACGGAGGAAGCGGCGG	tag78763
novel_sir3044	CTTAGGTTCGATCGTTCGCCG	ATCCAAGCTAGCAAGCGGCGC	tag288040
novel_sir3045	TCTTAGGTTCGATCGTTCGCCG	AATCCAAGCTAGCAAGCGGCGC	tag26931
novel_sir3046	CGTTCAGTCATAATCCGGCAC	CCGCAAGTCAGTATTAGGCCG	tag26844
novel_sir3047	GGCTTGGGCAGCCGACGGCCG	GAACCCGTCGGCTGCCGGCGG	tag197100
novel_sir3048	AGCTTCGAGGCGGCCGGCCG	CCTCGAAGCTCCGCCGGCCG	tag215065
novel_sir3049	GAGCTTCGAGGCGGCCGGCCG	CCCTCGAAGCTCCGCCGGCCG	tag91048
novel_sir3050	CTCCCCCTTCGTCGCCGGCCG	CGGAGGGGGAAGCAGCGGCCG	tag182865
novel_sir3051	GCGGAGGGGGAAGCAGCGGCCG	CCTCCCCCTTCGTCGCCGGCCG	tag189048
novel_sir3052	GCCACCTCTCGTCGCCGGCCG	GGCGGTGGAGAGCAGCGGCCG	tag77045
novel_sir3053	CTCCGCCCCACTCGCCGGCCG	CAGAGGCGGGGTGAGCGGCCG	tag101156
novel_sir3054	CCGGGTCAGGTTCGGCGGCCG	CCCAGTCCAAGCCGCCGGCCT	tag100779
novel_sir3055	CCGCAGATGCTGAAGTGGCGGCCG	CGTCTACGACTTCACCGCCGGCCT	tag81717
novel_sir3056	GACTAAAGATGGCTCAGCCGGCCA	CCCTGATTTCTACCGAGTCGGCCG	tag140161
novel_sir3057	GATAGTCTACATTCGCCCGGGCCG	ATCAGATGTAAGCGGGCCCGGCCT	tag19316
novel_sir3058	TTGGACATCGTCCGTGCCGGGCCG	CCTGTAGCAGGCACGGCCCGGCAC	tag165560
novel_sir3059	CTGCTGAATCACTAGTGGCCG	CGACTTAGTGATCACCGGCAC	tag288951

novel_sir3060	GGTTCCCGGGCACGGTGGCCG	AAGGGCCCGTGCCACCGGCCA	tag266805
novel_sir3061	GGGTTCCCGGGCACGGTGGCCG	CAAGGGCCCGTGCCACCGGCCA	tag50323
novel_sir3062	GGCTGCCGCCGATCGATGCCG	GACGGCGGCTAGCTACGGCGG	tag136456
novel_sir3063	CGGCTGCCGCCGATCGATGCCG	CGACGGCGGCTAGCTACGGCGG	tag204973
novel_sir3064	TCGCCTGATCGCCCGGATGCCG	CGGACTAGCGGGCCTACGGCTC	tag191695
novel_sir3065	CGCGGCGCTACCGGAACTGCCG	GCCGCGATGGCCTTGACGGCGA	tag5572
novel_sir3066	CTTGGACCTCGCCGACGGCGG	CCGAACCTGGAGCGGCTGCCG	tag290108
novel_sir3067	CCGCTCACCCCGCCTCTGCCG	CGAGTGGGGCGGAGACGGCAG	tag17522
novel_sir3068	CAGTAGTTGCCGTCGGTGCCG	CATCAACGGCAGCCACGGCAT	tag183316
novel_sir3069	CATGTGAGAAAAAGTCAACGGCGT	TTGTACACTCTTTTTCAGTTGCCG	tag235405
novel_sir3070	TCTCAACACCTCGACCCAAATCCG	AGTTGTGGAGCTGGGTTTAGGCAG	tag152204
novel_sir3071	TGTAGCCAGCGGGCTGCGCATCCG	ATCGGTCGCCCGACGCGTAGGCTC	tag17864
novel_sir3072	GGTAGCCAGCGGGCTGCGCATCCG	ATCGGTCGCCCGACGCGTAGGCTC	tag106376
novel_sir3073	TAGCCAGTTCTGACAGTGCATCCG	CGGTCAAGACTGTCACGTAGGCGC	tag61006
novel_sir3074	CGGTCAAGACCGCCACGTAGGCGC	GGGCCAGTTCTGGCGGTGCATCCG	tag156519
novel_sir3075	ATTGGTGACTCAATTCATCCG	ACCACTGAGTTAAGTAGGCAC	tag211223
novel_sir3076	ACCACTGAGTTAAGTAGGCAA	ATTGGTGACTCAATTCATCCG	tag211223
novel_sir3077	AACCACTGAGTTAAGTAGGCAA	GATTGGTGACTCAATTCATCCG	tag66746
novel_sir3078	CTCCGCTGGATGGTGAGGCAG	GGGAGGCGACCTACCACTCCG	tag192172
novel_sir3079	CGGCCGCTGCTTCCCCCTCCG	CGGCGACGAAGGGGGAGGCGC	tag272475
novel_sir3080	CCGGCCGCTGCTTCCCCCTCCG	CCGGCGACGAAGGGGGAGGCGC	tag140608
novel_sir3081	CGACTACAACAACGGAGGCAA	TTGCTGATGTTGTTGCCTCCG	tag220920
novel_sir3082	ACGACTACAACAACGGAGGCAA	CTTGCTGATGTTGTTGCCTCCG	tag99199
novel_sir3083	ACCTCGCCATCTACCGCTCCG	GAGCGGTAGATGGCGAGGCCG	tag285079
novel_sir3084	GACGGAGCGGCGACGAGGCGC	CTCTGCCTCGCCGCTGCTCCG	tag58935
novel_sir3085	CGGGGGTATCGTGACTAGAGGCTT	CGGCCCCCATAGCACTGATCTCCG	tag97215
novel_sir3086	GTTTCCTAAATCTGTCCAAGTCCG	AAGGATTTAGACAGGTTCAGGCCC	tag149233
novel_sir3087	CGTGTGGGTACGACGAGTCCG	ACACCCATGCTGCTCAGGCCC	tag21223
novel_sir3088	GTGGTGGCTGAGGCTTGTAGTCCG	CCACCGACTCCGAACATCAGGCCA	tag201528
novel_sir3089	CGGCCGAACCTGTAGCAGGCAC	GTGCCGGCTTGGACATCGTCCG	tag54415
novel_sir3090	CTGCTGCCTCGTGGTCGTCCG	CGACGGAGCACCAGCAGGCTG	tag226705
novel_sir3091	CACTAAGTGAAATACAGTTGTCCG	GATTCACTTTATGTCAACAGGCAG	tag66585
novel_sir3092	GGTGGTGCCGCAATTCAAATTCCG	ACCACGGCGTTAAGTTTAAGGCGT	tag9939
novel_sir3093	ACCGGCGCGAGCGGCAGTTCCG	GCCGCGCTCGCCGTCAAGGCCA	tag117194

novel_sir3094	AGTGGATCGTGGCAGCAAGGCCA	AGTCACCTAGCACCGTCGTTCCG	tag203248
novel_sir3095	CGATCCAACAATTCCAAGGCTT	ACGCTAGGTTGTTAAGGTTCCG	tag3171
novel_sir3096	AGGCTAGAGAAGTTAGGTTCCG	CGATCTCTTCAATCCAAGGCTA	tag133071
novel_sir3097	CCGCCCGCGCCTCTGGTTCCG	CGGGCGCGGAGACCAAGGCCG	tag91763
novel_sir3098	CAGCTAGGCTAGTATCAAAGGCAT	TGGTCGATCCGATCATAGTTTCCG	tag104825
novel_sir3099	CAATTAAGGACCGGTTCGCGA	CCGTTAATTCCTGGCCAAGCG	tag91640
novel_sir3100	ACCGTTAATTCCTGGCCAAGCG	GCAATTAAGGACCGGTTCGCGA	tag241686
novel_sir3101	CGGCACACGGTAGCTTCGCGC	GGGCCGTGTGCCATCGAAGCG	tag78285
novel_sir3102	CGGCACACGGTAGCTTCGCGC	AGGCCGTGTGCCATCGAAGCG	tag234622
novel_sir3103	CCGGCACACGGTAGCTTCGCGC	TAGGCCGTGTGCCATCGAAGCG	tag53728
novel_sir3104	TACGGCTCTCTGTACACCTAAGCG	GCCGAGAGACATGTGGATTCGCCG	tag134617
novel_sir3105	GCTGAGTCGATGCTGAGCACAGCG	ACTCAGCTACGACTCGTGTCGCCC	tag33730
novel_sir3106	CGGCGGCTACCGACGACAGCG	CGCCGATGGCTGCTGTCGCGC	tag89260
novel_sir3107	ATCCGTCGTCGTTCGTCGCGT	AGTAGGCAGCAGCAAGCAGCG	tag109056
novel_sir3108	CGCGCCTCCCCCTTCGTCGCCG	CGGCGCGGAGGGGGAAGCAGCG	tag124426
novel_sir3109	CGCCGCCACCTCTCGTCGCCG	TGGCGGCGGTGGAGAGCAGCG	tag155221
novel_sir3110	CCGCCGCCACCTCTCGTCGCCG	GTGGCGGCGGTGGAGAGCAGCG	tag19061
novel_sir3111	CTCTAGGTCGGGGCGCAGCG	GATCCAGCCCCGCGTCGCAC	tag204152
novel_sir3112	ACTCTAGGTCGGGGCGCAGCG	AGATCCAGCCCCGCGTCGCAC	tag17493
novel_sir3113	GACTCTAGGTCGGGGCGCAGCG	GAGATCCAGCCCCGCGTCGCAC	tag146284
novel_sir3114	GTGACTCTAGGTCGGGGCGCAGCG	CTGAGATCCAGCCCCGCGTCGCAC	tag22129
novel_sir3115	GCGGCAGCTAAGGCGGCAGCG	CCGTCGATTCCGCCGTCGCCG	tag120130
novel_sir3116	GCTCGTTCGGCGGCGGCAGCG	AGCAAGCCGCCGCCGTCGCCG	tag19751
novel_sir3117	ACTTACAATGCCCCGTCGCGT	GGTGAATGTTACGGGGCAGCG	tag251344
novel_sir3118	CTTGTCCTGATTCCCGAGTCGCTT	AGGAACAGGACTAAGGGCTCAGCG	tag288792
novel_sir3119	ATGTAGATGCACGTTTCAGCG	CATCTACGTGCAAAGTCGCAT	tag243248
novel_sir3120	CTAAGACACCGCACAAAGTCGCTT	GAGATTCTGTGGCGTGTTTCAGCG	tag58181
novel_sir3121	AATCCGAATTTTCTAAGCAGAGCG	AGGCTTAAAAGATTCGTCTCGCAA	tag274725
novel_sir3122	TACTTCAGTGGTATCTGCAGAGCG	GAAGTCACCATAGACGTCTCGCTG	tag108849
novel_sir3123	GAAGTCACCATAAACGTCTCGCTT	TACTTCAGTGGTATTTGCAGAGCG	tag200830
novel_sir3124	ACATGGATATAGTAAGTCTCGCTT	AATGTACCTATATCATTCAGAGCG	tag138511
novel_sir3125	CATCCTAGTACTACTCTCGCCC	GGGTAGGATCATGATGAGAGCG	tag122884
novel_sir3126	CGCCGCCGCTCGATCTCGCCG	GCGCGGCGGCGAGCTAGAGCG	tag78808
novel_sir3127	CCATCGCTCACGTGCTCGCTG	GCGGTAGCGAGTGCACGAGCG	tag228490

novel_sir3128	GCCTGGGTGGCGGCGCGAGCG	GACCCACCGCCGCGCTCGCCT	tag288092
novel_sir3129	CCAGCCCTCGCCCGCTCGCCG	GAGGTCGGGAGCGGGCGAGCG	tag29490
novel_sir3130	CCATTGAACTACGCTCGCTA	ACGGTAACTTGATGCGAGCG	tag290757
novel_sir3131	CCATTGAACTACGCTCGCTA	ATGGTAACTTGATGCGAGCG	tag290856
novel_sir3132	AGCTCGTGGCTCGTTAGCTCGCTT	CTTCGAGCACCGAGCAATCGAGCG	tag20605
novel_sir3133	AGCTCGTGGCTCGTTAGCTCGCTT	TTTCGAGCACCGAGCAATCGAGCG	tag53199
novel_sir3134	CGGCGGCGGCGGCCTCGAGCG	CGCCGCCGCCGGAGCTCGCCT	tag144397
novel_sir3135	CAACTCTCTGCCTCCTCGCGC	GGGTTGAGAGACGGAGGAGCG	tag166097
novel_sir3136	ACGGCAGAGGCGGGGTGAGCG	CCGTCTCCGCCCCACTCGCCG	tag125597
novel_sir3137	GCCTCTAGCATGTTATCGCAT	TACGGAGATCGTACAATAGCG	tag68185
novel_sir3138	ATGCTTAGGCCCTGATTTCTAGCG	CGAATCCGGGACTAAAGATCGCTA	tag256316
novel_sir3139	CTTTTTCCCTCTCCGTGCGCGC	CGGAAAAAGGGAGAGGCACGCG	tag17297
novel_sir3140	GCGGCGGCTGTCCTCGACGCG	CCGCCGACAGGAGCTGCGCCG	tag89428
novel_sir3141	AGGGTGCACCGTAACTACACCGCG	CCACGTGGCATTGATGTGGCGCTT	tag126980
novel_sir3142	CCACATGGCATTGACGTGGCGCTT	AGGGTGTACCGTAACTGCACCGCG	tag119027
novel_sir3143	GAGCCAGAAGTGTACTGCACCGCG	CGGTCTTCACATGACGTGGCGCTT	tag278265
novel_sir3144	TCTGCTTACCAGTTTCAACCCGCG	ACGAATGGTCAAAGTTGGGCGCGG	tag193667
novel_sir3145	TCTGCTTACCAGTTTCAACCCGCG	ACGAATGGTCAAAGTTGGGCGCTG	tag193667
novel_sir3146	ACGAACGGTCAAAGTTGGGCGCGG	TTTGCTTGCCAGTTTCAACCCGCG	tag287930
novel_sir3147	ACGGACGATCAAAGTTGGGCGCGG	TCTGCCTGCTAGTTTCAACCCGCG	tag288479
novel_sir3148	TATCACTAGTAACGGCCAGCCGCG	AGTGATCATTGCCGGTCGGCGCCC	tag172904
novel_sir3149	ACGAGCCGCACCGCCGCCGCG	CTCGGCGTGGCGGCGGCGCTA	tag217031
novel_sir3150	TCGAGGACAGCCGCCGCCGCG	CTCCTGTCGGCGGCGGCGCAC	tag110797
novel_sir3151	AGGTTCGATCGTTCGCCGCG	CAAGCTAGCAAGCGGCGCCT	tag280170
novel_sir3152	TAGGTTCGATCGTTCGCCGCG	CCAAGCTAGCAAGCGGCGCCT	tag236655
novel_sir3153	CACATCGCCGCGCCGGCGCCC	GGGTGTAGCGGCGCGGCCGCG	tag58949
novel_sir3154	GCTGCCGCCTTAGCTGCCGCG	ACGGCGGAATCGACGGCGCGG	tag162788
novel_sir3155	TAGGCCACTAGGCGATTATCCGCG	CCGGTGATCCGCTAATAGGCGCGT	tag80821
novel_sir3156	CGGAGAGGGAAAAAGGCGCCG	GTGCCTCTCCCTTTTTCCGCG	tag10997
novel_sir3157	GCACACGGTAGCTTCGCGCCA	GCCGTGTGCCATCGAAGCGCG	tag187572
novel_sir3158	AACTCTCTGCCTCCTCGCGCGT	GGTTGAGAGACGGAGGAGCGCG	tag218828
novel_sir3159	ACGGTCTAGCCGACACGCGCGCTG	CCTGCCAGATCGGCTGTGCGCGCG	tag125328
novel_sir3160	GGGCATTAAATCACCGGCGCG	CGTAATTTAGTGGCCGCGCCG	tag150361
novel_sir3161	CGTTGATATAAGTAGGCCGGCGCG	AACTATATTCATCCGGCCGCGCAC	tag257496

novel_sir3162	CGGAGGGGGTGTAGCGGCGCG	CTCCCCCACATCGCCGCGCCG	tag258052
novel_sir3163	CGGCGAGCGTCCGGCGGCGCG	CGCTCGCAGGCCGCCGCGCCA	tag160950
novel_sir3164	AATATAATGATGCTAGCCGCGCAA	TTTTATATTACTACGATCGGCGCG	tag108853
novel_sir3165	TTTTATATTACTACGATCGGCGCG	AATATAATGATGCTAGCCGCGCAG	tag108853
novel_sir3166	GTTCGGCCGGGCCGGCTGCGCG	AGCCGGCCCGGCCGACGCGCCG	tag4888
novel_sir3167	CTGTTCCCATTGTTAGCGCTA	AGGACAAGGGTAACAATCGCG	tag7778
novel_sir3168	GAGGACAAGGGTAACAATCGCG	CCTGTTCCCATTGTTAGCGCTA	tag273681
novel_sir3169	CTTCTACTGCTGTTAGCGCTA	AGGAAGATGACGACAATCGCG	tag37998
novel_sir3170	TGTCCTTGTTCTTGTATCGCG	AGGAACAAGAACATAGCGCAC	tag141484
novel_sir3171	TCTGCTCACCAATTTCAACTCGCG	ACGAGTGGTTAAAGTTGAGCGCGG	tag104834
novel_sir3172	ACGGACGATTAAAGTTGAGCGCGG	TTTGCCTGCTAATTTCAACTCGCG	tag63624
novel_sir3173	AGTCGAGCAAGCCAGAGCGCCT	CGTCAGCTCGTTCGGTCTCGCG	tag231444
novel_sir3174	CGAACTGAACTAAGAGCGCTT	GGGCTTGACTTGATTCTCGCG	tag89077
novel_sir3175	CGAACTGAACTAAGAGCGCTT	AGGCTTGACTTGATTCTCGCG	tag106973
novel_sir3176	CGAACTGAACTAAGAGCGCTT	CGGCTTGACTTGATTCTCGCG	tag207010
novel_sir3177	TCTGCCTGCTAGTTTCAATTCGCG	ACGGACGATCAAAGTTAAGCGCGG	tag262749
novel_sir3178	GGTAGAGGCAGGATTTTATTCGCG	ATCTCCGTCCTAAAATAAGCGCAG	tag292691
novel_sir3179	GATCTGCTAAACCCAAGCTTCGCG	AGACGATTTGGGTTCGAAGCGCCA	tag100047
novel_sir3180	CTCTAATACTGTTGGCAAGCGCAC	TTGAGATTATGACAACCGTTCGCG	tag46909
novel_sir3181	CGTGACAAACGTACGGCCAAGGCG	ACTGTTTGCATGCCGGTTCCGCAC	tag110683
novel_sir3182	CGTGACAACTACCCGGCCAAGGCG	ACTGTTGATGGGCCGGTTCCGCAC	tag141996
novel_sir3183	CGCCGCGCCGTCGATTCCGCCG	GCGCGGCGCGGCAGCTAAGGCG	tag108613
novel_sir3184	CGGGTTAAATCTCGCTGTCCGCCT	TGGCCCAATTTAGAGCGACAGGCG	tag226857
novel_sir3185	CACGCTCGTGTCAATCGTCCGCCG	TGGTGCGAGCACAGTTAGCAGGCG	tag181617
novel_sir3186	CCTCGGCATCCGGGCGATCAGGCG	AGCCGTAGGCCCGCTAGTCCGCTA	tag157592
novel_sir3187	CCCGAGAATGGCCCTCCGCTG	TGGGGCTCTTACCGGGAGGCG	tag145802
novel_sir3188	TGGGATGACTACTGGCACGGCG	CCTACTGATGACCGTGCCGCGA	tag186747
novel_sir3189	TGCTACATATGTCCAAGCCCGGCG	GATGTATACAGGTTCGGGCCGCTG	tag249303
novel_sir3190	CGCTACATATGTCCAAGCCCGGCG	GATGTATACAGGTTCGGGCCGCTG	tag287196
novel_sir3191	CGCTACATCTGTCCAAGCCCGGCG	GATGTAGACAGGTTCGGGCCGCTG	tag152653
novel_sir3192	CTTCGAGGCGGCCGGCCGCGG	TCGAAGCTCCGCCGGCCGGCG	tag102586
novel_sir3193	AGGTGAGGAACCCCGGCCGCGC	CCTCCACTCCTTGGGGCCGGCG	tag219087
novel_sir3194	GCCTCCCCCACATCGCCGCGC	CGCGGAGGGGGTGTAGCGGCG	tag250557
novel_sir3195	GCGGCTGTCCTCGACGCGGCG	CCGACAGGAGCTGCGCCGCCG	tag24017

novel_sir3196	GGCGGCTGTCCTCGACGCGGCG	GCCGACAGGAGCTGCGCCGCCG	tag193941
novel_sir3197	GATTCCGCCGTCGCCGCCGCTT	AGCTAAGGCGGCAGCGGCGGCG	tag173669
novel_sir3198	GGCTGTCCTCGACGCGGCGGCG	GACAGGAGCTGCGCCGCCGCCG	tag177079
novel_sir3199	CGGTGCGGCTCGTTCGGCGGCG	CACGCCGAGCAAGCCGCCGCCG	tag206450
novel_sir3200	AAAGGATTCAGCCCGCCGCCC	CGTTTCCTAAGTCGGGCGGCG	tag190232
novel_sir3201	AGGTCCAAGCCACCGCCGCCA	GCTCCAGGTTCGGTGGCGGCG	tag70906
novel_sir3202	AAAATATAATGATGCTAGCCGCGT	TCTTTTATATTACTACGATCGGCG	tag243508
novel_sir3203	TCTTTTATATTACTACGATCGGCG	AAAATATAATGATGCTAGCCGCGC	tag243508
novel_sir3204	ATGGACACAGCCGAGCCGCCC	CGTACCTGTGTCGGCTCGGCG	tag201662
novel_sir3205	CATGGACACAGCCGAGCCGCCC	TCGTACCTGTGTCGGCTCGGCG	tag46728
novel_sir3206	CGGCGGTGCGGCTCGTTCGGCG	CGCCACGCCGAGCAAGCCGCCG	tag44978
novel_sir3207	GATAAGCTTATCGGCACGGGCG	ATTCGAATAGCCGTGCCCGCGG	tag41690
novel_sir3208	ATTAACTAGCATGGTGGCCCGCGC	GATAATTGATCGTACCACCGGGCG	tag120583
novel_sir3209	GATAGTACATGTGCATTCCGGGCG	ATCATGTACACGTAAGGCCCGCTG	tag265463
novel_sir3210	GCGGACGAAGCGCGGCGGGCG	CCTGCTTCGCGCCGCCCGCCT	tag118531
novel_sir3211	ACTGGGAACCTCGGTTTACTGGCG	ACCCTTGGAGCCAAATGACCGCCT	tag273504
novel_sir3212	CGTCTAAAATCTGGCGGACCGCGT	TAGCAGATTTTAGACCGCCTGGCG	tag149368
novel_sir3213	GCCCCATCAGCGCCACCGCCG	GGCGGGGTAGTCGCGGTGGCG	tag233173
novel_sir3214	ATAGGTATCCCTGTCGAACCGCGG	GCTATCCATAGGGACAGCTTGGCG	tag292150
novel_sir3215	GAGAATTGGTGAGTGACGCAA	GTCTCTTAACCACTCACTGCG	tag171735
novel_sir3216	AGAGAATTGGTGAGTGACGCAA	GGTCTCTTAACCACTCACTGCG	tag49345
novel_sir3217	TCTGCTTACCAGTTTCAACCTGCG	ACGAATGGTCAAAGTTGGACGCGG	tag10209
novel_sir3218	TCTGCTTACTAGTTTCAACCTGCG	ACGAATGATCAAAGTTGGACGCGG	tag41804
novel_sir3219	TCTGTCTGCTAGTTTCAACCTGCG	ACAGACGATCAAAGTTGGACGCGG	tag97722
novel_sir3220	ACGAACGATCAAAGTTGGACGCGG	TCTGCTTGCTAGTTTCAACCTGCG	tag126810
novel_sir3221	TCTGTCTGTTATTTTCAACCTGCG	ACAGACAATAAAAGTTGGACGCGG	tag24355
novel_sir3222	AGACGAAGCAGGTGGACGCCG	CGTCTGCTTCGTCCACCTGCG	tag22634
novel_sir3223	ATTAGACAATGGACGCTT	CGTAATCTGTTACCTGCG	tag293522
novel_sir3224	AGTAATCTGTTACCTGCG	ATTAGACAATGGACGCTA	tag41943
novel_sir3225	TGTAATCTGTTACCTGCG	ATTAGACAATGGACGCTA	tag110449
novel_sir3226	GGTAATCTGTTACCTGCG	ATTAGACAATGGACGCTA	tag186886
novel_sir3227	CGTAATCTGTTACCTGCG	ATTAGACAATGGACGCTA	tag293522
novel_sir3228	AGGATAGGTAATCTGTTACCTGCG	CTATCCATTAGACAATGGACGCTT	tag251548
novel_sir3229	ACTGAAGATCAGACACTGCCTGCG	ACTTCTAGTCTGTGACGGACGCTG	tag74606

novel_sir3230	ACTGAAGATCGGACACTGCCTGCG	ACTTCTAGCCTGTGACGGACGCCA	tag164271
novel_sir3231	ACTGAAGATCGGACACTGCCTGCG	ACTTCTAGCCTGTGACGGACGCTG	tag164271
novel_sir3232	ACTGAAGATCGGACACTGCCTGCG	ACTTCTAGCCTGTGACGGACGCCG	tag164271
novel_sir3233	ATCTGTGGCTGCGGCTCCTGCG	GACACCGACGCCGAGGACGCGC	tag16680
novel_sir3234	CAGATCCGGCTCGCCGCTGCG	CTAGGCCGAGCGGCGACGCGG	tag164379
novel_sir3235	CGGGGCCGGCCAGCGACGCAA	CCGCCCCGGCCGGTCGCTGCG	tag147571
novel_sir3236	CGGTTCGGCCGGGCCGGCTGCG	CAAGCCGGCCCGGCCGACGCGC	tag41006
novel_sir3237	AGCACGGACTCCAAGACGCAG	CATCGTGCCTGAGGTTCTGCG	tag66051
novel_sir3238	TTCTAATTTTAAAAACACCGTGCG	GATTAAAATTTTTGTGGCACGCTT	tag178310
novel_sir3239	GTTCCGAGCGAACCACCGTGCG	AGGCTCGCTTGGTGGCACGCGG	tag179104
novel_sir3240	GATTAAGATTTTCGTGGCACGCTT	TTCTAATTCTAAAAGCACCGTGCG	tag32349
novel_sir3241	TTCTAATTTTAAAAGCACCGTGCG	GATTAAAATTTTCGTGGCACGCTT	tag165890
novel_sir3242	GTGGCCTCGAGCGGTCGTGCG	CCGGAGCTCGCCAGCACGCGG	tag133805
novel_sir3243	CTACTCTCGCCCAAGCACGCTT	ATGATGAGAGCGGGTTCGTGCG	tag252897
novel_sir3244	AACTAGGACTCGACGGTAGGTGCG	GATCCTGAGCTGCCATCCACGCAC	tag176955
novel_sir3245	GACTAGGACTCGACGGTAGGTGCG	GATCCTGAGCTGCCATCCACGCAC	tag205268
novel_sir3246	GATCCTGAGCTGCCATCCACGCAA	GACTAGGACTCGACGGTAGGTGCG	tag205268
novel_sir3247	ACTTTGCCACAGTTTGCCACGCCT	TGTGAAACGGTGTCAAACGGTGCG	tag225573
novel_sir3248	CAGGCATATAGGCTACACTGTGCG	CCGTATATCCGATGTGACACGCCA	tag68661
novel_sir3249	TCTGCCTGTCAGATCGGCTGTGCG	ACGGACAGTCTAGCCGACACGCGC	tag60565
novel_sir3250	CGGCCTAATACTGACTTGCG	CGGATTATGACTGAACGCCT	tag171047
novel_sir3251	ACGGCCTAATACTGACTTGCG	CCGGATTATGACTGAACGCCT	tag14170
novel_sir3252	GCAACGTCGCTATGAACGCTT	TTCGTTGCAGCGATACTTGCG	tag258466
novel_sir3253	AGCAACGTCGCTATGAACGCTT	TTTCGTTGCAGCGATACTTGCG	tag55361
novel_sir3254	ACGGATGATCAAAGTTAAACGCGG	TCTGCCTACTAGTTTCAATTTGCG	tag157194
novel_sir3255	TTTACGGCGTGGACGCAAAAATCG	ATGCCGCACCTGCGTTTTTAGCCT	tag193728
novel_sir3256	ATTGTCATGTAGGACTTTTAGCTT	TGTAACAGTACATCCTGAAAATCG	tag220360
novel_sir3257	CATAGTGTTTTGATGTCTAAATCG	ATCACAAAACTACAGATTTAGCGT	tag160669
novel_sir3258	CTCTCCAATTGACTCATTTAGCCA	CGGAGAGGTTAACTGAGTAAATCG	tag277377
novel_sir3259	ACCCATCCGCATCTAAGTTAGCTT	ACTGGGTAGGCGTAGATTCAATCG	tag77969
novel_sir3260	CAAACCACACACTTGTCTTAGCCG	TTGTTTGGTGTGTGAACAGAATCG	tag76609
novel_sir3261	CAGGACCTTGTGTTGGAATCG	CCTGGAACACAACCTTAGCCG	tag195619
novel_sir3262	GCCTTTTGCCTCACCAGATAATCG	GAAAACGGAGTGGTCTATTAGCGT	tag116527
novel_sir3263	GCCTTTTGACTCGCCAGGTAATCG	GAAAACTGAGCGGTCCATTAGCGC	tag255308

novel_sir3264	GCCTTTTGCCTCGCCAGGTAATCG	GAAAACGGAGCGGTCCATTAGCGT	tag33465
novel_sir3265	GAAAACGGAGCGGTCCATTAGCGA	GCCTTTTGCCTCGCCAGGTAATCG	tag33465
novel_sir3266	ATGAAGACCGAGTCAAATTAGCCA	TCTACTTCTGGCTCAGTTTAATCG	tag222383
novel_sir3267	CGTCGACCGAATTTTTAGACATCG	AGCTGGCTTAAAAATCTGTAGCCC	tag1181
novel_sir3268	GACTTTAGTCCCGGTTGGTAGCAC	CCCTGAAATCAGGGCCAACCATCG	tag229192
novel_sir3269	ATAATCCGGCACACGGTAGCTT	AGTATTAGGCCGTGTGCCATCG	tag179873
novel_sir3270	GCCTAGTCTGCGGGCTAGGCATCG	GATCAGACGCCCGATCCGTAGCCC	tag284295
novel_sir3271	CGTACTTGGCTGACTGCATCG	ATGAACCGACTGACGTAGCAA	tag94311
novel_sir3272	TTGTTACCCTTGTCCTCATCG	CAATGGGAACAGGAGTAGCGC	tag108154
novel_sir3273	GACATCATGTGAGATAAGTAGCAT	TTCTGTAGTACACTCTATTCATCG	tag255003
novel_sir3274	CAATGTAAGTCATTCTAGCAT	GTGTTACATTCAGTAAGATCG	tag187662
novel_sir3275	GCCGGCCGACCTCTCTAGCTC	TCCGGCCGGCTGGAGAGATCG	tag76880
novel_sir3276	ACCTGGCAAACCGTGTTGAGATCG	GACCGTTTGGCACAACTCTAGCTC	tag146591
novel_sir3277	CAGGCGCCGCTTGCTAGCTA	GCGTCCGCGGCGAACGATCG	tag79624
novel_sir3278	GCGCAGGGAGTGGCACGATCG	CGTCCCTCACCGTGCTAGCCG	tag47982
novel_sir3279	TGGAGCGGCTGCCGCCGATCG	CTCGCCGACGGCGGCTAGCTA	tag262777
novel_sir3280	GAGATTCAGTCTTAGGTTCGATCG	CTAAGTCAGAATCCAAGCTAGCAA	tag265141
novel_sir3281	CGGAAAGGGTGGGGTTTTCGATCG	CTTTCCCACCCCAAAAGCTAGCCA	tag280640
novel_sir3282	CCGTTCCGTCGTGGCGCCTAGCGG	CAGGCAAGGCAGCACCGCGGATCG	tag66547
novel_sir3283	CCGTTCCGTCGTGGCGCCTAGCGG	CGGGCAAGGCAGCACCGCGGATCG	tag160236
novel_sir3284	ATATTTATAGCTGGCTTATAGCCC	TATATAAATATCGACCGAATATCG	tag29290
novel_sir3285	TGCTGAAAGCCATCTTAGATATCG	GACTTTCGGTAGAATCTATAGCAA	tag238777
novel_sir3286	TGCTGAAAGCCATTTTAGATATCG	GACTTTCGGTAAAATCTATAGCAA	tag5724
novel_sir3287	GACTTTCGGCAAAATCTATAGCAA	TGCTGAAAGCCGTTTTAGATATCG	tag226375
novel_sir3288	CGTAGAACCTCAGGCACGATATCG	ATCTTGGAGTCCGTGCTATAGCTA	tag221704
novel_sir3289	CGTAGAACCTCAGGCACGATATCG	ATCTTGGAGTCCGTGCTATAGCTG	tag221704
novel_sir3290	CTCTACCGACTCACCAACTATCG	GATGGCTGAGTGGTTGATAGCTC	tag173950
novel_sir3291	CTTGTCCTTGTTCTTGTATCG	ACAGGAACAAGAACATAGCGC	tag187367
novel_sir3292	CTCGTGCAAAAAACCGACTTATCG	GCACGTTTTTTGGCTGAATAGCTA	tag199632
novel_sir3293	TGCGCCACAGGTGACCGTTTATCG	GCGGTGTCCACTGGCAAATAGCCA	tag37695
novel_sir3294	CTTCTATGCGTTTTGCTCCACTCG	AGATACGCAAAACGAGGTGAGCCA	tag50378
novel_sir3295	TCTGACTACCAGTTTTGCGACTCG	ACTGATGGTCAAAACGCTGAGCAC	tag125070
novel_sir3296	GAGGCCGACAAGTTGGAGCAT	TTCTCCGGCTGTTCAACCTCG	tag180466
novel_sir3297	GTTTTACTTGATCTCAACACCTCG	AAATGAACTAGAGTTGTGGAGCTG	tag273343

novel_sir3298	CTGTCCTCGACCTAGGCCTCG	CAGGAGCTGGATCCGGAGCTG	tag110615
novel_sir3299	CCTAGTGCGGCGCCGGAGCTC	ACGGATCACGCCGCGGCCTCG	tag260589
novel_sir3300	ACGGATCACGCCGTGGCCTCG	CCTAGTGCGGCACCGGAGCTC	tag177819
novel_sir3301	GACAACAAATTTTGGACGGAGCCG	TGCTGTTGTTTAAAACCTGCCTCG	tag136321
novel_sir3302	ACGGCGGAGGACACGGAGCGG	CCTGCCGCCTCCTGTGCCTCG	tag27748
novel_sir3303	GTTGATTAAACGTTTGCCTCG	ACTAATTTGCAAACGGAGCTC	tag83601
novel_sir3304	CGGCGGCTGCGGCTGTCCTCG	CGCCGACGCCGACAGGAGCTG	tag45090
novel_sir3305	CGCCGACGCCGACAGGAGCGG	CGGCGGCTGCGGCTGTCCTCG	tag45090
novel_sir3306	GGTCCCTATGGGAGGTAACGCTCG	AGGGATACCCTCCATTGCGAGCTG	tag205302
novel_sir3307	TGGAGGAAGGCGCACGACCGCTCG	CTCCTTCCGCGTGCTGGCGAGCTC	tag219152
novel_sir3308	CGATGGCCTTGACGGCGAGCGC	GCGCTACCGGAACTGCCGCTCG	tag232421
novel_sir3309	CATCTCCTACTGCAGCGCTCG	AGAGGATGACGTCGCGAGCTC	tag276168
novel_sir3310	AATACCTCCTCTCTACCGGCTCG	ATGGAGGAGAGATGGCCGAGCGG	tag207995
novel_sir3311	GCGCCGCCGCCACGCCGAGCAA	ATCGCGGCGGCGGTGCGGCTCG	tag86871
novel_sir3312	AGCATGGACACAGCCGAGCCG	GTTCGTACCTGTGTCGGCTCG	tag159950
novel_sir3313	AGCGAGCTAACGAGCCACGAGCTT	GCTCGCTCGATTGCTCGGTGCTCG	tag184218
novel_sir3314	AGCGAGCTAACGAGCCACGAGCTT	GTTCGCTCGATTGCTCGGTGCTCG	tag253975
novel_sir3315	CCGATTGGACCTATGGTAGAGCAG	GAGGCTAACCTGGATACCATCTCG	tag183964
novel_sir3316	ACTACGAATCTTAGCATAGAGCCT	GATGATGCTTAGAATCGTATCTCG	tag221007
novel_sir3317	AGAATTTCTAGGCCATGAGAGCGA	ACTCTTAAAGATCCGGTACTCTCG	tag49152
novel_sir3318	TTTGCTCCGTTACCTAATCTCTCG	ACGAGGCAATGGATTAGAGAGCTA	tag136852
novel_sir3319	ACCTGTTTGGATCCTTAAGAGCTA	GGTGGACAAACCTAGGAATTCTCG	tag157952
novel_sir3320	GAGGTGCCGATCCGGGTATTCTCG	CCACGGCTAGGCCCATAAGAGCAC	tag157480
novel_sir3321	AACGATATCGGGTGGCAAGAGCAC	TATTGCTATAGCCCACCGTTCTCG	tag43287
novel_sir3322	AGGGGTCCGAATGCAAAAGAGCGG	GTTCCCCAGGCTTACGTTTTCTCG	tag162118
novel_sir3323	CGTCGAGTCGGTTTGTCAAAGTCG	AGCTCAGCCAAACAGTTTCAGCTC	tag154926
novel_sir3324	CCTCGAGTCGGTTTGTCAAAGTCG	AGCTCAGCCAAACAGTTTCAGCTC	tag254939
novel_sir3325	CTTCGAGTCGGTTTGTCAAAGTCG	AGCTCAGCCAAACAGTTTCAGCTC	tag259749
novel_sir3326	GTTGGGACCTTTGCCAAGTCG	ACCCTGGAAACGGTTCAGCCG	tag126036
novel_sir3327	CTGCTACGTCGGTCAATTCAGCGT	GGGACGATGCAGCCAGTTAAGTCG	tag263810
novel_sir3328	CTTGATGAGGTAGCCGAACAGTCG	ACTACTCCATCGGCTTGTCAGCCA	tag9717
novel_sir3329	CACCTCCACCGTCCTCAGCCG	CCGTGGAGGTGGCAGGAGTCG	tag115729
novel_sir3330	TGTAGAACCTCAGGCACAACGTCG	ATCTTGGAGTCCGTGTTGCAGCCG	tag223598
novel_sir3331	CAGTGCTGCCAGTCGCCGTCG	CACGACGGTCAGCGGCAGCGC	tag18955

novel_sir3332	CATGTACTCGGCCTCGCAGCTG	CGGTACATGAGCCGGAGCGTCG	tag48279
novel_sir3333	TTTCCCTACATCGCGTCG	AGGGATGTAGCGCAGCTT	tag58299
novel_sir3334	ATTCCCTACATCGCGTCG	AGGGATGTAGCGCAGCTT	tag101317
novel_sir3335	TTTCCCTACATCGCGTCG	AGGGATGTAGCGCAGCTA	tag58299
novel_sir3336	ATTCCCTACATCGCGTCG	AGGGATGTAGCGCAGCTA	tag101317
novel_sir3337	TTTCCCTACATCGCGTCG	AGGGATGTAGCGCAGCTC	tag58299
novel_sir3338	ATTCCCTACATCGCGTCG	AGGGATGTAGCGCAGCTC	tag101317
novel_sir3339	TTTCCCTACATCGCGTCG	AGGGATGTAGCGCAGCTG	tag58299
novel_sir3340	ATTCCCTACATCGCGTCG	AGGGATGTAGCGCAGCTG	tag101317
novel_sir3341	GACACAACACGATGGACGCAGCGG	CGCTGTGTTGTGCTACCTGCGTCG	tag239647
novel_sir3342	TCGCCGGAGGCTTAAAGGTCG	CGGCCTCCGAATTTCCAGCGC	tag225079
novel_sir3343	AGCTCAGCCAAACAGTTCCAGCCT	CCTCGAGTCGGTTTGTCAAGGTCG	tag141096
novel_sir3344	AACTGTTTGGCTGAGCTCCAGCTC	CTTTGACAAACCGACTCGAGGTCG	tag189120
novel_sir3345	GCGTGGTCGAGGCCTAGGTCG	CACCAGCTCCGGATCCAGCTC	tag91500
novel_sir3346	CGCGTGGTCGAGGCCTAGGTCG	GCACCAGCTCCGGATCCAGCTC	tag41388
novel_sir3347	CGACTTTTGCGTCTTTGCCAGCGG	GGGCTGAAAACGCAGAAACGGTCG	tag133045
novel_sir3348	GGGCTGAAATCGCAGAAACGGTCG	CGACTTTAGCGTCTTTGCCAGCGG	tag291407
novel_sir3349	ATGCCTAGTCTGCGGTGCCAGCAT	TGTACGGATCAGACGCCACGGTCG	tag190054
novel_sir3350	GAGCTGAAATCGCAGAGACGGTCG	CGACTTTAGCGTCTCTGCCAGCGG	tag98029
novel_sir3351	GGGCTGAAATCGCAGAGACGGTCG	CGACTTTAGCGTCTCTGCCAGCGG	tag141181
novel_sir3352	GGTATATGCACCATTCAGCGGTCG	ATATACGTGGTAAGTCGCCAGCGG	tag114781
novel_sir3353	AGAAAAAACCACGGGCGCCAGCCA	TCTCTTTTTTGGTGCCCGCGGTCG	tag51769
novel_sir3354	GACGTTCGATCTTAGCGCCAGCGT	CCCTGCAAGCTAGAATCGCGGTCG	tag53983
novel_sir3355	AAGCTACAAAGTGCGAGCCAGCCC	TCTTCGATGTTTCACGCTCGGTCG	tag13007
novel_sir3356	TGCTTCATCTCAACCGGGTCG	GAAGTAGAGTTGGCCCAGCTG	tag71917
novel_sir3357	CGAAGTAGAGTTGGCCCAGCTG	GTGCTTCATCTCAACCGGGTCG	tag239995
novel_sir3358	CAGCCTACGCTAGTATGGTCG	CGGATGCGATCATACCAGCAC	tag163938
novel_sir3359	CAAGACCAAGACCAACCAGCCA	GTGTTCTGGTTCTGGTTGGTCG	tag102615
novel_sir3360	CCGCACCGACGTTTACAGCTT	TTGGCGTGGCTGCAAATGTCG	tag15399
novel_sir3361	CCGTGTGAAGCAGCCCATGTCG	CACACTTCGTCGGGTACAGCGA	tag269986
novel_sir3362	CGTAGAACCTCAGGCACGATGTCG	ATCTTGGAGTCCGTGCTACAGCTG	tag163783
novel_sir3363	CATAGAACTTCAGGCACGATGTCG	ATCTTGAAGTCCGTGCTACAGCGC	tag7889
novel_sir3364	CATAGAACTTCAGGCACGATGTCG	ATCTTGAAGTCCGTGCTACAGCTG	tag7889
novel_sir3365	CAGAGAACTTCAGGTACGATGTCG	CTCTTGAAGTCCATGCTACAGCTG	tag180808

novel_sir3366	CATCCGCTACATGGGCAACTGTCG	AGGCGATGTACCCGTTGACAGCGA	tag260057
novel_sir3367	ACTGAGAACCTCGGTTTACTGTCG	ACTCTTGGAGCCAAATGACAGCCT	tag105353
novel_sir3368	TGGGTAACCCGAGTCGTGCTGTCG	CCATTGGGCTCAGCACGACAGCAC	tag197145
novel_sir3369	ACCGCCCGGGCCAAACGACAGCAC	CCTGGCGGGCCCGGTTTGCTGTCG	tag130463
novel_sir3370	CTTCGACACGGATTCTCACAGCTG	TGGAAGCTGTGCCTAAGAGTGTCG	tag159375
novel_sir3371	TGGGCTTTGGCTTTGGCTGTGTCG	CCGAAACCGAAACCGACACAGCGT	tag73944
novel_sir3372	TTGTCCTCATCGCGATTGTCG	CAGGAGTAGCGCTAACAGCAG	tag132874
novel_sir3373	CATCCGCTGCATGGGCAGTTGTCG	AGGCGACGTACCCGTCAACAGCGA	tag32434
novel_sir3374	CACTTCAGCATCTGCGGGTTGTCG	GAAGTCGTAGACGCCCAACAGCGG	tag186057
novel_sir3375	ACGACAACCTGTCAACAACAGCCC	TCTGCTGTTGGACAGTTGTTGTCG	tag186036
novel_sir3376	TAGGTGAGCTTCACAATTTTGTCG	CCACTCGAAGTGTTAAAACAGCGA	tag282631
novel_sir3377	GAGGTTTCTTGAGGTCCCAATTCG	CCAAAGAACTCCAGGGTTAAGCGT	tag41190
novel_sir3378	GGGTAGTCTTGAGGCTTCAATTCG	CATCAGAACTCCGAAGTTAAGCGT	tag164012
novel_sir3379	AGCCACTCTAACACTATTAAGCCA	ATTCGGTGAGATTGTGATAATTCG	tag4446
novel_sir3380	CCCTGTCACCTCTGGGGGGATTCG	GACAGTGGAGACCCCCCTAAGCCC	tag134675
novel_sir3381	AGTAACTATCCAGCCGGGTATTCG	ATTGATAGGTCGGCCCATAAGCGG	tag199538
novel_sir3382	CCGCCTAAGTTGCCTCCTTCG	CGGATTCAACGGAGGAAGCGG	tag128865
novel_sir3383	ACCTGATACTGGACTCAAGCTTCG	GACTATGACCTGAGTTCGAAGCCT	tag36482
novel_sir3384	CCGCGGGTAACCGGAAGCTTCG	CGCCCATTGGCCTTCGAAGCTT	tag4929
novel_sir3385	GCGCCGGCCGGCGGAGCTTCG	CGGCCGGCCGCCTCGAAGCTC	tag43063
novel_sir3386	GGCGCCGGCCGGCGGAGCTTCG	GCGGCCGGCCGCCTCGAAGCTC	tag127790
novel_sir3387	CTTTTCGGTCACCGCGCTTCG	AAAGCCAGTGGCGCGAAGCTA	tag89758
novel_sir3388	ACTTTTCGGTCACCGCGCTTCG	AAAAGCCAGTGGCGCGAAGCTA	tag104892
novel_sir3389	GATGGGCTTTGCCAAGCTGCTTCG	ACCCGAAACGGTTCGACGAAGCTG	tag275761
novel_sir3390	ATGTCCAAGCCCGGCGACTCTTCG	CAGGTTCGGGCCGCTGAGAAGCGT	tag270427
novel_sir3391	AGTGCTGCGTACGCGCCTTCTTCG	ACGACGCATGCGCGGAAGAAGCGG	tag99721
novel_sir3392	GCGTGCCGCAGTTGAGTCAAGCCA	AACGCACGGCGTCAACTCAGTTCG	tag135043
novel_sir3393	GAGTGGTCAAACGTTGCAAGCAA	TGCTCACCAGTTTGCAACGTTCG	tag260517
novel_sir3394	GATCGGCTGTGCGCGCGACGTTCG	AGCCGACACGCGCGCTGCAAGCGA	tag229099
novel_sir3395	AACTCTAATACTGTTGGCAAGCGC	CCTTGAGATTATGACAACCGTTCG	tag181181
novel_sir3396	AACGTAGAGATGCTGCGCCGTTCG	GCATCTCTACGACGCGGCAAGCCG	tag6372
novel_sir3397	GGCTGGGTGCAATCCCCGCGTTCG	GACCCACGTTAGGGGCGCAAGCCC	tag160547
novel_sir3398	CAGTCTTAGGTTCGATCGTTCG	CAGAATCCAAGCTAGCAAGCGG	tag62609
novel_sir3399	CGGCGGCGGTGCGGCTCGTTCG	CGCCGCCACGCCGAGCAAGCCG	tag206906

novel_sir3400	AGCTAGGTTGTCATCAAAGGTTCG	GATCCAACAGTAGTTTCCAAGCTT	tag175364
novel_sir3401	ACGTCGGCCCAGTCCAAGCCG	GGTGCAGCCGGGTCAGGTTCG	tag114504
novel_sir3402	GGGTGCAGCCGGGTCAGGTTCG	CACGTCGGCCCAGTCCAAGCCG	tag5616
novel_sir3403	CGGAGATTCAGTCTTAGGTTCG	CTCTAAGTCAGAATCCAAGCTA	tag6645
novel_sir3404	CCGGGCACGGTGGCCGGTTCG	CCCGTGCCACCGGCCAAGCCA	tag67777
novel_sir3405	CCGGGCACGGTGGCCGGTTCG	CCCGTGCCACCGGCCAAGCCG	tag67777
novel_sir3406	CCCGGGCACGGTGGCCGGTTCG	GCCCGTGCCACCGGCCAAGCCG	tag49794
novel_sir3407	CTGTCTGGCGGATGCGGTTCG	CAGACCGCCTACGCCAAGCGC	tag204032
novel_sir3408	ATCATTCCAATTTCAACCAAGCTT	TGTAGTAAGGTTAAAGTTGGTTCG	tag248372
novel_sir3409	GATCATATTGCTTAGCCCTGTTCG	AGTATAACGAATCGGGACAAGCTT	tag269606
novel_sir3410	CGTTAGAATGCCACAGGTTGTTCG	AATCTTACGGTGTCCAACAAGCTA	tag265479
novel_sir3411	CCTGTCTCTGGATCAGTTTGTTCG	ACAGAGACCTAGTCAAACAAGCCA	tag209160
novel_sir3412	TCTGCTTCTGGATCAGTTTGTTCG	ACGAAGACCTAGTCAAACAAGCCA	tag3688
novel_sir3413	CCTGCTTTTGGATCAGTTTGTTCG	ACGAAAACCTAGTCAAACAAGCTA	tag103904
novel_sir3414	GAACGTCGGAAACTAAAGCGA	GCCTTGCAGCCTTTGATTTCG	tag144091
novel_sir3415	GAAGTCTATGTCCTAGGAAAGCTG	CCCTTCAGATACAGGATCCTTTCG	tag81162
novel_sir3416	GACACGTCCTAGACTCGAAAGCGA	GACTGTGCAGGATCTGAGCTTTCG	tag279129
novel_sir3417	CCGATCTGCTGGACCCAAGTTTCG	CTAGACGACCTGGGTTCAAAGCCT	tag148392
novel_sir3418	TACTGTGGGCTTACTGCGGTTTCG	GACACCCGAATGACGCCAAAGCAT	tag215723
novel_sir3419	TCGGTGAATCAACGGGTTTCG	CCACTTAGTTGCCCAAAGCCA	tag166996
novel_sir3420	ACCGTTTAGCAGTTTGAAAAGCCT	CGTGGCAAATCGTCAAACTTTTCG	tag26020
novel_sir3421	ACCGTTTAGCAGTTTGAAAAGCCT	TGTGGCAAATCGTCAAACTTTTCG	tag129269
novel_sir3422	CGTGGCAAATCGTCAAACTTTTCG	ACCGTTTAGCAGTTTGAAAAGCAT	tag26020
novel_sir3423	TGTGGCAAATCGTCAAACTTTTCG	ACCGTTTAGCAGTTTGAAAAGCAT	tag129269
novel_sir3424	ACCGTTTAGCAGTTTGAAAAGCGT	CGTGGCAAATCGTCAAACTTTTCG	tag26020
novel_sir3425	ACCGTTTAGCAGTTTGAAAAGCGT	TGTGGCAAATCGTCAAACTTTTCG	tag129269
novel_sir3426	AATTGATCAACTCATGCCTTTTCG	AACTAGTTGAGTACGGAAAAGCAT	tag81372
novel_sir3427	ACGGTCATTCCTGTGTTTTTCCTA	TTTGCCAGTAAGGACACAAAAAGG	tag108822
novel_sir3428	TTTGCCAGTAAGGACACAAAAAGG	ACGGTCATTCCTGTGTTTTTCCTG	tag108822
novel_sir3429	TTTGCCAGTAAGGATACAAAAAGG	ACGGTCATTCCTATGTTTTTCCTA	tag190357
novel_sir3430	ACGGTCATTCCTATATTTTTCCTG	TTTGCCAGTAAGGATATAAAAAGG	tag171293
novel_sir3431	CTCTAGGTACAGTCGTATAAAAGG	GATCCATGTCAGCATATTTTCCTA	tag270515
novel_sir3432	CGGCGTGATCCGTGACAAAGG	CGCACTAGGCACTGTTTCCTC	tag178071
novel_sir3433	AATCTAGGCCATCCGTTGTTCCTT	CTTTAGATCCGGTAGGCAACAAGG	tag271699

novel_sir3434	CGCTGAATCCAGGACAAACCAAGG	GACTTAGGTCCTGTTTGGTTCCAT	tag123295
novel_sir3435	GATCCTATCTGTAGCTCGTTCCTC	TACTAGGATAGACATCGAGCAAGG	tag18414
novel_sir3436	AACTTTTTCGTCACATCGTTCCAA	GGTTGAAAAAGCAGTGTAGCAAGG	tag182324
novel_sir3437	AGTTGAAAAGGCAGTGTAGCAAGG	AACTTTTCCGTCACATCGTTCCAA	tag119405
novel_sir3438	TGTTGAAAAGGCAGTGTAGCAAGG	AACTTTTCCGTCACATCGTTCCAA	tag153633
novel_sir3439	GGTTGAAAAGGCAGTGTAGCAAGG	AACTTTTCCGTCACATCGTTCCAA	tag206637
novel_sir3440	CCGCACTAGGCACCGTTCCCT	ACGGCGTGATCCGTGGCAAGG	tag23392
novel_sir3441	GCTTTAGTTTCCGACGTTCCGA	AGCGAAATCAAAGGCTGCAAGG	tag154280
novel_sir3442	GACTGTTCGGCTACCTCATCAAGG	GACAAGCCGATGGAGTAGTTCCAG	tag159698
novel_sir3443	ACCCTGTCGGATCCGAAGTTCCCC	AATGGGACAGCCTAGGCTTCAAGG	tag58081
novel_sir3444	TGTCTATGCCTATGCGGTAGAAGG	AGATACGGATACGCCATCTTCCTA	tag239005
novel_sir3445	GGGCGGTGGCCGCGGCGAAGG	CGCCACCGGCGCCGCTTCCCT	tag54674
novel_sir3446	CGCCGTCGCCGCCGCTTCCCC	AGGCGGCAGCGGCGGCGAAGG	tag272790
novel_sir3447	CTGTGCAGCCGCCGCTTCCTC	GGGACACGTCGGCGGCGAAGG	tag82368
novel_sir3448	GCTCGATTGCTCAGTGCTCGAAGG	AGCTAACGAGTCACGAGCTTCCTG	tag274815
novel_sir3449	AGCTAACGAGCCACGAGCTTCCTG	GCTCGATTGCTCGGTGCTCGAAGG	tag289372
novel_sir3450	AGGCCGAGGGGAGAAGGAAGG	CGGCTCCCCTCTTCCTTCCTC	tag113096
novel_sir3451	CGGCGGAGCTTCGAGGGAAGG	CGCCTCGAAGCTCCCTTCCCA	tag108199
novel_sir3452	CCGGCGGAGCTTCGAGGGAAGG	CCGCCTCGAAGCTCCCTTCCCA	tag265689
novel_sir3453	CGTACTCGCGGCGCTGGAAGG	ATGAGCGCCGCGACCTTCCAC	tag16784
novel_sir3454	GCTGCGCATCCGAGGAGAATAAGG	ACGCGTAGGCTCCTCTTATTCCTT	tag146152
novel_sir3455	ACCAGCCAAGACTTATTCCAA	GTTGGTCGGTTCTGAATAAGG	tag92621
novel_sir3456	GTTGGTCGGTTCTGAATAAGG	ACCAGCCAAGACTTATTCCTA	tag92621
novel_sir3457	GGTTGGTCGGTTCTGAATAAGG	AACCAGCCAAGACTTATTCCTA	tag12199
novel_sir3458	AACCAGCCAAGACTTATTCCAA	GGTTGGTCGGTTCTGAATAAGG	tag12199
novel_sir3459	GGTTCGAATTTGCTATGACTAAGG	AAGCTTAAACGATACTGATTCCAT	tag193772
novel_sir3460	GGTTCGGCTTTGCTATGACTAAGG	AAGCCGAAACGATACTGATTCCAC	tag9770
novel_sir3461	GATTGTACACTCGGGCTACTAAGG	AACATGTGAGCCCGATGATTCCTG	tag181204
novel_sir3462	AGTTCAGCGGTTATCAGCCTAAGG	AAGTCGCCAATAGTCGGATTCCTG	tag772
novel_sir3463	CGCCGCGCCGTCGATTCCGC	GCGCGGCGCGGCAGCTAAGG	tag267603
novel_sir3464	GCGCCGCGCCGTCGATTCCGC	GGCGCGGCGCGGCAGCTAAGG	tag25825
novel_sir3465	CGCGCCGCGCCGTCGATTCCGC	CGGCGCGGCGCGGCAGCTAAGG	tag2906
novel_sir3466	ATCTAATGGCTGTTTTGGCTAAGG	GATTACCGACAAAACCGATTCCAT	tag191599
novel_sir3467	ATCCTAGGACTAGTCTCATTCCAT	GGTAGGATCCTGATCAGAGTAAGG	tag244633

novel_sir3468	GGTTGGGTCCGGTTGGTAAGG	AACCCAGGCCAACCATTCCAC	tag87819
novel_sir3469	CGGTTGGGTCCGGTTGGTAAGG	CAACCCAGGCCAACCATTCCAC	tag224098
novel_sir3470	GTTCCCCCGGCGGACGTTTTAAGG	AGGGGGCCGCCTGCAAAATTCCAG	tag238924
novel_sir3471	AGTCTTCACCGAGTCTTTGTCCTA	CGTCAGAAGTGGCTCAGAAACAGG	tag48278
novel_sir3472	ATTCCAAACTGAATTTGTAACAGG	AGGTTTGACTTAAACATTGTCCTA	tag269171
novel_sir3473	GTGCCCGCCGAGCCGACACAGG	CGGGCGGCTCGGCTGTGTCCAT	tag229908
novel_sir3474	CCCAATGACTAGGATGGTGTCCAT	TTGGGTTACTGATCCTACCACAGG	tag182111
novel_sir3475	CCCAATGACTAGGATAGTGTCCAT	TTGGGTTACTGATCCTATCACAGG	tag263578
novel_sir3476	ACCGGGTTAAATCTCGCTGTCCGC	TTTGGCCCAATTTAGAGCGACAGG	tag106671
novel_sir3477	ACCGGGTTAAATCTCGCTGTCCGC	ATTGGCCCAATTTAGAGCGACAGG	tag107495
novel_sir3478	CCCAATGACTAGGATGATGTCCAT	TTGGGTTACTGATCCTACTACAGG	tag210750
novel_sir3479	GCTTGGTGGTAAGACGATGTCCGT	GCCGAACCACCATTCTGCTACAGG	tag202128
novel_sir3480	GTTGACCCCAGACTCGGAACCAGG	ACTGGGGTCTGAGCCTTGGTCCTC	tag102446
novel_sir3481	ACCGACACAGTGACAGCCCAGG	GCTGTGTCACTGTCGGGTCCAC	tag58191
novel_sir3482	TACGTTCGGCACGTGAGTTCCAGG	GCAAGCCGTGCACTCAAGGTCCTA	tag95166
novel_sir3483	TGTTGTAGGCTAGTCAAAAGCAGG	AACATCCGATCAGTTTTCGTCCCT	tag258903
novel_sir3484	TGCGCGGCAGTGGAGAGCAGG	GCGCCGTCACCTCTCGTCCTC	tag40860
novel_sir3485	GCTGCCGGTGTCTCCGTCCTC	GGCGACGGCCACAGAGGCAGG	tag207744
novel_sir3486	CAGCGGCGCTGCGGCGGCAGG	CGCCGCGACGCCGCCGTCCAC	tag184700
novel_sir3487	CGTGTCCAACGTTTGACCGTCCGT	AGGCACAGGTTGCAAACTGGCAGG	tag79064
novel_sir3488	CGTGCCCAACGTTTGACCGTCCGT	AGGCACGGGTTGCAAACTGGCAGG	tag262550
novel_sir3489	GCCGAACCCCTATTCTGCTGCAGG	GCTTGGGGATAAGACGACGTCCGT	tag240724
novel_sir3490	GACGACCGCTGTCTTAGCATCAGG	GCTGGCGACAGAATCGTAGTCCGC	tag84887
novel_sir3491	CCGGCTAAAGGTGTCATCAGG	CCGATTTCCACAGTAGTCCCG	tag81256
novel_sir3492	TTCGCGGTCACGGTGTTGATCAGG	GCGCCAGTGCCACAACTAGTCCAT	tag153782
novel_sir3493	CCGGCTAAAGGTGTTATCAGG	CCGATTTCCACAATAGTCCCG	tag25182
novel_sir3494	ATGTGTGTCACACAGAACCTCAGG	CACACAGTGTGTCTTGGAGTCCGT	tag256775
novel_sir3495	ATGTGTGTGACACAGAACCTCAGG	CACACACTGTGTCTTGGAGTCCGT	tag32289
novel_sir3496	ATGTGTACAATACAGAACCTCAGG	CACATGTTATGTCTTGGAGTCCGT	tag278102
novel_sir3497	ATGTGTGTAACGCAGAACCTCAGG	CACACATTGCGTCTTGGAGTCCGT	tag155679
novel_sir3498	GATACACTGCGTCTTGGAGTCCGT	ATCTATGTGACGCAGAACCTCAGG	tag184413
novel_sir3499	ACACACTGCGTCTTGGAGTCCGT	TCTGTGTGACGCAGAACCTCAGG	tag5924
novel_sir3500	GACACACTGCGTCTTGGAGTCCGT	GTCTGTGTGACGCAGAACCTCAGG	tag229205
novel_sir3501	CACACACTGCGTCTTGGAGTCCGT	GTGTGTGTGACGCAGAACCTCAGG	tag102632

novel_sir3502	CACACACTGCGTCTTGGAGTCCGT	ACGTGTGTGACGCAGAACCTCAGG	tag240384
novel_sir3503	TGTGTGTAATGCAGAACCTCAGG	ACACATTACGTCTTGGAGTCCGT	tag190212
novel_sir3504	ATGTGTGTAATGCAGAACCTCAGG	CACACATTACGTCTTGGAGTCCGT	tag255563
novel_sir3505	GTGTGTGTAATGCAGAACCTCAGG	CACACATTACGTCTTGGAGTCCGT	tag266249
novel_sir3506	GAGCATCACCAGCTACGTCAGG	CGTAGTGGTCGATGCAGTCCTC	tag116610
novel_sir3507	AGCCGATCTGAGTTTGAAGTCCCA	GATCGGCTAGACTCAAACTTCAGG	tag111287
novel_sir3508	CTGCCGCTGCAATCCTTCAGG	CGGCGACGTTAGGAAGTCCGG	tag232216
novel_sir3509	AATAGGTCACAGTTCAGG	ATCCAGTGTCAAGTCCAA	tag141082
novel_sir3510	ATCTATTATATACTAAAAGTCCAT	GATAGATAATATATGATTTTCAGG	tag100711
novel_sir3511	CGGTCGCGGGCTCAGCAGAGG	CAGCGCCCGAGTCGTCTCCAC	tag20927
novel_sir3512	GTTGAAAAGTCAGTGTAGCAGAGG	ACTTTTCAGTCACATCGTCTCCAA	tag136649
novel_sir3513	GCTTGCATATCCATCATTTGAGTCTCC	AACGAACGTATAGGTAGTAAACTCAG	tag227427
novel_sir3514	GCTCGGCCATCTCTCCTC	GGCGAGCCGGTAGAGAGG	tag54075
novel_sir3515	GCTCGGCCATCTCTCCTC	AGCGAGCCGGTAGAGAGG	tag209190
novel_sir3516	GCTCGGCCATCTCTCCTA	GGCGAGCCGGTAGAGAGG	tag54075
novel_sir3517	GCTCGGCCATCTCTCCTA	AGCGAGCCGGTAGAGAGG	tag209190
novel_sir3518	CGCTCGGCCATCTCTCCTA	TTGCGAGCCGGTAGAGAGG	tag68516
novel_sir3519	CGCTCGGCCATCTCTCCTA	TGGCGAGCCGGTAGAGAGG	tag180642
novel_sir3520	GGGCGAGCCGGTAGAGAGG	CGCTCGGCCATCTCTCCTC	tag52409
novel_sir3521	TTGCGAGCCGGTAGAGAGG	CGCTCGGCCATCTCTCCTC	tag68516
novel_sir3522	CGGCGAGCCGGTAGAGAGG	CGCTCGGCCATCTCTCCTC	tag164512
novel_sir3523	TGGCGAGCCGGTAGAGAGG	CGCTCGGCCATCTCTCCTC	tag180642
novel_sir3524	AGGCGAGCCGGTAGAGAGG	CGCTCGGCCATCTCTCCTC	tag253076
novel_sir3525	CCGCTCGGCCATCTCTCCTC	AAGGCGAGCCGGTAGAGAGG	tag156527
novel_sir3526	CCGCTCGGCCATCTCTCCTC	TTGGCGAGCCGGTAGAGAGG	tag233080
novel_sir3527	CCGCTCGGCCATCTCTCCTC	ATGGCGAGCCGGTAGAGAGG	tag281926
novel_sir3528	CCGCTCGGCCATCTCTCCTA	AAGGCGAGCCGGTAGAGAGG	tag156527
novel_sir3529	CCGCTCGGCCATCTCTCCTA	TTGGCGAGCCGGTAGAGAGG	tag233080
novel_sir3530	CCGCTCGGCCATCTCTCCTA	ATGGCGAGCCGGTAGAGAGG	tag281926
novel_sir3531	ACCGCTCGGCCATCTCTCCTC	CTTGGCGAGCCGGTAGAGAGG	tag205135
novel_sir3532	ACCGCTCGGCCATCTCTCCTC	ATTGGCGAGCCGGTAGAGAGG	tag208628
novel_sir3533	ACCGCTCGGCCATCTCTCCTA	CTTGGCGAGCCGGTAGAGAGG	tag205135
novel_sir3534	ACCGCTCGGCCATCTCTCCTA	ATTGGCGAGCCGGTAGAGAGG	tag208628
novel_sir3535	AACCGCTCGGCCATCTCTCCTC	TCTTGGCGAGCCGGTAGAGAGG	tag42836

novel_sir3536	AACCGCTCGGCCATCTCTCCTC	ACTTGGCGAGCCGGTAGAGAGG	tag94637
novel_sir3537	AACCGCTCGGCCATCTCTCCTC	CCTTGGCGAGCCGGTAGAGAGG	tag178767
novel_sir3538	GAACCGCTCGGCCATCTCTCCTC	AACTTGGCGAGCCGGTAGAGAGG	tag36821
novel_sir3539	CGCCTTGAACCGCTCGGCCATCTCTCC	CTGCGGAACTTGGCGAGCCGGTAGAG	tag45013
novel_sir3540	CGCCTTGAACCGCTCGGCCATCTCTCC	GTGCGGAACTTGGCGAGCCGGTAGAG	tag85866
novel_sir3541	CGCCTTGAACCGCTCGGCCATCTCTCC	ATGCGGAACTTGGCGAGCCGGTAGAG	tag146832
novel_sir3542	GATGCGGAACTTGGCGAGCCGGTAGA	ACGCCTTGAACCGCTCGGCCATCTCTC	tag257371
novel_sir3543	GGTGAGTCGGTAGAGAGG	ACTCAGCCATCTCTCCCC	tag68507
novel_sir3544	AGTGAGTCGGTAGAGAGG	ACTCAGCCATCTCTCCCC	tag267103
novel_sir3545	GGGTGAGTCGGTAGAGAGG	CACTCAGCCATCTCTCCCC	tag67066
novel_sir3546	TTGTGAGTCGGTAGAGAGG	CACTCAGCCATCTCTCCCC	tag98461
novel_sir3547	CGGTGAGTCGGTAGAGAGG	CACTCAGCCATCTCTCCCC	tag143940
novel_sir3548	AGGTGAGTCGGTAGAGAGG	CACTCAGCCATCTCTCCCC	tag245959
novel_sir3549	TGGTGAGTCGGTAGAGAGG	CACTCAGCCATCTCTCCCC	tag254120
novel_sir3550	ATGGTGAGTCGGTAGAGAGG	CCACTCAGCCATCTCTCCCC	tag235
novel_sir3551	TCGGTGAGTCGGTAGAGAGG	CCACTCAGCCATCTCTCCCC	tag42038
novel_sir3552	TAGGTGAGTCGGTAGAGAGG	CCACTCAGCCATCTCTCCCC	tag47294
novel_sir3553	CAGGTGAGTCGGTAGAGAGG	CCACTCAGCCATCTCTCCCC	tag78649
novel_sir3554	AAGGTGAGTCGGTAGAGAGG	CCACTCAGCCATCTCTCCCC	tag127759
novel_sir3555	GTGGTGAGTCGGTAGAGAGG	CCACTCAGCCATCTCTCCCC	tag180796
novel_sir3556	GAGGTGAGTCGGTAGAGAGG	CCACTCAGCCATCTCTCCCC	tag197456
novel_sir3557	CCGGTGAGTCGGTAGAGAGG	CCACTCAGCCATCTCTCCCC	tag242028
novel_sir3558	TTGGTGAGTCGGTAGAGAGG	CCACTCAGCCATCTCTCCCC	tag273193
novel_sir3559	AGTCCACTCAGCCATCTCTCCCC	CATCAGGTGAGTCGGTAGAGAGG	tag223021
novel_sir3560	CTTGGTGAGTCGGTAGAGAGG	ACCACTCAGCCATCTCTCCAC	tag85774
novel_sir3561	GTTGGTGAGTCGGTAGAGAGG	ACCACTCAGCCATCTCTCCAC	tag93543
novel_sir3562	ATTGGTGAGTCGGTAGAGAGG	ACCACTCAGCCATCTCTCCAC	tag188729
novel_sir3563	CGGCCGCGGAAAAAGGGAGAGG	CGGCGCCTTTTTCCCTCTCCGT	tag199967
novel_sir3564	CCGCGACCAGCGAGGGAGAGG	CGCTGGTCGCTCCCTCTCCCA	tag92027
novel_sir3565	GAGCTGACGAATCACTAGAGG	CGACTGCTTAGTGATCTCCTC	tag239864
novel_sir3566	GTTGAAAAGTCAGTGTAGTAGAGG	ACTTTTCAGTCACATCATCTCCAA	tag121631
novel_sir3567	TGGACGAAGCAGACGACGAGG	CTGCTTCGTCTGCTGCTCCGA	tag140068
novel_sir3568	GTGGACGAAGCAGACGACGAGG	CCTGCTTCGTCTGCTGCTCCGA	tag214808
novel_sir3569	GACGGGTAGCCGCAGGCGAGG	GCCCATCGGCGTCCGCTCCTC	tag201545

novel_sir3570	CGGCCGACCTCTCTAGCTCCGG	CGGCCGGCTGGAGAGATCGAGG	tag147273
novel_sir3571	CAGGGGTCTTCCGGCTAGCTCCAT	GAGTCCCCAGAAGGCCGATCGAGG	tag63003
novel_sir3572	CAGGGGTCTTCCGGCTAGCTCCAT	GGGTCCCCAGAAGGCCGATCGAGG	tag101422
novel_sir3573	CAGGGGTCTTCCGGCTAGCTCCAT	GTGTCCCCAGAAGGCCGATCGAGG	tag262200
novel_sir3574	AAAATGGCCCACTTGGAGCTCCCG	GTTTTTACCGGGTGAACCTCGAGG	tag99503
novel_sir3575	GGTCGAGGCCTAGGTCGAGG	AGCTCCGGATCCAGCTCCTG	tag151482
novel_sir3576	TGGTCGAGGCCTAGGTCGAGG	CAGCTCCGGATCCAGCTCCTG	tag284155
novel_sir3577	GTGGTCGAGGCCTAGGTCGAGG	CCAGCTCCGGATCCAGCTCCTG	tag173948
novel_sir3578	AGGAGGACCGCGTGGTCGAGG	CTCCTGGCGCACCAGCTCCGG	tag281342
novel_sir3579	CCGGCCGGCGGAGCTTCGAGG	CCGGCCGCCTCGAAGCTCCCT	tag16278
novel_sir3580	GCCGGCCGGCGGAGCTTCGAGG	GCCGGCCGCCTCGAAGCTCCCT	tag143223
novel_sir3581	CGGCGTGATCCGTGACAAAGGAGG	CGCACTAGGCACTGTTTCCTCCCT	tag282946
novel_sir3582	AGCCTCTCTGGCGCCGTCCTCCTG	GGTCGGAGAGACCGCGGCAGGAGG	tag35633
novel_sir3583	AGTGGCTAGGAGGGCATCCTCCCT	GTTCACCGATCCTCCCGTAGGAGG	tag289542
novel_sir3584	ATTTAGTCGACATACGGCCTCCGA	CGTAAATCAGCTGTATGCCGGAGG	tag189297
novel_sir3585	CATGATGCTTAGACCTGTCGGAGG	ACTACGAATCTGGACAGCCTCCTG	tag10186
novel_sir3586	ACTAGGCACCGTTCCCTCCCT	CGTGATCCGTGGCAAGGGAGG	tag208698
novel_sir3587	CGCACTAGGCACCGTTCCCTCCCT	CGGCGTGATCCGTGGCAAGGGAGG	tag121424
novel_sir3588	TATGGGGCTCTTACCGGGAGG	ACCCCGAGAATGGCCCTCCGC	tag95586
novel_sir3589	CGTCGCGGCGCCGCCCTCCTC	CGGCAGCGCCGCGGCGGGAGG	tag156734
novel_sir3590	AAGCCGCCGGCCTCCCCTCCTC	GGTTCGGCGGCCGGAGGGGAGG	tag291690
novel_sir3591	CCGCCACCGCTGGACCTCCCC	CCGGCGGTGGCGACCTGGAGG	tag191660
novel_sir3592	CGCCGGCCGCCGGACCTCCTC	CAGCGGCCGGCGGCCTGGAGG	tag172261
novel_sir3593	CATCAGCAAGTGCCGTGGAGG	AGTCGTTCACGGCACCTCCTC	tag218233
novel_sir3594	CTGCTGTTAGCGCTACTCCTG	ATGACGACAATCGCGATGAGG	tag184136
novel_sir3595	AACCTCCTTTCATAATACTCCCA	AATTGGAGGAAAGTATTATGAGG	tag8785
novel_sir3596	AATTGGAGGAAAGTATTATGAGG	AACCTCCTTTCATAATACTCCCT	tag8785
novel_sir3597	CATTGGAGGAAAGTATTATGAGG	AACCTCCTTTCATAATACTCCCT	tag109253
novel_sir3598	AACCTCCTTTCATAATACTCCCC	AATTGGAGGAAAGTATTATGAGG	tag8785
novel_sir3599	GATTAACCTCCTTTCATAATACTCCCT	TACTAATTGGAGGAAAGTATTATGAG[illegible]	tag174540
novel_sir3600	GATTAACCTCCTTTCATAATACTCCCT	AACTAATTGGAGGAAAGTATTATGAG[illegible]	tag255615
novel_sir3601	ACTTGTGTGCGGGCGACACTCCTC	GGTGAACACACGCCCGCTGTGAGG	tag74251
novel_sir3602	CACCTCTAGCTTCAAACTCCGA	CTGTGGAGATCGAAGTTTGAGG	tag282576
novel_sir3603	CCTGGGAGACCAGAATGCAATAGG	ACCCTCTGGTCTTACGTTATCCTT	tag121031

novel_sir3604	ATCTCCAACAGTCTTCTTATCCTA	CATAGAGGTTGTCAGAAGAATAGG	tag119135
novel_sir3605	AACTTTTCGCACGGTGCTCATAGG	GAAAAGCGTGCCACGAGTATCCAA	tag46138
novel_sir3606	GCCACTGACTTTGTCGATATCCAT	TACGGTGACTGAAACAGCTATAGG	tag288352
novel_sir3607	AAGTGAAAAGAGCATTATAGG	CACTTTTCTCGTAATATCCTT	tag164212
novel_sir3608	GCCGACAGGAGCTGGATCCGG	TGCGGCTGTCCTCGACCTAGG	tag155786
novel_sir3609	GGCTGCGGCTGTCCTCGACCTAGG	GACGCCGACAGGAGCTGGATCCGG	tag136277
novel_sir3610	GCGCACCAGCTCCGGATCCAG	ACCGCGTGGTCGAGGCCTAGG	tag122857
novel_sir3611	ATACTTCGACTGCAGAGGATCCTG	TGTATGAAGCTGACGTCTCCTAGG	tag75996
novel_sir3612	AGGAGGGCGTTCTTTAGCTAGG	CTCCCGCAAGAAATCGATCCGG	tag185724
novel_sir3613	CACGGTGAGGCAGCGGCTAGG	GCCACTCCGTCGCCGATCCGT	tag97526
novel_sir3614	TGGATGCCTAGTCTGCGGGCTAGG	CTACGGATCAGACGCCCGATCCGT	tag3182
novel_sir3615	AACTAAATAATCGTAGATCCGT	TATTGATTTATTAGCATCTAGG	tag50953
novel_sir3616	TCTGCGACGTCGAAGCCTTCTAGG	ACGCTGCAGCTTCGGAAGATCCAC	tag161938
novel_sir3617	ACGCTGCAGCTTCGGAAGATCCGC	TCTGCGACGTCGAAGCCTTCTAGG	tag161938
novel_sir3618	GTTTGAACAAGCTGACACAGTAGG	AACTTGTTCGACTGTGTCATCCCA	tag177139
novel_sir3619	GTTTGAACAAGCTGACACAGTAGG	AACTTGTTCGACTGTGTCATCCTA	tag177139
novel_sir3620	GTTTGAACAAGCTGACACAGTAGG	AACTTGTTCGACTGTGTCATCCCG	tag177139
novel_sir3621	TCTACAAAGATGCCAGGCAGTAGG	ATGTTTCTACGGTCCGTCATCCGT	tag84315
novel_sir3622	CTCGTCGCCGGCCGTCATCCGT	GAGAGCAGCGGCCGGCAGTAGG	tag255159
novel_sir3623	AGCTCCAACAGACTCTCCATCCGA	GTTCGAGGTTGTCTGAGAGGTAGG	tag178576
novel_sir3624	GATCCTGAGCTGCCATCCAC	GACTAGGACTCGACGGTAGG	tag114904
novel_sir3625	TGCGGGCTAGGCATCGGGGTAGG	GCCCGATCCGTAGCCCCATCCAA	tag168031
novel_sir3626	CAGGCTTTTGGGGAAGGCTGTAGG	CCGAAAACCCCTTCCGACATCCGG	tag156814
novel_sir3627	AACTACATTCCTGTCATAATCCTA	TGTTGATGTAAGGACAGTATTAGG	tag156261
novel_sir3628	AGGCGTTCAGTCATAATCCGG	TCTCCGCAAGTCAGTATTAGG	tag55336
novel_sir3629	GAGGCGTTCAGTCATAATCCGG	ATCTCCGCAAGTCAGTATTAGG	tag228281
novel_sir3630	CCGCTTCCTCCGTTGAATCCGC	GCGGCGAAGGAGGCAACTTAGG	tag207818
novel_sir3631	GATGAGGACAGGCCCCTACTTAGG	ACTCCTGTCCGGGGATGAATCCGC	tag245163
novel_sir3632	ACTTACAGTCCTGTCAGAATCCTA	TGTGAATGTCAGGACAGTCTTAGG	tag203640
novel_sir3633	TGCGGAGATTCAGTCTTAGG	GCCTCTAAGTCAGAATCCAA	tag252358
novel_sir3634	TTGCGGAGATTCAGTCTTAGG	CGCCTCTAAGTCAGAATCCAA	tag241821
novel_sir3635	GACTTGCGGAGATTCAGTCTTAGG	GAACGCCTCTAAGTCAGAATCCAA	tag268637
novel_sir3636	CTGACGAAGTGATTAGTTAGG	CTGCTTCACTAATCAATCCAA	tag157510
novel_sir3637	AACTTTTCGCACGGTGCTTTTAGG	GAAAAGCGTGCCACGAAAATCCAA	tag273278

novel_sir3638	ACTTACATTTCTATCAAAATCCTA	TGTGAATGTAAAGATAGTTTTAGG	tag229428
novel_sir3639	TGTGAATGTAAGGATAGTTTTAGG	ACTTACATTCCTATCAAAATCCTA	tag18257
novel_sir3640	ACTTGCATTCCTACCAAAATCCTG	TGTGAACGTAAGGATGGTTTTAGG	tag202320
novel_sir3641	GGTAGATATATCTGGTGAAAACGG	ATCTATATAGACCACTTTTGCCAT	tag112023
novel_sir3642	CCTGTGCTTTTGAGTCCCAAACGG	ACACGAAAACTCAGGGTTTGCCTT	tag72980
novel_sir3643	GGTCGGCTTTCAGATCAAACGG	AGCCGAAAGTCTAGTTTGCCTC	tag7248
novel_sir3644	TCGGCTGACGAGCTACAACGG	CCGACTGCTCGATGTTGCCGC	tag129793
novel_sir3645	CCTAGGCAAACGACCCAACGG	ATCCGTTTGCTGGGTTGCCTC	tag110928
novel_sir3646	CCGACTGCTCGACGTTGCCGC	TCGGCTGACGAGCTGCAACGG	tag85820
novel_sir3647	GTCTTTTTTTTCACACCGAACGG	GAAAAAAAAAGTGTGGCTTGCCAT	tag201311
novel_sir3648	GGGGCAAACGGTGCAGTCGAACGG	CCGTTTGCCACGTCAGCTTGCCGC	tag98343
novel_sir3649	GTGGCAGACGGTGCAGTCGAACGG	CCGTCTGCCACGTCAGCTTGCCGC	tag188031
novel_sir3650	CGGAGCAAACTGGCACCTGAACGG	CTCGTTTGACCGTGGACTTGCCTA	tag210525
novel_sir3651	CAGTGTAGATGGTATTGTGAACGG	CACATCTACCATAACACTTGCCCT	tag136258
novel_sir3652	CGCGCTTCGATGGCACACGG	GCGAAGCTACCGTGTGCCGG	tag213290
novel_sir3653	CCGCGCTTCGATGGCACACGG	CGCGAAGCTACCGTGTGCCGG	tag187595
novel_sir3654	GCTGTACCCGACGAAGTGTGCCTT	AGCGACATGGGCTGCTTCACACGG	tag186468
novel_sir3655	ACGATCGTGATTGGTGCCAC	AATGCTAGCACTAACCACGG	tag129840
novel_sir3656	AGGTGTATGTCACAGATACCACGG	CACATACAGTGTCTATGGTGCCAT	tag135347
novel_sir3657	CTTAAAAGAAGCAAGGCACGG	ATTTTCTTCGTTCCGTGCCAC	tag94154
novel_sir3658	CTATTCGAATAGCCGTGCCCG	TTGATAAGCTTATCGGCACGG	tag18540
novel_sir3659	ATTTTGATAAGCTTATCGGCACGG	AAACTATTCGAATAGCCGTGCCCG	tag166880
novel_sir3660	TTCGGGGGTTCCCGGGCACGG	GCCCCCAAGGGCCCGTGCCAC	tag125275
novel_sir3661	GTTCGGGGGTTCCCGGGCACGG	AGCCCCCAAGGGCCCGTGCCAC	tag181603
novel_sir3662	TGTATGAAAGAATTATGGGCACGG	ATACTTTCTTAATACCCGTGCCAA	tag185699
novel_sir3663	TGGGATGACTACTGGCACGG	CCTACTGATGACCGTGCCGC	tag94460
novel_sir3664	ATGGGATGACTACTGGCACGG	CCCTACTGATGACCGTGCCGC	tag80444
novel_sir3665	AATGGGATGACTACTGGCACGG	ACCCTACTGATGACCGTGCCGC	tag90787
novel_sir3666	GGAGGGAGGAAACAGTGCCTA	CCCCTCCCTCCTTTGTCACGG	tag207653
novel_sir3667	CGTGGTCGCGCTCTCTGCCGG	GCGCACCAGCGCGAGAGACGG	tag143073
novel_sir3668	GGAGTGGAGTTGTGGAGCTGCCTA	GACCTCACCTCAACACCTCGACGG	tag251590
novel_sir3669	CGTGAGATGTTTATGAGGATACGG	ACTCTACAAATACTCCTATGCCGC	tag180448
novel_sir3670	CTTGAACATCTTCAGCTACTACGG	ACTTGTAGAAGTCGATGATGCCGC	tag112392
novel_sir3671	CTCGAACAACGATGTTGGCTACGG	GCTTGTTGCTACAACCGATGCCCG	tag32199

novel_sir3672	ATTTAACAAGAAGCATGCCAG	GGTAAATTGTTCTTCGTACGG	tag231704
novel_sir3673	CCTGAATGTTACATTTATGTACGG	ACTTACAATGTAAATACATGCCGA	tag31135
novel_sir3674	CGAAACGAAAAGAATGCCTC	TAGCTTTGCTTTTCTTACGG	tag249621
novel_sir3675	ACCTAAGCGCTACGTCAATGCCAC	GGTGGATTCGCGATGCAGTTACGG	tag102537
novel_sir3676	GGTGCATTCGCGGTGCAGTTACGG	ACGTAAGCGCCACGTCAATGCCAC	tag265910
novel_sir3677	CTCTGCCACTTACAATGCCCC	GTGAGACGGTGAATGTTACGG	tag139710
novel_sir3678	ACTCTGCCACTTACAATGCCCC	GGTGAGACGGTGAATGTTACGG	tag66591
novel_sir3679	ACTGAAAAAGAGTGTACAAACCGG	ACTTTTTCTCACATGTTTGGCCAT	tag51517
novel_sir3680	ACTTTTCTAAACATGTTTGGCCGT	ACTGAAAAGATTTGTACAAACCGG	tag198278
novel_sir3681	ACTTTTCGACTAACGTTTGGCCAT	ACTGAAAAGCTGATTGCAAACCGG	tag48730
novel_sir3682	CGGCCGGGCCGGCTTGGCCGG	CAGCCGGCCCGGCCGAACCGG	tag225334
novel_sir3683	TGCTCATATATCGGAACCGG	GAGTATATAGCCTTGGCCGA	tag47324
novel_sir3684	TTGCTCATATATCGGAACCGG	CGAGTATATAGCCTTGGCCGA	tag35586
novel_sir3685	GTTGCTCATATATCGGAACCGG	ACGAGTATATAGCCTTGGCCGA	tag134720
novel_sir3686	CCGGCTGCACCCGGTGAACCGG	CCGACGTGGGCCACTTGGCCAC	tag45927
novel_sir3687	ACACTTTACTAGCACATTGGCCCA	GGTGTGAAATGATCGTGTAACCGG	tag146256
novel_sir3688	TGCTTGGCACTTTCGCACCGG	GAACCGTGAAAGCGTGGCCTA	tag271173
novel_sir3689	ATGCTTGGCACTTTCGCACCGG	CGAACCGTGAAAGCGTGGCCTA	tag132618
novel_sir3690	GATTAGCGATCTATTCTGCACCGG	AATCGCTAGATAAGACGTGGCCCA	tag223923
novel_sir3691	AGACACAATCGCCCACGTGGCCTG	CGTCTGTGTTAGCGGGTGCACCGG	tag255076
novel_sir3692	AGCAAGTCCGCTCTGGCCCT	TATCGTTCAGGCGAGACCGG	tag285903
novel_sir3693	CCTATCGTTCAGGCGAGACCGG	ATAGCAAGTCCGCTCTGGCCCT	tag75647
novel_sir3694	ACCTATCGTTCAGGCGAGACCGG	GATAGCAAGTCCGCTCTGGCCCT	tag276975
novel_sir3695	ACTGATTATTTACATGCTGGCCTT	TTTGACTAATAAATGTACGACCGG	tag170993
novel_sir3696	GCGGAGACGGCAGCTGGCCCC	CCCGCCTCTGCCGTCGACCGG	tag171042
novel_sir3697	CGGAGGTGCAAGCGAGAGTACCGG	CTCCACGTTCGCTCTCATGGCCTA	tag266127
novel_sir3698	CAATTCAACGTAATTAATGGCCTT	TAGTTAAGTTGCATTAATTACCGG	tag242870
novel_sir3699	TTTCGATAGTCTACATTCACCCGG	AGCTATCAGATGTAAGTGGGCCCG	tag46629
novel_sir3700	TACTCGTAGAAGCTTCTGACCCGG	GAGCATCTTCGAAGACTGGGCCGG	tag202070
novel_sir3701	GATTTTTATACAGGTTCGGGCCCC	TCCTAAAAATATGTCCAAGCCCGG	tag64218
novel_sir3702	GATTTTTATACAGGTTCGGGCCCC	CCCTAAAAATATGTCCAAGCCCGG	tag216872
novel_sir3703	CCCGCTACATATGTCCAAGCCCGG	GCGATGTATACAGGTTCGGGCCGC	tag28948
novel_sir3704	GCCGCTACATATGTCCAAGCCCGG	GCGATGTATACAGGTTCGGGCCGC	tag117977
novel_sir3705	ACACATACTATTATATCGGGCCTC	TATGTGTATGATAATATAGCCCGG	tag34249

novel_sir3706	CCTCCTCCTCGTCCGGGCCCG	GAGGAGGAGGAGCAGGCCCGG	tag261524
novel_sir3707	TATGGTGAAACATTGCAGGCCCGG	ACCACTTTGTAACGTCCGGGCCCA	tag231433
novel_sir3708	GACTAGATACCTGTGACGGGCCAT	CACTGATCTATGGACACTGCCCGG	tag224014
novel_sir3709	CACCATCCAGCGGAGGGCCAT	GAGTGGTAGGTCGCCTCCCGG	tag26469
novel_sir3710	TGTGCCACACATGTGCGCTCCCGG	ACGGTGTGTACACGCGAGGGCCTG	tag263217
novel_sir3711	GCGCGGGCGAGCGGAAGCCGG	CGCCCGCTCGCCTTCGGCCTC	tag7782
novel_sir3712	CGCCCGCCCGCCTTCGGCCTC	GCGCGGGCGGGCGGAAGCCGG	tag53140
novel_sir3713	AGGACTATTGTGGAATTCGGCCGA	GGTCCTGATAACACCTTAAGCCGG	tag206144
novel_sir3714	ACTGAGATAACGGTGCGCAGCCGG	ACTCTATTGCCACGCGTCGGCCAT	tag203872
novel_sir3715	CGGTGCAGTCGATCCCCTAGCCGG	CACGTCAGCTAGGGGATCGGCCCG	tag156103
novel_sir3716	TGGTGCAGTCGATCCCCTAGCCGG	CACGTCAGCTAGGGGATCGGCCCG	tag159030
novel_sir3717	AGGGAGCTTCGAGGCGGCCGG	CTTCCCTCGAAGCTCCGCCGG	tag41876
novel_sir3718	AAGGGAGCTTCGAGGCGGCCGG	CCTTCCCTCGAAGCTCCGCCGG	tag274211
novel_sir3719	GCTGCTTCCCCCTCCGCGCCGG	ACGAAGGGGGAGGCGCGGCCGG	tag33617
novel_sir3720	CTCGATCTCTCCAGCCGGCCGG	GCTAGAGAGGTCGGCCGGCCTA	tag217388
novel_sir3721	GCTTCGAGGCGGCCGGCCGC	CTCGAAGCTCCGCCGGCCGG	tag17500
novel_sir3722	AGCTTCGAGGCGGCCGGCCGC	CCTCGAAGCTCCGCCGGCCGG	tag178452
novel_sir3723	GAGCTTCGAGGCGGCCGGCCGC	CCCTCGAAGCTCCGCCGGCCGG	tag241320
novel_sir3724	GGAGCTTCGAGGCGGCCGGCCGC	TCCCTCGAAGCTCCGCCGGCCGG	tag127334
novel_sir3725	CTCCCCCTTCGTCGCCGGCCGC	CGGAGGGGGAAGCAGCGGCCGG	tag149144
novel_sir3726	CCACCTCTCGTCGCCGGCCGT	GCGGTGGAGAGCAGCGGCCGG	tag68122
novel_sir3727	GCCACCTCTCGTCGCCGGCCGT	GGCGGTGGAGAGCAGCGGCCGG	tag105253
novel_sir3728	CGTCCTCTGTGCGCCGGCCTC	GAGCAGGAGACACGCGGCCGG	tag139318
novel_sir3729	TCTCCCTTTTTCCGCGGCCGG	AGGGAAAAAGGCGCCGGCCGG	tag72743
novel_sir3730	CTCTCCCTTTTTCCGCGGCCGG	GAGGGAAAAAGGCGCCGGCCGG	tag269890
novel_sir3731	CGGGTCAGGTTCGGCGGCCGG	CCAGTCCAAGCCGCCGGCCTC	tag161409
novel_sir3732	AGCATTTAGTCGACATACGGCCTC	AGTCGTAAATCAGCTGTATGCCGG	tag95595
novel_sir3733	AGATGACTTCCTTGTGACGGCCCG	ACTCTACTGAAGGAACACTGCCGG	tag112365
novel_sir3734	GAAAGTGACGTGGCAGACGGCCGT	CGCTTTCACTGCACCGTCTGCCGG	tag258596
novel_sir3735	GAAAATGACGTGGCAGACGGCCGT	CGCTTTTACTGCACCGTCTGCCGG	tag137099
novel_sir3736	ACTTGCTATAAATCTATAGGCCGC	GTTGAACGATATTTAGATATCCGG	tag84035
novel_sir3737	TACGCTTGTCAACGAATCCTCCGG	GCGAACAGTTGCTTAGGAGGCCCT	tag140915
novel_sir3738	CAGATGTGCTGGCCCTAGCTCCGG	CTACACGACCGGGATCGAGGCCGC	tag72907
novel_sir3739	TAGATGTGCTGGCCCTAGCTCCGG	CTACACGACCGGGATCGAGGCCGC	tag195427

novel_sir3740	TGGTGCTGTATGGCCTTTCTCCGG	CACGACATACCGGAAAGAGGCCGT	tag85269
novel_sir3741	AGGATTTAGACAGGTTCAGGCCCT	GTTCCTAAATCTGTCCAAGTCCGG	tag121239
novel_sir3742	AGGATTTAGACAGGTTCAGGCCCT	TTTCCTAAATCTGTCCAAGTCCGG	tag278269
novel_sir3743	GTCCGTGGGTGCCAGGTAGTCCGG	GGCACCCACGGTCCATCAGGCCTT	tag184986
novel_sir3744	CAGATCCAGTCAGGCCTCGTCCGG	CTAGGTCAGTCCGGAGCAGGCCAG	tag84843
novel_sir3745	CAGGGATTTTCTTGCACTGTCCGG	CCCTAAAAGAACGTGACAGGCCAC	tag130532
novel_sir3746	TAGCATCTGCCACGGTGTCCGG	CGTAGACGGTGCCACAGGCCTT	tag90512
novel_sir3747	GCGTATCCTATGCACACAGGCCCT	CTCGCATAGGATACGTGTGTCCGG	tag36096
novel_sir3748	ACGTATCCTATGCACACAGGCCTT	GTTGCATAGGATACGTGTGTCCGG	tag199350
novel_sir3749	TATGATAGTACATGTGCATTCCGG	ACTATCATGTACACGTAAGGCCCG	tag280449
novel_sir3750	ACTCAATGACTAGAATAAGGCCAT	GTTGAGTTACTGATCTTATTCCGG	tag202564
novel_sir3751	CGGCGCGAGCGGCAGTTCCGG	CGCGCTCGCCGTCAAGGCCAT	tag127786
novel_sir3752	CGTCTGCCACGTCATTTTCGCCGC	AGGCAGACGGTGCAGTAAAAGCGG	tag128914
novel_sir3753	CGTCTGCCACGTCATTTTCGCCGC	CAGCAGACGGTGCAGTAAAAGCGG	tag179275
novel_sir3754	CGTCTGCCACGTCATTTTCGCCGC	CGGCAGACGGTGCAGTAAAAGCGG	tag250682
novel_sir3755	AGTCTAATAATCGGACCTCAAGCGG	AGATTATTAGCCTGGAGTTCGCCAA	tag169720
novel_sir3756	AATGGCTTTCAGCAACCACAGCGG	ACCGAAAGTCGTTGGTGTCGCCTG	tag90836
novel_sir3757	CTCAGCTACGACTCGTGTCGCCCC	CTGAGTCGATGCTGAGCACAGCGG	tag73663
novel_sir3758	TGCCTTTTTATCTCCTCACAGCGG	GGAAAAATAGAGGAGTGTCGCCCG	tag275078
novel_sir3759	GCGCCTCCCCCTTCGTCGCCGG	GGCGCGGAGGGGGAAGCAGCGG	tag55840
novel_sir3760	ACCGATCCACTGCGTCGTCGCCGC	CGTGGCTAGGTGACGCAGCAGCGG	tag3454
novel_sir3761	CGCCGCCACCTCTCGTCGCCGG	TGGCGGCGGTGGAGAGCAGCGG	tag91497
novel_sir3762	CTCGTTCGGCGGCGGCAGCGG	GCAAGCCGCCGCCGTCGCCGG	tag206926
novel_sir3763	GCTCGTTCGGCGGCGGCAGCGG	AGCAAGCCGCCGCCGTCGCCGG	tag198053
novel_sir3764	GAGTGACAACTGTCCATGCAGCGG	CACTGTTGACAGGTACGTCGCCTA	tag39973
novel_sir3765	CCTCTCGCTATGCTAAGTCGCCAT	AGGGAGAGCGATACGATTCAGCGG	tag2389
novel_sir3766	GCGCGGCGGCGAGCTAGAGCGG	CGCCGCCGCTCGATCTCGCCGC	tag63841
novel_sir3767	CCTCTCCGAGCGTGCTCGCCGG	AGGGAGAGGCTCGCACGAGCGG	tag261637
novel_sir3768	ACCCACCGCCGTGCTCGCCTG	CCTGGGTGGCGGCACGAGCGG	tag290957
novel_sir3769	CTCTCCGTGCGCGCTCGCCGG	GGGAGAGGCACGCGCGAGCGG	tag177203
novel_sir3770	CCTGGGTGGCGGCGCGAGCGG	ACCCACCGCCGCGCTCGCCTG	tag88840
novel_sir3771	GGCGGCGGCGGCCTCGAGCGG	GCCGCCGCCGGAGCTCGCCTG	tag76668
novel_sir3772	CAGCCGAGGAGCCAGGAGCGG	CGGCTCCTCGGTCCTCGCCCG	tag176605
novel_sir3773	CTCCGTCCTCGACCTCGCCCG	CAGAGGCAGGAGCTGGAGCGG	tag199867

novel_sir3774	CGGCAGAGGCGGGGTGAGCGG	CGTCTCCGCCCCACTCGCCGG	tag233376
novel_sir3775	CGAAGACTGAGTCAACATCGCCAC	TGGCTTCTGACTCAGTTGTAGCGG	tag69008
novel_sir3776	GATCCGGAGCTGGTGCGCCAG	ACCTAGGCCTCGACCACGCGG	tag262359
novel_sir3777	CGGCGGCTGTCCTCGACGCGG	CGCCGACAGGAGCTGCGCCGC	tag117236
novel_sir3778	CCGCCGACAGGAGCTGCGCCGC	GCGGCGGCTGTCCTCGACGCGG	tag222264
novel_sir3779	TTTAGCTGACATCGTATACCGCGG	ATCGACTGTAGCATATGGCGCCAT	tag170258
novel_sir3780	ATCGACTATAGCATATGGCGCCAT	TTTAGCTGATATCGTATACCGCGG	tag58995
novel_sir3781	TGTTCGATCGTTCGCCGCGG	AAGCTAGCAAGCGGCGCCTG	tag9482
novel_sir3782	AGTTCGATCGTTCGCCGCGG	AAGCTAGCAAGCGGCGCCTG	tag216681
novel_sir3783	GGTTCGATCGTTCGCCGCGG	AAGCTAGCAAGCGGCGCCTG	tag223965
novel_sir3784	AGGTTCGATCGTTCGCCGCGG	CAAGCTAGCAAGCGGCGCCTG	tag202125
novel_sir3785	TAGGTTCGATCGTTCGCCGCGG	CCAAGCTAGCAAGCGGCGCCGG	tag211559
novel_sir3786	TAGGTTCGATCGTTCGCCGCGG	CCAAGCTAGCAAGCGGCGCCTG	tag211559
novel_sir3787	CGGGGTGAGCGGCCGGCCGCGG	CCCACTCGCCGGCCGGCGCCTT	tag106185
novel_sir3788	CTCTATTAAAAGGGCGATCCGCGG	GATAATTTTCCCGCTAGGCGCCAC	tag239757
novel_sir3789	TGCCTCTCCCTTTTTCCGCGG	GGAGAGGGAAAAAGGCGCCGG	tag178192
novel_sir3790	GTGCCTCTCCCTTTTTCCGCGG	CGGAGAGGGAAAAAGGCGCCGG	tag34255
novel_sir3791	ACACGGTAGCTTCGCGCCAC	CGTGTGCCATCGAAGCGCGG	tag120108
novel_sir3792	CGGCCTTGGTCTCCGCGCCCG	CGGCCGGAACCAGAGGCGCGG	tag16133
novel_sir3793	GAGGCAGGGCCTGCGGCGCGG	CCGTCCCGGACGCCGCGCCCG	tag175196
novel_sir3794	CTATTCGAATAGCCGCGCCCT	TAGATAAGCTTATCGGCGCGG	tag23101
novel_sir3795	TCGGCCGGGCCGGCTGCGCGG	CCGGCCCGGCCGACGCGCCGC	tag145120
novel_sir3796	CGCCGGAGGCGCGGTGCGCGG	GGCCTCCGCGCCACGCGCCGC	tag13803
novel_sir3797	GGCCTCCACGCCACGCGCCGC	CGCCGGAGGTGCGGTGCGCGG	tag97495
novel_sir3798	AAGAGAGCACGAGGCATCGCGG	CTCTCGTGCTCCGTAGCGCCGC	tag54267
novel_sir3799	ATGTGGAGACATGAGCGCCAC	CGTACACCTCTGTACTCGCGG	tag108956
novel_sir3800	GCGGCGCGGCAGCTAAGGCGG	CCGCGCCGTCGATTCCGCCGT	tag123443
novel_sir3801	CGCGGCGCGGCAGCTAAGGCGG	GCCGCGCCGTCGATTCCGCCGT	tag8233
novel_sir3802	TGTTTTGTTTAATGTCTAAGGCGG	AAAACAAATTACAGATTCCGCCTA	tag67841
novel_sir3803	TGTTTTGTTTAATGTCTAAGGCGG	AAAACAAATTACAGATTCCGCCTT	tag67841
novel_sir3804	TGTTTTGTTTAATGTCTAAGGCGG	AAAACAAATTACAGATTCCGCCAG	tag67841
novel_sir3805	CGGCGGGTGGCTGGCAGGCGG	CGCCCACCGACCGTCCGCCCC	tag293016
novel_sir3806	CGGACCACCCTCTAGATCCGCCAC	TGGCCTGGTGGGAGATCTAGGCGG	tag292228
novel_sir3807	AGGTGAACCGATGTAGGCGG	CACTTGGCTACATCCGCCCC	tag133040

novel_sir3808	GGGTCAAGTTTAGACCCACGGCGG	CAGTTCAAATCTGGGTGCCGCCTA	tag186801
novel_sir3809	CAGTTCAAATCTGGGTGCCGCCTC	GGGTCAAGTTTAGACCCACGGCGG	tag186801
novel_sir3810	ACCGCTTGGCCATGCCGCCAC	AATGGCGAACCGGTACGGCGG	tag245875
novel_sir3811	ACCGCTTGGCCATGCCGCCAA	AATGGCGAACCGGTACGGCGG	tag245875
novel_sir3812	CGGAATGGCGAACCGGTACGGCGG	CTTACCGCTTGGCCATGCCGCCAA	tag277023
novel_sir3813	GGGGACGGAATGGCGAACCGGTACG[illegible]	CCTGCCTTACCGCTTGGCCATGCCGC[illegible]	tag62600
novel_sir3814	AGGGGACGGAATGGCGAACCGGTAC[illegible]	CCCTGCCTTACCGCTTGGCCATGCCG[illegible]	tag133028
novel_sir3815	CAGCTAAGGCGGCAGCGGCGG	CGATTCCGCCGTCGCCGCCGC	tag221921
novel_sir3816	GCGGCTGTCCTCGACGCGGCGG	CCGACAGGAGCTGCGCCGCCGC	tag267773
novel_sir3817	CTCGAGCGAACGAGGCGGCGG	GCTCGCTTGCTCCGCCGCCCA	tag259314
novel_sir3818	AAGGATTCAGCCCGCCGCCCG	GTTTCCTAAGTCGGGCGGCGG	tag193966
novel_sir3819	AAAGGATTCAGCCCGCCGCCCG	CGTTTCCTAAGTCGGGCGGCGG	tag39796
novel_sir3820	ATGGACACAGCCGAGCCGCCCG	CGTACCTGTGTCGGCTCGGCGG	tag202936
novel_sir3821	ATCTCACACTGACTCAGCCGCCAC	CCTAGAGTGTGACTGAGTCGGCGG	tag142555
novel_sir3822	GGCGGTGCGGCTCGTTCGGCGG	GCCACGCCGAGCAAGCCGCCGC	tag246889
novel_sir3823	CTCTAGCGCTGGGAAACAGGGCGG	GATCGCGACCCTTTGTCCCGCCAA	tag24994
novel_sir3824	TGGCGATGTTCAAATTCCTGGCGG	CGCTACAAGTTTAAGGACCGCCCG	tag74385
novel_sir3825	AGCTGAACATGTTCCTTCTGGCGG	GACTTGTACAAGGAAGACCGCCTT	tag251016
novel_sir3826	CTGTTTCTGAACCAGTTCTGGCGG	CAAAGACTTGGTCAAGACCGCCAT	tag204972
novel_sir3827	ACTGACCTGACGGTGCGCATGCGG	ACTGGACTGCCACGCGTACGCCAC	tag91046
novel_sir3828	GACGAAGCAGGTGGACGCCGA	GTCTGCTTCGTCCACCTGCGG	tag153428
novel_sir3829	CTGAAGATCGGACACTGCCTGCGG	CTTCTAGCCTGTGACGGACGCCAT	tag41724
novel_sir3830	GGACCGAGGAGCCGACGCCGA	CTCCTGGCTCCTCGGCTGCGG	tag217143
novel_sir3831	GATGATGCTTAGACCTGTCTGCGG	ACTACGAATCTGGACAGACGCCTC	tag61993
novel_sir3832	GTCCTAGTTTGACTGACCGTGCGG	GGATCAAACTGACTGGCACGCCCG	tag180116
novel_sir3833	GACGGTCGGCGGCCGGTGCGG	GCCAGCCGCCGGCCACGCCGG	tag54021
novel_sir3834	CCGTCCCGGACGCCACGCCCG	GAGGCAGGGCCTGCGGTGCGG	tag277095
novel_sir3835	GGCCTAATACTGACTTGCGG	GGATTATGACTGAACGCCTC	tag275776
novel_sir3836	CGGCCTAATACTGACTTGCGG	CGGATTATGACTGAACGCCTC	tag91479
novel_sir3837	ACGGCCTAATACTGACTTGCGG	CCGGATTATGACTGAACGCCTC	tag54076
novel_sir3838	ACCTTAACCTGGGTGGACAATCGG	GAATTGGACCCACCTGTTAGCCAC	tag66635
novel_sir3839	TGTCGAAGCCGTCTAGCGAATCGG	AGCTTCGGCAGATCGCTTAGCCCC	tag193100
novel_sir3840	AGGACCTTGTGTTGGAATCGG	CTGGAACACAACCTTAGCCGT	tag217562
novel_sir3841	GGTGGAAAATCGGAACTTAATCGG	ACCTTTTAGCCTTGAATTAGCCCC	tag3806

novel_sir3842	GGTGGAAAATCGGAACTTAATCGG	ACCTTTTAGCCTTGAATTAGCCCA	tag3806
novel_sir3843	ACCTTTTAGCCTTGAATTAGCCTA	GGTGGAAAATCGGAACTTAATCGG	tag3806
novel_sir3844	ACTTTATATCAAACTTGTAGCCCT	TTTGAAATATAGTTTGAACATCGG	tag82364
novel_sir3845	TGTCGACTGATATTTAGACATCGG	AGCTGACTATAAATCTGTAGCCCG	tag100351
novel_sir3846	TGTCGACTGATATTTAGACATCGG	AGCTGACTATAAATCTGTAGCCTG	tag100351
novel_sir3847	AGTCTGCGGGCTAGGCATCGG	AGACGCCCGATCCGTAGCCCC	tag293362
novel_sir3848	ACTAGTCTGCGGGCTAGGCATCGG	ATCAGACGCCCGATCCGTAGCCCC	tag16160
novel_sir3849	CCTAGTCTGCGGGCTAGGCATCGG	ATCAGACGCCCGATCCGTAGCCCC	tag94077
novel_sir3850	TCTAGTCTGCGGGCTAGGCATCGG	ATCAGACGCCCGATCCGTAGCCCC	tag178697
novel_sir3851	ACCTAGTCTGCGGGCTAGGCATCGG	GATCAGACGCCCGATCCGTAGCCCC	tag59077
novel_sir3852	ACCCATAATCTCCACAGTAGCCCC	CATGGGTATTAGAGGTGTCATCGG	tag128234
novel_sir3853	CTTCTACTCCAAACTGCTAGCCAA	TGGAAGATGAGGTTTGACGATCGG	tag263046
novel_sir3854	TCTCTTTTATATTACTACGATCGG	AGAAAATATAATGATGCTAGCCGC	tag241041
novel_sir3855	TTGGTATTCTGGAATACTGATCGG	CCATAAGACCTTATGACTAGCCAT	tag139950
novel_sir3856	AGCATTTAGCCGACTTATAGCCTA	AGTCGTAAATCGGCTGAATATCGG	tag85974
novel_sir3857	CATAGGTTGCTCATATATCGG	ATCCAACGAGTATATAGCCTT	tag113255
novel_sir3858	CGACGTCTTGTGGGGAGCCTT	TGGCTGCAGAACACCCCTCGG	tag160964
novel_sir3859	ATGGCTGCAGAACACCCCTCGG	CCGACGTCTTGTGGGGAGCCTT	tag258734
novel_sir3860	CATCAACACGAATTCTGGCCTCGG	AGTTGTGCTTAAGACCGGAGCCAA	tag70210
novel_sir3861	TTGGGACCTTTGCCAAGTCGG	CCCTGGAAACGGTTCAGCCGG	tag1353
novel_sir3862	GTTGGGACCTTTGCCAAGTCGG	ACCCTGGAAACGGTTCAGCCGG	tag192511
novel_sir3863	CGGCGGGTGGCTGGCAGTCGG	CGCCCACCGACCGTCAGCCCC	tag22075
novel_sir3864	TGTAGAACTAAGGTATAACGTCGG	ATCTTGATTCCATATTGCAGCCGG	tag74286
novel_sir3865	CTGCCATGGCCGCCGCAGCCAG	GAGACGGTACCGGCGGCGTCGG	tag69331
novel_sir3866	CGCTTTTCAAAGCTGGTTCGTCGG	GAAAAGTTTCGACCAAGCAGCCGG	tag196932
novel_sir3867	ACTTTTTGAATCTGAACCAGCCGT	GGTGAAAAACTTAGACTTGGTCGG	tag238133
novel_sir3868	ACTCTTTGAATCTGAACCAGCCAG	AGTGAGAAACTTAGACTTGGTCGG	tag241278
novel_sir3869	AGACGTCTCACAGTCAACAGCCAC	TATCTGCAGAGTGTCAGTTGTCGG	tag262032
novel_sir3870	TCCTGGTGGGGGTAGAAAATTCGG	GACCACCCCCATCTTTTAAGCCGT	tag207391
novel_sir3871	CGCTCTGCTTAGAAAATTCGG	GAGACGAATCTTTTAAGCCTA	tag15273
novel_sir3872	CGAGACGAATCTTTTAAGCCTA	GCGCTCTGCTTAGAAAATTCGG	tag137127
novel_sir3873	CCTGATACTGGACTCAAGCTTCGG	ACTATGACCTGAGTTCGAAGCCTC	tag265082
novel_sir3874	TGGGTGCAATCCCCGCGTTCGG	CCACGTTAGGGGCGCAAGCCCC	tag200887
novel_sir3875	GCTGGGTGCAATCCCCGCGTTCGG	ACCCACGTTAGGGGCGCAAGCCCC	tag90739

novel_sir3876	GCGGCGGTGCGGCTCGTTCGG	CCGCCACGCCGAGCAAGCCGC	tag276520
novel_sir3877	GGCGGCGGTGCGGCTCGTTCGG	GCCGCCACGCCGAGCAAGCCGC	tag112989
novel_sir3878	ATGTATCTACATAGTCCAAGCCTG	AATACATAGATGTATCAGGTTCGG	tag24120
novel_sir3879	CGGGTGCAGCCGGGTCAGGTTCGG	CCACGTCGGCCCAGTCCAAGCCGC	tag90589
novel_sir3880	GGGCACGGTGGCCGGTTCGG	CGTGCCACCGGCCAAGCCGG	tag173498
novel_sir3881	CGGGCACGGTGGCCGGTTCGG	CCGTGCCACCGGCCAAGCCGG	tag162156
novel_sir3882	CCGCTGATTCCGCCAAGCCCG	GGGGCGACTAAGGCGGTTCGG	tag202652
novel_sir3883	GAATCCGTTTAGATTGAAAGCCAA	CACTTAGGCAAATCTAACTTTCGG	tag153533
novel_sir3884	CATAGTAGGGCCAGGTTTCGG	ATCATCCCGGTCCAAAGCCAC	tag5384
novel_sir3885	TAGCGCGAACCAACTTTTCGG	CGCGCTTGGTTGAAAAGCCAG	tag104995
novel_sir3886	CCGGCCGGCGCCTTTTTCCCTC	GCGGCCGGCCGCGGAAAAAGGG	tag186637
novel_sir3887	GATACAAGCCGTGCGGACCAAGGG	ATGTTCGGCACGCCTGGTTCCCAA	tag130973
novel_sir3888	AGCTTACTGCCGACCGGTTCCCCC	CGTCGAATGACGGCTGGCCAAGGG	tag73648
novel_sir3889	CGCACTAGGCACCGTTCCCTC	CGGCGTGATCCGTGGCAAGGG	tag99039
novel_sir3890	AGGCGGCAGCGGCGGCGAAGGG	CGCCGTCGCCGCCGCTTCCCCT	tag142758
novel_sir3891	CGGCGGAGCTTCGAGGGAAGGG	CGCCTCGAAGCTCCCTTCCCAA	tag216211
novel_sir3892	AGCGGTATGTCATCTAGGTCCCTT	CATCGCCATACAGTAGATCCAGGG	tag21556
novel_sir3893	GTTGTAGGCTAGTCAAAAGCAGGG	ACATCCGATCAGTTTTCGTCCCTA	tag278067
novel_sir3894	TCGAGCGCAACTGATGCAGGG	CTCGCGTTGACTACGTCCCTG	tag114940
novel_sir3895	CCGACTCGGTAGAAATCAGGG	CTGAGCCATCTTTAGTCCCGG	tag23577
novel_sir3896	AGCCGGCTAAAGGTGTCATCAGGG	GGCCGATTTCCACAGTAGTCCCGG	tag198746
novel_sir3897	CAGAGGTATTCCGGCTAGTCCCAT	GGGTCTCCATAAGGCCGATCAGGG	tag195355
novel_sir3898	CGGCTAAAGGTGTTATCAGGG	CGATTTCCACAATAGTCCCGG	tag252761
novel_sir3899	CCGGCTAAAGGTGTTATCAGGG	CCGATTTCCACAATAGTCCCGG	tag229994
novel_sir3900	TGCCGGCTAAAGGTGTTATCAGGG	GGCCGATTTCCACAATAGTCCCGG	tag292167
novel_sir3901	AGCCGGCTTAAGGTGTTATCAGGG	GGCCGAATTCCACAATAGTCCCGG	tag65142
novel_sir3902	ACCGAGATTCCGGGCTCAGGG	GCTCTAAGGCCCGAGTCCCTC	tag56018
novel_sir3903	CCGCGATCTCGGTACCAGTCCCAG	TTGGCGCTAGAGCCATGGTCAGGG	tag75076
novel_sir3904	CCGCGTGCCGCTCCTCTCCCCG	CGGGCGCACGGCGAGGAGAGGG	tag260713
novel_sir3905	CGGCCGGCGGAGCTTCGAGGG	CGGCCGCCTCGAAGCTCCCTT	tag83006
novel_sir3906	CCGGCCGGCGGAGCTTCGAGGG	CCGGCCGCCTCGAAGCTCCCTT	tag118782
novel_sir3907	GAGCGCAGATAGTGAGGAGGG	CGCGTCTATCACTCCTCCCAT	tag151687
novel_sir3908	CTAGGCACCGTTCCCTCCCTC	GTGATCCGTGGCAAGGGAGGG	tag214331
novel_sir3909	GCACTAGGCACCGTTCCCTCCCTC	GGCGTGATCCGTGGCAAGGGAGGG	tag83374

novel_sir3910	CGCCACCGCCGGACCTCCCCG	CGGCGGTGGCGGCCTGGAGGG	tag48267
novel_sir3911	CTCCTTTCATAATACTCCCTA	CGGAGGAAAGTATTATGAGGG	tag17773
novel_sir3912	CTCCTTTCATAATACTCCCTA	AGGAGGAAAGTATTATGAGGG	tag189660
novel_sir3913	CTCCTTTCATAATACTCCCTA	TGGAGGAAAGTATTATGAGGG	tag239837
novel_sir3914	ATGGAGGAAAGTATTATGAGGG	CCTCCTTTCATAATACTCCCTA	tag121369
novel_sir3915	TTGGAGGAAAGTATTATGAGGG	CCTCCTTTCATAATACTCCCTA	tag184338
novel_sir3916	CTGGAGGAAAGTATTATGAGGG	CCTCCTTTCATAATACTCCCTA	tag278510
novel_sir3917	AACCTCCTTTCATAATACTCCCTA	TATTGGAGGAAAGTATTATGAGGG	tag102317
novel_sir3918	AACCTCCTTTCATAATACTCCCTA	AATTGGAGGAAAGTATTATGAGGG	tag198244
novel_sir3919	AACCTCCTTTCATAATACTCCCTA	CATTGGAGGAAAGTATTATGAGGG	tag281861
novel_sir3920	AACCTCCTTTCATAATACTCCCCA	TATTGGAGGAAAGTATTATGAGGG	tag102317
novel_sir3921	AACCTCCTTTCATAATACTCCCCA	AATTGGAGGAAAGTATTATGAGGG	tag198244
novel_sir3922	AACCTCCTTTCATAATACTCCCCA	CATTGGAGGAAAGTATTATGAGGG	tag281861
novel_sir3923	ACTTGGAGGAAAGTATTATGAGGG	AACCTCCTTTCATAATACTCCCAA	tag96935
novel_sir3924	TATTGGAGGAAAGTATTATGAGGG	AACCTCCTTTCATAATACTCCCAA	tag102317
novel_sir3925	AATTGGAGGAAAGTATTATGAGGG	AACCTCCTTTCATAATACTCCCAA	tag198244
novel_sir3926	CATTGGAGGAAAGTATTATGAGGG	AACCTCCTTTCATAATACTCCCAA	tag281861
novel_sir3927	ACGAAATCTTAGGTGGTATCCCGA	GATGCTTTAGAATCCACCATAGGG	tag151163
novel_sir3928	CGCGCGCACACCGAAAGGCTAGGG	GCGCGTGTGGCTTTCCGATCCCCA	tag230463
novel_sir3929	GAAAAGCTAGCCGGAAGATCCCAG	TACTTTTCGATCGGCCTTCTAGGG	tag250711
novel_sir3930	CCACAAAGTTTTCGCATCCCTT	AGGGTGTTTCAAAAGCGTAGGG	tag212463
novel_sir3931	GCGCAGTTGGCCAGTGTAGGG	CGTCAACCGGTCACATCCCTA	tag213972
novel_sir3932	CGTTGAATCCGCCACATCCCGA	AGGCAACTTAGGCGGTGTAGGG	tag109704
novel_sir3933	GGCTAAAGAAATAGTAGGAATCCCCC	AACCGATTTCTTTATCATCCTTAGGG	tag36235
novel_sir3934	GGCTAAAGAAATAGTAGGAATCCCCA	AACCGATTTCTTTATCATCCTTAGGG	tag36235
novel_sir3935	CGGCTAAAGAAATAGTAGGAATCCCC	AAGCCGATTTCTTTATCATCCTTAGGG	tag110920
novel_sir3936	CGGCTAAAGAAATAGTAGGAATCCCC	AAGCCGATTTCTTTATCATCCTTAGGG	tag110920
novel_sir3937	GCGGGGCCGCGGCCGAACGGG	CCCCGGCGCCGGCTTGCCCAG	tag120436
novel_sir3938	CGGTCGGTGTGACCGCGGCACGGG	CAGCCACACTGGCGCCGTGCCCGT	tag70734
novel_sir3939	TTGATAAGCTTATCGGCACGGG	CTATTCGAATAGCCGTGCCCGC	tag126389
novel_sir3940	CTCTGCCACTTACAATGCCCCG	GTGAGACGGTGAATGTTACGGG	tag152009
novel_sir3941	ACTCTGCCACTTACAATGCCCCG	GGTGAGACGGTGAATGTTACGGG	tag115773
novel_sir3942	ATGAAACATGACAATTACACCGGG	CTTTGTACTGTTAATGTGGCCCAA	tag121093
novel_sir3943	ATTAGCGATCTATTCTGCACCGGG	ATCGCTAGATAAGACGTGGCCCAC	tag263241

novel_sir3944	TATCGTTCAGGCGAGACCGGG	AGCAAGTCCGCTCTGGCCCTG	tag159466
novel_sir3945	GATAGCAAGTCCGCTCTGGCCCTG	ACCTATCGTTCAGGCGAGACCGGG	tag171837
novel_sir3946	CCGCCTCTGCCGTCGACCGGG	CGGAGACGGCAGCTGGCCCCC	tag150066
novel_sir3947	AAGAGGTTTGGGTAGCCAGCGGG	CTCCAAACCCATCGGTCGCCCGA	tag208617
novel_sir3948	CGCCCGCACGACGTCGCCCTC	GAGCGGGCGTGCTGCAGCGGG	tag252240
novel_sir3949	CGGGCGGCAAGGACGAGCGGG	CCGCCGTTCCTGCTCGCCCCG	tag96754
novel_sir3950	CCGGGCTTGGACAGATGTAGCGGG	CCCGAACCTGTCTACATCGCCCGT	tag2049
novel_sir3951	CGGGCTTGGACATATGTAGCGGG	CCGAACCTGTATACATCGCCCGT	tag64364
novel_sir3952	CCGGTCGACGGCAGAGGCGGG	CCAGCTGCCGTCTCCGCCCCA	tag60058
novel_sir3953	AGGATTCAGCCCGCCGCCCGT	TTTCCTAAGTCGGGCGGCGGG	tag239346
novel_sir3954	AAGGATTCAGCCCGCCGCCCGT	GTTTCCTAAGTCGGGCGGCGGG	tag234242
novel_sir3955	AAAGGATTCAGCCCGCCGCCCGT	CGTTTCCTAAGTCGGGCGGCGGG	tag34826
novel_sir3956	TGTGGATGCCTAGTCTGCGGG	ACCTACGGATCAGACGCCCGA	tag96349
novel_sir3957	ATGTGGATGCCTAGTCTGCGGG	CACCTACGGATCAGACGCCCGA	tag177393
novel_sir3958	TAGTCTGCGGGCTAGGCATCGGG	CAGACGCCCGATCCGTAGCCCCA	tag264770
novel_sir3959	ACTAGTCTGCGGGCTAGGCATCGGG	ATCAGACGCCCGATCCGTAGCCCCA	tag221951
novel_sir3960	CCTAGTCTGCGGGCTAGGCATCGGG	ATCAGACGCCCGATCCGTAGCCCCA	tag267830
novel_sir3961	ACTAGTCTGCGGGCTAGGCATCGGG	ATCAGACGCCCGATCCGTAGCCCCC	tag221951
novel_sir3962	CCTAGTCTGCGGGCTAGGCATCGGG	ATCAGACGCCCGATCCGTAGCCCCC	tag267830
novel_sir3963	AATACCACTTTCAACTGAGCCCAA	GTTTATGGTGAAAGTTGACTCGGG	tag5078
novel_sir3964	GCCGTAAAGACTATGAGAGCCCCC	GACGGCATTTCTGATACTCTCGGG	tag222407
novel_sir3965	GGCGGGTGGCTGGCAGTCGGG	GCCCACCGACCGTCAGCCCCC	tag197232
novel_sir3966	GTGGGCGCGGCAGCCGTCGGG	CCCGCGCCGTCGGCAGCCCGG	tag218256
novel_sir3967	CCCACGCCGTCGGCAGCCCGG	GTGGGTGCGGCAGCCGTCGGG	tag52691
novel_sir3968	GCTAGGTGACTCTAGGTCGGG	ATCCACTGAGATCCAGCCCCG	tag284147
novel_sir3969	AGATGACCCGTCACTGAAGCCCGG	TGTCTACTGGGCAGTGACTTCGGG	tag66024
novel_sir3970	ACTGAAATCCTATAGAAAGCCCGC	AGTGACTTTAGGATATCTTTCGGG	tag155933
novel_sir3971	GATCTGCTGGACCTAAGTTTCGGG	AGACGACCTGGATTCAAAGCCCAC	tag210227
novel_sir3972	GGCGGCAGCGGCGGCGAAGGGG	GCCGTCGCCGCCGCTTCCCCTC	tag64679
novel_sir3973	GGGCGCGCGGCGGGGAAGGGG	CGCGCGCCGCCCCTTCCCCCG	tag240429
novel_sir3974	CCGCTGGCGCCGACGCAGGGG	CGACCGCGGCTGCGTCCCCCC	tag163488
novel_sir3975	AACGATTAAGGCTAGAAGGAGGGG	GCTAATTCCGATCTTCCTCCCCCA	tag262509
novel_sir3976	GCTAATTCCGATCTTCCTCCCCAA	AACGATTAAGGCTAGAAGGAGGGG	tag262509
novel_sir3977	CCGATTTCGCCATATCCCCAT	AAGGCTAAAGCGGTATAGGGG	tag55963

novel_sir3978	GATGGTAGGCGTCGGTAGGGG	ACCATCCGCAGCCATCCCCGC	tag291357
novel_sir3979	GGCTAAAGAAATAGTAGGAATCCCCA	AACCGATTTCTTTATCATCCTTAGGGG	tag58030
novel_sir3980	AACCGATTTCTTTATCATCCTTAGGGG	GGCTAAAGAAATAGTAGGAATCCCCA	tag58030
novel_sir3981	CAGCTACGACTCGTGTTGCCCCAC	GAGTCGATGCTGAGCACAACGGGG	tag151541
novel_sir3982	CTGCCACTTACAATGCCCCGT	GAGACGGTGAATGTTACGGGG	tag204494
novel_sir3983	CCGCCTCTGCCGTCGACCGGGG	CGGAGACGGCAGCTGGCCCCCG	tag268783
novel_sir3984	CGGGCCTAGTGGTCGATTCCGGGG	CCGGATCACCAGCTAAGGCCCCTA	tag290702
novel_sir3985	GAGTCGATGCTGAGCACAGCGGGG	CAGCTACGACTCGTGTCGCCCCAC	tag88498
novel_sir3986	AGGTCGATACTAGGCACAGCGGGG	CAGCTATGATCCGTGTCGCCCCAC	tag95259
novel_sir3987	CGGTCGACGGCAGAGGCGGGG	CAGCTGCCGTCTCCGCCCCAC	tag89767
novel_sir3988	AGTCTGCGGGCTAGGCATCGGGG	AGACGCCCGATCCGTAGCCCCAT	tag148418
novel_sir3989	TAGTCTGCGGGCTAGGCATCGGGG	CAGACGCCCGATCCGTAGCCCCAT	tag166742
novel_sir3990	GCTAGGTGACTCTAGGTCGGGG	ATCCACTGAGATCCAGCCCCGC	tag63531
novel_sir3991	ACTAGATAAGTTACAGTCCCCCCT	TTTGATCTATTCAATGTCAGGGGG	tag118461
novel_sir3992	TGCTAGATCAACACAGTACGGGGG	GATCTAGTTGTGTCATGCCCCCTA	tag56385
novel_sir3993	TGCTAGTCCAACAGAGTACGGGGG	GATCAGGTTGTCTCATGCCCCCTA	tag118421
novel_sir3994	TGCTAGTTCAACAGAGTACGGGGG	GATCAAGTTGTCTCATGCCCCCTA	tag23591
novel_sir3995	GCTGGCGCCGACGCAGGGGGG	ACCGCGGCTGCGTCCCCCCTG	tag22071
novel_sir3996	GCGCGTGTGGCATTCCGACCCCCA	CGCGCGCACACCGTAAGGCTGGGG	tag264847
novel_sir3997	ACTTGTTCAGTTGTGTCACCCCGG	GTTGAACAAGTCAACACAGTGGGG	tag100546
novel_sir3998	AACGGCTTCGTTACAGTGGGG	GCCGAAGCAATGTCACCCCCA	tag195352
novel_sir3999	GGCGAAGAGTCAGACCGTGGGG	GCTTCTCAGTCTGGCACCCCGC	tag64488
novel_sir4000	ACAAAACCGATTCCATAACCCCTA	GCTGTTTTGGCTAAGGTATTGGGG	tag94449
novel_sir4001	CCACCTACGGCCGGAACCCCCG	GGGGTGGATGCCGGCCTTGGGG	tag133773
novel_sir4002	CTTGGACACTGTAAAACATGGG	ACCTGTGACATTTTGTACCCAA	tag134863
novel_sir4003	AGTCGATCGCGGGTGACTTATGGG	AGCTAGCGCCCACTGAATACCCAT	tag203286
novel_sir4004	GGTGGCGCCGGAGAACCTGGG	ACCGCGGCCTCTTGGACCCTC	tag86555
novel_sir4005	CCTGAATAGGCTATGTGGACCCAT	AGGGACTTATCCGATACACCTGGG	tag3461
novel_sir4006	AGCATCCGCCGCTTCGACCCAG	CGTCGTAGGCGGCGAAGCTGGG	tag254064
novel_sir4007	TTTGGTAGAAACCAGCCAGCTGGG	ACCATCTTTGGTCGGTCGACCCAA	tag268251
novel_sir4008	CGGGCGGCGGGCGGGGCTGGG	CCGCCGCCCGCCCCGACCCAC	tag202161
novel_sir4009	AGAATTTCCCGGTCGGTCACCCAT	CTTCTTAAAGGGCCAGCCAGTGGG	tag94518
novel_sir4010	CCACCTACGGCCGGAACCCCC	GGGGTGGATGCCGGCCTTGGG	tag100769
novel_sir4011	TGGGGTGGATGCCGGCCTTGGG	CCCACCTACGGCCGGAACCCCC	tag265613

novel_sir4012	CCTCAACACCTCGACGGATTTGGG	AGTTGTGGAGCTGCCTAAACCCAG	tag55649
novel_sir4013	TGGCCAGTGTAGGGATTTGGG	CGGTCACATCCCTAAACCCTT	tag262923
novel_sir4014	ATTTGGGATTTGGGATTTGGG	AACCCTAAACCCTAAACCCTA	tag177138
novel_sir4015	CCTACTGCAGCAACTGAAAAATGG	ATGACGTCGTTGACTTTTTACCAA	tag112736
novel_sir4016	CGTGGGCAACGGCTTTACCTT	TCGCACCCGTTGCCGAAATGG	tag222392
novel_sir4017	TAGATAAGAGTCGTAGTACAATGG	CTATTCTCAGCATCATGTTACCAT	tag156263
novel_sir4018	CCCGTTCGGCTCGTTACATGG	GCAAGCCGAGCAATGTACCCA	tag111875
novel_sir4019	ACCCGTTCGGCTCGTTACATGG	GGCAAGCCGAGCAATGTACCCA	tag233795
novel_sir4020	TTCTAAGCACGACGTTTTACATGG	GATTCGTGCTGCAAAATGTACCTA	tag192932
novel_sir4021	ATTTCCGATTATAAGCAGCCATGG	AAGGCTAATATTCGTCGGTACCGC	tag31516
novel_sir4022	CGCTCGTCGATGCCATGG	GAGCAGCTACGGTACCGT	tag239955
novel_sir4023	TGTTGAAAAGTCAGTGCAGCATGG	AACTTTTCAGTCACGTCGTACCAA	tag114441
novel_sir4024	AACTTTTCAGTCACATCGTACCAA	TGTTGAAAAGTCAGTGTAGCATGG	tag81264
novel_sir4025	TGTTGAAAAGATAGTGTAGCATGG	AACTTTTCTATCACATCGTACCAA	tag135663
novel_sir4026	TATTGAAAAGATAGTGTAGCATGG	AACTTTTCTATCACATCGTACCAA	tag259528
novel_sir4027	GTTGGGGAAACTGAAACAAGATGG	ACCCCTTTGACTTTGTTCTACCTC	tag175070
novel_sir4028	GAGACCCGCGTTCTACCGA	ACCTCTGGGCGCAAGATGG	tag33256
novel_sir4029	ATTGTCGGCTGGCGAGATGG	ACAGCCGACCGCTCTACCAC	tag185671
novel_sir4030	AATTGTCGGCTGGCGAGATGG	AACAGCCGACCGCTCTACCAA	tag264343
novel_sir4031	AACAGCCGACCGCTCTACCAC	AATTGTCGGCTGGCGAGATGG	tag264343
novel_sir4032	GAGGTGCACGGCCGACGATGG	CCACGTGCCGGCTGCTACCGC	tag173269
novel_sir4033	CCACGCGCCGGCTGCTACCGC	GAGGTGCGCGGCCGACGATGG	tag125887
novel_sir4034	AGCTGTTGTCAATCTATCCGATGG	GACAACAGTTAGATAGGCTACCCC	tag68164
novel_sir4035	TTCGGTCACCGCGCTTCGATGG	GCCAGTGGCGCGAAGCTACCGT	tag42657
novel_sir4036	GGCCTTGGGAATCATAAATTATGG	GGAACCCTTAGTATTTAATACCGG	tag216453
novel_sir4037	GGTGCATTCGCGGTGCAGTTATGG	ACGTAAGCGCCACGTCAATACCAC	tag244358
novel_sir4038	ACTGAAAAATCGTACACAAACTGG	ACTTTTTAGCATGTGTTTGACCGT	tag67142
novel_sir4039	ACTGAAAAATCATGCACAAACTGG	ACTTTTTAGTACGTGTTTGACCAT	tag138326
novel_sir4040	ACTTTTTAGTACGTGTTTGACCAA	ACTGAAAAATCATGCACAAACTGG	tag138326
novel_sir4041	ACTGAAAAATCGTGCACAAACTGG	ACTTTTTAGCACGTGTTTGACCAT	tag17475
novel_sir4042	GAACCATGTCCAGTGTTTGACCGT	CCCTTGGTACAGGTCACAAACTGG	tag101893
novel_sir4043	CTTTTTAGCATATGTTTGACCGC	CTGAAAAATCGTATACAAACTGG	tag266705
novel_sir4044	CTGAAAAATCGTATACAAACTGG	CTTTTTAGCATATGTTTGACCGT	tag266705
novel_sir4045	ACTGAAAAATCGTATACAAACTGG	ACTTTTTAGCATATGTTTGACCGC	tag110162

novel_sir4046	ACTGAAAAATCGTATACAAACTGG	ACTTTTTAGCATATGTTTGACCGT	tag110162
novel_sir4047	ACTGAAAAAATTTATACAAACTGG	ACTTTTTTAAATATGTTTGACCGT	tag69893
novel_sir4048	ACTGAAAAAGAATGTACAAACTGG	ACTTTTTCTTACATGTTTGACCAT	tag12730
novel_sir4049	ACTTTTTCTCACATGTTTGACCAA	ACTGAAAAAGAGTGTACAAACTGG	tag173348
novel_sir4050	ACTGAAAAAGAGTGTACAAACTGG	ACTTTTTCTCACATGTTTGACCAT	tag173348
novel_sir4051	ACTTTTTATCACATGTTTGACCGT	ACTGAAAAATAGTGTACAAACTGG	tag154379
novel_sir4052	ACTGAAAAATAGTGTACAAACTGG	ACTTTTTATCACATGTTTGACCGC	tag154379
novel_sir4053	CTTTTTAGCACATGTTTGACCGT	CTGAAAAATCGTGTACAAACTGG	tag265862
novel_sir4054	ACTTTTTAGCACATGTTTGACCGT	ACTGAAAAATCGTGTACAAACTGG	tag184885
novel_sir4055	ACTTTTTAGCACATGTTTGACCAA	ACTGAAAAATCGTGTACAAACTGG	tag184885
novel_sir4056	ACTGAAAAATCGTGTACAAACTGG	ACTTTTTAGCACATGTTTGACCGA	tag184885
novel_sir4057	ACTGAAAAATCGTGTACAAACTGG	ACTTTTTAGCACATGTTTGACCAT	tag184885
novel_sir4058	ACTTTTTAGCACATGTTTGACCGC	ACTGAAAAATCGTGTACAAACTGG	tag184885
novel_sir4059	ACTGAAAAATTGTGTACAAACTGG	ACTTTTTAACACATGTTTGACCGT	tag78427
novel_sir4060	ACTGAAAAAATTTGTACAAACTGG	ACTTTTTTAAACATGTTTGACCGT	tag131134
novel_sir4061	ACTGAAAAAATTTGTACAAACTGG	ACTTTTTTAAACATGTTTGACCAT	tag131134
novel_sir4062	ACTTTTTAAAACATGTTTGACCGT	ACTGAAAAATTTTGTACAAACTGG	tag222832
novel_sir4063	ACCTTTTGACCAATGTTTGACCAT	ACTGGAAAACTGGTTACAAACTGG	tag233996
novel_sir4064	ACTTTTTTAAAAATGTTTGACCGT	ACTGAAAAAATTTTTACAAACTGG	tag36012
novel_sir4065	ACTTTTCGATCAGCGTTTGACCAT	ACTGAAAAGCTAGTCGCAAACTGG	tag266832
novel_sir4066	ACTGAAAAGCTGGATGCAAACTGG	ACTTTTCGACCTACGTTTGACCAT	tag251064
novel_sir4067	CCCTTGACACAGGCTGCAAACTGG	GAACTGTGTCCGACGTTTGACCAT	tag191698
novel_sir4068	ACCCCGTGTCCGACGTTTGACCGT	CTTGGGGCACAGGCTGCAAACTGG	tag22899
novel_sir4069	ATTGAAAAATAATGTGCAAACTGG	ACTTTTTATTACACGTTTGACCAT	tag186798
novel_sir4070	ACTTTTTATCACACGTTTGACCAT	ACTGAAAAATAGTGTGCAAACTGG	tag283488
novel_sir4071	ACTGAAAAACAGATTGCAAACTGG	ACTTTTTGTCTAACGTTTGACCAT	tag231133
novel_sir4072	TCTTAGGCACAGATTGCAAACTGG	AATCCGTGTCTAACGTTTGACCAT	tag81341
novel_sir4073	ACTTTTCGTCTAACGTTTGACCAT	ATTGAAAAGCAGATTGCAAACTGG	tag52319
novel_sir4074	ACTTTTTATCTAACGTTTGACCAT	ACTGAAAAATAGATTGCAAACTGG	tag256478
novel_sir4075	ACTGAAAAGCTGATTGCAAACTGG	ACTTTTCGACTAACGTTTGACCAT	tag115629
novel_sir4076	ACTGAAAAACAAGTTGCAAACTGG	ACTTTTTGTTCAACGTTTGACCAT	tag56610
novel_sir4077	ACTTTTTATTCAACGTTTGACCAT	ATTGAAAAATAAGTTGCAAACTGG	tag119715
novel_sir4078	ACTTTTTGCTCAACGTTTGACCAT	ACTGAAAAACGAGTTGCAAACTGG	tag238923
novel_sir4079	ACTGAAAAGCTAGTTGCAAACTGG	ACTTTTCGATCAACGTTTGACCAT	tag11370

novel_sir4080	ATTTTTGTCCAACGTTTGACCAT	ACTAAAAAACAGGTTGCAAACTGG	tag281963
novel_sir4081	ACTAAAAAACAGGTTGCAAACTGG	ATTTTTGTCCAACGTTTGACCGT	tag281963
novel_sir4082	ACTGAAAAACAGGTTGCAAACTGG	ACTTTTGTCCAACGTTTGACCCT	tag213449
novel_sir4083	ATTGAAAAACAGGTTGCAAACTGG	ACTTTTGTCCAACGTTTGACCCT	tag213959
novel_sir4084	ACTGAAAAACAGGTTGCAAACTGG	ACTTTTTGTCCAACGTTTGACCAT	tag213449
novel_sir4085	ATTGAAAAACAGGTTGCAAACTGG	ACTTTTTGTCCAACGTTTGACCAT	tag213959
novel_sir4086	AATCCGTGTCCAACGTTTGACCGT	CTTTAGGCACAGGTTGCAAACTGG	tag222198
novel_sir4087	CCTTAGGCACAGGTTGCAAACTGG	AATCCGTGTCCAACGTTTGACCAT	tag44953
novel_sir4088	CTTTAGGCACAGGTTGCAAACTGG	AATCCGTGTCCAACGTTTGACCAT	tag222198
novel_sir4089	ATTGAAAAATAGGTTGCAAACTGG	ACTTTTTATCCAACGTTTGACCAT	tag116124
novel_sir4090	ACTGAAAAATAGGTTGCAAACTGG	ACTTTTTATCCAACGTTTGACCAT	tag224480
novel_sir4091	CCTTAGGCATAGGTTGCAAACTGG	AATCCGTATCCAACGTTTGACCGT	tag121779
novel_sir4092	ACTGAAAAACTGGTTGCAAACTGG	ACTTTTTGACCAACGTTTGACCAT	tag166447
novel_sir4093	ACTTTTTAACCAACGTTTGACCAC	ACTGAAAAATTGGTTGCAAACTGG	tag99260
novel_sir4094	ACTGAAAAATTGGTTGCAAACTGG	ACTTTTTAACCAACGTTTGACCAT	tag99260
novel_sir4095	ACTTTATAACCAACGTTTGACCAC	ACTGAAATATTGGTTGCAAACTGG	tag196081
novel_sir4096	TATAGGCATAAATCGCGAAACTGG	ATCCGTATTTAGCGCTTTGACCAT	tag264245
novel_sir4097	ACTGAAAAAGAGTGTATAAACTGG	ACTTTTTCTCACATATTTGACCAT	tag217838
novel_sir4098	CTTTTTAAGCACATATTTGACCGT	CTGAAAAATTCGTGTATAAACTGG	tag235278
novel_sir4099	AAAATATTTGGCGCCGTTGACCTT	GGTTTTATAAACCGCGGCAACTGG	tag185765
novel_sir4100	ACTTTTTATCACACATATGACCAT	ACTGAAAAATAGTGTGTATACTGG	tag77338
novel_sir4101	TGGTGACTTTCTGGAGCCTACTGG	CACTGAAAGACCTCGGATGACCGG	tag169656
novel_sir4102	ACTGAAAAAATTTGTGCAACCTGG	ACTTTTTTAAACACGTTGGACCGT	tag71208
novel_sir4103	GCGCTTTGCAAGTTCGTGGACCTA	CACGCGAAACGTTCAAGCACCTGG	tag18061
novel_sir4104	ACGCTTTGCAAGATCGTGGACCTA	CTTGCGAAACGTTCTAGCACCTGG	tag103686
novel_sir4105	GTGCAGCGGCCGGCGGCCTGG	CGTCGCCGGCCGCCGGACCTC	tag219892
novel_sir4106	GGGGTGTACAGTTGGATTTCCTGG	CCACATGTCAACCTAAAGGACCGC	tag215078
novel_sir4107	GGAGCCTACGCGTCGGGCGACCGA	CTCCTCGGATGCGCAGCCCGCTGG	tag238167
novel_sir4108	AGGAGGAACTTGCGGCGCTGG	CTCCTTGAACGCCGCGACCTC	tag152959
novel_sir4109	TCTGCATTTTCAGCCTGATGCTGG	ACGTAAAAGTCGGACTACGACCGT	tag127380
novel_sir4110	CCGGATAGCTAGGAAATCTGG	CCTATCGATCCTTTAGACCTT	tag71625
novel_sir4111	TCTCGCTCGGGGGTCGCAATCTGG	AGCGAGCCCCCAGCGTTAGACCGT	tag176304
novel_sir4112	CATAAAGGAATCTGAATCTGG	ATTTCCTTAGACTTAGACCAC	tag89133
novel_sir4113	CCTGGCAGTCAGGGCGCCATCTGG	ACCGTCAGTCCCGCGGTAGACCGC	tag177310

novel_sir4114	AGCGTTTCGCTCCACGTAGACCAG	GTTCGCAAAGCGAGGTGCATCTGG	tag179651
novel_sir4115	ACAGCCAAAACTAGAGTAGACCGT	TGTGTCGGTTTTGATCTCATCTGG	tag217714
novel_sir4116	ACGAATTGCTAAACGGTGATCTGG	CTTAACGATTTGCCACTAGACCCA	tag94410
novel_sir4117	CACGCGTACGCTATATAGACCAA	CGGTGCGCATGCGATATATCTGG	tag238965
novel_sir4118	CTGGCTTGACTTTTCTGACTCTGG	CCGAACTGAAAAGACTGAGACCGA	tag181438
novel_sir4119	ATGCCACGTGGGACGGAGACCTA	GTTACGGTGCACCCTGCCTCTGG	tag287261
novel_sir4120	AGTTACGGTGCACCCTGCCTCTGG	AATGCCACGTGGGACGGAGACCTA	tag193998
novel_sir4121	AACGACACGTGTAAAGGAGACCAG	AGTTGCTGTGCACATTTCCTCTGG	tag50807
novel_sir4122	CGAAAAGACCGAGACCGAGACCGA	TGGCTTTTCTGGCTCTGGCTCTGG	tag216202
novel_sir4123	TGGCTTTCTGGCTTCTGGCTCTGG	CGAAAGACCGAAGACCGAGACCGA	tag143161
novel_sir4124	GGCGTGAAGACTATGAGAGACCCC	GACCGCACTTCTGATACTCTCTGG	tag13247
novel_sir4125	AATGCCACGTGGGATGAAGACCTT	AGTTACGGTGCACCCTACTTCTGG	tag67872
novel_sir4126	AGTTACGGTGCACCCTACTTCTGG	AATGCCACGTGGGATGAAGACCTA	tag67872
novel_sir4127	AATGCTACGTCGGATGAAGACCGA	AGTTACGATGCAGCCTACTTCTGG	tag9038
novel_sir4128	AGTCACGGTGCAGCCTACTTCTGG	AGTGCCACGTCGGATGAAGACCGA	tag279856
novel_sir4129	AGTTACGATGCAGTCTACTTCTGG	AATGCTACGTCAGATGAAGACCGA	tag190619
novel_sir4130	AGTAACGGTGCAGTCTACTTCTGG	ATTGCCACGTCAGATGAAGACCGA	tag83503
novel_sir4131	AGTCACGGTGCAGTCTACTTCTGG	AGTGCCACGTCAGATGAAGACCGA	tag216901
novel_sir4132	ACTGCCACGTCAGATGAAGACCGA	AGTGACGGTGCAGTCTACTTCTGG	tag56918
novel_sir4133	ATGCCACGTCAGATGAAGACCGA	GTTACGGTGCAGTCTACTTCTGG	tag123407
novel_sir4134	AATGCCACGTCAGATGAAGACCGG	AGTTACGGTGCAGTCTACTTCTGG	tag47932
novel_sir4135	AGTTACGGTGCAGTCTACTTCTGG	AATGCCACGTCAGATGAAGACCGA	tag47932
novel_sir4136	AGTTACGGTGTAGTCTACTTCTGG	AATGCCACATCAGATGAAGACCAA	tag137103
novel_sir4137	AATGCCACGTGGGACGAAGACCTA	AGTTACGGTGCACCCTGCTTCTGG	tag225988
novel_sir4138	AATGCCACGTGGGACGAAGACCGA	AGTTACGGTGCACCCTGCTTCTGG	tag225988
novel_sir4139	AGCTGCTGTGCACCCTGCTTCTGG	GACGACACGTGGGACGAAGACCGG	tag245581
novel_sir4140	AGTTACGGTGCACTCTGCTTCTGG	AATGCCACGTGAGACGAAGACCTA	tag279022
novel_sir4141	GGCTTCTTGGCTTGGCTTTTCTGG	GAAGAACCGAACCGAAAAGACCGA	tag108866
novel_sir4142	GACTTGGGTTCGAAGCTTCACCCC	TGCTGAACCCAAGCTTCGAAGTGG	tag52180
novel_sir4143	ATCTTGATCAACCTTCACCTC	ACTAGAACTAGTTGGAAGTGG	tag217465
novel_sir4144	CTGCATGCTGCTGAAGTGG	CGTACGACGACTTCACCAT	tag48197
novel_sir4145	GCGATCCGAACACTTCACCGG	GGCGCTAGGCTTGTGAAGTGG	tag70145
novel_sir4146	CGCGATCCGAACACTTCACCGG	CGGCGCTAGGCTTGTGAAGTGG	tag249028
novel_sir4147	GTGGAGCAGTGCGAGCAGTGG	CCTCGTCACGCTCGTCACCGG	tag182552

novel_sir4148	CTGGAGGGGATGTCATAGCAGTGG	CCTCCCCTACAGTATCGTCACCGC	tag44112
novel_sir4149	GGGAGGGGTGCGCGGCAGTGG	CTCCCCACGCGCCGTCACCTC	tag226983
novel_sir4150	GATCTGGGTTCGAAGTCTCACCCC	TGCTAGACCCAAGCTTCAGAGTGG	tag131918
novel_sir4151	TGCTTGACCCAAGCTTCAGAGTGG	GAACTGGGTTCGAAGTCTCACCCC	tag42393
novel_sir4152	GACCTGGATTCGAAGTCTCACCCC	TGCTGGACCTAAGCTTCAGAGTGG	tag1488
novel_sir4153	GGGAGAGGCACCCGCGAGTGG	CTCTCCGTGGGCGCTCACCGG	tag248458
novel_sir4154	GACCTGGGTTCGAAGCCTCACCCC	TGCTGGACCCAAGCTTCGGAGTGG	tag284019
novel_sir4155	TGCTGGACCCAAGCTTCGGAGTGG	GACCTGGGTTCGAAGCCTCACCCA	tag284019
novel_sir4156	TGCTGGATCCAAGCTTCGGAGTGG	GACCTAGGTTCGAAGCCTCACCCC	tag32173
novel_sir4157	TGCTGAACCCAAATTTCGGAGTGG	GACTTGGGTTTAAAGCCTCACCAC	tag225825
novel_sir4158	TGCTGGACCCAAGTTTCGGAGTGG	GACCTGGGTTCAAAGCCTCACCCC	tag45899
novel_sir4159	TACTGGACCCAAGTTTCGGAGTGG	GACCTGGGTTCAAAGCCTCACCCC	tag58136
novel_sir4160	GATCTGGGTTCGAAGTATCACCCC	TGCTAGACCCAAGCTTCATAGTGG	tag118981
novel_sir4161	CGACTTAGTGATCACCGG	CTGCTGAATCACTAGTGG	tag120657
novel_sir4162	GTTCGGCTGAGGGCCAGGTAGTGG	AGCCGACTCCCGGTCCATCACCGC	tag226573
novel_sir4163	CTCCCCGCGCGCCATCACCGC	GGGAGGGGCGCGCGGTAGTGG	tag213288
novel_sir4164	ATCTTAAGACCTCGTTTGCACCGG	GGTAGAATTCTGGAGCAAACGTGG	tag7747
novel_sir4165	GATCCTCTATAGTCAATGCACCGT	TCCTAGGAGATATCAGTTACGTGG	tag61618
novel_sir4166	ATCTAGCTAGCTGTTTGGCACCTA	TGTAGATCGATCGACAAACCGTGG	tag95235
novel_sir4167	ACGGGCAGAGTCGCGAGGTGG	CCCGTCTCAGCGCTCCACCGG	tag120123
novel_sir4168	GGGTTCCCGGGCACGGTGG	CAAGGGCCCGTGCCACCGG	tag128822
novel_sir4169	GGGGTTCCCGGGCACGGTGG	CCAAGGGCCCGTGCCACCGG	tag137249
novel_sir4170	GGGGGTTCCCGGGCACGGTGG	CCCAAGGGCCCGTGCCACCGG	tag222027
novel_sir4171	CGGGGGTTCCCGGGCACGGTGG	CCCCAAGGGCCCGTGCCACCGG	tag66981
novel_sir4172	GGTGGCGAGTTGGTGCTTCGGTGG	ACCGCTCAACCACGAAGCCACCGC	tag125988
novel_sir4173	AACCTAGGTCGTCTAGCCCACCAC	GCTTGGATCCAGCAGATCGGGTGG	tag248854
novel_sir4174	AACCCAGATCGTCTAGCCCACCAC	GCTTGGGTCTAGCAGATCGGGTGG	tag122441
novel_sir4175	CCGCCGCCGCCGACCCACCCG	CGGGCGGCGGCGGCTGGGTGG	tag150318
novel_sir4176	CGGGCGGTGGCGGCTGGGTGG	CCGCCACCGCCGACCCACCCG	tag141628
novel_sir4177	AGCTGCGGCCGCTAGTGGTGG	GACGCCGGCGATCACCACCAC	tag285966
novel_sir4178	TGCGGTGCATCTGGTTTTGGTGG	GCCACGTAGACCAAAACCACCGC	tag285141
novel_sir4179	TGTAGTCGCAGGTTAAAAATGTGG	ATCAGCGTCCAATTTTTACACCTA	tag57894
novel_sir4180	CGTCCTCGGCTCGTACACCGC	AAGCAGGAGCCGAGCATGTGG	tag248973
novel_sir4181	GAAAAGCCACTTATACACCAG	CTCTTTTCGGTGAATATGTGG	tag60674

novel_sir4182	AGAAAAGCCACTTATACACCAG	TCTCTTTTCGGTGAATATGTGG	tag108824
novel_sir4183	TTGAATAAATTACGATACCTGTGG	CTTATTTAATGCTATGGACACCAT	tag79355
novel_sir4184	TCGAATAAATTACGATACCTGTGG	CTTATTTAATGCTATGGACACCAT	tag188182
novel_sir4185	TTGAATAAATTATGATACCTGTGG	CTTATTTAATACTATGGACACCAT	tag51592
novel_sir4186	TCGAATAAATTATGATACCTGTGG	CTTATTTAATACTATGGACACCAT	tag279084
novel_sir4187	ACACTTTTTGCCAGCGGACACCGC	TTTGTGAAAAACGGTCGCCTGTGG	tag39516
novel_sir4188	CTCTACGCCTAGGACACCAG	AGGAGATGCGGATCCTGTGG	tag103660
novel_sir4189	GACTTCCGGCTAAGCCTCCTGTGG	GAAGGCCGATTCGGAGGACACCGT	tag262271
novel_sir4190	GGGAGCGGCCGGCGGCTGTGG	CTCGCCGGCCGCCGACACCCT	tag285344
novel_sir4191	ATTAGCCATTAGATGTCACACCCT	GGTAATCGGTAATCTACAGTGTGG	tag68174
novel_sir4192	GCCAGCCGCCGGCCACACCGG	GACGGTCGGCGGCCGGTGTGG	tag286534
novel_sir4193	CCGGATCCGCATCTCCTTGGTGTGG	CCTAGGCGTAGAGGAACCACACCAA	tag71011
novel_sir4194	CTGGATCCGCATCTCCTTGGTGTGG	CCTAGGCGTAGAGGAACCACACCAA	tag136995
novel_sir4195	AAGGATCCGCATCTCCTTGGTGTGG	CCTAGGCGTAGAGGAACCACACCAA	tag211065
novel_sir4196	CAGGATCCGCATCTCCTTGGTGTGG	CCTAGGCGTAGAGGAACCACACCAA	tag212053
novel_sir4197	CCGGATCCGCATCTCCTTGGTGTGG	CCTAGGCGTAGAGGAACCACACCAC	tag71011
novel_sir4198	CTGGATCCGCATCTCCTTGGTGTGG	CCTAGGCGTAGAGGAACCACACCAC	tag136995
novel_sir4199	AAGGATCCGCATCTCCTTGGTGTGG	CCTAGGCGTAGAGGAACCACACCAC	tag211065
novel_sir4200	CAGGATCCGCATCTCCTTGGTGTGG	CCTAGGCGTAGAGGAACCACACCAC	tag212053
novel_sir4201	CAGGCCCTCGCGTGTACACACCAT	GTGTCCGGGAGCGCACATGTGTGG	tag133438
novel_sir4202	CGCGTAGCCGATGAACACCCC	AGGCGCATCGGCTACTTGTGG	tag154705
novel_sir4203	CGGCGATGCCGGGAGCTTGTGG	CGCTACGGCCCTCGAACACCCG	tag209050
novel_sir4204	CACCGCCTGTCATGTTTTAACCAG	CTGTGGCGGACAGTACAAAATTGG	tag16839
novel_sir4205	GGGTGTGCTTGGACGGAATTGG	CACACGAACCTGCCTTAACCCA	tag199166
novel_sir4206	TGGTGGCTTCCTGAATAACATTGG	CACCGAAGGACTTATTGTAACCAG	tag152972
novel_sir4207	CGCTGTCCGGAAGACTGTAACCAC	TAGCGACAGGCCTTCTGACATTGG	tag244630
novel_sir4208	CCCCACTTTTTGAATCTGAACCAG	TGGGGGTGAAAAACTTAGACTTGG	tag280835
novel_sir4209	GTTGGATCATGCTCTCCTTGG	ACCTAGTACGAGAGGAACCGT	tag270771
novel_sir4210	GCGTTGTCATCGAGCGCTTGG	CAACAGTAGCTCGCGAACCGG	tag290117
novel_sir4211	ATCCTCCTGCGAAAATAGAACCAC	AATAGGAGGACGCTTTTATCTTGG	tag271138
novel_sir4212	AGCCGGCTTAGACACAAGAACCAA	TGTCGGCCGAATCTGTGTTCTTGG	tag71508
novel_sir4213	AGCCGGCTTAGACACAAGAACCAA	TATCGGCCGAATCTGTGTTCTTGG	tag256554
novel_sir4214	CGCCACTGGCTTTTCAACCAA	GCGCGGTGACCGAAAAGTTGG	tag71033
novel_sir4215	GCGCCACTGGCTTTTCAACCAA	AGCGCGGTGACCGAAAAGTTGG	tag227246

novel_sir4216	CGCGCCACTGGCTTTTCAACCAA	AAGCGCGGTGACCGAAAAGTTGG	tag124438
novel_sir4217	ATCCGATTTTCGTCCTTCAACCAG	CTTAGGCTAAAAGCAGGAAGTTGG	tag177201
novel_sir4218	ACGTGACGTACGCGTGGCAACCTA	GATGCACTGCATGCGCACCGTTGG	tag147421
novel_sir4219	CGCGCATGCGCAGGTGATCGTTGG	GCGTACGCGTCCACTAGCAACCCC	tag272406
novel_sir4220	CAGCCTGACAACGTGTTAAACCAG	GTGTCGGACTGTTGCACAATTTGG	tag133961
novel_sir4221	ACGGCTCGACAACAATTAAACCAG	TGTGCCGAGCTGTTGTTAATTTGG	tag52590
novel_sir4222	AAGCAGACTTGTGTAGTAAACCAA	GGTTCGTCTGAACACATCATTTGG	tag125339
novel_sir4223	ACTGCAGTGTGAGTAAACCAA	GGTGACGTCACACTCATTTGG	tag22412
novel_sir4224	AACATTGTTGGATACGGAAACCCA	AGTTGTAACAACCTATGCCTTTGG	tag185122
novel_sir4225	AGCCAAAACCGCCATCGAAACCGC	AGTCGGTTTTGGCGGTAGCTTTGG	tag114298
novel_sir4226	AGCGTGGCATTTGGACGAAACCCA	GGTCGCACCGTAAACCTGCTTTGG	tag228795
novel_sir4227	ATCTCTGTAAATAGTTCTCTTTGG	GAGACATTTATCAAGAGAAACCAT	tag112134
novel_sir4228	CTTCTTTCGGCCCGTGCAAACCCA	GGGAAGAAAGCCGGGCACGTTTGG	tag45560
novel_sir4229	ACTGCCACGTGGATCCAAAACCAC	CGTGACGGTGCACCTAGGTTTTGG	tag237372
novel_sir4230	CATCCACGGTGCAGTCGGTTTTGG	AGGTGCCACGTCAGCCAAAACCGC	tag193336
novel_sir4231	CATACGCGGTGCAGTCGGTTTTGG	ATGCGCCACGTCAGCCAAAACCAC	tag94281
novel_sir4232	ATGCGCCACGTCAGCCAAAACCGC	CATACGCGGTGCAGTCGGTTTTGG	tag94281
novel_sir4233	CATCCGCGGTGCAGTCGGTTTTGG	AGGCGCCACGTCAGCCAAAACCAC	tag283946
novel_sir4234	AGACGCCACGTCAGCCAAAACCGC	CATCTGCGGTGCAGTCGGTTTTGG	tag204733
novel_sir4235	AGGTACCACGTCAGCCAAAACCGC	CATCCATGGTGCAGTCGGTTTTGG	tag76844
novel_sir4236	ACTGCCACGTGGACCCAAAACCAC	CCTGACGGTGCACCTGGGTTTTGG	tag186124
novel_sir4237	ACCTGACGGTGCATCTGGTTTTGG	GACTGCCACGTAGACCAAAACCAC	tag177444
novel_sir4238	GACTGCCACGTAGACCAAAACCGC	ACCTGACGGTGCATCTGGTTTTGG	tag177444
novel_sir4239	CGTGTATGTCGGACAGCTTTTTGG	ACATACAGCCTGTCGAAAAACCAT	tag135626
novel_sir4240	GACTCGCGGTATCTGGTTTTACGG	CCCTGAGCGCCATAGACCAAAATG	tag78890
novel_sir4241	CTGACGATCGCTTCTGGACAAATG	CTGCTAGCGAAGACCTGTTTACGC	tag60714
novel_sir4242	GTCGCACCCGTTGCCGAAATG	GCGTGGGCAACGGCTTTACCT	tag277467
novel_sir4243	AAAATAGTCGGCATGTATTTACAT	TTTTTTATCAGCCGTACATAAATG	tag28530
novel_sir4244	AAAATAGTCGACATGTATTTACAT	TTTTTTATCAGCTGTACATAAATG	tag266793
novel_sir4245	ATGTCGCGGTTCCGGTATTTACAT	ACTACAGCGCCAAGGCCATAAATG	tag251299
novel_sir4246	ATCGACCCACCGGGTGCATAAATG	GCTGGGTGGCCCACGTATTTACGC	tag79720
novel_sir4247	TAGAAATCAGGGCCAACCACAATG	CTTTAGTCCCGGTTGGTGTTACCA	tag149538
novel_sir4248	GGCCGGAAACGAGTAGCCTCAATG	GGCCTTTGCTCATCGGAGTTACAG	tag110973
novel_sir4249	CGTAATTGCCGAATCTTTAGAATG	ATTAACGGCTTAGAAATCTTACTG	tag92498

novel_sir4250	AGTATATAACAGCGGCCTTACGT	TGTCATATATTGTCGCCCGGAATG	tag10791
novel_sir4251	AAGGCCACTCTGCCACTTACAA	CGTTCCGGTGAGACGGTGAATG	tag110068
novel_sir4252	AAGGCCACTCTGCCACTTACAA	TGTTCCGGTGAGACGGTGAATG	tag180475
novel_sir4253	TGTCTAGTACTGAACCCATAATG	AGATCATGACTTGGGTATTACGT	tag44500
novel_sir4254	CTCCTGTTCTGACTGCTCCTAATG	GGACAAGACTGACGAGGATTACGA	tag63241
novel_sir4255	GATAGTGGCCCGCGCAGATTACGC	TACTATCACCGGGCGCGTCTAATG	tag195049
novel_sir4256	AGGGTGGACAGTATGTGTGTAATG	CCACCTGTCATACACACATTACGT	tag284711
novel_sir4257	ACTACGATTGTACTGTAATTACAT	TGTGATGCTAACATGACATTAATG	tag263193
novel_sir4258	TGTGATGCTAACATGATATTAATG	ACTACGATTGTACTATAATTACAT	tag49011
novel_sir4259	TTGAAATTTATGGCCTGCTTAATG	CTTTAAATACCGGACGAATTACCC	tag280437
novel_sir4260	TTGATACGTTTGGCCTGCTTAATG	CTATGCAAACCGGACGAATTACCC	tag187104
novel_sir4261	ACGCGGTCCACTGGCAAATTACCA	GCTGCGCCAGGTGACCGTTTAATG	tag32513
novel_sir4262	TGTTCAACGACCCTAGTAAACATG	AAGTTGCTGGGATCATTTGTACCC	tag152807
novel_sir4263	TAGATCAGATAACAACTCCACATG	CTAGTCTATTGTTGAGGTGTACAT	tag102905
novel_sir4264	ACGACTGACCTGACGGTGCACATG	CTGACTGGACTGCCACGTGTACGC	tag52493
novel_sir4265	AAGGCCAAGGGTGGTGATGTACGT	GGTTCCGGTTCCCACCACTACATG	tag47824
novel_sir4266	TAGCTCAGATAACGACTCTACATG	CGAGTCTATTGCTGAGATGTACAT	tag89489
novel_sir4267	GGCAAGCCGAGCAATGTACCC	ACCCGTTCGGCTCGTTACATG	tag95336
novel_sir4268	CTCTAGGAGACGTCAGTTTACATG	GATCCTCTGCAGTCAAATGTACGG	tag13209
novel_sir4269	GGGACCTAGTCATCAACCATG	CTGGATCAGTAGTTGGTACAC	tag40430
novel_sir4270	TTCCGTGTGAAGCAGCCCATG	GGCACACTTCGTCGGGTACAG	tag74831
novel_sir4271	GTTCCGTGTGAAGCAGCCCATG	AGGCACACTTCGTCGGGTACAG	tag141381
novel_sir4272	AGTGCCCGATCAAGGCCAGCCATG	ACGGGCTAGTTCCGGTCGGTACTA	tag127983
novel_sir4273	GCCTGTAGAGCAATAGCTGCCATG	GACATCTCGTTATCGACGGTACGT	tag232989
novel_sir4274	CTCCTCGAGGCAGCCTGATCCATG	GGAGCTCCGTCGGACTAGGTACTT	tag86093
novel_sir4275	CACCAAAACCAGATGCACCGCATG	GGTTTTGGTCTACGTGGCGTACAC	tag155645
novel_sir4276	GGTTTTGGTCTACGTGGCGTACGC	CACCAAAACCAGATGCACCGCATG	tag155645
novel_sir4277	CATCAAAACCAGATGCACCGCATG	AGTTTTGGTCTACGTGGCGTACGC	tag261483
novel_sir4278	ACTGACCTGACGGTGCGCATG	ACTGGACTGCCACGCGTACGT	tag283609
novel_sir4279	ACGACTGACCTGACGGTGCGCATG	CTGACTGGACTGCCACGCGTACGC	tag182498
novel_sir4280	GGCGTTCGAGCCGGAGTACGG	ATCCGCAAGCTCGGCCTCATG	tag4711
novel_sir4281	AGTAGGCCGGCGCGTGAAGTCATG	ATCCGGCCGCGCACTTCAGTACCT	tag166776
novel_sir4282	TATTCTGCTCACCAGTTTGTCATG	AAGACGAGTGGTCAAACAGTACAA	tag271247
novel_sir4283	TGTGTGTGCTATACTGCAAAGATG	ACACACGATATGACGTTTCTACCC	tag85885

novel_sir4284	TCTGTATTACAAGTGAAGATG	ACATAATGTTCACTTCTACTC	tag93964
novel_sir4285	CAGTAACTGAGTACACCTAAGATG	CATTGACTCATGTGGATTCTACAT	tag14532
novel_sir4286	CCTCGGCGTCGGTGTCTACCT	CAGGAGCCGCAGCCACAGATG	tag32586
novel_sir4287	GTTAGGCGACACCAAAACCAGATG	ATCCGCTGTGGTTTTGGTCTACGT	tag290273
novel_sir4288	AGGCCTGTGGCACCGTCTACGA	GTTCCGGACACCGTGGCAGATG	tag263001
novel_sir4289	CTGAGAGCTGGTGCCAGAGATG	CTCTCGACCACGGTCTCTACTA	tag130412
novel_sir4290	CCCATGCGACCATGCTCCTAGATG	GTACGCTGGTACGAGGATCTACGT	tag155840
novel_sir4291	CAGCAAGAACCCGGTGGCTAGATG	CGTTCTTGGGCCACCGATCTACGT	tag228831
novel_sir4292	CGTCTTGGAGTCCGTGCTACAG	ACGCAGAACCTCAGGCACGATG	tag136160
novel_sir4293	AAGTTATTTGGATGGGGCTACGG	CCTTCAATAAACCTACCCCGATG	tag49413
novel_sir4294	GAAGTTATTTGGATGGGGCTACGG	ACCTTCAATAAACCTACCCCGATG	tag137672
novel_sir4295	GGTGAAAATCCAGAGCACGCGATG	ACTTTTAGGTCTCGTGCGCTACCC	tag99903
novel_sir4296	GAGCGGCTGCCGCCGATCGATG	CGCCGACGGCGGCTAGCTACGG	tag244010
novel_sir4297	TTCCTGGCCAAGCGCTCGATG	GGACCGGTTCGCGAGCTACTG	tag220122
novel_sir4298	TTTCGGTCACCGCGCTTCGATG	AGCCAGTGGCGCGAAGCTACCG	tag131567
novel_sir4299	CCTCCACGGCACTTGCTGATG	AGGTGCCGTGAACGACTACAA	tag256031
novel_sir4300	CGGTCTAGGTCTCCGGTGATG	CAGATCCAGAGGCCACTACTT	tag284089
novel_sir4301	CTCCTGACGTAGCTGGTGATG	GGACTGCATCGACCACTACGA	tag41452
novel_sir4302	AATAGAGTTAGCCCGGATTTGATG	ATCTCAATCGGGCCTAAACTACGG	tag134024
novel_sir4303	ATTGCCCGTGGACGCCTTATACCC	GGTAACGGGCACCTGCGGAATATG	tag31747
novel_sir4304	AGAGAAAAGCCACTTATACAC	GTTCTCTTTTCGGTGAATATG	tag143703
novel_sir4305	AAGAGAAAAGCCACTTATACAC	CGTTCTCTTTTCGGTGAATATG	tag183780
novel_sir4306	GCAAGAGAAAAGCCACTTATACAC	TGCGTTCTCTTTTCGGTGAATATG	tag187934
novel_sir4307	TGTGTTCTGTGCCCGCTACATATG	ACAAGACACGGGCGATGTATACAG	tag120767
novel_sir4308	AAGGGTAACAATCGCGATATG	CCCATTGTTAGCGCTATACTG	tag229958
novel_sir4309	GGTATAACCGTACCTGCCTATATG	ATATTGGCATGGACGGATATACCC	tag149114
novel_sir4310	TTCTGGAATACTGATCGGTATATG	GACCTTATGACTAGCCATATACAT	tag232256
novel_sir4311	GTTCGGGTGGCAGAGTGCACTATG	AGCCCACCGTCTCACGTGATACGA	tag254095
novel_sir4312	ACTCCGAAACCTGGCCCTACTATG	AGGCTTTGGACCGGGATGATACAT	tag115308
novel_sir4313	GCTATATACTCGTTGGATACAT	TCCGATATATGAGCAACCTATG	tag244256
novel_sir4314	ACGGACAGTTAAAGTTGGATACGG	TCTGCCTGTCAATTTCAACCTATG	tag245099
novel_sir4315	TCTGCCTGTCAGTTTCAACCTATG	ACGGACAGTCAAAGTTGGATACGG	tag274206
novel_sir4316	TTTGCCTGTCAGTTTCAACCTATG	ACGGACAGTCAAAGTTGGATACGG	tag282140
novel_sir4317	GCGCCTCGCTGTCGACGGATACGT	TCCGCGGAGCGACAGCTGCCTATG	tag52535

novel_sir4318	AATTGCCAAAATCGAGATCCTATG	AACGGTTTTAGCTCTAGGATACGC	tag266974
novel_sir4319	AGGGCCTGTGTGCATAGGATACGC	GTTCCCGGACACACGTATCCTATG	tag121132
novel_sir4320	GTTCCCGGACACACGTATCCTATG	AGGGCCTGTGTGCATAGGATACAC	tag121132
novel_sir4321	GCTCCCGGACACACGTATCCTATG	AGGGCCTGTGTGCATAGGATACAC	tag143408
novel_sir4322	CTGCAGCGCTCGAGTTGCTATG	CGTCGCGAGCTCAACGATACTC	tag98444
novel_sir4323	TGGGTAGGTCTGTACAGGTCTATG	CCATCCAGACATGTCCAGATACAT	tag41334
novel_sir4324	CCATCACATCAACCTGTCATACAC	AAGGTAGTGTAGTTGGACAGTATG	tag101165
novel_sir4325	AACGGCCACCGAATCTGCATACGA	GTTTGCCGGTGGCTTAGACGTATG	tag51656
novel_sir4326	GGTTTTGGTCTACGTGGCATACAC	CACCAAAACCAGATGCACCGTATG	tag203366
novel_sir4327	GGCGATCCAGATATGGGTATG	GCTAGGTCTATACCCATACGA	tag53696
novel_sir4328	AGAAGCCTTATATTCGGAATACGA	TGTCTTCGGAATATAAGCCTTATG	tag12254
novel_sir4329	AGCTTGTGCCTATCGCGGCTTATG	GAACACGGATAGCGCCGAATACGG	tag134390
novel_sir4330	GTTGCCGCAGTATGTAATTTTATG	ACGGCGTCATACATTAAAATACGG	tag41831
novel_sir4331	ACGGCGCCATACATTAAAATACGG	GCTGCCGCGGTATGTAATTTTATG	tag153565
novel_sir4332	CATTTTGCTTGCACTGTTTGACCA	CTGTAAAACGAACGTGACAAACTG	tag231178
novel_sir4333	AACTGAAAAAGAGTGTACAAACTG	GACTTTTTCTCACATGTTTGACCT	tag161518
novel_sir4334	AACTGAAAAAGAGTGTACAAACTG	GACTTTTTCTCACATGTTTGACAA	tag161518
novel_sir4335	AACTGAAAAATAGTGTACAAACTG	GACTTTTTATCACATGTTTGACCG	tag253392
novel_sir4336	GACTTTTTATCACATGTTTGACTG	AACTGAAAAATAGTGTACAAACTG	tag253392
novel_sir4337	AACTGAAAAATAGTGTACAAACTG	GACTTTTTATCACATGTTTGACCA	tag253392
novel_sir4338	ACTGAAAAATCGTGTACAAACTG	ACTTTTTAGCACATGTTTGACCA	tag130033
novel_sir4339	ACTGAAAAATCGTGTACAAACTG	ACTTTTTAGCACATGTTTGACCG	tag130033
novel_sir4340	AACTGAAAAATCGTGTACAAACTG	GACTTTTTAGCACATGTTTGACCT	tag213819
novel_sir4341	AACTGAAAAATCGTGTACAAACTG	GACTTTTTAGCACATGTTTGACCG	tag213819
novel_sir4342	AACTGAAAAATCGTGTACAAACTG	GACTTTTTAGCACATGTTTGACTA	tag213819
novel_sir4343	GACTTTTTTAAACATGTTTGACCA	AACTGAAAAAATTTGTACAAACTG	tag175432
novel_sir4344	AACTGAAAAAATTTGTACAAACTG	GACTTTTTTAAACATGTTTGACCT	tag175432
novel_sir4345	AACTGAAAAAATTTGTACAAACTG	GACTTTTTTAAACATGTTTGACTG	tag175432
novel_sir4346	AACTGAAAAAATTTGTACAAACTG	GACTTTTTTAAACATGTTTGACAA	tag175432
novel_sir4347	GACTTTTTTAAACATGTTTGACTA	AACTGAAAAAATTTGTACAAACTG	tag175432
novel_sir4348	GACTTTTTTAAACATGTTTGACCG	AACTGAAAAAATTTGTACAAACTG	tag175432
novel_sir4349	AGGGAGGCAAAGCTTTACAAACTG	CCTCCGTTTCGAAATGTTTGACAC	tag47480
novel_sir4350	GACTTTTCGATCAACGTTTGACCA	AACTGAAAAGCTAGTTGCAAACTG	tag118238
novel_sir4351	AACTGAAAAGCTAGTTGCAAACTG	GACTTTTCGATCAACGTTTGACTA	tag118238

novel_sir4352	AACTGAAAAACAGGTTGCAAACTG	GACTTTTTGTCCAACGTTTGACCA	tag221550
novel_sir4353	CCTATAGGCACAGGTTGCAAACTG	ATATCCGTGTCCAACGTTTGACGT	tag32735
novel_sir4354	CTTATAGGCACAGGTTGCAAACTG	ATATCCGTGTCCAACGTTTGACGT	tag242559
novel_sir4355	CCTATAGGCACAGGTTGCAAACTG	ATATCCGTGTCCAACGTTTGACCG	tag32735
novel_sir4356	CTTATAGGCACAGGTTGCAAACTG	ATATCCGTGTCCAACGTTTGACCG	tag242559
novel_sir4357	CCTATAGGCACAGGTTGCAAACTG	ATATCCGTGTCCAACGTTTGACTG	tag32735
novel_sir4358	CTTATAGGCACAGGTTGCAAACTG	ATATCCGTGTCCAACGTTTGACTG	tag242559
novel_sir4359	CCTTAGGCACAGGTTGCAAACTG	AATCCGTGTCCAACGTTTGACCG	tag99712
novel_sir4360	ACTGAAAAATAGGTTGCAAACTG	ACTTTTTATCCAACGTTTGACCA	tag179831
novel_sir4361	ATATCTGTGCCCAACGTTTGACCG	CCTATAGACACGGGTTGCAAACTG	tag254269
novel_sir4362	ATATCCGTGCCCAACGTTTGACCG	CCTATAGGCACGGGTTGCAAACTG	tag233328
novel_sir4363	AACTGAAAAGCTGGTTGCAAACTG	GACTTTTCGACCAACGTTTGACCA	tag187065
novel_sir4364	AACTGAAAAGCTGGTTGCAAACTG	GACTTTTCGACCAACGTTTGACTA	tag187065
novel_sir4365	ACTTTTTAACCAACGTTTGACCA	ACTGAAAAATTGGTTGCAAACTG	tag211439
novel_sir4366	AACTGAAAAATTGGTTGCAAACTG	GACTTTTTAACCAACGTTTGACAA	tag200744
novel_sir4367	AACTGAAAAATTGGTTGCAAACTG	GACTTTTTAACCAACGTTTGACCA	tag200744
novel_sir4368	GACTTTATAACCAACGTTTGACCA	AACTGAAATATTGGTTGCAAACTG	tag199833
novel_sir4369	GACTTTTCAACCAACGTTTGACCA	AACTGAAAAGTTGGTTGCAAACTG	tag105738
novel_sir4370	CTTTACAAACTGCGGCAACTG	AATGTTTGACGCCGTTGACTT	tag138817
novel_sir4371	AGCGCCGCGATGGCCTTGACGG	GCTCGCGGCGCTACCGGAACTG	tag103500
novel_sir4372	GGCTATTAGGAACTCTGCACACTG	GATAATCCTTGAGACGTGTGACTT	tag82205
novel_sir4373	ACTCGTTTATCCGGTCTGTGACCA	CGTGAGCAAATAGGCCAGACACTG	tag47387
novel_sir4374	CGTGATCTGACCGGTCTGTGACTG	GGGCACTAGACTGGCCAGACACTG	tag257936
novel_sir4375	TGTGACCGGTTCCAGTGGACACTG	ACTGGCCAAGGTCACCTGTGACGG	tag132144
novel_sir4376	TAGGCCAGTTTGCAGACTACACTG	CCGGTCAAACGTCTGATGTGACAC	tag51221
novel_sir4377	CGCTGGACCTCCCCGTGACCC	TGGCGACCTGGAGGGGCACTG	tag76195
novel_sir4378	CAGAGAATTGGTGAGTGACGC	TGGTCTCTTAACCACTCACTG	tag22040
novel_sir4379	ATCGGGCCTATACGTGAGTGACAA	TGTAGCCCGGATATGCACTCACTG	tag172053
novel_sir4380	AGCATGTGCCGCCGAAAGTGACAG	CGTCGTACACGGCGGCTTTCACTG	tag257787
novel_sir4381	AATCCACCGCTGACCGCCAGACTG	AGGTGGCGACTGGCGGTCTGACGC	tag40099
novel_sir4382	CCCCAGAGCTCGGGGTGATGACTG	GGTCTCGAGCCCCACTACTGACGT	tag53482
novel_sir4383	CAAGACGCAGTGTGTGTATGACAG	AGGTTCTGCGTCACACACATACTG	tag206699
novel_sir4384	AGGGAGGCATAAAATTACATACTG	CCTCCGTATTTTAATGTATGACGC	tag115793
novel_sir4385	CAACTATCTCAGGCTATATGACGT	AGGTTGATAGAGTCCGATATACTG	tag172313

novel_sir4386	CGTGGCTGCCGTTGATGACGT	CGGCACCGACGGCAACTACTG	tag2793
novel_sir4387	CGGTAACAAAAACCGCGGCTACTG	CATTGTTTTTGGCGCCGATGACAT	tag182242
novel_sir4388	ACTATAGCCCGTCACAGATGACCT	TTTGATATCGGGCAGTGTCTACTG	tag241837
novel_sir4389	TGTGTTCAGACGAACCTTCTACTG	ACAAGTCTGCTTGGAAGATGACCA	tag218418
novel_sir4390	ACTGCGCCGTTCGACTCAGTACTG	ACGCGGCAAGCTGAGTCATGACAC	tag77084
novel_sir4391	GTCATTGGGTGTACACGATTACTG	GTAACCCACATGTGCTAATGACGG	tag214107
novel_sir4392	ACTGCGCACCACCTGTTCTTACTG	ACGCGTGGTGGACAAGAATGACCG	tag129245
novel_sir4393	ATCACGACAACAAATTTTGGACGG	TGTAGTGCTGTTGTTTAAAACCTG	tag265051
novel_sir4394	ACTCCCAGCGTGGCATTTGGACGA	GGTGAGGGTCGCACCGTAAACCTG	tag172765
novel_sir4395	ACGTGTAATTAGCTTGTTGGACAC	TTTGCACATTAATCGAACAACCTG	tag59784
novel_sir4396	GACATGCAATAAATGGTTGGACGT	TACTGTACGTTATTTACCAACCTG	tag174287
novel_sir4397	ATTCTGCCTGTCAATTTCAACCTG	AGACGGACAGTTAAAGTTGGACAC	tag153160
novel_sir4398	CTCGATCGGCCTTCTGGGAACCTG	GCTAGCCGGAAGACCCTTGGACGT	tag21899
novel_sir4399	TGTCCAAGCATCGATCCTAACCTG	AGGTTCGTAGCTAGGATTGGACTT	tag53404
novel_sir4400	GGTCCAAGCATCGATCCTAACCTG	AGGTTCGTAGCTAGGATTGGACTT	tag130697
novel_sir4401	GACATGCAATAAACGGGTGGACGC	TACTGTACGTTATTTGCCCACCTG	tag15338
novel_sir4402	TACTGTACGTTATTTGCCCACCTG	GACATGCAATAAACGGGTGGACGT	tag15338
novel_sir4403	AACTGTACGTTATTTGCCCACCTG	GACATGCAATAAACGGGTGGACGT	tag186116
novel_sir4404	GACATACAATAAACGGGTGGACGT	TACTGTATGTTATTTGCCCACCTG	tag237102
novel_sir4405	TACTGTACGTTCTTTGCCCACCTG	GACATGCAAGAAACGGGTGGACGT	tag232837
novel_sir4406	GACATGTTAGAAATAGGTGGACGT	TACTGTACAATCTTTATCCACCTG	tag127310
novel_sir4407	GCAGACGAAGCAGGTGGACGC	GTCGTCTGCTTCGTCCACCTG	tag156919
novel_sir4408	TTCTAGCACATAAATGAGCACCTG	GATCGTGTATTTACTCGTGGACAC	tag237986
novel_sir4409	GGAGAGATGGCTGAGTGGACTA	CCCCTCTCTACCGACTCACCTG	tag82817
novel_sir4410	CCCCTCTCTACCGACTCACCTG	GGAGAGATGGCTGAGTGGACTC	tag82817
novel_sir4411	GGAGAGATGGCTGAGTGGACAA	CCCCTCTCTACCGACTCACCTG	tag82817
novel_sir4412	ACCTAAGATTATCGAGCTCACCTG	GATTCTAATAGCTCGAGTGGACGT	tag239822
novel_sir4413	GTTGAAAACCGATACTTAGACCTG	ACTTTTGGCTATGAATCTGGACAT	tag66223
novel_sir4414	GTTGAAAACCGATACTTAGACCTG	ACTTTTGGCTATGAATCTGGACGC	tag66223
novel_sir4415	ACTTTTGGCTATGAATCTGGACAA	GTTGAAAACCGATACTTAGACCTG	tag66223
novel_sir4416	GTTGAAAACCGATACTTAGACCTG	ACTTTTGGCTATGAATCTGGACAC	tag66223
novel_sir4417	ACTTTTAGCTATGAATCTGGACAA	GTTGAAAATCGATACTTAGACCTG	tag284800
novel_sir4418	ACTTTTAGCTATGAATCTGGACAT	GTTGAAAATCGATACTTAGACCTG	tag284800
novel_sir4419	ACTTTTAGCTATGAATCTGGACAC	GTTGAAAATCGATACTTAGACCTG	tag284800

novel_sir4420	ACTCGTCTGCTGAGGTTCGACCTG	AGCAGACGACTCCAAGCTGGACGG	tag235600
novel_sir4421	GGTGCCGACGTGAATAAAACCCTG	ACGGCTGCACTTATTTTGGGACGG	tag35020
novel_sir4422	GAGAATAAACGATATCTGACCCTG	CTTATTTGCTATAGACTGGGACCG	tag209247
novel_sir4423	ACTTCCGAGCCGTGGGGACTC	GATGAAGGCTCGGCACCCCTG	tag223589
novel_sir4424	GGCTGATCGTACCACTCCCTG	GACTAGCATGGTGAGGGACGC	tag53818
novel_sir4425	CGGCTGATCGTACCACTCCCTG	CGACTAGCATGGTGAGGGACGC	tag165943
novel_sir4426	AATATCAAGACGTGGCAGGGACCC	AGTTATAGTTCTGCACCGTCCCTG	tag80766
novel_sir4427	TGGCCAAAACCAATCAATTCCCTG	CGGTTTTGGTTAGTTAAGGGACGT	tag46609
novel_sir4428	TTTGGTACTAGCCCTATCGCCTG	ACCATGATCGGGATAGCGGACGT	tag130012
novel_sir4429	TTTTGGTACTAGCCCTATCGCCTG	AACCATGATCGGGATAGCGGACGT	tag76743
novel_sir4430	ATTTGGTACTAGCCCTATCGCCTG	AACCATGATCGGGATAGCGGACGT	tag226868
novel_sir4431	AAAACACTTTTTGCCAGCGGACAC	CCTTTTGTGAAAAACGGTCGCCTG	tag28392
novel_sir4432	ACTGACAGTCAAAACGCCGGACAC	TCTGACTGTCAGTTTTGCGGCCTG	tag271610
novel_sir4433	CCTATCGACTATGAATACGGACAT	CTGGATAGCTGATACTTATGCCTG	tag120470
novel_sir4434	GGCTGAGTTTGTGCCTACTGCCTG	GACTCAAACACGGATGACGGACCG	tag293408
novel_sir4435	GGCAAATGATATATCCACGGACAT	GTCCGTTTACTATATAGGTGCCTG	tag25854
novel_sir4436	ACTGAGTTGATGATTTGAATCCTG	ACTCAACTACTAAACTTAGGACAT	tag20085
novel_sir4437	GGACAGCTGCGGTTAGTAGGACAG	ATCCTGTCGACGCCAATCATCCTG	tag34405
novel_sir4438	TCTCTAGCGAAAGCTCAGATCCTG	AGATCGCTTTCGAGTCTAGGACGT	tag293554
novel_sir4439	TTCTGATCAGACTACCCACTCCTG	GACTAGTCTGATGGGTGAGGACCG	tag217443
novel_sir4440	ATGATGCTTAGATCTGTACTCCTG	CTACGAATCTAGACATGAGGACAT	tag155855
novel_sir4441	GCAAACGAAAGAGGAGGACTA	CCCGTTTGCTTTCTCCTCCTG	tag114253
novel_sir4442	CATCTGTGGCTGCGGCTCCTG	AGACACCGACGCCGAGGACGC	tag142813
novel_sir4443	CTCCGGCCGCGTGTCTCCTG	GGCCGGCGCACAGAGGACGA	tag231782
novel_sir4444	AGGCCGGCGCACAGAGGACGA	CCTCCGGCCGCGTGTCTCCTG	tag10307
novel_sir4445	GGCCGGCGAGAGGCTGGTCCTG	GGCCGCTCTCCGACCAGGACAT	tag50875
novel_sir4446	TGTATGGCGATGTTCAAATTCCTG	ATACCGCTACAAGTTTAAGGACCG	tag175236
novel_sir4447	TGTGGGCGCGAGTTGAAATTCCTG	ACCCGCGCTCAACTTTAAGGACCG	tag145375
novel_sir4448	CCGATTTTGTATAGCTAAGGACTA	CTGGCTAAAACATATCGATTCCTG	tag104097
novel_sir4449	CATCGGATAGATTGACAACAGCTG	AGCCTATCTAACTGTTGTCGACAT	tag284127
novel_sir4450	GGCCGGCGGCGGCGTCGACGC	CGCCGGCCGCCGCCGCAGCTG	tag65315
novel_sir4451	CCACTTATACACCAGTCGACGA	TCGGTGAATATGTGGTCAGCTG	tag50972
novel_sir4452	CGCTCACGGTTAGATCGACGC	CCGCGAGTGCCAATCTAGCTG	tag231718
novel_sir4453	CGGCGACGGCGGAATCGACGG	CCGCCGCTGCCGCCTTAGCTG	tag268928

novel_sir4454	GCCGCCGCTGCCGCCTTAGCTG	GCGGCGACGGCGGAATCGACGG	tag274626
novel_sir4455	AGGAGCCTACGCGTCGGGCGACCG	TCTCCTCGGATGCGCAGCCCGCTG	tag107477
novel_sir4456	AGGTCCGGCGGCCGGCGACGT	CCTCCAGGCCGCCGGCCGCTG	tag246551
novel_sir4457	GCTGCCTACTGCCGGCCGCTG	ACGGATGACGGCCGGCGACGA	tag24260
novel_sir4458	TCTCCTCCCGCCGCGGCGCTG	AGGAGGGCGGCGCCGCGACGG	tag70834
novel_sir4459	GAGGAGGGCGGCGCCGCGACGG	TTCTCCTCCCGCCGCGGCGCTG	tag136979
novel_sir4460	CCTTAGATAGGCCGGCCGACCT	CGGGAATCTATCCGGCCGGCTG	tag133284
novel_sir4461	GTCCTCGACGCGGCGGCGGCTG	GGAGCTGCGCCGCCGCCGACGC	tag235098
novel_sir4462	CGCCGCCGCCGACGCCGACAG	ACGCGGCGGCGGCTGCGGCTG	tag162216
novel_sir4463	AGGTTTGGGTAGCCAGCGGGCTG	CAAACCCATCGGTCGCCCGACGC	tag32547
novel_sir4464	GAGGTTTGGGTAGCCAGCGGGCTG	CCAAACCCATCGGTCGCCCGACGC	tag88776
novel_sir4465	TGTCGCCTTCTGATCCCGCTGCTG	AGCGGAAGACTAGGGCGACGACCA	tag218678
novel_sir4466	GTTGCCTCCTCACCTCTGCTG	ACGGAGGAGTGGAGACGACGC	tag251759
novel_sir4467	TGGCTATCGAAACTGGGTCTGCTG	CGATAGCTTTGACCCAGACGACCA	tag244455
novel_sir4468	CCGACTTTAGTCGGGTTGCTG	CTGAAATCAGCCCAACGACTT	tag94287
novel_sir4469	TCCTTTACAAGGTTCTCCAATCTG	GAAATGTTCCAAGAGGTTAGACCT	tag40210
novel_sir4470	GCCAAGTTCGACTAAGTTAGACAT	ATCGGTTCAAGCTGATTCAATCTG	tag165326
novel_sir4471	GGCGAACAAACAAATCAGAATCTG	GCTTGTTTGTTTAGTCTTAGACTT	tag215775
novel_sir4472	GCTTGTTTGTTTTGGCTTAGACTT	GACGAACAAACAAAACCGAATCTG	tag58021
novel_sir4473	AGCTAGCTATAAATCTGTAGACCG	TGTCGATCGATATTTAGACATCTG	tag116145
novel_sir4474	CGATGGCGACACCACTGTAGACAC	TGGCTACCGCTGTGGTGACATCTG	tag238890
novel_sir4475	AGCTATAGATACCCATGTAGACAT	TGTCGATATCTATGGGTACATCTG	tag25287
novel_sir4476	AGCTATAGATACCCATGTAGACAT	TCTCGATATCTATGGGTACATCTG	tag179604
novel_sir4477	GTCCGGCCGCCACTTCAGCATCTG	GGCCGGCGGTGAAGTCGTAGACGC	tag33603
novel_sir4478	TTCTTGCACATAGAAGAGCATCTG	GAACGTGTATCTTCTCGTAGACAC	tag169642
novel_sir4479	CGGTCAAGACTGCCACGTAGACAC	GAGCCAGTTCTGACGGTGCATCTG	tag205515
novel_sir4480	CGATTAATATCGCCACGTAGACGC	GAGCTAATTATAGCGGTGCATCTG	tag25616
novel_sir4481	CGGTCAATACCGCCACGTAGACGC	GGGCCAGTTATGGCGGTGCATCTG	tag207500
novel_sir4482	TTTGGAATGTGAGACCTAGATCTG	ACCTTACACTCTGGATCTAGACAC	tag13399
novel_sir4483	ACGGAAAAGTTTAGGCCTAGACTT	TTTGCCTTTTCAAATCCGGATCTG	tag125826
novel_sir4484	GGTGATCTGGGTATACACTATCTG	ACTAGACCCATATGTGATAGACAC	tag257125
novel_sir4485	AACTGTACGTTATTTGCCTATCTG	GACATGCAATAAACGGATAGACGC	tag19180
novel_sir4486	ACTGGACTCACATGTCATAGACAC	GGTGACCTGAGTGTACAGTATCTG	tag5001
novel_sir4487	GGTGACCTGAGTGTACAGTATCTG	ACTGGACTCACATGTCATAGACAA	tag5001

novel_sir4488	GGTGACCTGGGTGTACAGTATCTG	ACTGGACCCACATGTCATAGACAT	tag5831
novel_sir4489	GGTGACCTGGGTGTACAGTATCTG	ACTGGACCCACATGTCATAGACAC	tag5831
novel_sir4490	GGTGACTTGGGTGTACAGTATCTG	ACTGAACCCACATGTCATAGACAC	tag242331
novel_sir4491	ACCGTCGAAAGGATCCTGTATCTG	GCAGCTTTCCTAGGACATAGACTT	tag37371
novel_sir4492	GCTGCACGGGCAGCTGTCACTCTG	ACGTGCCCGTCGACAGTGAGACGC	tag16913
novel_sir4493	ATTCTACGCCGTACACCTCTG	AGATGCGGCATGTGGAGACAT	tag94249
novel_sir4494	AGATGGTCAAAACGCTGGAGACGA	TGTCTACCAGTTTTGCGACCTCTG	tag188074
novel_sir4495	TTGCTAACCTGCTGCCCTCTG	CGATTGGACGACGGGAGACTG	tag77397
novel_sir4496	ATGCGCACAACAGATCGGAGACCA	CGTACGCGTGTTGTCTAGCCTCTG	tag207495
novel_sir4497	CGGCCGCTCACCCCGCCTCTG	CGGCGAGTGGGGCGGAGACGG	tag86015
novel_sir4498	CCGGCGAGTGGGGCGGAGACGG	CCGGCCGCTCACCCCGCCTCTG	tag194734
novel_sir4499	CGCCGGACGCTCGCCGCCTCTG	GGCCTGCGAGCGGCGGAGACGT	tag114832
novel_sir4500	CCGCTGCAATCCTTCAGGCCTCTG	CGACGTTAGGAAGTCCGGAGACGC	tag199227
novel_sir4501	TGTTAATTTCAATCCGTGCCTCTG	AATTAAAGTTAGGCACGGAGACTC	tag253416
novel_sir4502	ACTTTTATCTACGTGTTGCCTCTG	AAAATAGATGCACAACGGAGACAC	tag221635
novel_sir4503	CATGATGTTTAGACCTGTCCTCTG	ACTACAAATCTGGACAGGAGACAT	tag152477
novel_sir4504	CGCTAGGAACCTGCTTCCGCTCTG	GATCCTTGGACGAAGGCGAGACGC	tag20000
novel_sir4505	TTGACTTTTCTGACTCTGGCTCTG	CTGAAAAGACTGAGACCGAGACTG	tag59544
novel_sir4506	GTCAGCTGACCCCTTGCTCTG	GTCGACTGGGGAACGAGACCT	tag129633
novel_sir4507	AGTCGACTGGGGAACGAGACCT	CGTCAGCTGACCCCTTGCTCTG	tag185399
novel_sir4508	GCAGTCGACTGGGGAACGAGACCT	TTCGTCAGCTGACCCCTTGCTCTG	tag1337
novel_sir4509	AGGGCTATGAATCTAGAGAGACAT	GTTCCCGATACTTAGATCTCTCTG	tag158808
novel_sir4510	GCCCGACTTAGGAAACGTCTG	GGCTGAATCCTTTGCAGACGA	tag131731
novel_sir4511	GCGGCAAGCTGACGTGGCAGACGG	CCCGCCGTTCGACTGCACCGTCTG	tag106176
novel_sir4512	CCGCTTTTACTGCACCGTCTG	CGAAAATGACGTGGCAGACGG	tag26019
novel_sir4513	CAGAACTAAGGATTGTGTCGTCTG	CTTGATTCCTAACACAGCAGACGC	tag20627
novel_sir4514	TCTCTAGTCTAACGACGATGTCTG	AGATCAGATTGCTGCTACAGACGC	tag54205
novel_sir4515	CACTATCACTTCGGTTCCTGTCTG	GATAGTGAAGCCAAGGACAGACGA	tag237493
novel_sir4516	CTCTAACTGCTTCAGTGGTGTCTG	GATTGACGAAGTCACCACAGACGC	tag79597
novel_sir4517	CATAAGAACCTTCTGACTTGTCTG	ATTCTTGGAAGACTGAACAGACAT	tag218213
novel_sir4518	CATGTACATGTGTCGCTCATTCTG	ACATGTACACAGCGAGTAAGACGG	tag49477
novel_sir4519	GCTGCAGTACGGACTCTAAGACAT	GTCGACGTCATGCCTGAGATTCTG	tag46772
novel_sir4520	GCTGCAGTACGGACTCTAAGACGC	GTCGACGTCATGCCTGAGATTCTG	tag46772
novel_sir4521	GCTGTAGCACGGACTCTAAGACGC	GTCGACATCGTGCCTGAGATTCTG	tag97481

novel_sir4522	GCTGTAGCACGGACTCTAAGACAC	GTCGACATCGTGCCTGAGATTCTG	tag97481
novel_sir4523	GTCGACGTCGTGCCTGAGATTCTG	GCTGCAGCACGGACTCTAAGACGC	tag198573
novel_sir4524	GTCGACGTCGTGCCTGAGATTCTG	GCTGCAGCACGGACTCTAAGACAT	tag198573
novel_sir4525	CACTAAATTTATCGCCCGATTCTG	GATTTAAATAGCGGGCTAAGACAT	tag193691
novel_sir4526	CCAGATTATATGCAACTAAGACGT	ATGGTCTAATATACGTTGATTCTG	tag18066
novel_sir4527	GGCTTCCAACAAAGGCATATTCTG	GAAGGTTGTTTCCGTATAAGACAT	tag215949
novel_sir4528	AGGCAAGCCGGTCGGTGAAGACGT	CTTCCGTTCGGCCAGCCACTTCTG	tag221040
novel_sir4529	CAATGCTACGTCAGATGAAGACAT	TAGTTACGATGCAGTCTACTTCTG	tag279262
novel_sir4530	GCCCGGCTAGGCCCGCCTTCTG	GGCCGATCCGGGCGGAAGACAT	tag75528
novel_sir4531	GGTGCCATATGCCTGCCTTCTG	ACGGTATACGGACGGAAGACGT	tag143921
novel_sir4532	TGTCACGACCATAGTGTCCTTCTG	AGTGCTGGTATCACAGGAAGACAG	tag24332
novel_sir4533	GTCAAAGTAGCATCTCGAAGACCG	ACCAGTTTCATCGTAGAGCTTCTG	tag244030
novel_sir4534	ACCAGTTATATCGTAGAGCTTCTG	GTCAATATAGCATCTCGAAGACCG	tag208445
novel_sir4535	AGGCCTGCCGAGTAGCGAAGACAA	GTTCCGGACGGCTCATCGCTTCTG	tag181657
novel_sir4536	GTTCCGGACGGCTCATCGCTTCTG	AGGCCTGCCGAGTAGCGAAGACGA	tag181657
novel_sir4537	TTCTGTTCAACGCTTCCTCTTCTG	GACAAGTTGCGAAGGAGAAGACGT	tag68986
novel_sir4538	GCTGTAGCACGGACTTCAAGACGC	GTCGACATCGTGCCTGAAGTTCTG	tag212429
novel_sir4539	GTCGACATCGTGCCTGAAGTTCTG	GCTGTAGCACGGACTTCAAGACAC	tag212429
novel_sir4540	TTCGACATCGTGCCTGAAGTTCTG	GCTGTAGCACGGACTTCAAGACAC	tag240480
novel_sir4541	GTCGACATCGTGCCTAAGGTTCTG	GCTGTAGCACGGATTCCAAGACGC	tag114378
novel_sir4542	GCTGCAGCATGGACTCCAAGACGT	GTCGACGTCGTACCTGAGGTTCTG	tag95342
novel_sir4543	GTCGACGTCGTACCTGAGGTTCTG	GCTGCAGCATGGACTCCAAGACAC	tag95342
novel_sir4544	GTCGACATCGAGCCTGAGGTTCTG	GCTGTAGCTCGGACTCCAAGACGC	tag60500
novel_sir4545	GTCGATATCATGCCTGAGGTTCTG	GCTATAGTACGGACTCCAAGACGC	tag48861
novel_sir4546	GCTGCAGTACGGACTCCAAGACGT	GTCGACGTCATGCCTGAGGTTCTG	tag186931
novel_sir4547	GTCGACGTCATGCCTGAGGTTCTG	GCTGCAGTACGGACTCCAAGACGC	tag186931
novel_sir4548	GACATCGTGCCTGAGGTTCTG	GTAGCACGGACTCCAAGACGC	tag21309
novel_sir4549	CTGTAGCACGGACTCCAAGACGC	TCGACATCGTGCCTGAGGTTCTG	tag19916
novel_sir4550	GTCGACATCGTGCCTGAGGTTCTG	GCTGTAGCACGGACTCCAAGACGC	tag42532
novel_sir4551	TTCGACATCGTGCCTGAGGTTCTG	GCTGTAGCACGGACTCCAAGACGC	tag52422
novel_sir4552	ATCGACATCGTGCCTGAGGTTCTG	GCTGTAGCACGGACTCCAAGACGC	tag160492
novel_sir4553	GTCTACATCGTGCCTGAGGTTCTG	GATGTAGCACGGACTCCAAGACGC	tag132446
novel_sir4554	GGCGATATCGTGCCTGAGGTTCTG	GCTATAGCACGGACTCCAAGACAC	tag27060
novel_sir4555	GTCGATATCGTGCCTGAGGTTCTG	GCTATAGCACGGACTCCAAGACAC	tag137874

novel_sir4556	ATCGATATCGTGCCTGAGGTTCTG	GCTATAGCACGGACTCCAAGACAC	tag159765
novel_sir4557	GCTATAGCACGGACTCCAAGACGC	GGCGATATCGTGCCTGAGGTTCTG	tag27060
novel_sir4558	GCTATAGCACGGACTCCAAGACGC	GTCGATATCGTGCCTGAGGTTCTG	tag137874
novel_sir4559	GCTGCAGCACGGACTCCAAGACAC	CTCGACGTCGTGCCTGAGGTTCTG	tag6704
novel_sir4560	GCTGCAGCACGGACTCCAAGACAC	ATCGACGTCGTGCCTGAGGTTCTG	tag169332
novel_sir4561	CTCGACGTCGTGCCTGAGGTTCTG	GCTGCAGCACGGACTCCAAGACGC	tag6704
novel_sir4562	GTCGACGTCGTGCCTGAGGTTCTG	GCTGCAGCACGGACTCCAAGACGC	tag100582
novel_sir4563	ATCGACGTCGTGCCTGAGGTTCTG	GCTGCAGCACGGACTCCAAGACGC	tag169332
novel_sir4564	GGCGACGTCGTGCCTGAGGTTCTG	GCTGCAGCACGGACTCCAAGACGC	tag172642
novel_sir4565	TTCGACGTCGTGCCTGAGGTTCTG	GCTGCAGCACGGACTCCAAGACGC	tag282566
novel_sir4566	GCTGCAGCACGGACTCCAAGACAT	CTCGACGTCGTGCCTGAGGTTCTG	tag6704
novel_sir4567	GCTGCAGCACGGACTCCAAGACAT	ATCGACGTCGTGCCTGAGGTTCTG	tag169332
novel_sir4568	GTCGATGTCGTGCCTGAGGTTCTG	GCTACAGCACGGACTCCAAGACGC	tag260239
novel_sir4569	GTCGACATTGTGCCTGAGGTTCTG	GCTGTAACACGGACTCCAAGACAC	tag172067
novel_sir4570	GTCGACATTGTGCCTGAGGTTCTG	GCTGTAACACGGACTCCAAGACGC	tag172067
novel_sir4571	GTCGACGTTGTGCCTGAGGTTCTG	GCTGCAACACGGACTCCAAGACAT	tag46925
novel_sir4572	GGCGACGTTGTGCCTGAGGTTCTG	GCTGCAACACGGACTCCAAGACAT	tag289225
novel_sir4573	GTCGACGTTGTGCCTGAGGTTCTG	GCTGCAACACGGACTCCAAGACGT	tag46925
novel_sir4574	GGCGACGTTGTGCCTGAGGTTCTG	GCTGCAACACGGACTCCAAGACGT	tag289225
novel_sir4575	GTCGACGTTGTGCCTGAGGTTCTG	GCTGCAACACGGACTCCAAGACAC	tag46925
novel_sir4576	GGCGACGTTGTGCCTGAGGTTCTG	GCTGCAACACGGACTCCAAGACAC	tag289225
novel_sir4577	GTCGACATCGTGTCTGAGGTTCTG	GCTGTAGCACAGACTCCAAGACAC	tag3768
novel_sir4578	GTCGACATCGTGTCTGAGGTTCTG	GCTGTAGCACAGACTCCAAGACGC	tag3768
novel_sir4579	GCTGCAACACAGACTCCAAGACGT	GTCGACGTTGTGTCTGAGGTTCTG	tag195527
novel_sir4580	GCTGTAGCACGAACTCCAAGACGC	GTCGACATCGTGCTTGAGGTTCTG	tag214882
novel_sir4581	ACTACAGCACGGACTTAAAGACGC	CATGATGTCGTGCCTGAATTTCTG	tag259762
novel_sir4582	GCTGTAGCACGAACTCAAAGACAC	GTCGACATCGTGCTTGAGTTTCTG	tag132816
novel_sir4583	ACTATTACCCGCTGGCAAAGACGC	GGTGATAATGGGCGACCGTTTCTG	tag20352
novel_sir4584	GGTGATAATGGGCGACCGTTTCTG	ACTATTACCCGCTGGCAAAGACGT	tag20352
novel_sir4585	CGATAGGCCACGCTTTCACGG	TAGCTATCCGGTGCGAAAGTG	tag7483
novel_sir4586	AGGCAACAACATCAGCAAGTG	CGTTGTTGTAGTCGTTCACGG	tag257336
novel_sir4587	CGTCCTACCACCTGAAGTTCACAG	GCGCAGGATGGTGGACTTCAAGTG	tag230711
novel_sir4588	CGCGATCCGAACACTTCACCG	CGGCGCTAGGCTTGTGAAGTG	tag214157
novel_sir4589	CCGCGATCCGAACACTTCACCG	GCGGCGCTAGGCTTGTGAAGTG	tag226882

novel_sir4590	GTCATGGAGCACGATGTCACAGTG	GTACCTCGTGCTACAGTGTCACTG	tag180764
novel_sir4591	TACTATGAGGGCCAGGGTACAGTG	GATACTCCCGGTCCCATGTCACCG	tag155109
novel_sir4592	TGCATTCGCGATGCAGTTACAGTG	GTAAGCGCTACGTCAATGTCACGT	tag220777
novel_sir4593	ACCTTGAGACTTAGTCGGTCACGA	TGTGGAACTCTGAATCAGCCAGTG	tag147958
novel_sir4594	TACATTCTCGATGCATCCGCAGTG	GTAAGAGCTACGTAGGCGTCACGT	tag132900
novel_sir4595	TGTTTGAAAGTTGAAAAGGCAGTG	AAACTTTCAACTTTTCCGTCACGT	tag270602
novel_sir4596	CAGTGCCTGGCGCCGTCACTC	AGGTCACGGACCGCGGCAGTG	tag9228
novel_sir4597	AGGGAGGGGTGCGCGGCAGTG	CCTCCCCACGCGCCGTCACCT	tag63636
novel_sir4598	CAGTCAAACTATAGCCCGTCACAG	TGGTCAGTTTGATATCGGGCAGTG	tag193763
novel_sir4599	ACAGCATTGGCTTTTAAGTCACCA	AATGTCGTAACCGAAAATTCAGTG	tag227793
novel_sir4600	AATTGACGACACGTCAATTCAGTG	AACTGCTGTGCAGTTAAGTCACTG	tag218425
novel_sir4601	AAGCAGTAAGCAAACCATTCAGTG	CGTCATTCGTTTGGTAAGTCACCA	tag96332
novel_sir4602	TGCGGCAACTGAAAAAGAGTG	GCCGTTGACTTTTTCTCACAT	tag49157
novel_sir4603	TACTGCGGCAACTGAAAAAGAGTG	GACGCCGTTGACTTTTTCTCACAT	tag217533
novel_sir4604	TACTGCGGCAACTGAAAAAGAGTG	GACGCCGTTGACTTTTTCTCACAA	tag217533
novel_sir4605	TACTGCGGCAACTGAAAAAGAGTG	GACGCCGTTGACTTTTTCTCACAC	tag217533
novel_sir4606	TATGCGCAAGAAACCCACAGAGTG	ACGCGTTCTTTGGGTGTCTCACAG	tag48644
novel_sir4607	GCGCCAGAACTCCTCTCACTG	CTCGCGGTCTTGAGGAGAGTG	tag192645
novel_sir4608	GACGTTTTCTGGCTCCGAGTG	GCAAAAGACCGAGGCTCACCG	tag242824
novel_sir4609	CCGCGCCGGCCCCAAGGAGTG	CGCGGCCGGGGTTCCTCACCT	tag57190
novel_sir4610	ACCAACCGGGGCTAAAGATCACCG	CGTGGTTGGCCCCGATTTCTAGTG	tag170541
novel_sir4611	ACGGAAACTAGCGACACATCACTG	TCTGCCTTTGATCGCTGTGTAGTG	tag26621
novel_sir4612	CATCTTAAGACCTCGTTTGCACCG	TGGTAGAATTCTGGAGCAAACGTG	tag62639
novel_sir4613	GGTAGGAAACCATAGGAACACGTG	ATCCTTTGGTATCCTTGTGCACTT	tag127846
novel_sir4614	CTGGCGGCGCCGGTAGACGTG	CCGCCGCGGCCATCTGCACGA	tag242445
novel_sir4615	GCCTCCTTCGCCGCCGACGTG	GAGGAAGCGGCGGCTGCACAG	tag218104
novel_sir4616	TTTGCCTGTCAGTTTGCAACCGTG	ACGGACAGTCAAACGTTGGCACGG	tag180442
novel_sir4617	ACTGCCTGTCAGTTTGCAACCGTG	ACGGACAGTCAAACGTTGGCACGG	tag218250
novel_sir4618	TCTGCCTGTCAGTTTGCAACCGTG	ACGGACAGTCAAACGTTGGCACGG	tag240669
novel_sir4619	ATCCTGAAAATCGAATACACCGTG	GGACTTTTAGCTTATGTGGCACTA	tag100961
novel_sir4620	CCGGCTTGGCCGGTGGCACGG	CCGGCCGAACCGGCCACCGTG	tag109836
novel_sir4621	TAGTAAAAAGTAGGATCCACCGTG	CATTTTTCATCCTAGGTGGCACCT	tag154054
novel_sir4622	TGGCACCAGAACGGATGCACCGTG	CGTGGTCTTGCCTACGTGGCACGT	tag206968
novel_sir4623	AGTTTATGGACCTAGATGGCACAT	TCTCAAATACCTGGATCTACCGTG	tag166387

novel_sir4624	ACGGACGATTAAAGTTGGGCACGG	TCTGCCTGCTAATTTCAACCCGTG	tag256656
novel_sir4625	TCTGCCTGCTAGTTTCAACCCGTG	ACGGACGATCAAAGTTGGGCACGG	tag230499
novel_sir4626	AACGGCTGTTGCAACGGGGCACGC	GCTTGCCGACAACGTTGCCCCGTG	tag153097
novel_sir4627	AGCTCCTCCGCATCGGCACCG	GCTCGAGGAGGCGTAGCCGTG	tag45366
novel_sir4628	TCTAGGTCGGGGCGCAGCGTG	ATCCAGCCCCGCGTCGCACGG	tag187250
novel_sir4629	CTCTAGGTCGGGGCGCAGCGTG	GATCCAGCCCCGCGTCGCACGG	tag190006
novel_sir4630	GACTCTAGGTCGGGGCGCAGCGTG	GAGATCCAGCCCCGCGTCGCACGG	tag110107
novel_sir4631	CGTAGGATAATGGTCGGTAGCGTG	ATCCTATTACCAGCCATCGCACGA	tag84716
novel_sir4632	GCGAGCTGAGGAGTACGCGTG	CTCGACTCCTCATGCGCACTC	tag151267
novel_sir4633	GGTTTTGGTCTACGTGGCGCACAC	CACCAAAACCAGATGCACCGCGTG	tag112082
novel_sir4634	ATGTCTCAAGTAGGCGTTCGCGTG	CAGAGTTCATCCGCAAGCGCACGG	tag280612
novel_sir4635	AATTAGCTCCAGGACACCGCACGA	CCTTAATCGAGGTCCTGTGGCGTG	tag131223
novel_sir4636	AACTAGGACTCGACGGTAGGTGCGTG	GATCCTGAGCTGCCATCCACGCACAA	tag178891
novel_sir4637	GACTAGGACTCGACGGTAGGTGCGTG	GATCCTGAGCTGCCATCCACGCACAA	tag207367
novel_sir4638	AACTGTGGCAACTGAAAAATCGTG	GACACCGTTGACTTTTTAGCACAA	tag11651
novel_sir4639	AACTGTGGCAACTGAAAAATCGTG	GACACCGTTGACTTTTTAGCACAT	tag11651
novel_sir4640	CTTTAGTCCCGGTTGGTAGCACCA	CTGAAATCAGGGCCAACCATCGTG	tag228299
novel_sir4641	GGCCTTCAATCCAAACTAGCACGT	AACCGGAAGTTAGGTTTGATCGTG	tag188135
novel_sir4642	CCGCCGCCGCGATGCCTCGTG	CGGCGGCGCTACGGAGCACGA	tag118939
novel_sir4643	ACCGCCGCCGCGATGCCTCGTG	GCGGCGGCGCTACGGAGCACGA	tag38787
novel_sir4644	AGCCTACGCTAGTATGGTCGTG	GGATGCGATCATACCAGCACTA	tag169015
novel_sir4645	AGCCTACGCTAGTATGGTCGTG	GGATGCGATCATACCAGCACTT	tag169015
novel_sir4646	CGCTAGTATGGTCGTGATTTCGTG	GATCATACCAGCACTAAAGCACCG	tag90838
novel_sir4647	TTGGGTCCGGTTGGTAAGGTG	CCCAGGCCAACCATTCCACAT	tag56988
novel_sir4648	GTTGCCGATTTCACACACCAGGTG	ACGGCTAAAGTGTGTGGTCCACTT	tag220133
novel_sir4649	CTAGAATAGTCCTTGGGTCCACCA	CAGATCTTATCAGGAACCCAGGTG	tag19955
novel_sir4650	CTAGAATAGTCCTTGGGTCCACCA	CGGATCTTATCAGGAACCCAGGTG	tag74036
novel_sir4651	AGTCTATGACATATGGGTCCACAT	TTTCAGATACTGTATACCCAGGTG	tag60613
novel_sir4652	AATTGTAACTGGGTCGGTCCACCC	GCTTAACATTGACCCAGCCAGGTG	tag283447
novel_sir4653	CCTCCAAAGTGAATCCAGGTG	AGGTTTCACTTAGGTCCACGA	tag33219
novel_sir4654	GCGGCGCTGCGGCGGCAGGTG	CCGCGACGCCGCCGTCCACCG	tag65163
novel_sir4655	AGTTCCGCGGCAAATAGTCCACGA	TGTCAAGGCGCCGTTTATCAGGTG	tag55013
novel_sir4656	GGTCCCAGAAGGCCGATCGAGGTG	AGGGTCTTCCGGCTAGCTCCACAA	tag255380
novel_sir4657	GGTCCCAGAAGGCCGATCGAGGTG	AGGGTCTTCCGGCTAGCTCCACGC	tag255380

novel_sir4658	CCGCGGGCCCGACTCGAGGTG	CGCCCGGGCTGAGCTCCACCG	tag212887
novel_sir4659	CGGAACGACGGTGCTAGGTG	CTTGCTGCCACGATCCACTG	tag33886
novel_sir4660	CCGGAACGACGGTGCTAGGTG	CCTTGCTGCCACGATCCACTT	tag142279
novel_sir4661	CCGGAACGACGGTGCTAGGTG	CCTTGCTGCCACGATCCACTG	tag142279
novel_sir4662	ACCGGAACGACGGTGCTAGGTG	GCCTTGCTGCCACGATCCACTG	tag277408
novel_sir4663	GATCCTGAGCTGCCATCCACGC	GACTAGGACTCGACGGTAGGTG	tag17846
novel_sir4664	GATCCTGAGCTGCCATCCACGC	AACTAGGACTCGACGGTAGGTG	tag263646
novel_sir4665	ACACTTTGCCACAGTTTGCCACGC	GGTGTGAAACGGTGTCAAACGGTG	tag114768
novel_sir4666	ACACTTTGCCACAGTTTGCCACGT	GGTGTGAAACGGTGTCAAACGGTG	tag114768
novel_sir4667	TCTGAGCCAGTTATAACGGTG	ACTCGGTCAATATTGCCACGT	tag188974
novel_sir4668	GACTCGGTCAATATTGCCACGT	TTCTGAGCCAGTTATAACGGTG	tag221496
novel_sir4669	AAGACTCGGTCAATATTGCCACGT	GCTTCTGAGCCAGTTATAACGGTG	tag27567
novel_sir4670	GCTTAAAAGAAGCAAGGCACGGTG	AATTTTCTTCGTTCCGTGCCACTC	tag235179
novel_sir4671	GGGGGTTCCCGGGCACGGTG	CCCAAGGGCCCGTGCCACCG	tag241968
novel_sir4672	CGGGGGTTCCCGGGCACGGTG	CCCCAAGGGCCCGTGCCACCG	tag115183
novel_sir4673	TCGGGGGTTCCCGGGCACGGTG	CCCCCAAGGGCCCGTGCCACCG	tag286251
novel_sir4674	AGCAAGGCCACTCTGCCACTT	CGTCGTTCCGGTGAGACGGTG	tag126592
novel_sir4675	CAGCAAGGCCACTCTGCCACTT	CCGTCGTTCCGGTGAGACGGTG	tag250919
novel_sir4676	CAGCAAGGCCACTCTGCCACTT	TCGTCGTTCCGGTGAGACGGTG	tag251221
novel_sir4677	GCTTCTGGTTCAGTTGTGACGGTG	AAGACCAAGTCAACACTGCCACGT	tag80243
novel_sir4678	ACTTCTGGTTCAGTTGTGACGGTG	AAGACCAAGTCAACACTGCCACGT	tag148482
novel_sir4679	TGCATTCGCGATGCAGTTACGGTG	GTAAGCGCTACGTCAATGCCACGT	tag220379
novel_sir4680	CTAAGCGCTACGTCAATGCCACGT	TGGATTCGCGATGCAGTTACGGTG	tag54759
novel_sir4681	TGCATTCGCGGTGCAGTTACGGTG	GTAAGCGCCACGTCAATGCCACGT	tag15587
novel_sir4682	GTAAACGCCACGTCAATGCCACGT	TGCATTTGCGGTGCAGTTACGGTG	tag159472
novel_sir4683	GATTCACACGGTCCTGGGCCACCC	TGCTAAGTGTGCCAGGACCCGGTG	tag137695
novel_sir4684	AGATACAATCTATGCGGGCCACCA	TGTCTATGTTAGATACGCCCGGTG	tag56276
novel_sir4685	TTTGACTAAAAGCGACTGCCGGTG	ACTGATTTTCGCTGACGGCCACAT	tag159939
novel_sir4686	AAGACCGAGTCAAATTCGCCACGT	ACTTCTGGCTCAGTTTAAGCGGTG	tag185331
novel_sir4687	AAGACTAAGTCAACATCGCCACGT	GCTTCTGATTCAGTTGTAGCGGTG	tag50201
novel_sir4688	AGTTTGTTCGGTGCATACGCGGTG	AAACAAGCCACGTATGCGCCACGT	tag128926
novel_sir4689	CCACCCTCTAGATCCGCCACTG	CTGGTGGGAGATCTAGGCGGTG	tag95720
novel_sir4690	ACCACCCTCTAGATCCGCCACTG	CCTGGTGGGAGATCTAGGCGGTG	tag40774
novel_sir4691	GACCACCCTCTAGATCCGCCACTG	GCCTGGTGGGAGATCTAGGCGGTG	tag293237

novel_sir4692	ACGAATGTTCTTAGGCCGCCACGT	GATGCTTACAAGAATCCGGCGGTG	tag53777
novel_sir4693	GTCCAAGCCACCGCCGCCACCT	TCCAGGTTCGGTGGCGGCGGTG	tag244718
novel_sir4694	ACTGCCACGCGTACGCCACGT	CCTGACGGTGCGCATGCGGTG	tag215069
novel_sir4695	AGCTTACAGCCGGCTTAGCCACAG	ATTCGAATGTCGGCCGAATCGGTG	tag107911
novel_sir4696	GCTTCTGGATCAGTTTCCTCGGTG	AAGACCTAGTCAAAGGAGCCACGT	tag170117
novel_sir4697	CTCGGCTCGCTCGATTGCTCGGTG	GCCGAGCGAGCTAACGAGCCACGA	tag93034
novel_sir4698	CTGCAGTAGTTGCCGTCGGTG	CGTCATCAACGGCAGCCACGG	tag147204
novel_sir4699	ACTGCAGTAGTTGCCGTCGGTG	ACGTCATCAACGGCAGCCACGG	tag101318
novel_sir4700	ACGAAACAACCGCGCCAGCCACAC	CGTGCTTTGTTGGCGCGGTCGGTG	tag79821
novel_sir4701	GCCGTCCGACGACACCAGCCACTC	CGCGGCAGGCTGCTGTGGTCGGTG	tag52009
novel_sir4702	GCCTCTGGATCAGTTTGGTCGGTG	GAGACCTAGTCAAACCAGCCACGT	tag35172
novel_sir4703	GAGACCTAGCCAAACCAGCCACGT	GCCTCTGGATCGGTTTGGTCGGTG	tag111131
novel_sir4704	AGACCTAGTCAAAAGAAGCCACGT	TTTCTGGATCAGTTTTCTTCGGTG	tag38508
novel_sir4705	GTCGGCGAGGTCCAAGCCACCG	GGCAGCCGCTCCAGGTTCGGTG	tag148600
novel_sir4706	CATAACTGGGCCAGAAGCAGGGTG	ATTGACCCGGTCTTCGTCCCACGT	tag47332
novel_sir4707	TTTCCATTTCGGCAACGGGTG	AGGTAAAGCCGTTGCCCACGC	tag24814
novel_sir4708	TTTTCCATTTCGGCAACGGGTG	AAGGTAAAGCCGTTGCCCACGC	tag103822
novel_sir4709	AAGACTTTCTAGCATTGCCCACAT	TATTCTGAAAGATCGTAACGGGTG	tag254867
novel_sir4710	AAGACTTTCTAGCATTGCCCACAA	TATTCTGAAAGATCGTAACGGGTG	tag254867
novel_sir4711	CGACGACGACGGTGGCCCACGC	CTGCTGCTGCTGCCACCGGGTG	tag81033
novel_sir4712	GAACCTAGGTCGTCTAGCCCACCT	AGCTTGGATCCAGCAGATCGGGTG	tag73897
novel_sir4713	GAACCCAGGTCGTCTAGCCCACCA	AGCTTGGGTCCAGCAGATCGGGTG	tag64208
novel_sir4714	GAACCCAGGTCGTCTAGCCCACCA	AACTTGGGTCCAGCAGATCGGGTG	tag173762
novel_sir4715	GTCGATGCTGAGCACAGCGGGGTG	GCTACGACTCGTGTCGCCCCACAC	tag188899
novel_sir4716	GTGTTTGATTCGGAATGGGTG	CAAACTAAGCCTTACCCACTA	tag35146
novel_sir4717	ATTCCACTTTACTATGACCCACAT	CCTAAGGTGAAATGATACTGGGTG	tag149498
novel_sir4718	TAGAGTTACTGTACATCCTGGGTG	CTCAATGACATGTAGGACCCACAT	tag151234
novel_sir4719	GCTGATTTAGAGCCGTCCTGGGTG	ACTAAATCTCGGCAGGACCCACCA	tag104851
novel_sir4720	ACTGATTTAGAGCCGTCCTGGGTG	ACTAAATCTCGGCAGGACCCACCA	tag215221
novel_sir4721	GACTAAGCTCGGCAGGACCCACCA	AGCTGATTCGAGCCGTCCTGGGTG	tag196562
novel_sir4722	GACTAAACTCGGCAGGACCCACCA	AACTGATTTGAGCCGTCCTGGGTG	tag172734
novel_sir4723	GGCGGCGGGCGGGGCTGGGTG	GCCGCCCGCCCCGACCCACGT	tag244920
novel_sir4724	GGGCGGCGGGCGGGGCTGGGTG	CGCCGCCCGCCCCGACCCACGT	tag167356
novel_sir4725	ACCATTGGAGCCAAATGACCACCT	ACTGGTAACCTCGGTTTACTGGTG	tag175160

novel_sir4726	ACGATTTTGCAGGCGGACCACAT	CATGCTAAAAACGTCCGCCTGGTG	tag270711
novel_sir4727	GGGCCGTCTCTCGCGCTGGTG	CGGCAGAGAGCGCGACCACGC	tag50065
novel_sir4728	TTGGGGCCGTCTCTCGCGCTGGTG	CCCCGGCAGAGAGCGCGACCACGC	tag143489
novel_sir4729	ATAAAGACTCGGTCAAGACCACCA	CCTATTTCTGAGCCAGTTCTGGTG	tag158216
novel_sir4730	CGCTAGCTAGCCGAAGTGGTG	GATCGATCGGCTTCACCACTT	tag24700
novel_sir4731	TAGGAGATGCCAGCTGACGTGGTG	CCTCTACGGTCGACTGCACCACGC	tag58966
novel_sir4732	CTGTCGAGCTTTTCTTCCGTGGTG	CAGCTCGAAAAGAAGGCACCACGT	tag47978
novel_sir4733	TTGTCGAGCTTTTCTTCCGTGGTG	CAGCTCGAAAAGAAGGCACCACGT	tag62387
novel_sir4734	ACCGTACTCAGCAAGCCACCACAG	GTTGGCATGAGTCGTTCGGTGGTG	tag43187
novel_sir4735	CGCCGGCGATCACCACCACGG	CTGCGGCCGCTAGTGGTGGTG	tag286075
novel_sir4736	CTGCGGTCGCTAGTGGTGGTG	CGCCAGCGATCACCACCACGG	tag31512
novel_sir4737	GCTCGAGAAGCAGCTGTGGTG	AGCTCTTCGTCGACACCACCC	tag232768
novel_sir4738	GATGAGAAGGTTACGTTTGTGGTG	ACTCTTCCAATGCAAACACCACTA	tag187394
novel_sir4739	TTCTGGCCTCGATAGTTGGTG	GACCGGAGCTATCAACCACTC	tag23221
novel_sir4740	GTTCTGGCCTCGATAGTTGGTG	AGACCGGAGCTATCAACCACTC	tag173319
novel_sir4741	CATGCGGTGCATCTGGTTTTGGTG	ACGCCACGTAGACCAAAACCACCG	tag21828
novel_sir4742	GGTGTCCTACGGCCAATTACACGT	GGCCACAGGATGCCGGTTAATGTG	tag62944
novel_sir4743	GTGTCCGGGAGCGCACATGTG	CAGGCCCTCGCGTGTACACAC	tag220002
novel_sir4744	GAGAAAAGCCACTTATACACCA	TTCTCTTTTCGGTGAATATGTG	tag278981
novel_sir4745	TAGTGTAGTTGGACAGTATATGTG	CACATCAACCTGTCATATACACAC	tag16542
novel_sir4746	TAGGATGGATGCTTGGCCTATGTG	CCTACCTACGAACCGGATACACGT	tag154956
novel_sir4747	CTAGGTCTCATCTGTCATACACAC	ATGATCCAGAGTAGACAGTATGTG	tag115992
novel_sir4748	ATGGACCAGGATGGACAGTATGTG	CCTGGTCCTACCTGTCATACACAC	tag28645
novel_sir4749	TGGACCAGGGTGGACAGTATGTG	CTGGTCCCACCTGTCATACACAC	tag230578
novel_sir4750	ATGGACCAGGGTGGACAGTATGTG	CCTGGTCCCACCTGTCATACACAC	tag148848
novel_sir4751	GGTTGGATCATGACCTACACTGTG	AACCTAGTACTGGATGTGACACAT	tag186371
novel_sir4752	GCTCAAATCCCTGGCTCTACTGTG	AGTTTAGGGACCGAGATGACACAG	tag187545
novel_sir4753	ACGGACAGTTAAAGTTGGACACGG	TCTGCCTGTCAATTTCAACCTGTG	tag150751
novel_sir4754	TCTGCTTGCTAATTTCAACCTGTG	ACGAACGATTAAAGTTGGACACGG	tag224275
novel_sir4755	TTTGCCTGTCAGTTTCAACCTGTG	ACGGACAGTCAAAGTTGGACACGT	tag5188
novel_sir4756	TCTGCCTGTCAGTTTCAACCTGTG	ACGGACAGTCAAAGTTGGACACGT	tag128121
novel_sir4757	ACTGCCTGTCAGTTTCAACCTGTG	ACGGACAGTCAAAGTTGGACACGT	tag148988
novel_sir4758	TTTGCCTGTCAGTTTCAACCTGTG	ACGGACAGTCAAAGTTGGACACGG	tag5188
novel_sir4759	TCTGCCTGTCAGTTTCAACCTGTG	ACGGACAGTCAAAGTTGGACACGG	tag128121

novel_sir4760	ACTGCCTGTCAGTTTCAACCTGTG	ACGGACAGTCAAAGTTGGACACGG	tag148988
novel_sir4761	ACGGGCGATCAAAGTTGGACACGG	TCTGCCCGCTAGTTTCAACCTGTG	tag244354
novel_sir4762	ATTCAAATATCGGCCGAACCTGTG	AGTTTATAGCCGGCTTGGACACAA	tag42776
novel_sir4763	ATTTGAATGTCGGCCGAACCTGTG	AACTTACAGCCGGCTTGGACACAA	tag126771
novel_sir4764	GTGGTAATTAGCTGCTGGACACTC	TTCACCATTAATCGACGACCTGTG	tag114467
novel_sir4765	GTGGTAATTAGCTGCTGGACACCC	TTCACCATTAATCGACGACCTGTG	tag114467
novel_sir4766	AACTAAGAGCACTCTCGGACACTT	GATTGATTCTCGTGAGAGCCTGTG	tag305
novel_sir4767	CATGCCTGCGCAACTGCGACACAC	TGGTACGGACGCGTTGACGCTGTG	tag159911
novel_sir4768	TCGAGGGCGGCGGCTGCTGTG	CTCCCGCCGCCGACGACACCC	tag222101
novel_sir4769	CTAAACGCCATATCAACGACACGT	TGGATTTGCGGTATAGTTGCTGTG	tag17661
novel_sir4770	ACCGATACCGATAGCTAGACACGT	TGTGGCTATGGCTATCGATCTGTG	tag162870
novel_sir4771	GCTCCAGCTCCTGCCTCTGTG	AGGTCGAGGACGGAGACACCG	tag210948
novel_sir4772	GGGGGAAACCGTAGTTCTGTG	CCCTTTGGCATCAAGACACCA	tag12710
novel_sir4773	AGCACGGACTCCAAGACACAG	CATCGTGCCTGAGGTTCTGTG	tag215940
novel_sir4774	CGTCGTGCCTGAGGTTCTGTG	AGCACGGACTCCAAGACACAA	tag163047
novel_sir4775	CATCGTGCCTGAGGTTCTGTG	AGCACGGACTCCAAGACACAA	tag215940
novel_sir4776	GACGTCGTGCCTGAGGTTCTGTG	GCAGCACGGACTCCAAGACACAG	tag269485
novel_sir4777	TCTTCGGATGTATGCGGTTCTGTG	AAGCCTACATACGCCAAGACACGC	tag118435
novel_sir4778	CTTCGAGTACCGTTCAGTGTG	AGCTCATGGCAAGTCACACAG	tag265537
novel_sir4779	AAGCTCATGGCAAGTCACACAG	CCTTCGAGTACCGTTCAGTGTG	tag224837
novel_sir4780	TTCTAATTCTAAAAGCACCGTGTG	GATTAAGATTTTCGTGGCACACTT	tag111286
novel_sir4781	CAGTCATAATCCGGCACACGG	AAGTCAGTATTAGGCCGTGTG	tag277332
novel_sir4782	AACAAGAACATAGCGCACACAG	CCTTGTTCTTGTATCGCGTGTG	tag68623
novel_sir4783	AACATGGCGGCGCCAGCCACACTG	GGTTGTACCGCCGCGGTCGGTGTG	tag71161
novel_sir4784	ACTGTATGAGTTGTTCGGTGTG	ACATACTCAACAAGCCACACAG	tag198308
novel_sir4785	TGTGTCCGGGAGCGCACATGTGTG	ACAGGCCCTCGCGTGTACACACCG	tag289503
novel_sir4786	TGTGTCCGGGAGTGCACATGTGTG	ACAGGCCCTCACGTGTACACACCG	tag96570
novel_sir4787	GCTTGACCATGAACAAACACACCC	TGCGAACTGGTACTTGTTTGTGTG	tag155199
novel_sir4788	AGCACGAATCTTGGAACACAT	CGTCGTGCTTAGAACCTTGTG	tag211264
novel_sir4789	GTCGCCGCGATCCGAACACTT	GGCAGCGGCGCTAGGCTTGTG	tag267373
novel_sir4790	CGTCGCCGCGATCCGAACACTT	GGGCAGCGGCGCTAGGCTTGTG	tag148906
novel_sir4791	CGGGCATAGCACTCAACACGC	GTGCCCGTATCGTGAGTTGTG	tag111401
novel_sir4792	GGCGACGCGGCGGCAACACCA	CGCCGCTGCGCCGCCGTTGTG	tag216677
novel_sir4793	AGGCTCGCGAGACAGGAAACACAC	CTTCCGAGCGCTCTGTCCTTTGTG	tag27085

novel_sir4794	CGGTACTCGAAGGCGTTTGTG	CATGAGCTTCCGCAAACACTT	tag199225
novel_sir4795	AATAAGACGAATGACCAAACACAA	AATTATTCTGCTTACTGGTTTGTG	tag160252
novel_sir4796	CATTTTGTCTCCTAACGTTTTGTG	AAAACAGAGGATTGCAAAACACAG	tag52184
novel_sir4797	TGTCGCTGTGCCAGAGGTTTTGTG	AGCGACACGGTCTCCAAAACACAA	tag267040
novel_sir4798	AATCGGTAGGATCATTTTTTTGTG	AGCCATCCTAGTAAAAAAACACCC	tag30904
novel_sir4799	ATTGCCATGTAGGACTTTTAACTT	TGTAACGGTACATCCTGAAAATTG	tag263223
novel_sir4800	TGTAACTGTACATCCTGAAAATTG	ATTGACATGTAGGACTTTTAACTT	tag128206
novel_sir4801	ATTTCCGTGCCCAGTGTTTAACTG	CCTAAAGGCACGGGTCACAAATTG	tag105012
novel_sir4802	CTCGATACCAAAAGGCGCAAATTG	GCTATGGTTTTCCGCGTTTAACTT	tag292370
novel_sir4803	CATAGTCTCAGACCGATCAAATTG	ATCAGAGTCTGGCTAGTTTAACAT	tag211888
novel_sir4804	GTCTTCGAAATGCTACTTTAACCA	GCCAGAAGCTTTACGATGAAATTG	tag253842
novel_sir4805	AGCGAATTTTCCAGGATTTAACGG	CCTCGCTTAAAAGGTCCTAAATTG	tag87385
novel_sir4806	GCTCCGGACCCTCTAGACGAATTG	AGGCCTGGGAGATCTGCTTAACTC	tag256626
novel_sir4807	CAGCTGGCTTAATGTGGGGAATTG	CGACCGAATTACACCCCTTAACAC	tag228240
novel_sir4808	ACTTGTGTCTAGATTCATTAACAT	TCTGAACACAGATCTAAGTAATTG	tag53284
novel_sir4809	GCCTTTTGCCTCGCCAGGTAATTG	GAAAACGGAGCGGTCCATTAACGC	tag77639
novel_sir4810	GCTGGCCGGCACTCAGCAACATTG	ACCGGCCGTGAGTCGTTGTAACTC	tag8284
novel_sir4811	GTCTTGGAGTCCGTGCTGTAACTG	CGCAGAACCTCAGGCACGACATTG	tag210145
novel_sir4812	ATCTTTAGTCCCGGTTGGTAACAC	TCTAGAAATCAGGGCCAACCATTG	tag149020
novel_sir4813	ATCTTTAGTCCCGGTTGGTAACAC	CCTAGAAATCAGGGCCAACCATTG	tag289880
novel_sir4814	GTCATCAGTGACGTGTCGTAACTA	CTCAGTAGTCACTGCACAGCATTG	tag207125
novel_sir4815	TCTTCGGCTGAATCCGTATCATTG	AAGCCGACTTAGGCATAGTAACCA	tag84270
novel_sir4816	ACCCTGGGTTTCCGTGTCTAACTT	TTTGGGACCCAAAGGCACAGATTG	tag143734
novel_sir4817	TTTGGGACTCAAAGGCACAGATTG	ACCCTGAGTTTCCGTGTCTAACTT	tag123398
novel_sir4818	CTTCGAATATAGACCGGCTAACCT	CTGAAGCTTATATCTGGCCGATTG	tag43410
novel_sir4819	CCCTTGTCCTCATCGCGATTG	GAACAGGAGTAGCGCTAACAG	tag77780
novel_sir4820	TCTGCACGCCGCGCGGATCGATTG	ACGTGCGGCGCGCCTAGCTAACCC	tag15583
novel_sir4821	ATTCAAACCCAGACTAGCTAACCC	CTTAAGTTTGGGTCTGATCGATTG	tag287915
novel_sir4822	CGTCTCCGGACTTCCTAACGT	CCGCAGAGGCCTGAAGGATTG	tag252042
novel_sir4823	CACCTCCGTGCGCACCTAACGG	GAGTGGAGGCACGCGTGGATTG	tag269205
novel_sir4824	CATGAACCGTACGCCGACTAACCC	TGGTACTTGGCATGCGGCTGATTG	tag51865
novel_sir4825	TTGTTACGATCTTTCAGAATATTG	CAATGCTAGAAAGTCTTATAACCT	tag23224
novel_sir4826	CCGTTACGATCTTTCAGAATATTG	CAATGCTAGAAAGTCTTATAACCT	tag35579
novel_sir4827	CTGTTACGATCTTTCAGAATATTG	CAATGCTAGAAAGTCTTATAACCT	tag129317

novel_sir4828	TCGTTACGATCTTTCAGAATATTG	CAATGCTAGAAAGTCTTATAACCT	tag152151
novel_sir4829	CGCGGCTTTCAACTATCACTATTG	GCCGAAAGTTGATAGTGATAACTC	tag144067
novel_sir4830	CCGACTAGTTATAGGGGATAACTT	TAGGCTGATCAATATCCCCTATTG	tag72188
novel_sir4831	AGCACGGACTCCAAGACATAACAT	CATCGTGCCTGAGGTTCTGTATTG	tag109003
novel_sir4832	CGCTGTGCCAGAGGTTTTGTATTG	GACACGGTCTCCAAAACATAACTT	tag184545
novel_sir4833	TGCTGTGCCAGAGGTTTTGTATTG	GACACGGTCTCCAAAACATAACTT	tag260422
novel_sir4834	ATCAGCCGAGGCACACAATAACAA	CTTAGTCGGCTCCGTGTGTTATTG	tag38582
novel_sir4835	ACTAGGCATCGGGGTAGGTTTATTG	ATCCGTAGCCCCATCCAAATAACTA	tag1366
novel_sir4836	ACTAGGCATCGGGGTAGGTTTATTG	ATCCGTAGCCCCATCCAAATAACTC	tag1366
novel_sir4837	ACTAGGCATCGGGGTAGGTTTATTG	ATCCGTAGCCCCATCCAAATAACTG	tag1366
novel_sir4838	CGCTTTCGCTGCACTGGTTTATTG	GAAAGCGACGTGACCAAATAACAC	tag292785
novel_sir4839	CGGCTCGACCTAAACTTG	CGAGCTGGATTTGAACCA	tag257928
novel_sir4840	GGTCCTCCTCCACGGCACTTG	AGGAGGAGGTGCCGTGAACGA	tag103632
novel_sir4841	TATAATAATTAGTTCCGGCACTTG	ATTATTAATCAAGGCCGTGAACCC	tag152051
novel_sir4842	GCAGTACCCGCTCCATGAACTT	ACCGTCATGGGCGAGGTACTTG	tag141374
novel_sir4843	GGTGCAGAAATAGGATCCACCTTG	ACGTCTTTATCCTAGGTGGAACAA	tag157984
novel_sir4844	AGGGCGATCCGCGGTGCTGCCTTG	CCGCTAGGCGCCACGACGGAACGG	tag89460
novel_sir4845	TGTTCGTCCGAATCTGTATCCTTG	AAGCAGGCTTAGACATAGGAACCA	tag264066
novel_sir4846	CAGGCCCTGACAGCATTCGAACCG	CTGTCCGGGACTGTCGTAAGCTTG	tag64128
novel_sir4847	CCTTAATTGGTCTCCATCGAACCG	TTGGAATTAACCAGAGGTAGCTTG	tag74226
novel_sir4848	ACTACATCAGCATAAATCGAACCT	TTTGATGTAGTCGTATTTAGCTTG	tag201953
novel_sir4849	AGCGTTGTCATCGAGCGCTTG	GCAACAGTAGCTCGCGAACCG	tag280042
novel_sir4850	TGTCATTCACCCAGTCGGCGCTTG	AGTAAGTGGGTCAGCCGCGAACCC	tag47943
novel_sir4851	AGCTATCGGACTATGAGCGAACGC	GTTCGATAGCCTGATACTCGCTTG	tag22740
novel_sir4852	GTCAATCCAAACGGTCGCTTG	GTTAGGTTTGCCAGCGAACTT	tag94683
novel_sir4853	CCGTCGCCGCGATCCGAACAC	GGGGCAGCGGCGCTAGGCTTG	tag59863
novel_sir4854	CCCGTCGCCGCGATCCGAACAC	GGGGGCAGCGGCGCTAGGCTTG	tag168425
novel_sir4855	ACCTCTGGGCGCAAGATGGCTTG	GAGACCCGCGTTCTACCGAACTG	tag139124
novel_sir4856	GACTAAAGGGGGGTTACGAACCG	CCCTGATTTCCCCCCAATGCTTG	tag154275
novel_sir4857	AAGAACCTAAATACTTTCTGCTTG	CTTGGATTTATGAAAGACGAACAA	tag1624
novel_sir4858	CACGATCGGCCGCGGTGCTTG	GCTAGCCGGCGCCACGAACTG	tag3988
novel_sir4859	TGTACGTGGGCCTATGACATCTTG	ATGCACCCGGATACTGTAGAACAC	tag238049
novel_sir4860	AGAAACTTGGCACCACTAGAACTC	TCTCTTTGAACCGTGGTGATCTTG	tag136503
novel_sir4861	GCGATGCCTCGTGCTCTCTTG	CTACGGAGCACGAGAGAACTC	tag136455

novel_sir4862	CTTGCACTACGGAGTAAAGTCTTG	ACGTGATGCCTCATTTCAGAACTG	tag244139
novel_sir4863	TCGTGGCCTAGGGTAGTCTTG	CACCGGATCCCATCAGAACTC	tag87423
novel_sir4864	TTCGTGGCCTAGGGTAGTCTTG	GCACCGGATCCCATCAGAACTC	tag26533
novel_sir4865	AGTGTTTTGATGTCTAAATTCTTG	ACAAAACTACAGATTTAAGAACTT	tag45554
novel_sir4866	CCCCTTAGGATGTCGATCTTCTTG	GGAATCCTACAGCTAGAAGAACAT	tag161812
novel_sir4867	ATGTCAGCCGAACCTACGTTCTTG	CAGTCGGCTTGGATGCAAGAACCA	tag66759
novel_sir4868	AGTGTACGGTGTACCTGGTTCTTG	ACATGCCACATGGACCAAGAACCA	tag283736
novel_sir4869	TACATCTTGTCCTTGTTCTTG	GTAGAACAGGAACAAGAACAT	tag33831
novel_sir4870	GCGCCACTGGCTTTTCAACCA	AGCGCGGTGACCGAAAAGTTG	tag69165
novel_sir4871	GGCAGTGTAGCAAAGTTAAAGTTG	GTCACATCGTTTCAATTTCAACCA	tag122466
novel_sir4872	GGCAGTGTAGCAAGGTTAAAGTTG	GTCACATCGTTCCAATTTCAACCA	tag225664
novel_sir4873	ATCACATCGTACCAATTTCAACCA	GATAGTGTAGCATGGTTAAAGTTG	tag181449
novel_sir4874	CATAGTCTCAGACCGATCAAGTTG	ATCAGAGTCTGGCTAGTTCAACAT	tag291247
novel_sir4875	AGCCCTACACCGCCTAAGTTG	GGGATGTGGCGGATTCAACGG	tag277373
novel_sir4876	GAGCCCTACACCGCCTAAGTTG	CGGGATGTGGCGGATTCAACGG	tag32276
novel_sir4877	CCCGATCTCTTGAGTGTACAGTTG	GCTAGAGAACTCACATGTCAACCA	tag66544
novel_sir4878	TGCACCCTGCTTCTGGCCCAGTTG	GTGGGACGAAGACCGGGTCAACAC	tag41562
novel_sir4879	ATTGGATAAAGACCGGGTCAACAC	TATAACCTATTTCTGGCCCAGTTG	tag280657
novel_sir4880	AGTCGTAGCTGAGTCGGTCAACCT	GCTCAGCATCGACTCAGCCAGTTG	tag63914
novel_sir4881	AGTTTGTACAAATTTTTCCAGTTG	AAACATGTTTAAAAAGGTCAACGG	tag234676
novel_sir4882	TAGTAATGCGGAGGTGGTCAGTTG	CATTACGCCTCCACCAGTCAACCT	tag172763
novel_sir4883	GTGGACCGAAGACTAAGTCAACAT	TGCACCTGGCTTCTGATTCAGTTG	tag139408
novel_sir4884	AGTTTGTACAAATCTTTTCAGTTG	AAACATGTTTAGAAAAGTCAACGG	tag253834
novel_sir4885	AGTTTGTACAGATCTTTTCAGTTG	AAACATGTCTAGAAAAGTCAACGG	tag101271
novel_sir4886	AAACATGTTTTAAAAAGTCAACGG	AGTTTGTACAAAATTTTTCAGTTG	tag59136
novel_sir4887	AGTTTGTATAAAATTTTTCAGTTG	AAACATATTTTAAAAAGTCAACGT	tag152463
novel_sir4888	AAACATATTTTAAAAAGTCAACGG	AGTTTGTATAAAATTTTTCAGTTG	tag152463
novel_sir4889	AGTTTGCAAACAATTTTTCAGTTG	AAACGTTTGTTAAAAAGTCAACGG	tag179235
novel_sir4890	GTTTGTACACGATTTTTCAGTTG	AACATGTGCTAAAAAGTCAACGG	tag240608
novel_sir4891	AGTTTGTACACGATTTTTCAGTTG	AAACATGTGCTAAAAAGTCAACGG	tag131745
novel_sir4892	AATTTGTACACGATTTTTCAGTTG	AAACATGTGCTAAAAAGTCAACGG	tag230352
novel_sir4893	AAACACGTGCTAAAAAGTCAACGG	AGTTTGTGCACGATTTTTCAGTTG	tag204923
novel_sir4894	GTTTGTATACGATTTTTCAGTTG	AACATATGCTAAAAAGTCAACGG	tag231552
novel_sir4895	AATTTGTATACGATTTTTCAGTTG	AAACATATGCTAAAAAGTCAACAG	tag153171

novel_sir4896	AGTTTGTATACGATTTTTCAGTTG	AAACATATGCTAAAAAGTCAACAG	tag167213
novel_sir4897	AATTTGTATACGATTTTTCAGTTG	AAACATATGCTAAAAAGTCAACGG	tag153171
novel_sir4898	AGTTTGTATACGATTTTTCAGTTG	AAACATATGCTAAAAAGTCAACGG	tag167213
novel_sir4899	AGTTTGTACATGATTTTTCAGTTG	AAACATGTACTAAAAAGTCAACGG	tag36305
novel_sir4900	AGTTTGTGCATGATTTTTCAGTTG	AAACACGTACTAAAAAGTCAACGG	tag249175
novel_sir4901	AAACATATACTAAAAAGTCAACGG	AGTTTGTATATGATTTTTCAGTTG	tag129157
novel_sir4902	AGTTTGTGTATGATTTTTCAGTTG	AAACACATACTAAAAAGTCAACGG	tag126587
novel_sir4903	AAACACAAACTAAAAAGTCAACGG	GGTTTGTGTTTGATTTTTCAGTTG	tag208919
novel_sir4904	AGTTTGTACACTATTTTTCAGTTG	AAACATGTGATAAAAAGTCAACGG	tag8507
novel_sir4905	AAACATGTGAGAAAAAGTCAACGG	AATTTGTACACTCTTTTTCAGTTG	tag224049
novel_sir4906	AGTTTGTACATTCTTTTTCAGTTG	AAACATGTAAGAAAAAGTCAACGG	tag17145
novel_sir4907	AGTTTATAAAAATTTTTTCAGTTG	AAATATTTTTAAAAAAGTCAACGG	tag66507
novel_sir4908	AAATATGTTTAAAAAAGTCAACGG	AGTTTATACAAATTTTTTCAGTTG	tag55902
novel_sir4909	AAACATGTTTAAAAAAGTCAACGA	AATTTGTACAAATTTTTTCAGTTG	tag172158
novel_sir4910	AAACATGTTTAAAAAAGTCAACGA	GGTTTGTACAAATTTTTTCAGTTG	tag252271
novel_sir4911	AATTTGTACAAATTTTTTCAGTTG	AAACATGTTTAAAAAAGTCAACAG	tag172158
novel_sir4912	AGTTTGTACAAATTTTTTCAGTTG	AAACATGTTTAAAAAAGTCAACAG	tag224450
novel_sir4913	GGTTTGTACAAATTTTTTCAGTTG	AAACATGTTTAAAAAAGTCAACAG	tag252271
novel_sir4914	AATTTGTACAAATTTTTTCAGTTG	AAACATGTTTAAAAAAGTCAACGG	tag172158
novel_sir4915	AGTTTGTACAAATTTTTTCAGTTG	AAACATGTTTAAAAAAGTCAACGG	tag224450
novel_sir4916	GGTTTGTACAAATTTTTTCAGTTG	AAACATGTTTAAAAAAGTCAACGG	tag252271
novel_sir4917	AGTTTGTATAAATTTTTTCAGTTG	AAACATATTTAAAAAAGTCAACGT	tag176898
novel_sir4918	AGTTTGTATAAATTTTTTCAGTTG	AAACATATTTAAAAAAGTCAACGG	tag176898
novel_sir4919	AAACATGTGTAAAAAAGTCAACGG	AGTTTGTACACATTTTTTCAGTTG	tag249164
novel_sir4920	ACCGTAAGTTTCCGCGCTCAACTT	GTTGGCATTCAAAGGCGCGAGTTG	tag280265
novel_sir4921	GCCGGTATTCAAAGGCGCGAGTTG	GCCATAAGTTTCCGCGCTCAACTT	tag5070
novel_sir4922	GTCGGTATTCAAAGGCGCGAGTTG	GCCATAAGTTTCCGCGCTCAACTT	tag131904
novel_sir4923	GTGAAGGCTTGGAAGGAGTTG	CTTCCGAACCTTCCTCAACGA	tag188752
novel_sir4924	CCTTAAATATACTATACTCAACAT	TGGGAATTTATATGATATGAGTTG	tag271070
novel_sir4925	TGCTAGACCCAAGTTTCATAGTTG	GATCTGGGTTCAAAGTATCAACCC	tag149016
novel_sir4926	ACCACCTAAACGCCATATCAACGA	TATGGTGGATTTGCGGTATAGTTG	tag32880
novel_sir4927	AATCTTGGAACACATCAACGG	GCTTAGAACCTTGTGTAGTTG	tag62047
novel_sir4928	GTGCTTAGAACCTTGTGTAGTTG	CGAATCTTGGAACACATCAACGG	tag245853
novel_sir4929	CGTGCTTAGAACCTTGTGTAGTTG	ACGAATCTTGGAACACATCAACGG	tag66491

novel_sir4930	CACTTAGGAGATTGCAGCACGTTG	GAATCCTCTAACGTCGTGCAACTT	tag76687
novel_sir4931	CGCAGAACCTCAGGCACGACGTTG	GTCTTGGAGTCCGTGCTGCAACTG	tag206712
novel_sir4932	ATGTCTAAACATCGGCCGACGTTG	CAGATTTGTAGCCGGCTGCAACAC	tag144022
novel_sir4933	ATGTCTAAATATCGGCCGACGTTG	CAGATTTATAGCCGGCTGCAACAC	tag285602
novel_sir4934	TGTAGAACCTCAGGCATGACGTTG	ATCTTGGAGTCCGTACTGCAACTG	tag88529
novel_sir4935	AGTCTAGCGCCCAATCCACCGTTG	AGATCGCGGGTTAGGTGGCAACAG	tag41540
novel_sir4936	ACGACGAGGATGGCAACGG	GCTGCTGCTCCTACCGTTG	tag117410
novel_sir4937	CTCCTGCTGCTCCTACCGTTG	GGACGACGAGGATGGCAACGG	tag74160
novel_sir4938	TGGTGTTCGGTCGCACCCGTTG	CACAAGCCAGCGTGGGCAACGG	tag20793
novel_sir4939	CAGGCACTGGAACGGCAACGG	CGGTCCGTGACCTTGCCGTTG	tag109536
novel_sir4940	GAGGCTAGTATTCGGAGGCAACGA	TACTCCGATCATAAGCCTCCGTTG	tag87461
novel_sir4941	AAACATATTTAAAAGAGGCAACGG	AGTTTGTATAAATTTTCTCCGTTG	tag192287
novel_sir4942	CCGTGCCAATTACAGAGCGTTG	CACGGTTAATGTCTCGCAACAG	tag102381
novel_sir4943	AGTAGACACTGCCCGGTTGCGTTG	ATCTGTGACGGGCCAACGCAACTC	tag92800
novel_sir4944	GTAGAACTACTGTGACAGCAACAC	GACATCTTGATGACACTGTCGTTG	tag108805
novel_sir4945	CGAAGGATCAAAAAGCAACGT	TAGCTTCCTAGTTTTTCGTTG	tag201632
novel_sir4946	ATCCAGAGCGGTTTTGTCCAACGT	GTTAGGTCTCGCCAAAACAGGTTG	tag280737
novel_sir4947	TTTGGGACTCAAAAGCACAGGTTG	ACCCTGAGTTTTCGTGTCCAACTT	tag53845
novel_sir4948	TTTGGGACCCAAACGCACAGGTTG	ACCCTGGGTTTGCGTGTCCAACTT	tag132841
novel_sir4949	TTTGGGACCCAAAGGCACAGGTTG	ACCCTGGGTTTCCGTGTCCAACTT	tag78834
novel_sir4950	ACCCTGAGTTTCCGTGTCCAACGT	TTTGGGACTCAAAGGCACAGGTTG	tag118994
novel_sir4951	TTTGGGACTCAAAGGCACAGGTTG	ACCCTGAGTTTCCGTGTCCAACTT	tag118994
novel_sir4952	GTCGGCACTTATAGGCACAGGTTG	GCCGTGAATATCCGTGTCCAACGT	tag8256
novel_sir4953	ATCGGCACTTATAGGCACAGGTTG	GCCGTGAATATCCGTGTCCAACGT	tag177894
novel_sir4954	GCCATAAGTTTCCACGTCCAACTT	GTCGGTATTCAAAGGTGCAGGTTG	tag245181
novel_sir4955	CCTTGGTGTGCTGACCGACGGTTG	AACCACACGACTGGCTGCCAACTC	tag25963
novel_sir4956	AATCTAGAACAGTTTGGCCAACAT	GGTTAGATCTTGTCAAACCGGTTG	tag78476
novel_sir4957	TTTGGGATACAAAAACACCGGTTG	ACCCTATGTTTTTGTGGCCAACGT	tag267795
novel_sir4958	GCTTTCTCCCGGTTGGGCCAACGT	TCCGAAAGAGGGCCAACCCGGTTG	tag68645
novel_sir4959	AAACTTAGGTCGTCTAGCCAACCA	AGTTTGAATCCAGCAGATCGGTTG	tag103365
novel_sir4960	GCCGTAGATATCTGTGCCCAACGT	GTCGGCATCTATAGACACGGGTTG	tag79669
novel_sir4961	GTCGGCACCTATAGGCACGGGTTG	GCCGTGGATATCCGTGCCCAACGT	tag178011
novel_sir4962	GCCGTAGATATCCGTGCCCAACGT	GTCGGCATCTATAGGCACGGGTTG	tag204408
novel_sir4963	TATGGTGAAAGTTGACTCGGGTTG	ACCACTTTCAACTGAGCCCAACCC	tag222182

novel_sir4964	TGTGAAGGATCGGTCTACTGGTTG	ACTTCCTAGCCAGATGACCAACTT	tag209166
novel_sir4965	CACTGGCTGGCGGCGATCTGGTTG	GACCGACCGCCGCTAGACCAACAA	tag133308
novel_sir4966	GACCGACCGCCACTAGACCAACAA	CACTGGCTGGCGGTGATCTGGTTG	tag287511
novel_sir4967	CCTAGAGAATCGCACCGGTGGTTG	ATCTCTTAGCGTGGCCACCAACGA	tag19197
novel_sir4968	GGGTTAGACCATGGCGGCATGTTG	CAATCTGGTACCGCCGTACAACGG	tag79727
novel_sir4969	GGGTTAGACCGTGGCGGCATGTTG	CAATCTGGCACCGCCGTACAACGG	tag76742
novel_sir4970	AAGTAGAATCGATTTGGCATGTTG	CATCTTAGCTAAACCGTACAACAA	tag141613
novel_sir4971	CATCGGGAGCTGCGCTAGATGTTG	AGCCCTCGACGCGATCTACAACTT	tag185057
novel_sir4972	CGCAGAACCTCAGGCACGATGTTG	GTCTTGGAGTCCGTGCTACAACTA	tag61727
novel_sir4973	GTCTTGGAGTCCGTGCTACAACTG	CGCAGAACCTCAGGCACGATGTTG	tag61727
novel_sir4974	CCTCGACGTGACCTACAACTT	CGGGAGCTGCACTGGATGTTG	tag77881
novel_sir4975	AGCCCTCGACGTGACCTACAACTT	CATCGGGAGCTGCACTGGATGTTG	tag208207
novel_sir4976	GTCTTGGAGTCCGTACTACAACTT	CGCAGAACCTCAGGCATGATGTTG	tag276933
novel_sir4977	GTCTTCAAGTCCGTACTACAACTG	CGCAGAAGTTCAGGCATGATGTTG	tag246279
novel_sir4978	CGCTGTGCCAGAGGTTTTATGTTG	GACACGGTCTCCAAAATACAACTT	tag111973
novel_sir4979	CACTGTGCCAGAGGTTTTATGTTG	GACACGGTCTCCAAAATACAACTT	tag139934
novel_sir4980	AGCTGTGCCAGAGGTTTTATGTTG	GACACGGTCTCCAAAATACAACTT	tag232850
novel_sir4981	CCTGACACAGCCTGACAACGT	CCGGACTGTGTCGGACTGTTG	tag229167
novel_sir4982	GCCTGACACAGCCTGACAACGT	CCCGGACTGTGTCGGACTGTTG	tag201414
novel_sir4983	GGGCCTGACACAGCCTGACAACGT	CTCCCGGACTGTGTCGGACTGTTG	tag215533
novel_sir4984	CGTCGTTGTGTGCCTCTACTGTTG	AGCAACACACGGAGATGACAACGC	tag98562
novel_sir4985	CACTTTTAAAGCGTCCGCCTGTTG	GAAAATTTCGCAGGCGGACAACAA	tag185146
novel_sir4986	AGTATTTTTACAGGCGGACAACAA	TTTCATAAAAATGTCCGCCTGTTG	tag256441
novel_sir4987	TCCACCCTGTGAAGGATCCTGTTG	GTGGGACACTTCCTAGGACAACGA	tag181414
novel_sir4988	TCTGCCGGGTCAAGGAAGCTGTTG	ACGGCCCAGTTCCTTCGACAACCC	tag19640
novel_sir4989	TGCTAGACTGAGCATCAGCTGTTG	GATCTGACTCGTAGTCGACAACAG	tag123142
novel_sir4990	GTCGCTGCGCCGGGTTCGCTGTTG	GCGACGCGGCCCAAGCGACAACAG	tag181814
novel_sir4991	TATCGATCGAACATCAGGCTGTTG	AGCTAGCTTGTAGTCCGACAACCT	tag53735
novel_sir4992	GGCTCCAAGTCATCTAGACAACGT	GACCGAGGTTCAGTAGATCTGTTG	tag142450
novel_sir4993	ACCTGTGACGAATCTAGACAACTC	GTTGGACACTGCTTAGATCTGTTG	tag259945
novel_sir4994	ATGTGACCCCTCCTAAGACAACGA	CCTACACTGGGGAGGATTCTGTTG	tag114745
novel_sir4995	TATAGGGATCAGGTTTCAGTGTTG	ATCCCTAGTCCAAAGTCACAACTC	tag181667
novel_sir4996	CAGCGGATGGTGACTTTCGTGTTG	CGCCTACCACTGAAAGCACAACGC	tag284520
novel_sir4997	GCTGTGCCAGAGGTTTTGTGTTG	ACACGGTCTCCAAAACACAACTT	tag174324

novel_sir4998	GACACGGTCTCCAAAACACAACTT	CACTGTGCCAGAGGTTTTGTGTTG	tag161322
novel_sir4999	GACACGGTCTCCAAAACACAACTT	TGCTGTGCCAGAGGTTTTGTGTTG	tag170479
novel_sir5000	GACACGGTCTCCAAAACACAACTT	AGCTGTGCCAGAGGTTTTGTGTTG	tag272186
novel_sir5001	TCTGTTGTAATAGCGTCATTGTTG	ACAACATTATCGCAGTAACAACGT	tag32430
novel_sir5002	CCTGTTGTAATAGCGTCATTGTTG	ACAACATTATCGCAGTAACAACGT	tag139359
novel_sir5003	GAGGCCCGTTTGAGATAACAACTA	AACTCCGGGCAAACTCTATTGTTG	tag75729
novel_sir5004	AACCTAAATACTTTCTGCTTGTTG	GGATTTATGAAAGACGAACAACTG	tag142412
novel_sir5005	TTGCCTGCAGAGCGATAGTTGTTG	CGGACGTCTCGCTATCAACAACGA	tag224005
novel_sir5006	AAGGCTATATGAATCACGTTGTTG	CCGATATACTTAGTGCAACAACGT	tag258417
novel_sir5007	GTGAACGACTACAACAACGG	GGCACTTGCTGATGTTGTTG	tag292598
novel_sir5008	CGTGAACGACTACAACAACGG	CGGCACTTGCTGATGTTGTTG	tag45996
novel_sir5009	CACACAGATCCCAAACAACCC	CAGTGTGTCTAGGGTTTGTTG	tag104671
novel_sir5010	AGCTACAGCAACTGAAAAAATTTG	GATGTCGTTGACTTTTTTAAACAT	tag245338
novel_sir5011	GACGCCGTTGACTTTTTTAAACAA	AACTGCGGCAACTGAAAAAATTTG	tag213676
novel_sir5012	AACTGCGGCAACTGAAAAAATTTG	GACGCCGTTGACTTTTTTAAACAC	tag213676
novel_sir5013	AACTGCGGCAACTGAAAAAATTTG	GACGCCGTTGACTTTTTTAAACAT	tag213676
novel_sir5014	ACAGCCTGACAACGTGTTAAACCA	TGTGTCGGACTGTTGCACAATTTG	tag154026
novel_sir5015	CACGGCTCGACAACAATTAAACCA	CTGTGCCGAGCTGTTGTTAATTTG	tag26750
novel_sir5016	TGCTATTTTCAACCGGTGCATTTG	GATAAAAGTTGGCCACGTAAACTC	tag61686
novel_sir5017	ATCGAATGAATCCCTGACGATTTG	GCTTACTTAGGGACTGCTAAACGC	tag43713
novel_sir5018	AGGGACTAAAGTGGGGCTAAACTT	CCTCCCTGATTTCACCCCGATTTG	tag122510
novel_sir5019	AGGGACTAAAGTAGGGCTAAACTT	CCTCCCTGATTTCATCCCGATTTG	tag211192
novel_sir5020	GTCATGAATTGGTAGGCTAAACAT	ATCAGTACTTAACCATCCGATTTG	tag13188
novel_sir5021	AGGGACTAAAGTGGAGCTAAACTT	CCTCCCTGATTTCACCTCGATTTG	tag125260
novel_sir5022	CAAACTTCCGTGGCCTAAACGG	GAGTTTGAAGGCACCGGATTTG	tag142609
novel_sir5023	TTCAGCTTTTCAGATCCGGATTTG	GTCGAAAAGTCTAGGCCTAAACTT	tag136968
novel_sir5024	GGGATTTGGGATTTGGGATTTG	CTAAACCCTAAACCCTAAACCC	tag88007
novel_sir5025	GACTTGTTCAACCTCCTGGATTTG	GAACAAGTTGGAGGACCTAAACGA	tag79038
novel_sir5026	GCGTGCGAAAAGTTTGATGATTTG	CACGCTTTTCAAACTACTAAACGG	tag4409
novel_sir5027	TCGTGCGAAAAGTTTGATGATTTG	CACGCTTTTCAAACTACTAAACGG	tag19988
novel_sir5028	ATCGGATGGTTAAGTACTGATTTG	GCCTACCAATTCATGACTAAACAT	tag26530
novel_sir5029	AATTACCGATGTGGGACTAAACCT	AGTTAATGGCTACACCCTGATTTG	tag99127
novel_sir5030	AATTACCGATGTGGGACTAAACCC	AGTTAATGGCTACACCCTGATTTG	tag99127
novel_sir5031	GCCGAAAAGTCTAAGACTAAACAA	TTCGGCTTTTCAGATTCTGATTTG	tag9564

novel_sir5032	AACACTGCCCGGCTTTCACCTTTG	GTGACGGGCCGAAAGTGGAAACCG	tag95196
novel_sir5033	CAATTATGGCCCGCCTGGAAACAA	CAGTTAATACCGGGCGGACCTTTG	tag154848
novel_sir5034	GTGGTTGGAAATGGTCGGAAACGG	TTCACCAACCTTTACCAGCCTTTG	tag154210
novel_sir5035	GATCAAAGTTGGGCGCGGAAACTC	TGCTAGTTTCAACCCGCGCCTTTG	tag13897
novel_sir5036	TGCTAATTTCAACCTGCGCCTTTG	GATTAAAGTTGGACGCGGAAACTC	tag173851
novel_sir5037	TGCTAGTTTCAACCTGCGCCTTTG	GATCAAAGTTGGACGCGGAAACTC	tag246313
novel_sir5038	GATCAAAGTTGGACGCGGAAACGT	TGCTAGTTTCAACCTGCGCCTTTG	tag246313
novel_sir5039	TGCTAGTTTTAATCTGCGCCTTTG	GATCAAAATTAGACGCGGAAACTC	tag126502
novel_sir5040	TGTCAGTTTGCAACCGTGCCTTTG	AGTCAAACGTTGGCACGGAAACCC	tag207129
novel_sir5041	GCCAGTTTGCAACCCGTGCCTTTG	GTCAAACGTTGGGCACGGAAACCA	tag149149
novel_sir5042	GCCAGTTTGCAACCTGTGCCTTTG	GTCAAACGTTGGACACGGAAACCA	tag51813
novel_sir5043	TGTCAGTTTCAACCTGTGCCTTTG	AGTCAAAGTTGGACACGGAAACCC	tag221068
novel_sir5044	AGTCAAAGTTGGACACGGAAACCT	TGTCAGTTTCAACCTGTGCCTTTG	tag221068
novel_sir5045	TGTTAGTTTCAACCTGTGCCTTTG	AATCAAAGTTGGACACGGAAACCC	tag92757
novel_sir5046	ATTAGCTATGGTCAGGGTCCTTTG	ATCGATACCAGTCCCAGGAAACAG	tag13734
novel_sir5047	TTCGACCAGATGCACCTCGCTTTG	GCTGGTCTACGTGGAGCGAAACGC	tag65014
novel_sir5048	ACGTATCCGTCGACAGCGAAACGC	GTTGCATAGGCAGCTGTCGCTTTG	tag79474
novel_sir5049	TACTATATGGCAGCTGTCGCTTTG	GATATACCGTCGACAGCGAAACGT	tag235745
novel_sir5050	CAGCGTGGCATTTGGACGAAACCC	GGGTCGCACCGTAAACCTGCTTTG	tag223880
novel_sir5051	TAGACCGAACGGACTGTATCTTTG	CTGGCTTGCCTGACATAGAAACCA	tag97175
novel_sir5052	GCGTAGTACTTACAGTCTTTG	CATCATGAATGTCAGAAACAG	tag110061
novel_sir5053	ATTTAGGTGACATGTAAGAAACGG	AGTAAATCCACTGTACATTCTTTG	tag252126
novel_sir5054	AGTAAATCCACCGTACGTTCTTTG	ATTTAGGTGGCATGCAAGAAACGG	tag33818
novel_sir5055	AGTAAATCTACTGTACGTTCTTTG	ATTTAGATGACATGCAAGAAACGG	tag23622
novel_sir5056	GGTAAATCTACTGTACGTTCTTTG	ATTTAGATGACATGCAAGAAACGG	tag176423
novel_sir5057	AGCAGGCTCGGACACAAGAAACTC	TGTCGTCCGAGCCTGTGTTCTTTG	tag101886
novel_sir5058	ACTCGTGGCACGCTTTTCAAACTA	TATGAGCACCGTGCGAAAAGTTTG	tag80094
novel_sir5059	TATGAGCACCGTGCGAAAAGTTTG	ACTCGTGGCACGCTTTTCAAACTG	tag80094
novel_sir5060	TATGAGCACCGTGCGAAAAGTTTG	ACTCGTGGCACGCTTTTCAAACCG	tag80094
novel_sir5061	AATTTTTTCAGTTGCCACAGTTTG	AAAAAAGTCAACGGTGTCAAACAT	tag73130
novel_sir5062	TAGAGAAGGCTGTAGACCAGTTTG	CTCTTCCGACATCTGGTCAAACGT	tag2941
novel_sir5063	TGGAGAAGGCTGTAGACCAGTTTG	CTCTTCCGACATCTGGTCAAACGT	tag266491
novel_sir5064	TAGAGAAGGCTGTAGACCAGTTTG	CTCTTCCGACATCTGGTCAAACAT	tag2941
novel_sir5065	TGGAGAAGGCTGTAGACCAGTTTG	CTCTTCCGACATCTGGTCAAACAT	tag266491

novel_sir5066	TCGGGAAGGCTGTAGACCAGTTTG	CCCTTCCGACATCTGGTCAAACGT	tag172403
novel_sir5067	TGGGGAAGGCTGTAGACCAGTTTG	CCCTTCCGACATCTGGTCAAACGT	tag203419
novel_sir5068	TGCATCCGTTGTGGTACCAGTTTG	GTAGGCAACACCATGGTCAAACCA	tag147522
novel_sir5069	TGGAGAAGGCTGTACGCCAGTTTG	CTCTTCCGACATGCGGTCAAACGT	tag172440
novel_sir5070	TGCAGTCAGTTTGGCGCCAGTTTG	GTCAGTCAAACCGCGGTCAAACCA	tag282743
novel_sir5071	CTCTTCCGATATCCGGTCAAACGT	TGGAGAAGGCTATAGGCCAGTTTG	tag165648
novel_sir5072	TGGGGAAGGCTATAGGCCAGTTTG	CCCTTCCGATATCCGGTCAAACGT	tag53837
novel_sir5073	TAGGGAAGGCTATAGGCCAGTTTG	CCCTTCCGATATCCGGTCAAACGT	tag221811
novel_sir5074	CCCTTTCGACATCCGGTCAAACGT	TGGGGAAAGCTGTAGGCCAGTTTG	tag179904
novel_sir5075	TGGGAAAGGCTGTAGGCCAGTTTG	CCTTTCCGACATCCGGTCAAACGT	tag64839
novel_sir5076	TGGAGAAGGCTGTAGGCCAGTTTG	CTCTTCCGACATCCGGTCAAACGT	tag91925
novel_sir5077	GGGGAAGGCTGTAGGCCAGTTTG	CCTTCCGACATCCGGTCAAACGT	tag19259
novel_sir5078	TGGGAAGGCTGTAGGCCAGTTTG	CCTTCCGACATCCGGTCAAACGT	tag31230
novel_sir5079	CCCTTCCGACATCCGGTCAAACAT	GGGGGAAGGCTGTAGGCCAGTTTG	tag10443
novel_sir5080	CCCTTCCGACATCCGGTCAAACAT	TAGGGAAGGCTGTAGGCCAGTTTG	tag12321
novel_sir5081	CCCTTCCGACATCCGGTCAAACGT	GGGGGAAGGCTGTAGGCCAGTTTG	tag10443
novel_sir5082	CCCTTCCGACATCCGGTCAAACGT	TAGGGAAGGCTGTAGGCCAGTTTG	tag12321
novel_sir5083	CCATTCCGACATCCGGTCAAACGT	TGGGTAAGGCTGTAGGCCAGTTTG	tag173668
novel_sir5084	ACCTTTCGACAACCGGTCAAACGT	TGTGGAAAGCTGTTGGCCAGTTTG	tag233981
novel_sir5085	AAATAAGACGGACGGTCAAACGT	AGTTTATTCTGCCTGCCAGTTTG	tag123083
novel_sir5086	ATTTTTTCAGTTGCCGCAGTTTG	AAAAAGTCAACGGCGTCAAACAT	tag81735
novel_sir5087	GATTTTTCAGTTGCCGCAGTTTG	AAAAAGTCAACGGCGTCAAACAT	tag128059
novel_sir5088	AATTTTTCAGTTGCCGCAGTTTG	AAAAAGTCAACGGCGTCAAACAT	tag238635
novel_sir5089	AAAAAAGTCAACGGCGTCAAACAT	AATTTTTTCAGTTGCCGCAGTTTG	tag85451
novel_sir5090	CTCTTCCGACATCCAGTCAAACGT	TGGAGAAGGCTGTAGGTCAGTTTG	tag146422
novel_sir5091	TGGGGAAGGCTGTAGGTCAGTTTG	CCCTTCCGACATCCAGTCAAACGT	tag221833
novel_sir5092	TGCCCGGATCAACAATAGAGTTTG	GGGCCTAGTTGTTATCTCAAACGG	tag212065
novel_sir5093	TGGGAAAGGCTGTAGGCTAGTTTG	CCTTTCCGACATCCGATCAAACGT	tag79742
novel_sir5094	GGGGAGTTGATTAAACGTTTG	CCTCAACTAATTTGCAAACGG	tag212000
novel_sir5095	TTCTCGATAGAGTTAGCCCGTTTG	GAGCTATCTCAATCGGGCAAACGG	tag84891
novel_sir5096	GGTTGAATGCTTTTGGCCCGTTTG	AACTTACGAAAACCGGGCAAACGT	tag153601
novel_sir5097	GGACTGAAGTTTGGATCCAAACTT	TTCCTGACTTCAAACCTAGGTTTG	tag260023
novel_sir5098	GGACTGAAGTTTGGATCCAAACAC	TTCCTGACTTCAAACCTAGGTTTG	tag260023
novel_sir5099	GGACTGAAGTTTGGATCCAAACTA	TTCCTGACTTCAAACCTAGGTTTG	tag260023

novel_sir5100	AACTTAATCAGATACCTAGGTTTG	GAATTAGTCTATGGATCCAAACAT	tag274388
novel_sir5101	AACTTAATCGGATACCTAGGTTTG	GAATTAGCCTATGGATCCAAACGT	tag15473
novel_sir5102	GAATTAGCCTATGGATCCAAACAT	AACTTAATCGGATACCTAGGTTTG	tag15473
novel_sir5103	TTTAATCGAGAATTCCTAGGTTTG	ATTAGCTCTTAAGGATCCAAACAG	tag148479
novel_sir5104	CTTAATCGGGAGTTCCTAGGTTTG	ATTAGCCCTCAAGGATCCAAACAG	tag262154
novel_sir5105	GATGATGACGCTAGGGCCAAACAA	TGCTACTACTGCGATCCCGGTTTG	tag202342
novel_sir5106	TGCTACTGCTGCGATCCCGGTTTG	GATGACGACGCTAGGGCCAAACAA	tag112250
novel_sir5107	CTCTTCCGACATCCGGCCAAACGT	TGGAGAAGGCTGTAGGCCGGTTTG	tag30633
novel_sir5108	GCTCTAGCTCCATCAGCCAAACAG	GTCGAGATCGAGGTAGTCGGTTTG	tag141396
novel_sir5109	TGGGGAAGGCTGTAGGCTGGTTTG	CCCTTCCGACATCCGACCAAACGT	tag259033
novel_sir5110	ATCGTTCTAATTTCAACCAAACTT	TGTAGCAAGATTAAAGTTGGTTTG	tag59511
novel_sir5111	TGTAGCAAGGTTAAAGTTGGTTTG	ATCGTTCCAATTTCAACCAAACTT	tag19914
novel_sir5112	GGTGTTGACACCGATGTTGGTTTG	ACAACTGTGGCTACAACCAAACAC	tag288440
novel_sir5113	AGACTTGTGTAGTAAACCAAACAC	CGTCTGAACACATCATTTGGTTTG	tag98497
novel_sir5114	TGTAGTCTCTATCGGATCTGTTTG	ATCAGAGATAGCCTAGACAAACCC	tag104265
novel_sir5115	ATTATTGTCTCGGATCACAAACCC	TGTAATAACAGAGCCTAGTGTTTG	tag65318
novel_sir5116	TCCTTAGTCGGCTCCATGTGTTTG	GAATCAGCCGAGGTACACAAACTG	tag47184
novel_sir5117	ACCAGACACATACCTAACAAACTG	TGTGGTCTGTGTATGGATTGTTTG	tag212004
novel_sir5118	GCTTTTCAGATTCGGATTTGTTTG	AAAAGTCTAAGCCTAAACAAACAA	tag176582
novel_sir5119	GCTTTTCAGATTCGGTTTTGTTTG	AAAAGTCTAAGCCAAAACAAACAA	tag57468
novel_sir5120	ACTTTTCAGATTCGGTTTTGTTTG	AAAAGTCTAAGCCAAAACAAACAA	tag218588
novel_sir5121	GTTTTTCAGATTCGGTTTTGTTTG	AAAAGTCTAAGCCAAAACAAACAA	tag236194
novel_sir5122	ACACCTCTGGCCAGAGTAAAACTA	CATGTGGAGACCGGTCTCATTTTG	tag43511
novel_sir5123	GAGAGGCAGCCGCGACTTATTTTG	CTCCGTCGGCGCTGAATAAAACGA	tag41904
novel_sir5124	GTTCGGTCTGATACTGCACTTTTG	AGCCAGACTATGACGTGAAAACCC	tag67064
novel_sir5125	GTCAAAGTTGGGCGCGGAAAACCA	GCCAGTTTCAACCCGCGCCTTTTG	tag60812
novel_sir5126	ACCAGTTTCAACCTGCGCCTTTTG	GTCAAAGTTGGACGCGGAAAACTA	tag168375
novel_sir5127	CCCGGTGACATTCGAAAACAA	GTGGGCCACTGTAAGCTTTTG	tag173106
novel_sir5128	GATCTAAGTTGAAGACGAAAACTT	TACTAGATTCAACTTCTGCTTTTG	tag151447
novel_sir5129	GTTCGACTCAGTACTGTGCTTTTG	AGCTGAGTCATGACACGAAAACCC	tag80402
novel_sir5130	GTCAAACGTTGGACACGAAAACCC	GCCAGTTTGCAACCTGTGCTTTTG	tag121580
novel_sir5131	GGTGTGATGCCCTGTTGTCTTTTG	ACACTACGGGACAACAGAAAACAT	tag210074
novel_sir5132	GACGGTCCCGCAGCTTCAAAACGC	CGCTGCCAGGGCGTCGAAGTTTTG	tag4824
novel_sir5133	CAGTAGGTGAGCCTCACAGTTTTG	CATCCACTCGGAGTGTCAAAACAG	tag216745

novel_sir5134	CACATTTTGTCTCCTAACGTTTTG	GTAAAACAGAGGATTGCAAAACAC	tag118932
novel_sir5135	CACATTTTGTCTCCTAACGTTTTG	GTAAAACAGAGGATTGCAAAACGC	tag118932
novel_sir5136	AGACCGTGTCGCTGTCCAAAACGC	CCTCTGGCACAGCGACAGGTTTTG	tag46082
novel_sir5137	CCTCTGGCACAGCGACAGGTTTTG	AGACCGTGTCGCTGTCCAAAACGA	tag46082
novel_sir5138	ACAGCGACACGGTCTCCAAAACAT	CCTGTCGCTGTGCCAGAGGTTTTG	tag16796
novel_sir5139	ACAGCGACACGGTCTCCAAAACAC	CCTGTCGCTGTGCCAGAGGTTTTG	tag16796
novel_sir5140	GTCGACGTCGTGCCTGAGGTTTTG	GCTGCAGCACGGACTCCAAAACAT	tag121625
novel_sir5141	TATAGGCTACACCGTGCGGTTTTG	ATCCGATGTGGCACGCCAAAACTT	tag93413
novel_sir5142	TATAGGCTACACTGTGCGGTTTTG	ATCCGATGTGACACGCCAAAACCT	tag156273
novel_sir5143	TATGGGCTACACTGTGCGGTTTTG	ACCCGATGTGACACGCCAAAACTT	tag108804
novel_sir5144	TATGGGCTATACTGTGCGGTTTTG	ACCCGATATGACACGCCAAAACTT	tag53044
novel_sir5145	TTCAGCTTTTCAGATTCGGTTTTG	GTCGAAAAGTCTAAGCCAAAACAA	tag149225
novel_sir5146	CACATGCGGTGCATCTGGTTTTG	GTACGCCACGTAGACCAAAACCA	tag217164
novel_sir5147	TGCGCTGCGGTGCATCTGGTTTTG	GCGACGCCACGTAGACCAAAACCA	tag178198
novel_sir5148	CCTGCGCACCGGTGTGAGTTTTTG	ACGCGTGGCCACACTCAAAAACAA	tag115082
novel_sir5149	ACGCGTGGCCACACTCAAAAACAG	CCTGCGCACCGGTGTGAGTTTTTG	tag115082
novel_sir5150	CCCTGGTTGGCTAGGCGGTTTTTG	GACCAACCGATCCGCCAAAAACTG	tag15873
novel_sir5151	GTTGCCGCAGTAGATAATTTTTTG	ACGGCGTCATCTATTAAAAAACGG	tag22605
novel_sir5152	ACTGTAACACGGACTAAAAAACAC	GTTGACATTGTGCCTGATTTTTTG	tag8003
novel_sir5153	GTTTGACACCGTTGACTTTTTAGC	TACAAACTGTGGCAACTGAAAAAT	tag68504
novel_sir5154	GTTTGACACCGTTGACTTTTTAGT	TACAAACTGTGGCAACTGAAAAAT	tag68504
novel_sir5155	ACTTGGATACATATGGGTTTTAAT	TGTGAACCTATGTATACCCAAAAT	tag245185
novel_sir5156	AGGGGTTATGGAATCGGTTTTATC	GATCCCCAATACCTTAGCCAAAAT	tag226519
novel_sir5157	CCTTTCTCTCCTACGGTAAAAT	AAAGAGAGGATGCCATTTTAGG	tag291443
novel_sir5158	AGGCCAACCATTCCACATTTTAGT	GGTCCGGTTGGTAAGGTGTAAAAT	tag258197
novel_sir5159	GGTAGGCACATGAAAACAAAT	ATCCGTGTACTTTTGTTTATA	tag18809
novel_sir5160	TTCTGCTTGCCAGTTTGTACAAAT	GACGAACGGTCAAACATGTTTAAA	tag57405
novel_sir5161	TACTGCTTGCCGGTTTGTACAAAT	GACGAACGGCCAAACATGTTTAAA	tag268068
novel_sir5162	GTTTAGGGTTTAGGGTTTAGG	CCCAAATCCCAAATCCCAAAT	tag2315
novel_sir5163	TTTGAACGTCTAGTGAGCAAAT	ACTTGCAGATCACTCGTTTAGG	tag113290
novel_sir5164	CACCCAAGTCACTGTTTAGCAAAT	GGGTTCAGTGACAAATCGTTTAGA	tag227066
novel_sir5165	GTTCGTGGCGTGGCTGCAAAT	AGCACCGCACCGACGTTTACA	tag93848
novel_sir5166	GACTTGCCAGCTAAACGCTCAAAT	GAACGGTCGATTTGCGAGTTTAGG	tag173387
novel_sir5167	GTCTAACTCGGCTCAAAT	GATTGAGCCGAGTTTATA	tag286901

novel_sir5168	GTCTAACTCGGCTCAAAT	GATTGAGCCGAGTTTAAG	tag286901
novel_sir5169	GTCTAACTCGGCTCAAAT	GATTGAGCCGAGTTTACA	tag286901
novel_sir5170	GTCTAACTCGGCTCAAAT	GATTGAGCCGAGTTTAAA	tag286901
novel_sir5171	GTCTAACTCGGCTCAAAT	GATTGAGCCGAGTTTAGA	tag286901
novel_sir5172	AGTCTAACTCGGCTCAAAT	AGATTGAGCCGAGTTTAAA	tag135386
novel_sir5173	AGTCTAACTCGGCTCAAAT	AGATTGAGCCGAGTTTATA	tag135386
novel_sir5174	AGTCTAACTCGGCTCAAAT	AGATTGAGCCGAGTTTACA	tag135386
novel_sir5175	AGTCTAACTCGGCTCAAAT	AGATTGAGCCGAGTTTAAC	tag135386
novel_sir5176	AGTCTAACTCGGCTCAAAT	AGATTGAGCCGAGTTTAAT	tag135386
novel_sir5177	AAGTCTAACTCGGCTCAAAT	CAGATTGAGCCGAGTTTAAA	tag147952
novel_sir5178	AAGTCTAACTCGGCTCAAAT	CAGATTGAGCCGAGTTTAAC	tag147952
novel_sir5179	CACAAGTTCGGCCGACATTCAAAT	GTTCAAGCCGGCTGTAAGTTTACA	tag223736
novel_sir5180	CACAGGTTCGGCCGACATTCAAAT	GTCCAAGCCGGCTGTAAGTTTACA	tag41406
novel_sir5181	CACAGGTTCGGCCGACATTCAAAT	GTCCAAGCCGGCTGTAAGTTTATA	tag41406
novel_sir5182	GACCAAGCCGGCTGTAAGTTTACA	CACTGGTTCGGCCGACATTCAAAT	tag240808
novel_sir5183	CACAGGTTCGGTCTACATTCAAAT	GTCCAAGCCAGATGTAAGTTTACA	tag90297
novel_sir5184	CACAGATTCGGCCGATATTCAAAT	GTCTAAGCCGGCTATAAGTTTATG	tag38956
novel_sir5185	CACAGATTCGGCCGATATTCAAAT	GTCTAAGCCGGCTATAAGTTTACA	tag38956
novel_sir5186	CCTACTGTGTCGCCATGTTCAAAT	ATGACACAGCGGTACAAGTTTAGG	tag62589
novel_sir5187	CGGTCGCACCCGTTGCCGAAAT	CAGCGTGGGCAACGGCTTTACC	tag29673
novel_sir5188	AGTAGGATCCACTGTCGAATAAAT	ATCCTAGGTGACAGCTTATTTAAT	tag215609
novel_sir5189	ATGCCCCGTCGCGTATTTAAG	GTTACGGGGCAGCGCATAAAT	tag88894
novel_sir5190	AATGCCCCGTCGCGTATTTAAG	TGTTACGGGGCAGCGCATAAAT	tag255198
novel_sir5191	TTTCAGTTGCCGCAGTTTATAAAT	AGTCAACGGCGTCAAATATTTAGG	tag267321
novel_sir5192	AGTCAACGGCGTCAAATATTTAGA	TTTCAGTTGCCGCAGTTTATAAAT	tag267321
novel_sir5193	GTTGCTGAATCCTCTCCTAAAT	ACGACTTAGGAGAGGATTTAAA	tag141462
novel_sir5194	CACCCAGGTCACTGTTTAGTAAAT	GGGTCCAGTGACAAATCATTTATC	tag73238
novel_sir5195	ATTATAGGCGTCGGTTAATTTAAA	TATAATATCCGCAGCCAATTAAAT	tag40635
novel_sir5196	CACCCAGGTCACCGTTTATTAAAT	GGGTCCAGTGGCAAATAATTTAGC	tag92373
novel_sir5197	ACATTGTTCACAAAATTTACA	ATTGTAACAAGTGTTTTAAAT	tag175306
novel_sir5198	GATGAGGACAAGGGTAACAAT	ACTCCTGTTCCCATTGTTAGC	tag250077
novel_sir5199	GCGGGCGGGGCTGGGTGCAAT	CCCGCCCCGACCCACGTTAGG	tag248359
novel_sir5200	CTTTTCCGTACTCAACTAGTTAAT	ACGAAAAGGCATGAGTTGATCAAT	tag106776
novel_sir5201	AGGGTAGTCTTGAGGCTTCAAT	CCATCAGAACTCCGAAGTTAAG	tag277785

novel_sir5202	ACATGTTTGATCGTTCGTCTTATT	TTTGTACAAACTAGCAAGCAGAAT	tag132457
novel_sir5203	ACATGTTTGACCGTTCGTCTTATT	CCTGTACAAACTGGCAAGCAGAAT	tag140599
novel_sir5204	ACATGTTTGACCGTTCGTCTTATT	TTTGTACAAACTGGCAAGCAGAAT	tag248077
novel_sir5205	CATGCACAAACTGGTAAGCAGAAT	ACGTGTTTGACCATTCGTCTTATT	tag97117
novel_sir5206	ACATGTTTGACCATTCGTCTTATT	AGTGTACAAACTGGTAAGCAGAAT	tag119083
novel_sir5207	CGGGCTGCGCATCCGAGGAGAAT	CCGACGCGTAGGCTCCTCTTATT	tag139993
novel_sir5208	CTAGATCCGCCACTGCTTACC	GAGATCTAGGCGGTGACGAAT	tag120875
novel_sir5209	CCTCTAGATCCGCCACTGCTTACC	TGGGAGATCTAGGCGGTGACGAAT	tag116452
novel_sir5210	GTCTTCGCTAGCAGTCGCCTTAAG	TCCAGAAGCGATCGTCAGCGGAAT	tag123786
novel_sir5211	CCCTTTACGATCTTACTGAAT	GAAATGCTAGAATGACTTACA	tag262585
novel_sir5212	CTTAGGAAACGTCTGCTGAAT	ATCCTTTGCAGACGACTTAAA	tag97590
novel_sir5213	TGTAAATTACTCGCGGTTCTGAAT	ATTTAATGAGCGCCAAGACTTATA	tag173277
novel_sir5214	AAGGCCACTCTGCCACTTACA	CGTTCCGGTGAGACGGTGAAT	tag33157
novel_sir5215	GTTTTCGCAGGCGGACCACTTAAG	GACAAAAGCGTCCGCCTGGTGAAT	tag276329
novel_sir5216	GATGAATCCGCCGAGTGTATTATT	CCCTACTTAGGCGGCTCACATAAT	tag31488
novel_sir5217	CCTCCGAGTTTGCACCAACCTAAT	AGGCTCAAACGTGGTTGGATTAGC	tag84707
novel_sir5218	TTCGATGGCACACGGCCTAAT	GCTACCGTGTGCCGGATTATG	tag263176
novel_sir5219	TGTAGAGTCGTTATCTGATCTAAT	ATCTCAGCAATAGACTAGATTAAT	tag144299
novel_sir5220	GTGCGTCCTCTCTTTGTAATTATT	TTCACGCAGGAGAGAAACATTAAT	tag211459
novel_sir5221	AATCTTTTGAGCCTAATTAGT	GCTTAGAAAACTCGGATTAAT	tag22325
novel_sir5222	AATCTTTTGAGCCTAATTAGT	GTTTAGAAAACTCGGATTAAT	tag58124
novel_sir5223	AGTCTACTTCTGGCTCAGTTTAAT	AGATGAAGACCGAGTCAAATTAGC	tag115417
novel_sir5224	CGAGCTCCGTTTGCAAATTAGT	GGGCTCGAGGCAAACGTTTAAT	tag26439
novel_sir5225	AGTTAGTAGTTGGTCCAAATTACA	GGTCAATCATCAACCAGGTTTAAT	tag95266
novel_sir5226	GATACGTTTGGCCTGTTTAAT	ATGCAAACCGGACAAATTACC	tag144783
novel_sir5227	ACGTCCGGCTAGCTTGTTTGTAGG	AGTGCAGGCCGATCGAACAAACAT	tag245499
novel_sir5228	TCTTAACGGAGGCAACAACAT	AATTGCCTCCGTTGTTGTAGT	tag83146
novel_sir5229	GTGTCTTGGAGTCCGTGTTGTAGC	GACACAGAACCTCAGGCACAACAT	tag181340
novel_sir5230	TGGTATAACAGAGATTGACAACAT	CATATTGTCTCTAACTGTTGTAGG	tag86169
novel_sir5231	ATCCTAGTCGGTTACGATTGTATT	TCTAGGATCAGCCAATGCTAACAT	tag116056
novel_sir5232	ATCCTAGTCGGTTACGATTGTATT	CCTAGGATCAGCCAATGCTAACAT	tag116791
novel_sir5233	TGGCGTTGGACATTAGTAACAT	CGCAACCTGTAATCATTGTAAC	tag287772
novel_sir5234	TATGCGCTGCCCCGTAACAT	ACGCGACGGGGCATTGTAAG	tag217119
novel_sir5235	TTTATGCGCTGCCCCGTAACAT	ATACGCGACGGGGCATTGTAAG	tag280407

novel_sir5236	CCTGAGGTTCTGCGTCACACACAT	ACTCCAAGACGCAGTGTGTGTATG	tag150074
novel_sir5237	ACTCCAAGACACAGTGTGTGTATG	CCTGAGGTTCTGTGTCACACACAT	tag64024
novel_sir5238	CCTGAGGTTCTGCATTACACACAT	ACTCCAAGACGTAATGTGTGTATG	tag220344
novel_sir5239	CCTGAGGTTCTGCGTTACACACAT	ACTCCAAGACGCAATGTGTGTATG	tag277225
novel_sir5240	ACTCCAAGACATAACATGTGTATG	CCTGAGGTTCTGTATTGTACACAT	tag174292
novel_sir5241	TACCCTGGTCTATAATTATCACAT	GGGACCAGATATTAATAGTGTAGT	tag47270
novel_sir5242	AGCCACACTTTTTTTTCTGTAGT	GTTCGGTGTGAAAAAAAAGACAT	tag141414
novel_sir5243	TGTAGGATCATGTTGCTTAGACAT	ATCCTAGTACAACGAATCTGTATA	tag262894
novel_sir5244	GATGCTAGCCGTGCAATCTGTACG	TACTACGATCGGCACGTTAGACAT	tag145584
novel_sir5245	AACACGGAAACTCAGGCACGACAT	GTGCCTTTGAGTCCGTGCTGTAGC	tag175728
novel_sir5246	AACGCAGAACCTCAGGCACGACAT	GCGTCTTGGAGTCCGTGCTGTAAC	tag94900
novel_sir5247	GACGCAGAACCTCAGGCACGACAT	GCGTCTTGGAGTCCGTGCTGTAAC	tag198194
novel_sir5248	TACGCAGAACCTCAGGCACGACAT	GCGTCTTGGAGTCCGTGCTGTAAC	tag269149
novel_sir5249	AATGCAGAACCTCAGGCACGACAT	ACGTCTTGGAGTCCGTGCTGTAGC	tag264480
novel_sir5250	AATGCAGAACCTCAGGCACGACAT	ACGTCTTGGAGTCCGTGCTGTAAT	tag264480
novel_sir5251	AACACAGAACTTCAGGCACGACAT	GTGTCTTGAAGTCCGTGCTGTAGC	tag116785
novel_sir5252	GCGTCTTAGAGTCCATGCTGTAGC	GACGCAGAATCTCAGGTACGACAT	tag183208
novel_sir5253	CCGCACCAGCGACAGCGACAT	CGTGGTCGCTGTCGCTGTACC	tag94770
novel_sir5254	AACACAAAACCTCAGGCGCGACAT	GTGTTTTGGAGTCCGCGCTGTAGC	tag212842
novel_sir5255	AGAAAGTGCAAGCCCATGTATA	ATTCTTTCACGTTCGGGTACAT	tag167176
novel_sir5256	ACGTTTATTTTACCGACATGTAAA	TGTGCAAATAAAATGGCTGTACAT	tag151042
novel_sir5257	TCTGCTTACCAGTTTGTACAT	ACGAATGGTCAAACATGTAAG	tag159127
novel_sir5258	TTCTAAGCACGACGTTTTACAT	GATTCGTGCTGCAAAATGTACC	tag105833
novel_sir5259	GGTTCTAAGCACGACGTTTTACAT	AAGATTCGTGCTGCAAAATGTACC	tag9157
novel_sir5260	GTGCGACCGAACACCAACCAT	CGCTGGCTTGTGGTTGGTACG	tag96801
novel_sir5261	GATCTTTAGTCCCGGTTGGTAAC	AGCTAGAAATCAGGGCCAACCAT	tag208308
novel_sir5262	CTTCCCTACATCGCGTCGAACCAT	AGGGATGTAGCGCAGCTTGGTAGA	tag29744
novel_sir5263	TTTCCCTACATCGCGTCGAACCAT	AGGGATGTAGCGCAGCTTGGTAGA	tag248995
novel_sir5264	ATTCCCTACATCGCGTCGAACCAT	AGGGATGTAGCGCAGCTTGGTAGA	tag279372
novel_sir5265	CTTCCCTACATCGCGTCGAACCAT	AGGGATGTAGCGCAGCTTGGTAAC	tag29744
novel_sir5266	TTTCCCTACATCGCGTCGAACCAT	AGGGATGTAGCGCAGCTTGGTAAC	tag248995
novel_sir5267	ATTCCCTACATCGCGTCGAACCAT	AGGGATGTAGCGCAGCTTGGTAAC	tag279372
novel_sir5268	AGGGATGTAGCGCAGCTTGGTAGT	ATTCCCTACATCGCGTCGAACCAT	tag279372
novel_sir5269	CTTCCCTACATCGCGTCGAACCAT	AGGGATGTAGCGCAGCTTGGTAGC	tag29744

novel_sir5270	TTTCCCTACATCGCGTCGAACCAT	AGGGATGTAGCGCAGCTTGGTAGC	tag248995
novel_sir5271	ATTCCCTACATCGCGTCGAACCAT	AGGGATGTAGCGCAGCTTGGTAGC	tag279372
novel_sir5272	CGCTAGAAATCAGGGCCTAACCAT	GATCTTTAGTCCCGGATTGGTAGT	tag99017
novel_sir5273	CACAGAACACCCATGTCACCAT	GTCTTGTGGGTACAGTGGTACA	tag222750
novel_sir5274	GTTCCGTGTGAAGCAGCCCAT	AGGCACACTTCGTCGGGTACA	tag137402
novel_sir5275	CGTTCCGTGTGAAGCAGCCCAT	AAGGCACACTTCGTCGGGTACA	tag9188
novel_sir5276	ATTTCCGTGTGAAGCAGCCCAT	AAGGCACACTTCGTCGGGTACA	tag109594
novel_sir5277	AGGGCTATAGCTCAGTTCGGTAGA	ATTCCCGATATCGAGTCAAGCCAT	tag168096
novel_sir5278	AGGGCTATAGCTCAGTTCGGTAGA	GTTCCCGATATCGAGTCAAGCCAT	tag191718
novel_sir5279	ATAGGCGTCTGGAGAATCGGTATC	CGTATCCGCAGACCTCTTAGCCAT	tag289311
novel_sir5280	GGTCCTTAAACTTGTAGCGGTATG	CGCCAGGAATTTGAACATCGCCAT	tag170105
novel_sir5281	TGTGCCAATAATTCTTTCATCCAT	ACGGTTATTAAGAAAGTAGGTAGA	tag172057
novel_sir5282	CTTTGCCAAGTCGGCCTCCAT	AACGGTTCAGCCGGAGGTAGG	tag242085
novel_sir5283	GACGTGCCGCCGAATGTTCTCCAT	GCACGGCGGCTTACAAGAGGTATA	tag43873
novel_sir5284	GGCGTGCCGCCGAATGTTCTCCAT	GCACGGCGGCTTACAAGAGGTATA	tag117118
novel_sir5285	TGCAGCTGTCACCGCGCGGTCCAT	GTCGACAGTGGCGCGCCAGGTAGG	tag73179
novel_sir5286	AGGCCGGCGCCGGGCAGTGTCCAT	CGGCCGCGGCCCGTCACAGGTAAC	tag35661
novel_sir5287	TATAGCCAGACGTACCTTCCAT	ATCGGTCTGCATGGAAGGTAGC	tag192304
novel_sir5288	CGCTAGAAATCAGGGCCTAAGCAT	GATCTTTAGTCCCGGATTCGTAGT	tag197982
novel_sir5289	TGTCTGTCGAATAGATCTAAGCAT	AGACAGCTTATCTAGATTCGTAGT	tag266299
novel_sir5290	GCTACGTCAGGAGCTACAGCAT	ATGCAGTCCTCGATGTCGTAGG	tag220110
novel_sir5291	CAGCTACGTCAGGAGCTACAGCAT	CGATGCAGTCCTCGATGTCGTAGG	tag188460
novel_sir5292	TTTTTCAGTTGCCGCAGCAT	AAAGTCAACGGCGTCGTACT	tag46635
novel_sir5293	TTTTTCAGTTGCCGCAGCAT	AAAGTCAACGGCGTCGTACA	tag46635
novel_sir5294	ATTTTTCAGTTGCCGCAGCAT	AAAAGTCAACGGCGTCGTACA	tag166129
novel_sir5295	TAGATGTTAGACTTACCGGAGCAT	CTACAATCTGAATGGCCTCGTAAT	tag55680
novel_sir5296	AAGACCACAGGATCCGCAT	CTGGTGTCCTAGGCGTAGA	tag67716
novel_sir5297	TATAAGACCACAGGATCCGCAT	ATTCTGGTGTCCTAGGCGTAGG	tag285492
novel_sir5298	TATAAGACCACAGGATCCGCAT	ATTCTGGTGTCCTAGGCGTAGA	tag285492
novel_sir5299	CTCCTCTCTACCGGCTCGCCAAGTTCC	GGAGAGATGGCCGAGCGGTTCAAGGC	tag291606
novel_sir5300	TACAATGCCCCGTCGCGTATT	GAATGTTACGGGGCAGCGCAT	tag264759
novel_sir5301	CTTCGTCGCCGGCCGCCGTAGC	GGGAAGCAGCGGCCGGCGGCAT	tag159057
novel_sir5302	CAATTCGTACTTTGCTGGGGGCAT	TAAGCATGAAACGACCCCCGTACA	tag94450
novel_sir5303	ATCACTAAGTCGTCGACGTAGA	ACTAGTGATTCAGCAGCTGCAT	tag120147

novel_sir5304	GCTTTCAGTGGTAGGCGACGTACC	CACGAAAGTCACCATCCGCTGCAT	tag241763
novel_sir5305	GACGTCGTGCCTGAGGTTCTGCAT	GCAGCACGGACTCCAAGACGTAAT	tag154992
novel_sir5306	TGCTTAGATCTATCCGCAGTGCAT	GAATCTAGATAGGCGTCACGTACC	tag148980
novel_sir5307	TCTGAGCCAGTTATAACGGTGCAT	ACTCGGTCAATATTGCCACGTAGG	tag291204
novel_sir5308	GAATGGTCAAACCTTGAACGTAAA	TGCTTACCAGTTTGGAACTTGCAT	tag97169
novel_sir5309	TACCGTACGTACACCTCAAATCAT	GGCATGCATGTGGAGTTTAGTACC	tag216949
novel_sir5310	TCTGGTCTATAATTATCACATCAT	ACCAGATATTAATAGTGTAGTATG	tag114964
novel_sir5311	TGGTAGGAATGGGCAGTACATCAT	CATCCTTACCCGTCATGTAGTAAT	tag108830
novel_sir5312	GCCCCTATCAAGATTCGTAGTACT	TACGGGGATAGTTCTAAGCATCAT	tag127432
novel_sir5313	GCTATCATTAAGTTGGATCAT	ATAGTAATTCAACCTAGTACG	tag182491
novel_sir5314	TGTAAGTACTACGCGTATCAT	ATTCATGATGCGCATAGTATG	tag292762
novel_sir5315	AACAATGGGAACAGGAGTAGC	GATTGTTACCCTTGTCCTCAT	tag71855
novel_sir5316	TTCGCACGAACCCGCTCTCAT	GCGTGCTTGGGCGAGAGTAGT	tag4126
novel_sir5317	CACATACTGTCTATCCTGGTTCAT	GTATGACAGATAGGACCAAGTATC	tag267268
novel_sir5318	TGCTCACCGGTTTGTCATGTTCAT	GAGTGGCCAAACAGTACAAGTAAA	tag67845
novel_sir5319	AACCTGTGCCAATAATTCTTTCAT	GGACACGGTTATTAAGAAAGTAGG	tag134999
novel_sir5320	TGTGTATTTGAAGCTTGAAAAGAT	ACATAAACTTCGAACTTTTCTATC	tag145646
novel_sir5321	AAGTGTTACATTCTGAAAGAT	CACAATGTAAGACTTTCTAGC	tag194101
novel_sir5322	ATGTTCTTGTTCCTGTTCTACA	GATACAAGAACAAGGACAAGAT	tag49341
novel_sir5323	AGTGTTACATTCAGTAAGAT	ACAATGTAAGTCATTCTAGC	tag213333
novel_sir5324	AGACCAAGGATAAGTAATTCTACT	TGTCTGGTTCCTATTCATTAAGAT	tag283355
novel_sir5325	GGGGTAGTCTACAGACGGTCTAGT	CTCCCCATCAGATGTCTGCCAGAT	tag100259
novel_sir5326	CTCCGAAGCTTGGATCCAGCAGAT	GGCTTCGAACCTAGGTCGTCTAGC	tag31636
novel_sir5327	CTCAGCCAGCCGAAAGTCTAGT	GAGAGTCGGTCGGCTTTCAGAT	tag262595
novel_sir5328	CCTGAGAGCTGGTGCCAGAGAT	ACTCTCGACCACGGTCTCTACT	tag75545
novel_sir5329	ACTTAATAGCATCATTCCTCTAAA	TGTGAATTATCGTAGTAAGGAGAT	tag227721
novel_sir5330	GCCGTAGCCGATAGTGACTCTAAG	TACGGCATCGGCTATCACTGAGAT	tag227796
novel_sir5331	CTCTCCAGCCGGCCGGATAGAT	GAGGTCGGCCGGCCTATCTAAG	tag93957
novel_sir5332	AGCATCTCCAACAGCATATCTATC	TCTCGTAGAGGTTGTCGTATAGAT	tag75073
novel_sir5333	AAAAAGTCAACGGCGTCATCTATT	TCTTTTTCAGTTGCCGCAGTAGAT	tag222799
novel_sir5334	AGTAATCTGACTCAATAATCTAGA	TATCATTAGACTGAGTTATTAGAT	tag271163
novel_sir5335	GGTTAAGTAGATATCGGTTAGAT	AATTCATCTATAGCCAATCTAAT	tag585
novel_sir5336	GTTGCAGCCGGCTATAAATCTATA	CACAACGTCGGCCGATATTTAGAT	tag190004
novel_sir5337	CAGATTAGGCCAGGATCCAACGAT	CTAATCCGGTCCTAGGTTGCTATG	tag229318

novel_sir5338	GGCCAGAGCGGACTTGCTATC	TCCCGGTCTCGCCTGAACGAT	tag33677
novel_sir5339	GTCCCGGTCTCGCCTGAACGAT	GGGCCAGAGCGGACTTGCTATC	tag271735
novel_sir5340	AGGGCCAGAGCGGACTTGCTATC	GGTCCCGGTCTCGCCTGAACGAT	tag234456
novel_sir5341	GTGACGCAGAACCTCAGGCACGAT	CTGCGTCTTGGAGTCCGTGCTACA	tag13221
novel_sir5342	CTCATCCTGCCAAGTGCTAGC	AGGAGTAGGACGGTTCACGAT	tag118473
novel_sir5343	CGCCTGAAGAATTCCGCTGACGAT	GGACTTCTTAAGGCGACTGCTAGC	tag25744
novel_sir5344	GGTGACAACTTATTTAATGCTATG	ATCCACTGTTGAATAAATTACGAT	tag169202
novel_sir5345	GCGAAGTAGGGCCAATGCTATC	TTCGCTTCATCCCGGTTACGAT	tag200906
novel_sir5346	CCTATGACGGAGGCGAGCCGAT	ATACTGCCTCCGCTCGGCTATC	tag20254
novel_sir5347	TCCCTATGACGGAGGCGAGCCGAT	GGATACTGCCTCCGCTCGGCTATC	tag265615
novel_sir5348	GGGTATTGGCGCCCGTGCCGAT	CATAACCGCGGGCACGGCTATT	tag163136
novel_sir5349	ATCAAAAAGCAACGTCGCTATG	CCTAGTTTTTCGTTGCAGCGAT	tag40119
novel_sir5350	ACTTTTAGGTCTCGTGCGCTACC	GGTGAAAATCCAGAGCACGCGAT	tag109735
novel_sir5351	AGGTGAAAATCCAGAGCACGCGAT	CACTTTTAGGTCTCGTGCGCTACC	tag213277
novel_sir5352	GTCGCCGGCCGCCGTAGCTAGC	AGCAGCGGCCGGCGGCATCGAT	tag271777
novel_sir5353	GAGCGGCTGCCGCCGATCGAT	CGCCGACGGCGGCTAGCTACG	tag229524
novel_sir5354	CTGTTTGGATCCTTAAGAGCTAAT	TGGACAAACCTAGGAATTCTCGAT	tag144430
novel_sir5355	CAGAACCTCAGGCACGACGTCGAT	CTTGGAGTCCGTGCTGCAGCTAGC	tag138706
novel_sir5356	GGCAAGGCTCCGGCAGCTATG	CTCCGTTCCGAGGCCGTCGAT	tag59617
novel_sir5357	CTTGGAGTCCGTGCTACAGCTAGC	CAGAACCTCAGGCACGATGTCGAT	tag125472
novel_sir5358	ACTGCTGAGTTACCGCCCTTCGAT	ACGACTCAATGGCGGGAAGCTATC	tag104518
novel_sir5359	TTTTCGGTCACCGCGCTTCGAT	AAGCCAGTGGCGCGAAGCTACC	tag192905
novel_sir5360	AGATTCAGTCTTAGGTTCGAT	TAAGTCAGAATCCAAGCTAGC	tag165809
novel_sir5361	GAGATTCAGTCTTAGGTTCGAT	CTAAGTCAGAATCCAAGCTAGC	tag292420
novel_sir5362	CGGAGATTCAGTCTTAGGTTCGAT	CTCTAAGTCAGAATCCAAGCTAGC	tag226747
novel_sir5363	CTGACCAGCAGACCCAGTTTCGAT	CTGGTCGTCTGGGTCAAAGCTATC	tag292658
novel_sir5364	CAGCCAAGACTTATTCCTACA	TGGTCGGTTCTGAATAAGGAT	tag252241
novel_sir5365	ACTTTTTTTTTCTGTAGTCCTACA	TGTGAAAAAAAAAGACATCAGGAT	tag247109
novel_sir5366	GATATCAGGTCGGATAGTCCTATG	GACTATAGTCCAGCCTATCAGGAT	tag248848
novel_sir5367	GTGTACCCATTCGGCTAGGAT	CATGGGTAAGCCGATCCTAAG	tag73577
novel_sir5368	ACTTTCTTGTTTCATGGGCCTACA	TGTGAAAGAACAAAGTACCCGGAT	tag75233
novel_sir5369	CACTTGAGCGTGCGGAGTCCGGAT	GAACTCGCACGCCTCAGGCCTACA	tag283115
novel_sir5370	GGGGCACCCAGTACCGCGGAT	CCGTGGGTCATGGCGCCTAGA	tag5500
novel_sir5371	TAGGTAGAGAGATAAATATCGGAT	CCATCTCTCTATTTATAGCCTAAG	tag247302

novel_sir5372	CCAACGTCGACTATAACCCCTAGA	TTGGTTGCAGCTGATATTGGGGAT	tag94104
novel_sir5373	CCAACGTCGACTATAACCCCTAGC	TTGGTTGCAGCTGATATTGGGGAT	tag94104
novel_sir5374	CGCGAAACGTTCAAGCACCTGGAT	GCTTTGCAAGTTCGTGGACCTAAG	tag33433
novel_sir5375	GATAAACCGCATAAACCACCTACC	GGCTATTTGGCGTATTTGGTGGAT	tag190181
novel_sir5376	GTGACGTACGCGTGGCAACCTAGT	TGCACTGCATGCGCACCGTTGGAT	tag286756
novel_sir5377	TAAGTTCGCTGGCAAACCTAAC	AAATTCAAGCGACCGTTTGGAT	tag138866
novel_sir5378	TACAGATCTAAGTATCATAATGAT	GTCTAGATTCATAGTATTACTATG	tag105245
novel_sir5379	GTTGTCCAGATATGTCGTACTAGG	TGCAACAGGTCTATACAGCATGAT	tag204621
novel_sir5380	GTGACGCAGAACCTCAGGCATGAT	CTGCGTCTTGGAGTCCGTACTACA	tag287378
novel_sir5381	GAGGTCACCCATCCTAGTACTACT	TCCTCCAGTGGGTAGGATCATGAT	tag236489
novel_sir5382	GGAGAGATGGCTGAGTGGACTAAC	CCCCTCTCTACCGACTCACCTGAT	tag178895
novel_sir5383	GGAGAGATGGCTGAGTGGACTAAA	CCCCTCTCTACCGACTCACCTGAT	tag178895
novel_sir5384	CACACACATACTGTCCACCCTGAT	GTGTGTATGACAGGTGGGACTAGA	tag174963
novel_sir5385	GCGTCAAGCACCGCGGCTGAT	CAGTTCGTGGCGCCGACTAGC	tag154914
novel_sir5386	GAGGTGCCGTGAACGACTACA	TCCTCCACGGCACTTGCTGAT	tag163020
novel_sir5387	CGCTCGAGGCCGCGGCGTGAT	GAGCTCCGGCGCCGCACTAGG	tag6943
novel_sir5388	GCTCCTGACGTAGCTGGTGAT	AGGACTGCATCGACCACTACG	tag274758
novel_sir5389	CGGCGCCGGGCAGTGTCCATTGAT	CGCGGCCCGTCACAGGTAACTAGT	tag59679
novel_sir5390	TAGGATGGGTTGGAAACTATG	CGATCCTACCCAACCTTTGAT	tag20065
novel_sir5391	TGTGGAGACCGGTCTCATTTTGAT	ACCTCTGGCCAGAGTAAAACTATT	tag231228
novel_sir5392	CCTGTAATTTAGGTGGCCGAATAT	ACATTAAATCCACCGGCTTATAAA	tag12480
novel_sir5393	CCTGAGGTTCTGCGTCACACATAT	ACTCCAAGACGCAGTGTGTATATG	tag174224
novel_sir5394	CCTGAGGTTCTGCATTACACATAT	ACTCCAAGACGTAATGTGTATATG	tag120176
novel_sir5395	TACACAGAACCTCAGGCACGATAT	GTGTCTTGGAGTCCGTGCTATAGC	tag83221
novel_sir5396	GACACAGAACCTCAGGCACGATAT	GTGTCTTGGAGTCCGTGCTATAGC	tag137551
novel_sir5397	AACACAGAACCTCAGGCACGATAT	GTGTCTTGGAGTCCGTGCTATAGC	tag151726
novel_sir5398	GCGTCTTGGAGTCCGTGCTATAGC	GACGCAGAACCTCAGGCACGATAT	tag289886
novel_sir5399	GTGTCTTAGAGTCCGTGCTATAGC	GACACAGAATCTCAGGCACGATAT	tag175500
novel_sir5400	GACGCAGAACCTCAGGCATGATAT	GCGTCTTGGAGTCCGTACTATAGC	tag90381
novel_sir5401	GGCGCAGAACCTCAGGCATGATAT	GCGTCTTGGAGTCCGTACTATAGC	tag91848
novel_sir5402	GAATCGTGCCAAACAGACTATAAG	ACCTTAGCACGGTTTGTCTGATAT	tag112160
novel_sir5403	GCAATCTGCGCGGGCCACTATACT	CGCGTTAGACGCGCCCGGTGATAT	tag248643
novel_sir5404	GTGTCACATCGGGTATTATATAGG	CGCACAGTGTAGCCCATAATATAT	tag154107
novel_sir5405	TGCCGCCGAATGTTCTCCATATAT	GGCGGCTTACAAGAGGTATATATA	tag173708

novel_sir5406	TGGTATAACCGTACCTGCCTATAT	CATATTGGCATGGACGGATATACC	tag269926
novel_sir5407	TAGCATTGCCCACATTCATATAGA	AGATCGTAACGGGTGTAAGTATAT	tag233946
novel_sir5408	CACTGATCGTCAGCTTGAGTATAT	GACTAGCAGTCGAACTCATATAGG	tag137762
novel_sir5409	TGTGAACCGTGAATTCATGTATAT	ACTTGGCACTTAAGTACATATATT	tag245516
novel_sir5410	CACTCGCGATCTATGACATATAGG	CGGTGAGCGCTAGATACTGTATAT	tag3183
novel_sir5411	CGTAACAAACGTGGCCAAAACTAT	ATTGTTTGCACCGGTTTTGATAGT	tag285637
novel_sir5412	CAGAAGGCGACAACAAGAGACTAT	CTTCCGCTGTTGTTCTCTGATAGT	tag167825
novel_sir5413	TAGCGCCTGCCGTGGTTATACTAT	CGCGGACGGCACCAATATGATATT	tag9460
novel_sir5414	GCTTTGGACCGGGATGATACA	ACCGAAACCTGGCCCTACTAT	tag27032
novel_sir5415	GACGGGTTCGGAGATCAGTACTAT	GCCCAAGCCTCTAGTCATGATACT	tag140624
novel_sir5416	AGTTAAATACTAGACGCGGATATC	TGTCAATTTATGATCTGCGCCTAT	tag53355
novel_sir5417	TGCCAGTTTGCAACCCATGCCTAT	GGTCAAACGTTGGGTACGGATATC	tag8175
novel_sir5418	GTCAAAGCGCTAAATACGGATATT	ACCAGTTTCGCGATTTATGCCTAT	tag117219
novel_sir5419	TGCCAGTTTGCAACCCGTGCCTAT	GGTCAAACGTTGGGCACGGATATC	tag65446
novel_sir5420	GGTCAAACGTTGGACACGGATATC	TGCCAGTTTGCAACCTGTGCCTAT	tag8462
novel_sir5421	AGTCAAACGTTGGACACGGATATT	TGTCAGTTTGCAACCTGTGCCTAT	tag148280
novel_sir5422	TGCCAATTTGTAATCTGTGCCTAT	GGTTAAACATTAGACACGGATATC	tag81550
novel_sir5423	CACTTCCGGACACACGTATCCTAT	GAAGGCCTGTGTGCATAGGATACG	tag214504
novel_sir5424	CCCCATTAAGCCAGCTGGCGCTAT	GGTAATTCGGTCGACCGCGATAGT	tag81535
novel_sir5425	CACAGAAAGTCGGCAAAGTGCTAT	GTCTTTCAGCCGTTTCACGATATT	tag243313
novel_sir5426	ACCCAAGCCTCTAGTCACGATACC	GATGGGTTCGGAGATCAGTGCTAT	tag117882
novel_sir5427	ACTGCAGCGCTCGAGTTGCTAT	ACGTCGCGAGCTCAACGATACT	tag289731
novel_sir5428	TACTCCTAGGCGTACAGTATCTAT	GAGGATCCGCATGTCATAGATAGC	tag61714
novel_sir5429	GCATCAAATCCGGGCTAACTCTAT	TAGTTTAGGCCCGATTGAGATAAC	tag282514
novel_sir5430	TGCCAGTTTGCAACCCGTGTCTAT	GGTCAAACGTTGGGCACAGATATC	tag34907
novel_sir5431	GTTGGCCTTGATTTCTAATTCTAT	ACCGGAACTAAAGATTAAGATAAT	tag128633
novel_sir5432	GACACTGGACCCACATTTCATAGA	TACTGTGACCTGGGTGTAAAGTAT	tag16117
novel_sir5433	AAAAGTCAACGGCGTCATACA	TTTTTTCAGTTGCCGCAGTAT	tag62654
novel_sir5434	AAAAGTCAACGGCGTCATACA	ATTTTTCAGTTGCCGCAGTAT	tag241332
novel_sir5435	AAAAGTCAACGGCGTCATACA	GTTTTTCAGTTGCCGCAGTAT	tag289349
novel_sir5436	TAACATAACCAGTAGCATACT	GAATTGTATTGGTCATCGTAT	tag57252
novel_sir5437	GACAAACAAAGGTCCGCCCGGTAT	GTTTGTTTCCAGGCGGGCCATAAT	tag155400
novel_sir5438	ACATTAATGATTAGAGGCCATATT	TATGTAATTACTAATCTCCGGTAT	tag106526
novel_sir5439	CTGAAAGATCGTAACGGGTAT	CTTTCTAGCATTGCCCATATT	tag119629

novel_sir5440	CGCTAGGTCTATACCCATACG	AGGCGATCCAGATATGGGTAT	tag249571
novel_sir5441	GAGGCGATCCAGATATGGGTAT	CCGCTAGGTCTATACCCATACG	tag128279
novel_sir5442	GTGTATGACAGGTGGGACCATATA	CACACATACTGTCCACCCTGGTAT	tag247041
novel_sir5443	GGTAGCAGGTGAAGTTGGTAT	ATCGTCCACTTCAACCATAGC	tag54972
novel_sir5444	ACTGTGAACCGCGAATTCATGTAT	ACACTTGGCGCTTAAGTACATATA	tag135165
novel_sir5445	GGGCGTAGACCGAACGGACTGTAT	CGCATCTGGCTTGCCTGACATAGA	tag9829
novel_sir5446	TACAATATCTGTGTACACCTGTAT	GTTATAGACACATGTGGACATATG	tag246388
novel_sir5447	GACATCGTGCCTGAGGTTCTGTAT	GTAGCACGGACTCCAAGACATAGT	tag149015
novel_sir5448	AGATGTAAACTCAGCTGTGTAT	TACATTTGAGTCGACACATACG	tag177287
novel_sir5449	CCGATATTCGGTCGATATTTGTAT	CTATAAGCCAGCTATAAACATATT	tag240854
novel_sir5450	GGTCAAACGTTGGACATGAATATC	TGCCAGTTTGCAACCTGTACTTAT	tag249936
novel_sir5451	TGCCAGTTTGCAACCTGTACTTAT	GGTCAAACGTTGGACATGAATACC	tag249936
novel_sir5452	CCTAGGACACCAGAATATC	GCGGATCCTGTGGTCTTAT	tag245620
novel_sir5453	TACGCCTAGGACACCAGAATATC	AGATGCGGATCCTGTGGTCTTAT	tag61918
novel_sir5454	TACGCCTAGGACACCAGAATATC	TGATGCGGATCCTGTGGTCTTAT	tag271968
novel_sir5455	CACCGAACGGTGTCAAAAAGTTAT	GGCTTGCCACAGTTTTTCAATAGC	tag246822
novel_sir5456	TCCAATTTTCTGAAAGATCGTTAT	GTTAAAAGACTTTCTAGCAATACT	tag94080
novel_sir5457	GGTATATCCGTCCATGCCAATATG	ACCCATATAGGCAGGTACGGTTAT	tag27663
novel_sir5458	TTCACCAGGTATTCGTTATGTTAT	GTGGTCCATAAGCAATACAATATT	tag285886
novel_sir5459	GTTGGTTTGCAATTCGATTGTTAT	ACCAAACGTTAAGCTAACAATATG	tag226382
novel_sir5460	ATAGTATTCAGCCGAATAAATAGT	CTTATCATAAGTCGGCTTATTTAT	tag140181
novel_sir5461	GGTCTAAGTCTAAGGAAATACA	CACCAGATTCAGATTCCTTTAT	tag20233
novel_sir5462	ATAGACAGTTCCGCGGCAAATAGT	CCTATCTGTCAAGGCGCCGTTTAT	tag139567
novel_sir5463	TCTAGGCATCGGGGTAGGTTTAT	ATCCGTAGCCCCATCCAAATAAC	tag153574
novel_sir5464	GCTAGGCATCGGGGTAGGTTTAT	ATCCGTAGCCCCATCCAAATAAC	tag211518
novel_sir5465	TGCTAGGCATCGGGGTAGGTTTAT	GATCCGTAGCCCCATCCAAATAAC	tag3659
novel_sir5466	AGCTAGGCATCGGGGTAGGTTTAT	GATCCGTAGCCCCATCCAAATAAC	tag19446
novel_sir5467	GGCTAGGCATCGGGGTAGGTTTAT	GATCCGTAGCCCCATCCAAATAAC	tag139193
novel_sir5468	CGCTAGGCATCGGGGTAGGTTTAT	GATCCGTAGCCCCATCCAAATAAC	tag188732
novel_sir5469	AACGGCGTCATACATTAAAATACG	AGTTGCCGCAGTATGTAATTTTAT	tag81872
novel_sir5470	AACGGCGTCATACATTAAAATATG	AGTTGCCGCAGTATGTAATTTTAT	tag81872
novel_sir5471	AACGGCGTCATACATTAAAATATA	AGTTGCCGCAGTATGTAATTTTAT	tag81872
novel_sir5472	AACGGCGTCATACATTAAAATAGA	AGTTGCCGCAGTATGTAATTTTAT	tag81872
novel_sir5473	CCTTCTACCGCTAGGTCAAAAACT	AAGATGGCGATCCAGTTTTTGAAT	tag278126

novel_sir5474	GTATCGCTGCAACGAAAAACT	TAGCGACGTTGCTTTTTGATC	tag176394
novel_sir5475	CTTTTTCTCACATGTTTGACC	CTGAAAAAGAGTGTACAAACT	tag55471
novel_sir5476	ACTGAAAAAGAGTGTACAAACT	ACTTTTTCTCACATGTTTGACC	tag287321
novel_sir5477	CTGAAAAATCGTGTACAAACT	CTTTTTAGCACATGTTTGACC	tag106240
novel_sir5478	ACGTGCCACTGGAATTTGAGG	GATGCACGGTGACCTTAAACT	tag22376
novel_sir5479	CGTTCGACCAGGAACCACAACT	AAGCTGGTCCTTGGTGTTGATG	tag42968
novel_sir5480	GGTCGAGACTATGGTACAACT	AGCTCTGATACCATGTTGAGT	tag142177
novel_sir5481	AACCAGTAGCGCGAACCAACT	GGTCATCGCGCTTGGTTGAAA	tag75210
novel_sir5482	GTGCCACTACATGTACGGTTGAGT	TACACGGTGATGTACATGCCAACT	tag234713
novel_sir5483	ATGCCGTGGCTGCCGTTGATG	GGTACGGCACCGACGGCAACT	tag274075
novel_sir5484	CATGCCGTGGCTGCCGTTGATG	GGGTACGGCACCGACGGCAACT	tag55596
novel_sir5485	AAATGTTTGACGCCGTTGACT	GATTTACAAACTGCGGCAACT	tag54275
novel_sir5486	AAATGTTTGACGCCGTTGACT	GTTTTACAAACTGCGGCAACT	tag79852
novel_sir5487	AAATATTTGACGCCGTTGACT	GCTTTATAAACTGCGGCAACT	tag237328
novel_sir5488	AAATATTTGACGCCGTTGACT	GTTTTATAAACTGCGGCAACT	tag254720
novel_sir5489	TAATGTATGACGCCGTTGACT	AAATTACATACTGCGGCAACT	tag253281
novel_sir5490	AATTATCTACTGCGGCAACT	AATAGATGACGCCGTTGACT	tag120826
novel_sir5491	TAATAGATGACGCCGTTGACT	AAATTATCTACTGCGGCAACT	tag40213
novel_sir5492	TAATAGATGACGCCGTTGATT	AAATTATCTACTGCGGCAACT	tag40213
novel_sir5493	GCTTTATAAACTGTGGCAACT	AAATATTTGACACCGTTGACT	tag11374
novel_sir5494	GTTTTATAAACTGTGGCAACT	AAATATTTGACACCGTTGACT	tag267485
novel_sir5495	TAGACGTGGCCAAAACTATCAACT	CTGCACCGGTTTTGATAGTTGAGG	tag17580
novel_sir5496	ACTATTCTAGCCATTGAGTTGAGA	TGTGATAAGATCGGTAACTCAACT	tag264392
novel_sir5497	TTATTCTGCCTGCTAGTTTCAACT	TAAGACGGACGATCAAAGTTGAGC	tag196226
novel_sir5498	TGTAGGATTATGTTGCTTAGAACT	ATCCTAATACAACGAATCTTGACA	tag40928
novel_sir5499	ACCATATCCCATTCACGCTTGACC	GGTGGTATAGGGTAAGTGCGAACT	tag32409
novel_sir5500	TGTGGTTGGCCCTGATTTCTAACT	ACCAACCGGGACTAAAGATTGATT	tag83960
novel_sir5501	GCTAGAATGACTTACATTGTGAAA	TACGATCTTACTGAATGTAACACT	tag33999
novel_sir5502	CCCAACAAACCCTAGACACACT	GTTGTTTGGGATCTGTGTGACT	tag19556
novel_sir5503	GGACGATCAAAGTTGGGTGTGAAA	TGCCTGCTAGTTTCAACCCACACT	tag170243
novel_sir5504	CCGCGATCTGACCGGTCTGTGACC	TGGGCGCTAGACTGGCCAGACACT	tag249634
novel_sir5505	CCGCGATCTGACCGGTCTGTGACT	TGGGCGCTAGACTGGCCAGACACT	tag249634
novel_sir5506	TCTGCTTACCAGTTTGTACACT	ACGAATGGTCAAACATGTGAGA	tag39007
novel_sir5507	TTCTGCTTACCAGTTTGTACACT	GACGAATGGTCAAACATGTGAGA	tag58079

novel_sir5508	CGGCCAAGGCCAAGGGTGGTGATG	CGGCCGGTTCCGGTTCCCACCACT	tag159175
novel_sir5509	GTGGCTGTTGTGTGGGTGAGC	GCCACCGACAACACACCCACT	tag18459
novel_sir5510	TCTCATCATGATCCTACCCACT	AGTAGTACTAGGATGGGTGACC	tag20277
novel_sir5511	CTGCCGCTGACCGTCGTGACC	GCGACGGCGACTGGCAGCACT	tag158870
novel_sir5512	CGCCAGACAGCTTCGCACT	GGTCTGTCGAAGCGTGATC	tag263228
novel_sir5513	CGGAAACGATTTGGTACCGTGAGG	TAGCCTTTGCTAAACCATGGCACT	tag55489
novel_sir5514	AATCAGAACCTTTGCAGTTTCACT	AGTCTTGGAAACGTCAAAGTGAAA	tag148180
novel_sir5515	ACGCTTAACTTCGGAGTTCTGATG	CGTGCGAATTGAAGCCTCAAGACT	tag162980
novel_sir5516	ATTCGATGGCATATTCTGAAA	CTTAAGCTACCGTATAAGACT	tag111079
novel_sir5517	CGCTTGCTAGCTTGGATTCTGACT	CGGCGAACGATCGAACCTAAGACT	tag159477
novel_sir5518	GACTGCACCGCGGATGCACAGACT	GACGTGGCGCCTACGTGTCTGATT	tag135028
novel_sir5519	GGCTACGGATCGGGCGTCTGATC	CCCCGATGCCTAGCCCGCAGACT	tag65895
novel_sir5520	GGGCTACGGATCGGGCGTCTGATC	ACCCCGATGCCTAGCCCGCAGACT	tag95610
novel_sir5521	TCACGAGTGAAACAGTCTGACG	GAAGTGCTCACTTTGTCAGACT	tag167225
novel_sir5522	TGTTAGATCGAATATCATTAGACT	AATCTAGCTTATAGTAATCTGACT	tag291121
novel_sir5523	AAACTCGGCTCAATCTGAAA	AATTTGAGCCGAGTTAGACT	tag80594
novel_sir5524	TAAACTCGGCTCAATCTGAAA	AAATTTGAGCCGAGTTAGACT	tag125136
novel_sir5525	TAAACTCGGCTCAATCTGAAA	TAATTTGAGCCGAGTTAGACT	tag154511
novel_sir5526	TAAACTCGGCTCAATCTGAAA	CAATTTGAGCCGAGTTAGACT	tag160711
novel_sir5527	ATTAAACTCGGCTCAATCTGAAA	GTTAATTTGAGCCGAGTTAGACT	tag2668
novel_sir5528	ATTAAACTCGGCTCAATCTGAAA	ATTAATTTGAGCCGAGTTAGACT	tag144760
novel_sir5529	AATTAAACTCGGCTCAATCTGAAA	AGTTAATTTGAGCCGAGTTAGACT	tag291944
novel_sir5530	CAATTAAACTCGGCTCAATCTGAAA	AAGTTAATTTGAGCCGAGTTAGACT	tag14460
novel_sir5531	ACGCAGACATCGTCGTTAGACT	CGTCTGTAGCAGCAATCTGATC	tag98434
novel_sir5532	CACCTCGACTTTGACAAACCGACT	GGAGCTGAAACTGTTTGGCTGAGC	tag256758
novel_sir5533	TGATCTGAAAGCCGACCGACT	TAGACTTTCGGCTGGCTGAGA	tag111334
novel_sir5534	TTGATCTGAAAGCCGACCGACT	CTAGACTTTCGGCTGGCTGAGA	tag209610
novel_sir5535	CTCCTAAGTCGTTGGGCTGATT	GAGAGGATTCAGCAACCCGACT	tag142266
novel_sir5536	AGGGTTGCCCGCCGCCCGACT	CCAACGGGCGGCGGGCTGAAT	tag237013
novel_sir5537	GGGCTTTTACCTACCGCGACT	CGAAAATGGATGGCGCTGAAG	tag96448
novel_sir5538	ACCCCGCTGTGCTCAGCATCGACT	GGGCGACACGAGTCGTAGCTGAGT	tag139797
novel_sir5539	GGGCAACACGAGTCGTAGCTGAGT	ACCCCGTTGTGCTCAGCATCGACT	tag283127
novel_sir5540	GTATTCAGTGGGCGCTAGCTGAAA	CCCATAAGTCACCCGCGATCGACT	tag123028
novel_sir5541	CCTCCGAGCTTCGCTATGACT	AGGCTCGAAGCGATACTGACG	tag75782

novel_sir5542	GGCACACGGCCTAATACTGACT	GTGTGCCGGATTATGACTGAAA	tag5
novel_sir5543	GGCACACGGCCTAATACTGACT	GTGTGCCGGATTATGACTGAAC	tag5
novel_sir5544	TAGAAAAGTCGATCGCGGGTGACT	CTTTTCAGCTAGCGCCCACTGAAT	tag195629
novel_sir5545	GGGACCGGGGTGACACAACTGAAC	ATCCCTGGCCCCACTGTGTTGACT	tag272961
novel_sir5546	GTGGGTCCACATGTGTCTATGACA	TACACCCAGGTGTACACAGATACT	tag68448
novel_sir5547	CGTCAATGGACCGTCTTAGATACT	AGTTACCTGGCAGAATCTATGATT	tag42089
novel_sir5548	CCGTGGCTGCCGTTGATGACG	ACGGCACCGACGGCAACTACT	tag114197
novel_sir5549	CGCTGCTTGCTGCTGCCTACT	GACGAACGACGACGGATGACG	tag166301
novel_sir5550	CGACGAACGACGACGGATGACG	GCGCTGCTTGCTGCTGCCTACT	tag119783
novel_sir5551	AATTGGGTTCCGGATGCTACT	AACCCAAGGCCTACGATGATT	tag189896
novel_sir5552	GAATTGGGTTCCGGATGCTACT	TAACCCAAGGCCTACGATGATT	tag192801
novel_sir5553	TAATTAATTACCGGCTTCTACT	TAATTAATGGCCGAAGATGACT	tag169051
novel_sir5554	ATCTGTGTGACTTGCCATGAGC	CCTAGACACACTGAACGGTACT	tag2611
novel_sir5555	TGGCCAGGAATTAACGGTACT	CGGTCCTTAATTGCCATGAGC	tag223362
novel_sir5556	CCGGTCCTTAATTGCCATGAGC	TTGGCCAGGAATTAACGGTACT	tag285622
novel_sir5557	TCTTGACCTACGCCATGTACT	AACTGGATGCGGTACATGACC	tag150613
novel_sir5558	TGTCTGCTAGTTTCAATCTGTACT	AGACGATCAAAGTTAGACATGAAA	tag41964
novel_sir5559	GATGCGGCATGTGGAGACATGAGC	TTCTACGCCGTACACCTCTGTACT	tag130420
novel_sir5560	AGGGTTTTTCGGATACGAATGAAA	CTTCCCAAAAAGCCTATGCTTACT	tag94952
novel_sir5561	CACGGGCGCCGACCGGCAATGATC	TGGTGCCCGCGGCTGGCCGTTACT	tag152682
novel_sir5562	GGACTCGGAATCAGGCAAATGATA	TGCCTGAGCCTTAGTCCGTTTACT	tag72113
novel_sir5563	CGCGGCCTAAAACCCATGTTTACT	GCCGGATTTTGGGTACAAATGATC	tag113329
novel_sir5564	ATCAACCGATATATCCACAAACCT	GTTGGCTATATAGGTGTTTGGAGA	tag187857
novel_sir5565	GACTATGCTCACAGACTCAAACCT	GATACGAGTGTCTGAGTTTGGATT	tag4420
novel_sir5566	GGCATTTAGCTAGTCTGTTGGAGT	CTCCGTAAATCGATCAGACAACCT	tag110013
novel_sir5567	CTCGGTAAATCGGTGAGACAACCT	GCCATTTAGCCACTCTGTTGGAGT	tag227652
novel_sir5568	AAGGCTATATACTCGTTGGATA	GGTTCCGATATATGAGCAACCT	tag243934
novel_sir5569	ATTCTGCTTACCAGTTTGCAACCT	AGACGAATGGTCAAACGTTGGATA	tag213928
novel_sir5570	AGACGAATGGTCAAACGTTGGACA	ATTCTGCTTACCAGTTTGCAACCT	tag213928
novel_sir5571	CAGTATGTATGTAACGCAGAACCT	CATACATACATTGCGTCTTGGAGT	tag141481
novel_sir5572	CAGTATATGTGTAACGCAGAACCT	CATATACACATTGCGTCTTGGAGT	tag84373
novel_sir5573	CAGTATGTGTGTAACGCAGAACCT	CATACACACATTGCGTCTTGGAGT	tag283365
novel_sir5574	CATACACACACTGCGTCTTGGAGT	CAGTATGTGTGTGACGCAGAACCT	tag183928
novel_sir5575	TGTAAAACGTCGTGCTTAGAACCT	ATTTTGCAGCACGAATCTTGGAAC	tag192935

novel_sir5576	TCCACCGCCGCCACCGAACCT	GTGGCGGCGGTGGCTTGGACC	tag96031
novel_sir5577	CTCCACCGCCGCCACCGAACCT	GGTGGCGGCGGTGGCTTGGACC	tag8553
novel_sir5578	GCCGCTTGCTAGCTTGGATT	CGCGGCGAACGATCGAACCT	tag214114
novel_sir5579	CGCCGCTTGCTAGCTTGGATT	CCGCGGCGAACGATCGAACCT	tag190626
novel_sir5580	GCGCCGCTTGCTAGCTTGGATT	TCCGCGGCGAACGATCGAACCT	tag84309
novel_sir5581	GTGTGGAATCGATCGTTTTAACCT	CACCTTAGCTAGCAAAATTGGATT	tag243419
novel_sir5582	TGGGATGAGGACACAAGACCACCT	CCTACTCCTGTGTTCTGGTGGATC	tag285468
novel_sir5583	AGGCCATGAGAGCGAACGTGGAGG	GATCCGGTACTCTCGCTTGCACCT	tag140226
novel_sir5584	ACCCTCTCTACCGACTCACCT	GGAGAGATGGCTGAGTGGACC	tag112183
novel_sir5585	CCCCTCTCTACCGACTCACCT	GGAGAGATGGCTGAGTGGACC	tag124570
novel_sir5586	GGAGAGATGGCTGAGTGGACG	CCCCTCTCTACCGACTCACCT	tag124570
novel_sir5587	GGAGAGATGGCTGAGTGGACA	CCCCTCTCTACCGACTCACCT	tag124570
novel_sir5588	GGAGAGATGGCTGAGTGGACT	CCCCTCTCTACCGACTCACCT	tag124570
novel_sir5589	CCTACCGTTGCCTCCTCACCT	ATGGCAACGGAGGAGTGGAGA	tag138280
novel_sir5590	GGCTGGATCTCAGTGGATC	CCCCGACCTAGAGTCACCT	tag151307
novel_sir5591	GGGGCTGGATCTCAGTGGATC	CGCCCCGACCTAGAGTCACCT	tag186342
novel_sir5592	ATCGCGTGCTCTGGATTTTCACCT	GCGCACGAGACCTAAAAGTGGATG	tag282298
novel_sir5593	TCAACGTGTACTGCAGTCTGGAGA	GCAGTTGCACATGACGTCAGACCT	tag131408
novel_sir5594	CCGCTCGCCCGCTCCCGACCT	CGAGCGGGCGAGGGCTGGAGC	tag30464
novel_sir5595	GACGCCGACAGGAGCTGGATC	GGCTGCGGCTGTCCTCGACCT	tag142914
novel_sir5596	CGACGCCGACAGGAGCTGGATC	CGGCTGCGGCTGTCCTCGACCT	tag218157
novel_sir5597	GGCGGCTGCGGCTGTCCTCGACCT	GCCGACGCCGACAGGAGCTGGATC	tag162185
novel_sir5598	CTTCCTTCTGACATTCGACCT	AGGAAGACTGTAAGCTGGACA	tag279884
novel_sir5599	GGTGAGAATTTTAAACGATGACCT	ACTCTTAAAATTTGCTACTGGACC	tag331
novel_sir5600	AACTGACCTGAACTGTGACCT	GACTGGACTTGACACTGGATA	tag60733
novel_sir5601	ATTTGTTTTCATGTGCCTACCT	AACAAAAGTACACGGATGGAGA	tag80904
novel_sir5602	GCGGTGCTTGACGCGGTACCT	CCACGAACTGCGCCATGGAGC	tag74779
novel_sir5603	GCGTCCATTGTCTAATGGATA	TTCGCAGGTAACAGATTACCT	tag135841
novel_sir5604	TTGCCGCTACCGTAAAAACCCT	CGGCGATGGCATTTTTGGGACA	tag60928
novel_sir5605	CCGGGGGAGCCTAGCAAACCCT	CCCCCTCGGATCGTTTGGGACC	tag244877
novel_sir5606	GCGGGGGAGCCTAGCAAACCCT	CCCCCTCGGATCGTTTGGGACC	tag270942
novel_sir5607	CCGGGGGAGCCTAGCAAACCCT	CCCCCTCGGATCGTTTGGGACT	tag244877
novel_sir5608	GCGGGGGAGCCTAGCAAACCCT	CCCCCTCGGATCGTTTGGGACT	tag270942
novel_sir5609	ATGCGAAAACTTTGTGGGAGA	CCTACGCTTTTGAAACACCCT	tag186126

novel_sir5610	CGGCCTTCGTCCGCGCTGCACCCT	CGGAAGCAGGCGCGACGTGGGAAC	tag101515
novel_sir5611	TGGCGTGCTTCAGTGAAAACCCCT	CGCACGAAGTCACTTTTGGGGAAA	tag156345
novel_sir5612	AGGTGACGGCGCGTGGGGAGG	TCTCCACTGCCGCGCACCCCT	tag91213
novel_sir5613	ACAGGCAGTCGACTGGGGAAC	GGTGTCCGTCAGCTGACCCCT	tag66218
novel_sir5614	GGCGCGGCGATGTGGGGGAGG	GGCCGCGCCGCTACACCCCCT	tag277429
novel_sir5615	GGCCGGCGACGAAGGGGGAGG	CGCCGGCCGCTGCTTCCCCCT	tag200408
novel_sir5616	CGGCCGGCGACGAAGGGGGAGG	CCGCCGGCCGCTGCTTCCCCCT	tag137748
novel_sir5617	TCGCCACTACCGCGCGCCCCT	CGGTGATGGCGCGCGGGGAGG	tag289580
novel_sir5618	AACGAAGTAGGCTGGGCCCCT	GCTTCATCCGACCCGGGGAAA	tag132677
novel_sir5619	CGTCTGGCTATAACGTCCCCT	AGACCGATATTGCAGGGGAGT	tag7863
novel_sir5620	CAGTGCCCGATCAAGGCCAGCCCT	CACGGGCTAGTTCCGGTCGGGACT	tag210100
novel_sir5621	CTCATAACGAAAGTATGCCGCCCT	GTATTGCTTTCATACGGCGGGAGT	tag25076
novel_sir5622	CGAGTTACCAAACAAGCCGGGAGT	TAGCTCAATGGTTTGTTCGGCCCT	tag171051
novel_sir5623	CGCCGTCGGCAGCCCGGGAAG	GTGCGGCAGCCGTCGGGCCCT	tag1598
novel_sir5624	GTCCTAATCCAACCACACGGGATA	TGCAGGATTAGGTTGGTGTGCCCT	tag206601
novel_sir5625	CGGCTGATCGTACCACTCCCT	CGACTAGCATGGTGAGGGACG	tag161709
novel_sir5626	GCGGCTGATCGTACCACTCCCT	CCGACTAGCATGGTGAGGGACG	tag122869
novel_sir5627	ACTCTGAAAACCTTCCTCCCT	AGACTTTTGGAAGGAGGGAGT	tag161069
novel_sir5628	CAATATCAAGACGTGGCAGGGACC	AAGTTATAGTTCTGCACCGTCCCT	tag98597
novel_sir5629	ATGGTTGATGACTAGGTCCCT	CCAACTACTGATCCAGGGATT	tag64539
novel_sir5630	CTTCTACTTATCACGGTCCCT	AGATGAATAGTGCCAGGGAGG	tag202529
novel_sir5631	CGCCGCCCGTTGGGAAGGGAGC	GGGCGGCGGGCAACCCTTCCCT	tag235694
novel_sir5632	CCGCCGCCCGTTGGGAAGGGAGC	CGGGCGGCGGGCAACCCTTCCCT	tag40342
novel_sir5633	CTCCGGTTCTGACATTAAAAGCCT	GGCCAAGACTGTAATTTTCGGAGT	tag229047
novel_sir5634	GAGTTATGCCAAACGGTTCGGATT	ACCTCAATACGGTTTGCCAAGCCT	tag115835
novel_sir5635	CAAGCACGCTTAACTTCGGAGT	GGGTTCGTGCGAATTGAAGCCT	tag73973
novel_sir5636	GCTTTTACTCAAGCTAAGCCT	AAAATGAGTTCGATTCGGATA	tag30820
novel_sir5637	AGCTTTTACTCAAGCTAAGCCT	GAAAATGAGTTCGATTCGGATA	tag77255
novel_sir5638	AGCTTGCAAACTGACCTACAGCCT	GAACGTTTGACTGGATGTCGGAAG	tag281819
novel_sir5639	AGCTTGCAAACCGGCCTACAGCCT	GAACGTTTGGCCGGATGTCGGAAG	tag291341
novel_sir5640	AGCTTACAAACTGGCCTACAGCCT	GAATGTTTGACCGGATGTCGGAAG	tag79540
novel_sir5641	TCTGCAAACTGGCCTACAGCCT	ACGTTTGACCGGATGTCGGAAG	tag107081
novel_sir5642	CCTGCAAACTGGCCTACAGCCT	ACGTTTGACCGGATGTCGGAAG	tag138774
novel_sir5643	GGACGTTTGACCGGATGTCGGAAG	AACCTGCAAACTGGCCTACAGCCT	tag26631

novel_sir5644	GGACGTTTGACCGGATGTCGGAAG	ATCCTGCAAACTGGCCTACAGCCT	tag284526
novel_sir5645	AACCTGCAAACTGGCCTACAGCCT	GGACGTTTGACCGGATGTCGGAAA	tag26631
novel_sir5646	AGCCTGCAAACTGGCCTACAGCCT	GGACGTTTGACCGGATGTCGGAAA	tag236675
novel_sir5647	ATCCTGCAAACTGGCCTACAGCCT	GGACGTTTGACCGGATGTCGGAAA	tag284526
novel_sir5648	GCACGTTTGACCGGATGTCGGAAA	AGCGTGCAAACTGGCCTACAGCCT	tag33897
novel_sir5649	AGCTTGCAAACTGGCCTACAGCCT	GAACGTTTGACCGGATGTCGGAAG	tag149315
novel_sir5650	GAACGTTTGACCGGATGTCGGAAA	AGCTTGCAAACTGGCCTACAGCCT	tag149315
novel_sir5651	AGCTTGCAAACTGGTCTACAGCCT	GAACGTTTGACCAGATGTCGGAAC	tag153282
novel_sir5652	TACGAATATGTATATGATCAGCCT	GCTTATACATATACTAGTCGGATG	tag265796
novel_sir5653	TCTACGATACCCGAGATAGCCT	ATGCTATGGGCTCTATCGGACA	tag53150
novel_sir5654	AGCCTACAAACTGGCCTATAGCCT	GGATGTTTGACCGGATATCGGAAG	tag255220
novel_sir5655	AGCCTGCAAACTGGCCTATAGCCT	GGACGTTTGACCGGATATCGGAAG	tag161840
novel_sir5656	CCTCTCCCCCAATAATCGGAGC	TAGGAGAGGGGGTTATTAGCCT	tag237984
novel_sir5657	TGCCTCGAGCCCTACACCGCCT	GGAGCTCGGGATGTGGCGGATT	tag242448
novel_sir5658	TGGGATGAGGACACAAGACCGCCT	CCTACTCCTGTGTTCTGGCGGATC	tag152651
novel_sir5659	GGTAGTGTCCCGCTGCCGCCT	ATCACAGGGCGACGGCGGAGG	tag35197
novel_sir5660	CCCTTCGCCGCCGCTGCCGCCT	GAAGCGGCGGCGACGGCGGAAT	tag178446
novel_sir5661	CATCTGGTTTTGGTGGCGCCT	AGACCAAAACCACCGCGGATT	tag236205
novel_sir5662	TAGACCAAAACCACCGCGGATT	GCATCTGGTTTTGGTGGCGCCT	tag259819
novel_sir5663	CCCACAAGTAGCCGATGCGCCT	GTGTTCATCGGCTACGCGGAGG	tag132980
novel_sir5664	GATGATCAAAGTTAAACGCGGAAA	GCCTACTAGTTTCAATTTGCGCCT	tag86214
novel_sir5665	TTCCGGTCGAAGACGGCATCGCCT	GGCCAGCTTCTGCCGTAGCGGAGT	tag241843
novel_sir5666	GGCCAACTTCTGCCGTAGCGGAGC	TTCCGGTTGAAGACGGCATCGCCT	tag104359
novel_sir5667	TACCCATATCTGGATCGCCT	GGGTATAGACCTAGCGGAGT	tag166552
novel_sir5668	CATACCCATATCTGGATCGCCT	ATGGGTATAGACCTAGCGGAGT	tag103872
novel_sir5669	CCGCTGCAATCCTTCAGGCCT	CGACGTTAGGAAGTCCGGAGA	tag200395
novel_sir5670	GGCTGTCCTCGACCTAGGCCT	GACAGGAGCTGGATCCGGAGC	tag124851
novel_sir5671	CGCTTCGATGGCACACGGCCT	GAAGCTACCGTGTGCCGGATT	tag131810
novel_sir5672	GCGCTTCGATGGCACACGGCCT	CGAAGCTACCGTGTGCCGGATT	tag155560
novel_sir5673	GTGCGTGCGCCTGCCCGGCCT	CGCACGCGGACGGGCCGGAGG	tag215867
novel_sir5674	CGGTGCAGCGGCCGGCGGCCT	CACGTCGCCGGCCGCCGGACC	tag85294
novel_sir5675	ACCTTATGAAAAGCTAGCCGGAAG	AATGGAATACTTTTCGATCGGCCT	tag195796
novel_sir5676	AAGGAAGAGGGGAGCCGGACG	CCTTCCTTCTCCCCTCGGCCT	tag193023
novel_sir5677	GACGTTCTGGTTGTATGAAAGGGCCT	GCAAGACCAACATACTTTCCCGGAAA	tag90855

novel_sir5678	AACGTTCTGGTTGTATGAAAGGGCCT	GCAAGACCAACATACTTTCCCGGAAA	tag95165
novel_sir5679	GCTGCGTTGCCGCCCGGGCCT	ACGCAACGGCGGGCCCGGACG	tag79677
novel_sir5680	AGTGGGGGTTGATACGTTTGGCCT	ACCCCCAACTATGCAAACCGGACG	tag85697
novel_sir5681	GTCCTCCAACTACAAAACCGGATA	AGCAGGAGGTTGATGTTTTGGCCT	tag143103
novel_sir5682	CACTGGGATTTGGTGTTTTGGCCT	GACCCTAAACCACAAAACCGGATA	tag169333
novel_sir5683	TACCCACCGCCAGATCAGATGCCT	GGGTGGCGGTCTAGTCTACGGATT	tag61493
novel_sir5684	AGGTTAAAAATGTGGATGCCT	CAATTTTTACACCTACGGATC	tag90553
novel_sir5685	CGCAGGTTAAAAATGTGGATGCCT	GTCCAATTTTTACACCTACGGATC	tag29429
novel_sir5686	CGCACAGTGTAGCCTATATGCCT	GTGTCACATCGGATATACGGACA	tag24263
novel_sir5687	CCTACACCGCCTAAGTTGCCT	ATGTGGCGGATTCAACGGAGG	tag167316
novel_sir5688	CCCTACACCGCCTAAGTTGCCT	GATGTGGCGGATTCAACGGAGG	tag144707
novel_sir5689	GCTGCTGCTCCTACCGTTGCCT	ACGACGAGGATGGCAACGGAGA	tag189427
novel_sir5690	GCTGCTGCTCCTACCGTTGCCT	ACGACGAGGATGGCAACGGAGG	tag189427
novel_sir5691	ACAGTTTATAAAACTTTGCCT	TCAAATATTTTGAAACGGAGG	tag116783
novel_sir5692	ACAGTTTGTAAAACTTTGCCT	TCAAACATTTTGAAACGGAGG	tag283959
novel_sir5693	ATTGAATGTAACACTTTGCCT	ACTTACATTGTGAAACGGAGG	tag137977
novel_sir5694	ACTGAATGTAACACTTTGCCT	ACTTACATTGTGAAACGGAGG	tag153867
novel_sir5695	TTGAACTTGGTGGACTTTGCCT	CTTGAACCACCTGAAACGGATC	tag266683
novel_sir5696	ACTGAATGTAATACTTTGCCT	ACTTACATTATGAAACGGAGG	tag67162
novel_sir5697	GGAGTTGATTAAACGTTTGCCT	TCAACTAATTTGCAAACGGAGC	tag173692
novel_sir5698	GCCAACTGCCGCTGCAATCCT	GTTGACGGCGACGTTAGGAAG	tag214366
novel_sir5699	TTTAGTCGGGTTGCTGAATCCT	ATCAGCCCAACGACTTAGGAGA	tag25840
novel_sir5700	GGTGTAGTCGCGGTGCATCCT	ACATCAGCGCCACGTAGGATC	tag80027
novel_sir5701	GATGTGACATATCCTAGGACT	ATCTACACTGTATAGGATCCT	tag232245
novel_sir5702	AACCACACCAAGGAGATGCGGATCCT	GGTGTGGTTCCTCTACGCCTAGGAAA	tag71300
novel_sir5703	CACCACACCAAGGAGATGCGGATCCT	GGTGTGGTTCCTCTACGCCTAGGAAA	tag227394
novel_sir5704	TGCGCTCCCGGACACACGTATCCT	GCGAGGGCCTGTGTGCATAGGATA	tag241987
novel_sir5705	CGTGACTCGATGACTCCT	ACTGAGCTACTGAGGAAC	tag5593
novel_sir5706	TGTGACTCGATGACTCCT	ACTGAGCTACTGAGGAAC	tag62386
novel_sir5707	AGTGACTCGATGACTCCT	ACTGAGCTACTGAGGAAC	tag94802
novel_sir5708	GGTGACTCGATGACTCCT	ACTGAGCTACTGAGGAAC	tag99722
novel_sir5709	ACTGAGCTACTGAGGAAA	AGTGACTCGATGACTCCT	tag94802
novel_sir5710	CACTGAGCTACTGAGGAAA	AGGTGACTCGATGACTCCT	tag164800
novel_sir5711	CACTGAGCTACTGAGGAAC	AGGTGACTCGATGACTCCT	tag164800

novel_sir5712	AATGGTGACTCGATGACTCCT	ACCACTGAGCTACTGAGGAAC	tag56343
novel_sir5713	GATGGTGACTCGATGACTCCT	ACCACTGAGCTACTGAGGAAC	tag215789
novel_sir5714	TGATGGTGACTCGATGACTCCT	TACCACTGAGCTACTGAGGAAC	tag75468
novel_sir5715	AGATGGTGACTCGATGACTCCT	TACCACTGAGCTACTGAGGAAC	tag142444
novel_sir5716	CGATGGTGACTCGATGACTCCT	TACCACTGAGCTACTGAGGAAC	tag224384
novel_sir5717	GGATGGTGACTCGATGACTCCT	TACCACTGAGCTACTGAGGAAC	tag280551
novel_sir5718	TACAGCTGGATCAGAAGGCCTCCT	GTCGACCTAGTCTTCCGGAGGAGT	tag133791
novel_sir5719	TACACCGCCTAAGTTGCCTCCT	GTGGCGGATTCAACGGAGGAAG	tag224567
novel_sir5720	GAATGTAACACTTTGCCTCCT	TACATTGTGAAACGGAGGAAG	tag171692
novel_sir5721	AAATGTAACACTTTGCCTCCT	TACATTGTGAAACGGAGGAAG	tag239899
novel_sir5722	CCCCGTTTGCTTTCTCCTCCT	GGCAAACGAAAGAGGAGGACT	tag49726
novel_sir5723	ACCCCGTTTGCTTTCTCCTCCT	GGGCAAACGAAAGAGGAGGACT	tag29178
novel_sir5724	TGCGCTTGGCTGCACCGTCCTCCT	GCGAACCGACGTGGCAGGAGGAGG	tag281903
novel_sir5725	CCATCTGTGGCTGCGGCTCCT	TAGACACCGACGCCGAGGACG	tag137544
novel_sir5726	CGGGTTGCTGAATCCTCTCCT	CCAACGACTTAGGAGAGGATT	tag150364
novel_sir5727	TCGGGTTGCTGAATCCTCTCCT	CCCAACGACTTAGGAGAGGATT	tag155554
novel_sir5728	ACAGGCCCTTAAAGTCAGAGGATC	TTTGTCCGGGAATTTCAGTCTCCT	tag107668
novel_sir5729	TCCTCCGGCCGCGTGTCTCCT	GAGGCCGGCGCACAGAGGACG	tag41063
novel_sir5730	TCTACCCCGTTTGCTTTCTCCT	ATGGGGCAAACGAAAGAGGAGG	tag91653
novel_sir5731	GTTGCATACGTCGAAGGTCCT	ACGTATGCAGCTTCCAGGAGG	tag245398
novel_sir5732	CTGCAGAATTGTACCGGCGGTCCT	CGTCTTAACATGGCCGCCAGGAAA	tag239882
novel_sir5733	GGCCGCTCTCCGACCAGGACA	GGCCGGCGAGAGGCTGGTCCT	tag212165
novel_sir5734	ACCACGCGGCGGCGGCTGTCCT	GTGCGCCGCCGCCGACAGGAGC	tag285487
novel_sir5735	GGCGGCGGCTGCGGCTGTCCT	GCCGCCGACGCCGACAGGAGC	tag105070
novel_sir5736	CGGCGGCGGCTGCGGCTGTCCT	CGCCGCCGACGCCGACAGGAGC	tag154310
novel_sir5737	GTCCTAACCCAACCACACAGGATA	CGCAGGATTGGGTTGGTGTGTCCT	tag552
novel_sir5738	CGCGATTGTTACCCTTGTCCT	GCTAACAATGGGAACAGGAGT	tag102504
novel_sir5739	TCGCGATTGTTACCCTTGTCCT	CGCTAACAATGGGAACAGGAGT	tag152441
novel_sir5740	CTTTACATGTCAAGCTTAAGGAGT	TAGAAATGTACAGTTCGAATTCCT	tag229963
novel_sir5741	CACCAGATTCAGATTCCT	GGTCTAAGTCTAAGGAAC	tag56535
novel_sir5742	AACCAGATTCAGATTCCT	GGTCTAAGTCTAAGGAAC	tag267256
novel_sir5743	GGTCTAAGTCTAAGGAAA	CACCAGATTCAGATTCCT	tag56535
novel_sir5744	AGATTTGCCGCTGAACTTCCT	TAAACGGCGACTTGAAGGAGG	tag70315
novel_sir5745	TACAAACTGGTTTACAGCCTTCCT	GTTTGACCAAATGTCGGAAGGAGT	tag1842

novel_sir5746	AGGTCGCGGCGTTCAAGGAGG	TCTCCAGCGCCGCAAGTTCCT	tag205733
novel_sir5747	GTCTCCAGCGCCGCAAGTTCCT	GAGGTCGCGGCGTTCAAGGAGG	tag163638
novel_sir5748	CGTCTCCAGCGCCGCAAGTTCCT	AGAGGTCGCGGCGTTCAAGGAGG	tag214547
novel_sir5749	AACTCCGAAGGTCTAAAGGATC	GTTTGAGGCTTCCAGATTTCCT	tag221504
novel_sir5750	AAATTTGTACCGACAATGTTTCCT	TAAACATGGCTGTTACAAAGGATT	tag201383
novel_sir5751	CGAATCGAACTCATTTTCGAGT	AGGCTTAGCTTGAGTAAAAGCT	tag170144
novel_sir5752	TTTAATTATCACGTCAAAGCT	ATTAATAGTGCAGTTTCGATG	tag224410
novel_sir5753	AATTAATAGTGCAGTTTCGATG	ATTTAATTATCACGTCAAAGCT	tag93778
novel_sir5754	TTTATAGACCAAAACGCCAAGCT	ATATCTGGTTTTGCGGTTCGAGG	tag230093
novel_sir5755	TACAGTAAAGCGTAATAGCAAGCT	GTCATTTCGCATTATCGTTCGACT	tag261862
novel_sir5756	ACGGCGACTAGGCGTTCGAGC	GCTGCCGCTGATCCGCAAGCT	tag262869
novel_sir5757	CGACATCCAGTCAAACGTTCGATG	AGGCTGTAGGTCAGTTTGCAAGCT	tag136263
novel_sir5758	CGACATCCGGCCAAACGTTCGATG	AGGCTGTAGGCCGGTTTGCAAGCT	tag222704
novel_sir5759	CCTGCCTCTCCGCCTTCGAGC	GAGGACGGAGAGGCGGAAGCT	tag244362
novel_sir5760	CGAGGGCGCGGCTATTCGAAT	TGGCTCCCGCGCCGATAAGCT	tag254260
novel_sir5761	TTGGCTCCCGCGCCGATAAGCT	CCGAGGGCGCGGCTATTCGAAT	tag256970
novel_sir5762	TTGGCGCCCGTGCCGATAAGCT	CCGCGGGCACGGCTATTCGAAT	tag117465
novel_sir5763	AGGCTGTAGGCCAGTTTGTAAGCT	CGACATCCGGTCAAACATTCGATG	tag239784
novel_sir5764	GCTCACGCGTGTTGGTCGAGG	GCCGAGTGCGCACAACCAGCT	tag17593
novel_sir5765	GCGTGCTCGTAGTGGTCGATG	TCCGCACGAGCATCACCAGCT	tag230464
novel_sir5766	GAGGCGTGCTCGTAGTGGTCGATG	GACTCCGCACGAGCATCACCAGCT	tag59801
novel_sir5767	GACTGCACCGTCTGCCACCCAGCT	GACGTGGCAGACGGTGGGTCGACC	tag2280
novel_sir5768	GATGTTTCAACATCCAGCGCAGCT	ACAAAGTTGTAGGTCGCGTCGAGG	tag161760
novel_sir5769	AAAGTTGTAGATCGCGTCGAGG	TGTTTCAACATCTAGCGCAGCT	tag61297
novel_sir5770	GGGTCGGCGCGGCGCGGCAGCT	CAGCCGCGCCGCGCCGTCGATT	tag162290
novel_sir5771	GCTAAAAAGTTAACGACGTCGAAC	CACGATTTTTCAATTGCTGCAGCT	tag216482
novel_sir5772	GCGGCTAGGAGCAGCATCAGCT	CCGATCCTCGTCGTAGTCGACG	tag248818
novel_sir5773	TTCCGTAGCAGCATCAGCT	GGCATCGTCGTAGTCGATC	tag39730
novel_sir5774	CGCTTCCGTAGCAGCATCAGCT	GAAGGCATCGTCGTAGTCGATC	tag93906
novel_sir5775	GAGTCGTAGCTGAGTCAGTCGACC	TGCTCAGCATCGACTCAGTCAGCT	tag44446
novel_sir5776	TCCAGAGCAAGGGGTCAGCT	GTCTCGTTCCCCAGTCGACT	tag6480
novel_sir5777	ATCCAGAGCAAGGGGTCAGCT	GGTCTCGTTCCCCAGTCGACT	tag108948
novel_sir5778	ACCCAGAGCAAGGGGTCAGCT	GGTCTCGTTCCCCAGTCGACT	tag257060
novel_sir5779	GCCGGGACACGGTACTGTAGAGCT	GCCCTGTGCCATGACATCTCGACT	tag112604

novel_sir5780	GGCGCTCTGGCTTGCTCGACT	GTCCGCGAGACCGAACGAGCT	tag11840
novel_sir5781	GCGGCGCCGGCCGGCGGAGCT	CCGCGGCCGGCCGCCTCGAAG	tag259234
novel_sir5782	TCAAACTTGTAGCCCTCGACG	ATAGTTTGAACATCGGGAGCT	tag43371
novel_sir5783	AATATAGTTTGAACATCGGGAGCT	ATATCAAACTTGTAGCCCTCGACG	tag63007
novel_sir5784	ATATCAAACTTGTAGCCCTCGACA	AATATAGTTTGAACATCGGGAGCT	tag63007
novel_sir5785	CTCAACGAGAATCCGGGGCTAGCT	GTTGCTCTTAGGCCCCGATCGAGA	tag159931
novel_sir5786	AATGGTTGGCCTTGATTTCTAGCT	ACCAACCGGAACTAAAGATCGATG	tag31175
novel_sir5787	GCTGTAGCTCCTGACGTAGCT	ACATCGAGGACTGCATCGACC	tag10054
novel_sir5788	GCTGGTGCGGCCGCTGTAGCT	ACCACGCCGGCGACATCGACA	tag225208
novel_sir5789	CGCTGGTGCGGCCGCTGTAGCT	GACCACGCCGGCGACATCGACA	tag225906
novel_sir5790	TTGAACGTTTCGCGTGTAGCT	CTTGCAAAGCGCACATCGAGG	tag221100
novel_sir5791	TTGACTTGCCAGCTAAACGCT	CTGAACGGTCGATTTGCGAGT	tag279952
novel_sir5792	CCGCCGTCGGCCAAAATTGCGATT	ACGGCGGCAGCCGGTTTTAACGCT	tag132878
novel_sir5793	CTCTGCTCGGTCGTCGCTGCGACG	AGGAGACGAGCCAGCAGCGACGCT	tag103826
novel_sir5794	GGTGAATTCGCCGCGGACGCT	ACTTAAGCGGCGCCTGCGAAA	tag131112
novel_sir5795	CGCTTAAGTGGTCCGCCTGCGAAA	CGGCGAATTCACCAGGCGGACGCT	tag197351
novel_sir5796	AAGGTTCGGCGGCTGGCGACA	TCTTCCAAGCCGCCGACCGCT	tag240521
novel_sir5797	GGGACCTCGCCATCTACCGCT	CTGGAGCGGTAGATGGCGAGG	tag162931
novel_sir5798	GGGGACCTCGCCATCTACCGCT	CCTGGAGCGGTAGATGGCGAGG	tag272023
novel_sir5799	CTTCAATTCGCACGAACCCGCT	AGTTAAGCGTGCTTGGGCGAGA	tag160132
novel_sir5800	TAGCTAGCCGCCGTCGGCGAGG	GCATCGATCGGCGGCAGCCGCT	tag122025
novel_sir5801	CGGGCGCGGGTGGCGGCGAGC	CCGCCCGCGCCCACCGCCGCT	tag220891
novel_sir5802	CCGCCCGCGCCGACCGCCGCT	CGGGCGCGGCTGGCGGCGAGC	tag9189
novel_sir5803	CTTCACAAGCCTAGCGCCGCT	AGTGTTCGGATCGCGGCGACG	tag29396
novel_sir5804	ACTTCACAAGCCTAGCGCCGCT	AAGTGTTCGGATCGCGGCGACG	tag64045
novel_sir5805	CGCCGACCCGCAGCCGGCCGCT	GGCTGGGCGTCGGCCGGCGACG	tag102614
novel_sir5806	GGGTGTCGGCGGCCGGCGAGG	CTCCCACAGCCGCCGGCCGCT	tag139812
novel_sir5807	CGATCGATGCCGCCGGCCGCT	TAGCTACGGCGGCCGGCGACG	tag172263
novel_sir5808	CCTTTTTCCGCGGCCGGCCGCT	AAAAAGGCGCCGGCCGGCGAGT	tag175019
novel_sir5809	CGGCGCTACCGGAACTGCCGCT	CGCGATGGCCTTGACGGCGAGC	tag116023
novel_sir5810	GTTAATTCCTGGCCAAGCGCT	ATTAAGGACCGGTTCGCGAGC	tag51475
novel_sir5811	CGTTAATTCCTGGCCAAGCGCT	AATTAAGGACCGGTTCGCGAGC	tag97528
novel_sir5812	TGAGTCCGGGACAAATCAAGCGCT	TCAGGCCCTGTTTAGTTCGCGAAA	tag88886
novel_sir5813	GTATCGTTGAGCTCGCGACG	CTCATAGCAACTCGAGCGCT	tag114586

novel_sir5814	AGTATCGTTGAGCTCGCGACG	ACTCATAGCAACTCGAGCGCT	tag237221
novel_sir5815	GGCCACCCGTGGCATTCTAGCGCT	GGTGGGCACCGTAAGATCGCGAAT	tag115544
novel_sir5816	AACTTTTCGGTCACCGCGCT	GAAAAGCCAGTGGCGCGAAG	tag180458
novel_sir5817	AACCAACTTTTCGGTCACCGCGCT	GGTTGAAAAGCCAGTGGCGCGAAG	tag259212
novel_sir5818	CACGATGGACGCAGCGGCGCGAGT	TTGTGCTACCTGCGTCGCCGCGCT	tag216728
novel_sir5819	TCCTTACGGATCATTCGCGCT	GAATGCCTAGTAAGCGCGAGT	tag76680
novel_sir5820	GATGACTACTGGCACGGCGCT	ACTGATGACCGTGCCGCGATA	tag199971
novel_sir5821	GAAGTCACCATAGGCGCCGCGAGC	TACTTCAGTGGTATCCGCGGCGCT	tag273960
novel_sir5822	CTCTGACTTCGACTCGGCGCT	GACTGAAGCTGAGCCGCGACA	tag83371
novel_sir5823	CGTCTGCTGAATTTATGCGCT	AGACGACTTAAATACGCGACG	tag169835
novel_sir5824	AAACTTGTAGCCCTCGACGCGATC	AGTTTGAACATCGGGAGCTGCGCT	tag93453
novel_sir5825	CGTCGGTTCGCAAGTATCGCT	AGCCAAGCGTTCATAGCGACG	tag249371
novel_sir5826	CCGTCGGTTCGCAAGTATCGCT	CAGCCAAGCGTTCATAGCGACG	tag180055
novel_sir5827	GATGTACAATATCTATCGCTCGCT	ACATGTTATAGATAGCGAGCGACA	tag97048
novel_sir5828	TGCTGCATGGGCAGCTGTCGCT	GACGTACCCGTCGACAGCGAGG	tag73592
novel_sir5829	CGCTGCATGGGCAGCTGTCGCT	GACGTACCCGTCGACAGCGAGG	tag186509
novel_sir5830	GCTCATATATCGGAACCGGCT	AGTATATAGCCTTGGCCGACA	tag181203
novel_sir5831	TGCTCATATATCGGAACCGGCT	GAGTATATAGCCTTGGCCGACA	tag97487
novel_sir5832	AATACCTCCTCTCTACCGGCT	ATGGAGGAGAGATGGCCGAGA	tag75057
novel_sir5833	CATACCTCCTCTCTACCGGCT	ATGGAGGAGAGATGGCCGAGA	tag268643
novel_sir5834	ATGGAGGAGAGATGGCCGAGC	CATACCTCCTCTCTACCGGCT	tag268643
novel_sir5835	CTCCTTTCATCCCCTTTACCGGCT	GGAAAGTAGGGGAAATGGCCGATA	tag93342
novel_sir5836	TGGTAGAAAGCAGCCAGCCGGCT	CATCTTTCGTCGGTCGGCCGATT	tag252116
novel_sir5837	TTGGTAGAAAGCAGCCAGCCGGCT	CCATCTTTCGTCGGTCGGCCGATT	tag98619
novel_sir5838	CCATCTTTCGTCGGTCGGCCGAAT	TTGGTAGAAAGCAGCCAGCCGGCT	tag98619
novel_sir5839	TTGGTGTAAAATAGCCAGCCGGCT	CCACATTTTATCGGTCGGCCGAAT	tag254734
novel_sir5840	CCCTGATAACACCTTTAGCCGGCT	GACTATTGTGGAAATCGGCCGACC	tag92082
novel_sir5841	CCTTAGATAGGCCGGCCGACC	CGGGAATCTATCCGGCCGGCT	tag62317
novel_sir5842	CCCTTAGATAGGCCGGCCGACC	ACGGGAATCTATCCGGCCGGCT	tag106781
novel_sir5843	GCCACCGAACCTGGAGCGGCT	GTGGCTTGGACCTCGCCGACG	tag16638
novel_sir5844	CGCCACCGAACCTGGAGCGGCT	GGTGGCTTGGACCTCGCCGACG	tag191859
novel_sir5845	GGAGCTGCGCCGCCGCCGACG	GTCCTCGACGCGGCGGCGGCT	tag262298
novel_sir5846	TGTCCTCGACGCGGCGGCGGCT	AGGAGCTGCGCCGCCGCCGACG	tag233115
novel_sir5847	AGGTGGACGCCGACGCCGACG	CGTCCACCTGCGGCTGCGGCT	tag10935

novel_sir5848	TCAAGCATGGACACAGCCGAGC	GAAGTTCGTACCTGTGTCGGCT	tag292925
novel_sir5849	AGCTTTACGTCCGCTATCCCGATC	TATCGAAATGCAGGCGATAGGGCT	tag118962
novel_sir5850	TGTGGATGCCTAGTCTGCGGGCT	ACCTACGGATCAGACGCCCGATC	tag206023
novel_sir5851	ATGTGGATGCCTAGTCTGCGGGCT	CACCTACGGATCAGACGCCCGATC	tag94740
novel_sir5852	TCGCTGTCGCTGTACCCGACG	CCAGCGACAGCGACATGGGCT	tag24249
novel_sir5853	CTAAACTATCGAGGTTGACCGAAT	GGGATTTGATAGCTCCAACTGGCT	tag77716
novel_sir5854	AGCCTACGCGTCGGGCGACCGATG	CCTCGGATGCGCAGCCCGCTGGCT	tag285931
novel_sir5855	TGCTAGAGATGACGCGCATTGGCT	GATCTCTACTGCGCGTAACCGAAC	tag221255
novel_sir5856	GGTACATATTGCTGTTTGGCT	ATGTATAACGACAAACCGATG	tag150693
novel_sir5857	TGGGCCCTAGGATCAGCCAATGCT	CCGGGATCCTAGTCGGTTACGATT	tag244761
novel_sir5858	ATCATTAAGTTGGATCATGCT	GTAATTCAACCTAGTACGAGA	tag173585
novel_sir5859	TATCATTAAGTTGGATCATGCT	AGTAATTCAACCTAGTACGAGA	tag32285
novel_sir5860	GCTCCTGACGTAGCTGGTGATGCT	AGGACTGCATCGACCACTACGAGC	tag149654
novel_sir5861	TAGGTCTATACCCATACGAGA	CGATCCAGATATGGGTATGCT	tag205488
novel_sir5862	ACGCCGGCGCCGGTGACGAGC	GGTGCGGCCGCGGCCACTGCT	tag157158
novel_sir5863	CCCAGCGTGGCATTTGGACGAAA	GAGGGTCGCACCGTAAACCTGCT	tag244066
novel_sir5864	ATAGGTGCCTGCTAACCTGCT	TCCACGGACGATTGGACGACG	tag48152
novel_sir5865	TCCACGAACGATTGGACGACG	ATAGGTGCTTGCTAACCTGCT	tag71599
novel_sir5866	CGGCTGCCAACTCCCCCTGCT	CGACGGTTGAGGGGGACGACC	tag28316
novel_sir5867	GGGTTGATACGTTTGGCCTGCT	CAACTATGCAAACCGGACGAAT	tag90606
novel_sir5868	CCGACAAAGTGGATCCTGCT	CTGTTTCACCTAGGACGAGG	tag134590
novel_sir5869	GCCACATGCTCGGCTCCTGCT	GTGTACGAGCCGAGGACGAAG	tag90697
novel_sir5870	ATCGATGCCGCCGGCCGCTGCT	GCTACGGCGGCCGGCGACGAAG	tag62111
novel_sir5871	CGATCGATGCCGCCGGCCGCTGCT	TAGCTACGGCGGCCGGCGACGAAG	tag168205
novel_sir5872	GGATGACGGCCGGCGACGAGA	TGCCTACTGCCGGCCGCTGCT	tag185711
novel_sir5873	CGGATGACGGCCGGCGACGAGA	CTGCCTACTGCCGGCCGCTGCT	tag252920
novel_sir5874	ACGCCTAGGACGACGACGACG	GCTGCGGATCCTGCTGCTGCT	tag129502
novel_sir5875	GGATCCGATAAAACACTCTGCT	TAGGCTATTTTGTGAGACGAAT	tag281882
novel_sir5876	GGGGATCCGATAAAACGCTCTGCT	CCTAGGCTATTTTGCGAGACGAAT	tag10873
novel_sir5877	AGCGAAGGTAGCTCTCAGACGAGC	CTTCGCTTCCATCGAGAGTCTGCT	tag172393
novel_sir5878	TGGTCTTTGTCCGAGTGCT	CAGAAACAGGCTCACGAAT	tag12910
novel_sir5879	TTGGTCTTTGTCCGAGTGCT	CCAGAAACAGGCTCACGAAT	tag213403
novel_sir5880	ATGCTAGTCGGCGCCACGAAC	GGTACGATCAGCCGCGGTGCT	tag113237
novel_sir5881	CATGCTAGTCGGCGCCACGAAC	TGGTACGATCAGCCGCGGTGCT	tag719

novel_sir5882	GTGCTAGCCGGCGCCACGAAC	GGCACGATCGGCCGCGGTGCT	tag171215
novel_sir5883	GGGGAGGAGCGGCACACGAGC	GCCCCCTCCTCGCCGTGTGCT	tag279053
novel_sir5884	ATCGCCAGGAATTTGAACATTGCT	GCGGTCCTTAAACTTGTAACGAGG	tag290134
novel_sir5885	ATCGCCAGGAATTTGAACATTGCT	GCGGTCCTTAAACTTGTAACGATG	tag290134
novel_sir5886	ACTGGCTGACGGTCGATTGCT	ACCGACTGCCAGCTAACGAGG	tag229087
novel_sir5887	CAGTCGACTGGGGAACGAGA	TCGTCAGCTGACCCCTTGCT	tag63302
novel_sir5888	TTCGTCAGCTGACCCCTTGCT	GCAGTCGACTGGGGAACGAGA	tag198844
novel_sir5889	TACCCTGTAAAGTATCATGTTGCT	GGGACATTTCATAGTACAACGAAT	tag274173
novel_sir5890	TGGCCGGTGTGAAACGGTGTTGCT	CGGCCACACTTTGCCACAACGAAG	tag128261
novel_sir5891	CTTGTTCAACCTCCTGGATTTGCT	ACAAGTTGGAGGACCTAAACGAGT	tag59231
novel_sir5892	TGAGCCCTCTACCCCGTTTGCT	TCGGGAGATGGGGCAAACGAAA	tag192747
novel_sir5893	ACCAGCCAGGGTACACAAACGACA	CCTGGTCGGTCCCATGTGTTTGCT	tag153226
novel_sir5894	CGAGTACCGCGAGTGCCAATCT	TCATGGCGCTCACGGTTAGATC	tag60231
novel_sir5895	CATACACACGCTGCGTCTTAGAGT	CAGTATGTGTGCGACGCAGAATCT	tag136978
novel_sir5896	CGGCGAACAAACAAATCAGAATCT	CGCTTGTTTGTTTAGTCTTAGACT	tag158882
novel_sir5897	CTTGGATTCTGACTTAGAGG	TCGAACCTAAGACTGAATCT	tag64833
novel_sir5898	CTTGGATTCTGACTTAGAGC	TCGAACCTAAGACTGAATCT	tag64833
novel_sir5899	CTTGGATTCTGACTTAGAGT	TCGAACCTAAGACTGAATCT	tag64833
novel_sir5900	CTTGGATTCTGACTTAGAGA	TCGAACCTAAGACTGAATCT	tag64833
novel_sir5901	AGCTTGGATTCTGACTTAGAGA	GATCGAACCTAAGACTGAATCT	tag22718
novel_sir5902	AGCTTGGATTCTGACTTAGAGG	GATCGAACCTAAGACTGAATCT	tag22718
novel_sir5903	TAGCTTGGATTCTGACTTAGAGA	CGATCGAACCTAAGACTGAATCT	tag72613
novel_sir5904	CTAGCTTGGATTCTGACTTAGAGG	ACGATCGAACCTAAGACTGAATCT	tag281168
novel_sir5905	TAGCCATAGAGTTACTGTACATCT	CGGTATCTCAATGACATGTAGAAC	tag6493
novel_sir5906	TAGCCATAGAGTTACTGTACATCT	CGGTATCTCAATGACATGTAGAAT	tag6493
novel_sir5907	TCCTCAGTAGCTCAGTGGTAGAGC	AAAGGAGTCATCGAGTCACCATCT	tag42244
novel_sir5908	TCCTCAGTAGCTCAGTGGTAGAGC	CAAGGAGTCATCGAGTCACCATCT	tag262221
novel_sir5909	AAAGGAGTCATCGAGTCACCATCT	TCCTCAGTAGCTCAGTGGTAGAGA	tag42244
novel_sir5910	CAAGGAGTCATCGAGTCACCATCT	TCCTCAGTAGCTCAGTGGTAGAGA	tag262221
novel_sir5911	TATGGTATATAAGGGTAGAAT	GTATACCATATATTCCCATCT	tag237217
novel_sir5912	TGTTTCAGTCACCGTTTAGCATCT	AAAGTCAGTGGCAAATCGTAGACA	tag76714
novel_sir5913	AAAGTCAGTGGCAAATCGTAGACG	TGTTTCAGTCACCGTTTAGCATCT	tag76714
novel_sir5914	TGTTTCAGTCACCGTTTAGCATCT	AAAGTCAGTGGCAAATCGTAGAGG	tag76714
novel_sir5915	AAAGTCAGTGGCAAATCGTAGAGC	TGTTTCAGTCACCGTTTAGCATCT	tag76714

novel_sir5916	AGGTAGCTTTAGGCTGCGCATCT	CATCGAAAATCCGACGCGTAGACG	tag53717
novel_sir5917	GAGCTGGCGTCGGAGTAGACG	TGCTCGACCGCAGCCTCATCT	tag129182
novel_sir5918	CACCATTCGTCACCGCCTAGATCT	GGTAAGCAGTGGCGGATCTAGAGG	tag100453
novel_sir5919	TGGAGCGGCAGGTATGAACGATCT	CTCGCCGTCCATACTTGCTAGAGC	tag120991
novel_sir5920	GTGGTGCAAGCGAACCCCCGATCT	CCACGTTCGCTTGGGGGCTAGAAA	tag131398
novel_sir5921	TCCCAACAAACTGTCCTAGAGC	GTAGGGTTGTTTGACAGGATCT	tag262223
novel_sir5922	GTATATCGAAGTCCTAGATA	AACATATAGCTTCAGGATCT	tag6073
novel_sir5923	TCTTCCTTGTTGCCTACCGGATCT	AAGGAACAACGGATGGCCTAGATT	tag51157
novel_sir5924	ATCGTAACGGGTGTAAGTATATCT	GCATTGCCCACATTCATATAGATG	tag258330
novel_sir5925	TTGGCTGTGACTCGGTCTCTATCT	CCGACACTGAGCCAGAGATAGAGT	tag122002
novel_sir5926	TAGGAGGCAGGACTAAAATAGAAT	TAATCCTCCGTCCTGATTTTATCT	tag280626
novel_sir5927	TACACCTGAGTGTACAGTAACTCT	GTGGACTCACATGTCATTGAGATA	tag940
novel_sir5928	GTGGGTCCACATGTCATTGAGATA	TACACCCAGGTGTACAGTAACTCT	tag125882
novel_sir5929	GTGGATCCACATGTCATTGAGATA	TACACCTAGGTGTACAGTAACTCT	tag43651
novel_sir5930	TACACCTGGGTGTACAGTAACTCT	GTGGACCCACATGTCATTGAGATA	tag262733
novel_sir5931	TACACTTGGGTGTACAGTAACTCT	GTGAACCCACATGTCATTGAGATA	tag30180
novel_sir5932	TCTGCTTACCAGTTTGTACACTCT	ACGAATGGTCAAACATGTGAGAAA	tag176008
novel_sir5933	ACGACGGTGCTAGGTGACTCT	CTGCCACGATCCACTGAGATC	tag205704
novel_sir5934	CGGACTAAGGCTCAGGCATACTCT	CTGATTCCGAGTCCGTATGAGAAA	tag99231
novel_sir5935	GGGCGACCGATGGGTTTGGAGAAA	AGCCCGCTGGCTACCCAAACCTCT	tag183917
novel_sir5936	TACCGAACCGATCAGTTAACCTCT	GGCTTGGCTAGTCAATTGGAGAGT	tag217471
novel_sir5937	GCTGCAATCCTTCAGGCCTCT	ACGTTAGGAAGTCCGGAGACG	tag59273
novel_sir5938	GACTTACATTGTGAAACGGAGAGA	TACTGAATGTAACACTTTGCCTCT	tag217126
novel_sir5939	TACTGAATGTAACACTTTGCCTCT	GACTTACATTGTGAAACGGAGAAA	tag217126
novel_sir5940	TAGTCGGGTTGCTGAATCCTCT	CAGCCCAACGACTTAGGAGAGG	tag218367
novel_sir5941	CGATTGTCGTCATCTTCCTCT	TAACAGCAGTAGAAGGAGAAG	tag231528
novel_sir5942	GCTCGACGCCGGGCCATCAGCTCT	AGCTGCGGCCCGGTAGTCGAGATG	tag104753
novel_sir5943	GACTGGAGGTATGAAGCTAGCTCT	GACCTCCATACTTCGATCGAGAAA	tag12898
novel_sir5944	TATGTAGTGTCCGCTGGCTCT	ACATCACAGGCGACCGAGAAA	tag16536
novel_sir5945	TAGACGCGAGAGGACGAGAAA	CTATCTGCGCTCTCCTGCTCT	tag147472
novel_sir5946	CCTACTGCCGGCCGCTGCTCT	ATGACGGCCGGCGACGAGAGG	tag132943
novel_sir5947	GCCTACTGCCGGCCGCTGCTCT	GATGACGGCCGGCGACGAGAGG	tag83256
novel_sir5948	CGTCAGCTGACCCCTTGCTCT	AGTCGACTGGGGAACGAGACC	tag229085
novel_sir5949	TCGTCAGCTGACCCCTTGCTCT	CAGTCGACTGGGGAACGAGACC	tag262091

novel_sir5950	GACCTGGGTAGCATCTCT	GGACCCATCGTAGAGACG	tag78998
novel_sir5951	TCTTTAATTTCCTTGATCTCT	AAATTAAAGGAACTAGAGATG	tag68701
novel_sir5952	GGGAACCGACACCTCTCT	CTTGGCTGTGGAGAGAGA	tag32631
novel_sir5953	GGGAACCGACACCTCTCT	CTTGGCTGTGGAGAGAGC	tag32631
novel_sir5954	CCGCGATGCCTCGTGCTCTCT	CGCTACGGAGCACGAGAGAAC	tag77228
novel_sir5955	CGTCCGGTTGTTGATAGTCTCT	AGGCCAACAACTATCAGAGAAC	tag75440
novel_sir5956	CGGTGCGCGCTCCTCCGTCTCT	CACGCGCGAGGAGGCAGAGAGT	tag219654
novel_sir5957	GCGGCCGGCCGAAGGGTCTCT	CCGGCCGGCTTCCCAGAGAAA	tag13924
novel_sir5958	CCGGCCGGCTTGCCCAGAGAAA	GCGGCCGGCCGAACGGGTCTCT	tag15552
novel_sir5959	CGTTGGTGGCCACGCTAAGAGATC	AAGCAACCACCGGTGCGATTCTCT	tag10988
novel_sir5960	GTAGTTCTGTGGTTTTTCTCT	TCAAGACACCAAAAAGAGAGG	tag166161
novel_sir5961	GGTTCCGGATGCTACTAAGTCT	AAGGCCTACGATGATTCAGACC	tag285342
novel_sir5962	TGGGTTCCGGATGCTACTAAGTCT	CCAAGGCCTACGATGATTCAGACC	tag29622
novel_sir5963	AGCTTGCAAACTGGCTTACAGTCT	GAACGTTTGACCGAATGTCAGAAG	tag153831
novel_sir5964	TGCAACGCCTACTAGAACCAGTCT	GTTGCGGATGATCTTGGTCAGATG	tag200888
novel_sir5965	CCGTCACAGGTATCTAGTCAGACG	CGGGCAGTGTCCATAGATCAGTCT	tag246506
novel_sir5966	GGCCGGCGGCCGACTCAGTCT	GGCCGCCGGCTGAGTCAGAAG	tag140699
novel_sir5967	TCAACATGGTATCAGAGC	TGAGTTGTACCATAGTCT	tag51319
novel_sir5968	TTTCGTGGCCTAGGGTAGTCT	AGCACCGGATCCCATCAGAAC	tag289399
novel_sir5969	ATTTCGTGGCCTAGGGTAGTCT	AAGCACCGGATCCCATCAGAAC	tag264407
novel_sir5970	TGATTTCGTGGCCTAGGGTAGTCT	TAAAGCACCGGATCCCATCAGAAC	tag56828
novel_sir5971	GGGCCAAGGATTTGAACGTCT	CGGTTCCTAAACTTGCAGATC	tag104772
novel_sir5972	GCCCCGTAACATTCACCGTCT	GGGCATTGTAAGTGGCAGAGT	tag160159
novel_sir5973	TGCCCCGTAACATTCACCGTCT	GGGGCATTGTAAGTGGCAGAGT	tag216719
novel_sir5974	GAGATGGAAGGGCAGAGA	ATCTCTACCTTCCCGTCT	tag29074
novel_sir5975	GAGATGGAAGGGCAGAGC	ATCTCTACCTTCCCGTCT	tag29074
novel_sir5976	AGAGATGGAAGGGCAGAGA	GTTCTCTACCTTCCCGTCT	tag13000
novel_sir5977	AGAGATGGAAGGGCAGAGC	GTTCTCTACCTTCCCGTCT	tag13000
novel_sir5978	CAAGAGATGGAAGGGCAGAGC	AGGTTCTCTACCTTCCCGTCT	tag171532
novel_sir5979	TAGGTTCTCTACCTTCCCGTCT	CCAAGAGATGGAAGGGCAGAGA	tag140207
novel_sir5980	CCAAGAGATGGAAGGGCAGAGC	TAGGTTCTCTACCTTCCCGTCT	tag140207
novel_sir5981	ATAGGTTCTCTACCTTCCCGTCT	TCCAAGAGATGGAAGGGCAGAGC	tag251070
novel_sir5982	AGGCGACCTACCACTCCGTCT	CGCTGGATGGTGAGGCAGAAT	tag85021
novel_sir5983	ACATGATATACTCTCGCCAGGTCT	TACTATATGAGAGCGGTCCAGAAT	tag166841

novel_sir5984	CTTATCAAAATGAGACCGGTCT	ATAGTTTTACTCTGGCCAGAGG	tag28525
novel_sir5985	TTTATCAAAATGAGACCGGTCT	ATAGTTTTACTCTGGCCAGAGG	tag228400
novel_sir5986	TGTCCATTGATCAGTGCCCGGTCT	AGGTAACTAGTCACGGGCCAGAAG	tag92974
novel_sir5987	TACACATACTATCCATCCTGGTCT	GTGTATGATAGGTAGGACCAGATA	tag39544
novel_sir5988	TGTGGATGTTTTAGCTTCTGGTCT	ACCTACAAAATCGAAGACCAGATA	tag228610
novel_sir5989	ACGCCTAGGACACCAGAAA	GATGCGGATCCTGTGGTCT	tag46185
novel_sir5990	ACGCCTAGGACACCAGAAA	AATGCGGATCCTGTGGTCT	tag268189
novel_sir5991	GATGCGGATCCTGTGGTCT	ACGCCTAGGACACCAGAAT	tag46185
novel_sir5992	AATGCGGATCCTGTGGTCT	ACGCCTAGGACACCAGAAT	tag268189
novel_sir5993	TACGCCTAGGACACCAGAAT	AGATGCGGATCCTGTGGTCT	tag281133
novel_sir5994	GAGAAGAGCAGCGGGCTACAGATT	CTCTCTTCTCGTCGCCCGATGTCT	tag125463
novel_sir5995	CTCTCTTCTCGTCGCCCGATGTCT	GAGAAGAGCAGCGGGCTACAGATC	tag125463
novel_sir5996	ATCACGGGACATTTCACAGAGA	GTTAGTGCCCTGTAAAGTGTCT	tag193196
novel_sir5997	CGAGCTTGAAAAATCACCGTGTCT	TCGAACTTTTTAGTGGCACAGAGG	tag164496
novel_sir5998	CGAGCTTGAAAAATCACCGTGTCT	TCGAACTTTTTAGTGGCACAGAGA	tag164496
novel_sir5999	CCTTCCTCCGGCCGCGTGTCT	AAGGAGGCCGGCGCACAGAGG	tag98832
novel_sir6000	GGTCCAGTAGCAAATTTTAAGAGT	ACCCAGGTCATCGTTTAAAATTCT	tag17178
novel_sir6001	TACTCTCGCCAGGTCTTACATTCT	GAGAGCGGTCCAGAATGTAAGAAT	tag239049
novel_sir6002	GTCTACACCGTATAGGATTCT	GATGTGGCATATCCTAAGACT	tag186102
novel_sir6003	GACTAGCTAAATCGGATGAAGAGT	AACTGATCGATTTAGCCTACTTCT	tag38523
novel_sir6004	AGTACACTTGAGATTACTTCT	ATGTGAACTCTAATGAAGATG	tag220285
novel_sir6005	ACTAAACATGGAAGGTGGAAGAGG	AGTGATTTGTACCTTCCACCTTCT	tag97570
novel_sir6006	GCCCGGCTAGGCCCGCCTTCT	GGCCGATCCGGGCGGAAGACA	tag130284
novel_sir6007	TGTCTCTTGTTGTCGCCTTCT	AGAGAACAACAGCGGAAGACA	tag236431
novel_sir6008	AACCAGTTTCATCGTAGAGCTTCT	GGTCAAAGTAGCATCTCGAAGATC	tag106349
novel_sir6009	CATGATATTGCATCGTTAGCTTCT	ACTATAACGTAGCAATCGAAGATG	tag275616
novel_sir6010	CCTCCACTTCCAACTAGTTCT	AGGTGAAGGTTGATCAAGATC	tag92120
novel_sir6011	GGTCCTCCACTTCCAACTAGTTCT	AGGAGGTGAAGGTTGATCAAGATC	tag96581
novel_sir6012	ATTGGTGAGTGACGCAAGAGA	CTTAACCACTCACTGCGTTCT	tag240218
novel_sir6013	AATTGGTGAGTGACGCAAGAGA	TCTTAACCACTCACTGCGTTCT	tag55925
novel_sir6014	CGCTGTAGCTGTGGTCGTTCT	GACATCGACACCAGCAAGAGC	tag189391
novel_sir6015	CGACATCGACACCAGCAAGAGC	CCGCTGTAGCTGTGGTCGTTCT	tag40649
novel_sir6016	ACCGTTGATGTGTTCCAAGATT	TGTGGCAACTACACAAGGTTCT	tag292689
novel_sir6017	AGCTGCAGCACGGACTCCAAGACA	GGTCGACGTCGTGCCTGAGGTTCT	tag5628

novel_sir6018	AGCTGCAGCACGGACTCCAAGACG	GGTCGACGTCGTGCCTGAGGTTCT	tag5628
novel_sir6019	AGCTGCAGCACGGACTCCAAGATG	GGTCGACGTCGTGCCTGAGGTTCT	tag5628
novel_sir6020	AGCTGCAGCACGGACTCCAAGAAG	GGTCGACGTCGTGCCTGAGGTTCT	tag5628
novel_sir6021	GGTCGACGTCGTGCCTGAGGTTCT	AGCTGCAGCACGGACTCCAAGATA	tag5628
novel_sir6022	GGGTAGGTTTATTGAAGGTGTTCT	CATCCAAATAACTTCCACAAGATA	tag120435
novel_sir6023	ACATGGTGACATGGGTGTTCT	TACCACTGTACCCACAAGACA	tag215675
novel_sir6024	GAATGTCGGCCGAACCTGTGTTCT	TACAGCCGGCTTGGACACAAGAAC	tag222272
novel_sir6025	CAGTGTATCCTGCTTCTGTGTTCT	CACATAGGACGAAGACACAAGAAG	tag94845
novel_sir6026	CGGTGTATCCTGCTTCTGTGTTCT	CACATAGGACGAAGACACAAGAAG	tag130992
novel_sir6027	AGTACATCTTGTCCTTGTTCT	ATGTAGAACAGGAACAAGAAC	tag147209
novel_sir6028	TTATGGTTGGCCCTGATTTCT	TACCAACCGGGACTAAAGATC	tag135339
novel_sir6029	CAATGGTTGGCCCTGATTTCT	TACCAACCGGGACTAAAGATC	tag193390
novel_sir6030	TAACACCAACCGGGACTAAAGATG	CCATTGTGGTTGGCCCTGATTTCT	tag177148
novel_sir6031	TAACACCAACCGGGACTAAAGATC	CCATTGTGGTTGGCCCTGATTTCT	tag177148
novel_sir6032	GTTTGGTCGGATGTCGGAAAGAGT	TGCAAACCAGCCTACAGCCTTTCT	tag113849
novel_sir6033	TACGAAGTACGTACACAGGTTTCT	GCTTCATGCATGTGTCCAAAGATT	tag44936
novel_sir6034	TACGAGGTACGTACACAGGTTTCT	GCTCCATGCATGTGTCCAAAGATT	tag60906
novel_sir6035	GCTCCATGCATATGTCCAAAGATT	TACGAGGTACGTATACAGGTTTCT	tag9248
novel_sir6036	GACCAAAGCGACCGGTAAAAGAGT	GACTGGTTTCGCTGGCCATTTTCT	tag118524
novel_sir6037	ATGAATTCAGGCTAAAAGAGT	CTTACTTAAGTCCGATTTTCT	tag59265
novel_sir6038	CCTAATTAATCCGAGTTTTCT	ATTAATTAGGCTCAAAAGATT	tag154414
novel_sir6039	CCTGATTAATCCGAGTTTTCT	ACTAATTAGGCTCAAAAGATT	tag34686
novel_sir6040	CTTGATTAATCCGAGTTTTCT	ACTAATTAGGCTCAAAAGATT	tag72460
novel_sir6041	TCTCGGTTGTAGGAATTAAAAAGT	AGCCAACATCCTTAATTTTTCACT	tag90595
novel_sir6042	TCGCGCCACTGGCTTTTCAAC	GAAGCGCGGTGACCGAAAAGT	tag203756
novel_sir6043	GCTTCGCGCCACTGGCTTTTCAAC	ATCGAAGCGCGGTGACCGAAAAGT	tag5135
novel_sir6044	GACTGCACCGTGGTTTTTAAAAGT	GACGTGGCACCAAAAATTTTCATT	tag250613
novel_sir6045	TAATCGTAGATCCGTTTCAGG	TTATTAGCATCTAGGCAAAGT	tag39518
novel_sir6046	CCTCGAGTCGGTTTGTCAAAGT	AGCTCAGCCAAACAGTTTCAGC	tag293558
novel_sir6047	ACCTCGAGTCGGTTTGTCAAAGT	GAGCTCAGCCAAACAGTTTCAGC	tag285769
novel_sir6048	GACCTCGAGTCGGTTTGTCAAAGT	GGAGCTCAGCCAAACAGTTTCAGC	tag233113
novel_sir6049	TTTTTTGGTCGCTTAGAAAGT	AAAACCAGCGAATCTTTCATG	tag172980
novel_sir6050	TCGATAGGCCACGCTTTCACG	CTAGCTATCCGGTGCGAAAGT	tag124113
novel_sir6051	ATCGATAGGCCACGCTTTCACG	CCTAGCTATCCGGTGCGAAAGT	tag32701

novel_sir6052	AAGTCGTTGGGCTGATTTCAGC	GATTCAGCAACCCGACTAAAGT	tag136959
novel_sir6053	CTTGTTGCCTACCGGATCTAAAGT	ACAACGGATGGCCTAGATTTCATC	tag180171
novel_sir6054	GTGACGTAGGTGAATTAAAGT	CTGCATCCACTTAATTTCAAT	tag197437
novel_sir6055	CTTGAATTTCTGTATTACAAGT	ACTTAAAGACATAATGTTCACT	tag214351
novel_sir6056	GTATGCCGCCCTCAGTAACCAAGT	TACGGCGGGAGTCATTGGTTCAAA	tag185459
novel_sir6057	TACATAGATACTGTACACCCAAGT	GTATCTATGACATGTGGGTTCAGT	tag248004
novel_sir6058	CGGAGATGGACGCGGTTCAGG	CAGCCTCTACCTGCGCCAAGT	tag215712
novel_sir6059	CTCCTCTCTACCGGCTCGCCAAGT	GGAGAGATGGCCGAGCGGTTCAAG	tag109155
novel_sir6060	GAGGCAACAACATCAGCAAGT	CCGTTGTTGTAGTCGTTCACG	tag5875
novel_sir6061	CTGACTTAGAGGCGTTCAGT	AAGACTGAATCTCCGCAAGT	tag268449
novel_sir6062	TCTGACTTAGAGGCGTTCAGT	TAAGACTGAATCTCCGCAAGT	tag86700
novel_sir6063	TATCGCGATGTCAGTTTCTCAAGT	AGCGCTACAGTCAAAGAGTTCAAT	tag74073
novel_sir6064	CATCACCTACGCAATCAGTTCAGC	GGGTAGTGGATGCGTTAGTCAAGT	tag191440
novel_sir6065	AGTCAAATGCATGGTGTCTTCATC	CGTCAGTTTACGTACCACAGAAGT	tag92905
novel_sir6066	GGCTAATTTGACTCGGTCTTCATC	CACCGATTAAACTGAGCCAGAAGT	tag112028
novel_sir6067	CACCGCCACAACTGGGTCAGAAGT	GGCGGTGTTGACCCAGTCTTCATC	tag239650
novel_sir6068	CTGGCACTATTCATCTTCAAG	GGGACCGTGATAAGTAGAAGT	tag157288
novel_sir6069	TCTCGAGCGCGTGGAGGAAGT	AGCTCGCGCACCTCCTTCATC	tag286916
novel_sir6070	GGGAGACTTCTTACTTCAAA	ACCCCTCTGAAGAATGAAGT	tag116815
novel_sir6071	TGCTCTCCTTGGCAACTAAGT	GAGAGGAACCGTTGATTCACA	tag156058
novel_sir6072	GGGTTCCGGATGCTACTAAGT	CAAGGCCTACGATGATTCAGA	tag155589
novel_sir6073	CCAAGGCCTACGATGATTCAGA	TGGGTTCCGGATGCTACTAAGT	tag90493
novel_sir6074	TGGAAAACATATCGTACTAAGT	CTTTTGTATAGCATGATTCATT	tag10719
novel_sir6075	CTATCGATGAATGGATTCACC	GTGATAGCTACTTACCTAAGT	tag100164
novel_sir6076	TCGTCTGCAAAGGATTCAGC	TCAGCAGACGTTTCCTAAGT	tag202053
novel_sir6077	ACCGGCTCTCTGTCCAGATTCATA	CCTGGCCGAGAGACAGGTCTAAGT	tag187997
novel_sir6078	CACCGCTTAACTATCATTCATT	CTGTGGCGAATTGATAGTAAGT	tag212712
novel_sir6079	CACCGCTTAACTATCATTCATA	CTGTGGCGAATTGATAGTAAGT	tag212712
novel_sir6080	ACAGGTTGCAAACTGGTAAGT	TCCAACGTTTGACCATTCATC	tag92567
novel_sir6081	CTAGCATTTCCCACATTCATA	AAGATCGTAAAGGGTGTAAGT	tag164914
novel_sir6082	AAGATCGTAACGGGTGTAAGT	CTAGCATTGCCCACATTCATT	tag258855
novel_sir6083	GGCACGGCGCTATCATTAAGT	GTGCCGCGATAGTAATTCAAC	tag199761
novel_sir6084	TGGCACGGCGCTATCATTAAGT	CGTGCCGCGATAGTAATTCAAC	tag171948
novel_sir6085	CGATCCGGTATGCTCTTAAGT	TAGGCCATACGAGAATTCATG	tag191594

novel_sir6086	GTTTGAACAAGCTGACACAGT	AACTTGTTCGACTGTGTCATC	tag135612
novel_sir6087	GGAGTTTGAACAAGCTGACACAGT	TCAAACTTGTTCGACTGTGTCATT	tag187314
novel_sir6088	GGAGTTTGAACAAGCTGACACAGT	TCAAACTTGTTCGACTGTGTCATA	tag187314
novel_sir6089	GGAGTTTGAACAAGCTGACACAGT	TCAAACTTGTTCGACTGTGTCATC	tag187314
novel_sir6090	CGATTTTTCAGTTGCCACAGT	TAAAAAGTCAACGGTGTCAAA	tag97963
novel_sir6091	GAATTTTTCAGTTGCCACAGT	TAAAAAGTCAACGGTGTCAAA	tag161996
novel_sir6092	TCAGGGCCAAGCTTACGACAGT	TCCCGGTTCGAATGCTGTCAGG	tag230880
novel_sir6093	TCTGGTTGTGTGGGCTGTCACC	GGAGACCAACACACCCGACAGT	tag205974
novel_sir6094	TTGGAGACCAACACACCCGACAGT	CCTCTGGTTGTGTGGGCTGTCACC	tag20879
novel_sir6095	GCAGTAGTTGAGGCTGTACAGT	TCATCAACTCCGACATGTCAAG	tag279803
novel_sir6096	GTGTCATAGGATCCACATGTCAGT	TACACAGTATCCTAGGTGTACAGT	tag108509
novel_sir6097	CCGCTCCACAGCCACAATGTCAGA	AAGGCGAGGTGTCGGTGTTACAGT	tag218757
novel_sir6098	GAGAAAATGGCCAGCGAAACCAGT	CTTTTACCGGTCGCTTTGGTCAGG	tag158813
novel_sir6099	AGGTAACAAGTCTTCGGGGTCACA	GGTCCATTGTTCAGAAGCCCCAGT	tag190571
novel_sir6100	TAGGCCAAAACACCAAATCCCAGT	CCGGTTTTGTGGTTTAGGGTCATG	tag217125
novel_sir6101	TAGGCCAAAACACCAAATCCCAGT	CCGGTTTTGTGGTTTAGGGTCACG	tag217125
novel_sir6102	GGTCATTTGGCTCTAAGGGTCATG	CGCCAGTAAACCGAGATTCCCAGT	tag106760
novel_sir6103	TTGTTGAATTCTGAATCAGCCAGT	CAACTTAAGACTTAGTCGGTCACG	tag198237
novel_sir6104	TACTCCATGGTCAGCGGTCAAG	CAATGAGGTACCAGTCGCCAGT	tag11297
novel_sir6105	ACTATCGCGGCACGGTCATC	AATGATAGCGCCGTGCCAGT	tag51447
novel_sir6106	AGGACACCAGATTAGTTTGCCAGT	CTGTGGTCTAATCAAACGGTCATT	tag76342
novel_sir6107	ATTACAAGTGAAGATGAGCAGT	ATGTTCACTTCTACTCGTCATC	tag45385
novel_sir6108	AGTACATTGATTACTTAGCAGT	ATGTAACTAATGAATCGTCATT	tag62934
novel_sir6109	TCTTTTTCAGTTGCCGCAGT	AAAAAGTCAACGGCGTCATC	tag192777
novel_sir6110	TTTTTTTCAGTTGCCGCAGT	AAAAAGTCAACGGCGTCATC	tag197679
novel_sir6111	ATTTTTTCAGTTGCCGCAGT	AAAAAGTCAACGGCGTCATC	tag240995
novel_sir6112	TAAAAAGTCAACGGCGTCATA	CGATTTTTCAGTTGCCGCAGT	tag131838
novel_sir6113	TAAAAAGTCAACGGCGTCATA	CAATTTTTCAGTTGCCGCAGT	tag181754
novel_sir6114	CGATTTTTCAGTTGCCGCAGT	TAAAAAGTCAACGGCGTCAAA	tag131838
novel_sir6115	CTATTTTTCAGTTGCCGCAGT	TAAAAAGTCAACGGCGTCAAA	tag136465
novel_sir6116	CAATTTTTCAGTTGCCGCAGT	TAAAAAGTCAACGGCGTCAAA	tag181754
novel_sir6117	TTCTTTTTCAGTTGCCGCAGT	GAAAAAGTCAACGGCGTCATC	tag114753
novel_sir6118	CTCTTTTTCAGTTGCCGCAGT	GAAAAAGTCAACGGCGTCATC	tag256992
novel_sir6119	ACTCTTTTTCAGTTGCCGCAGT	AGAAAAAGTCAACGGCGTCATC	tag248637

novel_sir6120	CACTCTTTTTCAGTTGCCGCAGT	GAGAAAAAGTCAACGGCGTCATC	tag185530
novel_sir6121	CCTCTCGTCGCCGGCCGTCATC	GTGGAGAGCAGCGGCCGGCAGT	tag155106
novel_sir6122	AAGCGAGAGCCCGCTGCCGGCAGT	CGCTCTCGGGCGACGGCCGTCAAC	tag275237
novel_sir6123	TGTACCGGCGCGAGCGGCAGT	ATGGCCGCGCTCGCCGTCAAG	tag134250
novel_sir6124	CGGAAGAGGCAGCCGATGCAGT	CTTCTCCGTCGGCTACGTCAGT	tag117313
novel_sir6125	ACGGAAGAGGCAGCCGATGCAGT	CCTTCTCCGTCGGCTACGTCAGT	tag84700
novel_sir6126	TCGTTGAGCTCGCGACGTCATC	ATAGCAACTCGAGCGCTGCAGT	tag18659
novel_sir6127	AGTTATAAAGTCAACGACGTCATA	AATCAATATTTCAGTTGCTGCAGT	tag271647
novel_sir6128	TCCTCCCCCGTTTGCCACGTCAGC	CAAGGAGGGGGCAAACGGTGCAGT	tag109286
novel_sir6129	CAGCTGGGTGGCAGACGGTGCAGT	CGACCCACCGTCTGCCACGTCAGC	tag143429
novel_sir6130	CAACACCGCTACGTCAACGTCATG	CAGTTGTGGCGATGCAGTTGCAGT	tag234446
novel_sir6131	TGCTGTGAAATACTAGAAATCAGT	GACACTTTATGATCTTTAGTCAGA	tag280743
novel_sir6132	AAAGGGCAGGGACGTAGTCAAC	TGTTTCCCGTCCCTGCATCAGT	tag103875
novel_sir6133	GAGAAGTAGGCTAAATCGATCAGT	CTTCATCCGATTTAGCTAGTCAAA	tag212464
novel_sir6134	GACACGAGTCGTAGCTGAGTCAGT	CGCTGTGCTCAGCATCGACTCAGT	tag231581
novel_sir6135	CTTATGGTGCTCGGGACTCAGT	ATACCACGAGCCCTGAGTCATC	tag188467
novel_sir6136	TACGGATCATTCGCGCTCAGT	GCCTAGTAAGCGCGAGTCATC	tag113782
novel_sir6137	CTCACGGTTTACAGACGAGTCATG	CAGAGTGCCAAATGTCTGCTCAGT	tag217070
novel_sir6138	CATGCGCACCGTCAGGTCAGT	ACGCGTGGCAGTCCAGTCAGC	tag278405
novel_sir6139	TACGCGTGGCAGTCCAGTCAGC	GCATGCGCACCGTCAGGTCAGT	tag256705
novel_sir6140	CGTACGCGTGGCAGTCCAGTCAGC	CTGCATGCGCACCGTCAGGTCAGT	tag112122
novel_sir6141	CACTACCGCTACATTTGTGTCAGT	GATGGCGATGTAAACACAGTCACC	tag41593
novel_sir6142	AGGCAAAGTGTTACATTCAGT	CGTTTCACAATGTAAGTCATT	tag283510
novel_sir6143	GGAGGCAAAGTGTTACATTCAGT	TCCGTTTCACAATGTAAGTCATT	tag39578
novel_sir6144	AGAGGCAAAGTGTTACATTCAGT	TCCGTTTCACAATGTAAGTCATT	tag200251
novel_sir6145	AGGAGGCAAAGTGTTACATTCAGT	CTCCGTTTCACAATGTAAGTCATT	tag165214
novel_sir6146	GGGAGGCAAAGTGTTACATTCAGT	CTCCGTTTCACAATGTAAGTCATT	tag250004
novel_sir6147	GTCCGTCACAGGCTAGAAGTCACT	CGCAGGCAGTGTCCGATCTTCAGT	tag15268
novel_sir6148	ACCAAGTGCACACCGGATTTCAGT	GTTCACGTGTGGCCTAAAGTCATA	tag212721
novel_sir6149	CCAGTTTGCAACCAATTTTTCAGT	TCAAACGTTGGTTAAAAAGTCAAT	tag12801
novel_sir6150	GTTTGTACACGATTTTTCAGT	AACATGTGCTAAAAAGTCAAC	tag44530
novel_sir6151	ATTTGTACACGATTTTTCAGT	AACATGTGCTAAAAAGTCAAC	tag116730
novel_sir6152	AGTTTGTACACGATTTTTCAGT	AAACATGTGCTAAAAAGTCAAC	tag226030
novel_sir6153	CAAACATGTGCTAAAAAGTCAAC	CAGTTTGTACACGATTTTTCAGT	tag92811

novel_sir6154	TCAAACATGTGCTAAAAAGTCAAC	TCAGTTTGTACACGATTTTTCAGT	tag231826
novel_sir6155	GTTTGTACACTCTTTTTCAGT	AACATGTGAGAAAAAGTCAAC	tag131218
novel_sir6156	AAACATGTGAGAAAAAGTCAAC	AGTTTGTACACTCTTTTTCAGT	tag116593
novel_sir6157	AGTTTGTACAAATTTTTTCAGT	AAACATGTTTAAAAAAGTCAAC	tag246006
novel_sir6158	TCAAACATGTTTAAAAAAGTCAAC	CTAGTTTGTACAAATTTTTTCAGT	tag179422
novel_sir6159	TTGCGGCAACTGAAAAAGAGT	CGCCGTTGACTTTTTCTCACA	tag142195
novel_sir6160	CTGCGGCAACTGAAAAAGAGT	CGCCGTTGACTTTTTCTCACA	tag224544
novel_sir6161	ACTGCGGCAACTGAAAAAGAGT	ACGCCGTTGACTTTTTCTCACA	tag184794
novel_sir6162	CTACTGCGGCAACTGAAAAAGAGT	TGACGCCGTTGACTTTTTCTCACA	tag188154
novel_sir6163	CTATTGCGGCAACTGAAAAAGAGT	TAACGCCGTTGACTTTTTCTCACA	tag212827
novel_sir6164	TGACGCCGTTAACTTTTTCTCACA	CTACTGCGGCAATTGAAAAAGAGT	tag213826
novel_sir6165	GTTGTGCGCACTCGGCAAGAGT	ACACGCGTGAGCCGTTCTCATC	tag19339
novel_sir6166	CGGAGTCCGGATGTCTGCGAGAGT	CTCAGGCCTACAGACGCTCTCAAC	tag30148
novel_sir6167	CACTGCCCGGATCAACAATAGAGT	GACGGGCCTAGTTGTTATCTCAAA	tag238572
novel_sir6168	CACTGCCCGGATCAACAATAGAGT	GACGGGCCTAGTTGTTATCTCAAT	tag238572
novel_sir6169	CTCCAACAGTCTACTTATCTCACT	TAGAGGTTGTCAGATGAATAGAGT	tag31262
novel_sir6170	GACGCGGGGCTGGATCTCAGT	CGCTGCGCCCCGACCTAGAGT	tag54671
novel_sir6171	CGACGCGGGGCTGGATCTCAGT	ACGCTGCGCCCCGACCTAGAGT	tag31235
novel_sir6172	CGACGCGGGGCTGGATCTCAGT	TCGCTGCGCCCCGACCTAGAGT	tag119128
novel_sir6173	AACTGTCCTAGAGCATCTCAAC	GTTTGACAGGATCTCGTAGAGT	tag69492
novel_sir6174	CTGGTCGAACGCCTAAACGAGT	CCAGCTTGCGGATTTGCTCACC	tag77027
novel_sir6175	GACTTTCTAGCATTGCTCACA	TTCTGAAAGATCGTAACGAGT	tag213671
novel_sir6176	AGGAAGAACGCGACCGACGAGT	CTTCTTGCGCTGGCTGCTCAGG	tag259618
novel_sir6177	CTGCAAAAGACCGAGGCTCACC	GAGACGTTTTCTGGCTCCGAGT	tag32645
novel_sir6178	CTCAGGCCTACAGACGCGCTCAAG	CAGAGTCCGGATGTCTGCGCGAGT	tag65631
novel_sir6179	CGATCCGGGAGCAAAGCTCAAG	GGGCTAGGCCCTCGTTTCGAGT	tag201654
novel_sir6180	TCCGCGCCGGCCCCAAGGAGT	GCGCGGCCGGGGTTCCTCACC	tag75420
novel_sir6181	GGTTAGTGCCAGGCTCAGGAGT	AATCACGGTCCGAGTCCTCATG	tag35354
novel_sir6182	CGGGCGACGAGAAGAGAGAGGAGT	CCGCTGCTCTTCTCTCTCCTCATT	tag38893
novel_sir6183	CTTCGACGTATGCAACGGAGT	AGCTGCATACGTTGCCTCACG	tag183265
novel_sir6184	ACCACTACGAGCACGCCTCAGC	GCTGGTGATGCTCGTGCGGAGT	tag126144
novel_sir6185	ATCTAAATCGTATCCCTCAAC	CTTAGATTTAGCATAGGGAGT	tag268311
novel_sir6186	GGACTGGGTGTACAGTAACTGAGT	TGACCCACATGTCATTGACTCATG	tag69015
novel_sir6187	GTTAGGCGGCGAAATCAGGTGAGT	ATCCGCCGCTTTAGTCCACTCAGC	tag15281

novel_sir6188	ATGCTACCGTAAAGAACCTTGAGT	CGATGGCATTTCTTGGAACTCACA	tag208355
novel_sir6189	GGCCCCACATGTCATCAACTCAAT	TACCGGGGTGTACAGTAGTTGAGT	tag84411
novel_sir6190	AGTTTGTAACTTATGCCTTTGAGT	AAACATTGAATACGGAAACTCACA	tag136677
novel_sir6191	TCTCTGCGTCGGCTTATCACG	GCAGAGACGCAGCCGAATAGT	tag5633
novel_sir6192	GGGCCAGAGAGTAACATAGATAGT	CGGTCTCTCATTGTATCTATCATA	tag276618
novel_sir6193	GGATCCGGAGTACTTGACTAGT	TAGGCCTCATGAACTGATCATG	tag220706
novel_sir6194	GTAGAACTCCGTAGTACTAGT	TCTTGAGGCATCATGATCAGT	tag22168
novel_sir6195	GTTAAAAATGTGGATGCCTAGT	ATTTTTACACCTACGGATCAGA	tag147777
novel_sir6196	GGTTAAAAATGTGGATGCCTAGT	AATTTTTACACCTACGGATCAGA	tag76945
novel_sir6197	AGGTTAAAAATGTGGATGCCTAGT	CAATTTTTACACCTACGGATCAGA	tag288759
novel_sir6198	CTCAATCCGTATAGGTATTCTAGT	GTTAGGCATATCCATAAGATCATC	tag162502
novel_sir6199	TATCGCGGCACGGTCATCAGT	TGATAGCGCCGTGCCAGTAGT	tag178218
novel_sir6200	TTAGAGAAGGACGATCAGTAGT	TCTCTTCCTGCTAGTCATCATT	tag59730
novel_sir6201	GGATCATTCGCGCTCAGTAGT	TAGTAAGCGCGAGTCATCAGC	tag283134
novel_sir6202	CGGATCATTCGCGCTCAGTAGT	CTAGTAAGCGCGAGTCATCAGC	tag288994
novel_sir6203	GCATATTGCGCGGCTAGCATCATT	CGCGTATAACGCGCCGATCGTAGT	tag220608
novel_sir6204	GCAGATTGCGCGGCTAGCATCATT	CGCGTCTAACGCGCCGATCGTAGT	tag258557
novel_sir6205	CGCGTTTAACGCGCCGATCGTAGT	GCAAATTGCGCGGCTAGCATCATT	tag155703
novel_sir6206	TTTCGTGGCCTAGGGTAGT	AGCACCGGATCCCATCAGA	tag233237
novel_sir6207	GATTTCGTGGCCTAGGGTAGT	AAAGCACCGGATCCCATCAGA	tag219335
novel_sir6208	TGATTTCGTGGCCTAGGGTAGT	TAAAGCACCGGATCCCATCAGA	tag54228
novel_sir6209	CCGTACTGAGCTGTCACCATCAGC	TGGGCATGACTCGACAGTGGTAGT	tag229497
novel_sir6210	GCACGAATCTTGGAACACATCAAC	GTCGTGCTTAGAACCTTGTGTAGT	tag137004
novel_sir6211	TCACTAATGGGATGAATCATA	CGAGTGATTACCCTACTTAGT	tag124651
novel_sir6212	CGGTACGGCTCTGTCATATTTAGT	CATGCCGAGACAGTATAAATCACT	tag222611
novel_sir6213	GCCACTCGGAGCCAGAAAACGT	GTGAGCCTCGGTCTTTTGCAGA	tag100930
novel_sir6214	TGTAGGGCTCGAGGCAAACGT	ATCCCGAGCTCCGTTTGCAAA	tag125560
novel_sir6215	GTGTAGGGCTCGAGGCAAACGT	CATCCCGAGCTCCGTTTGCAAA	tag200045
novel_sir6216	AGACCTCGATGTGCGCTTTGCAAG	TCTCTGGAGCTACACGCGAAACGT	tag229507
novel_sir6217	AACGCAGAACCTCAGGCACAACGT	GCGTCTTGGAGTCCGTGTTGCAGC	tag129662
novel_sir6218	CTGCTCACCAGTTTGCAACGT	CGAGTGGTCAAACGTTGCAAG	tag15830
novel_sir6219	CCCGGTGATCGGGTTTAACGT	GCCACTAGCCCAAATTGCAAA	tag224167
novel_sir6220	CATCCTTTGGTATCCTTGTGCACT	CGGTAGGAAACCATAGGAACACGT	tag286137
novel_sir6221	CGGGCACGACACGGGACACGT	CCGTGCTGTGCCCTGTGCAGC	tag59536

novel_sir6222	TCAGAATGGGGATAATTTACACGT	TCTTACCCCTATTAAATGTGCATG	tag211308
novel_sir6223	AGCTGGAGCGCGTCGTGCACC	GGTCGACCTCGCGCAGCACGT	tag95563
novel_sir6224	CTCCCTTCTTTCGGCCCGTGCAAA	CAGAGGGAAGAAAGCCGGGCACGT	tag42782
novel_sir6225	CGGGAAGAAGAGGTGGCACGT	CCTTCTTCTCCACCGTGCACG	tag115109
novel_sir6226	CCTAAACGAGTGATCTGCAAG	CTGGATTTGCTCACTAGACGT	tag125744
novel_sir6227	GTCTTGGAGTCCGTGCTGCAGC	CACAGAACCTCAGGCACGACGT	tag275930
novel_sir6228	GTGTCTTGGAGTCCGTGCTGCAGC	GACACAGAACCTCAGGCACGACGT	tag215645
novel_sir6229	GTGTCTTGGAGTCCGTGCTGCAGC	CACACAGAACCTCAGGCACGACGT	tag291437
novel_sir6230	GACACAGAACCTCAGGCACGACGT	GTGTCTTGGAGTCCGTGCTGCAAC	tag215645
novel_sir6231	CACACAGAACCTCAGGCACGACGT	GTGTCTTGGAGTCCGTGCTGCAAC	tag291437
novel_sir6232	GACGCAGAACCTCAGGCACGACGT	GCGTCTTGGAGTCCGTGCTGCAAC	tag146107
novel_sir6233	AACGCAGAACCTCAGGCACGACGT	GCGTCTTGGAGTCCGTGCTGCAAC	tag151857
novel_sir6234	GACGCAGAACCTCAGGCACGACGT	GCGTCTTGGAGTCCGTGCTGCAGC	tag146107
novel_sir6235	AACGCAGAACCTCAGGCACGACGT	GCGTCTTGGAGTCCGTGCTGCAGC	tag151857
novel_sir6236	ACGTCTTGGAGTCCGTGCTGCAGT	AATGCAGAACCTCAGGCACGACGT	tag32270
novel_sir6237	ACGTCTTGGAGTCCGTGCTGCAGC	AATGCAGAACCTCAGGCACGACGT	tag32270
novel_sir6238	AATGCAGAACCTCAGGCACGACGT	ACGTCTTGGAGTCCGTGCTGCAAC	tag32270
novel_sir6239	GTGTCTTAGAGTCCGTGCTGCAGG	AACACAGAATCTCAGGCACGACGT	tag211356
novel_sir6240	AACACAGAATCTCAGGCACGACGT	GTGTCTTAGAGTCCGTGCTGCAGT	tag211356
novel_sir6241	AACACAGAATCTCAGGCACGACGT	GTGTCTTAGAGTCCGTGCTGCAGC	tag211356
novel_sir6242	AATGCAGAACCTCAGGTACGACGT	ACGTCTTGGAGTCCATGCTGCAGC	tag168818
novel_sir6243	CGATGTCTAGACATCGGTCGACGT	TACAGATCTGTAGCCAGCTGCAGC	tag293560
novel_sir6244	TTGGAAGTGGAGGACCTTCGACGT	CCTTCACCTCCTGGAAGCTGCATA	tag285610
novel_sir6245	ATTCGACTAGGTTACTGCAAA	TTTAAGCTGATCCAATGACGT	tag205059
novel_sir6246	TCAGCACTCCGTTGCATACGT	TCGTGAGGCAACGTATGCAGC	tag262220
novel_sir6247	GTTGGCCTTGATTTCTAGCTACGT	ACCGGAACTAAAGATCGATGCATT	tag252218
novel_sir6248	GAGGACGAGAAATGTTGGCAAC	CTCTCCTGCTCTTTACAACCGT	tag23034
novel_sir6249	CGCTGGCTTGTGGTTGGCACG	GTGCGACCGAACACCAACCGT	tag13338
novel_sir6250	TTCTGCCTGTCAGTTTGCAACCGT	GACGGACAGTCAAACGTTGGCACG	tag115483
novel_sir6251	GTGTTCCGAGCGAACCACCGT	CAAGGCTCGCTTGGTGGCACG	tag292059
novel_sir6252	ATCTCAGTGGATCGTGGCAGC	CCTAGAGTCACCTAGCACCGT	tag58512
novel_sir6253	GATCTCAGTGGATCGTGGCAGC	ACCTAGAGTCACCTAGCACCGT	tag255794
novel_sir6254	CATTCAATATCAAGACGTGGCAGG	AAGTAAGTTATAGTTCTGCACCGT	tag268651
novel_sir6255	CTGCCCCGTAACATTCACCGT	CGGGGCATTGTAAGTGGCAGA	tag142183

novel_sir6256	TGATTCAGACCGAACTGGCAAC	CTACTAAGTCTGGCTTGACCGT	tag229084
novel_sir6257	CGGAACCCTAACGGCGATGGCATT	TTGCCTTGGGATTGCCGCTACCGT	tag83287
novel_sir6258	TGAAAGTCGTGCTCATGGCAAT	AAACTTTCAGCACGAGTACCGT	tag73160
novel_sir6259	GTTTGCGGAAGCTCATGGCAAG	CACAAACGCCTTCGAGTACCGT	tag54988
novel_sir6260	TCTGCCTGCCAGTTTGCAACCCGT	ACGGACGGTCAAACGTTGGGCACG	tag282714
novel_sir6261	ATTTATGCGCTGCCCCGT	AATACGCGACGGGGCATT	tag123945
novel_sir6262	CTGAATTTATGCGCTGCCCCGT	CTTAAATACGCGACGGGGCATT	tag267885
novel_sir6263	TGCTGAATTTATGCGCTGCCCCGT	GACTTAAATACGCGACGGGGCATT	tag160214
novel_sir6264	AGCGGCGGCTGCACAGGGCACA	CTTCGCCGCCGACGTGTCCCGT	tag248562
novel_sir6265	CCACGAGTGGCAATATCGGCATT	TGGGTGCTCACCGTTATAGCCGT	tag102503
novel_sir6266	GAATCTAAACACAGCCGCGGCAAC	CACTTAGATTTGTGTCGGCGCCGT	tag66439
novel_sir6267	GTCACGACGGTCAGCGGCAGC	GCCAGTGCTGCCAGTCGCCGT	tag189233
novel_sir6268	GGTCACGACGGTCAGCGGCAGC	AGCCAGTGCTGCCAGTCGCCGT	tag133411
novel_sir6269	CTGCTGAATCACTAGTGGCCGT	CGACTTAGTGATCACCGGCACC	tag8113
novel_sir6270	CGAGTGGGGCGGAGACGGCAGC	CCGCTCACCCCGCCTCTGCCGT	tag106873
novel_sir6271	AGTAGTTGCCGTCGGTGCCGT	ATCAACGGCAGCCACGGCATG	tag151633
novel_sir6272	CAGTAGTTGCCGTCGGTGCCGT	CATCAACGGCAGCCACGGCATG	tag279923
novel_sir6273	GGAGGCGACCTACCACTCCGT	TCCGCTGGATGGTGAGGCAGA	tag106623
novel_sir6274	GGCTGATGTTGTTGCCTCCGT	GACTACAACAACGGAGGCAAT	tag209328
novel_sir6275	TGCTGATGTTGTTGCCTCCGT	GACTACAACAACGGAGGCAAT	tag265308
novel_sir6276	TTGCTGATGTTGTTGCCTCCGT	CGACTACAACAACGGAGGCAAT	tag211656
novel_sir6277	GAAATGGTTTCTATGTCAGGCAAG	CTCTTTACCAAAGATACAGTCCGT	tag72679
novel_sir6278	GGTCGATCCGATCATAGTTTCCGT	AGCTAGGCTAGTATCAAAGGCATG	tag182281
novel_sir6279	TTAGCACTGTCATGGGAAGCGT	TCGTGACAGTACCCTTCGCACA	tag134149
novel_sir6280	CTCTAGGTCGGGGCGCAGCGT	GATCCAGCCCCGCGTCGCACG	tag90962
novel_sir6281	ACTCTAGGTCGGGGCGCAGCGT	AGATCCAGCCCCGCGTCGCACG	tag144432
novel_sir6282	TGTAGATGCACGTTTCAGCGT	ATCTACGTGCAAAGTCGCATC	tag94265
novel_sir6283	ACGGAGATCGTACAATAGCGT	CCTCTAGCATGTTATCGCATT	tag248447
novel_sir6284	GCCTCTAGCATGTTATCGCATT	TACGGAGATCGTACAATAGCGT	tag206700
novel_sir6285	CTAGTCAATCCGCTTTAGCGT	TCAGTTAGGCGAAATCGCAAG	tag7753
novel_sir6286	CGGCCGACACGCATGGCGCAGC	CCGCCGGCTGTGCGTACCGCGT	tag14845
novel_sir6287	TCCTGTCGGCGGCGGCGCACC	TGAGGACAGCCGCCGCCGCGT	tag169341
novel_sir6288	TCCTGTCGGCGGCGGCGCACC	CGAGGACAGCCGCCGCCGCGT	tag274855
novel_sir6289	CTCCTGTCGGCGGCGGCGCACC	TCGAGGACAGCCGCCGCCGCGT	tag250238

novel_sir6290	GCAGGCTCGTTCGGAAGCGCGT	TCCGAGCAAGCCTTCGCGCAAC	tag123928
novel_sir6291	ACAGCATCCTCTCGAGCGCGT	TCGTAGGAGAGCTCGCGCACC	tag115258
novel_sir6292	CTGAACGGTACTCGAAGGCGT	CTTGCCATGAGCTTCCGCAAA	tag132653
novel_sir6293	TAAGACCGTAGCTCGATAAAACGGCG	TCTGGCATCGAGCTATTTTGCCGCAGC	tag262796
novel_sir6294	TAAGACCGTAGCTCGATAAAACGGCG	TCTGGCATCGAGCTATTTTGCCGCAGA	tag262796
novel_sir6295	GAGTACAGAGGTGTACGGCGT	CATGTCTCCACATGCCGCATC	tag56049
novel_sir6296	TGTAGAGCTGATGGCCCGGCGT	ATCTCGACTACCGGGCCGCAGC	tag151312
novel_sir6297	TCTCAGTCTGGCACCCCGCAAT	GAAGAGTCAGACCGTGGGGCGT	tag39116
novel_sir6298	AGAATTGGTGAGTGACGCAAG	TCTCTTAACCACTCACTGCGT	tag131653
novel_sir6299	GAGAATTGGTGAGTGACGCAAG	GTCTCTTAACCACTCACTGCGT	tag229181
novel_sir6300	AATTACAAAGAGAGGACGCACT	TATTAATGTTTCTCTCCTGCGT	tag61403
novel_sir6301	CGTCGTGCCTGAGGTTCTGCGT	AGCACGGACTCCAAGACGCAGT	tag61000
novel_sir6302	CATCGTGCCTGAGGTTCTGCGT	AGCACGGACTCCAAGACGCAGT	tag282590
novel_sir6303	GTAGCACGGACTCCAAGACGCAAG	GACATCGTGCCTGAGGTTCTGCGT	tag41026
novel_sir6304	GACATCGTGCCTGAGGTTCTGCGT	GTAGCACGGACTCCAAGACGCAGC	tag41026
novel_sir6305	GTAGCACGGACTCCAAGACGCAGG	GACATCGTGCCTGAGGTTCTGCGT	tag41026
novel_sir6306	GACATCGTGCCTGAGGTTCTGCGT	GTAGCACGGACTCCAAGACGCAAT	tag41026
novel_sir6307	GACATCGTGCCTGAGGTTCTGCGT	GTAGCACGGACTCCAAGACGCAGT	tag41026
novel_sir6308	GTAGCACGGACTCCAAGACGCACC	GACATCGTGCCTGAGGTTCTGCGT	tag41026
novel_sir6309	GACGTCGTGCCTGAGGTTCTGCGT	GCAGCACGGACTCCAAGACGCAAG	tag178212
novel_sir6310	GACGTCGTGCCTGAGGTTCTGCGT	GCAGCACGGACTCCAAGACGCAAT	tag178212
novel_sir6311	GACGTCGTGCCTGAGGTTCTGCGT	GCAGCACGGACTCCAAGACGCAGT	tag178212
novel_sir6312	GACATCGTGTCTGAGGTTCTGCGT	GTAGCACAGACTCCAAGACGCAGT	tag78680
novel_sir6313	AGAACCCGTCGACTCGCACGCATG	AGTCTTGGGCAGCTGAGCGTGCGT	tag34121
novel_sir6314	AGATTAGGTTTCCGATTTTTGCGT	TAATCCAAAGGCTAAAAACGCAGG	tag243840
novel_sir6315	GACGTCGGTACTGTGTAGCATC	TTCTGCAGCCATGACACATCGT	tag177331
novel_sir6316	TGCTAAAACCTCACAGGACATCGT	GATTTTGGAGTGTCCTGTAGCAAA	tag233385
novel_sir6317	CTTCTCTCATGTACTAGTAGCACT	TGGAAGAGAGTACATGATCATCGT	tag210898
novel_sir6318	ATTATAAGACTTTCTAGCATT	TATAATATTCTGAAAGATCGT	tag141048
novel_sir6319	AATGTAAGTCATTCTAGCATT	TGTTACATTCAGTAAGATCGT	tag92930
novel_sir6320	TGTTATATTCAGTAAGATCGT	AATATAAGTCATTCTAGCATT	tag135812
novel_sir6321	GTCAAGCACCGCGGCCGATCGT	GTTCGTGGCGCCGGCTAGCACG	tag70622
novel_sir6322	GTCAAGCACCGCGGCTGATCGT	GTTCGTGGCGCCGACTAGCATG	tag212627
novel_sir6323	TAGATTGGTGTATCATTTTATCGT	CTAACCACATAGTAAAATAGCACT	tag256612

novel_sir6324	GCCAATACCCGCTCAACTCGT	GTTATGGGCGAGTTGAGCAAT	tag192737
novel_sir6325	CGCCAATACCCGCTCAACTCGT	GGTTATGGGCGAGTTGAGCAAT	tag44776
novel_sir6326	CGGTCCTTAATTGTCATGAGCACG	TGGCCAGGAATTAACAGTACTCGT	tag23863
novel_sir6327	CGGTCCTTAATTGCCATGAGCACG	TGGCCAGGAATTAACGGTACTCGT	tag180447
novel_sir6328	TAAGCGTGCTTGGCCTGGAGCAAT	CAATTCGCACGAACCGGACCTCGT	tag33930
novel_sir6329	TGCCCGGGCACCATGGAGCACC	CCACGGGCCCGTGGTACCTCGT	tag148320
novel_sir6330	CGCGGCGGCGGTGCGGCTCGT	GCCGCCGCCACGCCGAGCAAG	tag103292
novel_sir6331	CGCCGCCGCCACGCCGAGCAAG	TCGCGGCGGCGGTGCGGCTCGT	tag65987
novel_sir6332	GCAGCGTCGCTGCTGGCTCGT	TCGCAGCGACGACCGAGCAGA	tag198949
novel_sir6333	CATCGACACCAGCAAGAGCACG	CTGTAGCTGTGGTCGTTCTCGT	tag254946
novel_sir6334	CATGCGCACCGTCAGGTCAGTCGT	ACGCGTGGCAGTCCAGTCAGCACT	tag188084
novel_sir6335	CGCGCACCTCCTTCATCCAGCACC	GAGCGCGTGGAGGAAGTAGGTCGT	tag32037
novel_sir6336	CAGCCTACGCTAGTATGGTCGT	CGGATGCGATCATACCAGCACT	tag157533
novel_sir6337	TGCTAGAAGCCCACAGAGTGTCGT	GATCTTCGGGTGTCTCACAGCAAA	tag177700
novel_sir6338	TGTCCTCATCGCGATTGTCGT	AGGAGTAGCGCTAACAGCAGT	tag152735
novel_sir6339	TTGTCCTCATCGCGATTGTCGT	CAGGAGTAGCGCTAACAGCAGT	tag186263
novel_sir6340	CCAGATCACCGTTTAGCAATTCGT	TCTAGTGGCAAATCGTTAAGCACC	tag190849
novel_sir6341	GACCGAGCCATGAAGTTAAGCATG	TCCTGGCTCGGTACTTCAATTCGT	tag140293
novel_sir6342	AGTGGTCAAACGTTGCAAGCAAA	GCTCACCAGTTTGCAACGTTCGT	tag103971
novel_sir6343	TGGTGGATTCGGCGGTTGGTTCGT	CACCTAAGCCGCCAACCAAGCAGC	tag66651
novel_sir6344	GAGTGGTCAAACGTTACAAGCAAA	TGCTCACCAGTTTGCAATGTTCGT	tag183280
novel_sir6345	ATTGATCAACTCATGCCTTTTCGT	ACTAGTTGAGTACGGAAAAGCATG	tag86471
novel_sir6346	CCGCTGATTTCAACGACAAAGGT	CGACTAAAGTTGCTGTTTCCATT	tag144524
novel_sir6347	ACTTTTCCGTCACATCGTTCCAAT	GTTGAAAAGGCAGTGTAGCAAGGT	tag130125
novel_sir6348	CCAGGTGCCGCTGGTCAAGGT	TCCACGGCGACCAGTTCCAGC	tag166375
novel_sir6349	CAACGTATGCAGCTTCCAGG	CCGTTGCATACGTCGAAGGT	tag88199
novel_sir6350	GCAACGTATGCAGCTTCCAGG	TCCGTTGCATACGTCGAAGGT	tag272654
novel_sir6351	CGTACTCGCGGCGCTGGAAGGT	ATGAGCGCCGCGACCTTCCACG	tag3463
novel_sir6352	CATCGGGGTAGGTTTATTGAAGGT	AGCCCCATCCAAATAACTTCCACA	tag43430
novel_sir6353	TAATGAGCATTGGTAACAGGT	TACTCGTAACCATTGTCCACA	tag231938
novel_sir6354	AAACAAACAGATTTAGTGTCCAGG	CCTTTGTTTGTCTAAATCACAGGT	tag76188
novel_sir6355	TACACAGATACTGTACACCCAGGT	GTGTCTATGACATGTGGGTCCAGT	tag127994
novel_sir6356	GTCATAGACACATGTGGGTCCACA	TACAGTATCTGTGTACACCCAGGT	tag1941
novel_sir6357	GATAGAGTTACTGTATACCCAGGT	ATCTCAATGACATATGGGTCCACA	tag224177

novel_sir6358	GTGTCTATGACATGTAGGTCCAGT	TACACAGATACTGTACATCCAGGT	tag166033
novel_sir6359	CGGTTTTCGTATTGTTCCAGGT	CAAAAGCATAACAAGGTCCAGT	tag177283
novel_sir6360	GTACGGTGACGAAACAGCAGGT	TGCCACTGCTTTGTCGTCCATA	tag141382
novel_sir6361	CCCCACTAGTAGATAGCGTCCATA	CGGGGGTGATCATCTATCGCAGGT	tag115810
novel_sir6362	AGCGGCGCTGCGGCGGCAGGT	GCCGCGACGCCGCCGTCCACC	tag241931
novel_sir6363	CCGACATCCGGTCAAACGTCCAAT	AAGGCTGTAGGCCAGTTTGCAGGT	tag122266
novel_sir6364	TGCACCGCATACGCACCGTCAGGT	GTGGCGTATGCGTGGCAGTCCAGT	tag214980
novel_sir6365	TGCACCGCATGCGCACCGTCAGGT	GTGGCGTACGCGTGGCAGTCCAGT	tag187853
novel_sir6366	TCAGGTAACATCGGATTTTCAGGT	TCCATTGTAGCCTAAAAGTCCAAC	tag151590
novel_sir6367	ATTGCAGCTCAAAAGTCCAAC	GGTAACGTCGAGTTTTCAGGT	tag64639
novel_sir6368	CCCATTGCAGCTCAAAAGTCCAAC	TCGGGTAACGTCGAGTTTTCAGGT	tag63672
novel_sir6369	TACAGTCGCAGGCTAAAAAGAGGT	GTCAGCGTCCGATTTTTCTCCAAA	tag173970
novel_sir6370	CCGACTTCGTCCATCTCCATC	GGGGCTGAAGCAGGTAGAGGT	tag139736
novel_sir6371	GGGGGCTGAAGCAGGTAGAGGT	CCCGACTTCGTCCATCTCCATC	tag262683
novel_sir6372	AAGAAAAGGCCTTCGGTTAGAGGT	CTTTTCCGGAAGCCAATCTCCAAA	tag130851
novel_sir6373	TGCCCGTCTCAGCGCTCCACC	CGACGGGCAGAGTCGCGAGGT	tag133050
novel_sir6374	CTCGCCATCTACCGCTCCAGG	CGGAGCGGTAGATGGCGAGGT	tag242598
novel_sir6375	GAAACTGTTTGGCTGAGCTCCAAC	GACTTTGACAAACCGACTCGAGGT	tag67657
novel_sir6376	GAAACTGTTTGGCTGAGCTCCAGC	GACTTTGACAAACCGACTCGAGGT	tag67657
novel_sir6377	TGTCAAATACACAGTGTAGGAGGT	AGTTTATGTGTCACATCCTCCAAA	tag24644
novel_sir6378	CAGCTGCAGCACGGACTCCAAG	CGGTCGACGTCGTGCCTGAGGT	tag250883
novel_sir6379	TACACAGATACTGTACACCTAGGT	GTGTCTATGACATGTGGATCCAGT	tag203592
novel_sir6380	ATCATAAACACATGTGGATCCACA	TATAGTATTTGTGTACACCTAGGT	tag131130
novel_sir6381	CGGAACTTAATCGGATACCTAGGT	CTTGAATTAGCCTATGGATCCAAA	tag81729
novel_sir6382	CGCGTGGTCGAGGCCTAGGT	GCACCAGCTCCGGATCCAGC	tag95808
novel_sir6383	CCGCGTGGTCGAGGCCTAGGT	CGCACCAGCTCCGGATCCAGC	tag281932
novel_sir6384	ACCGCGTGGTCGAGGCCTAGGT	GCGCACCAGCTCCGGATCCAGC	tag18626
novel_sir6385	TACAATATCTGTGTATTCCTAGGT	GTTATAGACACATAAGGATCCATA	tag106954
novel_sir6386	CCGGAACGACGGTGCTAGGT	CCTTGCTGCCACGATCCACT	tag95031
novel_sir6387	ACCGGAACGACGGTGCTAGGT	GCCTTGCTGCCACGATCCACT	tag68258
novel_sir6388	CATGTGCTAGAAAAGCATCTAGGT	ACACGATCTTTTCGTAGATCCATT	tag61822
novel_sir6389	AATGTGCTAGAAAAGCATCTAGGT	ACACGATCTTTTCGTAGATCCATT	tag174529
novel_sir6390	TATGTGCTAGAAAAGCATCTAGGT	ACACGATCTTTTCGTAGATCCATT	tag185442
novel_sir6391	CGCCGACTCAGTCACACTCTAGGT	GGCTGAGTCAGTGTGAGATCCACG	tag178737

novel_sir6392	CGGTGCTAGGTGACTCTAGGT	CACGATCCACTGAGATCCAGC	tag57046
novel_sir6393	ACGGTGCTAGGTGACTCTAGGT	CCACGATCCACTGAGATCCAGC	tag292410
novel_sir6394	CAGTAATACGGTATGGCTCTAGGT	CATTATGCCATACCGAGATCCATC	tag256943
novel_sir6395	TCTCATATAGTACAGATCCATC	CGAGAGTATATCATGTCTAGGT	tag38837
novel_sir6396	GCTCTCATATAGTACAGATCCATC	GGCGAGAGTATATCATGTCTAGGT	tag255322
novel_sir6397	ACATTGGCCCAAGCATCCACA	ACTGTAACCGGGTTCGTAGGT	tag284754
novel_sir6398	AGCGGGCTAGGCATCGGGGTAGGT	GCCCGATCCGTAGCCCCATCCAAA	tag57720
novel_sir6399	TGCGGGCTAGGCATCGGGGTAGGT	GCCCGATCCGTAGCCCCATCCAAA	tag279897
novel_sir6400	CCGTTAAGACGGAGTGGTAGGT	CAATTCTGCCTCACCATCCAGC	tag83311
novel_sir6401	ACGCCCATTTCACATGTAGGT	CGGGTAAAGTGTACATCCACT	tag89285
novel_sir6402	TGCGGAGATTCAGTCTTAGGT	GCCTCTAAGTCAGAATCCAAG	tag55236
novel_sir6403	TTGCGGAGATTCAGTCTTAGGT	CGCCTCTAAGTCAGAATCCAAG	tag268014
novel_sir6404	CTGACGAAGTGATTAGTTAGGT	CTGCTTCACTAATCAATCCAAT	tag266635
novel_sir6405	GTGGCGTACGCGTGGCAATCCAGT	TGCACCGCATGCGCACCGTTAGGT	tag198523
novel_sir6406	CGGTGTGAAACGGTGTCAAACGGT	CACACTTTGCCACAGTTTGCCACG	tag188455
novel_sir6407	TAGCTAAAAATGTGACACAACGGT	CGATTTTTACACTGTGTTGCCATG	tag220788
novel_sir6408	GTGCTAAGGTGTGTACAACGGT	CGATTCCACACATGTTGCCATG	tag292939
novel_sir6409	AAAGAGCATTATAGGAACGGT	TCTCGTAATATCCTTGCCAAC	tag223864
novel_sir6410	AACCGGTCCTTAATTGCCATG	GCTTGGCCAGGAATTAACGGT	tag191126
novel_sir6411	CTTAAAAGAAGCAAGGCACGGT	ATTTTCTTCGTTCCGTGCCACT	tag185800
novel_sir6412	TTCGGGGGTTCCCGGGCACGGT	GCCCCCAAGGGCCCGTGCCACC	tag144739
novel_sir6413	GGATGAAAGAATTATGGGCACGGT	TACTTTCTTAATACCCGTGCCAGC	tag190322
novel_sir6414	TACTTTCTTAATACCCGTGCCAAC	GGATGAAAGAATTATGGGCACGGT	tag190322
novel_sir6415	GTACGAACTTCTACTTATCACGGT	TGCTTGAAGATGAATAGTGCCAGG	tag141827
novel_sir6416	CAGCAAGGCCACTCTGCCACT	CCGTCGTTCCGGTGAGACGGT	tag203160
novel_sir6417	TATGAACGCTTGGCTGCCACA	CGATACTTGCGAACCGACGGT	tag8111
novel_sir6418	TGCCTCCTCGCGCGTGGCCAAG	AGACGGAGGAGCGCGCACCGGT	tag239951
novel_sir6419	AATAGTTTTACTCTGGCCAGA	GCTTATCAAAATGAGACCGGT	tag101585
novel_sir6420	TAAGCTTATCAAAATGAGACCGGT	TCGAATAGTTTTACTCTGGCCAGA	tag265049
novel_sir6421	GTTAAGGCTCAAGTTTACCGGT	ATTCCGAGTTCAAATGGCCAAG	tag74717
novel_sir6422	CCGCGCGTCGTCCGCCCCCGGT	CGCGCAGCAGGCGGGGGCCAGC	tag232942
novel_sir6423	ACCATCCAGCGGAGGGCCATT	AGTGGTAGGTCGCCTCCCGGT	tag85655
novel_sir6424	CTTGACGGCGAGCGCGGCCATG	CGGAACTGCCGCTCGCGCCGGT	tag267005
novel_sir6425	TGGGTCTACACCCTCTAATCCGGT	CCAGATGTGGGAGATTAGGCCATT	tag177912

novel_sir6426	TCTTTAATGAATGGTATCCGGT	AAATTACTTACCATAGGCCACG	tag125679
novel_sir6427	GCGTTCAACGCGGGCTCCGGT	CAAGTTGCGCCCGAGGCCATC	tag213304
novel_sir6428	CGGCGCGAGCGGCAGTTCCGGT	CGCGCTCGCCGTCAAGGCCATC	tag273013
novel_sir6429	TGGTGGGTGTGCGGCTGTAGCGGT	CACCCACACGCCGACATCGCCAAC	tag157419
novel_sir6430	GATCCGGAGCTGGTGCGCCAGG	ACCTAGGCCTCGACCACGCGGT	tag120362
novel_sir6431	TACCTCGACACCGGCGCCACC	CCATGGAGCTGTGGCCGCGGT	tag157852
novel_sir6432	ACACGGTAGCTTCGCGCCACT	CGTGTGCCATCGAAGCGCGGT	tag159269
novel_sir6433	CACACGGTAGCTTCGCGCCACT	CCGTGTGCCATCGAAGCGCGGT	tag119944
novel_sir6434	TGGTGGGTGTGTGGCTGTCGCGGT	CACCCACACACCGACAGCGCCAAC	tag45387
novel_sir6435	ACCACCCTCTAGATCCGCCACT	CCTGGTGGGAGATCTAGGCGGT	tag198660
novel_sir6436	GACCACCCTCTAGATCCGCCACT	GCCTGGTGGGAGATCTAGGCGGT	tag115068
novel_sir6437	TCCTCCGTTGAATCCGCCACA	GAAGGAGGCAACTTAGGCGGT	tag102786
novel_sir6438	GATGCGACGGGCGCCGCCACC	GGCTACGCTGCCCGCGGCGGT	tag121780
novel_sir6439	ACGAGGCATCGCGGCGGCGGT	CTCCGTAGCGCCGCCGCCACG	tag32887
novel_sir6440	GTCCAAGCCACCGCCGCCACC	TCCAGGTTCGGTGGCGGCGGT	tag6243
novel_sir6441	TCCCAAGGATAGCTTCCCGCCATT	CAAGGGTTCCTATCGAAGGGCGGT	tag86346
novel_sir6442	AAAGACGAATTGCCGGGCGGT	TCTGCTTAACGGCCCGCCAAC	tag172927
novel_sir6443	CATCCCCACTATTTTCTAGCCAAT	TGGTAGGGGTGATAAAAGATCGGT	tag238517
novel_sir6444	AATTCTTAAATCTCAGATCGGT	AAGAATTTAGAGTCTAGCCAAG	tag109883
novel_sir6445	ACCAAACAGTCAACTGGAGCCATC	TTTGGTTTGTCAGTTGACCTCGGT	tag28485
novel_sir6446	ACTAGCAATAACTAGGTCGGT	ATCGTTATTGATCCAGCCACA	tag223964
novel_sir6447	TATGTATCCCAACCAGCCATG	ATATACATAGGGTTGGTCGGT	tag245791
novel_sir6448	CCGGTTTCTGACAGCTGTCGGT	CCAAAGACTGTCGACAGCCACG	tag3346
novel_sir6449	CCTGGGCTGTCACTGTGTCGGT	ACCCGACAGTGACACAGCCACC	tag74053
novel_sir6450	GAGAGGTTGGCCTATCGATTCGGT	CTCCAACCGGATAGCTAAGCCATC	tag97320
novel_sir6451	CTCCATCCCACTTTCCAAGCCAAA	GAGAGGTAGGGTGAAAGGTTCGGT	tag224855
novel_sir6452	GAATCCGTTTAGATTGAAAGCCAAA	AACTTAGGCAAATCTAACTTTCGGT	tag249248
novel_sir6453	TGACGCAAGAGAAAAGCCACT	TCACTGCGTTCTCTTTTCGGT	tag130764
novel_sir6454	TAGCGCTACTCCTGTTCCCATT	CAATCGCGATGAGGACAAGGGT	tag281696
novel_sir6455	GTGGAGCAAAACGCATAGAAGGGT	CCTCGTTTTGCGTATCTTCCCAAT	tag256007
novel_sir6456	GCGGAGCTTCGAGGGAAGGGT	CCTCGAAGCTCCCTTCCCAAC	tag107230
novel_sir6457	GGCGGAGCTTCGAGGGAAGGGT	GCCTCGAAGCTCCCTTCCCAAC	tag213632
novel_sir6458	GATGCAACACGAGGACTTCCCAGG	CCCTACGTTGTGCTCCTGAAGGGT	tag274070
novel_sir6459	CGCGATCTCGGTACCAGTCCCAGG	TGGCGCTAGAGCCATGGTCAGGGT	tag109177

novel_sir6460	GAAGTAAGAAGTCTCCCAGA	AACTTCATTCTTCAGAGGGT	tag104438
novel_sir6461	GTGATTTCGTGGCCTAGGGT	CTAAAGCACCGGATCCCATC	tag97072
novel_sir6462	CGTGATTTCGTGGCCTAGGGT	ACTAAAGCACCGGATCCCATC	tag252620
novel_sir6463	TCGTGATTTCGTGGCCTAGGGT	CACTAAAGCACCGGATCCCATC	tag149386
novel_sir6464	GTTTTCCATTTCGGCAACGGGT	AAAGGTAAAGCCGTTGCCCACG	tag237106
novel_sir6465	ATGTTTTCCATTTCGGCAACGGGT	CAAAAGGTAAAGCCGTTGCCCACG	tag172024
novel_sir6466	ACATTGCTCGGCTTGCCCATA	CATGTAACGAGCCGAACGGGT	tag48412
novel_sir6467	CCATGTAACGAGCCGAACGGGT	TACATTGCTCGGCTTGCCCATA	tag2030
novel_sir6468	ACGAAGTAGAGTTGGCCCAGC	CGTGCTTCATCTCAACCGGGT	tag18757
novel_sir6469	CTCACTCTTTCTCTATGGCCCAGA	TAGAGTGAGAAAGAGATACCGGGT	tag6833
novel_sir6470	GCTCCAGGATCGGGATGGCCCAAC	GACGAGGTCCTAGCCCTACCGGGT	tag257856
novel_sir6471	CGGAGGTCCACGGTACCGGGT	CTCCAGGTGCCATGGCCCACG	tag219291
novel_sir6472	AAGTGGCCCACGTCGGCCCAGT	GGTTCACCGGGTGCAGCCGGGT	tag41550
novel_sir6473	ATAGGAGACTCGATGTCCGGGT	TCCTCTGAGCTACAGGCCCACC	tag106411
novel_sir6474	TTAGGAGACTCGATGTCCGGGT	TCCTCTGAGCTACAGGCCCACC	tag133930
novel_sir6475	ATCCTAGTACTACTCTCGCCCAAG	GGTAGGATCATGATGAGAGCGGGT	tag209766
novel_sir6476	AGACCGCCGGCTTGCGCCCATG	CCTCTGGCGGCCGAACGCGGGT	tag65354
novel_sir6477	ATAGAAAAGTCGATCGCGGGT	TCTTTTCAGCTAGCGCCCACT	tag266183
novel_sir6478	TGGATAGAAAAGTCGATCGCGGGT	CTATCTTTTCAGCTAGCGCCCACT	tag101928
novel_sir6479	CCGTCGCCGGCCGACGCCCAGC	GCGGCAGCGGCCGGCTGCGGGT	tag38888
novel_sir6480	TACGTTATTTGCCTATCTGCGGGT	GCAATAAACGGATAGACGCCCACT	tag20375
novel_sir6481	AGGAGGAGAAAGCAAACGGGGT	CTCCTCTTTCGTTTGCCCCATC	tag171842
novel_sir6482	CGGAATGGTGAACCGGTGCGGGGT	CTTACCACTTGGCCACGCCCCATT	tag117563
novel_sir6483	AGGAATGGTGAACCGGTGCGGGGT	CTTACCACTTGGCCACGCCCCATT	tag292961
novel_sir6484	TGTCTGCGGGCTAGGCATCGGGGT	AGACGCCCGATCCGTAGCCCCACC	tag32102
novel_sir6485	AGTCTGCGGGCTAGGCATCGGGGT	AGACGCCCGATCCGTAGCCCCACC	tag235548
novel_sir6486	TGTCTGCGGGCTAGGCATCGGGGT	AGACGCCCGATCCGTAGCCCCATC	tag32102
novel_sir6487	AGTCTGCGGGCTAGGCATCGGGGT	AGACGCCCGATCCGTAGCCCCATC	tag235548
novel_sir6488	GAGCTGCTGTCCTCGTGGGGT	CGACGACAGGAGCACCCCAGG	tag278574
novel_sir6489	CGAGCTGCTGTCCTCGTGGGGT	TCGACGACAGGAGCACCCCAGG	tag46283
novel_sir6490	GGTGTTTGATTCGGAATGGGT	ACAAACTAAGCCTTACCCACT	tag123474
novel_sir6491	CCGTTCGGCTCGTTACATGGGT	CAAGCCGAGCAATGTACCCAAG	tag43115
novel_sir6492	TGAGGCGATCCAGATATGGGT	TCCGCTAGGTCTATACCCATA	tag9805
novel_sir6493	GTCGATCGCGGGTGACTTATGGGT	GCTAGCGCCCACTGAATACCCATA	tag125989

novel_sir6494	CGTCACCGATAGCTTTGACCCAGA	GGGCAGTGGCTATCGAAACTGGGT	tag240400
novel_sir6495	GGACGCCGAATTAAACTGGGT	TGCGGCTTAATTTGACCCAAC	tag83962
novel_sir6496	CGGACGCCGAATTAAACTGGGT	CTGCGGCTTAATTTGACCCAAC	tag123772
novel_sir6497	TACAGTATCTGTGTACACCTGGGT	GTCATAGACACATGTGGACCCACA	tag23487
novel_sir6498	CGGGCGGCGGGCGGGGCTGGGT	CCGCCGCCCGCCCCGACCCACG	tag211674
novel_sir6499	GAGGTGTTGAGGTGAGGTCTGGGT	CCACAACTCCACTCCAGACCCAAC	tag29934
novel_sir6500	TCACAACTCCACTCTAAACCCAAC	GAAGTGTTGAGGTGAGATTTGGGT	tag209751
novel_sir6501	CGCAGGCTAAAAAGAGGTTTGGGT	GTCCGATTTTTCTCCAAACCCATC	tag85438
novel_sir6502	CACTTTTTTGGCAACTTTACCAAA	CGGTGAAAAAACCGTTGAAATGGT	tag228618
novel_sir6503	CTAGATCCGCCACTGCTTACCACC	GAGATCTAGGCGGTGACGAATGGT	tag79705
novel_sir6504	CTAGATCCGCCACTGCTTACCACT	GAGATCTAGGCGGTGACGAATGGT	tag79705
novel_sir6505	GGTAAGATGGGAATATATGGT	ATTCTACCCTTATATACCATA	tag198243
novel_sir6506	AACCACGTGCCACATACCACC	CATTGGTGCACGGTGTATGGT	tag60753
novel_sir6507	CTGAAAAAGAGTGTACAAACTGGT	CTTTTTCTCACATGTTTGACCATT	tag77643
novel_sir6508	CTGAAAAACAGGTTGCAAACTGGT	CTTTTTGTCCAACGTTTGACCATT	tag126689
novel_sir6509	CTGAAAAATTGGTTGCAAACTGGT	CTTTTTAACCAACGTTTGACCACT	tag150374
novel_sir6510	TCAACCAAGCGCGATGACCAAT	AAAGTTGGTTCGCGCTACTGGT	tag181229
novel_sir6511	GAGTGTATGATAGGTGGGACCAGA	TACTCACATACTATCCACCCTGGT	tag240002
novel_sir6512	GACACACATACTGTCCACCCTGGT	GTGTGTATGACAGGTGGGACCATA	tag50620
novel_sir6513	CACACACATACTGTCCACCCTGGT	GTGTGTATGACAGGTGGGACCATA	tag182326
novel_sir6514	TACACACATACTGTCCACCCTGGT	GTGTGTATGACAGGTGGGACCATA	tag223075
novel_sir6515	GATGTAGTTGTGGTTCCTGGT	ACATCAACACCAAGGACCAGC	tag64963
novel_sir6516	TACATCAACACCAAGGACCAGC	CGATGTAGTTGTGGTTCCTGGT	tag288964
novel_sir6517	TCGAGGACTGCATCGACCACT	GTAGCTCCTGACGTAGCTGGT	tag288307
novel_sir6518	TGTGGTCGCTCCCATCGCGCTGGT	ACCAGCGAGGGTAGCGCGACCAAA	tag215704
novel_sir6519	TCTTCTGATCCCGCTGCTGGT	AAGACTAGGGCGACGACCACA	tag216201
novel_sir6520	GAAGACTAGGGCGACGACCACA	GTCTTCTGATCCCGCTGCTGGT	tag33317
novel_sir6521	TAGTCTGGCTGGCGGCGATCTGGT	CAGACCGACCGCCGCTAGACCAAC	tag215195
novel_sir6522	CACGCGTACGCTATATAGACCAAA	CGGTGCGCATGCGATATATCTGGT	tag212453
novel_sir6523	CAGCTAAATGCTCAAATCTCTGGT	CGATTTACGAGTTTAGAGACCAGG	tag218371
novel_sir6524	GTCGCTAGCTAGCCGAAGTGGT	GCGATCGATCGGCTTCACCACT	tag91536
novel_sir6525	GCTCTAAGCAGGTGCAGTGGT	AGATTCGTCCACGTCACCATG	tag288540
novel_sir6526	AAAGAGAACGCAGTGAGTGGT	TCTCTTGCGTCACTCACCAAT	tag293342
novel_sir6527	CGTGGAGGAGGACCGCGTGGT	ACCTCCTCCTGGCGCACCAGC	tag86582

novel_sir6528	TCCTGTCGGCGGCGGCACCACC	CGAGGACAGCCGCCGCGTGGT	tag216833
novel_sir6529	ACCGCAGTAGAGTTTAACCACCAAA	AGTGGCGTCATCTCAAATTGGTGGT	tag194610
novel_sir6530	TAGCTAGAATACAAATGATGTGGT	CGATCTTATGTTTACTACACCATC	tag215025
novel_sir6531	AAAAGCCACTTATACACCAGT	TCTTTTCGGTGAATATGTGGT	tag115169
novel_sir6532	CTACGCCTAGGACACCAGA	GAGATGCGGATCCTGTGGT	tag283390
novel_sir6533	TCTACGCCTAGGACACCAGA	GGAGATGCGGATCCTGTGGT	tag34419
novel_sir6534	AGGAGATGCGGATCCTGTGGT	CTCTACGCCTAGGACACCAGA	tag162597
novel_sir6535	CCGCTCGATGTAGTTGTGGT	CGAGCTACATCAACACCAAG	tag39535
novel_sir6536	ACCCATCTTTAAATTCTAACCATT	GGTGGGTAGAAATTTAAGATTGGT	tag166801
novel_sir6537	CCGGATCCGCATCTCCTTGGT	CCTAGGCGTAGAGGAACCACC	tag72135
novel_sir6538	CCGGATCCGCATCTCCTTGGT	CCTAGGCGTAGAGGAACCACA	tag72135
novel_sir6539	CCGCTTTCTGATTAGCTTGGT	CGAAAGACTAATCGAACCATC	tag189981
novel_sir6540	CGCCACTGGCTTTTCAACCAAG	GCGCGGTGACCGAAAAGTTGGT	tag250421
novel_sir6541	GCGCCACTGGCTTTTCAACCAAG	AGCGCGGTGACCGAAAAGTTGGT	tag28096
novel_sir6542	CGCGCCACTGGCTTTTCAACCAAG	AAGCGCGGTGACCGAAAAGTTGGT	tag158046
novel_sir6543	TAGTGTAGCAAGATTAAAGTTGGT	CACATCGTTCTAATTTCAACCAAA	tag83826
novel_sir6544	CACATCGTTTCAATTTCAACCAAA	CAGTGTAGCAAAGTTAAAGTTGGT	tag147193
novel_sir6545	CAGTGTAGCAAGGTTAAAGTTGGT	CACATCGTTCCAATTTCAACCAAA	tag108863
novel_sir6546	TAGTGTAGCAAGGTTAAAGTTGGT	CACATCGTTCCAATTTCAACCAAA	tag206352
novel_sir6547	GTTCTGGCCTCGATAGTTGGT	AGACCGGAGCTATCAACCACT	tag203612
novel_sir6548	TGCCCGTTACCCGTACCCGTTGGT	GGGCAATGGGCATGGGCAACCATC	tag157979
novel_sir6549	ACATTAACCGTGCCAACCACA	TTTGTAATTGGCACGGTTGGT	tag77383
novel_sir6550	ACATTAACCGTGCCAACCACA	TCTGTAATTGGCACGGTTGGT	tag146901
novel_sir6551	CAGTTTGCAACCTGTGCCTTTGGT	CAAACGTTGGACACGGAAACCAGG	tag271842
novel_sir6552	CAGTTTGTAACCTGTGCCTTTGGT	CAAACATTGGACACGGAAACCAGG	tag232944
novel_sir6553	CAGATCAAACCTCCAAACCAGG	ATGTCTAGTTTGGAGGTTTGGT	tag89400
novel_sir6554	ACTCGTGCTGAAAGTTTTGGT	AGCACGACTTTCAAAACCATG	tag24436
novel_sir6555	ACTGTAGTCGCAGGTTAAAAATGT	ACATCAGCGTCCAATTTTTACACC	tag103484
novel_sir6556	CAAGTTCGGCCGACATTCAAATGT	TCAAGCCGGCTGTAAGTTTACAAC	tag223432
novel_sir6557	GACACAGAACCTCAGGCACAATGT	GTGTCTTGGAGTCCGTGTTACACC	tag44742
novel_sir6558	GTGTCTTGGAGTCCGTGTTACAGC	GACACAGAACCTCAGGCACAATGT	tag44742
novel_sir6559	TATGGGTTCAGTACTAGACAATGT	ACCCAAGTCATGATCTGTTACATA	tag123617
novel_sir6560	CCATACACCGTGCACCAATGT	TATGTGGCACGTGGTTACACG	tag149883
novel_sir6561	ACAATTTGTCCAGCTTACAGT	TGTGTTAAACAGGTCGAATGT	tag18147

novel_sir6562	CTAAGCCCACGACACAATTACAAG	CCGATTCGGGTGCTGTGTTAATGT	tag115991
novel_sir6563	TGCACTGCATACGCACCGTCATGT	GTGACGTATGCGTGGCAGTACAGT	tag258338
novel_sir6564	TACTAAACTGTTACACCACGATGT	GATTTGACAATGTGGTGCTACAGT	tag292411
novel_sir6565	ACAGAACCTCAGGCACGATGT	TCTTGGAGTCCGTGCTACAGC	tag114372
novel_sir6566	GCAGAACCTCAGGCACGATGT	TCTTGGAGTCCGTGCTACAGC	tag274816
novel_sir6567	CGCAGAACCTCAGGCACGATGT	GTCTTGGAGTCCGTGCTACAGC	tag7751
novel_sir6568	TACAGAACCTCAGGCACGATGT	GTCTTGGAGTCCGTGCTACAGC	tag51831
novel_sir6569	CACAGAACCTCAGGCACGATGT	GTCTTGGAGTCCGTGCTACAGC	tag162172
novel_sir6570	AACACAGAACCTCAGGCACGATGT	GTGTCTTGGAGTCCGTGCTACAAC	tag27300
novel_sir6571	GACACAGAACCTCAGGCACGATGT	GTGTCTTGGAGTCCGTGCTACAAC	tag156657
novel_sir6572	CACACAGAACCTCAGGCACGATGT	GTGTCTTGGAGTCCGTGCTACAAC	tag219497
novel_sir6573	AACACAGAACCTCAGGCACGATGT	GTGTCTTGGAGTCCGTGCTACAGT	tag27300
novel_sir6574	GACACAGAACCTCAGGCACGATGT	GTGTCTTGGAGTCCGTGCTACAGT	tag156657
novel_sir6575	CACACAGAACCTCAGGCACGATGT	GTGTCTTGGAGTCCGTGCTACAGT	tag219497
novel_sir6576	AACACAGAACCTCAGGCACGATGT	GTGTCTTGGAGTCCGTGCTACAGC	tag27300
novel_sir6577	GACACAGAACCTCAGGCACGATGT	GTGTCTTGGAGTCCGTGCTACAGC	tag156657
novel_sir6578	CACACAGAACCTCAGGCACGATGT	GTGTCTTGGAGTCCGTGCTACAGC	tag219497
novel_sir6579	AACACAGAACCTCAGGCACGATGT	GTGTCTTGGAGTCCGTGCTACATC	tag27300
novel_sir6580	GACACAGAACCTCAGGCACGATGT	GTGTCTTGGAGTCCGTGCTACATC	tag156657
novel_sir6581	CACACAGAACCTCAGGCACGATGT	GTGTCTTGGAGTCCGTGCTACATC	tag219497
novel_sir6582	CGTCTTGGAGTCCGTGCTACAGC	ACGCAGAACCTCAGGCACGATGT	tag108768
novel_sir6583	GCGTCTTGGAGTCCGTGCTACAGC	GACGCAGAACCTCAGGCACGATGT	tag287097
novel_sir6584	AACGCAGAACCTCAGGCACGATGT	GCGTCTTGGAGTCCGTGCTACATC	tag231534
novel_sir6585	GACGCAGAACCTCAGGCACGATGT	GCGTCTTGGAGTCCGTGCTACATC	tag287097
novel_sir6586	AACGCAGAACCTCAGGCACGATGT	GCGTCTTGGAGTCCGTGCTACAAC	tag231534
novel_sir6587	GACGCAGAACCTCAGGCACGATGT	GCGTCTTGGAGTCCGTGCTACAAC	tag287097
novel_sir6588	GACGTAGAACCTCAGGCACGATGT	GCATCTTGGAGTCCGTGCTACAGC	tag113615
novel_sir6589	GACACAGAATCTCAGGCACGATGT	GTGTCTTAGAGTCCGTGCTACAGC	tag194323
novel_sir6590	GTGTCTTAAAGTCCGTGCTACAGC	GACACAGAATTTCAGGCACGATGT	tag273952
novel_sir6591	CCTGGATCTACTGTATGGCGATGT	ACCTAGATGACATACCGCTACAAG	tag211467
novel_sir6592	GCGTCTTGGAGTCCGAGCTACAGC	GACGCAGAACCTCAGGCTCGATGT	tag25504
novel_sir6593	CTCCACGGCACTTGCTGATGT	GGTGCCGTGAACGACTACAAC	tag178506
novel_sir6594	AGAGAAAAGCCACTTATACACC	GTTCTCTTTTCGGTGAATATGT	tag84591
novel_sir6595	TCGCCGGGCTTGGACATATGT	CGGCCCGAACCTGTATACATC	tag119482

novel_sir6596	GAGTCGCCGGGCTTGGACATATGT	CAGCGGCCCGAACCTGTATACATC	tag118425
novel_sir6597	GGCTTTGGACCGGGATGATACATT	CACCGAAACCTGGCCCTACTATGT	tag147280
novel_sir6598	CACCGAAACCTGGCCCTACTATGT	GGCTTTGGACCGGGATGATACAAT	tag147280
novel_sir6599	GCGTCTTAGAGTCCATAATACAGC	AACGCAGAATCTCAGGTATTATGT	tag15736
novel_sir6600	GGTCTAAGTCTAAGGAAATACAAA	CACCAGATTCAGATTCCTTTATGT	tag7144
novel_sir6601	GGTCTAAGTCTAAGGAAATACAAA	AACCAGATTCAGATTCCTTTATGT	tag289352
novel_sir6602	GGTGTGAAAAAACTCGGTAACTGT	ACACTTTTTTGAGCCATTGACACG	tag37070
novel_sir6603	AGGCCAGTTTGCAGACTACACTGT	CGGTCAAACGTCTGATGTGACACC	tag195189
novel_sir6604	AGGGCGTAGACCGAACGGACTGT	CCGCATCTGGCTTGCCTGACATA	tag119531
novel_sir6605	CAGGGCGTAGACCGAACGGACTGT	CCCGCATCTGGCTTGCCTGACATA	tag161566
novel_sir6606	ACGGACGGTCAAACGTTGGACACG	TCTGCCTGCCAGTTTGCAACCTGT	tag162183
novel_sir6607	TTGCCGCTACCGTAAAAACCCTGT	CGGCGATGGCATTTTTGGGACAAA	tag286489
novel_sir6608	CCACTCACTCCCTACACGGACAGC	CGGGTGAGTGAGGGATGTGCCTGT	tag87477
novel_sir6609	CCGGCGAGAGGCTGGTCCTGT	CCGCTCTCCGACCAGGACATG	tag13994
novel_sir6610	TGCCACTATCACTTCGGTTCCTGT	GGTGATAGTGAAGCCAAGGACAGA	tag200741
novel_sir6611	GAGGCCCTAGTGCGAAGCTGT	CCGGGATCACGCTTCGACAGA	tag88833
novel_sir6612	TGGTCATTGTCCATCGCTAGCTGT	CAGTAACAGGTAGCGATCGACAGG	tag188607
novel_sir6613	CGCGGCGGCGGCTGCGGCTGT	GCCGCCGCCGACGCCGACAGG	tag220592
novel_sir6614	GATTACATACAGCGGGACGACAGG	CGCTAATGTATGTCGCCCTGCTGT	tag231777
novel_sir6615	AGAAACACAGACATAGACAGG	CCTCTTTGTGTCTGTATCTGT	tag80348
novel_sir6616	TGTTGCTTGAATTGTATTATCTGT	AACGAACTTAACATAATAGACAGT	tag187678
novel_sir6617	GATGCGGCATGTGGAGACATG	TTCTACGCCGTACACCTCTGT	tag262141
novel_sir6618	ATTCTACGCCGTACACCTCTGT	AGATGCGGCATGTGGAGACATG	tag191160
novel_sir6619	CGCTCCAGCTCCTGCCTCTGT	GAGGTCGAGGACGGAGACACC	tag135683
novel_sir6620	CGAGCTACTGTTGCGAGACATT	GCGCTCGATGACAACGCTCTGT	tag244709
novel_sir6621	CAAACATAAGACTCTAAAGACATG	TGGTTTGTATTCTGAGATTTCTGT	tag89954
novel_sir6622	TACGCCGAAGAATTCACCAAGTGT	GCGGCTTCTTAAGTGGTTCACATG	tag83490
novel_sir6623	GATCGGTCGGCCAAAATTCACAAT	AACTAGCCAGCCGGTTTTAAGTGT	tag127703
novel_sir6624	TGCCACTGACTTTGTCACACA	ATACGGTGACTGAAACAGTGT	tag106233
novel_sir6625	CCGAAGGTGAAGACCGGGCAGTGT	CTTCCACTTCTGGCCCGTCACAGG	tag222487
novel_sir6626	AAGCTCATGGCAAGTCACACA	CCTTCGAGTACCGTTCAGTGT	tag265906
novel_sir6627	GCGGCAACTGAAAAAGAGTGT	CCGTTGACTTTTTCTCACATG	tag237214
novel_sir6628	TTATCTAAACCAAATGAGTGT	TAGATTTGGTTTACTCACACT	tag34973
novel_sir6629	TGGAAGTGATGAGTAAGTTAGTGT	CTTCACTACTCATTCAATCACAAG	tag276305

novel_sir6630	AGGAAGCGGCGGCTGCACAGG	CCTCCTTCGCCGCCGACGTGT	tag71461
novel_sir6631	GAGGAAGCGGCGGCTGCACAGG	GCCTCCTTCGCCGCCGACGTGT	tag83067
novel_sir6632	CCGTTAGGCCCTGTTTGGCACAGC	CTGGCAATCCGGGACAAACCGTGT	tag28992
novel_sir6633	TCTCCTAGATCTGACAAACCGTGT	AGGATCTAGACTGTTTGGCACAAC	tag217719
novel_sir6634	AATCATATCGATAGGAAGGATACCGT	AGTATAGCTATCCTTCCTATGGCACA	tag74290
novel_sir6635	CATCATATCGATAGGAAGGATACCGT	AGTATAGCTATCCTTCCTATGGCACA	tag121652
novel_sir6636	TCAGTCATAATCCGGCACACG	CAAGTCAGTATTAGGCCGTGT	tag257088
novel_sir6637	CCTTGTTCTTGTATCGCGTGT	AACAAGAACATAGCGCACACA	tag291883
novel_sir6638	GTGGCAACTGAAAAATCGTGT	CCGTTGACTTTTTAGCACATG	tag212560
novel_sir6639	AAGTGCTTGCCTCGTTCGTGT	CACGAACGGAGCAAGCACATC	tag35492
novel_sir6640	GTCGCCTACCACTGAAAGCACAAC	TGCAGCGGATGGTGACTTTCGTGT	tag240891
novel_sir6641	TGGGTCCGGTTGGTAAGGTGT	CCAGGCCAACCATTCCACATT	tag171954
novel_sir6642	CCGATGTGTCATAGGATCCACATG	TGGGCTACACAGTATCCTAGGTGT	tag205400
novel_sir6643	CGGTATGAAAACGGTGTACGGTGT	CATACTTTTGCCACATGCCACAAC	tag214106
novel_sir6644	CTGCCAGCCGCCGGCCACACC	GGGACGGTCGGCGGCCGGTGT	tag219823
novel_sir6645	CTCCGTTGAATCCGCCACATC	AGGAGGCAACTTAGGCGGTGT	tag29816
novel_sir6646	CAAGATGAGAAAATAACAGGGTGT	TCTACTCTTTTATTGTCCCACATG	tag183757
novel_sir6647	CTTCTGATCCCGCTGCTGGTGT	AGACTAGGGCGACGACCACAGG	tag248998
novel_sir6648	CAGGATCCGCATCTCCTTGGTGT	CCTAGGCGTAGAGGAACCACACC	tag101618
novel_sir6649	ACAGGATCCGCATCTCCTTGGTGT	TCCTAGGCGTAGAGGAACCACACC	tag175400
novel_sir6650	AAAGGATCCGCATCTCCTTGGTGT	TCCTAGGCGTAGAGGAACCACACC	tag175737
novel_sir6651	TACCTATCGAACAACCACAGT	CTATGGATAGCTTGTTGGTGT	tag22319
novel_sir6652	GTGTCCGGGAGCGCACATGTGT	CAGGCCCTCGCGTGTACACACC	tag266230
novel_sir6653	AAATCCCTGGTCCTACTGTGT	TAGGGACCAGGATGACACAGC	tag83333
novel_sir6654	ATCTTCAAGCATGGACACAGC	AGTAGAAGTTCGTACCTGTGT	tag54337
novel_sir6655	GCAGCGCGGAATCTAAGACACATT	GACGTCGCGCCTTAGATTCTGTGT	tag181018
novel_sir6656	GACATCGTGCCTGAGGTTCTGTGT	GTAGCACGGACTCCAAGACACAAT	tag262457
novel_sir6657	GACATCGTGCCTGAGGTTCTGTGT	GTAGCACGGACTCCAAGACACAGT	tag262457
novel_sir6658	GACATCGTGCCTGAGGTTCTGTGT	GTAGCACGGACTCCAAGACACACA	tag262457
novel_sir6659	GCAGCACGGACTCCAAGACACACT	GACGTCGTGCCTGAGGTTCTGTGT	tag103282
novel_sir6660	GACGTCGTGCCTGAGGTTCTGTGT	GCAGCACGGACTCCAAGACACAGT	tag103282
novel_sir6661	GACGTCGTGCCTGAGGTTCTGTGT	GCAGCACGGACTCCAAGACACAAT	tag103282
novel_sir6662	AGCTCATGGCAAGTCACACAGA	CTTCGAGTACCGTTCAGTGTGT	tag193409
novel_sir6663	CTTGTTCTTGTATCGCGTGTGT	ACAAGAACATAGCGCACACAGT	tag46541

novel_sir6664	TCCTTGTTCTTGTATCGCGTGTGT	GAACAAGAACATAGCGCACACAGT	tag156207
novel_sir6665	GTGTTCTGTGCTAAGGTGTGT	CAAGACACGATTCCACACATG	tag71245
novel_sir6666	TCAGTAGTTGGTACACACATA	CTAGTCATCAACCATGTGTGT	tag171991
novel_sir6667	CGTCGTGCTTAGAACCTTGTGT	AGCACGAATCTTGGAACACATC	tag269186
novel_sir6668	CTTTTAAGTCACCAAGAACACATA	CTGAAAATTCAGTGGTTCTTGTGT	tag29607
novel_sir6669	TAGTAAAAATCAGGACCAAATTGT	CATTTTTAGTCCTGGTTTAACACG	tag277370
novel_sir6670	CCTTGTCCTCATCGCGATTGT	AACAGGAGTAGCGCTAACAGC	tag167480
novel_sir6671	TGAGATCAAGGGTTGAACAGC	GCACTCTAGTTCCCAACTTGT	tag151600
novel_sir6672	TGACGAGTAGAAGTGAACATT	CTACTGCTCATCTTCACTTGT	tag24456
novel_sir6673	ACTACTGCTCATCTTCACTTGT	ATGACGAGTAGAAGTGAACATT	tag266768
novel_sir6674	AACTTTTACCGTAGTTTCACTTGT	GAAAATGGCATCAAAGTGAACAGG	tag23204
novel_sir6675	TGTTTTTTCGCTCGACCTTGT	AAAAAAGCGAGCTGGAACATC	tag236255
novel_sir6676	GGTACCTCGACAGGACCTTGT	ATGGAGCTGTCCTGGAACACA	tag58970
novel_sir6677	TGGGAACCAGGCGTGCCGAACATA	CAACCCTTGGTCCGCACGGCTTGT	tag108834
novel_sir6678	AGAGGCATCATGTAGAACAGG	GATCTCCGTAGTACATCTTGT	tag245798
novel_sir6679	ACATCTTGTCCTTGTTCTTGT	TAGAACAGGAACAAGAACATA	tag41891
novel_sir6680	CAATCTCCATCATTGATCAACACA	CTGTTAGAGGTAGTAACTAGTTGT	tag217060
novel_sir6681	CGTGCCAATTACAGAGCGTTGT	ACGGTTAATGTCTCGCAACAGT	tag153595
novel_sir6682	CACGGCACTTGCTGATGTTGT	GCCGTGAACGACTACAACAAC	tag195904
novel_sir6683	CCACGGCACTTGCTGATGTTGT	TGCCGTGAACGACTACAACAAC	tag168574
novel_sir6684	CCGTCCATGGCCGATAACAACATA	CAGGCAGGTACCGGCTATTGTTGT	tag264247
novel_sir6685	AACTTTTACCGTAGTTTCATTTGT	GAAAATGGCATCAAAGTAAACAGG	tag201675
novel_sir6686	GGCCTTAGTCAAACTTGAAACAGT	TACCGGAATCAGTTTGAACTTTGT	tag105919
novel_sir6687	GTACAGGAGCAGGATCCACTTTGT	TGTCCTCGTCCTAGGTGAAACAGC	tag55728
novel_sir6688	TAGTGTCCGCTGGCTCTTTGT	CACAGGCGACCGAGAAACAGC	tag245615
novel_sir6689	ATCATGAATGTCAGAAACAGG	CGTAGTACTTACAGTCTTTGT	tag128353
novel_sir6690	TATTCTGCTTACCAGTTTGT	AAGACGAATGGTCAAACATG	tag129442
novel_sir6691	TTATTCTGCTTACCAGTTTGT	TAAGACGAATGGTCAAACATG	tag191498
novel_sir6692	CTTATTCTGCTTACCAGTTTGT	ATAAGACGAATGGTCAAACATG	tag113627
novel_sir6693	TTTATTCTGCTTACCAGTTTGT	ATAAGACGAATGGTCAAACATG	tag189002
novel_sir6694	TGGGAAGGCTGTAGGCCAGTTTGT	CCTTCCGACATCCGGTCAAACATC	tag108806
novel_sir6695	TAAGACGAACGGTCAAACATG	TTATTCTGCTTGCCAGTTTGT	tag241040
novel_sir6696	ATAAGACGAACGGTCAAACATG	TTTATTCTGCTTGCCAGTTTGT	tag292038
novel_sir6697	AAAAAGTCAACGGCGTCAAACATT	GATTTTTCAGTTGCCGCAGTTTGT	tag65128

novel_sir6698	TAAGACGAATGATCAAACATG	TTATTCTGCTTACTAGTTTGT	tag124781
novel_sir6699	TACGAGGTACGTACACAGGTTTGT	GCTCCATGCATGTGTCCAAACATT	tag39802
novel_sir6700	TATGAGATTTAATCACAGGTTTGT	ACTCTAAATTAGTGTCCAAACATT	tag205460
novel_sir6701	TATGAGATTTAATCACAGGTTTGT	ACTCTAAATTAGTGTCCAAACATC	tag205460
novel_sir6702	ACTTTAAATTAGCGTCCAAACATT	TATGAAATTTAATCGCAGGTTTGT	tag287563
novel_sir6703	TATGAGATTTAATCGCAGGTTTGT	ACTCTAAATTAGCGTCCAAACATC	tag39330
novel_sir6704	TATGAGGTTTAATCGCAGGTTTGT	ACTCCAAATTAGCGTCCAAACATT	tag289636
novel_sir6705	AACTTATTCTGCTCACCGGTTTGT	GAATAAGACGAGTGGCCAAACAAT	tag264391
novel_sir6706	AACTTATTCTGCTTGCCGGTTTGT	GAATAAGACGAACGGCCAAACATG	tag118391
novel_sir6707	TCACCAGTTTGCAATGTTTGT	TGGTCAAACGTTACAAACAAA	tag255595
novel_sir6708	TGGGCCACTGTAAGCTTTTGT	CCGGTGACATTCGAAAACAAT	tag18165
novel_sir6709	GTGGGCCACTGTAAGCTTTTGT	CCCGGTGACATTCGAAAACAAT	tag145316
novel_sir6710	ACTGCGGCAACTGAAAAAATT	ACGCCGTTGACTTTTTTAAAC	tag37193
novel_sir6711	GATAGCACGGCACAACTTTTAAAA	GGCTATCGTGCCGTGTTGAAAATT	tag85180
novel_sir6712	ACGAACGGTCAAACATGTTTAAAA	TTTGCTTGCCAGTTTGTACAAATT	tag178013
novel_sir6713	GTCTAACTCGGCTCAAATT	GATTGAGCCGAGTTTAAAG	tag144623
novel_sir6714	GTCTAACTCGGCTCAAATT	GATTGAGCCGAGTTTAAAC	tag144623
novel_sir6715	GTCTAACTCGGCTCAAATT	GATTGAGCCGAGTTTAAAA	tag144623
novel_sir6716	GTCTAACTCGGCTCAAATT	GATTGAGCCGAGTTTAATG	tag144623
novel_sir6717	GTCTAACTCGGCTCAAATT	GATTGAGCCGAGTTTAAAT	tag144623
novel_sir6718	AGTCTAACTCGGCTCAAATT	AGATTGAGCCGAGTTTAAAT	tag110993
novel_sir6719	AGTCTAACTCGGCTCAAATT	AGATTGAGCCGAGTTTAAAC	tag110993
novel_sir6720	AGTCTAACTCGGCTCAAATT	AGATTGAGCCGAGTTTAAAA	tag110993
novel_sir6721	AAGTCTAACTCGGCTCAAATT	CAGATTGAGCCGAGTTTAATT	tag234525
novel_sir6722	AAGTCTAACTCGGCTCAAATT	CAGATTGAGCCGAGTTTAAAA	tag234525
novel_sir6723	AAGTCTAACTCGGCTCAAATT	CAGATTGAGCCGAGTTTAATA	tag234525
novel_sir6724	AAAGTCTAACTCGGCTCAAATT	TCAGATTGAGCCGAGTTTAAAT	tag110245
novel_sir6725	AAAGTCTAACTCGGCTCAAATT	TCAGATTGAGCCGAGTTTAAAA	tag110245
novel_sir6726	AAAGTCTAACTCGGCTCAAATT	TCAGATTGAGCCGAGTTTAATA	tag110245
novel_sir6727	AAAGTCTAACTCGGCTCAAATT	TCAGATTGAGCCGAGTTTAATT	tag110245
novel_sir6728	TTTTTCTAACTCGGCTCAAATT	AAAGATTGAGCCGAGTTTAAAT	tag273265
novel_sir6729	TTTTTCTAACTCGGCTCAAATT	AAAGATTGAGCCGAGTTTAACT	tag273265
novel_sir6730	TTTTTCTAACTCGGCTCAAATT	AAAGATTGAGCCGAGTTTAAAA	tag273265
novel_sir6731	TTTTTCTAACTCGGCTCAAATT	AAAGATTGAGCCGAGTTTAAAC	tag273265

novel_sir6732	AAAGATTGAGCCGAGTTTAATG	TTTTTCTAACTCGGCTCAAATT	tag273265
novel_sir6733	AAAGATTGAGCCGAGTTTAATT	TTTTTCTAACTCGGCTCAAATT	tag273265
novel_sir6734	AAAGATTGAGCCGAGTTTAATA	TTTTTCTAACTCGGCTCAAATT	tag273265
novel_sir6735	AAAGATTGAGCCGAGTTTAATC	TTTTTCTAACTCGGCTCAAATT	tag273265
novel_sir6736	TGCCCCGTCGCGTATTTAAGT	TTACGGGGCAGCGCATAAATT	tag257909
novel_sir6737	ATGCCCCGTCGCGTATTTAAGT	GTTACGGGGCAGCGCATAAATT	tag29268
novel_sir6738	AATGCCCCGTCGCGTATTTAAGT	TGTTACGGGGCAGCGCATAAATT	tag130002
novel_sir6739	CAATGCCCCGTCGCGTATTTAAGT	ATGTTACGGGGCAGCGCATAAATT	tag35400
novel_sir6740	TCTGCTTGCCAGTTTGTATAAATT	ACGAACGGTCAAACATATTTAAAA	tag290376
novel_sir6741	CACCCGATCTCCTAGACCTAAATT	GGGCTAGAGGATCTGGATTTAAAG	tag78460
novel_sir6742	GGTTGCTGAATCCTCTCCTAAATT	AACGACTTAGGAGAGGATTTAAAA	tag226769
novel_sir6743	TTACCATCGAGTGCTACCAATT	TGGTAGCTCACGATGGTTAATC	tag210631
novel_sir6744	AGTAGCATCCGGAACCCAATT	ATCGTAGGCCTTGGGTTAAGG	tag206489
novel_sir6745	GGGTCTAGTGGCAAATCGTTAAGC	CACCCAGATCACCGTTTAGCAATT	tag77428
novel_sir6746	GGGTCTAGTGGCAAATCGTTAAGC	AACCCAGATCACCGTTTAGCAATT	tag289898
novel_sir6747	AATTCCTGGCTCGGTACTTCAATT	AAGGACCGAGCCATGAAGTTAAGC	tag269935
novel_sir6748	GGTAGTCTTGAGGCTTCAATT	ATCAGAACTCCGAAGTTAAGC	tag61504
novel_sir6749	GGGTAGTCTTGAGGCTTCAATT	CATCAGAACTCCGAAGTTAAGC	tag181721
novel_sir6750	ATTTTCACTAGCGGCCTCTTAACA	GATAAAAGTGATCGCCGGAGAATT	tag157282
novel_sir6751	TGTGGCTCGCATGCTTAAAT	GTACACCGAGCGTACGAATT	tag58955
novel_sir6752	ATGTGGCTCGCATGCTTAAAT	TGTACACCGAGCGTACGAATT	tag110636
novel_sir6753	AACGAGGTCCGGTTCGTGCGAATT	GCTCCAGGCCAAGCACGCTTAACC	tag286115
novel_sir6754	ACTCTCGCCCAAGCACGCTTAACT	GATGAGAGCGGGTTCGTGCGAATT	tag280607
novel_sir6755	ATCCGATATGATAATGACTTAAAA	TGTAGGCTATACTATTACTGAATT	tag267202
novel_sir6756	TGTAGGCTACACTATCCCTGAATT	ATCCGATGTGATAGGGACTTAAAA	tag89723
novel_sir6757	TTTAGGCTACACTATCCCTGAATT	ATCCGATGTGATAGGGACTTAAAA	tag197527
novel_sir6758	GGTATCAGAGCGGACGACTTAAGG	TACCATAGTCTCGCCTGCTGAATT	tag98254
novel_sir6759	TTAGGAAACGTCTGCTGAATT	TCCTTTGCAGACGACTTAAAT	tag133655
novel_sir6760	AGATTGTAGGTGCCGGTATTAAAT	TATCTAACATCCACGGCCATAATT	tag71822
novel_sir6761	CAGTTGCCGCAGTAGATAATT	CAACGGCGTCATCTATTAAAA	tag92838
novel_sir6762	TTTCAGTTGCCGCAGTAGATAATT	AGTCAACGGCGTCATCTATTAAAA	tag101290
novel_sir6763	GTTTCCCACAACCAGCTAATT	AAGGGTGTTGGTCGATTAAGA	tag46153
novel_sir6764	GATGGTTGGCCTTGATTTCTAATT	ACCAACCGGAACTAAAGATTAAGA	tag135427
novel_sir6765	CATCATATCCACAGCCAATTAATT	AGTATAGGTGTCGGTTAATTAAAA	tag237997

novel_sir6766	AGTGGCAGGCGGACACTTTTAATT	ACCGTCCGCCTGTGAAAATTAATT	tag91366
novel_sir6767	TATGCGCTGCCCCGTAACATT	ACGCGACGGGGCATTGTAAGT	tag262301
novel_sir6768	TTATGCGCTGCCCCGTAACATT	TACGCGACGGGGCATTGTAAGT	tag42181
novel_sir6769	TACTAGAAATCAGGGCCAACCATT	GATCTTTAGTCCCGGTTGGTAACA	tag97135
novel_sir6770	AGCTAGAAATCAGGGCCAACCATT	GATCTTTAGTCCCGGTTGGTAACA	tag147572
novel_sir6771	TGCCTAGGCGAGTCATGTGGCATT	GGATCCGCTCAGTACACCGTAATT	tag182154
novel_sir6772	TACTGGCACGGCGCTATCATT	GACCGTGCCGCGATAGTAATT	tag171093
novel_sir6773	GTTCACCAGTTTGCAATGCTCATT	AGTGGTCAAACGTTACGAGTAAAA	tag200877
novel_sir6774	CTTTGTCCTTGTATGGTCATT	AACAGGAACATACCAGTAATG	tag174427
novel_sir6775	AGTGGTCAAACGATATAAGTAAAA	GTTCACCAGTTTGCTATATTCATT	tag192564
novel_sir6776	GCTCACCAGTTTGCAACGTTCATT	AGTGGTCAAACGTTGCAAGTAAAA	tag158207
novel_sir6777	GCTCACCGGTTTGTCATGTTCATT	AGTGGCCAAACAGTACAAGTAAAA	tag197574
novel_sir6778	ACTTGTGGCTAACTATTTCTAAGC	TGTGAACACCGATTGATAAAGATT	tag42844
novel_sir6779	TGTGAACACCGGTTGATAAAGATT	ACTTGTGGCCAACTATTTCTAAGC	tag127376
novel_sir6780	TGTGAACACCGGTTGATAAAGATT	ACTTGTGGCCAACTATTTCTAAGT	tag127376
novel_sir6781	TAATACTGACTTGCGGAGATT	TATGACTGAACGCCTCTAAGT	tag25232
novel_sir6782	CGGTCGACGTCATGCCTGAGATT	CAGCTGCAGTACGGACTCTAAGA	tag117140
novel_sir6783	AATTCATCTATAGCCAATCTAATA	GGTTAAGTAGATATCGGTTAGATT	tag57525
novel_sir6784	AATTCATCTATAGCCAATCTAATA	GATTAAGTAGATATCGGTTAGATT	tag218146
novel_sir6785	TTTGCCAGTTTGTACACGATT	ACGGTCAAACATGTGCTAAAA	tag39823
novel_sir6786	CTTGCCAGTTTGTACACGATT	ACGGTCAAACATGTGCTAAAA	tag235369
novel_sir6787	CTTGCCAGTTTGTATACGATT	ACGGTCAAACATATGCTAAAA	tag163070
novel_sir6788	TTTGCCAGTTTGTATACGATT	ACGGTCAAACATATGCTAAAA	tag288013
novel_sir6789	GTTTGCCAGTTTGTATACGATT	AACGGTCAAACATATGCTAAAA	tag22721
novel_sir6790	GCTTGCCAGTTTGTATACGATT	AACGGTCAAACATATGCTAAAA	tag108023
novel_sir6791	TCAAACTTCCGTGGCCTAAAC	GGAGTTTGAAGGCACCGGATT	tag35064
novel_sir6792	ATCTTACGAACGCGTCGCCTAACA	TGTAGAATGCTTGCGCAGCGGATT	tag57150
novel_sir6793	CGTGCGTTGGTTTGTGATCGGATT	ACGCAACCAAACACTAGCCTAAAA	tag203953
novel_sir6794	AGACGAATCTTTTAAGCCTAATT	GCTCTGCTTAGAAAATTCGGATT	tag89644
novel_sir6795	TTGACTTGTTCAACCTCCTGGATT	CTGAACAAGTTGGAGGACCTAAAC	tag87476
novel_sir6796	TCACCTCCGTGCGCACCTAACG	GGAGTGGAGGCACGCGTGGATT	tag95819
novel_sir6797	GGTCTGACTCGGTGTTTGATT	AGACTGAGCCACAAACTAAGC	tag194738
novel_sir6798	TCTAGGATCGTCTTTATAAAA	CGAGATCCTAGCAGAAATATT	tag245144
novel_sir6799	TGGCTCAGTCTGGTTATAAAC	ACACCGAGTCAGACCAATATT	tag96789

novel_sir6800	ATTACGATCTTTCAGAATATT	ATGCTAGAAAGTCTTATAACC	tag82674
novel_sir6801	TTTACGATCTTTCAGAATATT	ATGCTAGAAAGTCTTATAACC	tag119649
novel_sir6802	GTTACGATCTTTCAGAATATT	ATGCTAGAAAGTCTTATAACC	tag143211
novel_sir6803	AGCCTTGAGTTTCCGTGTATAAAT	CGTCGGAACTCAAAGGCACATATT	tag183818
novel_sir6804	CGTCAAATGTCCGCCTTAGATATT	AGTTTACAGGCGGAATCTATAATT	tag247951
novel_sir6805	GTGCCGTTTCGAAAAGCTATT	CGGCAAAGCTTTTCGATAACC	tag33627
novel_sir6806	AGTTTAGGCCCGATTGAGATAACA	CATCAAATCCGGGCTAACTCTATT	tag220780
novel_sir6807	AGTTTAGGCCCGATTGAGATAACG	CATCAAATCCGGGCTAACTCTATT	tag220780
novel_sir6808	GATTCGTAAAGATCAAGTATT	AAGCATTTCTAGTTCATAACA	tag176756
novel_sir6809	TAGAGGCGTTCAGTCATAATC	GAATCTCCGCAAGTCAGTATT	tag24089
novel_sir6810	ATATTTGAAGTTTACCGGTATT	TAAACTTCAAATGGCCATAACT	tag253028
novel_sir6811	AATCACTTTTTATACCGCGGTATT	AGTGAAAAATATGGCGCCATAAAT	tag226806
novel_sir6812	AGTGAAAAATATGGCGCCATAAAA	AATCACTTTTTATACCGCGGTATT	tag226806
novel_sir6813	AACTACTTTTTATACCGCGGTATT	GATGAAAAATATGGCGCCATAAAT	tag27316
novel_sir6814	TGTAACGAGCCGAACGGGTATT	ATTGCTCGGCTTGCCCATAACC	tag250821
novel_sir6815	GCTAGGCATCGGGGTAGGTTTATT	ATCCGTAGCCCCATCCAAATAACT	tag148528
novel_sir6816	CTTCTACCGCTAGGTCAAAAACTT	AGATGGCGATCCAGTTTTTGAATG	tag74436
novel_sir6817	GCAGATTCGGTGGCCGTTTGAAGT	TACGTCTAAGCCACCGGCAAACTT	tag215239
novel_sir6818	TACGTCTAAGCCGCCGGCAAACTT	GCAGATTCGGCGGCCGTTTGAAGT	tag126429
novel_sir6819	ACGCACCGTTTAGCAGTTTGAAAA	TTTGCGTGGCAAATCGTCAAACTT	tag82484
novel_sir6820	ACCAGTAGCGCGAACCAACTT	GTCATCGCGCTTGGTTGAAAA	tag114396
novel_sir6821	TCCTACGGGAGGATCGGTGAACTT	GATGCCCTCCTAGCCACTTGAAGC	tag250108
novel_sir6822	GTTCAAAACCAAGCAAGGTAACTT	AGTTTTGGTTCGTTCCATTGAATC	tag164405
novel_sir6823	TATGGCTTTGAGTTTGTGAATG	CCATACCGAAACTCAAACACTT	tag50624
novel_sir6824	TCACCTGCATGAGCTACCACTT	TGGACGTACTCGATGGTGAAGT	tag288768
novel_sir6825	TGTCCTCGTCCTAGGTGAAAC	GTACAGGAGCAGGATCCACTT	tag21382
novel_sir6826	TGCTTGCTCCGTTCGTGAAAA	ACACGAACGAGGCAAGCACTT	tag264038
novel_sir6827	GGTCCTTAGCTTGCGCCCTCACTT	AGGAATCGAACGCGGGAGTGAACG	tag278842
novel_sir6828	ATAGCTGGCTTTAAGTGAAAA	ATTATCGACCGAAATTCACTT	tag292779
novel_sir6829	AAACTCGGCTCAATCTGAAAA	AATTTGAGCCGAGTTAGACTT	tag24646
novel_sir6830	TAAACTCGGCTCAATCTGAAAA	AAATTTGAGCCGAGTTAGACTT	tag134129
novel_sir6831	TAAACTCGGCTCAATCTGAAAA	TAATTTGAGCCGAGTTAGACTT	tag145235
novel_sir6832	TTAAACTCGGCTCAATCTGAAAA	AAAATTTGAGCCGAGTTAGACTT	tag51763
novel_sir6833	TTAAACTCGGCTCAATCTGAAAA	ATAATTTGAGCCGAGTTAGACTT	tag99005

novel_sir6834	TTAAACTCGGCTCAATCTGAAAA	TTAATTTGAGCCGAGTTAGACTT	tag282294
novel_sir6835	ATTAAACTCGGCTCAATCTGAAAA	GTTAATTTGAGCCGAGTTAGACTT	tag225829
novel_sir6836	TTTCTGACCCGGCCGTTTAGACTT	AGACTGGGCCGGCAAATCTGAAGA	tag148562
novel_sir6837	GGGTTGCCCGCCGCCCGACTT	CAACGGGCGGCGGGCTGAATC	tag262935
novel_sir6838	AGGGTTGCCCGCCGCCCGACTT	CCAACGGGCGGCGGGCTGAATC	tag75133
novel_sir6839	GGGTTTCACCGTTTTATAGGACTT	CAAAGTGGCAAAATATCCTGAAGG	tag147578
novel_sir6840	CACACGGCCTAATACTGACTT	GTGCCGGATTATGACTGAACG	tag21814
novel_sir6841	TGATAATGAAGACACTGAACT	ACACTATTACTTCTGTGACTT	tag252855
novel_sir6842	CCACCACTCCACGACCTACTT	TGGTGAGGTGCTGGATGAAGG	tag288750
novel_sir6843	TAGATTGGCTATAGATGAATT	TAATCTAACCGATATCTACTT	tag133318
novel_sir6844	ACTAATACTGGCGAAGTACTT	ATTATGACCGCTTCATGAACT	tag84587
novel_sir6845	TTAATAATGTGCGCATGAAAT	TCAATTATTACACGCGTACTT	tag288679
novel_sir6846	GGAGTTTTGTACCAAAGCAACCTT	TCAAAACATGGTTTCGTTGGAATG	tag225702
novel_sir6847	AATTATTTGAGCATTGGAAGT	CTTTAATAAACTCGTAACCTT	tag46551
novel_sir6848	TCCCACTCTCGCGCGGACCTT	GGTGAGAGCGCGCCTGGAAGC	tag179763
novel_sir6849	TTTTAGAGAATATAATACCTT	AATCTCTTATATTATGGAACG	tag260651
novel_sir6850	CGTTATAGCCAGACGTACCTT	AATATCGGTCTGCATGGAAGG	tag255266
novel_sir6851	TACTATTATCCACCGACTTACCTT	GATAATAGGTGGCTGAATGGAATA	tag194127
novel_sir6852	ATAGCGCTAACAATGGGAACA	CATATCGCGATTGTTACCCTT	tag177455
novel_sir6853	GGTGTCCGTCAGCTGACCCCTT	ACAGGCAGTCGACTGGGGAACG	tag177112
novel_sir6854	CCGTCGGCAGCCCGGGAAGG	GCGGCAGCCGTCGGGCCCTT	tag257990
novel_sir6855	CGCTTTGGTGTCGGTTCCCTT	GAAACCACAGCCAAGGGAACG	tag97739
novel_sir6856	CTTGCAAACTGACCTACAGCCTT	ACGTTTGACTGGATGTCGGAAGA	tag100709
novel_sir6857	ACGTTTGGTCGGATGTCGGAAGG	CCTGCAAACCAGCCTACAGCCTT	tag41431
novel_sir6858	GATGTTTGACCGGATGTCGGAAGG	GCCTACAAACTGGCCTACAGCCTT	tag252523
novel_sir6859	ACGTTTGACCGGATGTCGGAAGA	CCTGCAAACTGGCCTACAGCCTT	tag51516
novel_sir6860	ACGTTTGACCGGATGTCGGAAGA	ATTGCAAACTGGCCTACAGCCTT	tag120438
novel_sir6861	ACGTTTGACCGGATGTCGGAAGA	TCTGCAAACTGGCCTACAGCCTT	tag284455
novel_sir6862	TTTGCAAACTGGCCTACAGCCTT	ACGTTTGACCGGATGTCGGAAGG	tag4661
novel_sir6863	CCTGCAAACTGGCCTACAGCCTT	ACGTTTGACCGGATGTCGGAAGG	tag51516
novel_sir6864	ATTGCAAACTGGCCTACAGCCTT	ACGTTTGACCGGATGTCGGAAGG	tag120438
novel_sir6865	CTTGCAAACTGGCCTACAGCCTT	ACGTTTGACCGGATGTCGGAAGG	tag130701
novel_sir6866	TCTGCAAACTGGCCTACAGCCTT	ACGTTTGACCGGATGTCGGAAGG	tag284455
novel_sir6867	GTCTGCAAACTGGCCTACAGCCTT	GACGTTTGACCGGATGTCGGAAGA	tag2193

novel_sir6868	GCCTGCAAACTGGCCTACAGCCTT	GACGTTTGACCGGATGTCGGAAGA	tag38733
novel_sir6869	TCCTGCAAACTGGCCTACAGCCTT	GACGTTTGACCGGATGTCGGAAGA	tag183656
novel_sir6870	GTCTGCAAACTGGCCTACAGCCTT	GACGTTTGACCGGATGTCGGAAAG	tag2193
novel_sir6871	GCCTGCAAACTGGCCTACAGCCTT	GACGTTTGACCGGATGTCGGAAAG	tag38733
novel_sir6872	TCCTGCAAACTGGCCTACAGCCTT	GACGTTTGACCGGATGTCGGAAAG	tag183656
novel_sir6873	GACGTTTGACCGGATGTCGGAAGG	GTCTGCAAACTGGCCTACAGCCTT	tag2193
novel_sir6874	GACGTTTGACCGGATGTCGGAAGG	GCCTGCAAACTGGCCTACAGCCTT	tag38733
novel_sir6875	GCTTGCAAACTGGCCTACAGCCTT	AACGTTTGACCGGATGTCGGAAGG	tag247663
novel_sir6876	AACGTTTGACCGGATGTCGGAAGA	GCTTGCAAACTGGCCTACAGCCTT	tag247663
novel_sir6877	GCCTGCAAACTGGCTTACAGCCTT	GACGTTTGACCGAATGTCGGAAGT	tag219699
novel_sir6878	TGGTGAAATACCCTGATCGGCCTT	CACTTTATGGGACTAGCCGGAATA	tag239756
novel_sir6879	CGCCGAATCCTCGCAAACATCCTT	GGCTTAGGAGCGTTTGTAGGAAGT	tag205788
novel_sir6880	GGCTTGAGAGCGTTTGTAGGAAGT	CGCCGAACTCTCGCAAACATCCTT	tag128311
novel_sir6881	GGTGACTCGATGACTCCTT	ACTGAGCTACTGAGGAACA	tag195589
novel_sir6882	CACCGCCTAAGTTGCCTCCTT	GGCGGATTCAACGGAGGAAGC	tag107723
novel_sir6883	ACACCGCCTAAGTTGCCTCCTT	TGGCGGATTCAACGGAGGAAGC	tag251055
novel_sir6884	ACATTGTGAAACGGAGGAAGT	AATGTAACACTTTGCCTCCTT	tag241628
novel_sir6885	GTACTTACAGTCTTTGTCCTT	TGAATGTCAGAAACAGGAACA	tag112983
novel_sir6886	GGTCTAAGTCTAAGGAAAT	CACCAGATTCAGATTCCTT	tag2212
novel_sir6887	GGTCTAAGTCTAAGGAAAT	AACCAGATTCAGATTCCTT	tag59923
novel_sir6888	GGTCTAAGTCTAAGGAAAC	CACCAGATTCAGATTCCTT	tag2212
novel_sir6889	GGTCTAAGTCTAAGGAAAC	AACCAGATTCAGATTCCTT	tag59923
novel_sir6890	CACCAGATTCAGATTCCTT	GGTCTAAGTCTAAGGAAAA	tag2212
novel_sir6891	TACCAGATTCAGATTCCTT	GGTCTAAGTCTAAGGAAAA	tag6546
novel_sir6892	AACCAGATTCAGATTCCTT	GGTCTAAGTCTAAGGAAAA	tag59923
novel_sir6893	CCTAAACGGCCGGGTCAGAAGCTT	ATTTGCCGGCCCAGTCTTCGAAGA	tag177631
novel_sir6894	TGGCTCCCGCGCCGATAAGCTT	CGAGGGCGCGGCTATTCGAATA	tag58487
novel_sir6895	TGGCGCCCGTGCCGATAAGCTT	CGCGGGCACGGCTATTCGAATA	tag230163
novel_sir6896	GATGGACCGCGCGGTTGACAGCTT	ACCTGGCGCGCCAACTGTCGAAAC	tag188087
novel_sir6897	CTCCCTTTATGTCATCGTCGAACT	CGGAGGGAAATACAGTAGCAGCTT	tag202402
novel_sir6898	CGGCGCCGGCCGGCGGAGCTT	CGCGGCCGGCCGCCTCGAAGC	tag183541
novel_sir6899	GCGGCGCCGGCCGGCGGAGCTT	CCGCGGCCGGCCGCCTCGAAGC	tag2062
novel_sir6900	TATTTTTATCCGAATCGAACT	CCATAAAAATAGGCTTAGCTT	tag231794
novel_sir6901	ACTTTTCGGTCACCGCGCTT	AAAAGCCAGTGGCGCGAAGC	tag25796

novel_sir6902	AACTTTTCGGTCACCGCGCTT	GAAAAGCCAGTGGCGCGAAGC	tag38617
novel_sir6903	CAACTTTTCGGTCACCGCGCTT	TGAAAAGCCAGTGGCGCGAAGC	tag78513
novel_sir6904	ACCAACTTTTCGGTCACCGCGCTT	GTTGAAAAGCCAGTGGCGCGAAGC	tag161983
novel_sir6905	TTCCGCAAACACTTCCGAACC	CGAAGGCGTTTGTGAAGGCTT	tag126450
novel_sir6906	GGGCTTGATAGCGCCAGCAGGCTT	CGAACTATCGCGGTCGTCCGAATT	tag289740
novel_sir6907	CAATTAATTAATGGCCGAAGA	GGGTTAATTAATTACCGGCTT	tag25839
novel_sir6908	CATCTTTCGTCGGTCGGCCGAATT	TGGTAGAAAGCAGCCAGCCGGCTT	tag238195
novel_sir6909	ACGTGTTGAGACCTAGGCCGAAAC	GGTGCACAACTCTGGATCCGGCTT	tag21756
novel_sir6910	GGGGAGGAGAGTCGGTCGGCTT	CCTCCTCTCAGCCAGCCGAAAG	tag280435
novel_sir6911	ACCTCTGGGCGCAAGATGGCTT	GAGACCCGCGTTCTACCGAACT	tag178129
novel_sir6912	TACGGTCGTACAGCCCAGTGGCTT	GCCAGCATGTCGGGTCACCGAACT	tag179497
novel_sir6913	CGATGCCGCCGGCCGCTGCTT	TACGGCGGCCGGCGACGAAGG	tag221500
novel_sir6914	GCTATGAGTGGTTGCGCTGCTT	ATACTCACCAACGCGACGAACG	tag38552
novel_sir6915	AATTGGCATACAGGAAGACGAAAG	TATTAACCGTATGTCCTTCTGCTT	tag90893
novel_sir6916	TGCTAGTCGGCGCCACGAACT	GTACGATCAGCCGCGGTGCTT	tag94777
novel_sir6917	ATGCTAGTCGGCGCCACGAACT	GGTACGATCAGCCGCGGTGCTT	tag266448
novel_sir6918	GCACGATCGGCCGCGGTGCTT	TGCTAGCCGGCGCCACGAACT	tag9277
novel_sir6919	GTACATCTAGGACTAAAATCTT	TGTAGATCCTGATTTTAGAAAA	tag48799
novel_sir6920	ACGTCCACTCGAGTTATTAGAATC	CCTGCAGGTGAGCTCAATAATCTT	tag161610
novel_sir6921	CCTGCAGGTGAGCTCGATAATCTT	ACGTCCACTCGAGCTATTAGAATC	tag215267
novel_sir6922	CTTGAGGAGAGTGACGATCTT	ACTCCTCTCACTGCTAGAAAC	tag110577
novel_sir6923	AGAACTCCTCTCACTGCTAGAAAC	GGTCTTGAGGAGAGTGACGATCTT	tag266744
novel_sir6924	AATGTGGGCAATGCTAGAAAG	ACTTACACCCGTTACGATCTT	tag90601
novel_sir6925	AATGTGGGCAATGCTAGAATA	ACTTACACCCGTTACGATCTT	tag90601
novel_sir6926	AATGTGGACAATGCTAGAAAG	ACTTACACCTGTTACGATCTT	tag110212
novel_sir6927	ACTTATACCTGTTACGATCTT	AATATGGACAATGCTAGAAAG	tag55617
novel_sir6928	AATGTGGGAAATGCTAGAATG	ACTTACACCCTTTACGATCTT	tag232161
novel_sir6929	ACTTACACTCTTTACGATCTT	AATGTGAGAAATGCTAGAATG	tag84910
novel_sir6930	AATGTGGAAAATGCTAGAATG	ACTTACACCTTTTACGATCTT	tag77459
novel_sir6931	TGGTGCAAGCGAACCCCCGATCTT	CACGTTCGCTTGGGGGCTAGAAAT	tag68076
novel_sir6932	GGTGCAAGCGACACCTCCGATCTT	ACGTTCGCTGTGGAGGCTAGAAAT	tag15923
novel_sir6933	GTACCGCAGGCAGTGTCCGATCTT	TGGCGTCCGTCACAGGCTAGAAGT	tag102963
novel_sir6934	TGGGGAACGAGACCTAGAACA	GGACCCCTTGCTCTGGATCTT	tag163672
novel_sir6935	AGGTATGCTACCTTTTTGTATCTT	CATACGATGGAAAAACATAGAACC	tag64955

novel_sir6936	GGACTAAGGCTCAGGCATACTCTT	TGATTCCGAGTCCGTATGAGAAAG	tag12090
novel_sir6937	GGCGACCGATGGGTTTGGAGAAAA	GCCCGCTGGCTACCCAAACCTCTT	tag263906
novel_sir6938	GACGTTGGCCCAACCGGGAGAAAG	CTCTGCAACCGGGTTGGCCCTCTT	tag95069
novel_sir6939	TGACTGCATCGGCTGCCTCTT	TGACGTAGCCGACGGAGAAGG	tag78961
novel_sir6940	CTATTCGATCCGGTATGCTCTT	TAAGCTAGGCCATACGAGAATT	tag272591
novel_sir6941	TAGACGCGAGAGGACGAGAAAT	CTATCTGCGCTCTCCTGCTCTT	tag264869
novel_sir6942	GACTAGGGTGCACCCGGTGCTCTT	GATCCCACGTGGGCCACGAGAAAA	tag189039
novel_sir6943	CGTAGCTATCCGGACGCCTCTCTT	ATCGATAGGCCTGCGGAGAGAACA	tag131091
novel_sir6944	CGGCCGGCCGAAGGGTCTCTT	CGGCCGGCTTCCCAGAGAAAG	tag120600
novel_sir6945	CGGCCGGCTTGCCCAGAGAAAA	CGGCCGGCCGAACGGGTCTCTT	tag171452
novel_sir6946	CGGCCGGCTTGCCCAGAGAAAG	CGGCCGGCCGAACGGGTCTCTT	tag171452
novel_sir6947	CGGCCGGCTTGCCCAGAGAAGG	CGGCCGGCCGAACGGGTCTCTT	tag171452
novel_sir6948	AAGCCCGTGACCGCCTTAAGTCTT	CGGGCACTGGCGGAATTCAGAATT	tag82509
novel_sir6949	GACTTGCGGAGATTCAGTCTT	GAACGCCTCTAAGTCAGAATC	tag157392
novel_sir6950	TTCGTGGCCTAGGGTAGTCTT	GCACCGGATCCCATCAGAACT	tag119620
novel_sir6951	TTCGTACTGGAAATCAGAATC	ATAAGCATGACCTTTAGTCTT	tag202611
novel_sir6952	ACGCCTAGGACACCAGAATA	GATGCGGATCCTGTGGTCTT	tag194782
novel_sir6953	AGATGCGGATCCTGTGGTCTT	TACGCCTAGGACACCAGAATA	tag75422
novel_sir6954	TACGCCTAGGACACCAGAAAA	AGATGCGGATCCTGTGGTCTT	tag75422
novel_sir6955	GCTCCGTTTTCCGTGTGAAGAAGA	GGCGAGGCAAAAGGCACACTTCTT	tag73482
novel_sir6956	GAATCTATCGATCTCTGACTTCTT	TAGATAGCTAGAGACTGAAGAAAC	tag53147
novel_sir6957	TGTGGCAAATGGAAATAAAGAACT	TCACACCGTTTACCTTTATTTCTT	tag7596
novel_sir6958	TTCATCGAAACGAAAAGAATG	GTAAGTAGCTTTGCTTTTCTT	tag162778
novel_sir6959	CGCGCCACTGGCTTTTCAACC	AAGCGCGGTGACCGAAAAGTT	tag179068
novel_sir6960	TCGCGCCACTGGCTTTTCAACC	GAAGCGCGGTGACCGAAAAGTT	tag130726
novel_sir6961	TTCGCGCCACTGGCTTTTCAACC	CGAAGCGCGGTGACCGAAAAGTT	tag254618
novel_sir6962	TGACGTAGGTGAATTAAAGTT	TGCATCCACTTAATTTCAATC	tag125559
novel_sir6963	TCCGTTTCAGGTGGTTCAAGT	CTAGGCAAAGTCCACCAAGTT	tag48692
novel_sir6964	AGTGGCTGGCGCCGTCGTTCAACA	TCTCACCGACCGCGGCAGCAAGTT	tag270831
novel_sir6965	CTGGCACTATTCATCTTCAAGC	GGGACCGTGATAAGTAGAAGTT	tag23379
novel_sir6966	CGAGCCCTACACCGCCTAAGTT	TCGGGATGTGGCGGATTCAACG	tag59529
novel_sir6967	GCACGGCGCTATCATTAAGTT	TGCCGCGATAGTAATTCAACC	tag79812
novel_sir6968	GGCACGGCGCTATCATTAAGTT	GTGCCGCGATAGTAATTCAACC	tag221499
novel_sir6969	TCTGTATCTGTCCGTTAAGTT	ACATAGACAGGCAATTCAAGG	tag137719

novel_sir6970	AAAGTCAACGGTGTCAAAC	CTTTTCAGTTGCCACAGTT	tag12601
novel_sir6971	ACTATAAAGTCAACGGTGTCAAAT	CATGATATTTCAGTTGCCACAGTT	tag282552
novel_sir6972	AAAAAGTCAACGGTGTCAAAC	TTTTTTTCAGTTGCCACAGTT	tag24212
novel_sir6973	AAAAAGTCAACGGTGTCAAAC	TATTTTTCAGTTGCCACAGTT	tag120100
novel_sir6974	AAAAAGTCAACGGTGTCAAAC	ATTTTTTCAGTTGCCACAGTT	tag264409
novel_sir6975	GCTAAAAAGTCAACGGTGTCAAAC	TACGATTTTTCAGTTGCCACAGTT	tag124908
novel_sir6976	GCTAAAAAGTCAACGGTGTCAAAC	CACGATTTTTCAGTTGCCACAGTT	tag292860
novel_sir6977	GCTAAAAAGTCAACGGTGTCAAAT	TACGATTTTTCAGTTGCCACAGTT	tag124908
novel_sir6978	GCTAAAAAGTCAACGGTGTCAAAT	CACGATTTTTCAGTTGCCACAGTT	tag292860
novel_sir6979	TATGATTTTTCAGTTGCCACAGTT	ACTAAAAAGTCAACGGTGTCAAAC	tag50458
novel_sir6980	CATGATTTTTCAGTTGCCACAGTT	ACTAAAAAGTCAACGGTGTCAAAC	tag155769
novel_sir6981	TATAAAAGAATTATAGGCACAGTT	ATTTTCTTAATATCCGTGTCAACT	tag226127
novel_sir6982	ACTCTATGACTACCGTATACAGTT	AGATACTGATGGCATATGTCAAAC	tag205534
novel_sir6983	GCAATCGACCGTCAGCCAGTT	TTAGCTGGCAGTCGGTCAAGC	tag266115
novel_sir6984	TTTGGGGAAGGCTGTAGGCCAGTT	ACCCCTTCCGACATCCGGTCAAAC	tag85989
novel_sir6985	TTTGGGGAAGGTTGTAGGCCAGTT	ACCCCTTCCAACATCCGGTCAAAC	tag70599
novel_sir6986	AAAAAGTCAACGGCGTCAAAC	ATTTTTTCAGTTGCCGCAGTT	tag38350
novel_sir6987	TACGATTTTTCAGTTGCCGCAGTT	GCTAAAAAGTCAACGGCGTCAAAC	tag95194
novel_sir6988	AACGATTTTTCAGTTGCCGCAGTT	GCTAAAAAGTCAACGGCGTCAAAC	tag197053
novel_sir6989	CACGATTTTTCAGTTGCCGCAGTT	GCTAAAAAGTCAACGGCGTCAAAC	tag219090
novel_sir6990	CACTATTTTTCAGTTGCCGCAGTT	GATAAAAAGTCAACGGCGTCAAAC	tag91034
novel_sir6991	TACTATTTTTCAGTTGCCGCAGTT	GATAAAAAGTCAACGGCGTCAAAC	tag199396
novel_sir6992	TCTTCATCCAACGCGTCAACC	GGAGAAGTAGGTTGCGCAGTT	tag90187
novel_sir6993	GTACCGGCGCGAGCGGCAGTT	TGGCCGCGCTCGCCGTCAAGG	tag2241
novel_sir6994	TGTACCGGCGCGAGCGGCAGTT	ATGGCCGCGCTCGCCGTCAAGG	tag104523
novel_sir6995	ACATGTGCTAAAAAGTCAACG	TTTGTACACGATTTTTCAGTT	tag273978
novel_sir6996	AATTTGTACACGATTTTTCAGTT	AAACATGTGCTAAAAAGTCAACG	tag112571
novel_sir6997	AGTTTGTACACGATTTTTCAGTT	AAACATGTGCTAAAAAGTCAACG	tag121818
novel_sir6998	TTTTTGTACACGATTTTTCAGTT	AAACATGTGCTAAAAAGTCAACG	tag160664
novel_sir6999	TGTTTGTACACGATTTTTCAGTT	AAACATGTGCTAAAAAGTCAACG	tag186366
novel_sir7000	CAAACATGTGCTAAAAAGTCAACG	TAGTTTGTACACGATTTTTCAGTT	tag223059
novel_sir7001	TAGTTTGTGCACGATTTTTCAGTT	CAAACACGTGCTAAAAAGTCAACG	tag21723
novel_sir7002	CAGTTTGTGCACGATTTTTCAGTT	CAAACACGTGCTAAAAAGTCAACG	tag130748
novel_sir7003	AGTTTGTATACGATTTTTCAGTT	AAACATATGCTAAAAAGTCAACG	tag72934

novel_sir7004	TTTTTGTATACGATTTTTCAGTT	AAACATATGCTAAAAAGTCAACG	tag223719
novel_sir7005	AAACACGTACTAAAAAGTCAACG	AGTTTGTGCATGATTTTTCAGTT	tag129856
novel_sir7006	TAGTTTGTGCATGATTTTTCAGTT	CAAACACGTACTAAAAAGTCAACG	tag101920
novel_sir7007	ACATGTGAGAAAAAGTCAACG	ATTGTACACTCTTTTTCAGTT	tag268969
novel_sir7008	AAACATGTGAGAAAAAGTCAACG	TTTTTGTACACTCTTTTTCAGTT	tag183876
novel_sir7009	CAGTTTGTACACTCTTTTTCAGTT	CAAACATGTGAGAAAAAGTCAACG	tag112753
novel_sir7010	AAGTTTGTACACTCTTTTTCAGTT	CAAACATGTGAGAAAAAGTCAACG	tag203792
novel_sir7011	TAGTTTGTACACTCTTTTTCAGTT	CAAACATGTGAGAAAAAGTCAACG	tag217748
novel_sir7012	AAACATGTTTAAAAAAGTCAACG	TTTTTGTACAAATTTTTTCAGTT	tag34558
novel_sir7013	AAACATGTTTAAAAAAGTCAACG	AGTTTGTACAAATTTTTTCAGTT	tag226987
novel_sir7014	AAGTTTGTACAAATTTTTTCAGTT	CAAACATGTTTAAAAAAGTCAACG	tag38480
novel_sir7015	CAGTTTGTACAAATTTTTTCAGTT	CAAACATGTTTAAAAAAGTCAACG	tag71158
novel_sir7016	TAGTTTGTACAAATTTTTTCAGTT	CAAACATGTTTAAAAAAGTCAACG	tag255332
novel_sir7017	AGTTTGTATAAATTTTTTCAGTT	AAACATATTTAAAAAAGTCAACG	tag255271
novel_sir7018	AGTTTGTACAAATTTTTTTCAGTT	AAACATGTTTAAAAAAAGTCAACG	tag140477
novel_sir7019	ATTTGACTGCAGAGGATCTCAATC	TGTAAACTGACGTCTCCTAGAGTT	tag107443
novel_sir7020	GATGGGGTGTACAGTATCTGAGTT	ACCCCACATGTCATAGACTCAATC	tag18087
novel_sir7021	CGCTTATGGGCCGACCTATCAATG	TGGCGAATACCCGGCTGGATAGTT	tag245139
novel_sir7022	GTAGTTCCGCTCGATGTAGTT	TCAAGGCGAGCTACATCAACA	tag115426
novel_sir7023	CGCAAAAAGGACGCCAGGTTAGTT	GTTTTTCCTGCGGTCCAATCAAAC	tag174024
novel_sir7024	CACAAAAAGGACGCCAGGTTAGTT	GTTTTTCCTGCGGTCCAATCAAAC	tag195655
novel_sir7025	TACAAAAAGGACGCCAGGTTAGTT	GTTTTTCCTGCGGTCCAATCAAAC	tag282251
novel_sir7026	AGTGGCAGGCGGACGCTTTTAGTT	ACCGTCCGCCTGCGAAAATCAATT	tag9553
novel_sir7027	GTGGACTGCCAGCGAAAATCAATT	GACACCTGACGGTCGCTTTTAGTT	tag93989
novel_sir7028	TCCCGAGCTCCGTTTGCAAAT	GTAGGGCTCGAGGCAAACGTT	tag23811
novel_sir7029	GGAGCTACACGCGAAACGTT	TCGATGTGCGCTTTGCAAGT	tag17127
novel_sir7030	GGAGCTACACGCGAAACGTT	TCGATGTGCGCTTTGCAAGG	tag17127
novel_sir7031	TTCTGCTCACCAGTTTGCAACGTT	GACGAGTGGTCAAACGTTGCAAGC	tag18811
novel_sir7032	GAATCCAGGTGCTTGAACGTT	TAGGTCCACGAACTTGCAAAG	tag117864
novel_sir7033	CTGGGCTCACCGATTGCAACC	ACGACCCGAGTGGCTAACGTT	tag254405
novel_sir7034	CCGGTGATCGGGTTTAACGTT	CCACTAGCCCAAATTGCAAAG	tag226640
novel_sir7035	CCACTAGCCCAAATTGCAAGG	CCGGTGATCGGGTTTAACGTT	tag226640
novel_sir7036	TTTGCAACTTGTACTTATCACGTT	ACGTTGAACATGAATAGTGCAAAA	tag51324
novel_sir7037	GCACAAAGAGCCGGAGACGTT	TGTTTCTCGGCCTCTGCAAAA	tag163943

novel_sir7038	CAGATTTGTAGCCGGCTGCAACA	ATGTCTAAACATCGGCCGACGTT	tag65035
novel_sir7039	TTAAGCTGATCCAATGACGTT	TTCGACTAGGTTACTGCAAAC	tag2244
novel_sir7040	CTAAGAGGTCTGCGGATACGTT	TTCTCCAGACGCCTATGCAAGA	tag154624
novel_sir7041	CAACGATCCCCATACTATGCAAAT	TGGTTGCTAGGGGTATGATACGTT	tag102231
novel_sir7042	CCTCCTGCTGCTCCTACCGTT	AGGACGACGAGGATGGCAACG	tag236929
novel_sir7043	ACGACGACGAGGATGGCAACG	CCTGCTGCTGCTCCTACCGTT	tag64091
novel_sir7044	TCAGGCGGGACCGGTACCGTT	TCCGCCCTGGCCATGGCAAAG	tag45357
novel_sir7045	GTATATACACTTACACCCGTT	TATATGTGAATGTGGGCAATG	tag253467
novel_sir7046	GTAGATATACTTACACCCGTT	TCTATATGAATGTGGGCAATG	tag107375
novel_sir7047	TTAGATATACTTACACCCGTT	TCTATATGAATGTGGGCAATG	tag256845
novel_sir7048	GTAGATATACTTACACCCGTT	TCTATATGAATGTGGGCAAGG	tag107375
novel_sir7049	TTAGATATACTTACACCCGTT	TCTATATGAATGTGGGCAAGG	tag256845
novel_sir7050	GTATATATACTTACACCCGTT	TATATATGAATGTGGGCAATG	tag89151
novel_sir7051	GTATATATACTTACACCCGTT	TATATATGAATGTGGGCAAGG	tag89151
novel_sir7052	GTATATATACTTACATCCGTT	TATATATGAATGTAGGCAATG	tag66542
novel_sir7053	TTATATATACTTACATCCGTT	TATATATGAATGTAGGCAATG	tag240361
novel_sir7054	TTCCTGAGCAGCCAGCGCAAGG	GGAAGGACTCGTCGGTCGCGTT	tag128473
novel_sir7055	TTCCTGAGCAGCCAGCGCAAGA	GGAAGGACTCGTCGGTCGCGTT	tag128473
novel_sir7056	GGAACGGTACTCGAAGGCGTT	TTGCCATGAGCTTCCGCAAAC	tag26030
novel_sir7057	TGAACGGTACTCGAAGGCGTT	TTGCCATGAGCTTCCGCAAAC	tag179730
novel_sir7058	AGAGTCAGACCGTGGGGCGTT	TCAGTCTGGCACCCCGCAATG	tag58919
novel_sir7059	GGAGTCAGACCGTGGGGCGTT	TCAGTCTGGCACCCCGCAATG	tag292465
novel_sir7060	CTCAGTCTGGCACCCCGCAATG	AAGAGTCAGACCGTGGGGCGTT	tag157623
novel_sir7061	GAATTGGTGAGTGACGCAAGA	CTCTTAACCACTCACTGCGTT	tag90537
novel_sir7062	GAATTGGTGAGTGACGCAAGG	CTCTTAACCACTCACTGCGTT	tag90537
novel_sir7063	AGAATTGGTGAGTGACGCAAGG	TCTCTTAACCACTCACTGCGTT	tag105990
novel_sir7064	AGAATTGGTGAGTGACGCAAGA	TCTCTTAACCACTCACTGCGTT	tag105990
novel_sir7065	CGTCGTGCCTGAGGTTCTGCGTT	AGCACGGACTCCAAGACGCAATG	tag264614
novel_sir7066	AGCACGGACTCCAAGACGCAAGG	CGTCGTGCCTGAGGTTCTGCGTT	tag264614
novel_sir7067	CGAACTGAGTTGACGCCGTGCGTT	TTGACTCAACTGCGGCACGCAACC	tag121579
novel_sir7068	CCGAGTGGCAGCAGCGTGCGTT	CTCACCGTCGTCGCACGCAAGG	tag221850
novel_sir7069	GCTCACCGTCGTCGCACGCAAGG	TCCGAGTGGCAGCAGCGTGCGTT	tag151929
novel_sir7070	GCGCGCTGCAAGCAAGTAGCAATG	TGCGCGCGACGTTCGTTCATCGTT	tag142175
novel_sir7071	TCAGTCTTAGGTTCGATCGTT	TCAGAATCCAAGCTAGCAAGC	tag289201

novel_sir7072	TCAGTCTTAGGTTCGATCGTT	TCAGAATCCAAGCTAGCAAGG	tag289201
novel_sir7073	TCAGTCTTAGGTTCGATCGTT	TCAGAATCCAAGCTAGCAACC	tag289201
novel_sir7074	TTATGGGCGAGTTGAGCAATG	CCAATACCCGCTCAACTCGTT	tag143207
novel_sir7075	GCGGCGGCGGTGCGGCTCGTT	CCGCCGCCACGCCGAGCAAGC	tag70718
novel_sir7076	TTATGGGCAAGCCGAGCAATG	CCAATACCCGTTCGGCTCGTT	tag202205
novel_sir7077	AGTGGATCGTGGCAGCAAGG	AGTCACCTAGCACCGTCGTT	tag90495
novel_sir7078	CCACTACCAGGAGCCGTCGTT	TGATGGTCCTCGGCAGCAAGG	tag206800
novel_sir7079	GCTGCATCTGTGGGTGGTCGTT	ACGTAGACACCCACCAGCAAGG	tag205560
novel_sir7080	GGATCACCGTTTAGCAATTCGTT	TAGTGGCAAATCGTTAAGCAACA	tag47936
novel_sir7081	AGATCACCGTTTAGCAATTCGTT	TAGTGGCAAATCGTTAAGCAACA	tag90499
novel_sir7082	TTCTAAGCATGGCTAAGCAATT	CAAAGATTCGTACCGATTCGTT	tag186184
novel_sir7083	TACAAAGATTCGTACCGATTCGTT	GTTTCTAAGCATGGCTAAGCAATT	tag155265
novel_sir7084	AGTGGTCAAACGTTACAAGCAAAA	GCTCACCAGTTTGCAATGTTCGTT	tag206677
novel_sir7085	TCGAAGGATCAAAAAGCAACG	GTAGCTTCCTAGTTTTTCGTT	tag262194
novel_sir7086	GGTTAAGTCTCGCCAAAACAGGTT	AATTCAGAGCGGTTTTGTCCAATG	tag144584
novel_sir7087	AATCCAGAGCGGTTTTGTCCAACG	GGTTAGGTCTCGCCAAAACAGGTT	tag155159
novel_sir7088	GGTCGACATCGTGCCTGAGGTT	AGCTGTAGCACGGACTCCAAGG	tag97898
novel_sir7089	CGGTCGACATCGTGCCTGAGGTT	CAGCTGTAGCACGGACTCCAAGG	tag264331
novel_sir7090	CGGTCGACATCGTGCCTGAGGTT	CAGCTGTAGCACGGACTCCAAGA	tag264331
novel_sir7091	CAGCTATAGCACGGACTCCAAGA	CGGTCGATATCGTGCCTGAGGTT	tag35063
novel_sir7092	AGCTGCAGCACGGACTCCAAGG	GGTCGACGTCGTGCCTGAGGTT	tag56581
novel_sir7093	CGGTCGACGTCGTGCCTGAGGTT	CAGCTGCAGCACGGACTCCAAGA	tag11556
novel_sir7094	CAGCTGCAGCACGGACTCCAAGG	CGGTCGACGTCGTGCCTGAGGTT	tag11556
novel_sir7095	AGTAGATATGACGGAACCTAGGTT	ATCTATACTGCCTTGGATCCAAAT	tag12470
novel_sir7096	CGGAGATTCAGTCTTAGGTT	CTCTAAGTCAGAATCCAAGC	tag6266
novel_sir7097	GCGGAGATTCAGTCTTAGGTT	CCTCTAAGTCAGAATCCAAGC	tag43705
novel_sir7098	GCGGAGATTCAGTCTTAGGTT	CCTCTAAGTCAGAATCCAAGG	tag43705
novel_sir7099	TGCGGAGATTCAGTCTTAGGTT	GCCTCTAAGTCAGAATCCAAGC	tag282812
novel_sir7100	TGCGGAGATTCAGTCTTAGGTT	GCCTCTAAGTCAGAATCCAAGG	tag282812
novel_sir7101	TTGCGGAGATTCAGTCTTAGGTT	CGCCTCTAAGTCAGAATCCAAGC	tag260350
novel_sir7102	TTGCGGAGATTCAGTCTTAGGTT	CGCCTCTAAGTCAGAATCCAAGG	tag260350
novel_sir7103	AGTGATTTCACACCGTGTTAGGTT	ACTAAAGTGTGGCACAATCCAATT	tag213754
novel_sir7104	GTCCGCCGCGTTAGAAATCCAATC	ACCAGGCGGCGCAATCTTTAGGTT	tag175264
novel_sir7105	TTGACGCTTTCGTAAACGGTT	CTGCGAAAGCATTTGCCAAGG	tag66422

novel_sir7106	AATATATGTACTTAAGTGCCAAGG	TTTTATATACATGAATTCACGGTT	tag49866
novel_sir7107	AATATATGTACTTAAGTGCCAAGT	TTTTATATACATGAATTCACGGTT	tag49866
novel_sir7108	CTCCTCGCGCGTGGCCAAGG	CGGAGGAGCGCGCACCGGTT	tag109203
novel_sir7109	CCTCCTCGCGCGTGGCCAAGG	ACGGAGGAGCGCGCACCGGTT	tag29865
novel_sir7110	AATATTTATAATTAGTGGCCAAAC	AATTATAAATATTAATCACCGGTT	tag50418
novel_sir7111	AACTTAGCGCTAACATACCGGTT	GAATCGCGATTGTATGGCCAACC	tag266279
novel_sir7112	TACTTAGCGCTAACATACCGGTT	GAATCGCGATTGTATGGCCAACC	tag284658
novel_sir7113	AAGACGAATTGCCGGGCGGTT	CTGCTTAACGGCCCGCCAACC	tag168196
novel_sir7114	AAGCTGTCTGGCGGATGCGGTT	CGACAGACCGCCTACGCCAAGG	tag14933
novel_sir7115	CGACAGACCGCCTACGCCAAGC	AAGCTGTCTGGCGGATGCGGTT	tag14933
novel_sir7116	AATATCCGATGTGGCACGCCAAAA	TATTATAGGCTACACCGTGCGGTT	tag162758
novel_sir7117	ATTCTTAAATCTCAGATCGGTT	AGAATTTAGAGTCTAGCCAAGG	tag271767
novel_sir7118	CATACTCTCATTTGCTAGCCAAAG	CAGTATGAGAGTAAACGATCGGTT	tag196996
novel_sir7119	TACTCCAGGATTGGTATCGGTT	GAGGTCCTAACCATAGCCAAGG	tag199579
novel_sir7120	AGCAATGCCACTATCACTTCGGTT	GTTACGGTGATAGTGAAGCCAAGG	tag240915
novel_sir7121	CGGAGCTTCGAGGGAAGGGTT	CTCGAAGCTCCCTTCCCAACG	tag263278
novel_sir7122	GCGGAGCTTCGAGGGAAGGGTT	CCTCGAAGCTCCCTTCCCAACG	tag113249
novel_sir7123	GACAAAGAAAGTCGAATGAGGGTT	GTTTCTTTCAGCTTACTCCCAATA	tag219407
novel_sir7124	AGTCGACTGCAGAGGATCCCAATC	CGTCAGCTGACGTCTCCTAGGGTT	tag266975
novel_sir7125	AAGCCGAGCAATGTACCCAAGT	CGTTCGGCTCGTTACATGGGTT	tag53474
novel_sir7126	AAGCCGAGCAATGTACCCAAGG	CGTTCGGCTCGTTACATGGGTT	tag53474
novel_sir7127	GGTATCTGTTCTCATGGCTGGGTT	ATAGACAAGAGTACCGACCCAACC	tag66734
novel_sir7128	TCTAAGAATCTTCGACCAATGGTT	ATTCTTAGAAGCTGGTTACCAACC	tag81490
novel_sir7129	TTGAAAAGTCAGTGTAGCATGGTT	CTTTTCAGTCACATCGTACCAATT	tag153269
novel_sir7130	TTGAAAAGATAGTGTAGCATGGTT	CTTTTCTATCACATCGTACCAATT	tag192632
novel_sir7131	AACCAAGCGCGATGACCAATT	AGTTGGTTCGCGCTACTGGTT	tag104066
novel_sir7132	AACCAAGCGCGATGACCAATT	TGTTGGTTCGCGCTACTGGTT	tag282127
novel_sir7133	ACGCGTACGCTATATAGACCAAAA	GGTGCGCATGCGATATATCTGGTT	tag172366
novel_sir7134	GGTGATTAGTGACTGGTTCTGGTT	ACTAATCACTGACCAAGACCAAAC	tag18424
novel_sir7135	ACCACAAGACCAAGACCAACC	CGTGGTGTTCTGGTTCTGGTT	tag284402
novel_sir7136	CACCACAAGACCAAGACCAACC	CCGTGGTGTTCTGGTTCTGGTT	tag237911
novel_sir7137	TATCTTTTATTTTCACCAATT	AAATAGAAAATAAAAGTGGTT	tag224823
novel_sir7138	CGAACGCCTGAACGAGTGGTT	TTGCGGACTTGCTCACCAAGG	tag192319
novel_sir7139	TTGCGGACTTGCTCACCAAGC	CGAACGCCTGAACGAGTGGTT	tag192319

novel_sir7140	TGAATCACTAGTGGCCGTGGTT	TTAGTGATCACCGGCACCAAGG	tag89168
novel_sir7141	GTGCTTTGGCGCACGGTGGTT	CGAAACCGCGTGCCACCAAGC	tag1994
novel_sir7142	CCGCTCGATGTAGTTGTGGTT	CGAGCTACATCAACACCAAGG	tag148122
novel_sir7143	AGAGCGGCACGCATAATTGGTT	TCGCCGTGCGTATTAACCAAGG	tag9593
novel_sir7144	AGTTAGAGGCTACAGTTGGTT	AATCTCCGATGTCAACCAAGG	tag160180
novel_sir7145	GGCATATAGGCTACACTGTTGGTT	GTATATCCGATGTGACAACCAAAC	tag44457
novel_sir7146	AATCTTATTCAGGGACCTTTGGTT	AGAATAAGTCCCTGGAAACCAAAC	tag37338
novel_sir7147	AAGGCCACTCTGCCACTTACAATG	CGTTCCGGTGAGACGGTGAATGTT	tag225319
novel_sir7148	TTAACTCGTTCTGACTCATGTT	TTGAGCAAGACTGAGTACAACC	tag93734
novel_sir7149	TAGATCGCGTCGAGGGCTACAAGG	ACATCTAGCGCAGCTCCCGATGTT	tag34765
novel_sir7150	TAGATCGCGTCGAGGGCTACAAGT	ACATCTAGCGCAGCTCCCGATGTT	tag34765
novel_sir7151	TAGATCGCGTCGAGGGCTACAAGA	ACATCTAGCGCAGCTCCCGATGTT	tag34765
novel_sir7152	ACATCTAGCGCAGCTCCCGATGTT	TAGATCGCGTCGAGGGCTACAAAT	tag34765
novel_sir7153	TCGGGAGCTGCACTGGATGTT	CCCTCGACGTGACCTACAACT	tag151374
novel_sir7154	GGAACCTGGCCCTACTATGTT	TTGGACCGGGATGATACAATA	tag91548
novel_sir7155	GAAACCTGGCCCTACTATGTT	TTGGACCGGGATGATACAATA	tag209596
novel_sir7156	CATTCGGACGGCAACCATACAAGT	CAGTAAGCCTGCCGTTGGTATGTT	tag253865
novel_sir7157	GTATATATACTTACACCTGTT	TATATATGAATGTGGACAATG	tag144008
novel_sir7158	AAGACTGTAAGCTGGACAAAT	CCTTCTGACATTCGACCTGTT	tag45864
novel_sir7159	CGATACCCGAGATAGCCTGTT	TATGGGCTCTATCGGACAAGT	tag92333
novel_sir7160	ATCAACTATTGAAACCGGACAAAT	GGTAGTTGATAACTTTGGCCTGTT	tag223093
novel_sir7161	CTCAACTATTTAAAACGGACAAAT	GGGAGTTGATAAATTTTGCCTGTT	tag9590
novel_sir7162	TGACCTGAGTGTACAGTATCTGTT	TGGACTCACATGTCATAGACAAAT	tag22053
novel_sir7163	GAACAACAGCGGAAGACAAAA	CTCTTGTTGTCGCCTTCTGTT	tag235759
novel_sir7164	TTCCACTTTCGGCCCGTCACAAAT	CAAAGGTGAAAGCCGGGCAGTGTT	tag291605
novel_sir7165	GAAAAAGTGTGGCAAGTCACAACT	GACTTTTTCACACCGTTCAGTGTT	tag105188
novel_sir7166	TCACTAATCGGCTCAGAACGTGTT	TGATTAGCCGAGTCTTGCACAAGA	tag254316
novel_sir7167	ACCAACCAGCCGGTTTAAGGTGTT	GTTGGTCGGCCAAATTCCACAATA	tag267831
novel_sir7168	TCCCAGAAGGCCGATCGAGGTGTT	GGTCTTCCGGCTAGCTCCACAAGG	tag47677
novel_sir7169	ATATTGGTCTGACTCGGTGTT	TAACCAGACTGAGCCACAAAC	tag80665
novel_sir7170	CAAATATTGGTCTGACTCGGTGTT	TTATAACCAGACTGAGCCACAAAC	tag149838
novel_sir7171	CCTAAGGAGTCCAACTGGTGTT	ATTCCTCAGGTTGACCACAAAC	tag107731
novel_sir7172	GTAATTGGCACGGTTGGTGTT	TTAACCGTGCCAACCACAAGG	tag12482
novel_sir7173	TTAACCGTGCCAACCACAAGC	GTAATTGGCACGGTTGGTGTT	tag12482

novel_sir7174	TGTAATTGGCACGGTTGGTGTT	ATTAACCGTGCCAACCACAAGC	tag77857
novel_sir7175	ACATTCGGCCGGGCTACTTGTT	TAAGCCGGCCCGATGAACAAGG	tag2226
novel_sir7176	AAAACCAATATTCAAGCAACAATC	TCTTTTGGTTATAAGTTCGTTGTT	tag253862
novel_sir7177	TTATTAGAATTCGACTTTGTT	TAATCTTAAGCTGAAACAAAC	tag256190
novel_sir7178	GTCTGGCCGGGACCTGTCTTTGTT	GACCGGCCCTGGACAGAAACAATC	tag36868
novel_sir7179	AGCGGTCAAACAGTTCAAACAAAA	GCTCGCCAGTTTGTCAAGTTTGTT	tag85288
novel_sir7180	GGTGGGCCACTGTAAGCTTTTGTT	ACCCGGTGACATTCGAAAACAATG	tag33469
novel_sir7181	TGACGCCGTTGATTTTTTTAAACA	AAACTGCGGCAACTAAAAAAATTT	tag278461
novel_sir7182	TGCTTGCCAGTTTGTAAAAAATTT	GAACGGTCAAACATTTTTTAAAAA	tag13825
novel_sir7183	GCTTGCCAGTTTGTAAAAATTT	AACGGTCAAACATTTTTAAAAA	tag189787
novel_sir7184	GACGTCCACCCGAGTGTTTAAAAC	ACCTGCAGGTGGGCTCACAAATTT	tag144933
novel_sir7185	GGTCAAACATGTTTAAAAA	TGCCAGTTTGTACAAATTT	tag172373
novel_sir7186	TTGCCAGTTTGTACAAATTT	CGGTCAAACATGTTTAAAAA	tag160909
novel_sir7187	CTTGCCAGTTTGTACAAATTT	ACGGTCAAACATGTTTAAAAA	tag191948
novel_sir7188	TTTGCCAGTTTGTACAAATTT	ACGGTCAAACATGTTTAAAAA	tag291580
novel_sir7189	AACGGTCAAACATGTTTAAAAA	GTTTGCCAGTTTGTACAAATTT	tag279556
novel_sir7190	ATTTGCCGGCCCAGTCTTTAAAGA	TCTAAACGGCCGGGTCAGAAATTT	tag179599
novel_sir7191	CTTGCCAGTTTGTATAAATTT	ACGGTCAAACATATTTAAAAA	tag269625
novel_sir7192	AACGGTCAAACATATTTAAAAA	GCTTGCCAGTTTGTATAAATTT	tag173678
novel_sir7193	GTTGCTGAATCCTCTCCTAAATTT	ACGACTTAGGAGAGGATTTAAAAA	tag4699
novel_sir7194	CAGCCTGACAACGTGTTAAACC	GTGTCGGACTGTTGCACAATTT	tag125859
novel_sir7195	ATACCTAATTAATCCGAATTT	TGGATTAATTAGGCTTAAAAA	tag27674
novel_sir7196	ACACACCGTTTAGCAGCTTAAAAA	TTTGTGTGGCAAATCGTCGAATTT	tag139214
novel_sir7197	TAGGAAACGTCTGCTGAATTT	CCTTTGCAGACGACTTAAATA	tag2511
novel_sir7198	TTAGGAAACGTCTGCTGAATTT	TCCTTTGCAGACGACTTAAATA	tag252115
novel_sir7199	TTCAGTTGCCGCAGTAGATAATTT	GTCAACGGCGTCATCTATTAAAAA	tag154559
novel_sir7200	ATTCAGTCTTACCCCTATTAAATG	AATAAGTCAGAATGGGGATAATTT	tag142228
novel_sir7201	GTGGTCAAACGATATAAGTAAAAA	TTCACCAGTTTGCTATATTCATTT	tag96580
novel_sir7202	GTGGTCAAACAGTACAAGTAAAAA	CACACCAGTTTGTCATGTTCATTT	tag189520
novel_sir7203	AGCTTCACAACTCCACTCTAAACC	TGTCGAAGTGTTGAGGTGAGATTT	tag128075
novel_sir7204	CGCCAACAAGCTGTCCCACGATTT	GGTTGTTCGACAGGGTGCTAAACT	tag277493
novel_sir7205	GAACGGTCAAACATATGCTAAAAA	TGCTTGCCAGTTTGTATACGATTT	tag219954
novel_sir7206	ATAACCAAACCAAATCGATTT	TTGGTTTGGTTTAGCTAAAGG	tag218159
novel_sir7207	ATAACCAAACCAAATCGATTT	TTGGTTTGGTTTAGCTAAATG	tag218159

novel_sir7208	TCAAACTTCCGTGGCCTAAACG	GGAGTTTGAAGGCACCGGATTT	tag80278
novel_sir7209	TGTTATCTCAATCGGGCCTAAACT	TTACAATAGAGTTAGCCCGGATTT	tag3147
novel_sir7210	TGTCATCCTGGTCCCTAAACT	ACACAGTAGGACCAGGGATTT	tag89181
novel_sir7211	CTAAACCCTAAACCCTAAACC	TGGATTTGGGATTTGGGATTT	tag27697
novel_sir7212	ACAAGTTGGAGGACCTAAACG	CTTGTTCAACCTCCTGGATTT	tag181004
novel_sir7213	TCTTGTTCAACCTCCTGGATTT	AACAAGTTGGAGGACCTAAACG	tag67724
novel_sir7214	ACTTGTTCAACCTCCTGGATTT	AACAAGTTGGAGGACCTAAACG	tag105260
novel_sir7215	TGACTTGTTCAACCTCCTGGATTT	TGAACAAGTTGGAGGACCTAAACG	tag67623
novel_sir7216	TGCTTACCAGTTTGTGCATGATTT	GAATGGTCAAACACGTACTAAAAA	tag224784
novel_sir7217	TAGTTAATGGCTACATCGTGATTT	CAATTACCGATGTAGCACTAAACC	tag19820
novel_sir7218	ACACTCTTACCTCTGTGATTT	TGAGAATGGAGACACTAAATC	tag141813
novel_sir7219	TGGCTCAGTCTGGTTATAAACA	ACACCGAGTCAGACCAATATTT	tag245255
novel_sir7220	AGAAGTTAGGTTCCGATATTT	TTCAATCCAAGGCTATAAATA	tag162334
novel_sir7221	GTTCACTCTCACGGCCTATAAAAT	TGCAAGTGAGAGTGCCGGATATTT	tag218986
novel_sir7222	TGCTTACCAGTTTGCAACCTATTT	GAATGGTCAAACGTTGGATAAAAA	tag173800
novel_sir7223	GTATTCCGGCTAGTCCCATAAAGT	TCCATAAGGCCGATCAGGGTATTT	tag229529
novel_sir7224	ATAATTTGCATCTGATAATTATTT	TTAAACGTAGACTATTAATAAAAC	tag104155
novel_sir7225	TGGTAGATCAACTGTACGTTATTT	CATCTAGTTGACATGCAATAAACG	tag21356
novel_sir7226	ATCAAAGTCGACTCCAAATAAAGT	TGTAGTTTCAGCTGAGGTTTATTT	tag84375
novel_sir7227	CCAGTAGCGCGAACCAACTTT	TCATCGCGCTTGGTTGAAAGG	tag145968
novel_sir7228	CCAGTAGCGCGAACCAACTTT	TCATCGCGCTTGGTTGAAAAG	tag145968
novel_sir7229	ACCAGTAGCGCGAACCAACTTT	GTCATCGCGCTTGGTTGAAAAG	tag46087
novel_sir7230	AGTTAAGCCAGGATCCATCACTTT	AATTCGGTCCTAGGTAGTGAAAGA	tag292242
novel_sir7231	TATAATCTAGATATGTATCACTTT	ATTAGATCTATACATAGTGAAATA	tag47659
novel_sir7232	TGTAATCTAGATATGTATCACTTT	ATTAGATCTATACATAGTGAAATA	tag98038
novel_sir7233	TTCGTGGATCTAAGTGAAACC	TCAAGCACCTAGATTCACTTT	tag206658
novel_sir7234	GTATGCACCAGAGCTATAGACTTT	TACGTGGTCTCGATATCTGAAAAC	tag39686
novel_sir7235	TAAACTCGGCTCAATCTGAAAAA	TAATTTGAGCCGAGTTAGACTTT	tag52782
novel_sir7236	TAAACTCGGCTCAATCTGAAAAA	AAATTTGAGCCGAGTTAGACTTT	tag205680
novel_sir7237	GCCTGATAGGCTGCACCCGACTTT	GACTATCCGACGTGGGCTGAAAGA	tag14326
novel_sir7238	ATACCGATCCTACCCAACCTTT	TGGCTAGGATGGGTTGGAAACT	tag202617
novel_sir7239	AACACTGCCCGGCTTTCACCTTT	GTGACGGGCCGAAAGTGGAAACC	tag86816
novel_sir7240	AAACACTGCCCGGCTTTCACCTTT	TGTGACGGGCCGAAAGTGGAAACC	tag275094
novel_sir7241	TCAATATGAATGTGGGAAATG	GTAGTTATACTTACACCCTTT	tag117645

novel_sir7242	ACGTTTGGTCGGATGTCGGAAAGA	CCTGCAAACCAGCCTACAGCCTTT	tag123663
novel_sir7243	CTAGTTTCAACCTGCGCCTTT	TCAAAGTTGGACGCGGAAAAC	tag26986
novel_sir7244	TGCCAGTTTGCAACCCGTGCCTTT	GGTCAAACGTTGGGCACGGAAACC	tag81941
novel_sir7245	TGCCAGTTTGCAACCCGTGCCTTT	GGTCAAACGTTGGGCACGGAAATC	tag81941
novel_sir7246	GCCAGTTTGCAACCTGTGCCTTT	GTCAAACGTTGGACACGGAAACC	tag260619
novel_sir7247	GGTCAAACGTTGGACACGGAAACC	TGCCAGTTTGCAACCTGTGCCTTT	tag271255
novel_sir7248	GGTCAAACGTTGGACACGGAAATC	TGCCAGTTTGCAACCTGTGCCTTT	tag271255
novel_sir7249	GGTCTAAGTCTAAGGAAAAA	AACCAGATTCAGATTCCTTT	tag278034
novel_sir7250	CACCAGATTCAGATTCCTTT	GGTCTAAGTCTAAGGAAATC	tag206614
novel_sir7251	AACCAGATTCAGATTCCTTT	GGTCTAAGTCTAAGGAAATC	tag278034
novel_sir7252	CACCAGATTCAGATTCCTTT	GGTCTAAGTCTAAGGAAATA	tag206614
novel_sir7253	AACCAGATTCAGATTCCTTT	GGTCTAAGTCTAAGGAAATA	tag278034
novel_sir7254	GGTTTGACGATCGGTTGTTCCTTT	AAACTGCTAGCCAACAAGGAAATC	tag201550
novel_sir7255	AGTTGCCGCAGTTTGTAAAGCTTT	AACGGCGTCAAACATTTCGAAATG	tag5488
novel_sir7256	AGTTGCCGCAGTTTGTAAAGCTTT	AACGGCGTCAAACATTTCGAAACG	tag5488
novel_sir7257	AGCTACTTCTTGCATCGCTTT	GATGAAGAACGTAGCGAAATG	tag278961
novel_sir7258	AACGTATCCGTCGACAGCGAAACG	CGTTGCATAGGCAGCTGTCGCTTT	tag155447
novel_sir7259	AGTTACCTAGTCCAAAGCGAAAAG	TTTCAATGGATCAGGTTTCGCTTT	tag86848
novel_sir7260	AGACTTAAGGCGGTCACGGGCTTT	TGAATTCCGCCAGTGCCCGAAATG	tag233332
novel_sir7261	TGAATTCCGCCAGTGCCCGAAAGG	AGACTTAAGGCGGTCACGGGCTTT	tag233332
novel_sir7262	AGCGACCATCTAGGTGAATGCTTT	GCTGGTAGATCCACTTACGAAAAT	tag200777
novel_sir7263	AAATCGGCCGACCGACGAAAGA	CCTTTAGCCGGCTGGCTGCTTT	tag210409
novel_sir7264	CACCTTTAGCCGGCTGGCTGCTTT	GGAAATCGGCCGACCGACGAAAGG	tag222417
novel_sir7265	GTCAAACTTTTCACACGGTGCTTT	GTTTGAAAAGTGTGCCACGAAAAT	tag142092
novel_sir7266	GTCAAACTTTTCGCACGGTGCTTT	GTTTGAAAAGCGTGCCACGAAAAT	tag29826
novel_sir7267	GACAAACTTTTCGCACGGTGCTTT	GTTTGAAAAGCGTGCCACGAAAAT	tag86501
novel_sir7268	GTCAAATTTTTCGCACGGTGCTTT	GTTTAAAAAGCGTGCCACGAAAAT	tag86692
novel_sir7269	CTTACACCCGTTACGATCTTT	ATGTGGGCAATGCTAGAAAGT	tag85314
novel_sir7270	CTTACACCTGTTACGATCTTT	ATGTGGACAATGCTAGAAAGT	tag71272
novel_sir7271	GTTCGCTCTCATGGCCTAGAAATT	TGCAAGCGAGAGTACCGGATCTTT	tag34292
novel_sir7272	CCTGAACTAACCAGGCGGATCTTT	ACTTGATTGGTCCGCCTAGAAAAA	tag100990
novel_sir7273	TATACAGGTCTGTGCAATATCTTT	ATGTCCAGACACGTTATAGAAACG	tag110609
novel_sir7274	TATCAGTTTGCAACCTATATCTTT	AGTCAAACGTTGGATATAGAAACA	tag231876
novel_sir7275	GTAGACCGAACGGACTGTATCTTT	TCTGGCTTGCCTGACATAGAAACC	tag173667

novel_sir7276	TTACAAGTGACGATCATTATCTTT	TGTTCACTGCTAGTAATAGAAAGC	tag206984
novel_sir7277	GACTAAGGCTCAGGCATACTCTTT	GATTCCGAGTCCGTATGAGAAAGG	tag48486
novel_sir7278	GACTAAGGCTCAGGCATACTCTTT	GATTCCGAGTCCGTATGAGAAAGT	tag48486
novel_sir7279	GGCCGGCTTGCCCAGAGAAAGA	GGCCGGCCGAACGGGTCTCTTT	tag270069
novel_sir7280	TGTCGATGTCATCGTATTCTCTTT	AGCTACAGTAGCATAAGAGAAAGG	tag43107
novel_sir7281	GATCTAACGTTGGACACAGAAATT	TGCTAGATTGCAACCTGTGTCTTT	tag190840
novel_sir7282	GGCTGGCACGGGTATTAAGAAAGT	CTCCGACCGTGCCCATAATTCTTT	tag252458
novel_sir7283	GGTTGAACATTATACAGCTTCTTT	AACTTGTAATATGTCGAAGAAATA	tag96370
novel_sir7284	AGTGGTCAAACAGTGCAAGAAATC	GTTCACCAGTTTGTCACGTTCTTT	tag193968
novel_sir7285	GGTAAATCTACTGTACGTTCTTT	ATTTAGATGACATGCAAGAAACG	tag36643
novel_sir7286	GTTCACCAGTTTGCAAGGTTCTTT	AGTGGTCAAACGTTCCAAGAAAAA	tag121671
novel_sir7287	CGGTACTCGTGCTGAAAGTTT	CATGAGCACGACTTTCAAAAC	tag98969
novel_sir7288	TTGACCGTTCGTCTTATTCAAAAA	CAAACTGGCAAGCAGAATAAGTTT	tag18268
novel_sir7289	ACTGGTAAGCAGAATAAGTTT	ACCATTCGTCTTATTCAAAAA	tag205248
novel_sir7290	CAAACTGGTAAGCAGAATAAGTTT	TTGACCATTCGTCTTATTCAAAAA	tag143826
novel_sir7291	TGTAGGATCATGTTGCTTAAGTTT	ATCCTAGTACAACGAATTCAAACA	tag66913
novel_sir7292	CTTTTCAGTTGCCACAGTTT	AAAGTCAACGGTGTCAAACA	tag46321
novel_sir7293	TTTTTCAGTTGCCACAGTTT	AAAGTCAACGGTGTCAAACA	tag200702
novel_sir7294	CTTTTTCAGTTGCCACAGTTT	AAAAGTCAACGGTGTCAAATA	tag44432
novel_sir7295	CATTTTCAGTTGCCACAGTTT	AAAAGTCAACGGTGTCAAATA	tag52383
novel_sir7296	ACTTTTCAGTTGCCACAGTTT	AAAAGTCAACGGTGTCAAATA	tag84727
novel_sir7297	TTTTTTCAGTTGCCACAGTTT	AAAAGTCAACGGTGTCAAATA	tag180398
novel_sir7298	ATTTTTCAGTTGCCACAGTTT	AAAAGTCAACGGTGTCAAATA	tag256123
novel_sir7299	ACTTATTCTGCTCACCAGTTT	AATAAGACGAGTGGTCAAACA	tag125931
novel_sir7300	ACTTATTCTGCTCACCAGTTT	AATAAGACGAGTGGTCAAACG	tag125931
novel_sir7301	TTCCGACATCCGGTCAAACG	GGAAGGCTGTAGGCCAGTTT	tag199140
novel_sir7302	GTTTATTCTGCCTGCCAGTTT	AATAAGACGGACGGTCAAACG	tag68444
novel_sir7303	AAATAAGACGGACGGTCAAACG	AGTTTATTCTGCCTGCCAGTTT	tag265404
novel_sir7304	AAAAGTCAACGGCGTCAAACA	TTTTTTCAGTTGCCGCAGTTT	tag81919
novel_sir7305	AAAAGTCAACGGCGTCAAACA	TCTTTTCAGTTGCCGCAGTTT	tag99151
novel_sir7306	AAAAGTCAACGGCGTCAAACA	TGTTTTCAGTTGCCGCAGTTT	tag101114
novel_sir7307	AAAAGTCAACGGCGTCAAACA	CTTTTTCAGTTGCCGCAGTTT	tag186500
novel_sir7308	GCTTAGTCTTGGAAACGTCAAAGT	AACGAATCAGAACCTTTGCAGTTT	tag203050
novel_sir7309	CCATCTAATTTGGCTAGTCAAACT	GAGGTAGATTAAACCGATCAGTTT	tag23010

novel_sir7310	GTGGGACGAAGACATAGTCAAACC	TACACCCTGCTTCTGTATCAGTTT	tag183709
novel_sir7311	GGACGCAGCGGCGCGAGTCAAAAG	TACCTGCGTCGCCGCGCTCAGTTT	tag44724
novel_sir7312	GTCAGATGAAGACCGAGTCAAATT	TGCAGTCTACTTCTGGCTCAGTTT	tag147992
novel_sir7313	AGGCAAGACCACAGTCAAATG	CATCCGTTCTGGTGTCAGTTT	tag10178
novel_sir7314	TAGGCAAGACCACAGTCAAATG	GCATCCGTTCTGGTGTCAGTTT	tag225373
novel_sir7315	TTTGCAACGTTCGCTTTTCAGTTT	ACGTTGCAAGCGAAAAGTCAAAGC	tag126193
novel_sir7316	GACAATCGAAGCCGGTGAGAGTTT	GTTAGCTTCGGCCACTCTCAAAAA	tag194626
novel_sir7317	CGGGCCTAGTTGTTATCTCAAACG	CTGCCCGGATCAACAATAGAGTTT	tag1649
novel_sir7318	GTTACCGATGACATATCTCAAACT	TACAATGGCTACTGTATAGAGTTT	tag253642
novel_sir7319	CACTAGCCCAAATTGCAAAGG	CGGTGATCGGGTTTAACGTTT	tag38053
novel_sir7320	TGTTTCTCGGCCTCTGCAAAAG	GCACAAAGAGCCGGAGACGTTT	tag133739
novel_sir7321	TACTATATACCCAGATCACCGTTT	GATATATGGGTCTAGTGGCAAATC	tag79489
novel_sir7322	TACTGTACACCCGGATCACCGTTT	GACATGTGGGCCTAGTGGCAAATT	tag30334
novel_sir7323	TCTTTAAGTTCGCTGGCAAACC	ACAGAAATTCAAGCGACCGTTT	tag111
novel_sir7324	ATACAGAAATTCAAGCGACCGTTT	TGTCTTTAAGTTCGCTGGCAAACC	tag196924
novel_sir7325	TGGTAGAAACCAGCCAGGCCGTTT	CATCTTTGGTCGGTCCGGCAAAAT	tag234733
novel_sir7326	TAGTAGAAACCAGCCAGGCCGTTT	CATCTTTGGTCGGTCCGGCAAAAT	tag289223
novel_sir7327	GTACTCTGCGCAGTGCCGTTT	TGAGACGCGTCACGGCAAAGC	tag121645
novel_sir7328	CGGACTCGGAATCAGGCAAATG	ATGCCTGAGCCTTAGTCCGTTT	tag28802
novel_sir7329	TGACTGGACTGCCACGCGCAAAAC	CGACTGACCTGACGGTGCGCGTTT	tag123919
novel_sir7330	GAACGGTACTCGAAGGCGTTT	TGCCATGAGCTTCCGCAAACA	tag124654
novel_sir7331	TGAACGGTACTCGAAGGCGTTT	TTGCCATGAGCTTCCGCAAACA	tag273621
novel_sir7332	GGAACGGTACTCGAAGGCGTTT	TTGCCATGAGCTTCCGCAAACA	tag283855
novel_sir7333	GTGGTCAAACGTTGCAAGCAAAAA	TTCACCAGTTTGCAACGTTCGTTT	tag292584
novel_sir7334	CTCACCAGTTTGCAATGTTCGTTT	GTGGTCAAACGTTACAAGCAAAAA	tag92861
novel_sir7335	TTCACCAGTTTGCAATGTTCGTTT	GTGGTCAAACGTTACAAGCAAAAA	tag147448
novel_sir7336	TAACATAGTAGGGCCAGGTTT	TGTATCATCCCGGTCCAAAGC	tag129875
novel_sir7337	TTAACTCCAGTGCAGGTCCAAAGC	CGAATTGAGGTCACGTCCAGGTTT	tag145240
novel_sir7338	CAGCGAGACTGCCGAGGCAGGTTT	CGCTCTGACGGCTCCGTCCAAAAT	tag201359
novel_sir7339	CAGTCGCAGGCTAAAAAGAGGTTT	CAGCGTCCGATTTTTCTCCAAACC	tag289319
novel_sir7340	TGACTGAAGTTTGGATCCAAACT	TTACTGACTTCAAACCTAGGTTT	tag7868
novel_sir7341	GGACTGAAGTTTGGATCCAAACT	TTCCTGACTTCAAACCTAGGTTT	tag93753
novel_sir7342	AGGACTGAAGTTTGGATCCAAACT	TTTCCTGACTTCAAACCTAGGTTT	tag82539
novel_sir7343	TTTCCTGACTTCAAACCTAGGTTT	AGGACTGAAGTTTGGATCCAAACC	tag82539

novel_sir7344	AGGACTGAAGTTTGGATCCAAACA	TTTCCTGACTTCAAACCTAGGTTT	tag82539
novel_sir7345	GCTAGGCATCGGGGTAGGTTT	ATCCGTAGCCCCATCCAAATA	tag37790
novel_sir7346	CGGGCTAGGCATCGGGGTAGGTTT	CCGATCCGTAGCCCCATCCAAATA	tag215814
novel_sir7347	TAGATGCAAATCAAACAACGGTTT	CTACGTTTAGTTTGTTGCCAAATT	tag211561
novel_sir7348	CCGATGTAAATCAAACGACGGTTT	CTACATTTAGTTTGCTGCCAAATT	tag251181
novel_sir7349	CTATTTTCTTAGGCATGCCAAAAT	CGGATAAAAGAATCCGTACGGTTT	tag198440
novel_sir7350	ATATTCTGTATAACACGCCAAAAA	CATATAAGACATATTGTGCGGTTT	tag86284
novel_sir7351	GGACATGACCATTGGAGCCAAATG	CGCCTGTACTGGTAACCTCGGTTT	tag601
novel_sir7352	AGACATGATCCTTGAAGCCAAATG	AGTCTGTACTAGGAACTTCGGTTT	tag96615
novel_sir7353	TGTAGCATGGTTAAAGTTGGGTTT	ATCGTACCAATTTCAACCCAAACT	tag282796
novel_sir7354	ACAACTGTGGCAGTGAACCAAACA	GGTGTTGACACCGTCACTTGGTTT	tag131488
novel_sir7355	GGTGTGAATACCTTCGGTTGGTTT	ACACTTATGGAAGCCAACCAAACA	tag123056
novel_sir7356	GCAGTGTGAGTAAACCAAATC	GACGTCACACTCATTTGGTTT	tag30666
novel_sir7357	ACTGAGCCTGCCGTTGATATGTTT	ACTCGGACGGCAACTATACAAATC	tag48533
novel_sir7358	CACCAGATTCAGATTCCTTTATGTTT	GGTCTAAGTCTAAGGAAATACAAAAA	tag223998
novel_sir7359	TACTGTACACCCAGGTCACTGTTT	GACATGTGGGTCCAGTGACAAATA	tag151088
novel_sir7360	ACTGTATAACATCTTGTACTGTTT	ACATATTGTAGAACATGACAAAGT	tag21449
novel_sir7361	TATTGGTCTGACTCGGTGTTT	AACCAGACTGAGCCACAAACT	tag242218
novel_sir7362	ATATTGGTCTGACTCGGTGTTT	TAACCAGACTGAGCCACAAACT	tag162048
novel_sir7363	TGGTCCAAAGTGCAACAAAGT	CCACCAGGTTTCACGTTGTTT	tag273252
novel_sir7364	TACTTGCCAGTTTGTAATTTGTTT	GAACGGTCAAACATTAAACAAATA	tag238551
novel_sir7365	GCTTTTCAGATTCGGATTTGTTT	AAAAGTCTAAGCCTAAACAAAGA	tag110817
novel_sir7366	AAAAGTCTAAGCCTAAACAAACA	GCTTTTCAGATTCGGATTTGTTT	tag110817
novel_sir7367	AAAAGTCTAAGCCTAAACAAACT	GCTTTTCAGATTCGGATTTGTTT	tag110817
novel_sir7368	CTATATCCTATAACCAAACAAAAT	AGGATATAGGATATTGGTTTGTTT	tag125751
novel_sir7369	GCTTTTCAGATTCGGTTTTGTTT	AAAAGTCTAAGCCAAAACAAACA	tag136300
novel_sir7370	TGCTTGCCAGTTTGTAAAAATTTT	GAACGGTCAAACATTTTTAAAACA	tag70698
novel_sir7371	GAACGGTCAAACATTTTTAAAAAA	TGCTTGCCAGTTTGTAAAAATTTT	tag70698
novel_sir7372	GAACGGTCAAACATTTTTAAAAAG	TGCTTGCCAGTTTGTAAAAATTTT	tag70698
novel_sir7373	TTTGCCAGTTTGTACAAATTTT	ACGGTCAAACATGTTTAAAAAA	tag149590
novel_sir7374	CTTGCCAGTTTGTACAAATTTT	ACGGTCAAACATGTTTAAAAAA	tag188726
novel_sir7375	TACTTGCCAGTTTGTATAAATTTT	GAACGGTCAAACATATTTAAAAAA	tag53788
novel_sir7376	TGCTTGCCAGTTTGTATAAATTTT	GAACGGTCAAACATATTTAAAAAA	tag251856
novel_sir7377	ACGTCCACTCGAGCTGTTAAAACA	CCTGCAGGTGAGCTCGACAATTTT	tag57372

novel_sir7378	ACGGCGTCATCTATTAAAAAA	ATTGCCGCAGTAGATAATTTT	tag131514
novel_sir7379	AGTGGGCTTTAGTTCTGGCATTTT	ACCCGAAATCAAGACCGTAAAACT	tag162588
novel_sir7380	AAGAGTTTTCACCAAAGCGATTTT	CTCAAAAGTGGTTTCGCTAAAATG	tag168350
novel_sir7381	TGCGCATGCGGTGTATCCTATTTT	GCGTACGCCACATAGGATAAAACC	tag213325
novel_sir7382	AGCTCTAGCACCGGCGATAAAAAC	TCTCGAGATCGTGGCCGCTATTTT	tag286816
novel_sir7383	TTAAAGAATTCAGCAGGCTATTTT	TTTCTTAAGTCGTCCGATAAAAGT	tag52098
novel_sir7384	TGTTATCAGGGCCTACGTTATTTT	AATAGTCCCGGATGCAATAAAAAC	tag19676
novel_sir7385	TCAGTTGCCACAGTTTATTTT	TCAACGGTGTCAAATAAAACG	tag186928
novel_sir7386	TACGTGGCAAATCGGCAAACTTTT	GCACCGTTTAGCCGTTTGAAAAGC	tag130644
novel_sir7387	CGTGGCAAATCGTCAAACTTTT	ACCGTTTAGCAGTTTGAAAAGC	tag268860
novel_sir7388	CAGTAGCGCGAACCAACTTTT	CATCGCGCTTGGTTGAAAAGC	tag258661
novel_sir7389	CCAGTAGCGCGAACCAACTTTT	TCATCGCGCTTGGTTGAAAAGC	tag202663
novel_sir7390	ATTAGAGTTCACATGAAAAAG	AGTAATCTCAAGTGTACTTTT	tag126973
novel_sir7391	TCAATATGAATGTGGAAAATG	GTAGTTATACTTACACCTTTT	tag185856
novel_sir7392	GGCAGCACCGCGGATCGCCCTTTT	GTCGTGGCGCCTAGCGGGAAAATT	tag193846
novel_sir7393	TACCAGTTTCAACCCGCGCCTTTT	GGTCAAAGTTGGGCGCGGAAAACC	tag4097
novel_sir7394	TGCCAGTTTCAACCCGCGCCTTTT	GGTCAAAGTTGGGCGCGGAAAACC	tag4622
novel_sir7395	GGTTAAAGTTGGACGCGGAAAACC	TACCAATTTCAACCTGCGCCTTTT	tag292908
novel_sir7396	TGCTAATTTCAACCTGCGCCTTTT	GATTAAAGTTGGACGCGGAAAACC	tag5503
novel_sir7397	TACCAGTTTCAACCTGCGCCTTTT	GGTCAAAGTTGGACGCGGAAAACT	tag15466
novel_sir7398	TGCCAGTTTCAACCTGCGCCTTTT	GGTCAAAGTTGGACGCGGAAAACT	tag94351
novel_sir7399	TACTAGTTTCAACCTGCGCCTTTT	GATCAAAGTTGGACGCGGAAAATC	tag31053
novel_sir7400	TACTAGTTTCAACCTGCGCCTTTT	GATCAAAGTTGGACGCGGAAAACT	tag31053
novel_sir7401	GATCAAAGTTAGACGCGGAAAACC	TACTAGTTTCAATCTGCGCCTTTT	tag105216
novel_sir7402	TGTTAATTTCATCTTGTGCCTTTT	AATTAAAGTAGAACACGGAAAACT	tag106834
novel_sir7403	GGCAAACTAACTCGGCGTCCTTTT	GTTTGATTGAGCCGCAGGAAAAAC	tag252118
novel_sir7404	GGCAAACTAACCTGGCGTCCTTTT	GTTTGATTGGACCGCAGGAAAAAC	tag221872
novel_sir7405	GGCAAACTAATCTGGCGTCCTTTT	GTTTGATTAGACCGCAGGAAAAAT	tag110331
novel_sir7406	GACAAACTAATCTGGCGTCCTTTT	GTTTGATTAGACCGCAGGAAAAAT	tag235936
novel_sir7407	TCTCCTAACGTTTTGTGTCCTTTT	AGGATTGCAAAACACAGGAAAAAC	tag47084
novel_sir7408	TATTCGGTTGAATATTCAGCTTTT	AAGCCAACTTATAAGTCGAAAAGT	tag58720
novel_sir7409	GAACTCGCCGCGGACGCTTTT	TGAGCGGCGCCTGCGAAAATG	tag92130
novel_sir7410	TCAAACTTTTCGCACGGTGCTTTT	TTTGAAAAGCGTGCCACGAAAATC	tag206030
novel_sir7411	GCTTACCAGTTTGTACACTCTTTT	AATGGTCAAACATGTGAGAAAAAG	tag190396

novel_sir7412	TGAGTGACGCAAGAGAAAAGC	CCACTCACTGCGTTCTCTTTT	tag12777
novel_sir7413	TGGCACGGAACGAAGAAAATT	TCACCGTGCCTTGCTTCTTTT	tag172580
novel_sir7414	CTCACCAGTTTGTCACGTTCTTTT	GTGGTCAAACAGTGCAAGAAAAAC	tag70832
novel_sir7415	CATGAGCACGACTTTCAAAACC	CGGTACTCGTGCTGAAAGTTTT	tag167049
novel_sir7416	CCGTTCGTCTTATTCAAAAAC	TTGGCAAGCAGAATAAGTTTT	tag239983
novel_sir7417	CTGGTAAGCAGAATAAGTTTT	CCATTCGTCTTATTCAAAAAT	tag46953
novel_sir7418	TTGGTAAGCAGAATAAGTTTT	CCATTCGTCTTATTCAAAAAT	tag115781
novel_sir7419	CTTAAGAAGTCCGCTTGCAAAAAT	CGGAATTCTTCAGGCGAACGTTTT	tag233718
novel_sir7420	TGCAACTTGCACTTATCACGTTTT	GTTGAACGTGAATAGTGCAAAACT	tag239247
novel_sir7421	TTTCTCGGCCTCTGCAAAAGA	ACAAAGAGCCGGAGACGTTTT	tag65172
novel_sir7422	TTCCAAGATTCGTGCTGCAAAATG	ACAAGGTTCTAAGCACGACGTTTT	tag158620
novel_sir7423	GGTGAATTCCTCGGCGTACGTTTT	ACTTAAGGAGCCGCATGCAAAAAT	tag38033
novel_sir7424	ATCTTTGGTCGGTCCGGCAAAATC	GGTAGAAACCAGCCAGGCCGTTTT	tag15196
novel_sir7425	TTAGATTCGTCTCGCAAAATA	GTAATCTAAGCAGAGCGTTTT	tag168272
novel_sir7426	AATGGGCGACCGTTTCTGCGTTTT	ACCCGCTGGCAAAGACGCAAAAGT	tag88467
novel_sir7427	GTGTGCTAAACCGCAACTCGTTTT	CACGATTTGGCGTTGAGCAAAAGG	tag244431
novel_sir7428	ACCTAAGTGACCGTCAGCAAAAGT	CGTGGATTCACTGGCAGTCGTTTT	tag38392
novel_sir7429	TCACCAGTTTGCAACGTTCGTTTT	TGGTCAAACGTTGCAAGCAAAAAC	tag56
novel_sir7430	TAAAGCTAGCCTTAAAACGGTTTT	TTCGATCGGAATTTTGCCAAAACG	tag49100
novel_sir7431	ACCAAAATTAGTCATGCCAAAACT	AATGGTTTTAATCAGTACGGTTTT	tag161234
novel_sir7432	AATGGTTTTAATCAGTACGGTTTT	ACCAAAATTAGTCATGCCAAAATT	tag161234
novel_sir7433	TATGCCGACTACCTGTGATGTTTT	ACGGCTGATGGACACTACAAAAAT	tag28123
novel_sir7434	TGTGCCGACTACCTGTGATGTTTT	ACGGCTGATGGACACTACAAAAAT	tag210652
novel_sir7435	TATGCCGACTACCTGTGATGTTTT	ACGGCTGATGGACACTACAAAATT	tag28123
novel_sir7436	TGTGCCGACTACCTGTGATGTTTT	ACGGCTGATGGACACTACAAAATT	tag210652
novel_sir7437	TATGCCGACTACCTGTGATGTTTT	ACGGCTGATGGACACTACAAAAAA	tag28123
novel_sir7438	TGTGCCGACTACCTGTGATGTTTT	ACGGCTGATGGACACTACAAAAAA	tag210652
novel_sir7439	ACGGTCAAATATTTTTTAAAAAAT	CTTGCCAGTTTATAAAAAATTTTT	tag241177
novel_sir7440	TTTGCCAGTTTGTATAAAATTTTT	ACGGTCAAACATATTTTAAAAAAT	tag281748
novel_sir7441	ACGGTCAAACATGTTTAAAAAAA	CTTGCCAGTTTGTACAAATTTTT	tag82989
novel_sir7442	CTTGCCAGTTTGTACAAATTTTT	ACGGTCAAACATGTTTAAAAAGG	tag82989
novel_sir7443	TTTGCCAGTTTGTACAAATTTTT	ACGGTCAAACATGTTTAAAAAGG	tag205218
novel_sir7444	ACGGTCAAACATGTTTAAAAAAG	CTTGCCAGTTTGTACAAATTTTT	tag82989
novel_sir7445	CAGTTGCCGCAGTAGATAATTTTT	CAACGGCGTCATCTATTAAAAAAC	tag20673

novel_sir7446	TGGCAACACAGTGTAAAAATC	GTACCGTTGTGTCACATTTTT	tag233100
novel_sir7447	CACCGGTTTGCAACATTCATTTTT	GGCCAAACGTTGTAAGTAAAAACT	tag291431
novel_sir7448	ACGGTCAAACATGTGCTAAAAAGT	CTTGCCAGTTTGTACACGATTTTT	tag30323
novel_sir7449	GCGGTCAAACATATGCTAAAAAGT	TTCGCCAGTTTGTATACGATTTTT	tag60727
novel_sir7450	ACGGTCAAACATATGCTAAAAAGT	CTTGCCAGTTTGTATACGATTTTT	tag122605
novel_sir7451	ACGGTCAAACATATGCTAAAAAGT	TTTGCCAGTTTGTATACGATTTTT	tag156131
novel_sir7452	ACGGTCAAACATATGCTAAAAAAT	CTTGCCAGTTTGTATACGATTTTT	tag122605
novel_sir7453	ACGGTCAAACATATGCTAAAAAAT	TTTGCCAGTTTGTATACGATTTTT	tag156131
novel_sir7454	ACAAACAGAGACACGGTGATTTTT	TTTGTCTCTGTGCCACTAAAAAGT	tag55086
novel_sir7455	ATCCTAGTCAGTCAAATAAAAAAC	CGTAGGATCAGTCAGTTTATTTTT	tag67693
novel_sir7456	GGATTTACCGACCTCCAACTTTTT	TAAATGGCTGGAGGTTGAAAAAAC	tag86667
novel_sir7457	CGTTGGTTTGTGATCGGACTTTTT	AACCAAACACTAGCCTGAAAAATT	tag31010
novel_sir7458	TATCGGTTTGCAATCTTCCTTTTT	AGCCAAACGTTAGAAGGAAAAAGT	tag34821
novel_sir7459	GTTCAAAGTTGGAAGCGAAAAACC	TGCAAGTTTCAACCTTCGCTTTTT	tag195557
novel_sir7460	GGTCAAAGTTGGGTACGAAAAACC	TGCCAGTTTCAACCCATGCTTTTT	tag25379
novel_sir7461	GGTCAAAGTTGGGCACGAAAAACC	CGCCAGTTTCAACCCGTGCTTTTT	tag61443
novel_sir7462	ATGGTCAAACATGTGAGAAAAAGT	CTTACCAGTTTGTACACTCTTTTT	tag7488
novel_sir7463	GTAAGCAGAATAAGTTTTT	TTCGTCTTATTCAAAAATT	tag154667
novel_sir7464	AATGCGCCGTAATCTTAAGTTTTT	ACGCGGCATTAGAATTCAAAAAAT	tag228112
novel_sir7465	CTTACCAGTTTGCAGTCAGTTTTT	ATGGTCAAACGTCAGTCAAAAAGT	tag194692
novel_sir7466	GGTCAAACGTTGCAAGCAAAAACT	TACCAGTTTGCAACGTTCGTTTTT	tag228548
novel_sir7467	CAGTTTGCAATGTTTGTTTTT	CAAACGTTACAAACAAAAACT	tag96783
novel_sir7468	CTTGCCAGTTTGTAAAAATTTTTT	ACGGTCAAACATTTTTAAAAAAAA	tag283458
novel_sir7469	CTTGCCAGTTTGTAAAAATTTTTT	ACGGTCAAACATTTTTAAAAAAAT	tag283458
novel_sir7470	TTTGCCAGTTTGTACAAATTTTTT	ACGGTCAAACATGTTTAAAAAAGT	tag83094
novel_sir7471	CTTGCCAGTTTGTACAAATTTTTT	ACGGTCAAACATGTTTAAAAAAGT	tag165143
novel_sir7472	ACGGTCAAACATGTTTAAAAAATC	CTTGCCAGTTTGTACAAATTTTTT	tag165143
novel_sir7473	TTTGCCAGTTTGTACAAATTTTTT	ACGGTCAAACATGTTTAAAAAAAA	tag83094
novel_sir7474	CTTGCCAGTTTGTACAAATTTTTT	ACGGTCAAACATGTTTAAAAAAAA	tag165143
novel_sir7475	TTTGCCAGTTTGTACAAATTTTTT	ACGGTCAAACATGTTTAAAAAAAT	tag83094
novel_sir7476	CTTGCCAGTTTGTACAAATTTTTT	ACGGTCAAACATGTTTAAAAAAAT	tag165143
novel_sir7477	ACGGTCAAACATGTTTAAAAAAGC	CTTGCCAGTTTGTACAAATTTTTT	tag165143
novel_sir7478	ACGGTCAAACATATTTAAAAAAAT	TTTGCCAGTTTGTATAAATTTTTT	tag80003
novel_sir7479	ACGGTCAAACATATTTAAAAAAGT	TTTGCCAGTTTGTATAAATTTTTT	tag80003

novel_sir7480	TGTGCCGACTACCTGTGATTTTTT	ACGGCTGATGGACACTAAAAAATG	tag238088
novel_sir7481	GAAGACTATGTCGAAGAAAAAAAT	AGCTTCTGATACAGCTTCTTTTTT	tag13111
