# Supplementary material for: Alternative Polyadenylation Dynamics During the Rice Blast Immune Response
Source: Mol Plant Pathol. 2026 Jun 26;27(7):e70301. doi: 10.1111/mpp.70301 (PMC13305335; doi:10.1111/mpp.70301)
Supplement: Supplementary file 20 — Table S11: Sequences of primers used for synthesising gRNA spacers and genotyping CRISPR‐edited mutants. [file MPP-27-e70301-s006.pdf]

Table S11 Primer sequences used for synthesizing gRNA spacers and genotyping CRISPR-edited mutants

Name	sequence 5’→3’	Purpose
gRNAs- *Os05g0509500*	CCCGCGCGCTGGCGCAGATGAGC	CRISPR/Cas9
Os05g0509500-F	CGATCACGGAGATAAACACTC	Screening of lines
Os05g0509500-R	CCTGTTCTAATCTTCAACTCC	Screening of lines
